# A Canonical Biophysical Model of the CsrA Global Regulator Suggests Flexible Regulator-Target Interactions

**DOI:** 10.1038/s41598-018-27474-2

**Published:** 2018-07-02

**Authors:** A. N. Leistra, G. Gelderman, S. W. Sowa, A. Moon-Walker, H. M. Salis, L. M. Contreras

**Affiliations:** 1 McKetta Department of Chemical Engineering, University of Texas at Austin, 200 E. Dean Keeton St. Stop C0400, Austin, TX 78712 USA; 2 Microbiology Graduate Program, University of Texas at Austin, 100 E. 24th St. Stop A6500, Austin, TX 78712 USA; 3 Biological Sciences Program College of Natural Sciences, University of Texas at Austin, 120 Inner Campus Drive Stop G2500, Austin, TX 78712 USA; 4 Department of Chemical Engineering, Pennsylvania State University, 210 Agricultural Engineering Building, University Park, PA 16802 USA

## Abstract

Bacterial global post-transcriptional regulators execute hundreds of interactions with targets that display varying molecular features while retaining specificity. Herein, we develop, validate, and apply a biophysical, statistical thermodynamic model of canonical target mRNA interactions with the CsrA global post-transcriptional regulator to understand the molecular features that contribute to target regulation. Altogether, we model interactions of CsrA with a pool of 236 mRNA: 107 are experimentally regulated by CsrA and 129 are suspected interaction partners. Guided by current understanding of CsrA-mRNA interactions, we incorporate (i) mRNA nucleotide sequence, (ii) cooperativity of CsrA-mRNA binding, and (iii) minimization of mRNA structural changes to identify an ensemble of likely binding sites and their free energies. The regulatory impact of bound CsrA on mRNA translation is determined with the RBS calculator. Predicted regulation of 66 experimentally regulated mRNAs adheres to the principles of canonical CsrA-mRNA interactions; the remainder implies that other, diverse mechanisms may underlie CsrA-mRNA interaction and regulation. Importantly, results suggest that this global regulator may bind targets in multiple conformations, via flexible stretches of overlapping predicted binding sites. This novel observation expands the notion that CsrA always binds to its targets at specific consensus sequences.

## Introduction

Large-scale omics techniques have been applied with increasing frequency to the study of bacterial post-transcriptional global regulators (e.g., *E*. *coli* Hfq, ProQ, and CsrA), aiming to elucidate the scope of their targets and regulatory effects^1–6^. Results have established that these global regulators can act upon over hundreds of targets. For example, the Hfq global regulator has been implicated as a chaperone for nearly all characterized small RNA (sRNA)-messenger RNA (mRNA) interactions in *E*. *coli*^7,8^. Hfq-RNA interactions are characterized by Hfq binding U-rich sequences in the 3′ portions of sRNAs^3,9–11^ and A-rich sequences in the 5′ untranslated regions (UTRs) of mRNAs^3,12,13^. These motifs are considered specific to the proximal and distal binding faces, respectively, of the Hfq hexamer^12,14^. However, interaction-specific variation is observed, in which sRNAs contact both faces, like ChiX and McaS^15,16^. The impact of such variation in binding on target control and network regulation is still unfolding.

For the case of CsrA, approximately 800 mRNA have been identified across multiple environmental conditions as potentially interacting with CsrA^2,5^: this total approaches 20% of the *E*. *coli* genome. Generally, the CsrA homodimer binds a target mRNA at two copies^17^ of a consensus sequence (ANGGA)^18^, preferentially located in the loop of a hairpin structure^18,19^. However substantial variation in these traits is observed. For example, among the 31 classical and well-characterized targets of CsrA (defined in Table 1), mRNAs like *pgaA* may hold to the general pattern^20^, but *clpB*, *dps*, *patA*, and *purM* do not present the consensus ANGGA binding motif^5^. Similarly, *hfq* and *ycdT* present only a single copy of the consensus sequence^20–24^ and *cstA*^21^ presents footprinted binding sites outside of the typical stem loop structures that have been shown favorable to CsrA binding.

**Table 1:** mRNAs experimentally regulated by CsrA.

mRNA Target Type	Foot-printed?	Direct Binding?	Regulation of translation?	Literature of mRNA association with CsrA	Number of mRNA modeled	Number of mRNA with Correctly-Predicted Regulation (rep or act)
Classical	Yes	Yes	Yes	Multiple Studies^20,21,23,25,39,42–47^	10	8
Well-characterized	No	Yes	Yes	Multiple Studies^5,6,24^	21	13
Functional	No	No	Yes	Sequence-Search model^40,41^	30	23
				Multi-Omics study^5^	28	17
				Co-IP^2^, HITS-CLIP^5^, or mRNA stability^4^ study	18	5
***Total***					***107***	***66***

The question thus arises of how post-transcriptional global regulators execute hundreds of interactions with targets of variable sequence and structural features while retaining specificity. Although mutation followed by biochemical footprinting, gel shift, and reporter assays are typically used to assess how such features may contribute to regulator-target interactions^25–27^, these are low throughput. For this reason, omics techniques and, in particular, co-immunoprecipitation studies have proven helpful for establishing pools of a post-transcriptional regulator’s potential targets^3,5,28^. These studies, however, cannot assess how specific molecular features of a target mRNA may influence its recognition and control by the regulator.

Thermodynamic modeling approaches offer higher-throughput opportunities to test the impact of specific molecular features hypothesized to be important on regulator-target interactions. For instance, thermodynamic models have been used to characterize the energetics of single post-transcriptional regulator-target, e.g., sRNA-mRNA, interactions and predict energetically likely mRNA targets from bacterial genomes^29^. These models have also begun to incorporate estimation of a regulator’s effect on RNA target expression^30–32^ and to predict local RNA accessibility patterns that indicate potential areas for RNA interactions^33^. Recent advances in thermodynamic modeling of translation initiation^34–37^ have further expanded the ability to calculate a regulator’s impact on the expression of its mRNA targets by bridging molecular interactions with cellular translation.

**Figure 1:**
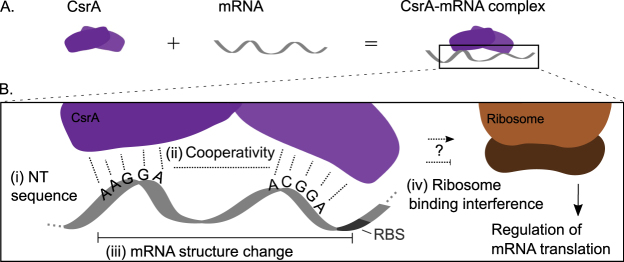
Biophysical considerations for modeling the CsrA post-transcriptional regulator. (**A**) The CsrA protein regulator binds mRNA, preferentially with a stoichiometry of one CsrA homodimer to two binding sites within an mRNA. (**B**) Zooming in on the CsrA-mRNA interaction highlights the biophysical factors that affect it. CsrA preferentially binds (i) ANGGA nucleotide sequences (ii) spaced approximately 10–55 nucleotides apart to support cooperative dual-site binding. The (iii) structure of the mRNA determines how accessible these sequences may be and the energetic cost of CsrA-mRNA binding. Lastly, CsrA bound to the mRNA may (iv) interfere with ribosome binding to regulate mRNA translation.

In this work, we develop, validate, and apply a biophysical, statistical thermodynamic model of CsrA-mRNA targets to investigate how the molecular features of a target mRNA may impact its binding and regulation by CsrA. We employ the *E*. *coli* CsrA protein (Fig. 1A) as a model post-transcriptional regulator given that its binding and regulation of several mRNA targets has been well-characterized. Moreover, we investigate this system because, as described above, its well-characterized mRNA targets demonstrate variance in the sequence and structural features typically considered as hallmarks of CsrA regulatory interactions. This experimentally-demonstrated flexibility in mRNA recognition^17,19,38^ raises questions as to how molecular diversity of target interactions could enable diverse control schemes to support the large proposed scope and known complexity of the CsrA target network^2,5,39^.

Previous models of CsrA-mRNA interactions have focused on genome-wide identification of potential mRNAs bound by CsrA with variations of the ANGGA consensus binding site sequence^18,40,41^. Specifically, work by McKee *et al*. identified *E*. *coli* genes with a [A/C/U]A[A/G/U]GGA[A/G/U][A/C/U] version of the CsrA binding motif within a 22 nucleotide window upstream of their translation start site^40^. Similarly, Kulkarni *et al*. published an algorithm that identified genes containing an A(N)GGA sequence (where N is any or a gap nucleotide) in a window 30 nucleotides upstream to 5 nucleotides downstream of their translation start sites. Additionally, the number and spacing of total available A(N)GGA-like binding sites were considered. It was required that within a window from its transcription start site to the 30th nucleotide of coding sequence, the gene must include at least 3 total A(N)GGA sequences within 10–60 nucleotides of each other or 2A(N)GGA sequences, each with a degenerate GGA-like sequence within a 10 nucleotide distance^41^.

Given the detailed, mechanistic characterizations of several CsrA-mRNA interactions, termed “classical” targets^20,21,23,25,39,42–47^ (defined in Table 1), and the recent expansion of the well-characterized CsrA mRNA target repertoire^5,6^, we expand the previous sequence-based approaches to build a model with greater molecular-level resolution. Specifically, we build a model of canonical CsrA-mRNA binding and regulation by incorporating the biophysical factors characteristic of these known regulatory interactions: mRNA nucleotide sequence^18^, potential cooperativity of two-site binding^17,19^, and minimization of mRNA structural changes. We use these factors to identify and estimate the free energies of an ensemble of CsrA-bound conformations of an mRNA of interest (Fig. 1B, part i–iii). Importantly, these calculations are performed in the context of translation initiation rate calculations^34,35^ to capture the impact of bound CsrA on translation of the mRNA (Fig. 1B, part iv). In this way, our model is able to capture mRNAs expected to be activated, bound but not regulated, or repressed by CsrA binding outside of the Shine-Dalgarno region. Moreover, the current approach enables observation of the effects molecular variations in mRNA features can have on CsrA-mRNA regulation; these variations include mRNA structure and the number, location, spacing, and sequence of potential binding sites.

We employ this model to assess in detail molecular traits that contribute to CsrA regulation of experimentally-verified CsrA-regulated mRNAs that can be described by the canonical model. For this purpose, we analyze probable CsrA-target interactions captured within ensembles of CsrA-bound target conformations. Specifically, we model 236 mRNA: 107 are experimentally-confirmed CsrA-regulated mRNAs (includes the 31 classical and well-characterized targets) that are either repressed or activated in the presence of CsrA (Supplementary Table S1). We conduct detailed molecular analysis of resulting ensembles of CsrA-bound conformations for 66 of these mRNA targets given that their regulation is well-captured by our canonical biophysical model. The model is also applied to 129 mRNAs for which regulation by CsrA is suspected based on results of an integrated omics analysis of the Csr system^5^ or prior literature evidence (summarized in Supplementary Table S1), but has not been experimentally confirmed. Importantly, results suggest that CsrA may bind targets in multiple conformations, allowing use of flexible “binding pockets” that contain a continuous stretch of potential binding sites; this is a novel observation that expands the notion that CsrA always binds to its targets rigidly at a specific five-nucleotide consensus sequence. As such, this work provides insights into molecular features that can be important in understanding ways by which a global post-transcriptional regulator can bind and affect a large pool of cellular mRNAs.

## Results

### Biophysics of CsrA-mRNA Interactions Guide Canonical Model of CsrA Binding and Regulation

**Figure 2:**
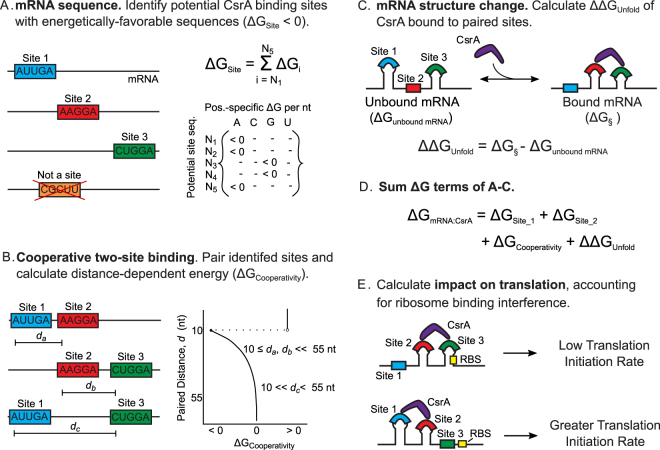
Biophysical, statistical thermodynamic model of CsrA-mRNA interaction and impact on translation. (**A**) Potential CsrA binding sites are identified within an mRNA sequence. A position weight matrix, which quantifies the energy of CsrA binding (ΔG_Site_) to 5 nucleotide-long sequence segments, is used to identify favorable binding sites (ΔG_Site_ < 0). (**B**) To account for cooperativity in CsrA binding to two sites in an mRNA, potential CsrA binding sites are paired and ΔG_cooperativity_ calculated as a function of their distance apart. Sites 10–55 nucleotides apart are rewarded (ΔG_cooperativity_ < 0), and sites less than or equal to 9 nucleotides apart penalized. (**C**) The free energy change due to CsrA binding and altering mRNA structure (ΔΔG_unfold_ ≥ 0) is estimated for each pair of sites. (**D**) The ΔG terms calculated in panels A–C are summed for each pair of sites and (**E**) translation initiation modeled for the top 15 most energetically-favorable bound conformations to determine CsrA-mRNA regulation.

To develop a thermodynamic model of CsrA-mRNA interaction, we considered four biophysical factors understood to govern CsrA binding and regulation, as inferred from studies of classical mRNA targets: consensus sequence (the best-studied aspect of CsrA-mRNA binding), cooperativity, structural change, and ribosome binding ability (Fig. 1B). As such, we term it the “canonical biophysical model”. With respect to nucleotide sequence, we identified plausible five-nucleotide CsrA binding sites in an mRNA of interest based on sequence similarity to the optimal AAGGA binding site sequence previously identified experimentally^17,18^. To do this quantitatively, we derived and employed a position-specific mono-nucleotide free energy model (FEM) that quantifies the energetic contribution of each nucleotide of a five-nucleotide sequence to CsrA-mRNA binding (Fig. 2A) (Methods). Thus the likelihood of CsrA binding to a five-nucleotide sequence, quantified as ΔG_site,_ is the sum of the energetic contributions of each nucleotide. Five-nucleotide sequences with ΔG_site_ < 0 are identified as potential CsrA binding sites.

To address cooperativity in CsrA binding, we quantified the likelihood of two sites within an mRNA being bound by a single CsrA homodimer after identifying all unique pairs of identified potential binding sites. In our model, the cooperativity term (ΔG_cooperativity_) creates a distance-dependent negative free energy for site pairs spaced 10 to 55 nucleotides apart, as this distance was observed to support cooperative binding^17,19^ and has been used in a prior model to represent two-site binding^41^ (Fig. 2B). The ΔG_cooperativity_ term has the greatest energetic contribution when the binding sites are 10 nucleotides apart and decreases to no contribution for sites greater than 55 nucleotides apart (Methods).

All of the unique combinations of two potential binding sites identified in an mRNA of interest constitute an ensemble of the possible conformations in which the CsrA can bind the mRNA at two sites. For each member of this ensemble, we quantified the energetic cost of unfolding the mRNA structure to accommodate CsrA binding (Fig. 2C). The change in free energy, ΔΔG_unfold_, between the unbound mRNA and the mRNA bound to CsrA (as predicted by the ViennaRNA RNA folding package^48^) is determined for each member of the ensemble (Methods).

The sum of the contributions of each individual term described above results in the ability to calculate the total free energy of CsrA-mRNA binding (ΔG_mRNA:CsrA_) for each CsrA-bound mRNA conformation (Fig. 2D):

1 $${{\rm{\Delta }}{\rm{G}}}_{{\rm{mRNA}}:{\rm{CsrA}}}={{\rm{\Delta }}{\rm{G}}}_{{\rm{site}}1}+{{\rm{\Delta }}{\rm{G}}}_{{\rm{site}}2}+{{\rm{\Delta }}{\rm{G}}}_{{\rm{cooperativity}}}+{{\rm{\Delta }}{\rm{\Delta }}{\rm{G}}}_{{\rm{unfold}}}$$

In this model, each CsrA-bound mRNA conformation represents varying optimality of binding site sequence, cooperativity, and structural unfolding. We quantified the probability of CsrA binding to the mRNA of interest in a given conformation as the Boltzmann probability of the system existing in that particular energy state, described by ΔG_mRNA:CsrA_, compared to the energy states of all the other possible conformations (Methods).

To predict the effects of CsrA binding on mRNA translation, we selected the 15 most-likely (lowest energy ΔG_mRNA:CsrA_) CsrA-bound mRNA conformations and modeled the regulatory impact of CsrA on their translation initiation rates, the rate-limiting step of translation, using the RBS Calculator^34,35^ (Fig. 2E). We selected the top 15 conformations, as ranked by high to low Boltzman probability, because this portion represents the majority of the most likely conformations in the distribution of possible CsrA-mRNA interactions. As a point of reference, the 15^th^ most-likely conformation is, on average, 6.0 ± 4.6 fold less likely than the most-likely bound conformation for an mRNA. The fold change in translation for each mRNA target was calculated for each of the 15 lowest energy CsrA-bound conformations relative to the unbound/reference state (Methods). Three possible outcomes result from this calculation: (1) repression of translation (e.g., if CsrA binding directly or indirectly blocks the RBS or start codon), (2) activation of translation (e.g., if CsrA binding leads to increased accessibility of the RBS or start codon), or (3) no impact on translation (e.g., if CsrA binding does not alter translation initiation rate relative to the unbound conformation). It is worth noting that the model-calculated fold change in translation and the type of regulation predicted for each of the top 15 energetically-favorable CsrA-bound conformations of an mRNA can vary in magnitude and in direction given that unique binding site pairs comprise each conformation. Overall, the complete model has only 8 free parameters; 5 parameters quantifying CsrA’s binding affinity to mRNA sites and 3 parameters quantifying the free energy of dimeric CsrA cooperativity. Importantly, free model parameters were fit to experimental measurements prior to performing predictions on full-length mRNA sequences, and no attempt was made to optimize model parameter values.

### Selection of 236 CsrA-mRNA Interactions

We identified a set of 236 mRNAs with known or suspected CsrA interaction and/or regulation and applied the canonical biophysical model to predict how CsrA regulates their translation rates. Specifically, 10 mRNA are classical CsrA targets whose binding sites have been footprinted *in vitro*, direct binding demonstrated *in vitro* or *in vivo*, and regulation demonstrated *in vivo* in prior studies^20,21,23,25,39,42–47^. 21 mRNAs represent well-characterized CsrA targets, whose direct binding has been demonstrated *in vivo* or *in vitro* (without footprinting data) and regulation demonstrated *in vivo*^5,6,24^. Lastly, 76 mRNAs are functional targets, as their regulation by CsrA has been shown *in vivo*, but their direct binding with CsrA has not been confirmed or footprinted. These three types of CsrA targets comprise a set of 107 mRNA that are regulated by CsrA experimentally (Tables 1 and S1).

**Figure 3:**
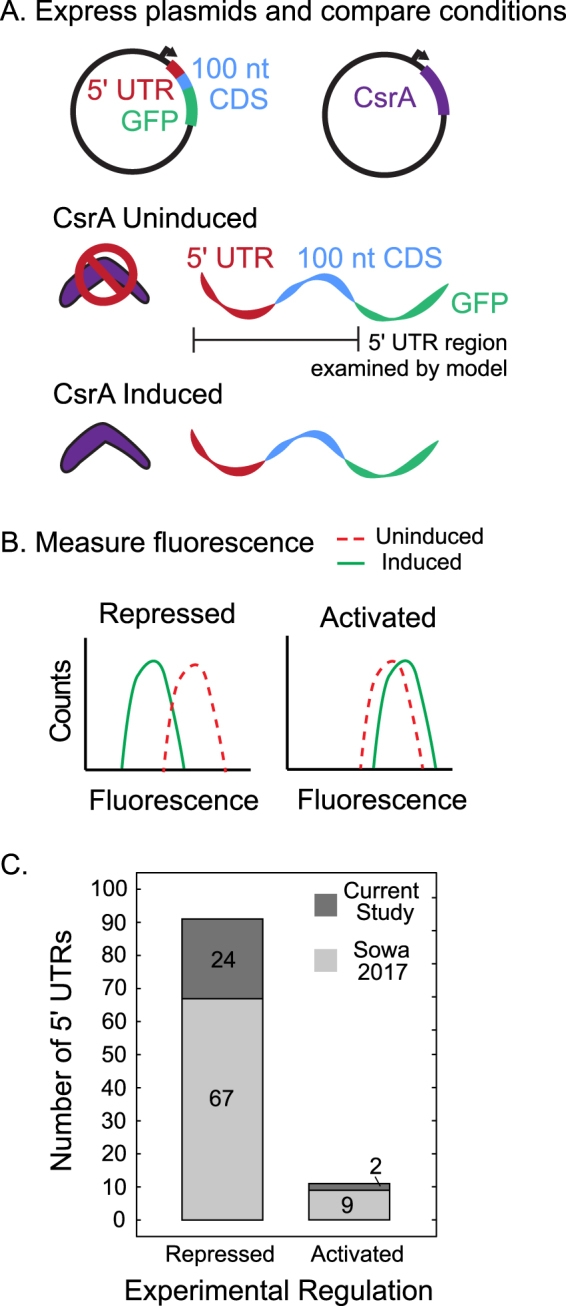
Fluorescent translational reporter assay determines 5′ UTRs repressed and activated in the presence of CsrA. (**A**) Schematic of the fluorescent translational reporter assay. A two plasmid system, one that contains an inducible CsrA and the other that constitutively expresses the 5′ UTR of an mRNA of interest fused to GFP, is used to determine the regulatory relationship of CsrA to the target 5′ UTR. The assay compares 5′ UTR-controlled GFP fluorescence under two conditions: CsrA present (induced condition) and CsrA absent (uninduced condition). (**B**) Significant down- or up-shift in fluorescence upon induction indicates CsrA regulates the 5′ UTR-GFP reporter. (**C**) Bars indicate number of 5′ UTRs which display repression (downshift, P-value < 0.1), or activation (upshift, P-value < 0.1). Bar shading indicates the study the reporter assay results are published in: Sowa *et al*. (lower, light gray) (ref.^5^) or the current work (upper, dark gray).

The majority of the well-characterized and functional CsrA targets were previously tested for CsrA regulation using an *in vivo* fluorescent translational reporter assay (Supplementary Table S1)^5^. This study performed an integrated omics analysis of the Csr system in *E*. *coli* and identified mRNAs likely to be regulated by CsrA; many were tested for CsrA regulation with the *in vivo* assay as follow-up. In the current work, additional mRNAs were tested for CsrA regulation using the same fluorescent translational reporter assay (Fig. 3A,B) (Supplementary Methods, Supplementary Table S2). It is worth acknowledging that for 23 functional targets showing activation or repression by CsrA in the current work, this is their first specific evidence of CsrA regulation. Figure 3 C summarizes results of the fluorescent translational reporter assay across both the initial (Sowa *et al*.)^5^ and the current works. A total of 91 mRNAs showed repression and 11 showed activation by CsrA. It should be noted that the remaining 5 mRNA targets of the 107 experimentally CsrA-regulated mRNAs are classical or well-characterized targets extensively studied in other works^24,39,45,46^ (Supplementary Table S1).

The remaining 129 of the 236 modeled mRNAs were initially identified as potential CsrA-controlled mRNAs in a prior integrated omics study^5^, other transcriptomics-based experimental studies^2,4^, or sequence-search based computational studies^40,41^ (more detail in Supplementary Table S1). We tested the vast majority of these mRNAs (124) for CsrA regulation by fluorescent translational reporter assay (Supplementary Table S2 or in ref.^5^). However, fluorescence results of the 5′ UTR-GFP constructs were inconclusive for various reasons, largely attributed to noise in the fluorescence data, inconsistencies amongst the biological replicas, or low signal indistinguishable from fluorescence background (Supplementary Methods). These are tabulated and regulation indicated as undetermined in Supplementary Table S1.

### Canonical Biophysical Model Captures CsrA Regulation of 66 mRNA Targets

For each of the 236 mRNAs, we extracted its 5′ untranslated region and the first 100 nucleotides of protein coding sequence, and inputted this sequence into the canonical biophysical model to calculate: (i) the locations where CsrA binds, (ii) the predicted CsrA-bound mRNA structures, (iii) the calculated CsrA-mRNA binding free energies for all ensemble conformations (Supplementary Data 1), and (iv) the predicted changes in translation rate for the top (i.e. lowest-energy) fifteen ensemble conformations (Supplementary Table S3). For analysis, we then classified each mRNA as being repressed, activated, or not impacted by CsrA-mediated translation regulation. In most cases, the top fifteen ensemble conformations had similar modes of translation regulation; however, when the most probable ensemble conformations had different equally-likely regulatory modes, we classified these model predictions as heterogeneous (Methods).

To assess model performance, we used the subset of 107 mRNAs known to be regulated by CsrA (10 classical, 21 well-characterized, and 76 functional targets) (Tables 1 and S1). Across the set of 107 mRNAs with known experimental CsrA-mediated regulation, the canonical biophysical model correctly predicted the regulatory modes of 66 mRNAs: 65 of these mRNA have repressed expression, while one is activated (Table 2). It is unsurprising that the canonical biophysical model lacks robust capability for predicting activating CsrA-mRNA interactions given that the biophysical principles used to “train” the model represent the canonical understanding of CsrA interactions with its classical targets. Only one classical target is activated by CsrA, while nine are repressed. This pattern holds for the well-characterized targets (19 repressed and 2 activated) and in Fig. 3C; it is also supported by the understanding that CsrA typically binds in the Shine-Dalgarno region of an mRNA, due to similarities of the consensus CsrA binding site and Shine-Dalgarno sequences, and likely represses translation through direct occlusion of the RBS. The model was best able to correctly classify how CsrA regulates translation when the mRNA contained consensus or near-consensus CsrA binding sites, as in the classical and functional targets identified by sequence search studies (Table 1). Notably here, the model’s formula for calculating ΔG_site_ was parameterized by using binding affinity measurements to consensus CsrA sites with only single nucleotide mutations^18^. Therefore, the model does not account for any non-additive energetic contributions to CsrA’s binding affinity, which could play a role when mRNAs contain non-consensus CsrA sites.

**Table 2:** Experimentally-measured and model-calculated regulation of mRNAs.

Experimental Regulation	Model-Calculated Regulation				*Total*
	Repression	Activation	No Impact	Heterogeneous	
Repression	65	3	11	16	95
Activation	5	1	0	6	12
***Total***	*70*	*4*	*11*	*22*	*107*

Remarkably, for the 41 mRNAs where the model incorrectly predicted regulation, regulation of 22 mRNAs was predicted as heterogeneous: in 21 of 22 cases, regulation of at least 3 of the top 15 most-likely ensemble conformations was correct, but predicted regulation of the ensemble was heterogeneous overall. For 14 mRNAs, repression was missed (3 were predicted as activated and 11 as non-impacted) and for the remaining 5 mRNAs repression was falsely predicted (experimental data showed activation) (Table 2). These results indicate that rather than over-predicting repression, the model does not as readily capture activation mechanisms. More broadly, the 41 mRNAs not captured by the canonical biophysical model might be regulated by CsrA through mechanisms not yet fully described in the literature. Targets for which the model does not clearly predict one regulatory outcome (i.e. heterogeneous regulation) or predicts regulation that does not match experimental results constitute excellent candidates for further exploration of potentially new mechanisms of CsrA post-transcriptional regulation.

### Comparison to Prior CsrA-mRNA Interaction Models

The canonical biophysical model is unique from prior models of CsrA-mRNA interactions in that it (i) calculates a predicted free energy of CsrA-mRNA binding that (ii) incorporates changes in predicted mRNA structure upon CsrA binding. It also (iii) estimates a regulatory outcome of CsrA binding on mRNA translation by calculating a change in estimated translation rate and (iv) predicts *an ensemble* of potential CsrA-bound mRNA conformations and their translation rates. Previous models focused on using consensus binding site sequences to predict high affinity interactions between CsrA and mRNAs across the genome. Importantly, when attempting to predict whether CsrA plays a role in repressing an mRNA’s translation rate, the canonical biophysical model’s accuracy is greater than published sequence-based models^40,41^ (Supplementary Table S4). The difference in accuracy is mainly due to an increased number of correct repression predictions by the canonical biophysical model (65 mRNAs) compared to the McKee and Kulkarni sequence-search approaches (17 and 40 mRNAs, respectively) (Supplementary Table S4 and Supplementary Fig. S1). While the high stringency of the sequence-search approaches is useful for identifying potential targets with a low false positive rate on a genome scale (and avoiding heterogeneous predictions), the greater number of repression predictions provided by the canonical biophysical model is useful for studying mRNAs expected to be canonically regulated by CsrA and for identifying those that could be regulated by alternative mechanisms.

### Validation of Model Binding Site Predictions and Free Energy Calculations

#### Model-identified Binding Sites Align with Known CsrA-mRNA Footprints

**Figure 4:**
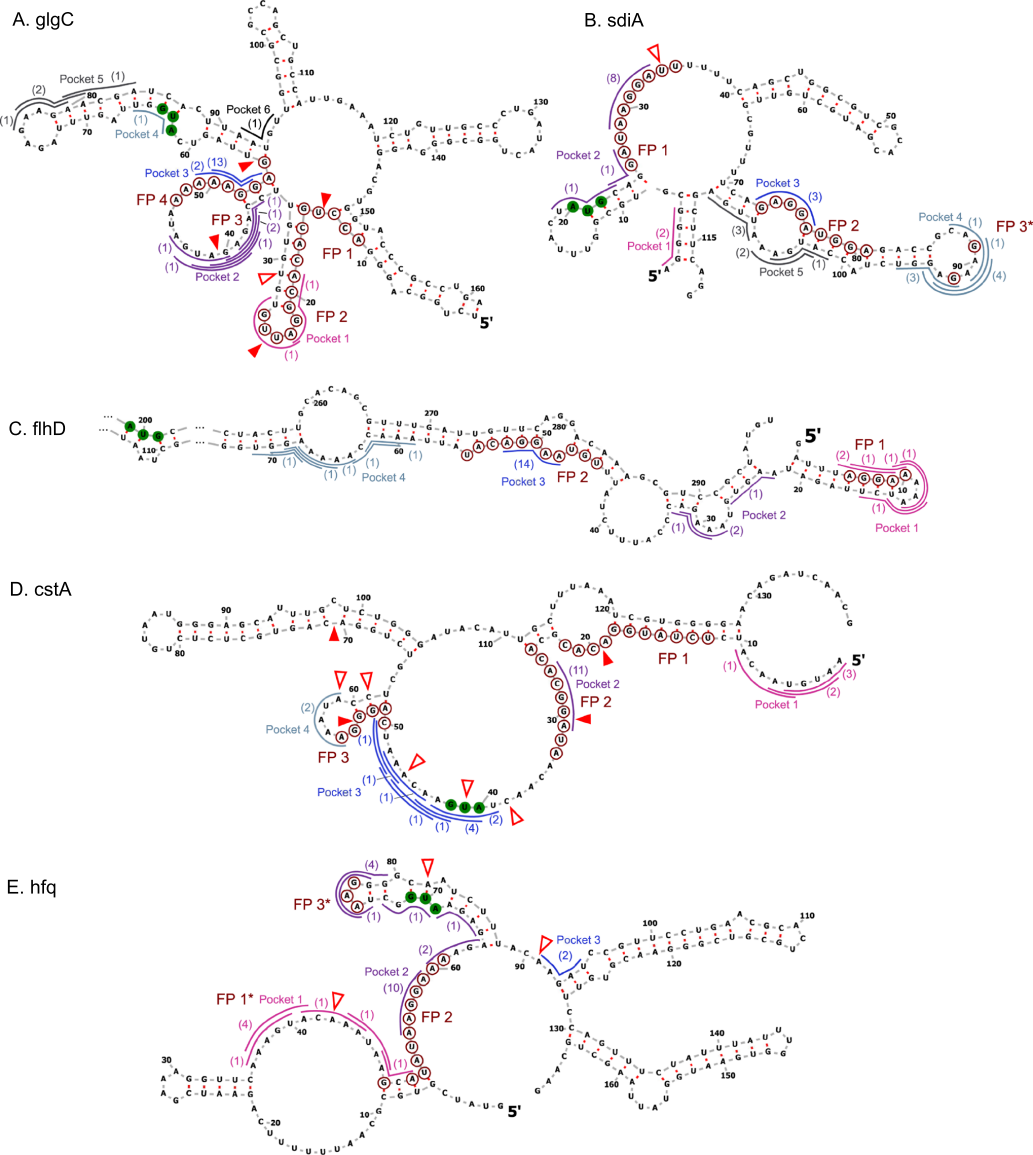
Model binding site predictions capture known CsrA-mRNA footprints. (**A**–**E**) Vienna-RNA predicted structures of eight canonical CsrA-mRNA targets (unbound by CsrA). Model-identified CsrA binding sites of the fifteen most-likely CsrA-bound conformations of each target are shown as colored outlines. Number in parentheses adjacent to the outline indicates the number of times that particular 5-nucleotide binding site is identified in the fifteen most-likely CsrA-bound mRNA conformations. Overlapping or adjacent mRNA binding sites form pockets, indicated by the color of the outline. Start codon nucleotides are shown with green fill. Nucleotides within experimentally-determined *in vitro* footprints are marked with red circular outlines. Red fill wedges indicate 3′ edges of binding sites identified by *in vitro* 3′ boundary analyses; red-outlined wedges indicate 3′ edges of binding sites identified by *in vitro* toeprint analyses. *In vitro*-identified binding sites, regardless of method, are labeled from 5′ to 3′, starting with “FP 1”. *In vitro* binding assays were performed in, (**A**) refs^17,25^; (**B**) ref.^43^; (**C**) ref.^44^; (**D**) ref.^21^; and (**E**) ref.^23^. (**B** and **E**) FP 3 of *sdiA* and FP 1 and FP 3 of *hfq* are marked with an “*” to indicate weaker *in vitro* signals newly interpreted as binding sites. (**D**) Toeprint signals within pocket 3 of *cstA* did not indicate a clear binding site, and thus was not labelled with an “FP”.

**Figure 5:**
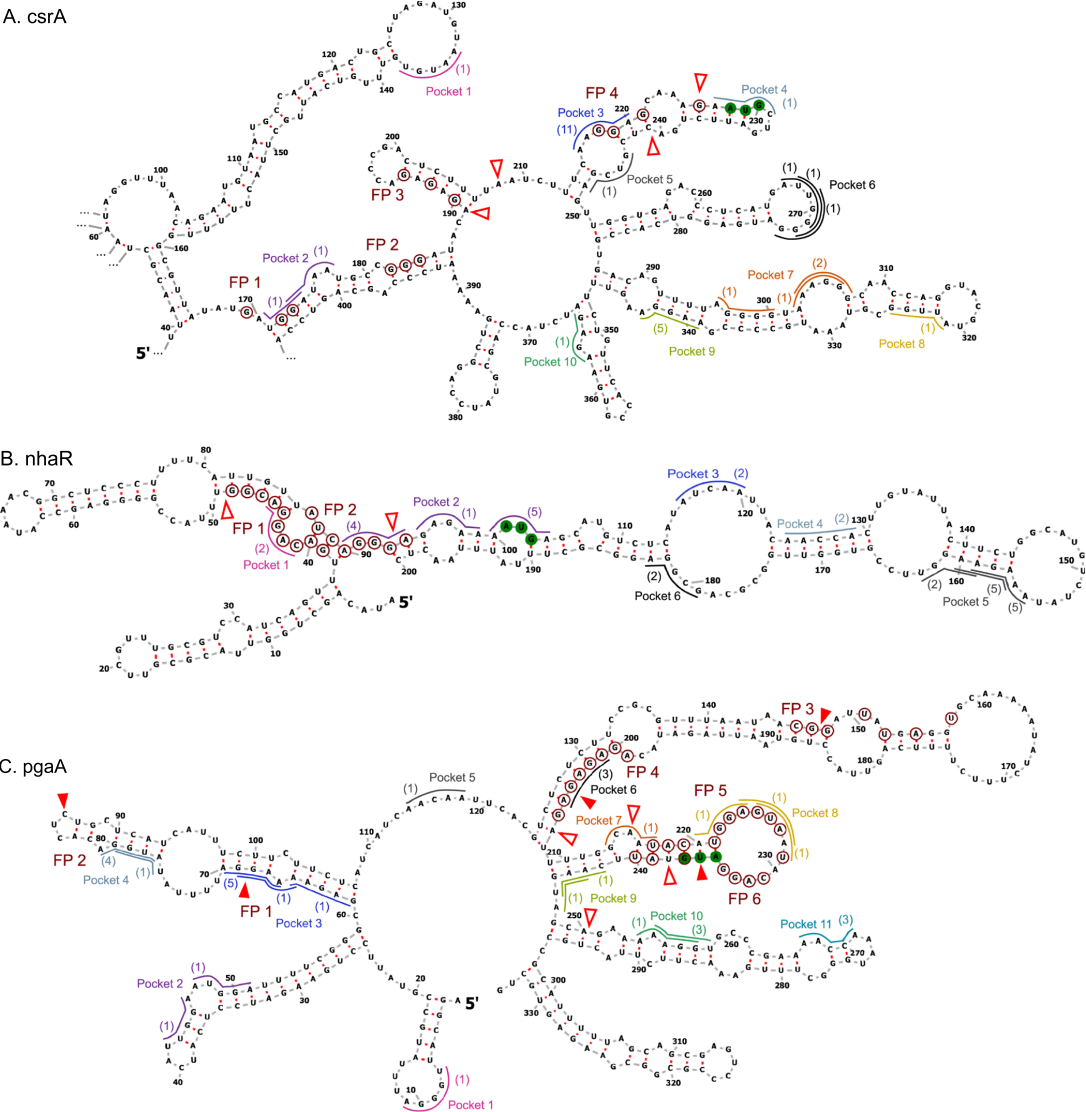
Model binding site predictions capture known CsrA-mRNA footprints. (**A**–**C**) Vienna-RNA predicted structures of eight canonical CsrA-mRNA targets (unbound by CsrA). Model-identified CsrA binding sites of the fifteen most-likely CsrA-bound conformations of each target are shown as colored outlines. Number in parentheses adjacent to the outline indicates the number of times that particular 5-nucleotide binding site is identified in the fifteen most-likely CsrA-bound mRNA conformations. Overlapping or adjacent mRNA binding sites form pockets, indicated by the color of the outline. Start codon nucleotides are shown with green fill. Nucleotides within experimentally-determined *in vitro* footprints are marked with red circular outlines. Red fill wedges indicate 3′ edges of binding sites identified by *in vitro* 3′ boundary analyses; red-outlined wedges indicate 3′ edges of binding sites identified by *in vitro* toeprint analyses. *In vitro*-identified binding sites, regardless of method, are labeled from 5′ to 3′, starting with “FP 1”. *In vitro* binding assays were performed in, (**A**) ref.^47^; (**B**) ref.^42^; and (**C**) ref.^20^.

After establishing that the model correctly predicted CsrA regulatory effects on 66 mRNAs, we assessed if the model captured specific experimentally-determined CsrA-mRNA binding sites. We aimed to validate model predictions by comparing model-identified binding sites with experimentally-determined CsrA footprints in eight classical CsrA targets: *glgC*, *sdiA*, *flhD*, *cstA*, *hfq*, *csrA*, *nhaR*, and *pgaA* (Figs 4 and 5). For most of the classical mRNA targets, the model-identified binding sites of the 15 lowest-energy CsrA-bound conformations align well with at least one of the known footprints (Figs 4A–E and 5A). For this subset of classical targets (Figs 4A–E and 5A), the model identifies binding sites within one of its known footprints at least 9 times among the top 15 ensemble conformations. It is important to note that (i) the same binding site can be identified multiple times, as long as it is paired with a second site that makes the binding site *pair* unique. Additionally, it is noteworthy that (ii) binding sites identified multiple times within the 15 most-likely CsrA-bound conformations of an mRNA adhere more strictly to the ANGGA consensus sequence. Model-predicted binding sites in the *nhaR* and *pgaA* mRNAs (Fig. 5B,C) present exceptions.

Given that overlapping binding sites are commonly identified by the model in the top fifteen CsrA-bound conformations of the classical mRNA targets, we grouped these identified neighboring binding sites into “pockets”. A pocket is defined as a set of contiguous predicted binding sites that overlap, are adjacent, or have, at maximum, one nucleotide between them. Importantly, analysis of predicted binding site pockets within the classical CsrA-mRNA targets indicated that the model can capture CsrA-mRNA interactions of a wide range of *in vivo* affinities. For example, just upstream of the *glgC* Shine-Dalgarno sequence, binding site pocket 2, which contains 7 overlapping binding sites, aligns with a weaker, non-consensus sequence CsrA-mRNA binding site^17^ (FP 3 in Fig. 4A). Similarly, we suspect the presence of weaker, non-consensus sequence binding sites within pocket 4 of *sdiA* (Fig. 4B) and pocket 1 of *hfq* (Fig. 4E), given that these pockets contain 8–9 overlapping non-consensus binding sites. Earlier results from published *in vitro* experiments support this possibility. First, G residues in pocket 4 of *sdiA* may be weakly protected, based on footprint analysis^43^; second, CsrA may bind in pocket 1 of *hfq*, based on toeprint analysis^23^. In any of these cases, consideration of individual binding sites would not have substantially indicated a preference for CsrA to bind in the region. These results indicate that collective analysis of overlapping binding sites as pockets can suggest mRNA regions that bind CsrA. Biophysically, this represents a different concept in that CsrA-mRNA binding may not be strictly defined by rigid consensus sequences, as mostly used in the literature^40,41^. Instead, by capturing binding sites of differential affinities, our model suggests that CsrA binding may be influenced by extended sequences with weak binding affinity (as calculated by the FEM) that *collectively* contribute to CsrA-mRNA binding. In this way, numerous weak, non-consensus sites in close proximity may enhance the strength of the CsrA-RNA interaction at that region by allowing CsrA many opportunities to bind and slide along the stretch of RNA.

#### Calculated ΔG of CsrA-mRNA Binding is Indicative of Relative Affinities Observed *In Vivo*

**Figure 6:**
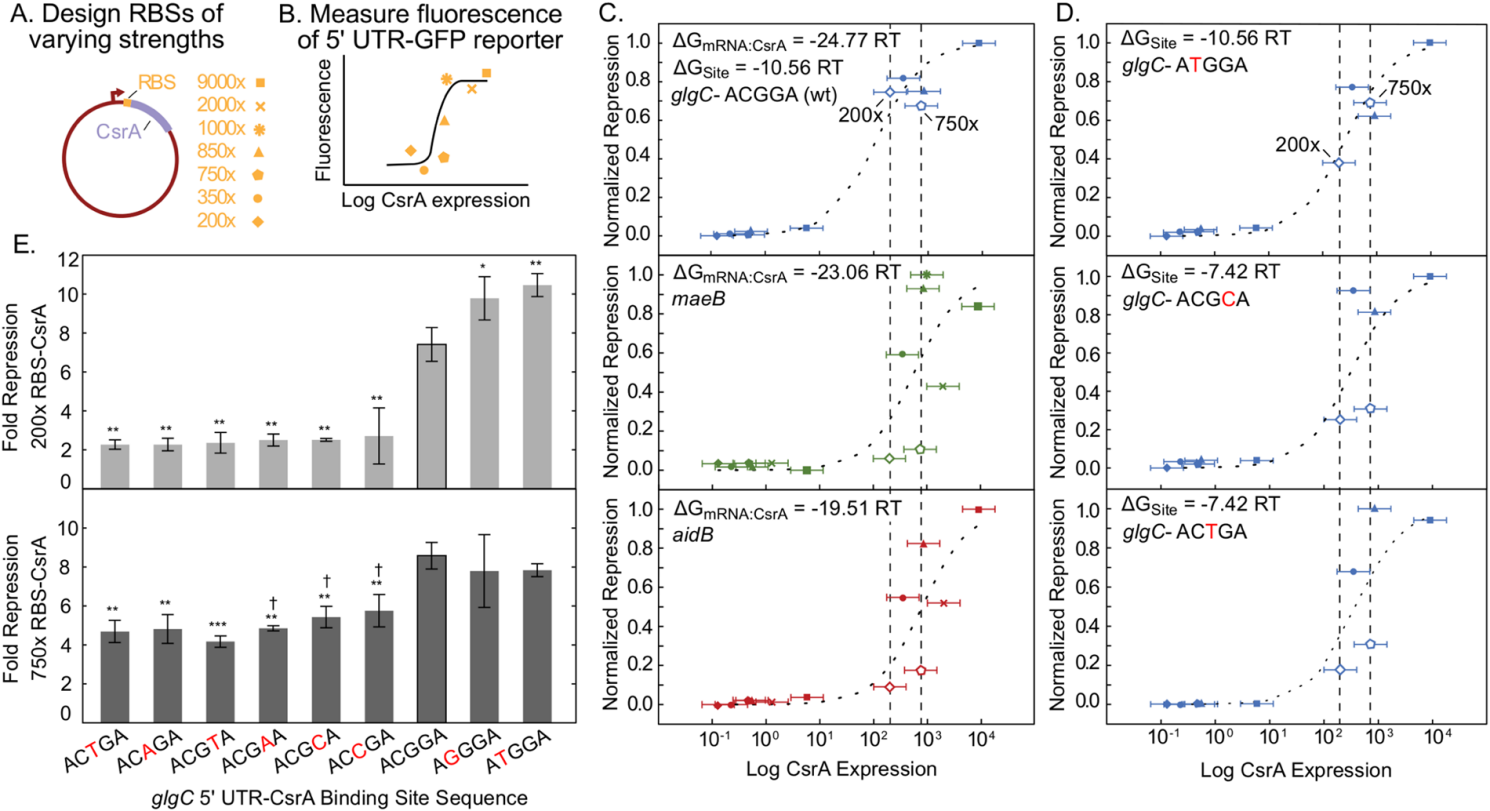
Relative *in vivo* CsrA-mRNA affinity correlates with calculated free energies. (**A**) Using the RBS calculator, multiple RBSs were designed to vary CsrA expression. These RBS-CsrA constructs were paired with target 5′ UTR-GFP reporter plasmids to titrate expression of the reporter and (**B**) fluorescence was measured. (**C**) Titration of *glgC*, *maeB*, and *aidB* 5′ UTRs. Symbols used for each RBS construct are indicated in (**A**). Normalized repression (minimum and maximum repression ratios scaled linearly from 0 to 1) is plotted as function of CsrA expression, as determined by the RBS calculator, on a log scale. Dotted lines represent fit to titration curve (Methods). Induced 200x and 750x RBS-CsrA constructs (open diamond and pentagon symbols, respectively) offer best resolution of differences between 5′ UTRs. 1000x and 2000x RBS constructs produced inconsistent results and were excluded from analysis in some cases. (**D**) Titration, as described for (**C**), of mutated *glgC* sequences. Induced 200x and 750x RBS-CsrA constructs offer best resolution of mutations’ effects. (**E**) The fluorescent translational reporter assay performed with the 200x (upper panel) and 750x (lower panel) RBS-CsrA constructs for the wild type (ACGGA, black outlined bar) and eight mutant *glgC* 5′ UTR-GFP reporters. Asterisks indicate results of heteroscedastic one-tailed T-tests, comparing average fold repression (Uninduced/Induced) of mutants to wild type *glgC*: *P-value < 0.05; **P-value < 0.01; ***P-value < 0.001. (lower panel) Daggers indicate results of heteroscedastic one-tailed T-tests, comparing average fold repression (Uninduced/Induced) of ACTGA, ACAGA, ACGAA, ACGCA and ACCGA constructs to the ACGTA mutant: ^†^P-value < 0.05.

Physiological relevance of the model’s calculated free energy values was assessed by a series of *in vivo* fluorescence assays. As a proxy for measuring CsrA-mRNA binding affinity, we measured extent of CsrA-mRNA regulation using a variation of the fluorescent translational reporter assay. Here, fluorescent expression of a 5′ UTR-GFP reporter is measured in the presence of varying CsrA expression levels, induced by a series of plasmids with varying RBS strength (Supplementary Methods) (Fig. 6A,B). Testing three representative targets, *glgC*, *aidB*, and *maeB*, reveals that CsrA shows increased regulation of *glgC*, relative to *maeB* and *aidB* (Fig. 6C). This is in agreement with the model’s predicted relative binding affinities: *glgC* has the lowest ΔG_CsrA:mRNA_ as compared to the other targets (Table 3). This variation of the fluorescent translational reporter assay was also used to test a versions of the *glgC* 5′ UTR with single nucleotide mutations to a footprinted binding site (Supplementary Methods) (Fig. 6D,E). Results are in general agreement with the FEM used in ΔG_site_ calculations as well as conclusions drawn in previous works^18^. Most importantly, these results collectively support the relevance of predicted free energies, both ΔG_mRNA:CsrA_ and ΔG_site_, to observable differences in *in vivo* CsrA-mRNA binding and regulation at consensus or near-consensus sites.

**Table 3:** Model-calculated free energies of CsrA-mRNA binding, where the ensemble includes the 15 most likely CsrA-bound mRNA conformations.

5′ UTR	ΔG_mRNA:CsrA_ (RT) (most likely conformation)	Ensemble Average ΔG_mRNA:CsrA_ (RT)
*glgC*	−24.77	−21.38
*maeB*	−23.06	−19.35
*aidB*	−19.51	−16.66

### Predicted CsrA-Bound mRNA Conformations Highlight Molecular mRNA Features Expected to Contribute to Regulation

To obtain insights into predicted molecular features of CsrA-mRNA interactions at a large scale, we analyzed modeling results obtained for the pool of 66 targets well captured by the canonical biophysical model. Importantly, we make three major observations that provide insights as to how different molecular features of mRNAs impact CsrA binding and regulation. First, we observe the frequent identification of CsrA binding sites within the Shine-Dalgarno region of the mRNAs: 61% of the 66 mRNA targets contain at least one predicted CsrA binding site overlapping the Shine-Dalgarno region (5–15 nucleotides upstream of the start codon) in their most-likely CsrA-bound conformation (Supplementary Table S3). Notably, 24% of the 66 mRNAs contain a predicted binding site that overlaps its start codon by at least one nucleotide; 14% of the 66 mRNAs fit both criteria (i.e., paired sites that overlap both the Shine-Dalgarno and the start codon are predicted in the most-likely CsrA-bound conformation). The remaining 29% of the 66 mRNAs contain predicted binding sites in other portions of the 5′ UTR in their most-likely bound conformations. The high frequency of binding to the Sine-Dalgarno is expected, given the similarity of the CsrA consensus sequence to the Shine-Dalgarno consensus sequence. While start codons have been identified as a major CsrA binding location in *Salmonella*^3^, they have only recently been implicated in a CsrA-mRNA interaction in *E*. *coli*^39^.

Second, we observed that the model identifies two high affinity binding sites (ANGGN sequences) for binding of a CsrA homodimer in only 13 (20%) of the 66 mRNAs’ most-likely CsrA-bound conformations. Instead, 38 members (58%) present a single high affinity binding site and 15 mRNAs (23%) present no high affinity binding sites (Supplementary Table S3). Given the importance placed on sequence of CsrA binding sites in the literature, the apparent preference towards a single high affinity binding site was unexpected. Counting the number of unique high affinity binding sites identified throughout mRNAs’ ensembles of CsrA-bound conformations reveals that this pattern is not due to a lack of ANGGN sites. 24 (~63%) of the 38 mRNA with a single high affinity site in their most-likely conformation have at least two unique high affinity sites in the modeled sequence that could be identified as a pair (Supplementary Table S3). This suggests that CsrA dimers may preferentially recognize (but not always) two binding sites of varying affinity (i.e. one high, one low), even in the presence of multiple possible high affinity binding sites along the target.

A third observation we made is that 46 mRNAs (70% of the 66 well-captured mRNA) are predicted to have strong cooperativity (ΔG_Cooperativity_ ≤ −4.0 RT, <25 nt apart) between the identified binding sites in their most-likely CsrA-bound conformation, while only 16 (24%) are predicted to have large structural changes (ΔΔG_Structure_ ≥ 3.0 RT) (Supplementary Table S3). Considered alongside our second observation, these results suggest that many mRNAs display a single high affinity site in its most-likely CsrA-bound conformation to (i) minimize free energy changes from structural unfolding and (ii) maximize free energy changes from inter-site cooperativity. Table 4 quantifies this pattern for the 15 lowest-energy CsrA-bound ensemble conformations of each of the 66 well-captured mRNA. CsrA-bound mRNA conformations that present two high affinity ANGGN sequences are significantly depleted for strong inter-site cooperativity and enriched in large structural changes (P-value < 0.01 by hypergeometric test). It should be noted that seven mRNAs present most-likely CsrA-bound conformations that are exceptions to this pattern: *hfq*, *pgaA*, *relA*, *dgcZ*, *deoD*, *tnaA*, and *yfgM*. These targets exhibit two high affinity ANGGN sequences that are a short length apart (strong cooperativity) and require minimal unfolding of mRNA structure to be bound.

**Table 4:** Frequency of types of CsrA-mRNA binding predicted across ensembles.

	Total	Large Structural Change (ΔG ≥ 3 RT)	Strong Inter-site Cooperativity (ΔG ≤ −4 RT)
All Conformations	990	258	518
Conformations with 2 High Affinity Sites	75	49^a^	25^b^
Conformations with 1 High Affinity Site	539	151^c^	286^d^

^a^Hypergeometric test relative to condition in all conformations. P-value = 8 E-14, significantly enriched (P-value < 0.01); ^b^Hypergeometric test P-value = 3 E-4, significantly depleted (P-value < 0.01); ^c^Hypergeometric test P-value = 2 E-2, not significant (P-value ≥ 0.01); ^d^Hypergeometric test P-value = 4 E-2, not significant (P-value ≥ 0.01).

### Patterns in Predicted Binding Site Pockets Suggest Flexibility in CsrA-mRNA Recognition

**Figure 7:**
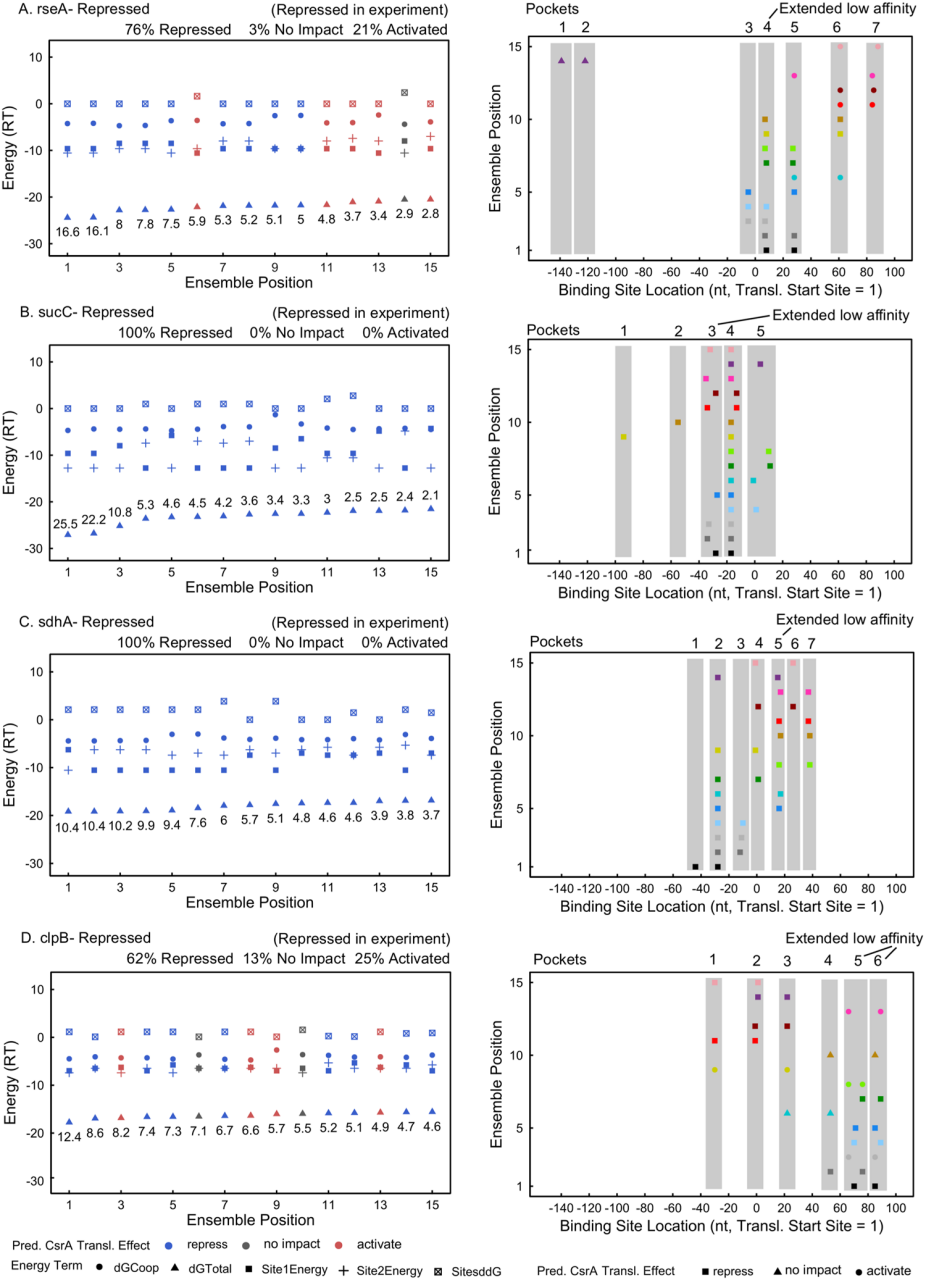
Patterns of binding site frequency within pockets suggest probable strong and weak *in vivo* CsrA-mRNA binding sites. (A, left panel) Free energy terms calculated for the most-likely fifteen members of the ensemble of CsrA-bound *rseA* conformations. Data labels mark the Boltzmann probability of each conformation, scaled such that the total probability of the fifteen most-likely conformations is one hundred percent. Total scaled Boltzmann probabilities of the repressed, not impacted, or activated conformations are noted, as well as the most-likely regulatory outcome as “rseA- Repressed”. Regulation observed in fluorescent translational reporter assay is noted in parentheses as “Repressed in experiment”. (A, right panel) Distribution of binding sites (by location) predicted in *rseA*. Gray panels highlight pockets of binding sites. Seven pockets are identified, one of which, pocket 4, contains 7–10 non-consensus low affinity predicted binding sites. (**B**) 5 pockets, one of extended low CsrA affinity are predicted for *sucC*. (**C**) 7 pockets, one of extended low CsrA affinity, are predicted for *sdhA*. (**D**) 6 pockets, two of extended low CsrA affinity are predicted for *clpB* in the absence of any high affinity binding sites.

We next analyzed in detail the fifteen most-likely CsrA-bound conformations predicted for each of the 66 mRNAs captured by the canonical model. Specifically, we looked to determine mRNAs in which CsrA binding could be influenced by extended sequences of weak binding affinity that may collectively contribute to CsrA-mRNA binding as calculated for *glgC*, *sdiA*, and *hfq* above. This was executed by identifying and analyzing pockets of predicted binding sites in each mRNA (Supplementary Table S3) and mapping predicted free energy terms across their most-likely fifteen CsrA-bound conformations (Supplementary Fig. S2). We counted pockets with 7–10 predicted non-consensus binding sites (i.e., not ANGGN sequences) as potential extended regions of low affinity CsrA interactions given that this number of overlapping weak binding sites was predicted in *glgC*, *sdiA* and *hfq*. Importantly, 17 of the 66 mRNA well-captured by the canonical biophysical model (26%) contain a pocket of 7–10 predicted non-consensus binding sites; we propose that these extended regions of low affinity may collectively contribute to CsrA binding and regulation. Some mRNAs in this category, like *rseA*, *sucC* and *sdhA*, contain predicted extended sequences of weak CsrA binding affinity in addition to predicted high affinity binding sites (Fig. 7A–C). Overall, the analysis suggests that a model where CsrA does not always immediately recognize the highest affinity binding site(s) within an mRNA, but sometimes recognizes extended sequences of lower affinity that collectively contribute to binding and regulation, may be relevant to a portion of modeled targets beyond the examples of *glgC*, *sdiA*, and *hfq* (Fig. 4). We term this conceptual model as “flexible” CsrA-mRNA binding and propose that mRNAs exhibiting one or more low frequency pockets of binding sites may be regulated in part by such a mechanism.

This interpretation is especially interesting for the *clpB* mRNA, in which only extended sequences of weak CsrA binding affinity are predicted (i.e., no ANGGNs are identified) (Fig. 7D). Perhaps an ability of CsrA to recognize and bind multiple weak sites in the absence of a strong one may contribute to the observed scope and diversity among CsrA targets. These results suggest that predicted pockets of overlapping low affinity binding sites may be useful for identifying non-traditional CsrA-mRNA binding sites within mRNAs of known CsrA regulation that contribute to flexible binding and regulation.

### Analysis of Remaining Modeled mRNA Targets

To elucidate potential molecular diversity within the CsrA targets modeled, we extended our binding site pocket analysis to the 41 mRNAs (out of 107 known to be regulated by CsrA) for which the canonical biophysical model did not correctly predict the mode of regulation. It was expected that predicted binding sites in these targets could still provide insight into CsrA-mRNA interactions because differences between model-predicted and experimental regulation could arise (i) within the translation initiation rate prediction step (as for *flhD*) or (ii) via CsrA regulating translation by destabilizing the transcript^20^, a mechanism of CsrA regulation beyond the scope of the canonical biophysical model. Among their most-likely CsrA-bound conformations, the most notable difference between these 41 mRNAs and the 66 for which the model correctly predicted regulation is the relative depletion of pairs of high affinity ANGGN binding sites in the top conformations of the incorrectly predicted mRNAs. Additionally, 44% (18 of 41) of these mRNAs yield a pocket of 7–10 predicted, non-consensus CsrA binding sites, suggestive of an extended non-traditional binding site that may contribute to flexible binding and regulation (Supplementary Table S3 and Supplementary Fig. S2).

Lastly, we analyzed model results for 129 mRNAs that are suspected to be regulated by CsrA (Supplementary Table S3 and Supplementary Fig. S2). The majority of this pool (78 mRNAs) did not show regulation in our fluorescent translational reporter assays (presumed due to improper expression of GFP) while the others fluoresced, but an impact of CsrA on translation could not be determined (46 mRNAs) or were not tested (5 mRNAs). Analysis of predicted binding site pockets in these 5′ UTRs (Supplementary Table S3) presents excellent starting points for designing mutations in likely binding sites for testing CsrA regulation (and elimination thereof) in alternative assays.

## Discussion

In this work, we developed a biophysical model of canonical CsrA binding and regulation to investigate how the molecular features of a target mRNA can influence its regulation (Figs 1 and 2). We considered a set of 107 mRNAs known to be regulated by CsrA (Tables 1 and S1 and Fig. 3) and 129 mRNAs suspected to interact with CsrA (Supplementary Table S1). After establishing that the model correctly predicted the regulatory modes of 66 of 107 mRNA targets with known CsrA regulation (Table 2), we demonstrated the ability of the model to (i) correctly predict CsrA binding site locations across a range of binding affinities (Figs 4 and 5). Importantly, when examining ensembles of CsrA-mRNA complexes collectively, the model can differentiate between rigidly defined binding pockets with a single high affinity site and loosely defined low affinity binding pockets containing several overlapping sites (Fig. 7 and Supplementary Fig. S2). In Supplementary Table S3, we include all model calculations, including the locations of potential weak, non-consensus sequence CsrA-mRNA interactions across all 236 mRNAs considered in this study.

**Figure 8:**
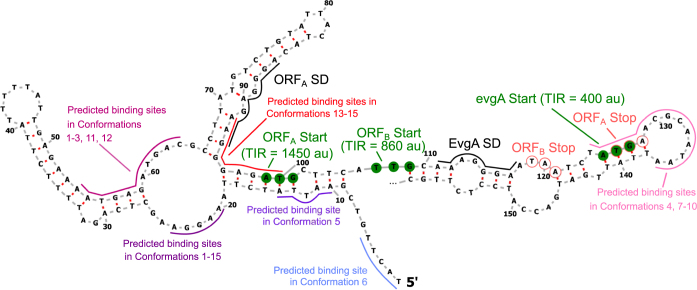
*The evgA* mRNA demonstrates potential co-translational repression mechanism. Two upstream open reading frames are present in the 5′ UTR of *evgA*, both of which are predicted to be translated more quickly than the *evgA* start codon by the RBS calculator (default parameters). The stop codon of ORF_A_ overlaps with the start codon of *evgA*, a signature of co-translation. Notably, CsrA is predicted to bind the Shine-Dalgarno region of ORF_A_, rather than the shine-Dalgarno of *evgA*. While these conformations are predicted to not impact or activate translation, CsrA binding here likely represses *evgA* co-translationally with ORF_A_, a mechanism not incorporated into design of the biophysical model.

For a fraction of the 107 mRNA known to be regulated by CsrA, the model either does not clearly predict a single regulatory outcome (22 mRNAs) or clearly predicts a wrong regulatory outcome (19 mRNAs) across the most-likely fifteen members of their ensemble of CsrA-bound conformations (Table 1). Given that the biophysical model was built from our current understanding of CsrA-mRNA regulatory mechanisms, these CsrA targets offer opportunities to explore unusual or as-of-yet unknown CsrA regulatory mechanisms. Specifically, we were inspired by a recent study of the CsrA-*iraD* mRNA interaction^45^ to look for mRNAs that could be repressed indirectly via a translational coupling mechanism. Here, CsrA indirectly represses *iraD* translation by binding and repressing translation of the *idlP* open reading frame, which is located in the same operon and within the 5′ UTR of *iraD* (as defined by *iraD’s* annotated promoters). The canonical biophysical model does predict a CsrA binding site in the Shine-Dalgarno region of *idlP* (which also overlaps an *in vitro* CsrA footprint^45^), but it does not predict repression because translational coupling and thus potential co-regulation between coding sequences is not currently accounted for (Supplementary Table S3). The *evgA* mRNA, a well-characterized CsrA target not captured by the model, presents a similar case of potentially complex CsrA interaction and regulation that falls outside the canonical model. Employing the RBS Calculator indicates that the translation rates of two upstream start codons are substantial compared to the translation rate of the annotated *evgA* start codon (Fig. 8). Furthermore, the first upstream start codon reveals an open reading frame (ORF_A_ in Fig. 8) that contains a stop codon overlapping with the annotated *evgA* start codon (5′-ATGA-3′). The biophysical model identifies a predicted binding site within the SD of ORF_A_, but predicts this conformation to inconsistently impact *evgA* translation. Importantly, features exhibited by the *evgA* mRNA are commonly associated with the ribosome re-initiation mode of translational coupling^49^, a mechanistic feature not currently accounted for in the biophysical model. Biophysical models of translational coupling have previously been developed^49^, and could be leveraged in the future to further extend and improve the model predictions here. We anticipate that further investigation of all 41 mRNAs whose regulation is not well-predicted by the canonical biophysical model will uncover similar insights into poorly understood CsrA-mRNA regulatory mechanisms.

More broadly, our results suggest that CsrA participates in more flexible binding interactions than previously discussed in the literature. The presence of many low affinity CsrA binding sites nearby a single high affinity site suggests that CsrA may be initially recruited to mRNAs at one of the low affinity sites, followed by sliding into a high affinity site, which often appear within Shine-Dalgarno sequences with a clear regulatory outcome. Analogously, transcription factors have been proposed to hop or slide across low-specificity DNA sites to accelerate their search for high affinity sites^50,51^. Similarly, RNA-binding proteins like CsrA may utilize similar mechanisms of facilitated diffusion to accelerate their search for consensus, high affinity sites, which become all the more important as mRNAs are more well-mixed inside the cytoplasm, compared to genomic DNA.

This perspective adds an interesting biophysical possibility for mRNA in which CsrA-bound conformations in the ensemble lead to different types of regulation, where multiple modes of binding (using different binding site combinations) could lead to opposing regulatory outcomes. In this sense, it is possible that the specific CsrA-mRNA binding modes that take place *in vivo* are biased by additional factors, such as other regulators, small molecules, or environmental stresses, shifting the preference of one regulatory outcome or the other. For instance, CsrA-bound conformations 1–5 and 7–10 identified in the *rseA* mRNA are predicted to repress translation, while CsrA-bound conformations 6, 11–13, and 15 are predicted to activate translation (Fig. 7A). Although the conformations that lead to activation represent an energetic minority (21%) considering only CsrA-mRNA interactions, it is possible that the binding of other RNA-binding proteins or small molecules may increase the chance that CsrA visits these conformations, activating translation of *rseA*. Dual regulatory modes may also exist for the mRNAs *iscR*, *nnr*, and *dkgA* (Supplementary Fig. S2). It is therefore conceivable that translation of such mRNAs could be controlled by several factors hierarchically; in the absence of the external factor, CsrA may repress the mRNA’s translation, but in its presence, the mRNA’s translation may be activated.

Future developments in understanding CsrA binding across its broad target pool will continue to provide other valuable insights about CsrA regulation. One important factor to consider about the model described in this work is that it was derived from thermodynamic first principles and parameterization using only canonical CsrA binding sites^17,18,52^. The fact that the model has accuracy with only a minimal amount of input suggests that similar models of regulators could be generated and analyzed in a comparable manner. We envision that the model principles described here may be valuable for other systems of protein-based mRNA translation control, such as the trp RNA-binding attenuation protein (TRAP) of *B*. *subtilis*^53,54^. More broadly, the concept of thermodynamic models elucidating affinity-based target hierarchies is readily extendable to other types of regulators, including those involved in transcriptional control where target hierarchies have already been experimentally implicated^55–57^. Overall, we anticipate that this work will provide a generalizable strategy as a starting point to model post-transcriptional regulation in a variety of contexts.

## Materials and Methods

### Development of Model

#### Identifying Potential CsrA Binding Sites

Given an arbitrary mRNA sequence, the model first calculates CsrA’s binding free energy to all possible binding sites. To do this, we assign a thermodynamic binding free energy to each nucleotide within a 5-nucleotide binding site, determined by converting the EMSA-determined binding affinities for mutated CsrA binding sites into free energy changes^18^. The resulting position-specific mono-nucleotide free energy model (FEM) is:

$$Nucleotide\,Position\,\begin{array}{c}{N}_{1}\\ {N}_{2}\\ {N}_{3}\\ {N}_{4}\\ {N}_{5}\end{array}\,[\begin{array}{c}\begin{array}{cccc}{\boldsymbol{A}}\,({\rm{\Delta }}{G}_{i}\,in\,RT) & {\boldsymbol{C}}\,({\rm{\Delta }}{G}_{i}\,in\,RT) & {\boldsymbol{G}}\,({\rm{\Delta }}{G}_{i}\,in\,RT) & {\boldsymbol{U}}\,({\rm{\Delta }}{G}_{i}\,in\,RT)\\ -\,2.63 & 0 & 0 & 0\\ -\,2.20 & 0 & 0 & 0\\ 0 & 0 & -\,3.14 & 0\\ 0 & 0 & -\,3.14 & 0\\ -\,1.65 & 0 & 0 & 0\end{array}\end{array}].$$

All energy units are RT, which is the gas constant multiplied by system temperature. The binding free energy of CsrA to a 5-nucleotide site is then calculated by summing the contributions:

2 $${{\rm{\Delta }}{\rm{G}}}_{Site}=\sum _{i={N}_{1}}^{{N}_{5}}{{\rm{\Delta }}{\rm{G}}}_{i}$$

Position score matrices have been used previously for the identification of CsrA targets^23,43,58,59^. Here, we quantify changes in binding occupancy in terms of free energies to develop a more comprehensive multi-term free energy model that incorporates CsrA binding, inter-site cooperativity, mRNA structural changes, and regulation of translation rate.

#### Calculating Cooperative Binding Effects

The model next quantifies the cooperative effects of a CsrA homodimer binding to two sites in an mRNA. Previous studies have indicated that the dimeric CsrA protein will bind to two adjacent sites in an mRNA, separated by 10 to 46 nucleotides^17,19^, with the potential for cooperative binding^19–22^. We quantify the energy of the cooperative effect of a CsrA homodimer binding two adjacent sites, denoted by ΔG_cooperativity_, in terms of the inter-site distance *d*, according to the following formulas:

3 $${\rm{For}}\,10\le {\rm{d}}\le 55,\,{{\rm{\Delta }}{\rm{G}}}_{{\rm{cooperativity}}}=0.001{({\rm{d}}-5)}^{2}+0.05({\rm{d}}-5)-5.0\,{\rm{RT}}\,{\rm{units}}$$

4 $${\rm{For}}\,{\rm{d}} < 10,\,{\rm{Steric}}\,{\rm{penalty}}\,{\rm{of}}\,{{\rm{\Delta }}{\rm{G}}}_{{\rm{cooperativity}}}=20\,{\rm{RT}}\,{\rm{units}}$$

Here, if the inter-site distance is too small, then two adjacent sites will sterically clash, resulting in a large, repulsive free energy contribution. If the inter-site distance is 10 nucleotides, the cooperative interaction is strongest (ΔG_cooperativity_ = −4.725 RT). The strength of the cooperative interaction is then proposed to decrease as the inter-site distance increases, reaching zero at 55 nucleotides. These formula fit previous observations of cooperative CsrA binding across relatively long inter-site distances^17^.

#### Calculating CsrA-induced mRNA Structural Changes

When CsrA binds to an mRNA, it has the potential to induce changes in the mRNA’s structure. When these changes take place nearby the start codon of a protein coding sequence, it can alter the ribosome’s ability to bind the mRNA and initiate translation, changing the mRNA’s translation rate. To determine where and when this takes place, we determine the free energy needed to remodel the mRNA’s structure by calculating the difference in mRNA folding free energy with and without accessible CsrA binding sites (ΔG_§_ versus ΔG_unbound mRNA_). We perform these calculations for each unique pair of the identified CsrA binding sites. For a pair of sites, we first calculate the most stable mRNA structure in the absence of CsrA, using ViennaRNA’s dynamic programming algorithm^48^ and the Turner 1999 RNA free energy parameters^60^. The default temperature of 37 °C is used. Dangling nucleotide energies are not used. We then repeat the same calculation with the addition of a structural constraint that prevents the two 5-nucleotide CsrA binding sites from participating in any mRNA structure. The CsrA binding sites in the resulting mRNA structure must be single-stranded, but they can be located anywhere within the mRNA, including within a hairpin loop. We then take the difference between these two folding free energies to determine the amount of energy needed to induce this structural change, which must be positive:

5 $${{\rm{\Delta }}{\rm{\Delta }}{\rm{G}}}_{{\rm{unfold}}}={{\rm{\Delta }}{\rm{G}}}_{\S }-{{\rm{\Delta }}{\rm{G}}}_{{\rm{unbound}}{\rm{mRNA}}}$$

#### An Overall Free Energy Model for CsrA-mRNA Interactions

Given an arbitrary mRNA sequence, we enumerate all possible CsrA binding sites, evaluate each free energy term, and sum them together to calculate the total free energy change when a CsrA homodimer binds the mRNA at two sites, according to the following formula:

$${{\rm{\Delta }}{\rm{G}}}_{{\rm{mRNA}}:{\rm{CsrA}}}={{\rm{\Delta }}{\rm{G}}}_{{\rm{site}}1}+{{\rm{\Delta }}{\rm{G}}}_{{\rm{site}}2}+{{\rm{\Delta }}{\rm{G}}}_{{\rm{cooperativity}}}+{{\rm{\Delta }}{\rm{\Delta }}{\rm{G}}}_{{\rm{unfold}}}$$

where ΔG_site1_ is the binding free energy of a CsrA to an upstream site (negative), ΔG_site2_ is the binding free energy of a CsrA to a downstream site (negative), ΔG_cooperativity_ is the cooperative free energy whenever CsrA is bound to two sites at most 55 nucleotides apart (positive or negative), and ΔΔG_unfold_ is the free energy needed to unfold one or both CsrA binding sites (positive). Together, the total binding free energy (ΔG_mRNA:CsrA_) for a particular pair of sites may be positive or negative. For the mRNAs in this study, each has between 1800 to 14,000 possible pairs of CsrA binding sites, comprising an ensemble of possible CsrA-bound mRNA states with varying binding free energies. Formally, the probability of CsrA binding to one of these CsrA binding site conformations can be expressed in terms in a Boltzmann distribution:

6 $$p(\alpha )=\frac{{e}^{-\beta {{\rm{\Delta }}{\rm{G}}}_{{\rm{mRNA}}:{\rm{CsrA}}\_{\rm{\alpha }}}}}{\sum _{i}^{M}{e}^{-\beta {{\rm{\Delta }}{\rm{G}}}_{{\rm{mRNA}}:{\rm{CsrA}}\_{\rm{i}}}}},$$

where α is a particular CsrA-bound mRNA conformation and ΔG_CsrA:mRNA,α_ is the total free energy change in that conformation. *M* is the total number of possible CsrA-bound mRNA conformations for a particular mRNA sequence.

#### Predicting CsrA-induced Translation Regulation

When CsrA binds an mRNA and induces structural changes, the mRNA’s translation initiation rate can increase *or* decrease, depending on where the CsrA binding site is located and the structures that form after CsrA is bound. To predict these effects, we calculate the ribosome’s binding free energy to mRNAs that are either unbound or bound twice by a CsrA protein, employing a previously developed free energy model, called the RBS Calculator^34,35^. For a free mRNA without CsrA bound, the RBS Calculator’s free energy model has the following five terms:

7 $${{\rm{\Delta }}G}_{{\rm{t}}{\rm{o}}{\rm{t}}{\rm{a}}{\rm{l}},{\rm{f}}{\rm{r}}{\rm{e}}{\rm{e}}}=({{\rm{\Delta }}G}_{{\rm{m}}{\rm{R}}{\rm{N}}{\rm{A}}:{\rm{r}}{\rm{R}}{\rm{N}}{\rm{A}}}+{{\rm{\Delta }}G}_{{\rm{s}}{\rm{t}}{\rm{a}}{\rm{r}}{\rm{t}}}+{{\rm{\Delta }}G}_{{\rm{s}}{\rm{p}}{\rm{a}}{\rm{c}}{\rm{i}}{\rm{n}}{\rm{g}}}+{{\rm{\Delta }}G}_{{\rm{s}}{\rm{t}}{\rm{a}}{\rm{n}}{\rm{d}}{\rm{b}}{\rm{y}}})-{{\rm{\Delta }}G}_{{\rm{u}}{\rm{n}}{\rm{b}}{\rm{o}}{\rm{u}}{\rm{n}}{\rm{d}}{\rm{m}}{\rm{R}}{\rm{N}}{\rm{A}}}$$

where the first four terms quantify the free energy of the ribosome-mRNA complex in its final state, and the last term quantifies the folding free energy of the mRNA by itself in the initial state. The ΔG_total,free_ calculation is performed by identifying the most stable initial and final states with their lowest respective free energies, and then computing their difference in free energy. The mRNA’s translation initiation rate *r* is then determined according to:

8 $${r}_{free}\propto \exp (\,-\,\beta {{\rm{\Delta }}{\rm{G}}}_{{\rm{total}},{\rm{free}}})$$

where *β* is an empirically measured Boltzmann constant for *E*. *coli* (*β* = 0.45 mol/kcal)^37,61^.

When a CsrA protein binds to an mRNA, the ΔG_mRNA:rRNA_ and ΔG_standby_ terms will change whenever there is a CsrA-induced mRNA structural change that overlaps with an upstream standby site or the ribosome’s footprint, which includes the Shine-Dalgarno sequence, the start codon, and the first 13 nucleotides of the protein coding sequence^62^. For any change in mRNA structure, we recalculate these free energy model terms to determine the change in the ribosome’s binding free energy, ΔG_total,CsrA_, and the corresponding change in the mRNA’s translation rate, r_CsrA_, using the free energy model:

9 $${{\rm{\Delta }}{\rm{G}}}_{{\rm{total}},{\rm{CsrA}}}=({{\rm{\Delta }}{\rm{G}}}_{{\rm{mRNA}}:{\rm{CsrA}}:{\rm{rRNA}}}+{{\rm{\Delta }}{\rm{G}}}_{{\rm{start}}}+{{\rm{\Delta }}{\rm{G}}}_{{\rm{spacing}}}+{{\rm{\Delta }}{\rm{G}}}_{{\rm{standby}}:{\rm{CsrA}}})-{{\rm{\Delta }}{\rm{G}}}_{{\rm{mRNA}}:{\rm{CsrA}}}$$

Calculating the free energy terms ΔG_mRNA:CsrA:rRNA_ and ΔG_standby:CsrA_ follows the same procedure as for a free mRNA, except with two structural constraints: first, the mRNA region overlapping with the ribosomal footprint may neither have a bound CsrA nor a folded inhibitory mRNA structure, and second, a CsrA-bound site outside the ribosomal footprint must be single-stranded. In this way, if CsrA is predicted to bind to a site overlapping the ribosomal footprint, the energy required to remove bound CsrA is included in ΔG_mRNA:CsrA:rRNA_. Calculating ΔG_mRNA:CsrA_ is as described in the previous section. Importantly, an mRNA will often contain thousands of potential pairs of sites where CsrA can bind; each CsrA-bound mRNA conformation can have different ΔG_mRNA:CsrA_ free energies. In practice, we consider the most probable 15 double-site CsrA-bound mRNA conformations, i.e., those with the most negative ΔG_CsrA:mRNA_ free energies, and apply the RBS Calculator’s free energy model to calculate their respective translation initiation rates. For each of these *i*^th^ conformations, we calculate their translation initiation rates using:

10 $${r}_{CsrA,i}\propto \exp (\,-\,\beta {{\rm{\Delta }}{\rm{G}}}_{{\rm{total}},{\rm{CsrA}},{\rm{i}}})$$

Finally, for each *i*^th^ CsrA-bound mRNA conformation, we determine whether the mRNA’s translation rate was activated or repressed by calculating the ratio:

11 $${R}_{i}={r}_{{\rm{free}}}/{r}_{CsrA,i}$$

#### Metric for evaluating predictions of CsrA targets

We label each CsrA-bound mRNA conformation’s translation rate as “repressed” if *R*_*i*_ > 1.2, as “activated” if *R*_*i*_ < 0.8, and as “not impacted” otherwise. We then determine the most likely mode of translation regulation by comparing the 15 conformations’ calculated ΔG_CsrA:mRNA_ free energies and ensuring that the most frequent states, energetically weighted, all had the same regulatory label. If predictions yielded CsrA-bound conformations with different regulatory labels with approximately equal frequency, then the mRNA was labeled as having a “heterogeneous” mode of translation regulation. Specifically, we summed the probabilities (*p*(*α*), defined above) of all of the repressed, activated or not impacted CsrA-bound conformations of an mRNA; the most likely mode of regulation was taken to represent the ensemble only if it was at least 1.8-fold more likely than the other two possible types of regulation (threshold determined from inspection of classical CsrA target model results). This qualitative analysis is valid across a wide range of CsrA concentrations inside the cell. Using an ensemble-based collective regulation prediction allows us to account more thoroughly for mRNAs with multiple pairs of equally likely CsrA binding sites. However, a higher-energy conformation within an mRNA’s most likely 15 CsrA-bound conformations may only be occupied in *in vivo* under conditions of excess CsrA and after the more likely bind site pairs are bound. By weighting regulation predictions with Boltzmann distribution-derived probabilities we aim to account for this discrepancy while still incorporating the diversity of the binding sites predicted by the model within a given mRNA.

### Fluorescent Translational Reporter Assay

Fluorescent translational reporter assays were performed as described in the Supplementary Methods.

### Titration of CsrA targets

To test whether ΔG_CsrA:mRNA_ was indicative of the thermodynamics of CsrA binding to transcripts *in vivo*, we constructed variants of pHL 600 that produce a range of CsrA expression (Supplementary Tables S5 and S6). Specifically, the RBS calculator^35^ was used to design RBSs with an anticipated 50-fold range of translation rates, including variants that were predicted to yield CsrA expression with putative levels of translation, as calculated by the RBS Calculator, of 2000, 1000, 800, 750, 350, and 200 AU (original plasmid, used in the fluorescent translational reporter assay, was 9000 AU). In order to estimate the level of CsrA produced from each plasmid in the uninduced condition, i.e. from promoter “leakiness”, the putative translation level of the RBS was multiplied by 0.000645, a value of promoter expression in the undinduced condition determined in a prior work^63^. Additionally, the putative CsrA translation levels are expected to have error between 50% and 200%^37^.

We then paired this set of seven RBS-CsrA variants with three 5′ UTRs-GFP reporters (*glgC*, *aidB*, and *maeB*), each with 5′ UTRs predicted to (1) be repressed by CsrA, (2) have a range of ΔG_CsrA:mRNA_ values (*glgC*, −24.77 RT units; *aidB*, −19.53 RT units; and *maeB*, −23.06 RT units), and (3) contain one identified binding site of the most likely CsrA-bound conformation in the Shine-Dalgarno region. These 5′ UTRs were cloned into pHL 1756 plasmids previously (Supplementary Table S5). Eight mutations to the *glgC* 5′ UTR-GFP reporter plasmid were also constructed via site directed mutagenesis (Quickchange II protocol for Agilent Technologies) to vary the middle three nucleotides of the footprinted non-Shine Dalgarno CsrA binding site (wild type sequence: ACGGA) (Supplementary Tables S5 and S6). In this way, the mutated sequences created a range of ΔG_Site_ values. Pairing a single 5′ UTR-GFP reporter with the set of RBS-CsrA variants at a time, we took fluorescence measurements in biological triplicates as described in the fluorescent translational reporter assay section above. However, analysis of titration experiment data differs; in these tests, rather than comparing corresponding uninduced and induced conditions, a repression ratio due to CsrA was calculated for each RBS in each condition, with respect to the maximum average mean fluorescence of all of the samples:

12 $$Repression\,Ratio=\frac{Maximum\,of\,all\,average\,mean\,sample\,fluorescences}{Average\,mean\,sample\,fluorescence}.$$

For example, in the case of *glgC*, the maximum of all average mean sample fluorescences corresponded to the average mean fluorescence of the weakest RBS*-*CsrA variant (200x) in the uninduced condition. In this way, 14 repression ratios, each corresponding to a different level of CsrA expression (uninduced 200x, induced 200x, uninduced 350x, induced 350x, etc.), are determined with the minimum repression ratio always equal to 1. To compare results of the titrated 5′ UTR-GFP reporters, the repression ratios measured for each were scaled from 0 to 1, where 0 represents the minimum repression ratio and 1 the maximum repression ratio:

13 $$Normalized\,Repression\,Ratio=\frac{(Repression\,Ratio-minimum\,Repression\,Ratio)}{(maximum\,Repression\,Ratio-minimum\,Repression\,Ratio)}.$$

Lastly, dissociation constants (K_d_) for the *glgC*, *maeB*, and *aidB* titration datasets were approximated as a means to generate fits for their titration curves^64^. Assuming a near constant pool of each 5′ UTR-GFP reporter transcript, K_d_ can be approximated with,

14 $$\theta =\frac{[CsrA]}{{K}_{d}+[CsrA]\,},$$

where θ indicates the fractional extent of CsrA-5′ UTR binding (i.e. normalized repression ratio) and [CsrA] indicates CsrA expression levels (i.e. putative RBS translation levels). The R Package for Statistical Computing non-linear least squares equation fitting function, nls, was employed to fit each titration dataset with the above equation and approximate a K_d_ for the CsrA-5′ UTR interaction.

### Data availability

All data generated or analyzed during the current study are included in this published article (and its Supplementary Information files). Fluorescent translational reporter assay results analyzed in this work but published previously are available as Supplementary Information files at 10.1093/nar/gkx048. Original scripts are available from the corresponding author upon request.

## Electronic supplementary material

Supplementary Information

Supplementary Tables S1-6

Supplementary Dataset 1
