# Supplementary material for: A Canonical Biophysical Model of the CsrA Global Regulator Suggests Flexible Regulator-Target Interactions
Source: Sci Rep. 2018 Jul 2;8:9892. doi: 10.1038/s41598-018-27474-2 (PMC6028588; doi:10.1038/s41598-018-27474-2)
Supplement: Supplementary file 1 — Supplementary Information [file 41598_2018_27474_MOESM1_ESM.pdf]

Supplementary Information for

A Canonical Biophysical Model of the CsrA Global Regulator Suggests Flexible Regulator-Target Interactions

Leistra, A. N.,[1] Gelderman, G.,[1] Sowa, S. W.,[2] Moon-Walker, A.,[3] Salis, H. M.,[4] Contreras, L. M.[1*]

1 McKetta Department of Chemical Engineering, University of Texas at Austin, 200 E. Dean Keeton St. Stop C0400, Austin, TX 78712, USA

2 Microbiology Graduate Program, University of Texas at Austin, 100 E. 24th St. Stop A6500, Austin, TX 78712, USA

3 Biological Sciences Program College of Natural Sciences, University of Texas at Austin, 120 Inner Campus Drive Stop G2500, Austin, TX 78712 USA

4 Department of Chemical Engineering, Pennsylvania State University, University Park, PA 16802, USA

* Corresponding Author: lcontrer@che.utexas.edu

Table of Contents

Supplementary Methods 3
Fluorescent Translational Reporter Assay 3
Supplementary Figures 5
Supplementary Tables 7
Supplementary Data 8

Supplementary Methods

Fluorescent Translational Reporter Assay

Fluorescent translational reporter assays were performed to develop a set of CsrA targets of known regulation. To designate the length of the 5' untranslated region (UTR) of an mRNA to be tested, we used RegulonDB[1] to identify the shortest annotated 5' UTR associated with each gene of interest which minimizes opportunities to include unanticipated regulatory elements. For genes inside an operon, we chose a 100-nucleotide sequence preceding the translation initiation site based on evidence that CsrA acts specifically on some operons internally[2]. We also included the first 100 nucleotides of each gene's coding sequence with the defined 5' UTR for testing and modeling; here we refer to this composite sequence as the "5' UTR."

Once designed, the 5' UTR constructs were cloned into pHL 1756 between the SalI and SphI restriction sites to generate an in-frame fusion of the 5' UTR plus coding sequence of the gene to GFP (Supplementary Tables S5 and S6). Experimental conditions for the fluorescent translational reporter assay have been described previously[3]. Briefly, once designed and constructed, these 5' UTR-GFP plasmids were transformed into a strain of modified *E. coli* K-12 MG1655 containing *ΔcsrABCDΔglgCAPΔpgaABCD* deletions, a chromosomal copy of LacI and a previously transformed plasmid, pHL 600, expressing CsrA upon induction with IPTG (Supplementary Table S5)[4]. Biological duplicates of each 5' UTR-GFP reporter were split in half, grown to exponential phase (OD 0.3), and two of four samples induced with IPTG (100 µM in final culture) to stimulate CsrA expression. After induction, samples were grown for three hours and green fluorescence measured with a BD FACSCalibur flow cytometer. The two uninduced samples were used as controls to account for background fluorescence levels.

After collecting the data, means of the fluorescence peaks for the CsrA-induced and CsrA-uninduced samples of a given 5' UTR-GFP reporter were identified; the fold change from uninduced to induced ("fold repression") was determined by dividing the average mean uninduced fluorescence by the average mean induced fluorescence. Two initial quality checks were applied. If the induced and uninduced populations both yielded an average mean fluorescence less than 10 A.U., the 5' UTR-GFP reporter was determined to be "non-fluorescent" (e.g. unable to appropriately capture CsrA regulation). Also, if the differences between the induced and uninduced samples were inconsistent across biological duplicates regulation of the 5' UTR-GFP reporter was termed "not determined." Next, 5' UTR-GFP reporters with fold repression values > 1.2 and P-values < 0.1 (heteroscedastic one-tailed T-test) were considered to be "repressed" by CsrA. Likewise, 5' UTR-GFP reporters with fold repression values < 0.8 and P-values < 0.1 (heteroscedastic one-tailed T-test) were considered to be "activated" by CsrA. 5' UTR-GFP reporters with fold repression > 1.2 or less than < 0.8 with P-values ≥ 0.1 were considered as having "not determined" CsrA regulation and were excluded from comparison to model predictions. Additionally, 5' UTR-GFP reporters with fold repression within the 0.8-1.2 range (inclusive and regardless of P-value) were considered as having "not determined" CsrA regulation and were excluded from comparison to model predictions. In most cases, 5' UTR-GFP constructs with fold repression values in the 0.8-1.2 range presented low absolute fluorescence values (10-30 A.U.), offering limited sensitivity to any possible CsrA regulatory impact. Moreover, given that this assay tests for CsrA regulation in a very specific context, the lack of measured activation or repression does not necessarily mean CsrA does not regulate the target natively. As such, no 5' UTR-GFP reporters were considered to be "not

regulated” by CsrA, only activated, repressed, not determined, or non-fluorescent; only activated or repressed 5’ UTRs were included in comparison to model predictions.

Supplementary Figures

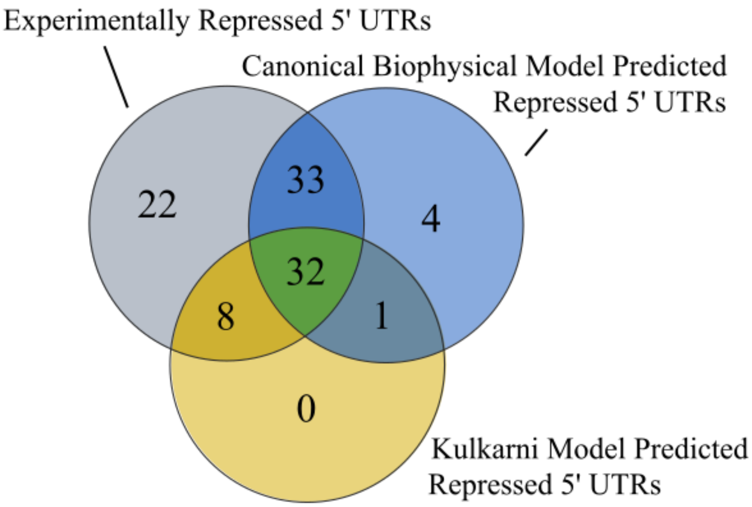

Fig. S1. Venn diagram comparing experimentally-repressed and model-predicted repressed 5’ UTRs. 5’ UTRs repressed in the fluorescent translational reporter assay (or in a prior study) are shown in the large gray circle (upper left). 5’ UTRs predicted to be repressed with the Canonical Biophysical and Kulkarni et. al. models are shown in the large blue (upper right) and large yellow (lower) circles, respectively. Overlaps indicate predicted or observed repressed 5’ UTRs shared between the categories. Model-predicted repressed mRNAs for which *in vivo* CsrA-mRNA regulation are unknown were excluded from this comparison. The Venn diagram compares experimental and model results on repressed targets only as the Kulkarni model exclusively predicted mRNA targets expected to be repressed.

(Multi-page figure starts on page 10)

Fig. S2. Predicted free energy terms and binding site positions for 236 modeled mRNA. (Upper panel) Free energy terms calculated for the most-likely fifteen members of the ensemble of CsrA-bound conformations of an mRNA. Data labels mark the Boltzmann probability of each conformation, scaled such that the total probability of the fifteen most-likely conformations is one hundred percent. Total scaled Boltzmann probabilities of the repressed, not impacted, or activated conformations are noted. Regulation observed in fluorescent translational reporter assay is noted in the title as “mRNA name repressed/activated/not_determined/non_fluorescent /not_tested in expt.” (Lower panel) Distribution of binding sites (by location) predicted in an mRNA.

Supplementary Tables

Table S1. CsrA targets modeled in this study. Table lists classical, well-characterized, and functional mRNA targets of CsrA, their regulation, and references establishing that regulation. Potential target mRNAs are listed as “other modeled,” non-fluorescent or not determined result of fluorescent translational reporter assay is listed, and evidence supporting potential regulation is cited.

Table S2. Canonical biophysical model predictions. Predicted binding sites, pockets of binding sites, ΔG values, translation initiation rates, and collective ensemble regulation of mRNA from the canonical biophysical model.

Table S3. Results of fluorescent translational reporter assay (flow cytometry data).

Table S4. Comparison of experimental regulation with the Canonical Biophysical model, Kulkarni et. al. model, and McKee et. al. model predictions.

Table S5. Plasmids used in this study.

Table S6. Primers used in this study.

Supplementary Data

Supplementary Data 1. Raw model results for 236 mRNA. The full ensemble of identified binding sites and predicted free energies, in rank order, is included for each mRNA on a labeled tab.

References

1 Salgado, H. *et al.* RegulonDB v8.0: omics data sets, evolutionary conservation, regulatory phrases, cross-validated gold standards and more. *Nucleic Acids Res* **41**, D203-213, doi:10.1093/nar/gks1201 (2013).

2 Pannuri, A. *et al.* Translational repression of NhaR, a novel pathway for multi-tier regulation of biofilm circuitry by CsrA. *J. Bacteriol.* **194**, 79-89, doi:10.1128/jb.06209-11 (2012).

3 Sowa, S. W. *et al.* Integrative FourD omics approach profiles the target network of the carbon storage regulatory system. *Nucleic Acids Res.* **45**, 1673-1686, doi:10.1093/nar/gkx048 (2017).

4 Adamson, D. N. & Lim, H. N. Rapid and robust signaling in the CsrA cascade via RNA–protein interactions and feedback regulation. *Proc. Natl. Acad. Sci. U. S. A.* **110**, 13120-13125, doi:10.1073/pnas.1308476110 (2013).

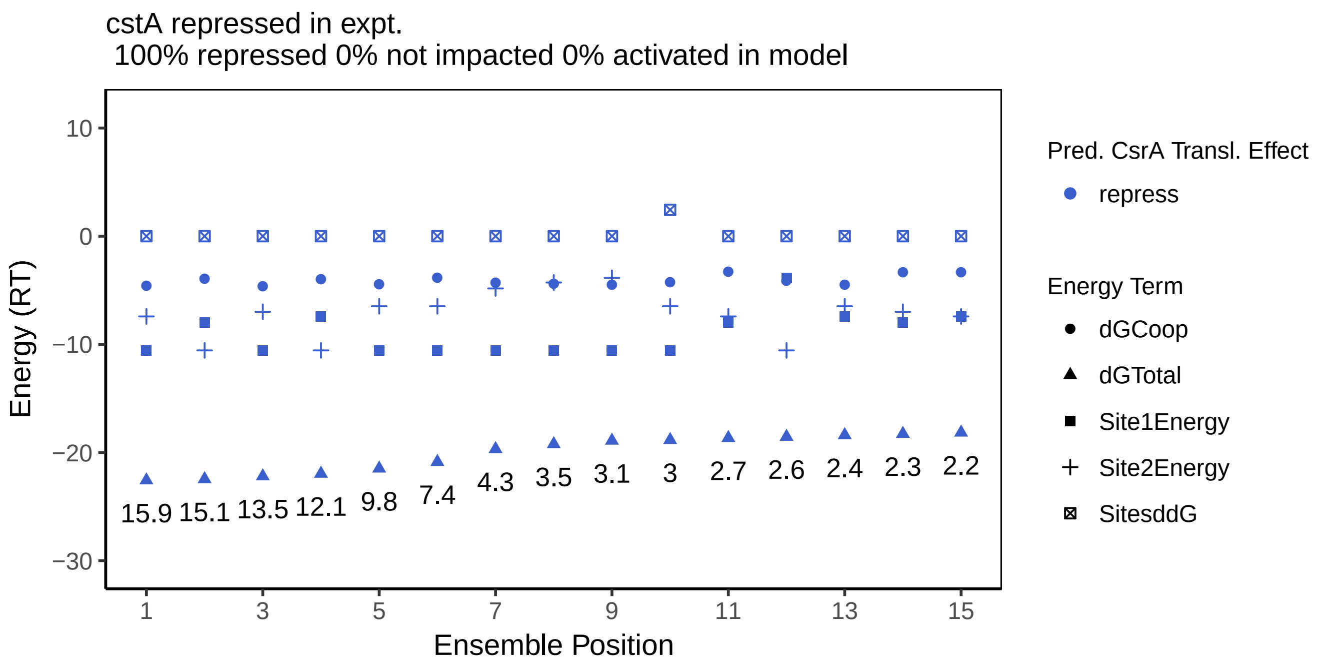

cstA repressed in expt.
100% repressed 0% not impacted 0% activated in model
Energy (RT)
10
0
−10
−20
−30
15.9 15.1 13.5 12.1 9.8 7.4 4.3 3.5 3.1 3 2.7 2.6 2.4 2.3 2.2
1 3 5 7 9 11 13 15
Ensemble Position
Pred. CsrA Transl. Effect
repress
Energy Term
dGCoop
dGTotal
Site1Energy
Site2Energy
SitesddG

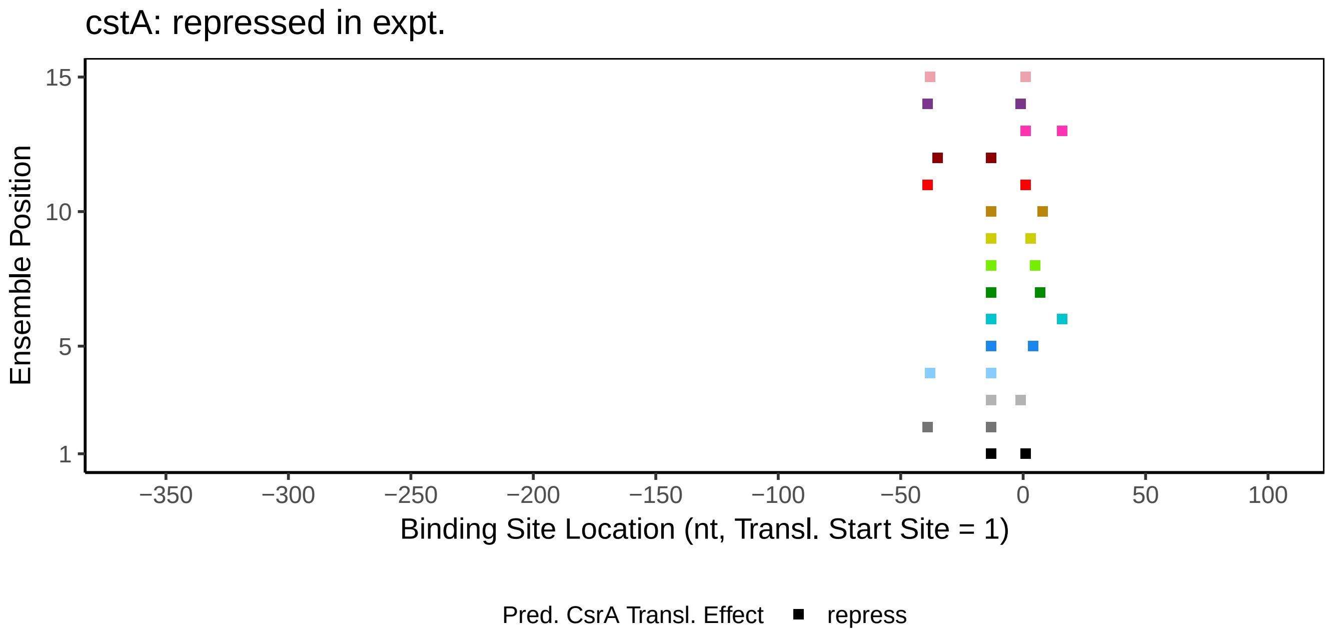

cstA: repressed in expt.
Ensemble Position
15
10
5
1
−350 −300 −250 −200 −150 −100 −50 0 50 100
Binding Site Location (nt, Transl. Start Site = 1)
Pred. CsrA Transl. Effect
repress

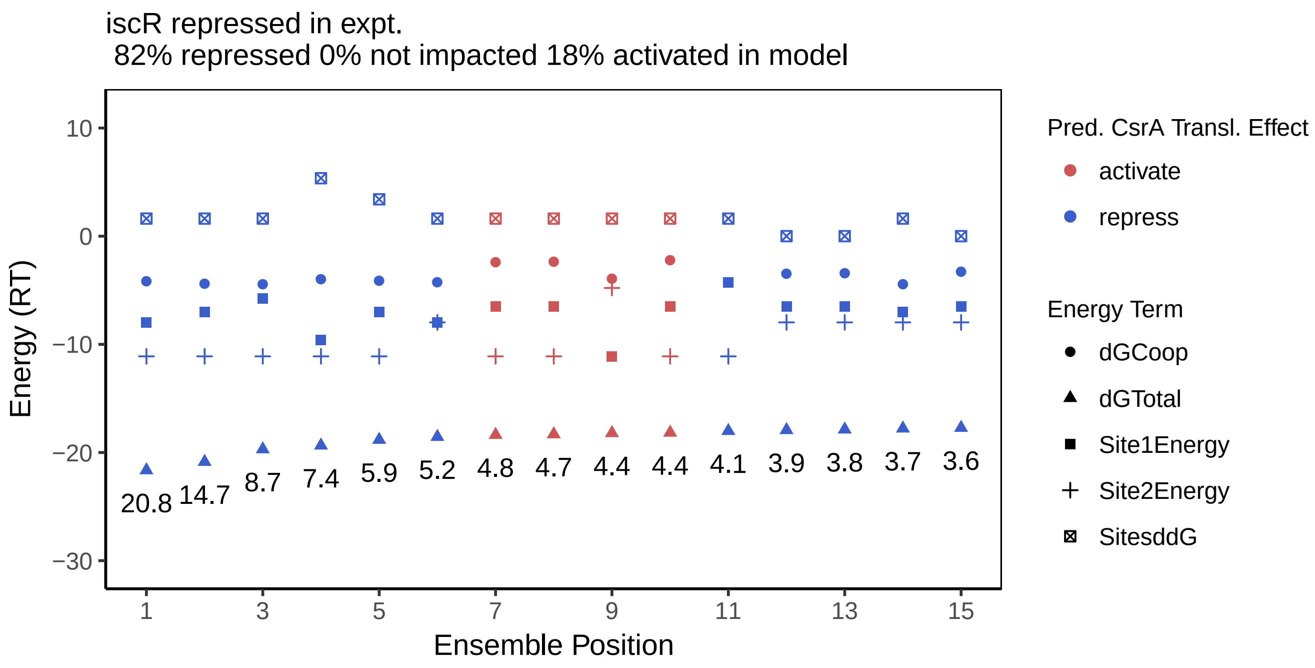

iscR repressed in expt.
82% repressed 0% not impacted 18% activated in model
Energy (RT)
Ensemble Position
20.8 14.7 8.7 7.4 5.9 5.2 4.8 4.7 4.4 4.4 4.1 3.9 3.8 3.7 3.6
Pred. CsrA Transl. Effect
activate
repress
Energy Term
dGCoop
dGTotal
Site1Energy
Site2Energy
SitesddG

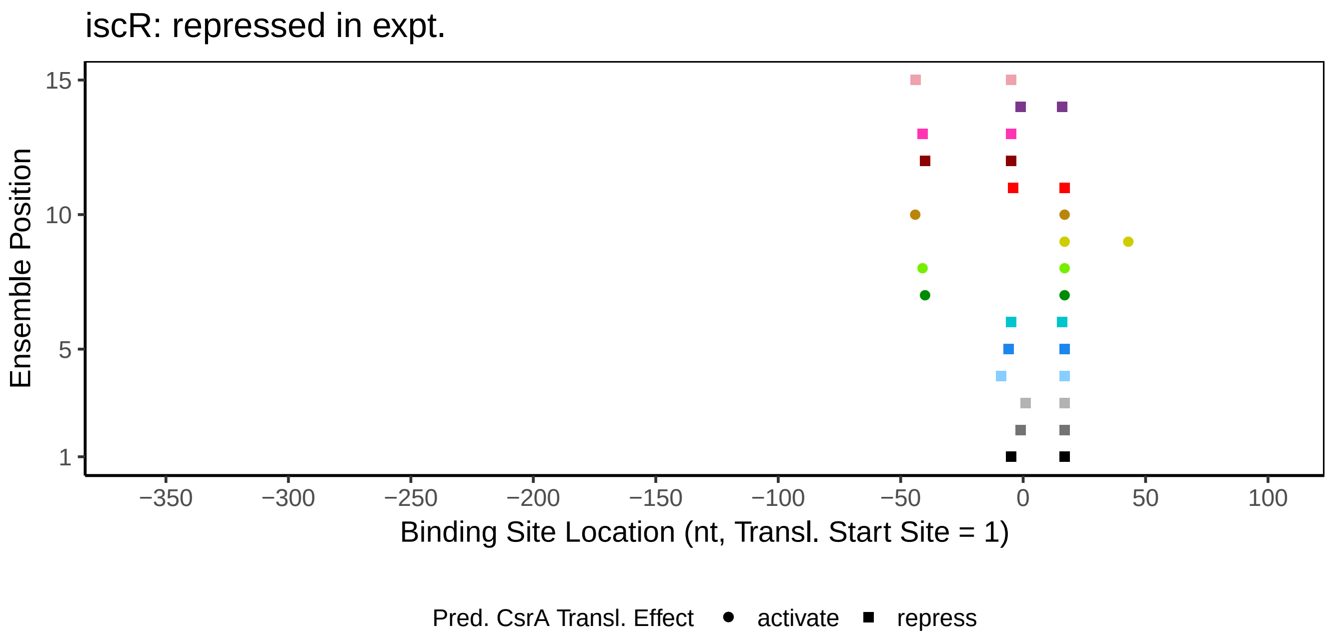

iscR: repressed in expt.
Ensemble Position
Binding Site Location (nt, Transl. Start Site = 1)
Pred. CsrA Transl. Effect
activate
repress

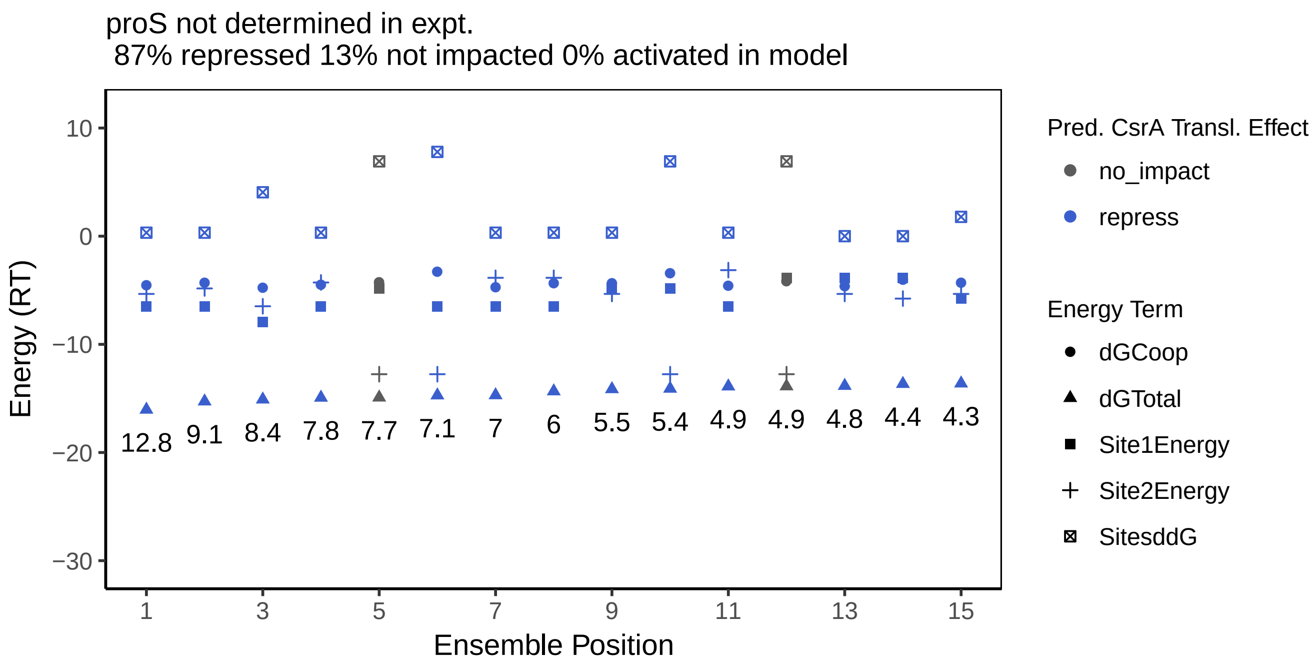

proS not determined in expt.
87% repressed 13% not impacted 0% activated in model
Energy (RT)
Ensemble Position
12.8
9.1
8.4
7.8
7.7
7.1
7
6
5.5
5.4
4.9
4.9
4.8
4.4
4.3
Pred. CsrA Transl. Effect
no_impact
repress
Energy Term
dGCoop
dGTotal
Site1Energy
Site2Energy
SitesddG

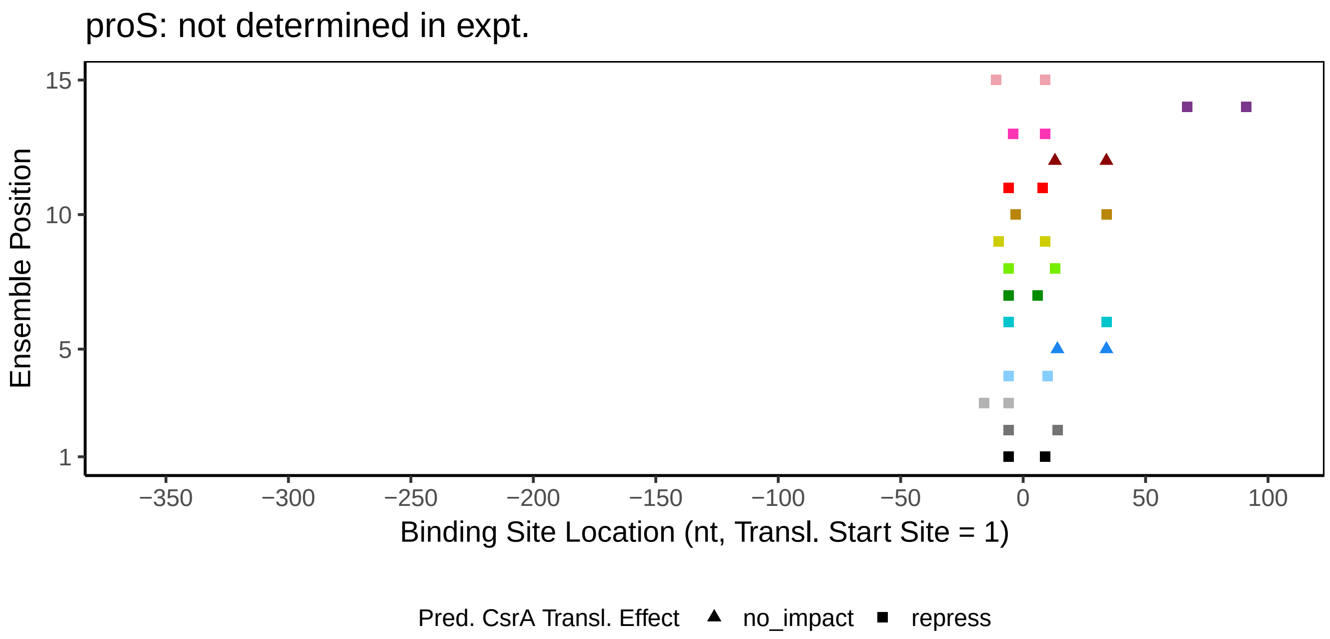

proS: not determined in expt.
Ensemble Position
Binding Site Location (nt, Transl. Start Site = 1)
Pred. CsrA Transl. Effect
no_impact
repress

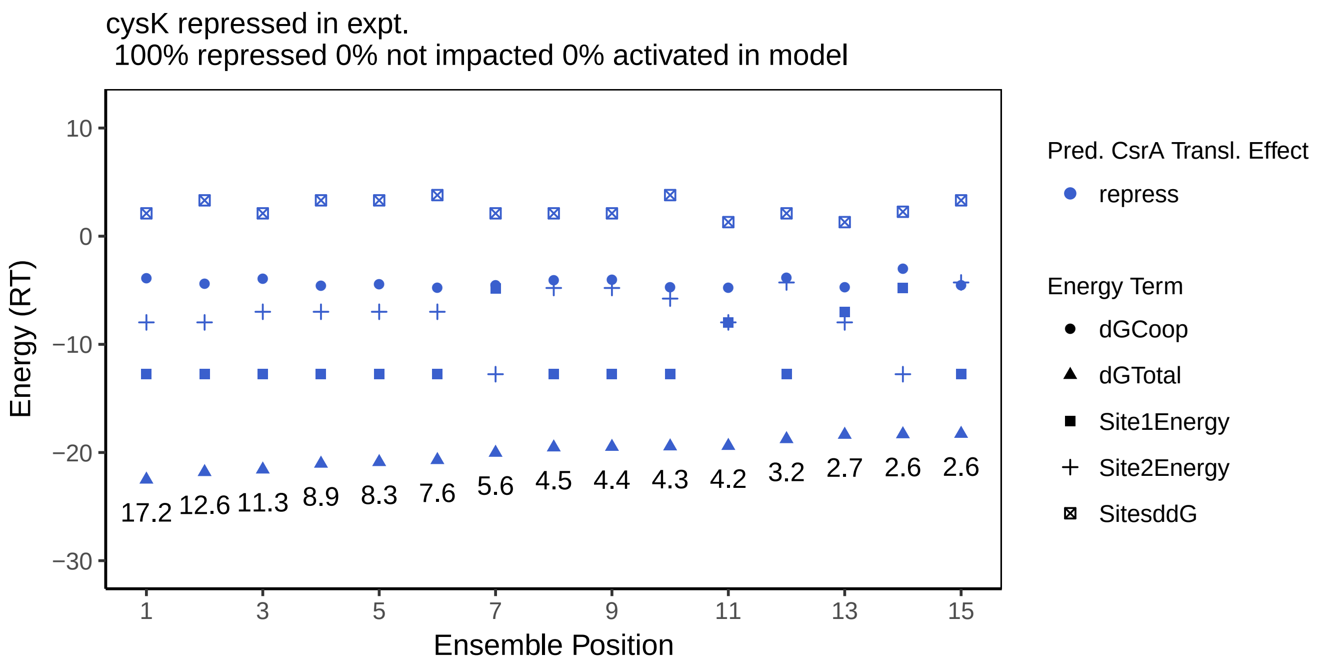
cysK repressed in expt.
100% repressed 0% not impacted 0% activated in model
Energy (RT)
10
0
−10
−20
−30
17.2
12.6
11.3
8.9
8.3
7.6
5.6
4.5
4.4
4.3
4.2
3.2
2.7
2.6
2.6
1
3
5
7
9
11
13
15
Ensemble Position
Pred. CsrA Transl. Effect
repress
Energy Term
dGCoop
dGTotal
Site1Energy
Site2Energy
SitesddG

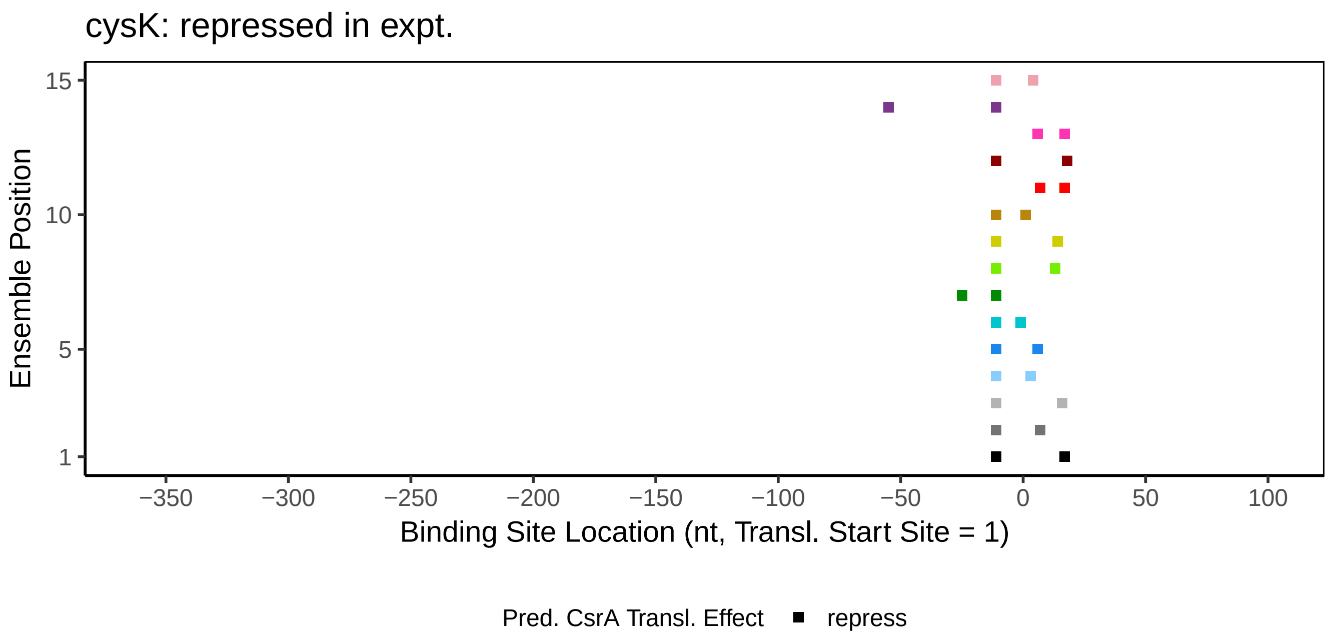
cysK: repressed in expt.
Ensemble Position
15
10
5
1
−350
−300
−250
−200
−150
−100
−50
0
50
100
Binding Site Location (nt, Transl. Start Site = 1)
Pred. CsrA Transl. Effect
repress

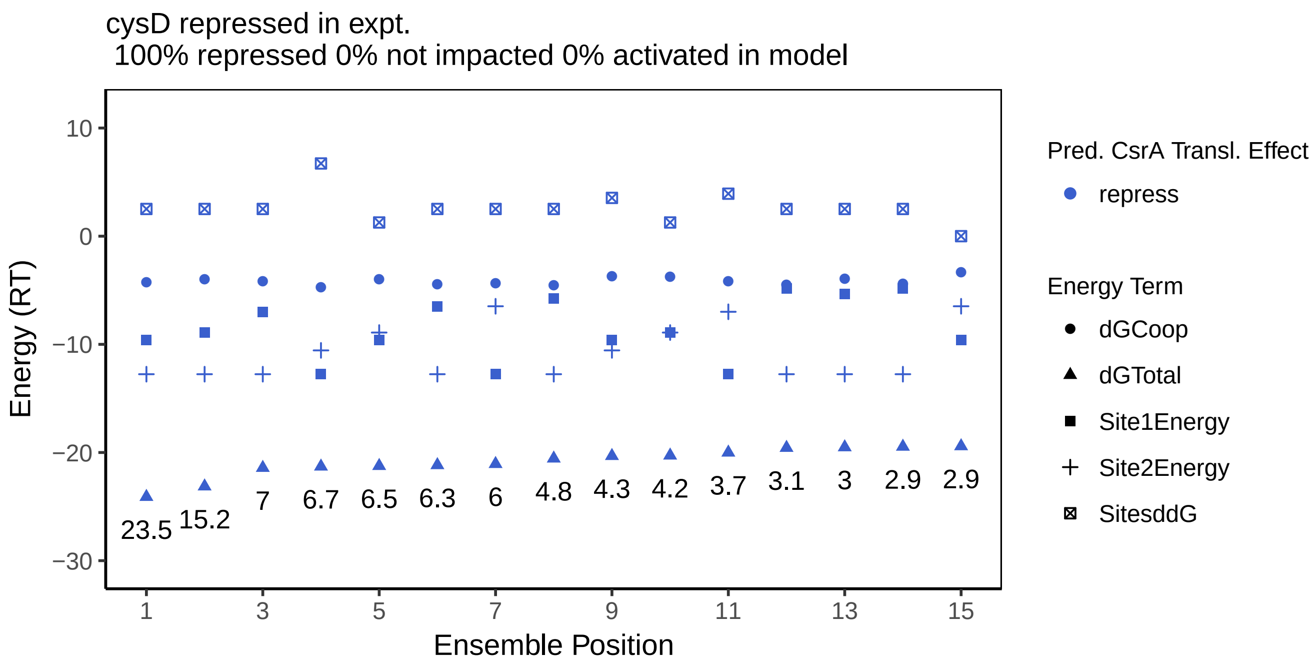

cysD repressed in expt.
100% repressed 0% not impacted 0% activated in model
Energy (RT)
Ensemble Position
23.5
15.2
7
6.7
6.5
6.3
6
4.8
4.3
4.2
3.7
3.1
3
2.9
2.9
Pred. CsrA Transl. Effect
repress
Energy Term
dGCoop
dGTotal
Site1Energy
Site2Energy
SitesddG

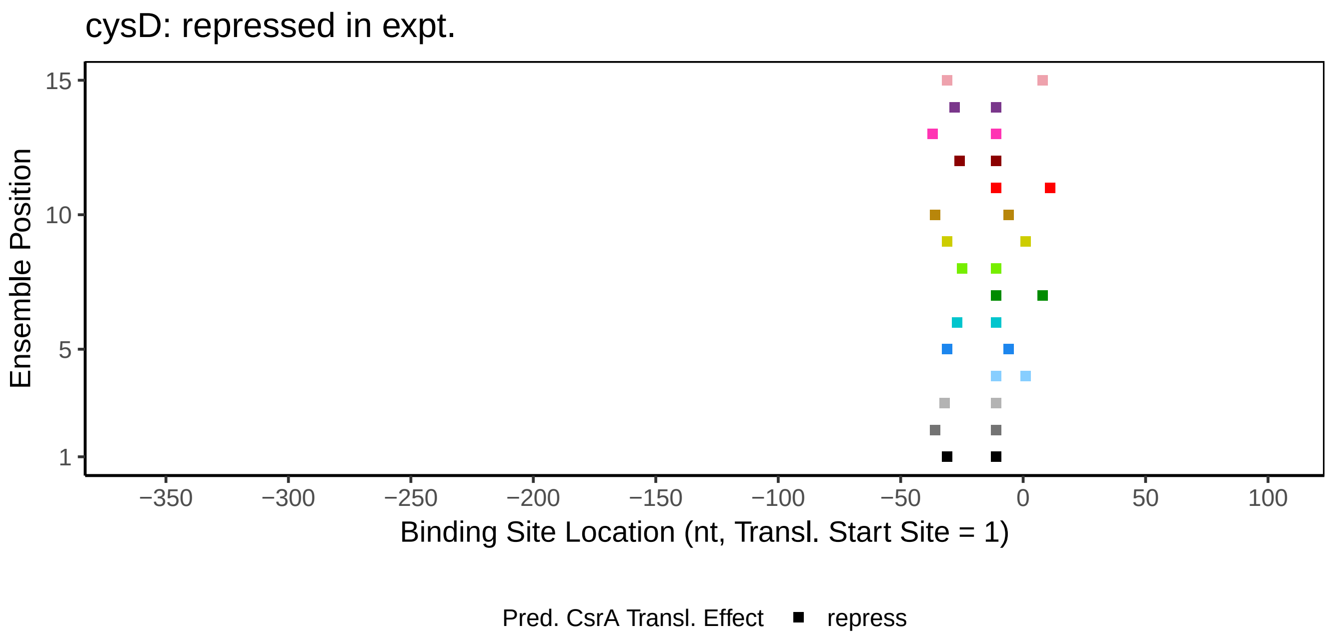

cysD: repressed in expt.
Ensemble Position
Binding Site Location (nt, Transl. Start Site = 1)
Pred. CsrA Transl. Effect
repress

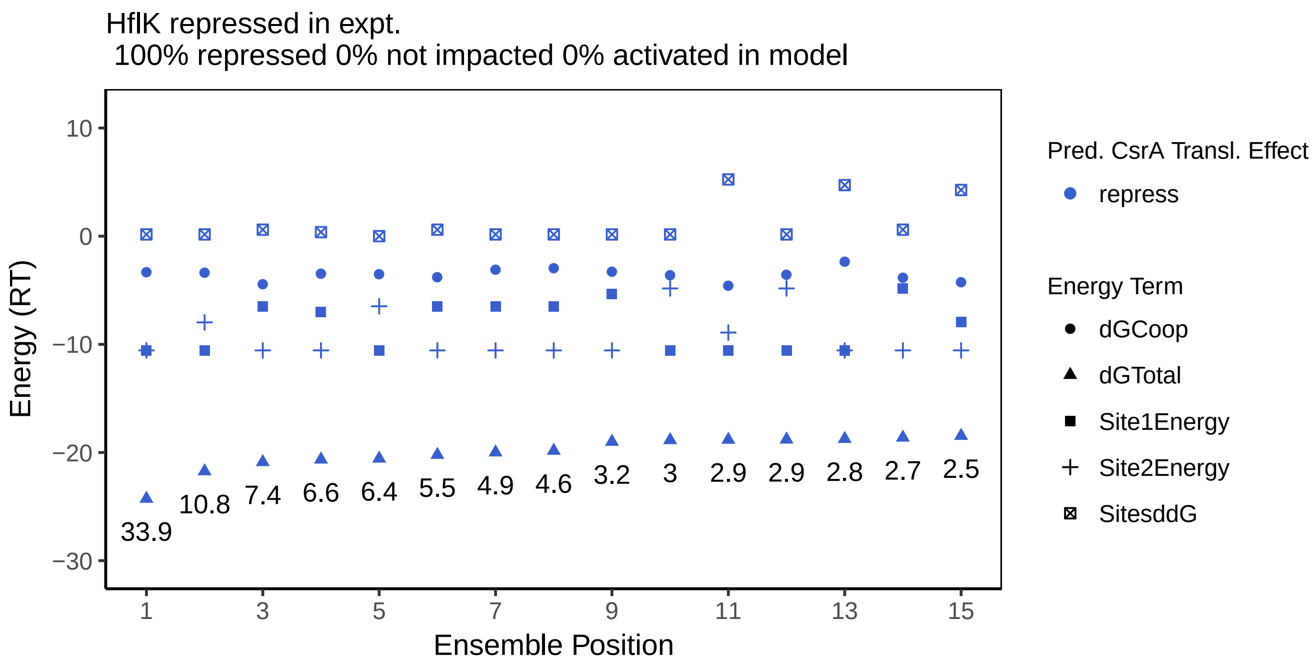
HflK repressed in expt.
100% repressed 0% not impacted 0% activated in model
Energy (RT)
Ensemble Position
33.9
10.8
7.4
6.6
6.4
5.5
4.9
4.6
3.2
3
2.9
2.9
2.8
2.7
2.5
Pred. CsrA Transl. Effect
repress
Energy Term
dGCoop
dGTotal
Site1Energy
Site2Energy
SitesddG

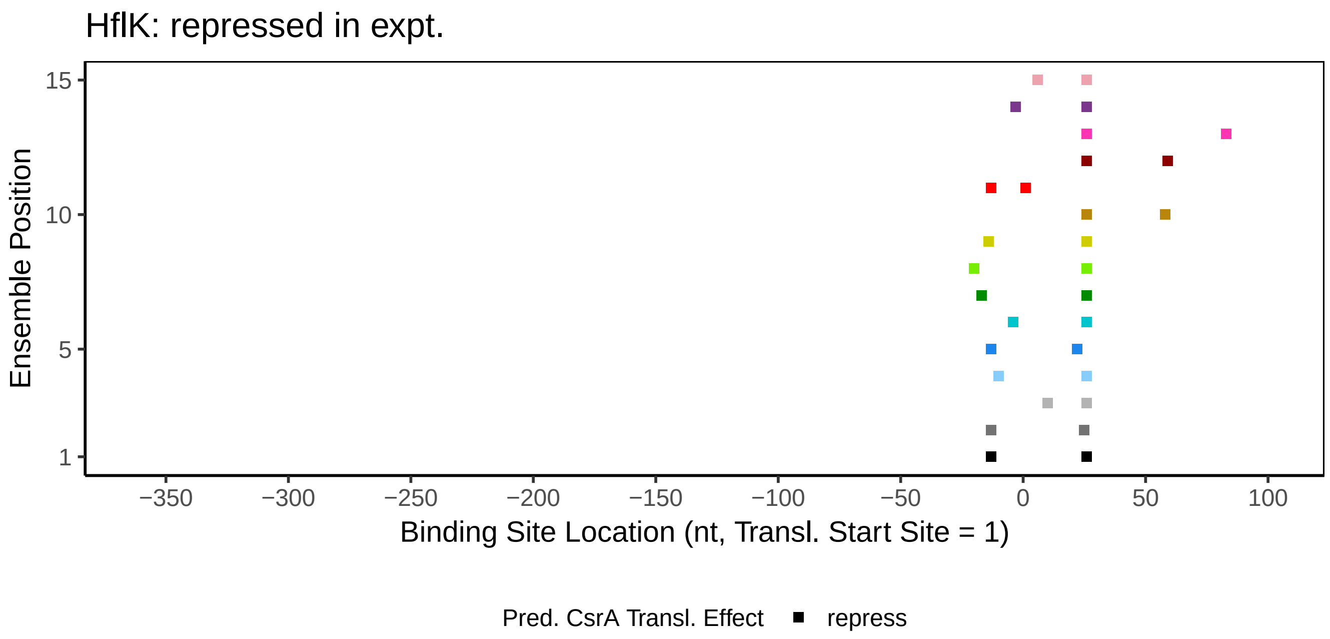
HflK: repressed in expt.
Ensemble Position
Binding Site Location (nt, Transl. Start Site = 1)
Pred. CsrA Transl. Effect
repress

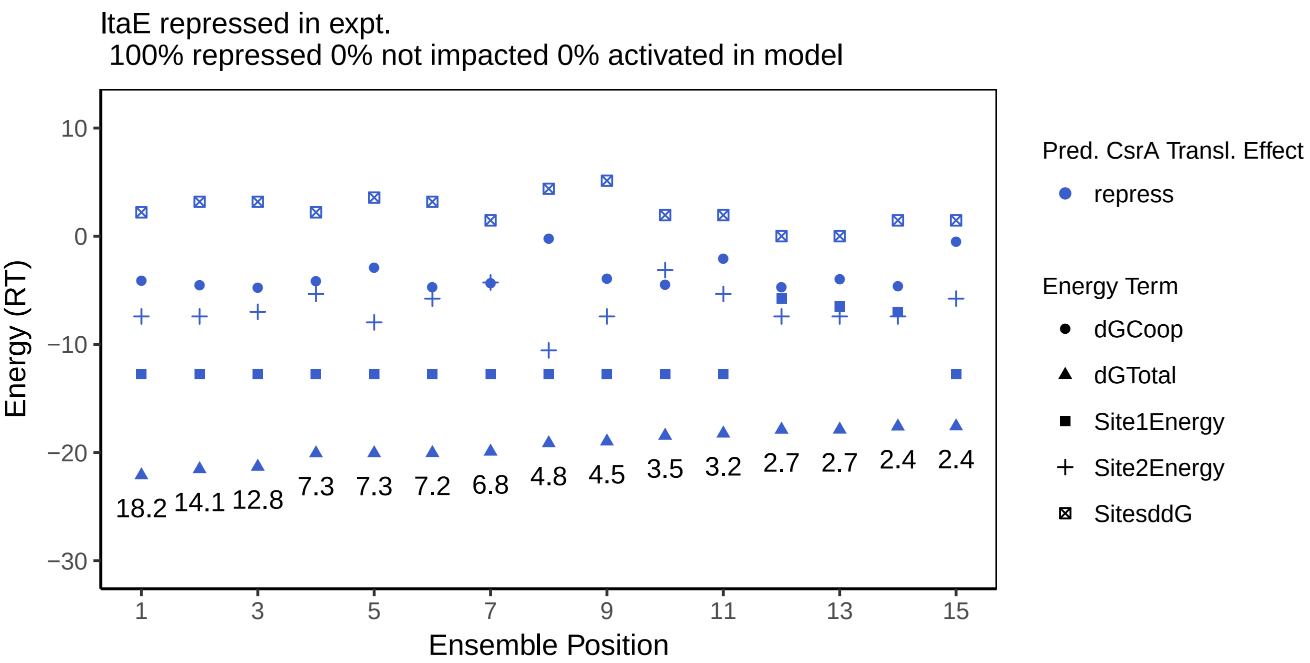
ltaE repressed in expt.
100% repressed 0% not impacted 0% activated in model
Energy (RT)
Ensemble Position
18.2 14.1 12.8 7.3 7.3 7.2 6.8 4.8 4.5 3.5 3.2 2.7 2.7 2.4 2.4
Pred. CsrA Transl. Effect
repress
Energy Term
dGCoop
dGTotal
Site1Energy
Site2Energy
SitesddG

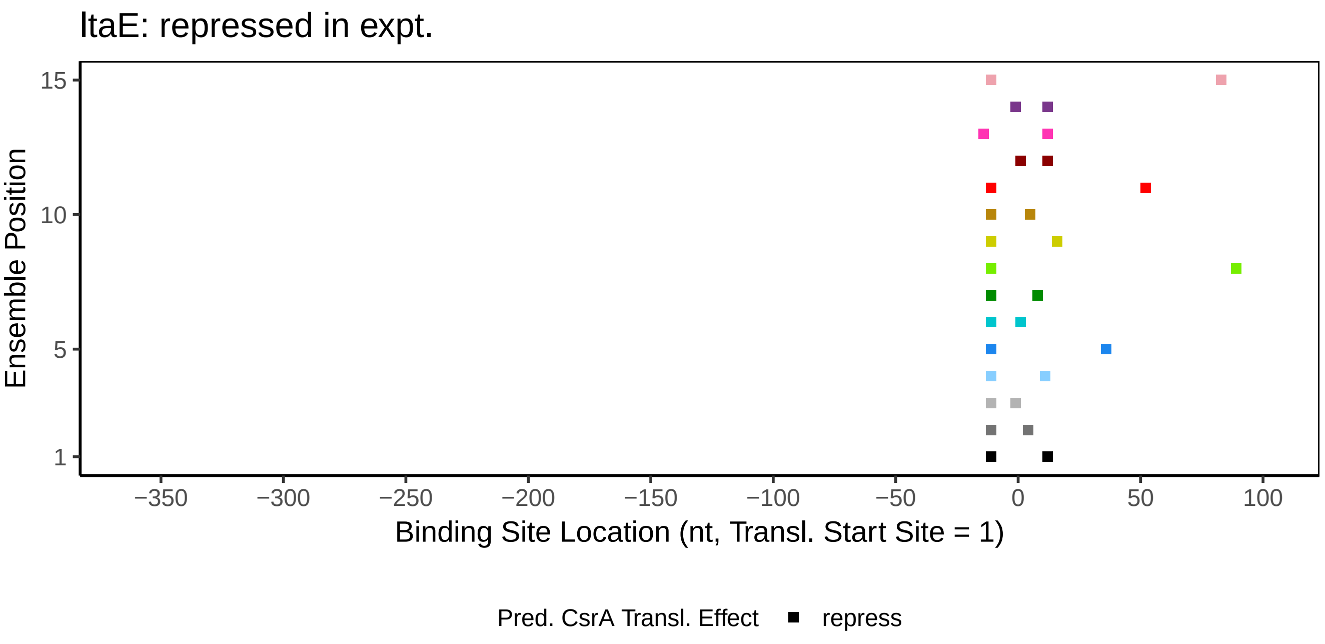
ltaE: repressed in expt.
Ensemble Position
Binding Site Location (nt, Transl. Start Site = 1)
Pred. CsrA Transl. Effect
repress

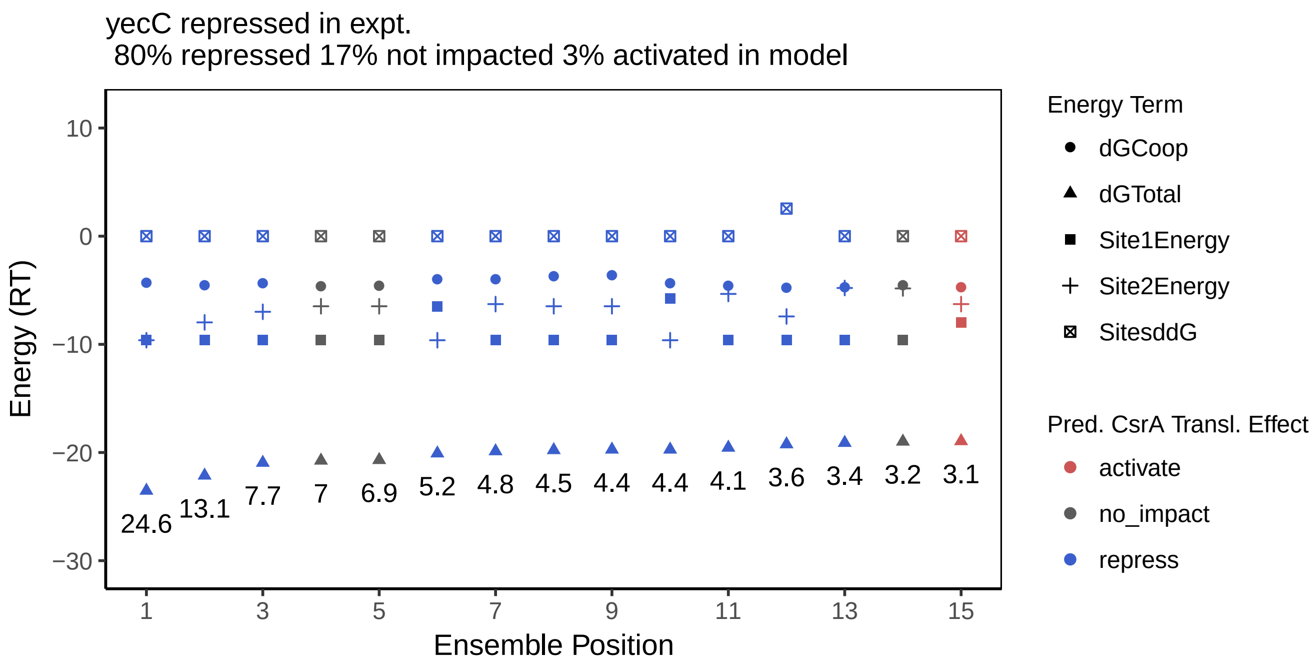
yecC repressed in expt.
80% repressed 17% not impacted 3% activated in model
Energy (RT)
Ensemble Position
24.6
13.1
7.7
7
6.9
5.2
4.8
4.5
4.4
4.4
4.1
3.6
3.4
3.2
3.1
Energy Term
dGCoop
dGTotal
Site1Energy
Site2Energy
SitesddG
Pred. CsrA Transl. Effect
activate
no_impact
repress

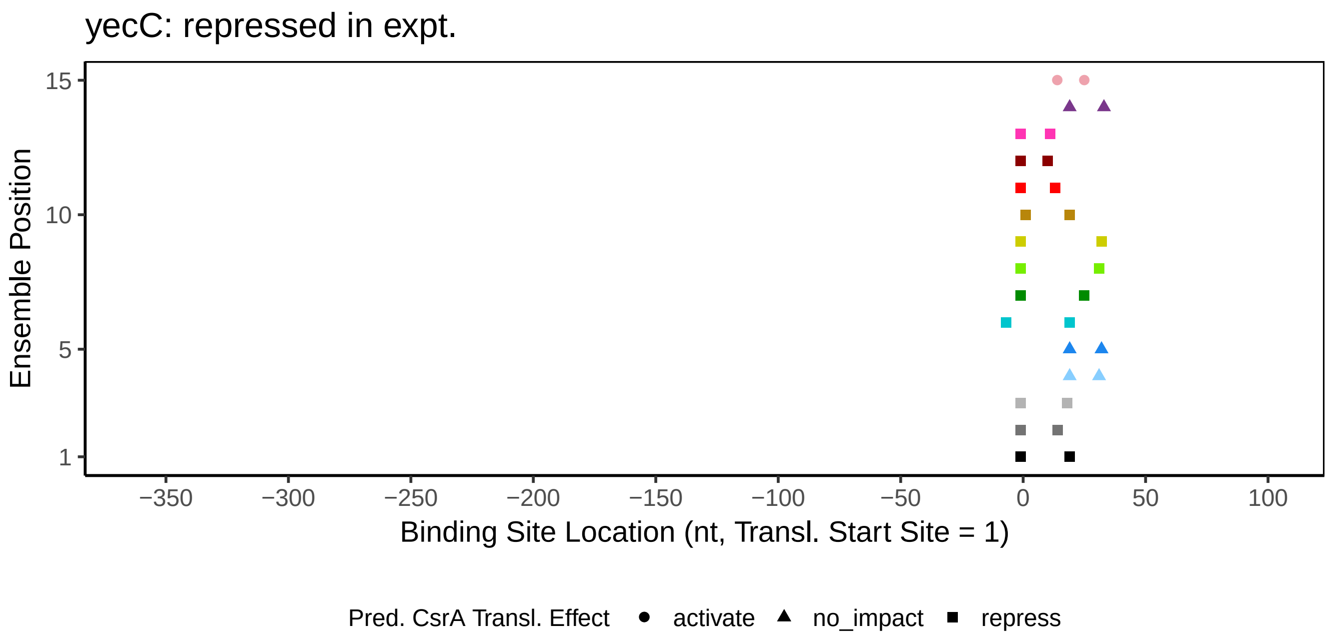
yecC: repressed in expt.
Ensemble Position
Binding Site Location (nt, Transl. Start Site = 1)
Pred. CsrA Transl. Effect
activate
no_impact
repress

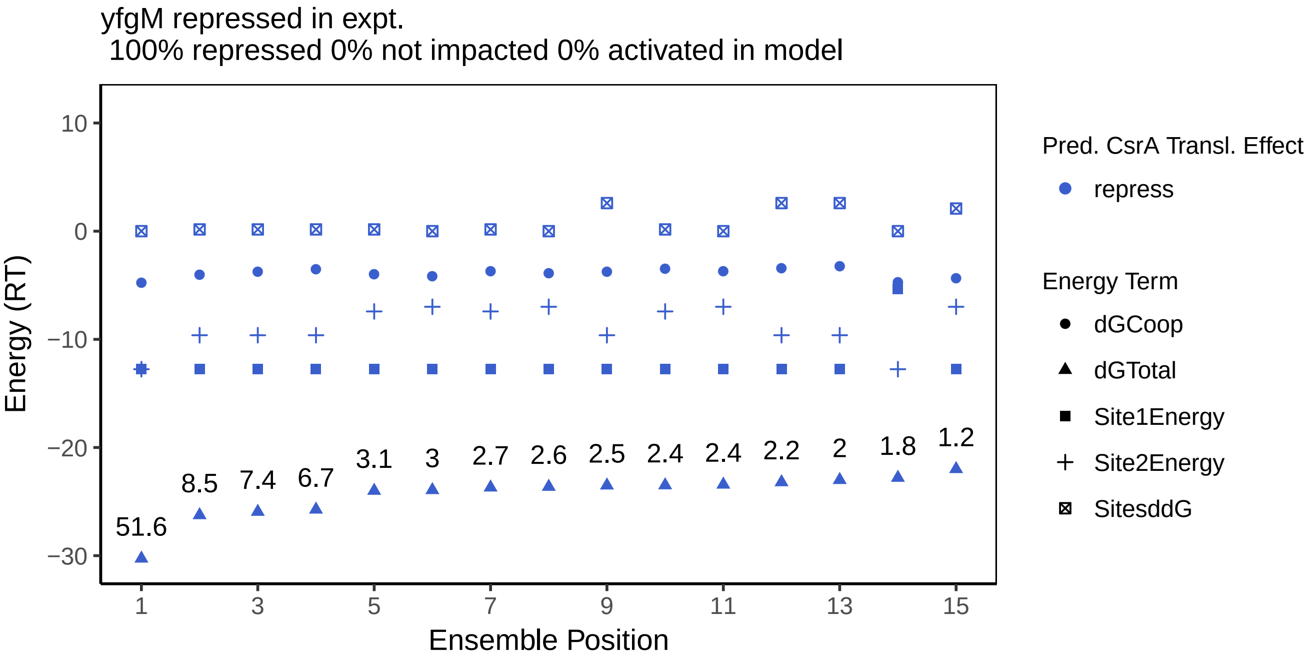

yfgM repressed in expt.
100% repressed 0% not impacted 0% activated in model
10
0
−10
−20
−30
Energy (RT)
51.6
8.5
7.4
6.7
3.1
3
2.7
2.6
2.5
2.4
2.4
2.2
2
1.8
1.2
1
3
5
7
9
11
13
15
Ensemble Position
Pred. CsrA Transl. Effect
repress
Energy Term
dGCoop
dGTotal
Site1Energy
Site2Energy
SitesddG

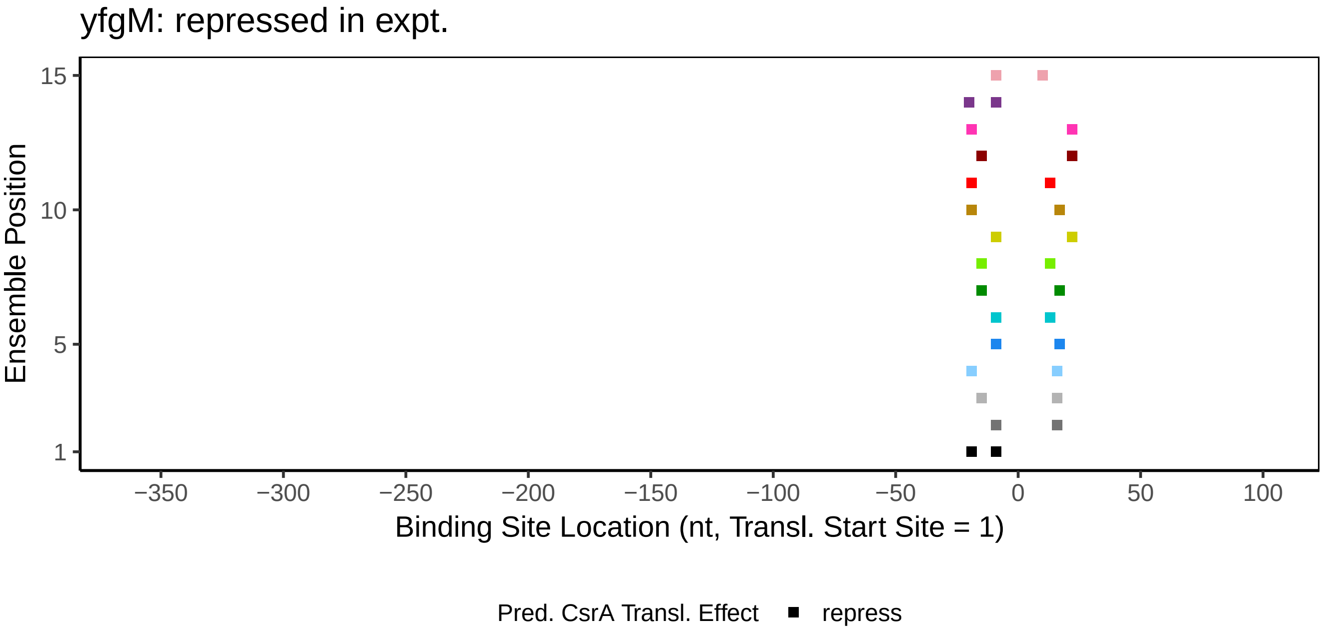

yfgM: repressed in expt.
15
10
5
1
Ensemble Position
−350
−300
−250
−200
−150
−100
−50
0
50
100
Binding Site Location (nt, Transl. Start Site = 1)
Pred. CsrA Transl. Effect
repress

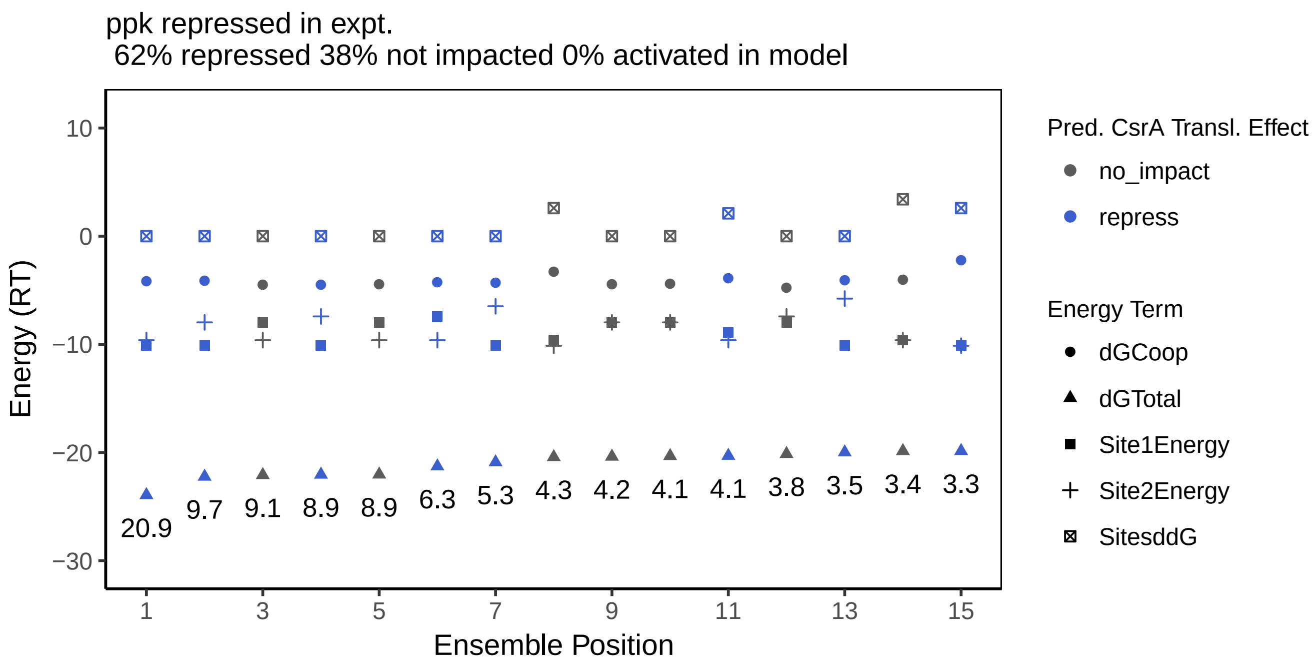
ppk repressed in expt.
62% repressed 38% not impacted 0% activated in model
Energy (RT)
Ensemble Position
20.9
9.7
9.1
8.9
8.9
6.3
5.3
4.3
4.2
4.1
4.1
3.8
3.5
3.4
3.3
Pred. CsrA Transl. Effect
no_impact
repress
Energy Term
dGCoop
dGTotal
Site1Energy
Site2Energy
SitesddG

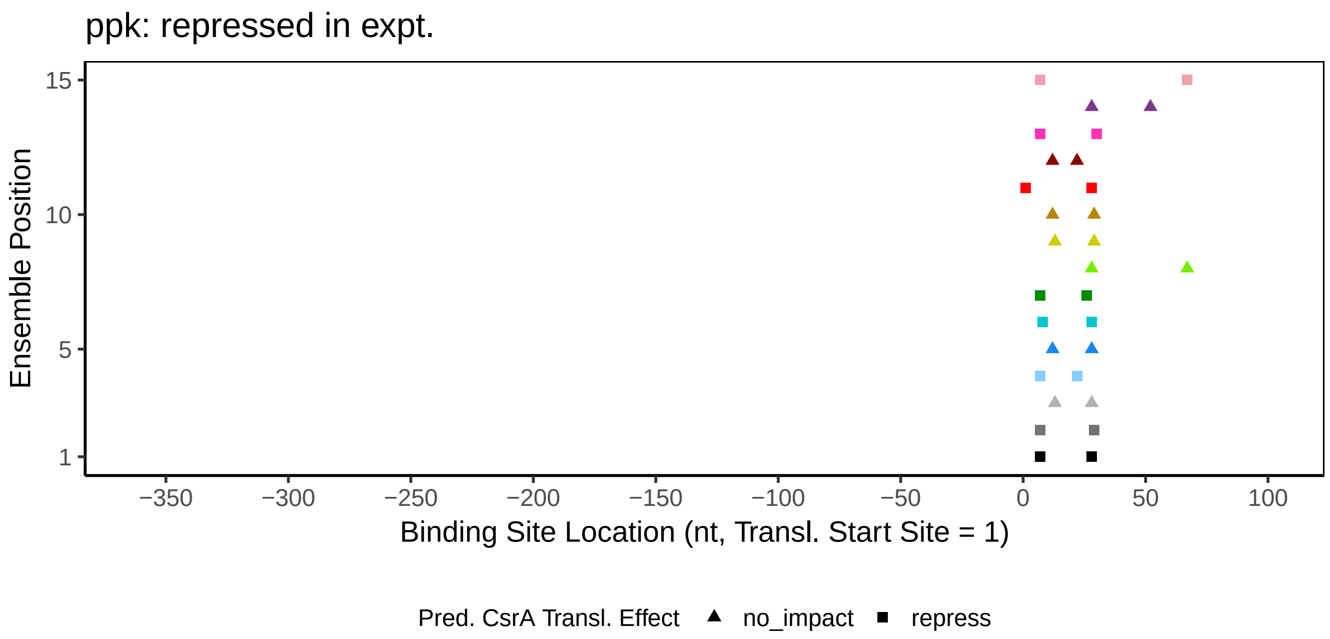
ppk: repressed in expt.
Ensemble Position
Binding Site Location (nt, Transl. Start Site = 1)
Pred. CsrA Transl. Effect
no_impact
repress

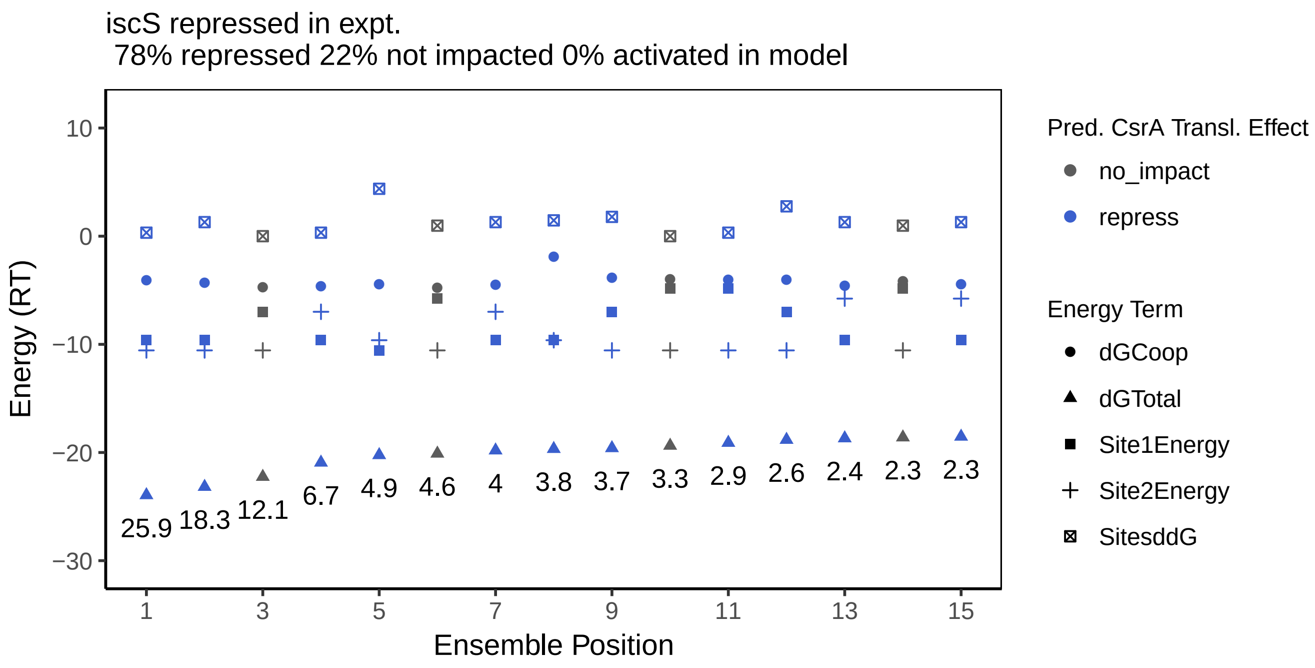

iscS repressed in expt.
78% repressed 22% not impacted 0% activated in model
Energy (RT)
Ensemble Position
Pred. CsrA Transl. Effect
no_impact
repress
Energy Term
dGCoop
dGTotal
Site1Energy
Site2Energy
SitesddG
25.9
18.3
12.1
6.7
4.9
4.6
4
3.8
3.7
3.3
2.9
2.6
2.4
2.3
2.3

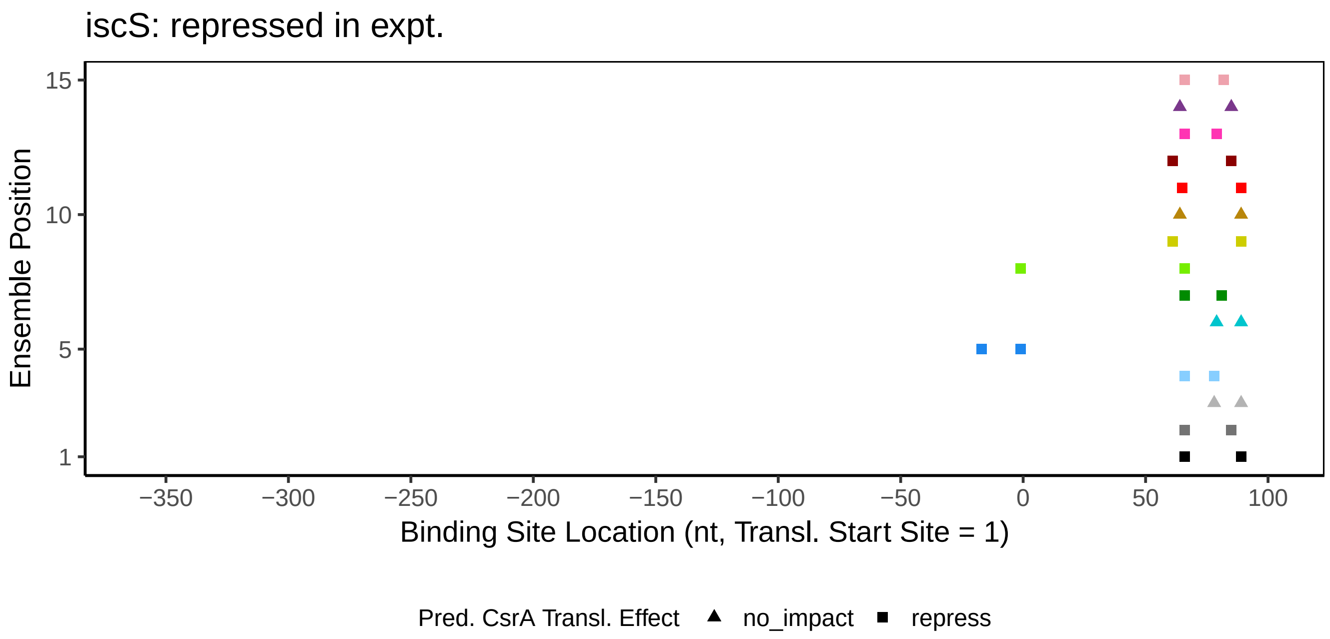

iscS: repressed in expt.
Ensemble Position
Binding Site Location (nt, Transl. Start Site = 1)
Pred. CsrA Transl. Effect
no_impact
repress

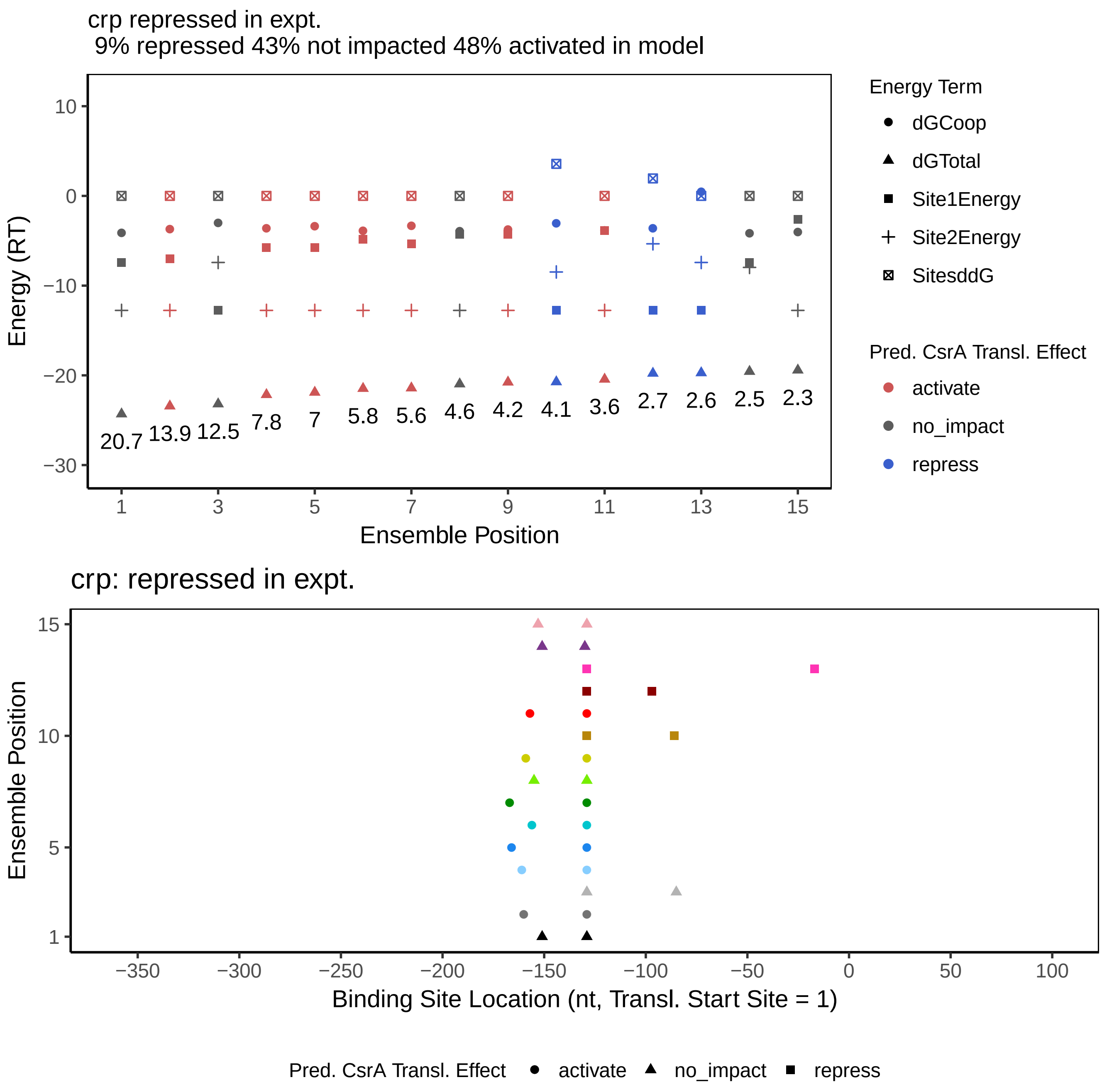

crp repressed in expt.
9% repressed 43% not impacted 48% activated in model
Energy (RT)
Ensemble Position
20.7 13.9 12.5 7.8 7 5.8 5.6 4.6 4.2 4.1 3.6 2.7 2.6 2.5 2.3
Energy Term
dGCoop
dGTotal
Site1Energy
Site2Energy
SitesddG
Pred. CsrA Transl. Effect
activate
no_impact
repress
crp: repressed in expt.
Ensemble Position
Binding Site Location (nt, Transl. Start Site = 1)
Pred. CsrA Transl. Effect activate no_impact repress

dnaK not determined in expt.
36% repressed 20% not impacted 44% activated in model

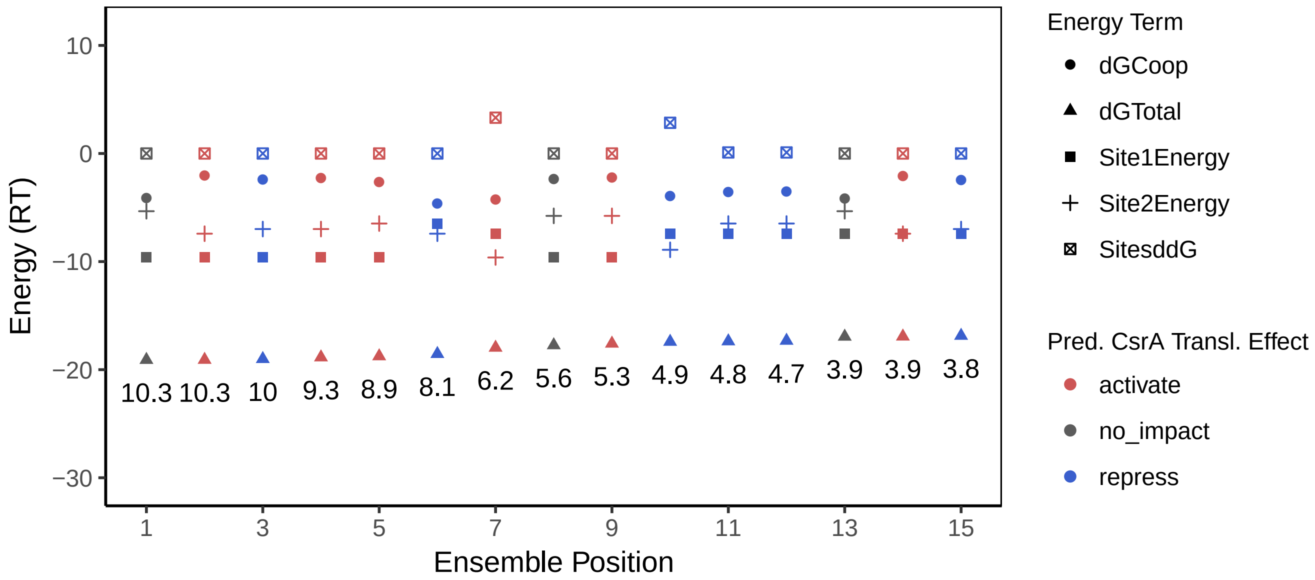

dnaK: not determined in expt.

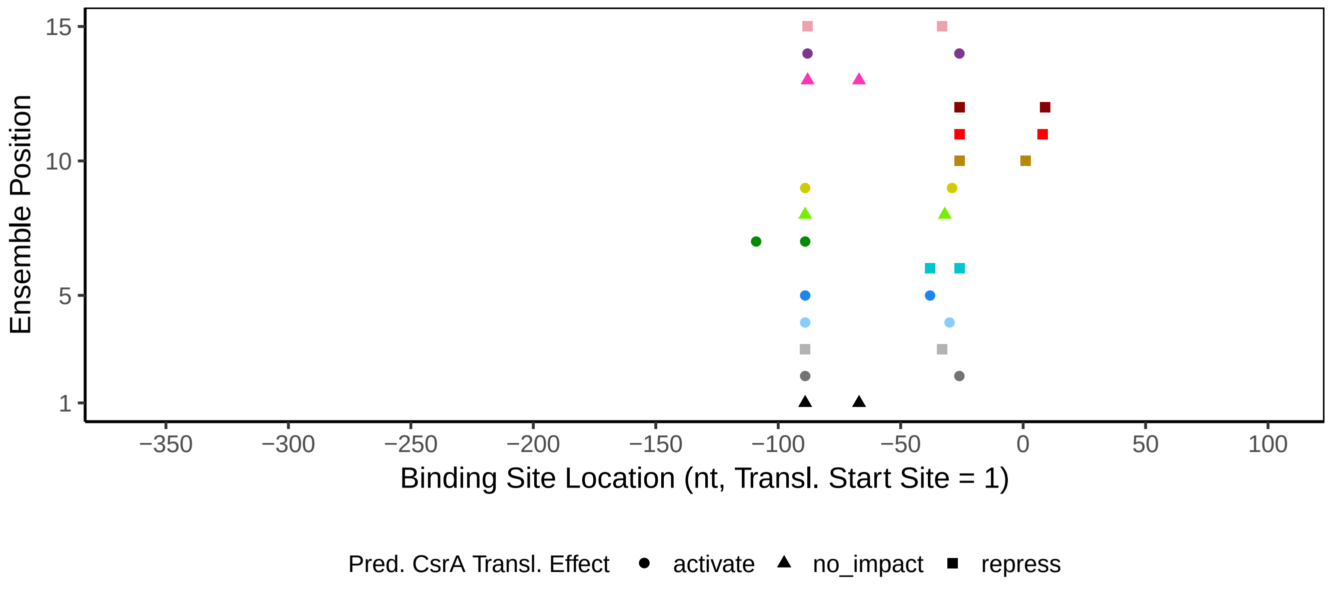

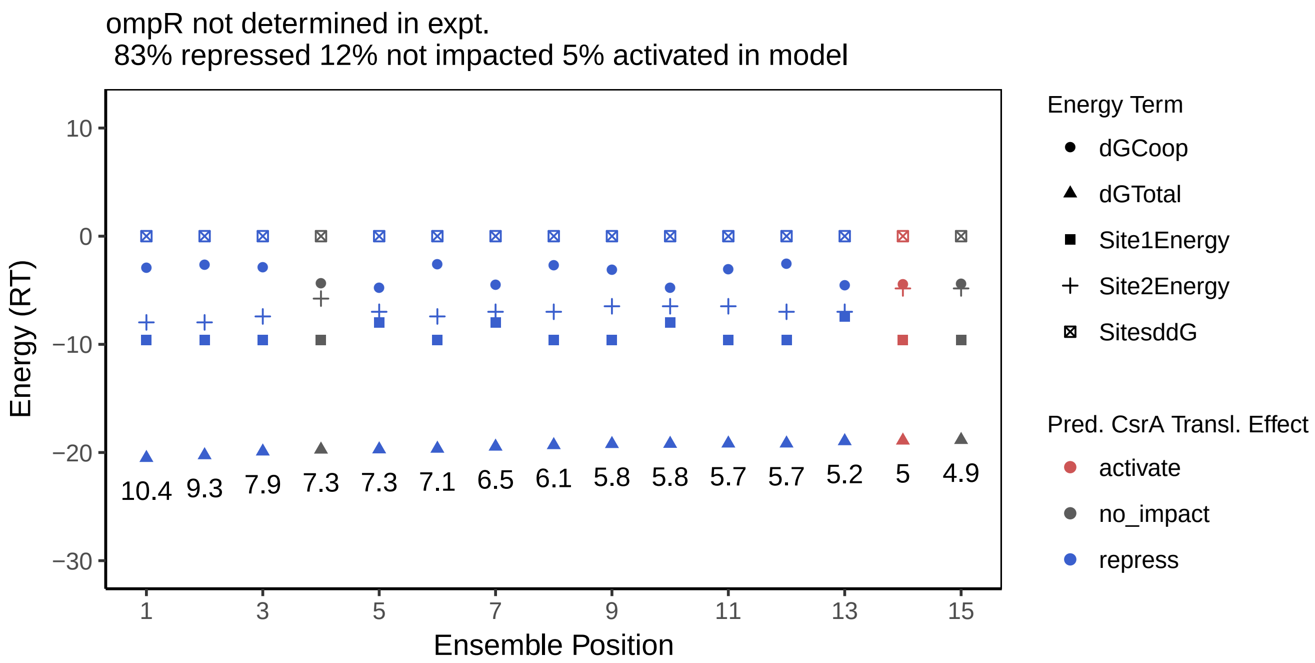
ompR not determined in expt.
83% repressed 12% not impacted 5% activated in model
Energy (RT)
10
0
−10
−20
−30
10.4 9.3 7.9 7.3 7.3 7.1 6.5 6.1 5.8 5.8 5.7 5.7 5.2 5 4.9
1 3 5 7 9 11 13 15
Ensemble Position
Energy Term
dGCoop
dGTotal
Site1Energy
Site2Energy
SitesddG
Pred. CsrA Transl. Effect
activate
no_impact
repress

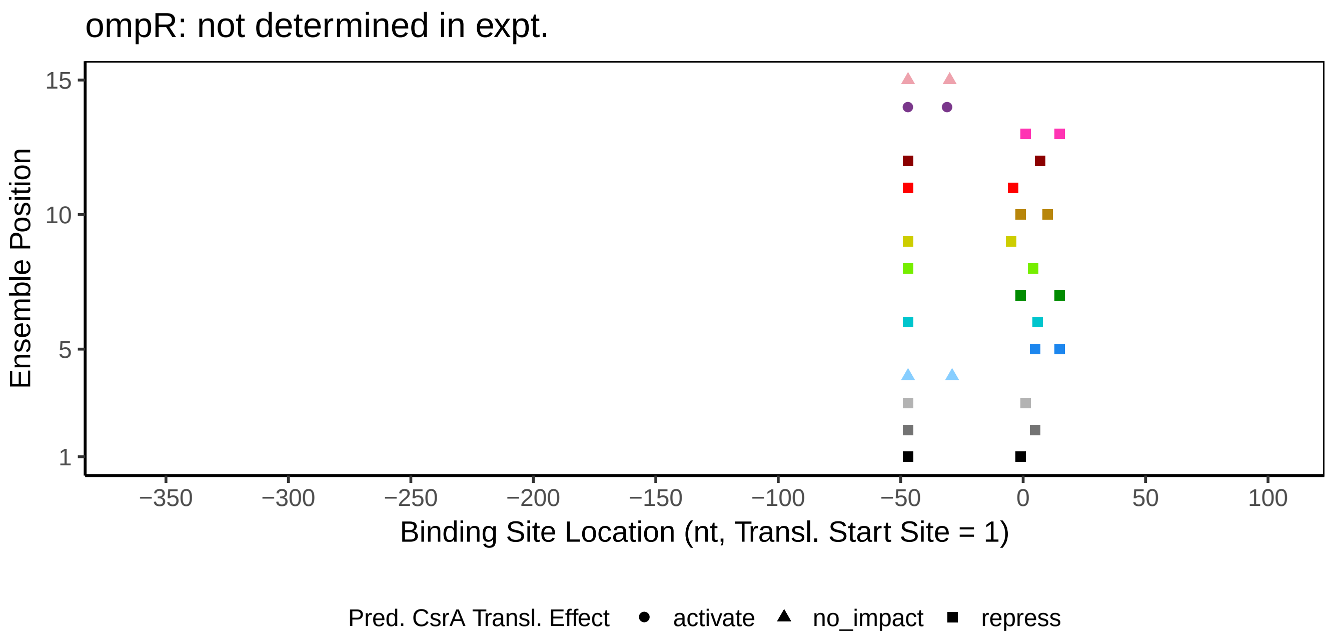
ompR: not determined in expt.
Ensemble Position
15
10
5
1
−350 −300 −250 −200 −150 −100 −50 0 50 100
Binding Site Location (nt, Transl. Start Site = 1)
Pred. CsrA Transl. Effect
activate
no_impact
repress

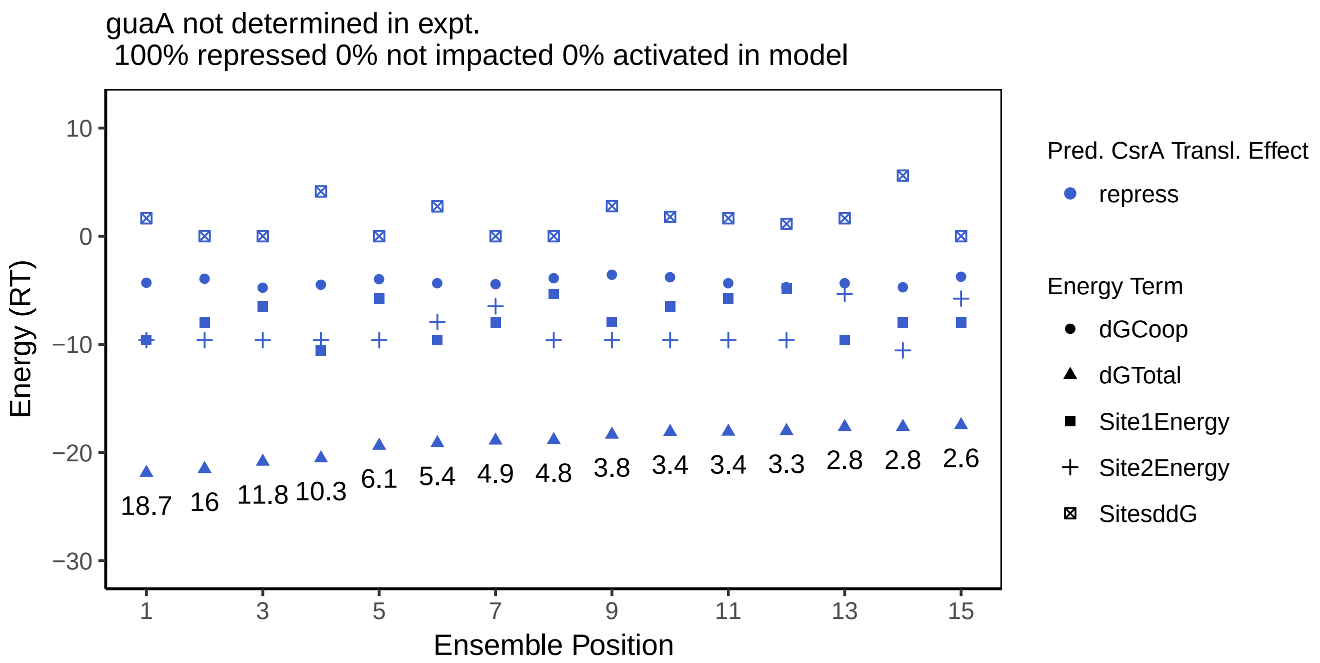
guaA not determined in expt.
100% repressed 0% not impacted 0% activated in model
Energy (RT)
Ensemble Position
18.7
16
11.8
10.3
6.1
5.4
4.9
4.8
3.8
3.4
3.4
3.3
2.8
2.8
2.6
Pred. CsrA Transl. Effect
repress
Energy Term
dGCoop
dGTotal
Site1Energy
Site2Energy
SitesddG

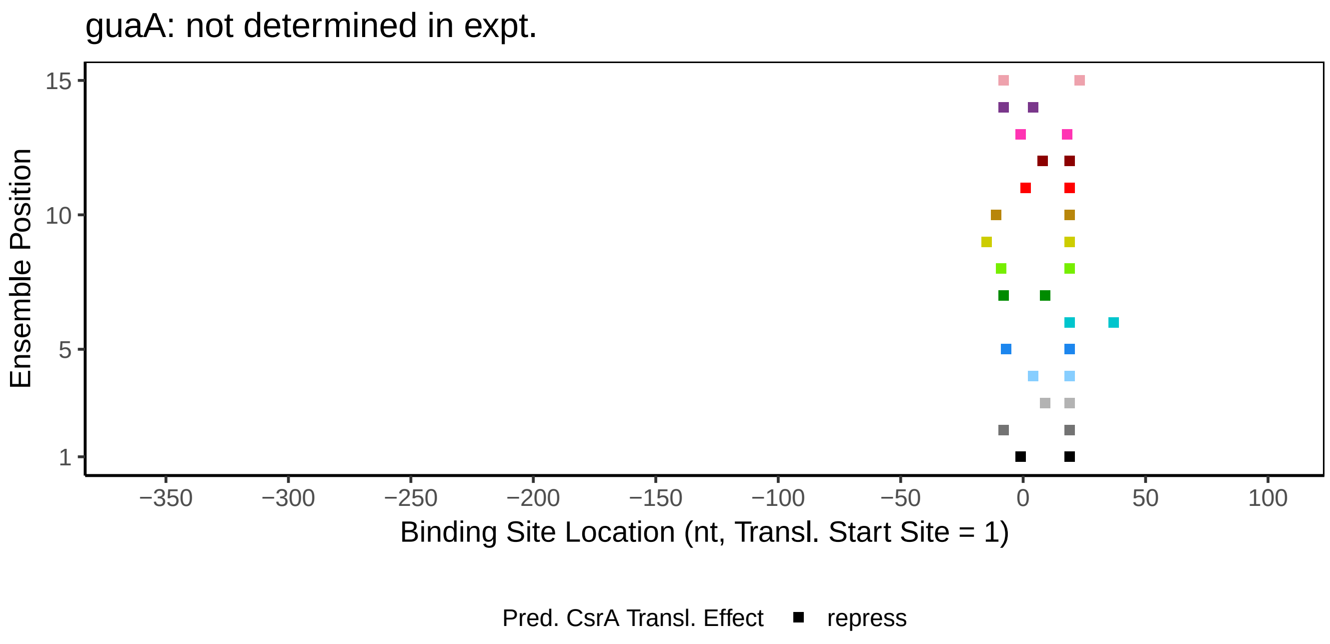
guaA: not determined in expt.
Ensemble Position
Binding Site Location (nt, Transl. Start Site = 1)
Pred. CsrA Transl. Effect
repress

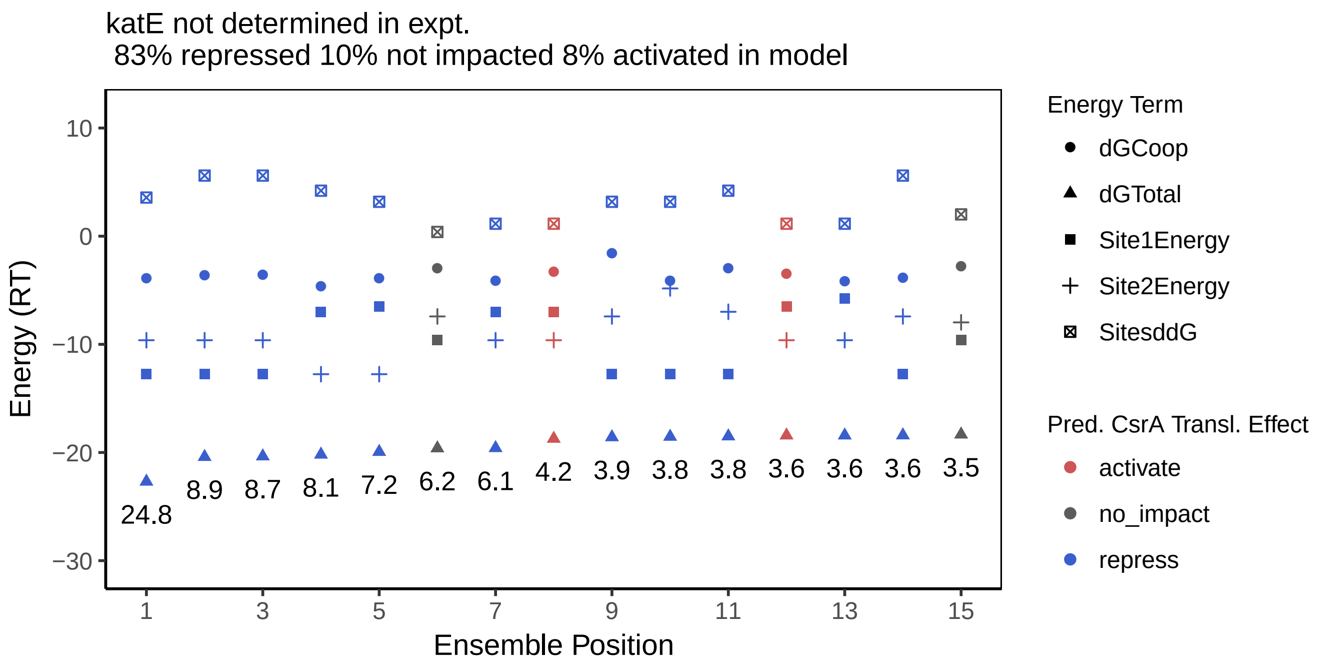

katE not determined in expt.
83% repressed 10% not impacted 8% activated in model
Energy (RT)
10
0
−10
−20
−30
24.8
8.9
8.7
8.1
7.2
6.2
6.1
4.2
3.9
3.8
3.8
3.6
3.6
3.6
3.5
1
3
5
7
9
11
13
15
Ensemble Position
Energy Term
dGCoop
dGTotal
Site1Energy
Site2Energy
SitesddG
Pred. CsrA Transl. Effect
activate
no_impact
repress

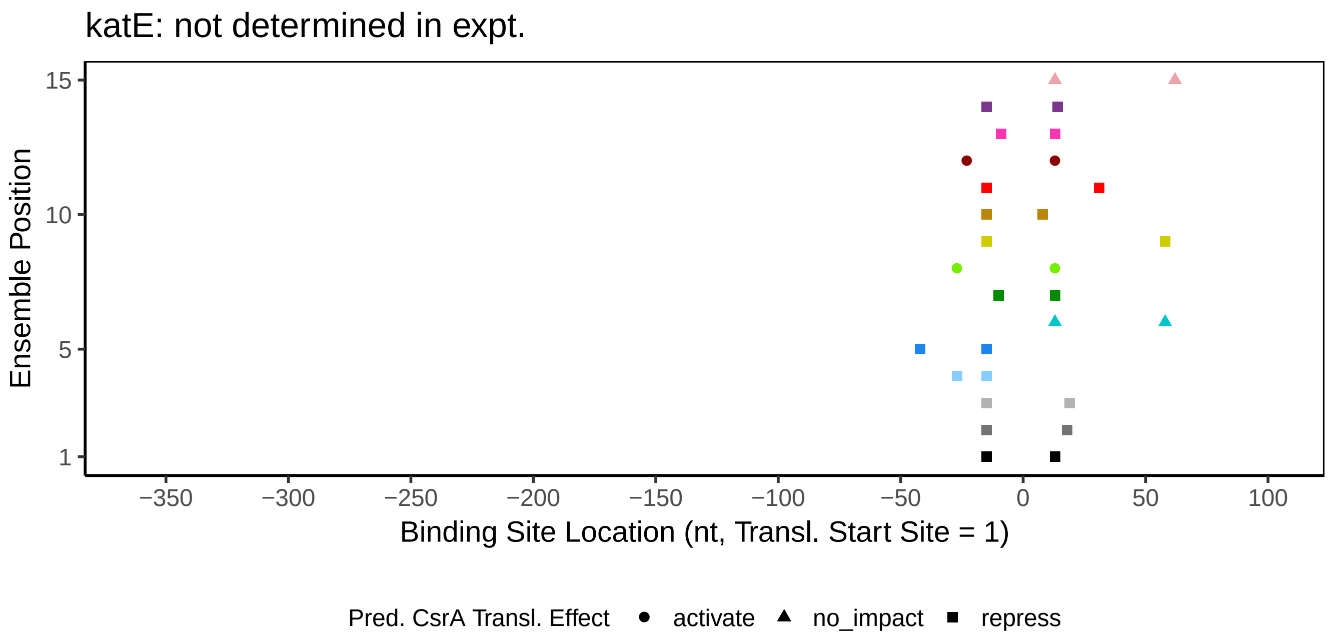

katE: not determined in expt.
Ensemble Position
15
10
5
1
−350
−300
−250
−200
−150
−100
−50
0
50
100
Binding Site Location (nt, Transl. Start Site = 1)
Pred. CsrA Transl. Effect
activate
no_impact
repress

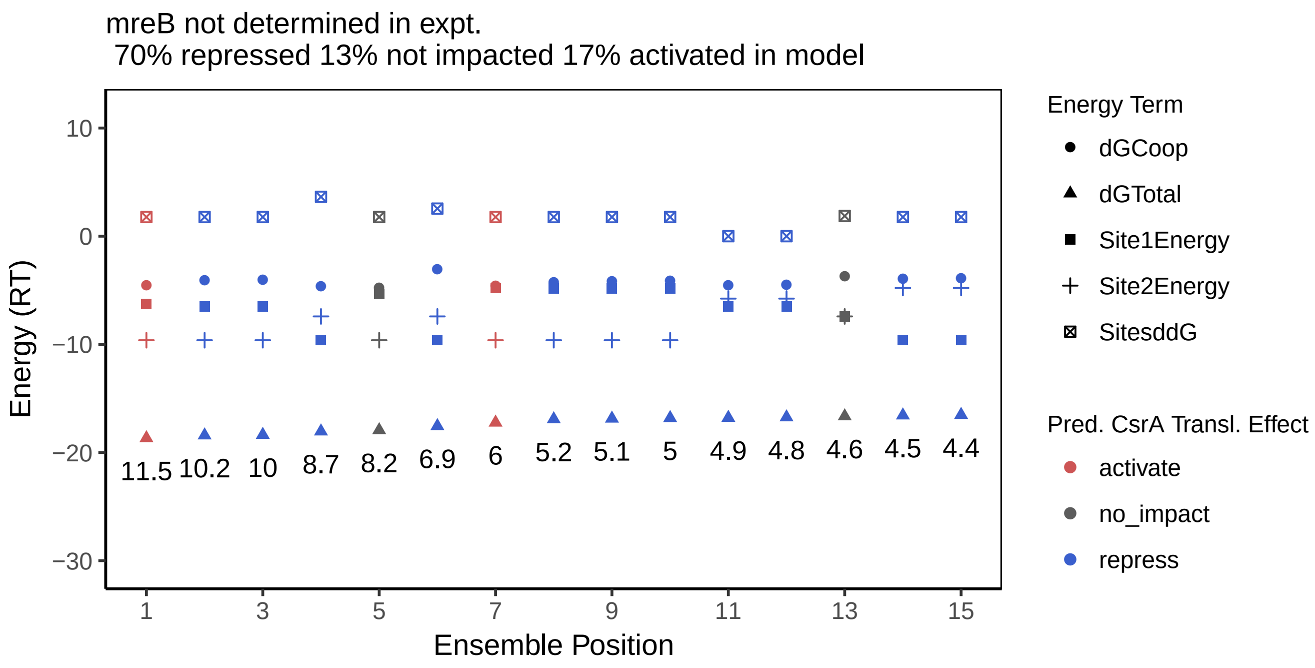

mreB not determined in expt.
70% repressed 13% not impacted 17% activated in model
Energy (RT)
Ensemble Position
11.5 10.2 10 8.7 8.2 6.9 6 5.2 5.1 5 4.9 4.8 4.6 4.5 4.4
Energy Term
dGCoop
dGTotal
Site1Energy
Site2Energy
SitesddG
Pred. CsrA Transl. Effect
activate
no_impact
repress

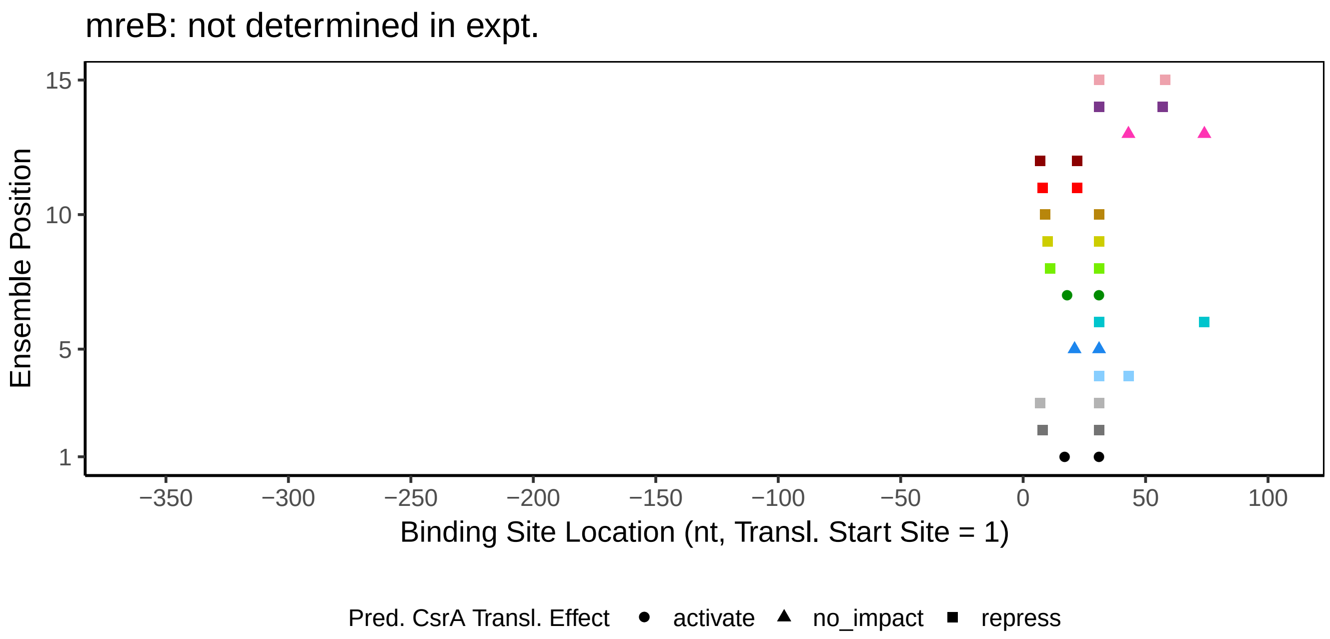

mreB: not determined in expt.
Ensemble Position
Binding Site Location (nt, Transl. Start Site = 1)
Pred. CsrA Transl. Effect
activate
no_impact
repress

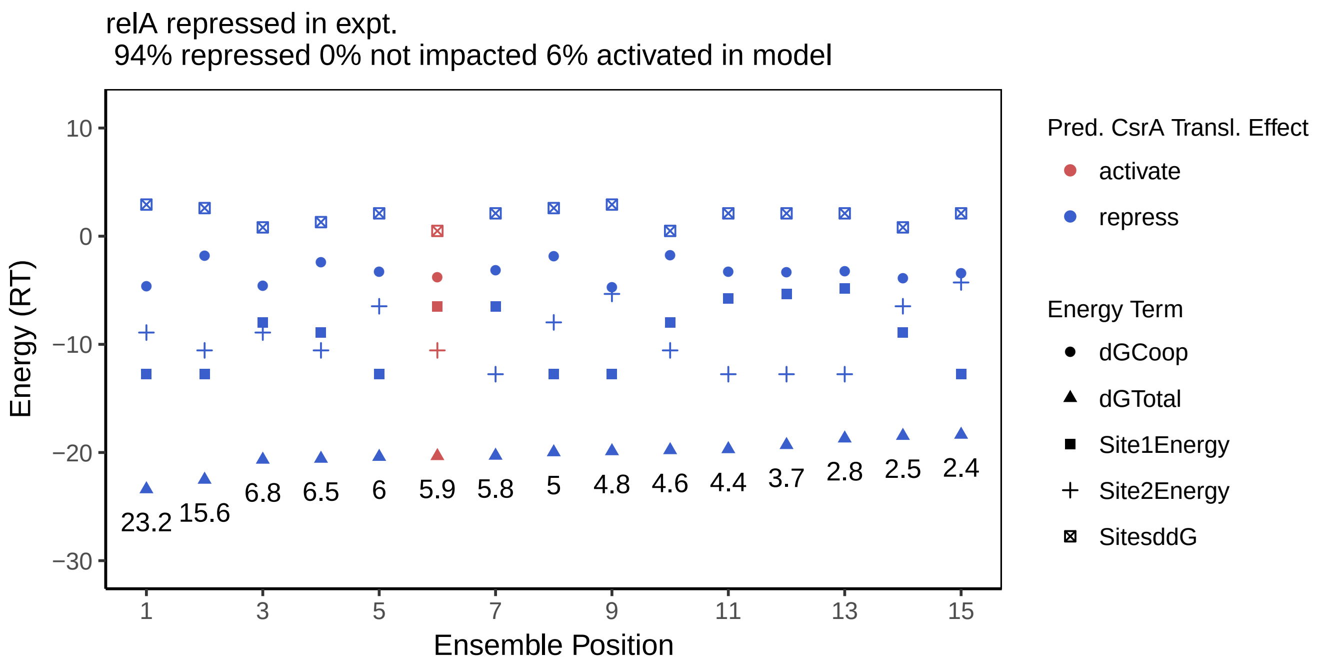

relA repressed in expt.
94% repressed 0% not impacted 6% activated in model
Energy (RT)
Ensemble Position
23.2
15.6
6.8
6.5
6
5.9
5.8
5
4.8
4.6
4.4
3.7
2.8
2.5
2.4
Pred. CsrA Transl. Effect
activate
repress
Energy Term
dGCoop
dGTotal
Site1Energy
Site2Energy
SitesddG

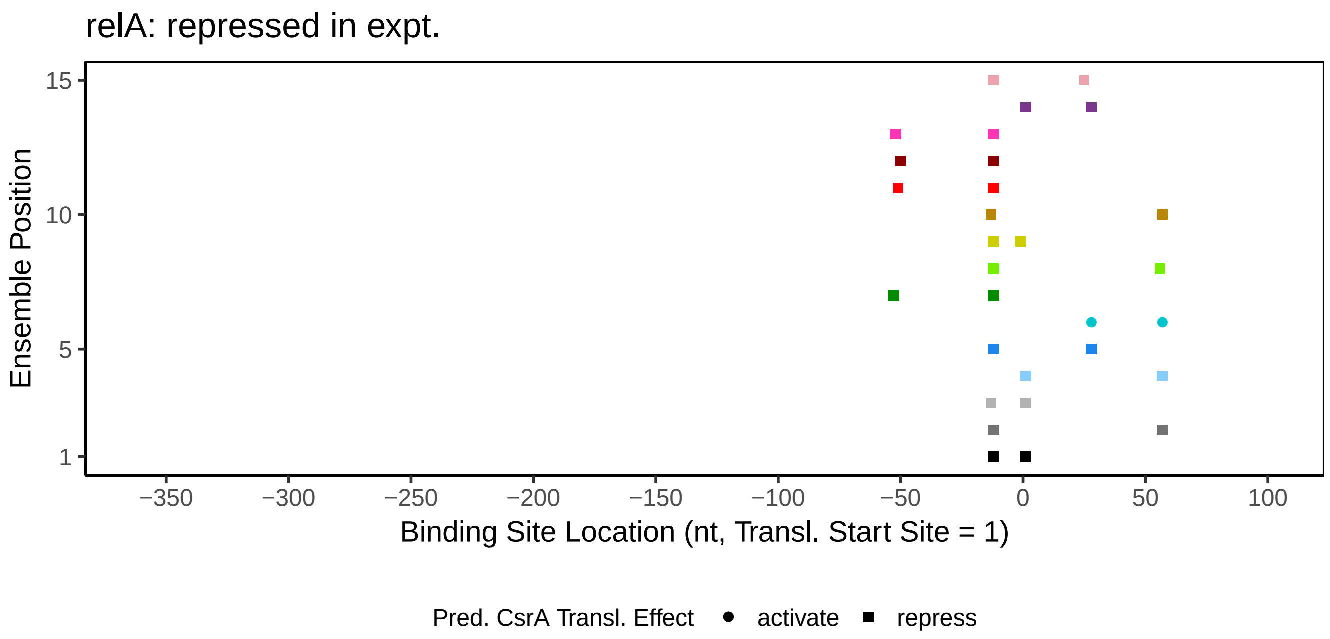

relA: repressed in expt.
Ensemble Position
Binding Site Location (nt, Transl. Start Site = 1)
Pred. CsrA Transl. Effect
activate
repress

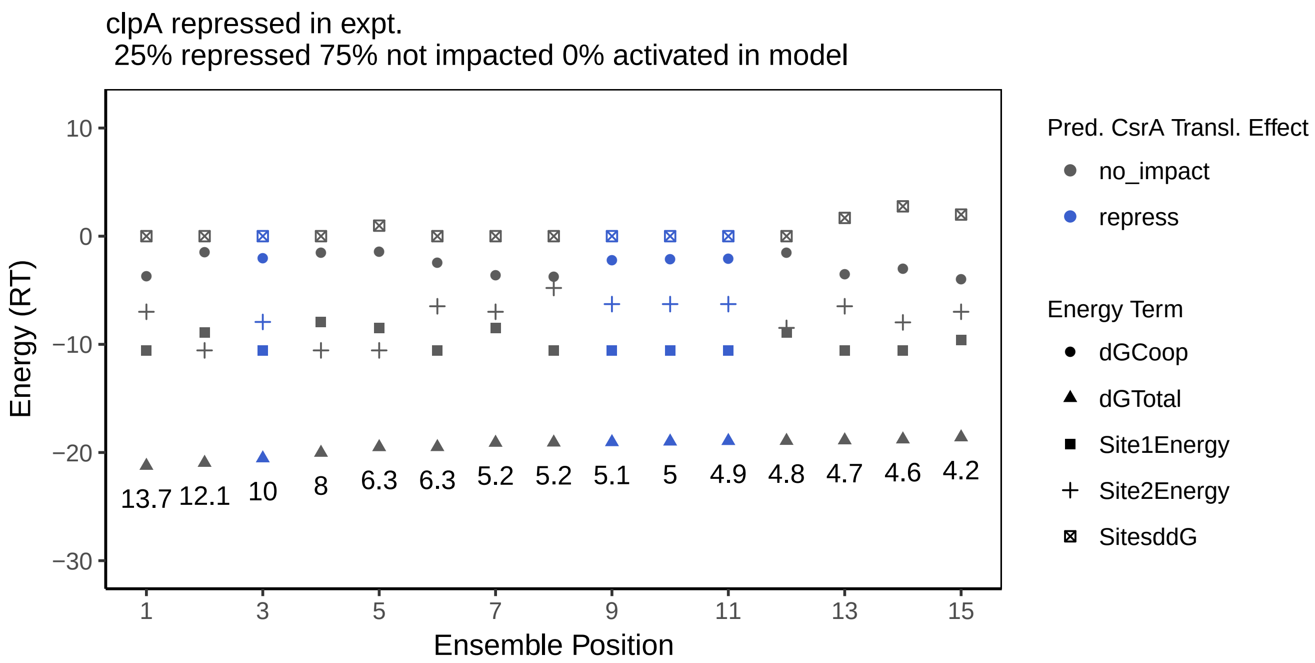

clpA repressed in expt.
25% repressed 75% not impacted 0% activated in model
Energy (RT)
Ensemble Position
13.7 12.1 10 8 6.3 6.3 5.2 5.2 5.1 5 4.9 4.8 4.7 4.6 4.2
Pred. CsrA Transl. Effect
no_impact
repress
Energy Term
dGCoop
dGTotal
Site1Energy
Site2Energy
SitesddG

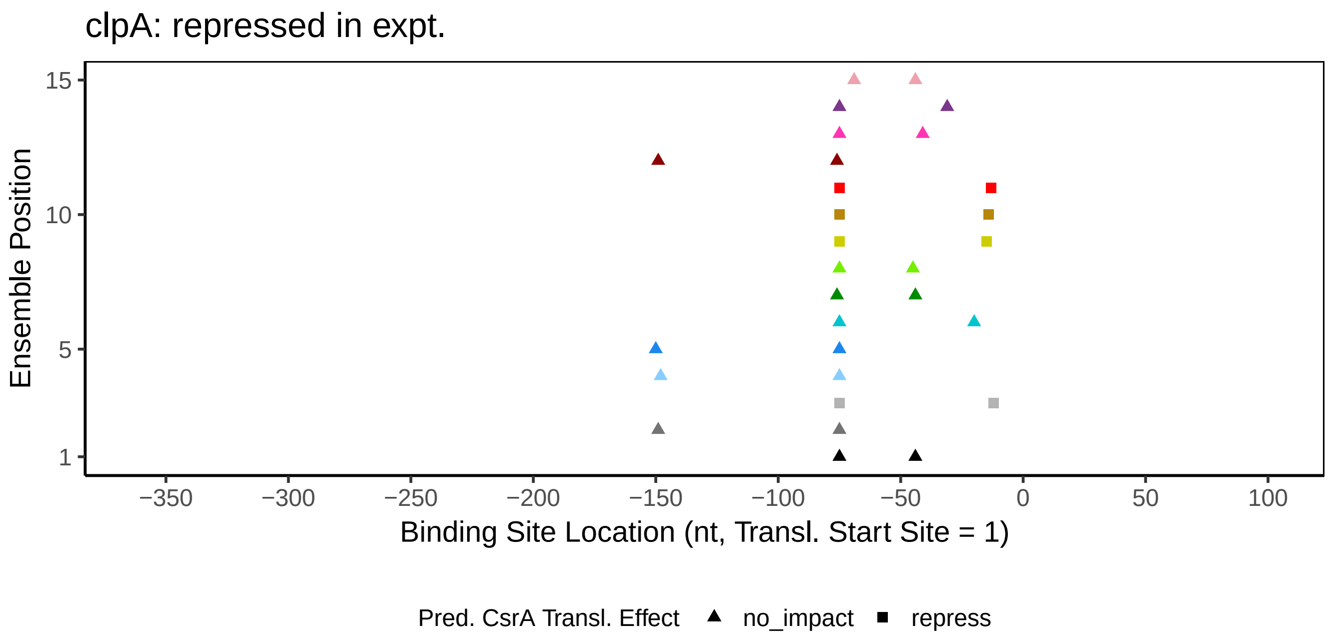

clpA: repressed in expt.
Ensemble Position
Binding Site Location (nt, Transl. Start Site = 1)
Pred. CsrA Transl. Effect
no_impact
repress

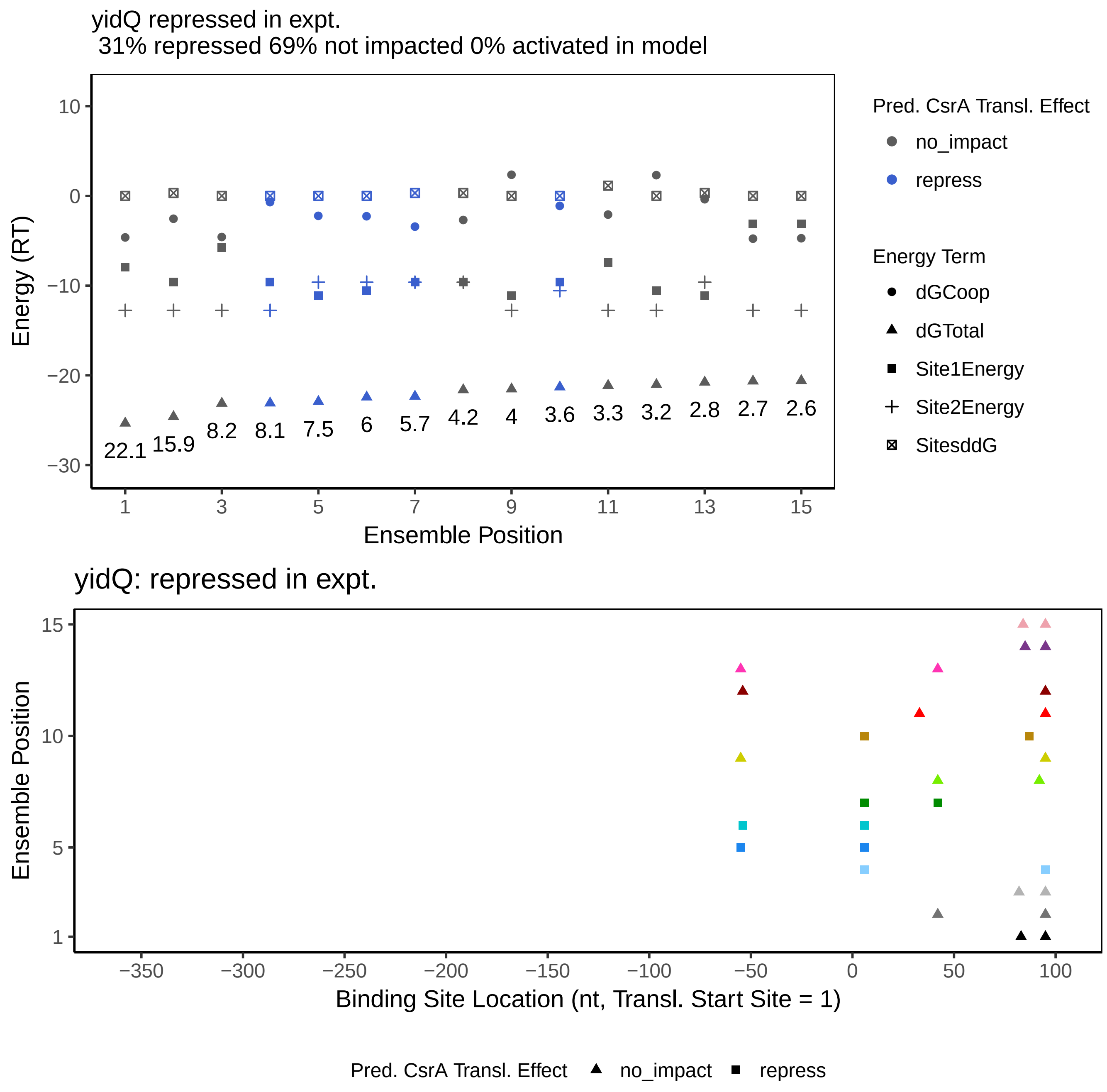

yidQ repressed in expt.
31% repressed 69% not impacted 0% activated in model
Energy (RT)
Ensemble Position
22.1
15.9
8.2
8.1
7.5
6
5.7
4.2
4
3.6
3.3
3.2
2.8
2.7
2.6
Pred. CsrA Transl. Effect
no_impact
repress
Energy Term
dGCoop
dGTotal
Site1Energy
Site2Energy
SitesddG
yidQ: repressed in expt.
Ensemble Position
Binding Site Location (nt, Transl. Start Site = 1)
Pred. CsrA Transl. Effect
no_impact
repress

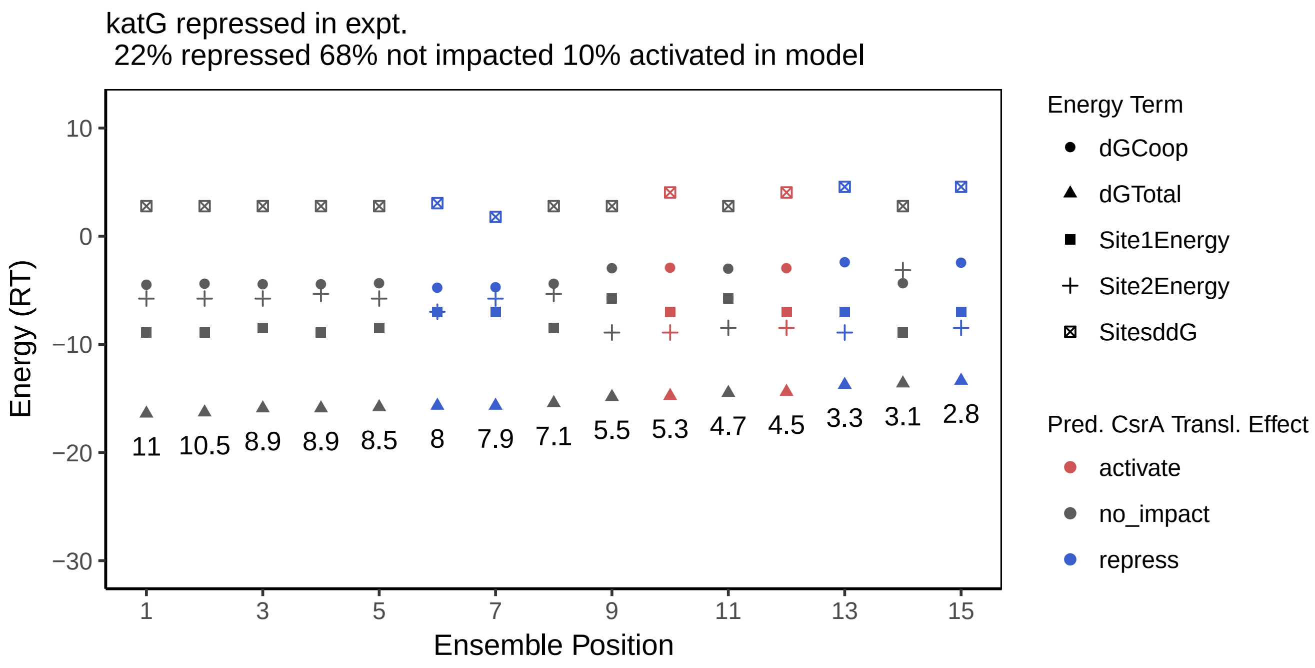
katG repressed in expt.
22% repressed 68% not impacted 10% activated in model
Energy (RT)
10
0
−10
−20
−30
11
10.5
8.9
8.9
8.5
8
7.9
7.1
5.5
5.3
4.7
4.5
3.3
3.1
2.8
1
3
5
7
9
11
13
15
Ensemble Position
Energy Term
dGCoop
dGTotal
Site1Energy
Site2Energy
SitesddG
Pred. CsrA Transl. Effect
activate
no_impact
repress

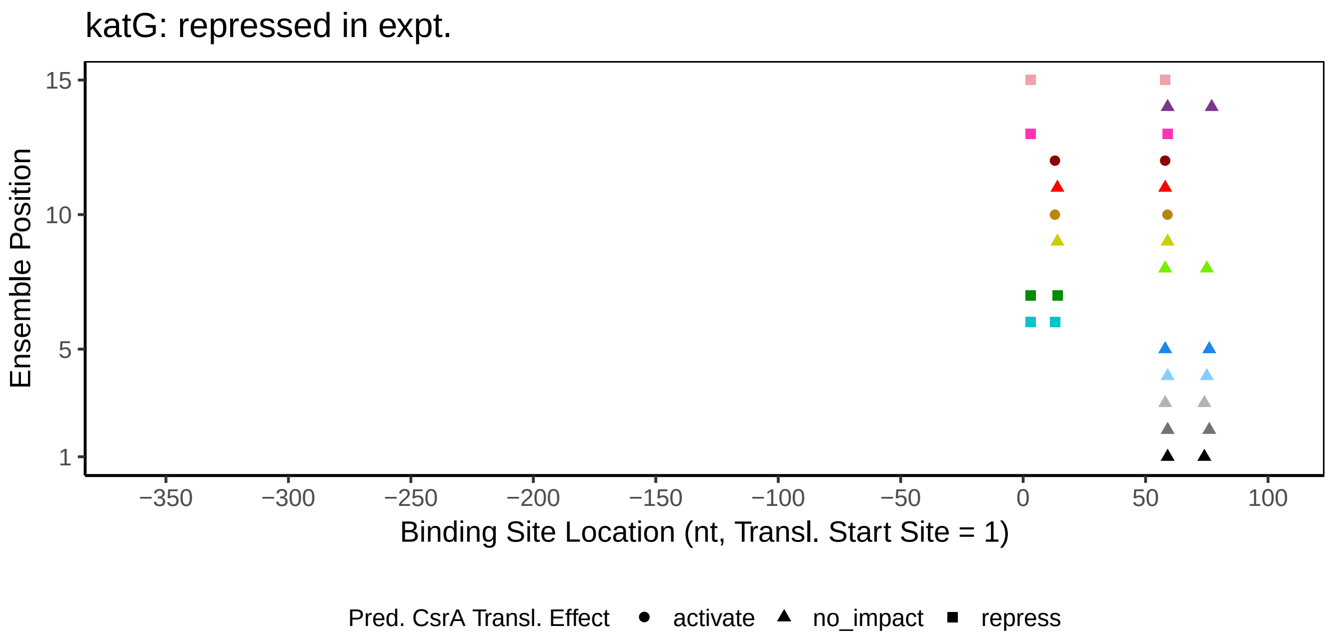
katG: repressed in expt.
Ensemble Position
15
10
5
1
−350
−300
−250
−200
−150
−100
−50
0
50
100
Binding Site Location (nt, Transl. Start Site = 1)
Pred. CsrA Transl. Effect
activate
no_impact
repress

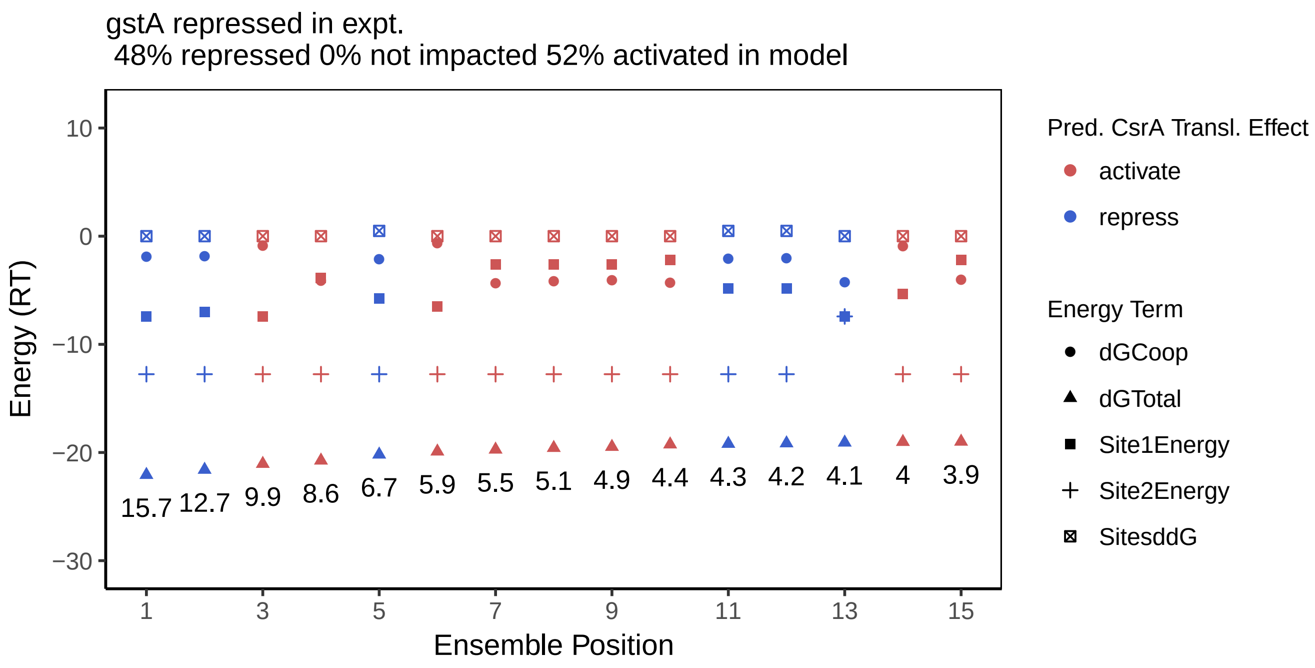
gstA repressed in expt.
48% repressed 0% not impacted 52% activated in model
Energy (RT)
10
0
−10
−20
−30
15.7 12.7 9.9 8.6 6.7 5.9 5.5 5.1 4.9 4.4 4.3 4.2 4.1 4 3.9
1 3 5 7 9 11 13 15
Ensemble Position
Pred. CsrA Transl. Effect
activate
repress
Energy Term
dGCoop
dGTotal
Site1Energy
Site2Energy
SitesddG

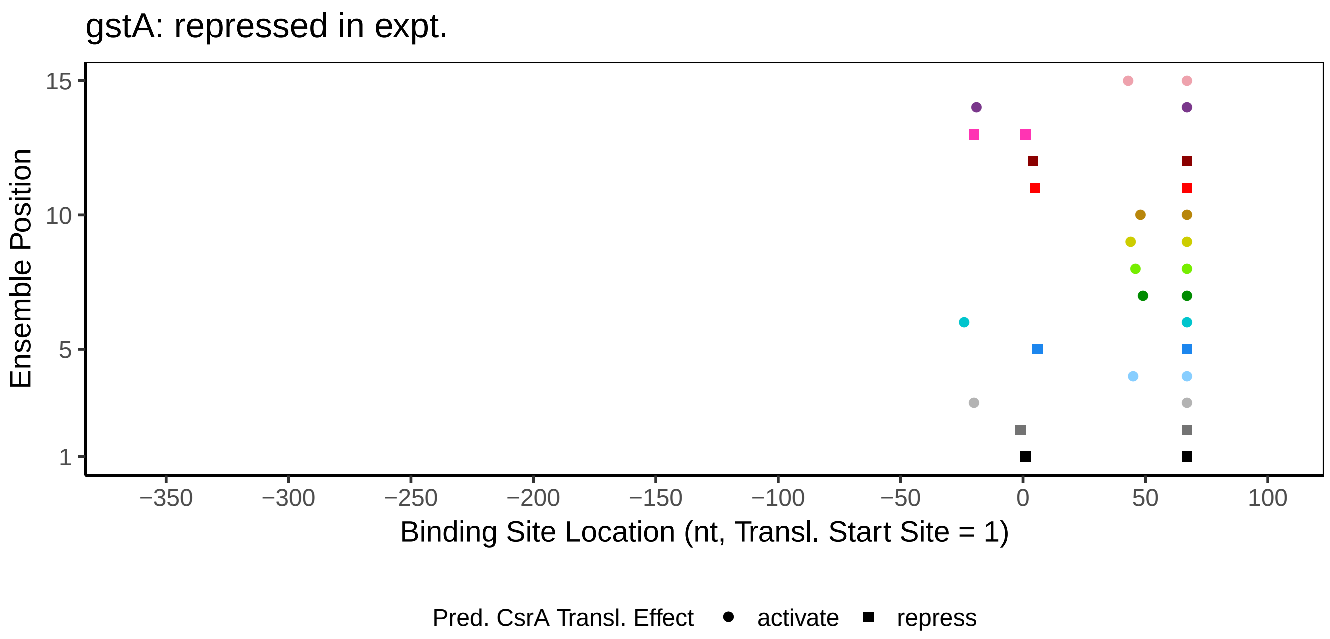
gstA: repressed in expt.
Ensemble Position
15
10
5
1
−350 −300 −250 −200 −150 −100 −50 0 50 100
Binding Site Location (nt, Transl. Start Site = 1)
Pred. CsrA Transl. Effect
activate
repress

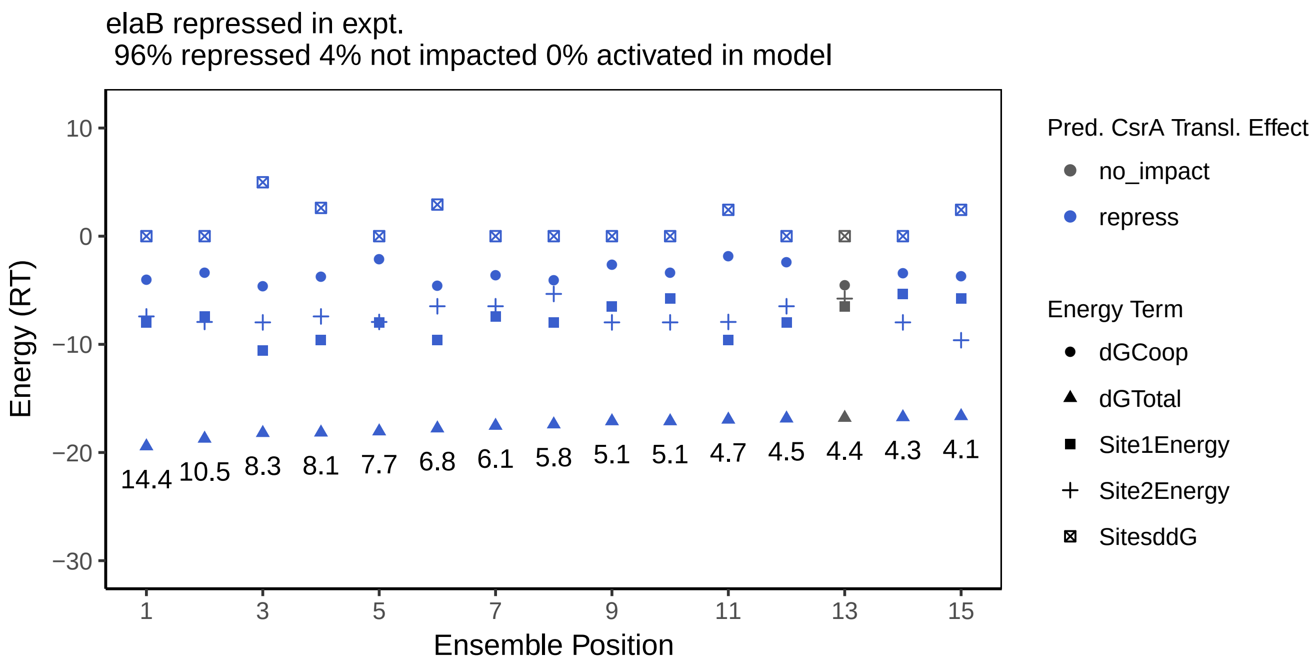
elaB repressed in expt.
96% repressed 4% not impacted 0% activated in model
Energy (RT)
10
0
−10
−20
−30
14.4 10.5 8.3 8.1 7.7 6.8 6.1 5.8 5.1 5.1 4.7 4.5 4.4 4.3 4.1
1
3
5
7
9
11
13
15
Ensemble Position
Pred. CsrA Transl. Effect
no_impact
repress
Energy Term
dGCoop
dGTotal
Site1Energy
Site2Energy
SitesddG

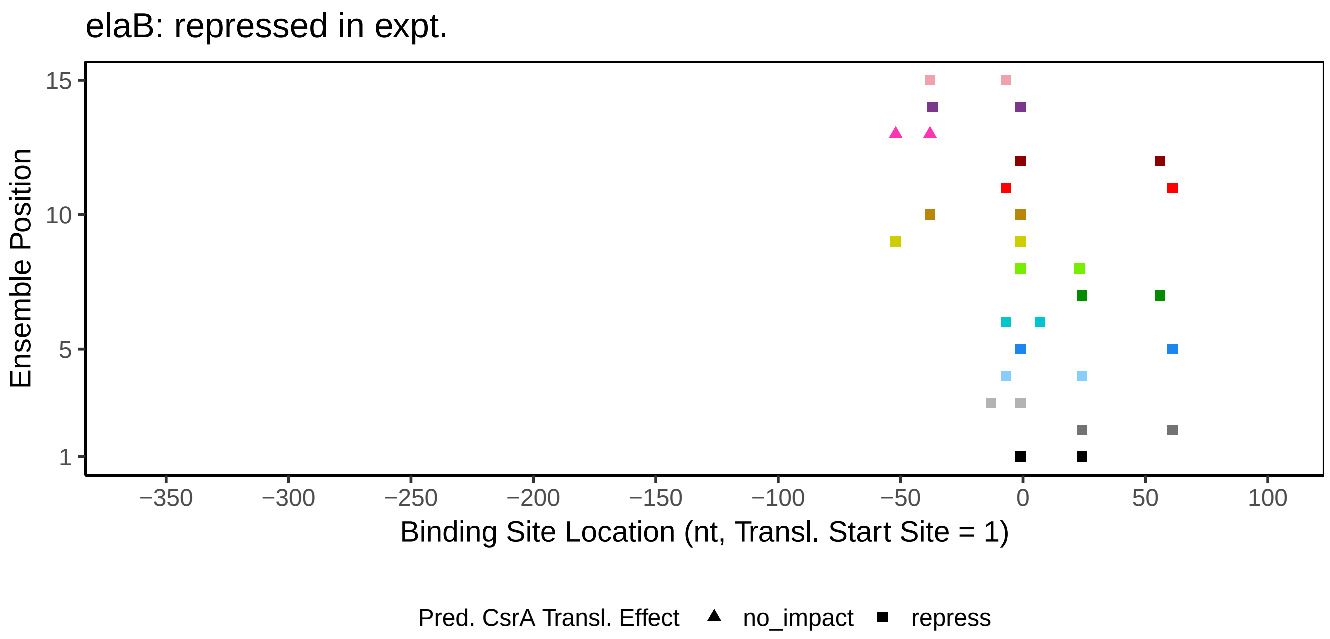
elaB: repressed in expt.
Ensemble Position
15
10
5
1
−350
−300
−250
−200
−150
−100
−50
0
50
100
Binding Site Location (nt, Transl. Start Site = 1)
Pred. CsrA Transl. Effect
no_impact
repress

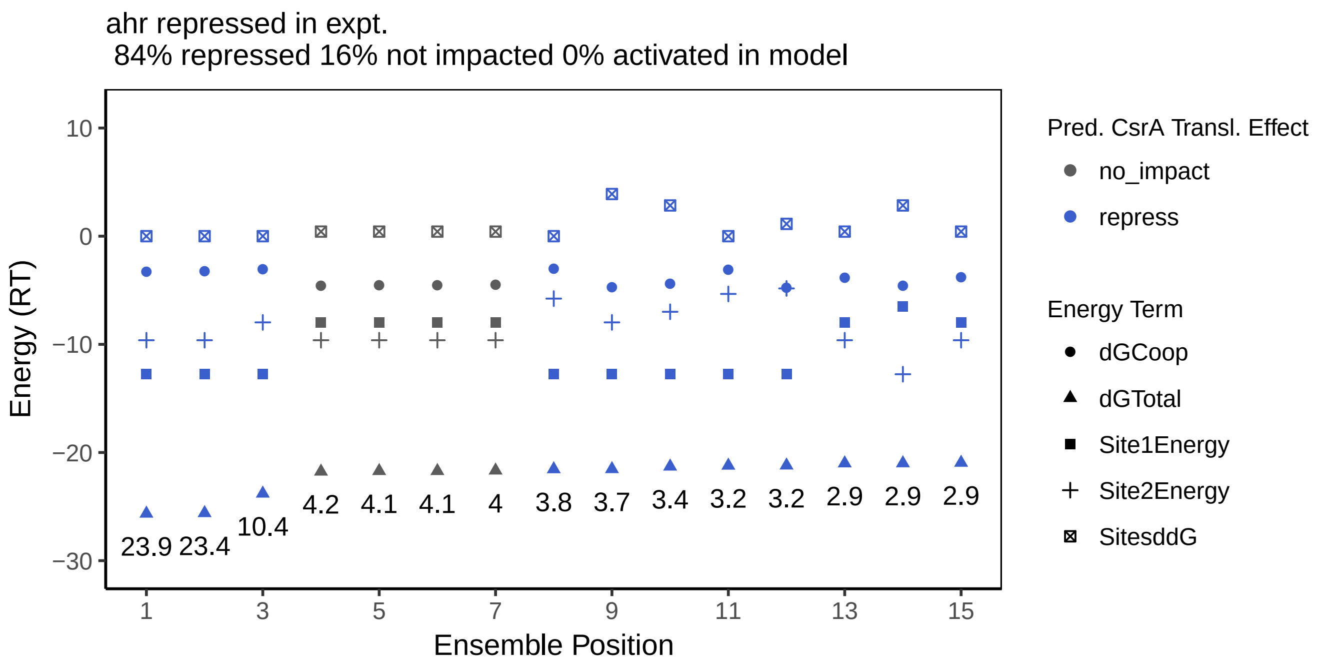

ahr repressed in expt.
84% repressed 16% not impacted 0% activated in model
Energy (RT)
Ensemble Position
23.9 23.4 10.4 4.2 4.1 4.1 4 3.8 3.7 3.4 3.2 3.2 2.9 2.9 2.9
Pred. CsrA Transl. Effect
no_impact
repress
Energy Term
dGCoop
dGTotal
Site1Energy
Site2Energy
SitesddG

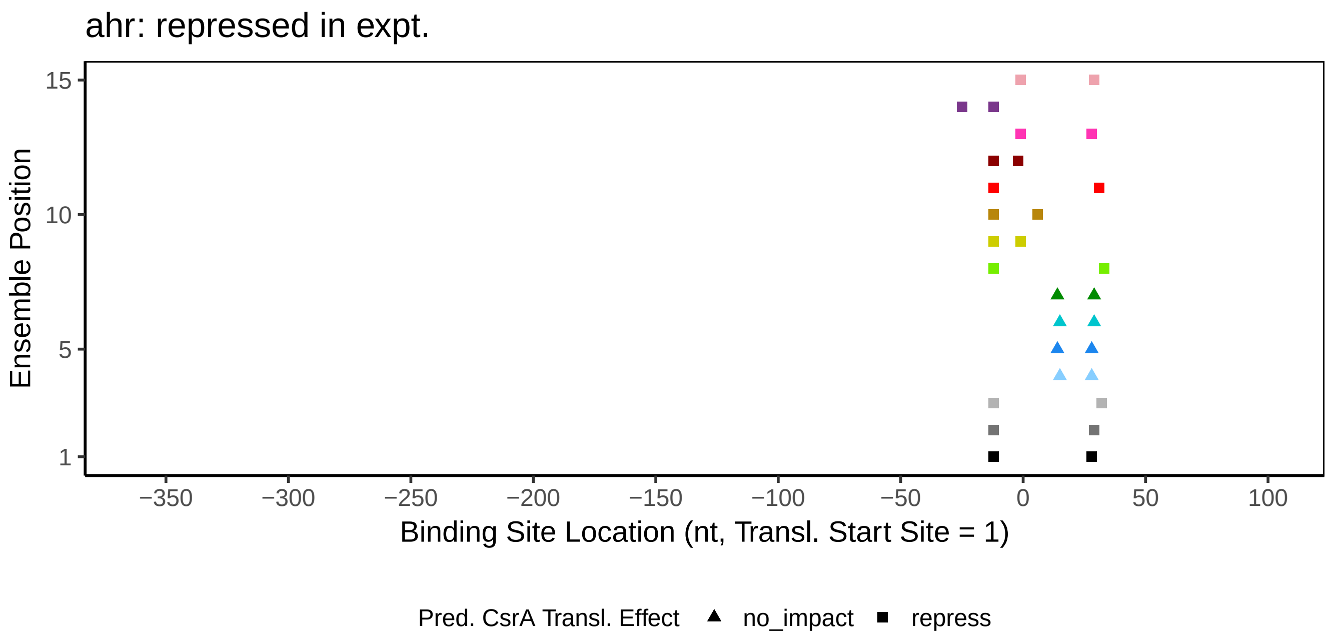

ahr: repressed in expt.
Ensemble Position
Binding Site Location (nt, Transl. Start Site = 1)
Pred. CsrA Transl. Effect
no_impact
repress

uspG repressed in expt.
100% repressed 0% not impacted 0% activated in model

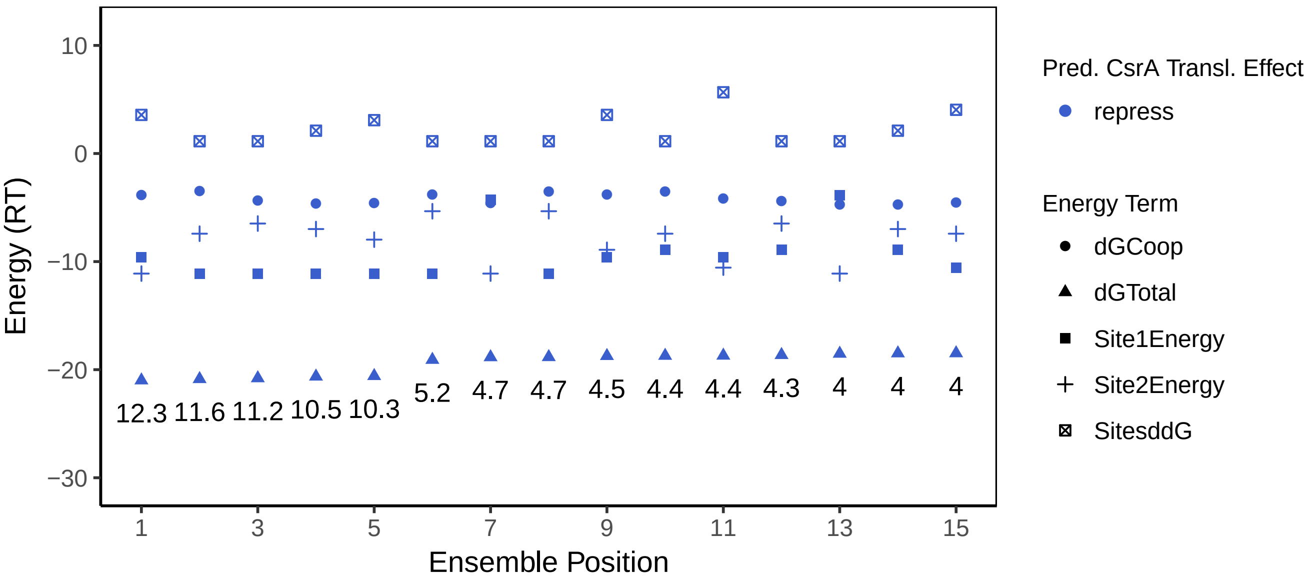

uspG: repressed in expt.

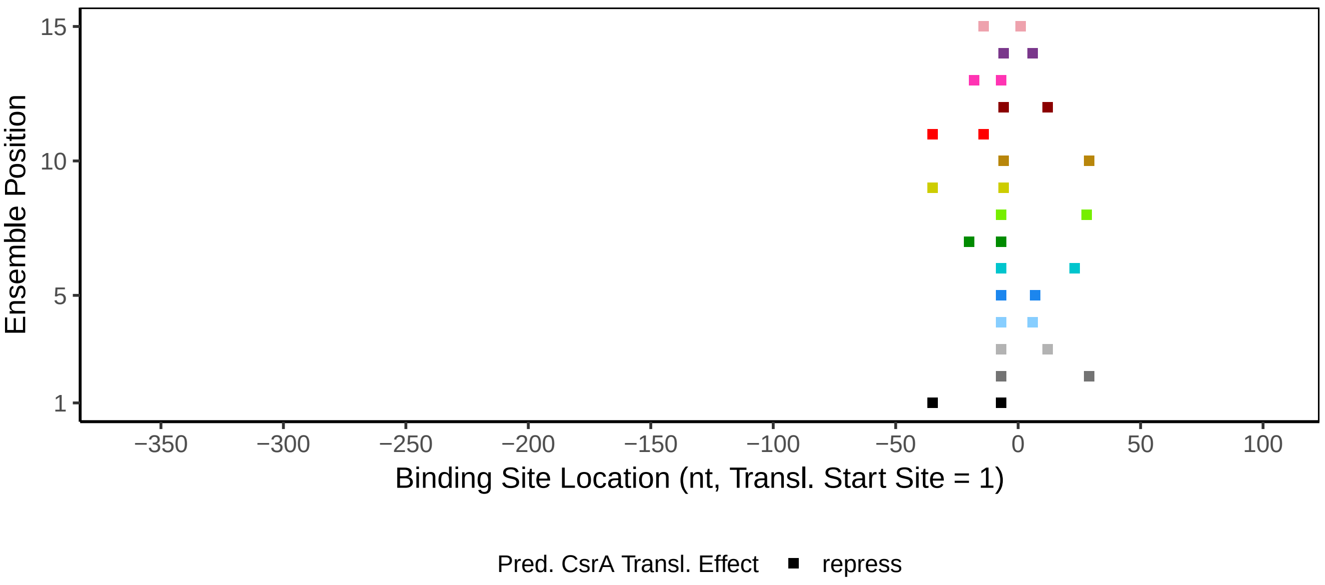

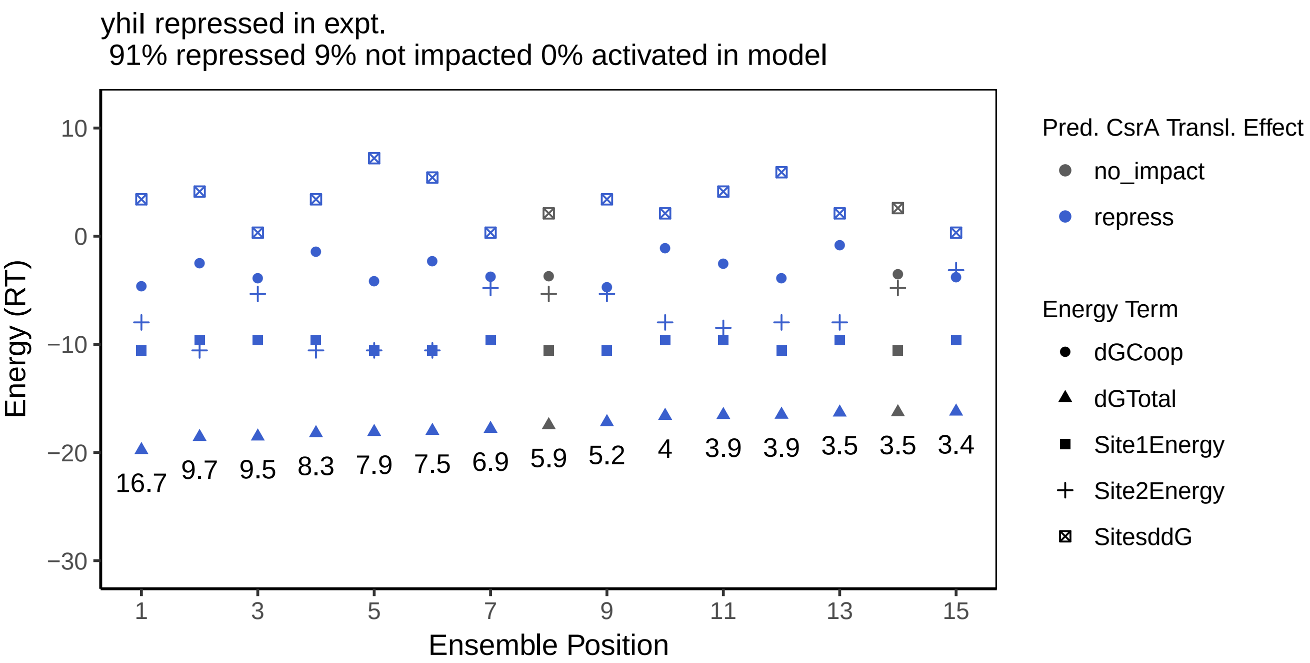
yhiI repressed in expt.
91% repressed 9% not impacted 0% activated in model
Energy (RT)
10
0
−10
−20
−30
16.7
9.7
9.5
8.3
7.9
7.5
6.9
5.9
5.2
4
3.9
3.9
3.5
3.5
3.4
1
3
5
7
9
11
13
15
Ensemble Position
Pred. CsrA Transl. Effect
no_impact
repress
Energy Term
dGCoop
dGTotal
Site1Energy
Site2Energy
SitesddG

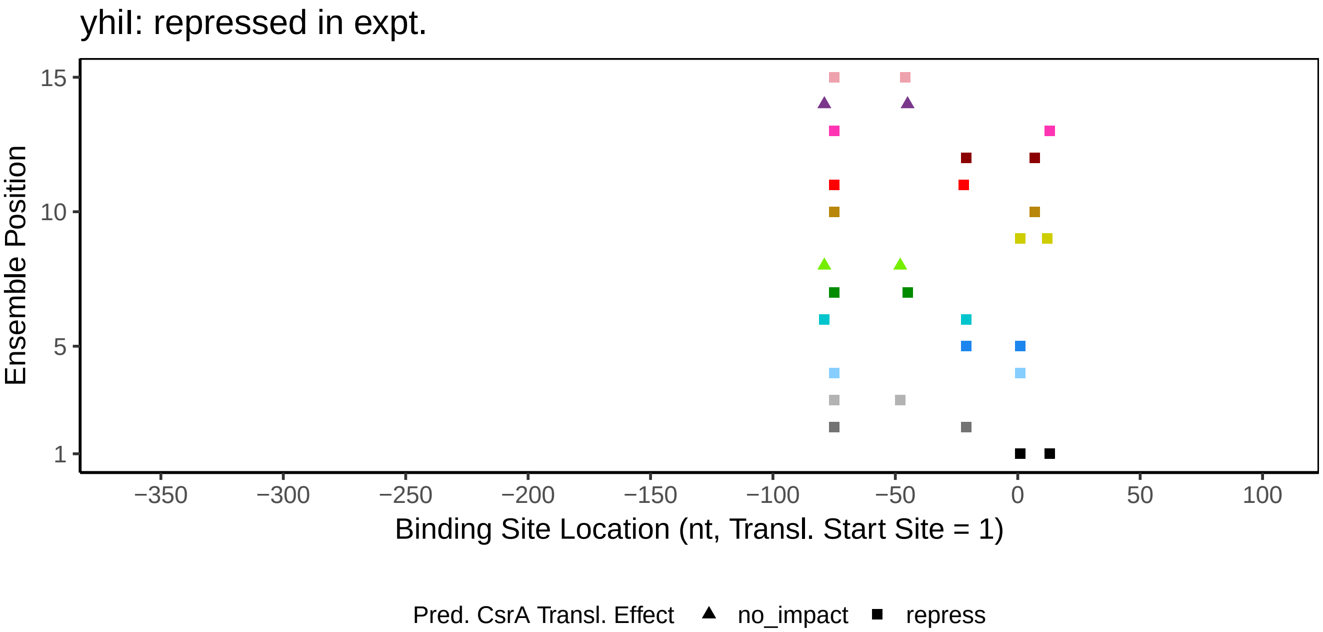
yhiI: repressed in expt.
Ensemble Position
15
10
5
1
−350
−300
−250
−200
−150
−100
−50
0
50
100
Binding Site Location (nt, Transl. Start Site = 1)
Pred. CsrA Transl. Effect
no_impact
repress

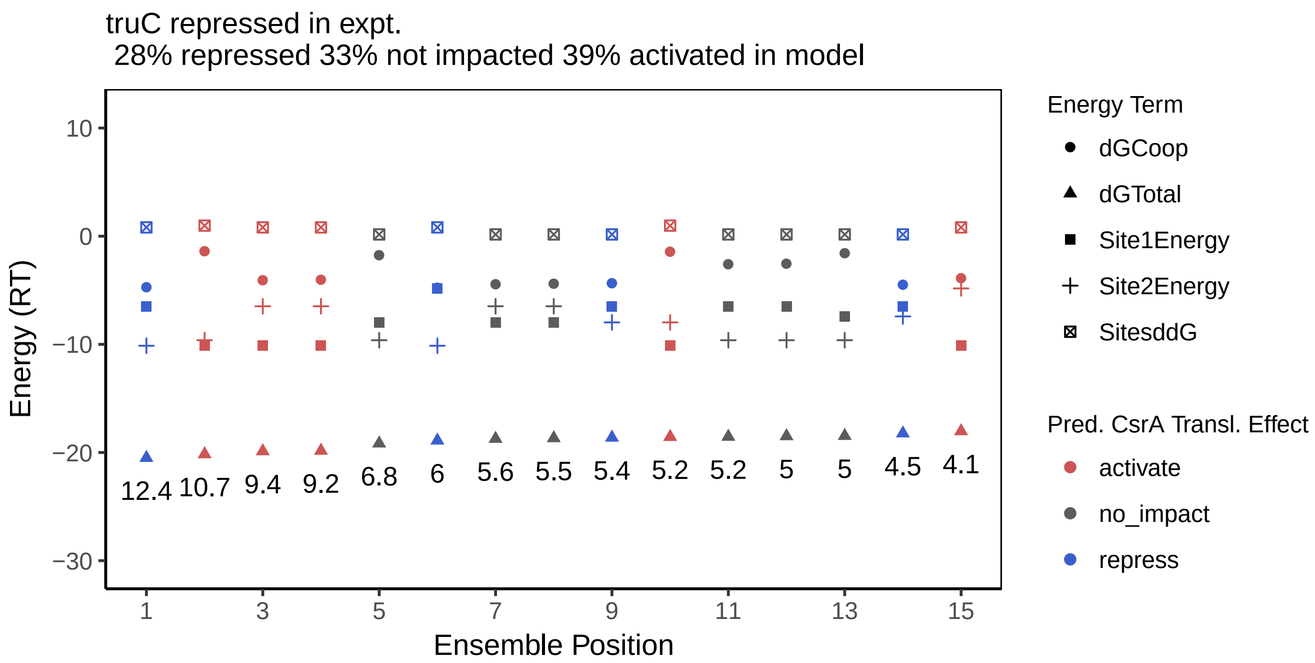
truC repressed in expt.
28% repressed 33% not impacted 39% activated in model
Energy (RT)
10
0
−10
−20
−30
12.4 10.7 9.4 9.2 6.8 6 5.6 5.5 5.4 5.2 5.2 5 5 4.5 4.1
1 3 5 7 9 11 13 15
Ensemble Position
Energy Term
dGCoop
dGTotal
Site1Energy
Site2Energy
SitesddG
Pred. CsrA Transl. Effect
activate
no_impact
repress

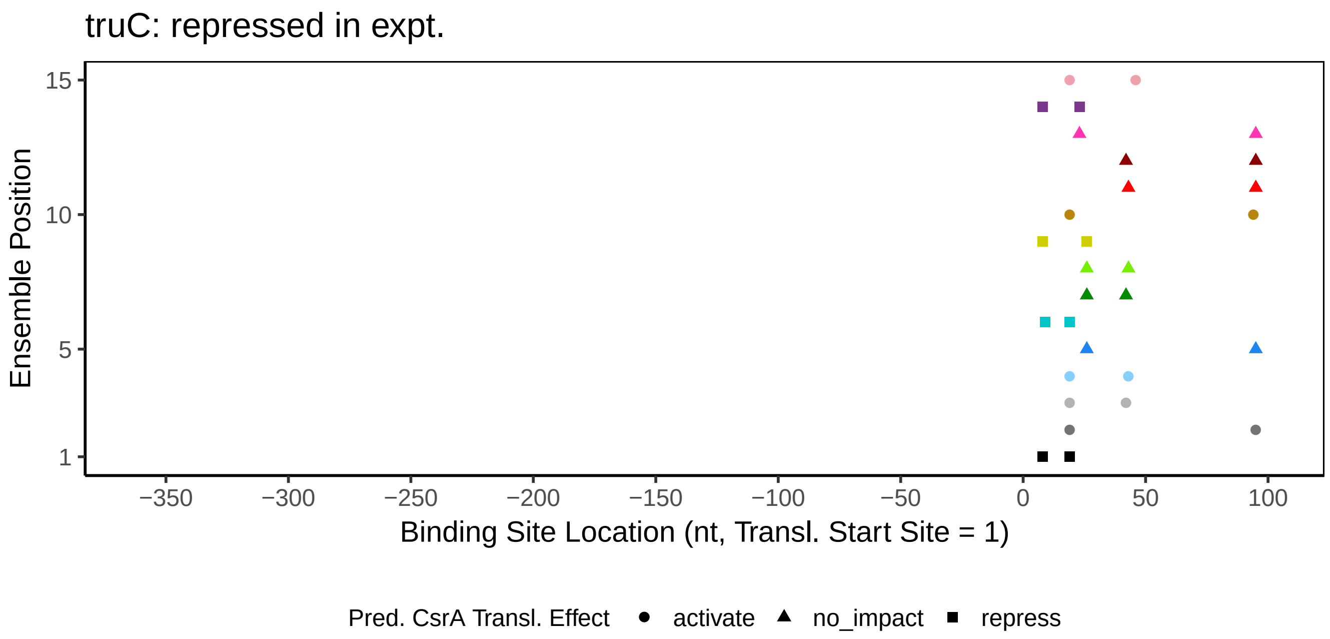
truC: repressed in expt.
Ensemble Position
15
10
5
1
−350 −300 −250 −200 −150 −100 −50 0 50 100
Binding Site Location (nt, Transl. Start Site = 1)
Pred. CsrA Transl. Effect
activate
no_impact
repress

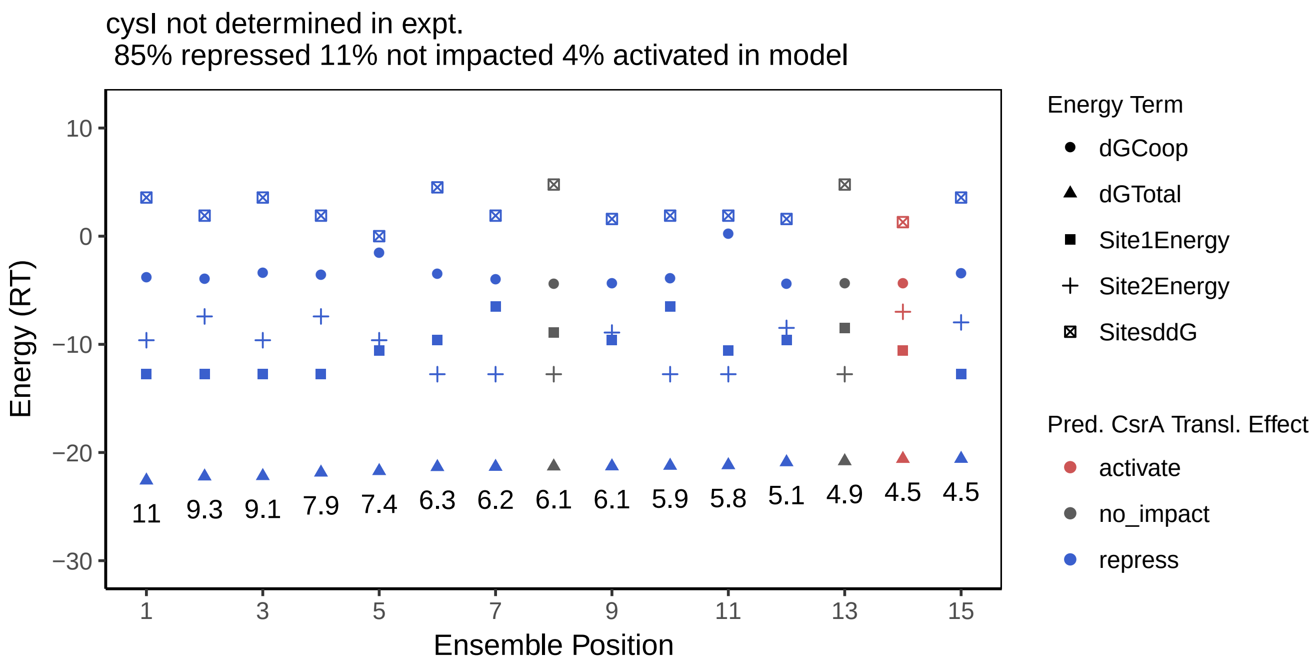
cysI not determined in expt.
85% repressed 11% not impacted 4% activated in model
Energy (RT)
10
0
−10
−20
−30
11
9.3
9.1
7.9
7.4
6.3
6.2
6.1
6.1
5.9
5.8
5.1
4.9
4.5
4.5
1
3
5
7
9
11
13
15
Ensemble Position
Energy Term
dGCoop
dGTotal
Site1Energy
Site2Energy
SitesddG
Pred. CsrA Transl. Effect
activate
no_impact
repress

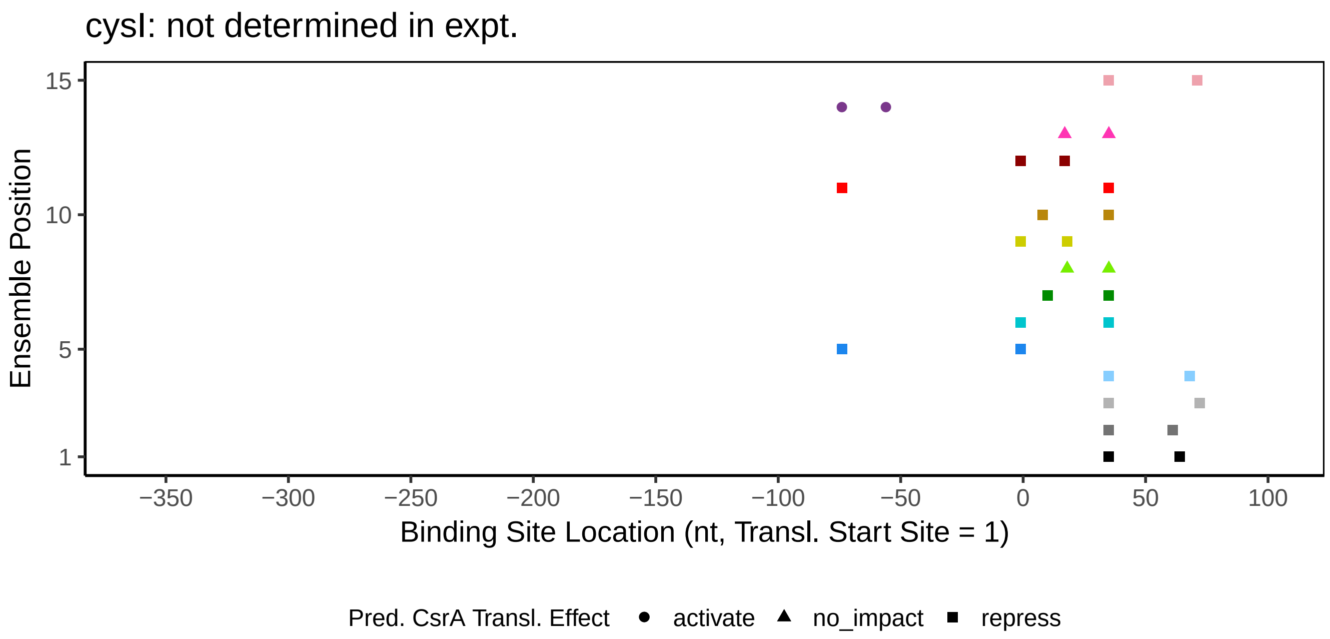
cysI: not determined in expt.
Ensemble Position
15
10
5
1
−350
−300
−250
−200
−150
−100
−50
0
50
100
Binding Site Location (nt, Transl. Start Site = 1)
Pred. CsrA Transl. Effect
activate
no_impact
repress

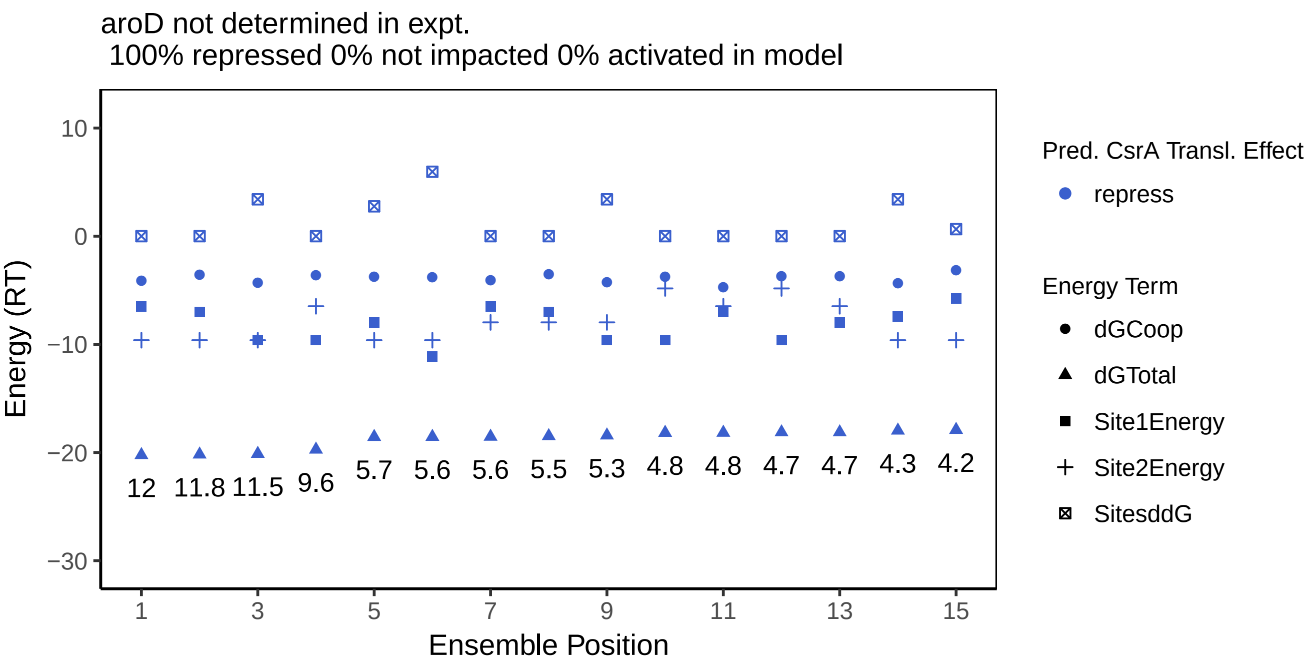
aroD not determined in expt.
100% repressed 0% not impacted 0% activated in model
Energy (RT)
10
0
−10
−20
−30
12
11.8
11.5
9.6
5.7
5.6
5.6
5.5
5.3
4.8
4.8
4.7
4.7
4.3
4.2
1
3
5
7
9
11
13
15
Ensemble Position
Pred. CsrA Transl. Effect
repress
Energy Term
dGCoop
dGTotal
Site1Energy
Site2Energy
SitesddG

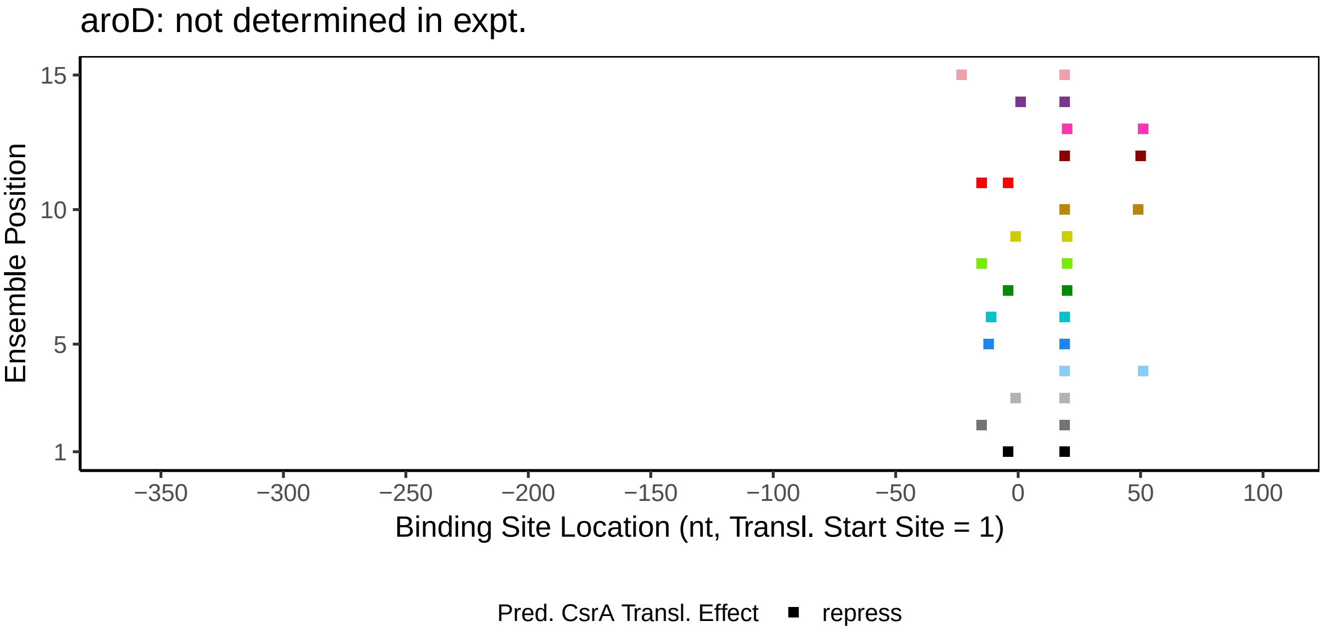
aroD: not determined in expt.
Ensemble Position
15
10
5
1
−350
−300
−250
−200
−150
−100
−50
0
50
100
Binding Site Location (nt, Transl. Start Site = 1)
Pred. CsrA Transl. Effect
repress

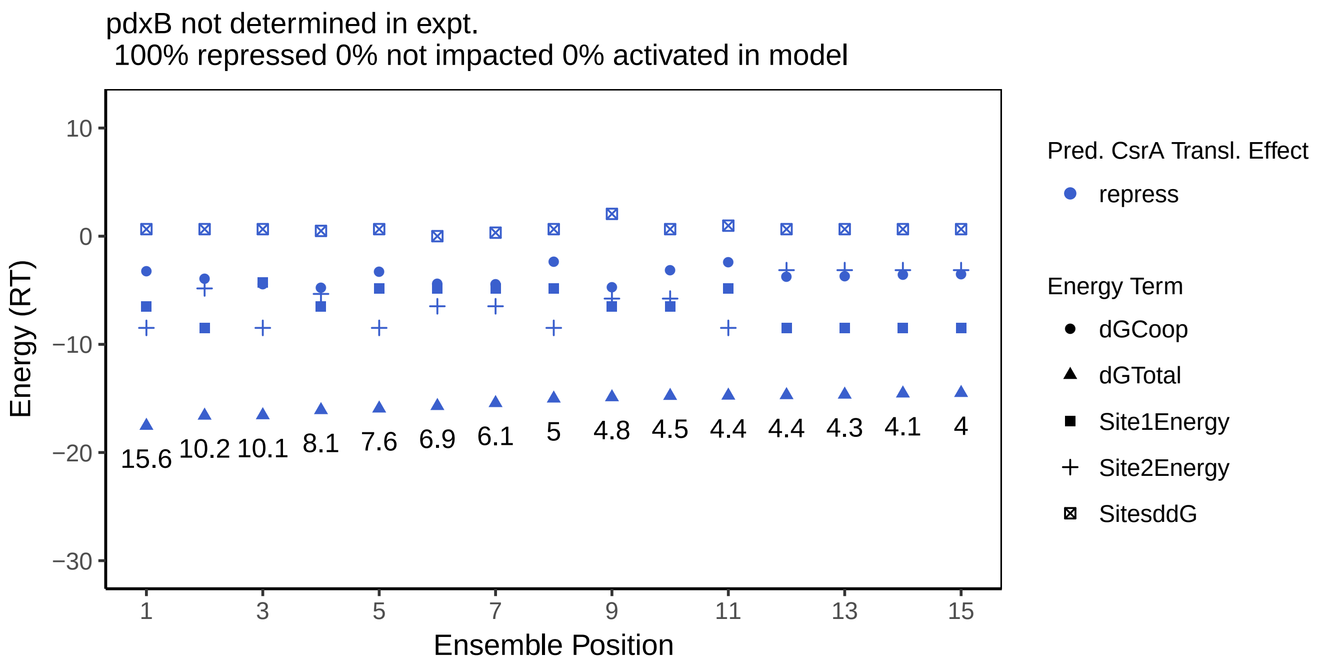

pdxB not determined in expt.
100% repressed 0% not impacted 0% activated in model
Energy (RT)
Ensemble Position
15.6
10.2
10.1
8.1
7.6
6.9
6.1
5
4.8
4.5
4.4
4.4
4.3
4.1
4
Pred. CsrA Transl. Effect
repress
Energy Term
dGCoop
dGTotal
Site1Energy
Site2Energy
SitesddG

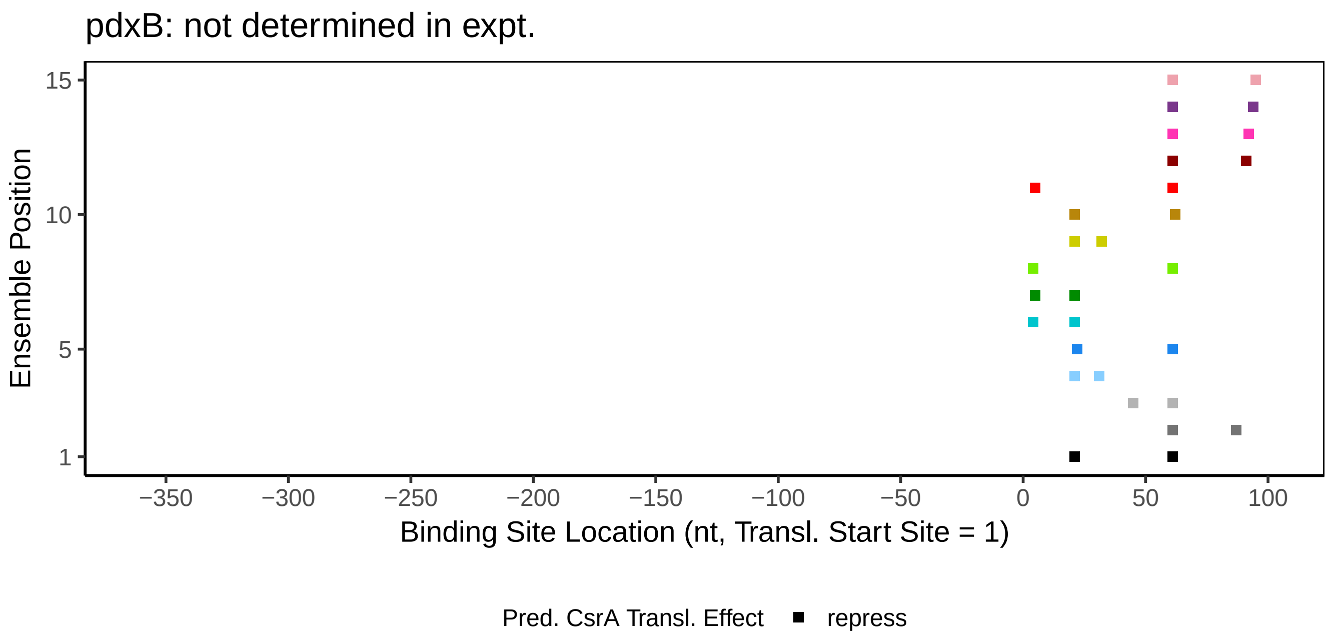

pdxB: not determined in expt.
Ensemble Position
Binding Site Location (nt, Transl. Start Site = 1)
Pred. CsrA Transl. Effect
repress

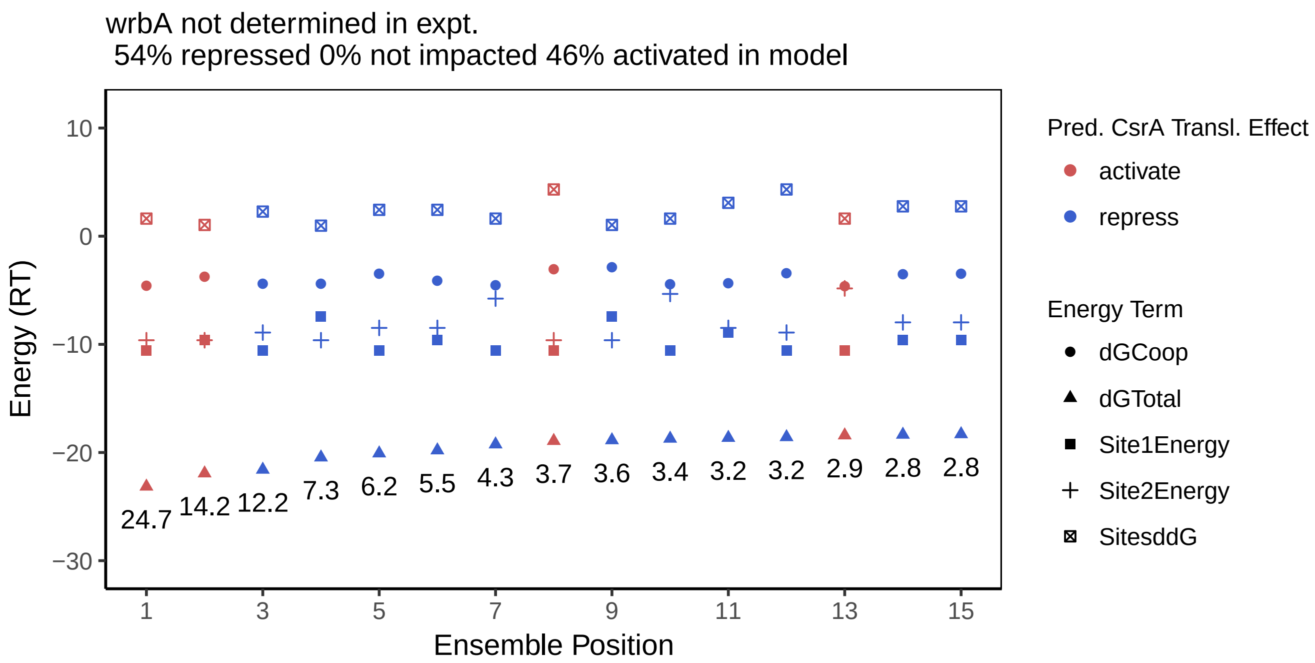

wrbA not determined in expt.
54% repressed 0% not impacted 46% activated in model
Energy (RT)
Ensemble Position
24.7 14.2 12.2 7.3 6.2 5.5 4.3 3.7 3.6 3.4 3.2 3.2 2.9 2.8 2.8
Pred. CsrA Transl. Effect
activate
repress
Energy Term
dGCoop
dGTotal
Site1Energy
Site2Energy
SitesddG

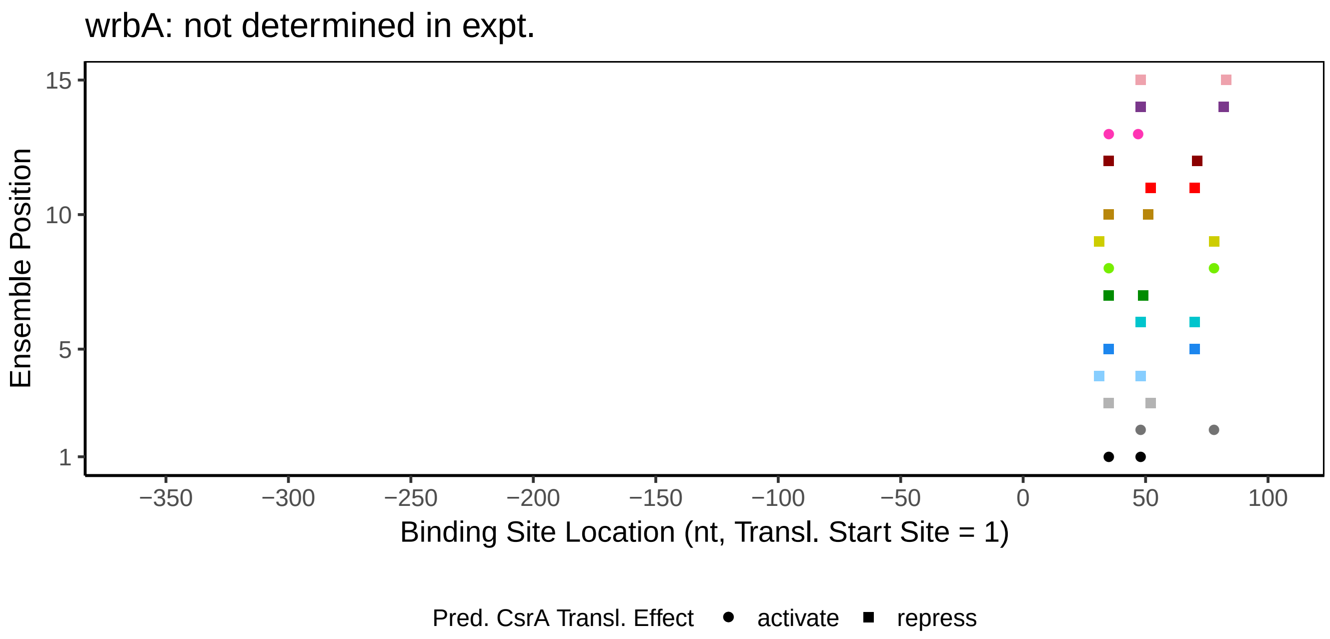

wrbA: not determined in expt.
Ensemble Position
Binding Site Location (nt, Transl. Start Site = 1)
Pred. CsrA Transl. Effect
activate
repress

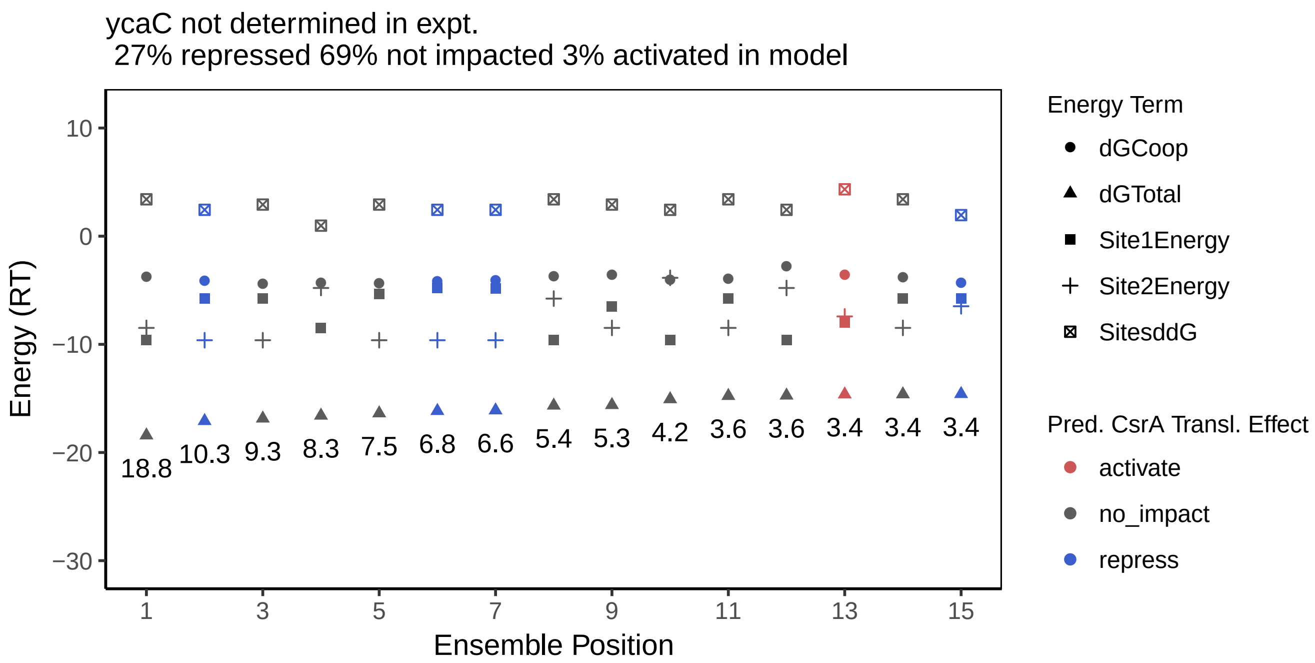
ycaC not determined in expt.
27% repressed 69% not impacted 3% activated in model
Energy (RT)
10
0
−10
−20
−30
18.8 10.3 9.3 8.3 7.5 6.8 6.6 5.4 5.3 4.2 3.6 3.6 3.4 3.4 3.4
1 3 5 7 9 11 13 15
Ensemble Position
Energy Term
dGCoop
dGTotal
Site1Energy
Site2Energy
SitesddG
Pred. CsrA Transl. Effect
activate
no_impact
repress

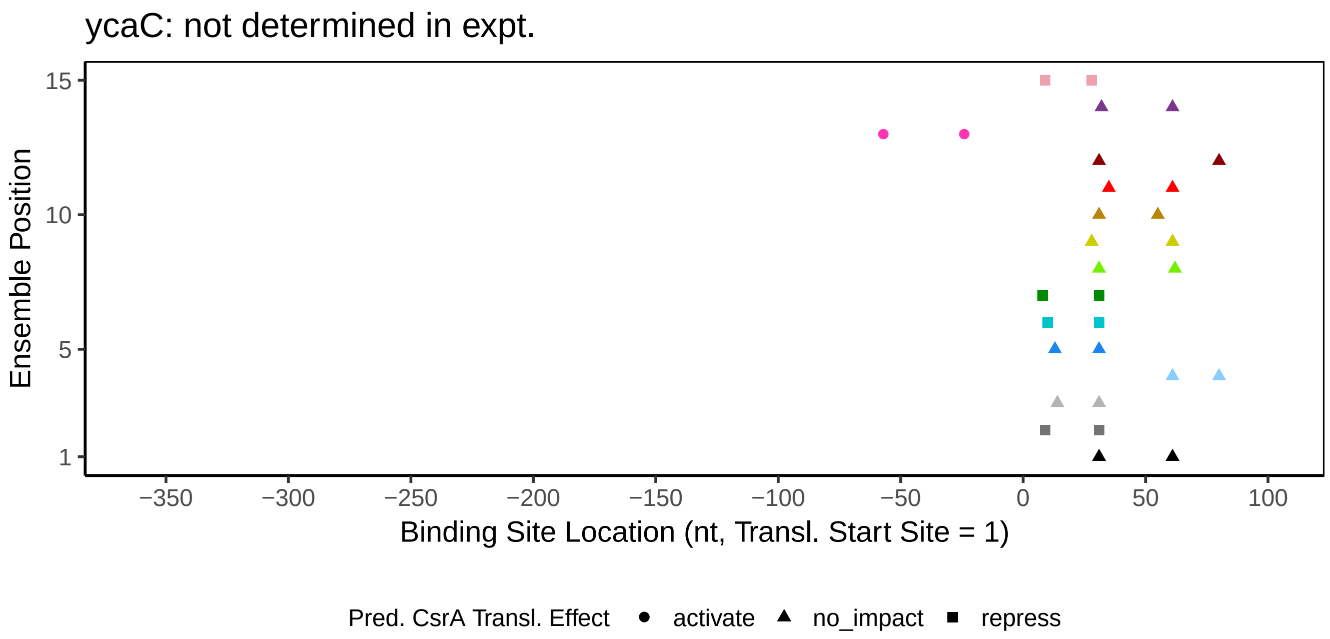
ycaC: not determined in expt.
Ensemble Position
15
10
5
1
−350 −300 −250 −200 −150 −100 −50 0 50 100
Binding Site Location (nt, Transl. Start Site = 1)
Pred. CsrA Transl. Effect
activate
no_impact
repress

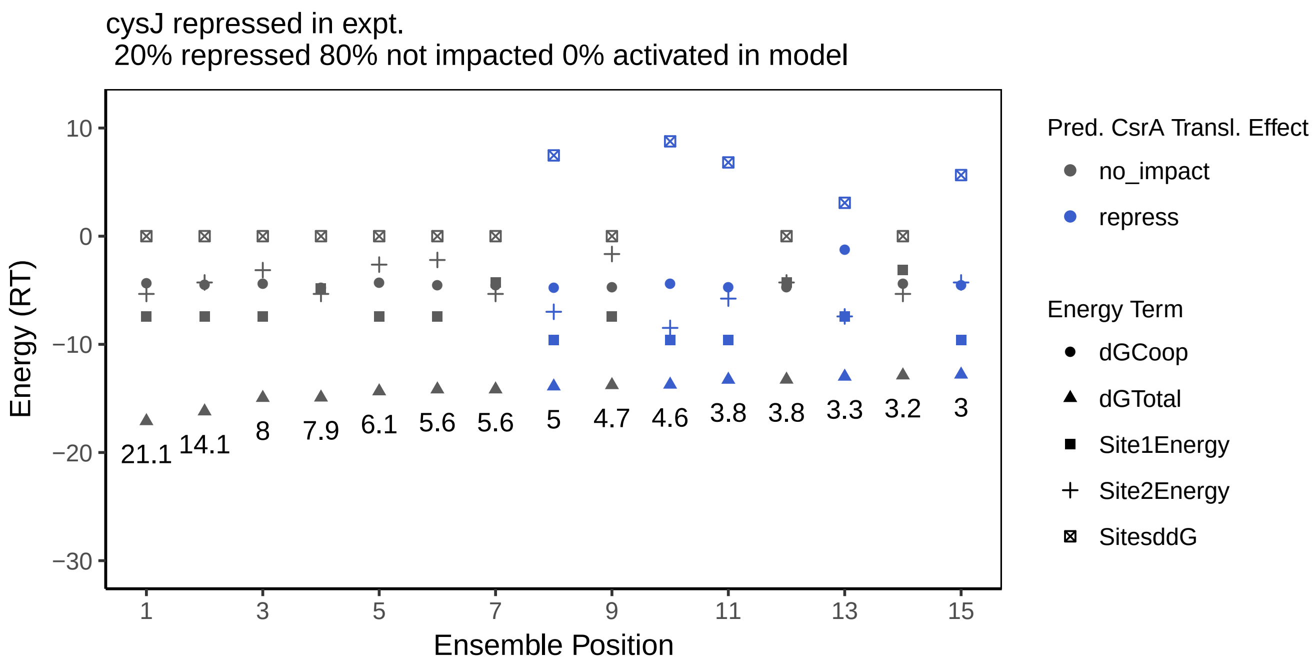
cysJ repressed in expt.
20% repressed 80% not impacted 0% activated in model
Energy (RT)
10
0
−10
−20
−30
21.1 14.1 8 7.9 6.1 5.6 5.6 5 4.7 4.6 3.8 3.8 3.3 3.2 3
1 3 5 7 9 11 13 15
Ensemble Position
Pred. CsrA Transl. Effect
no_impact
repress
Energy Term
dGCoop
dGTotal
Site1Energy
Site2Energy
SitesddG

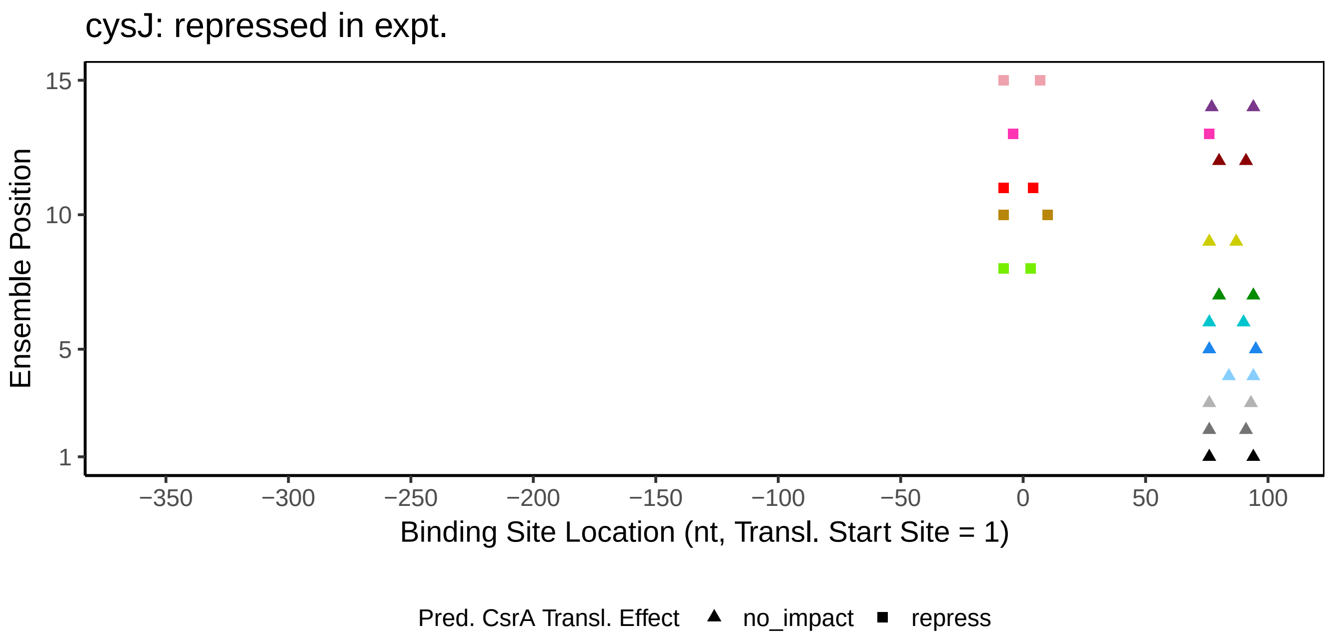
cysJ: repressed in expt.
Ensemble Position
15
10
5
1
−350 −300 −250 −200 −150 −100 −50 0 50 100
Binding Site Location (nt, Transl. Start Site = 1)
Pred. CsrA Transl. Effect
no_impact
repress

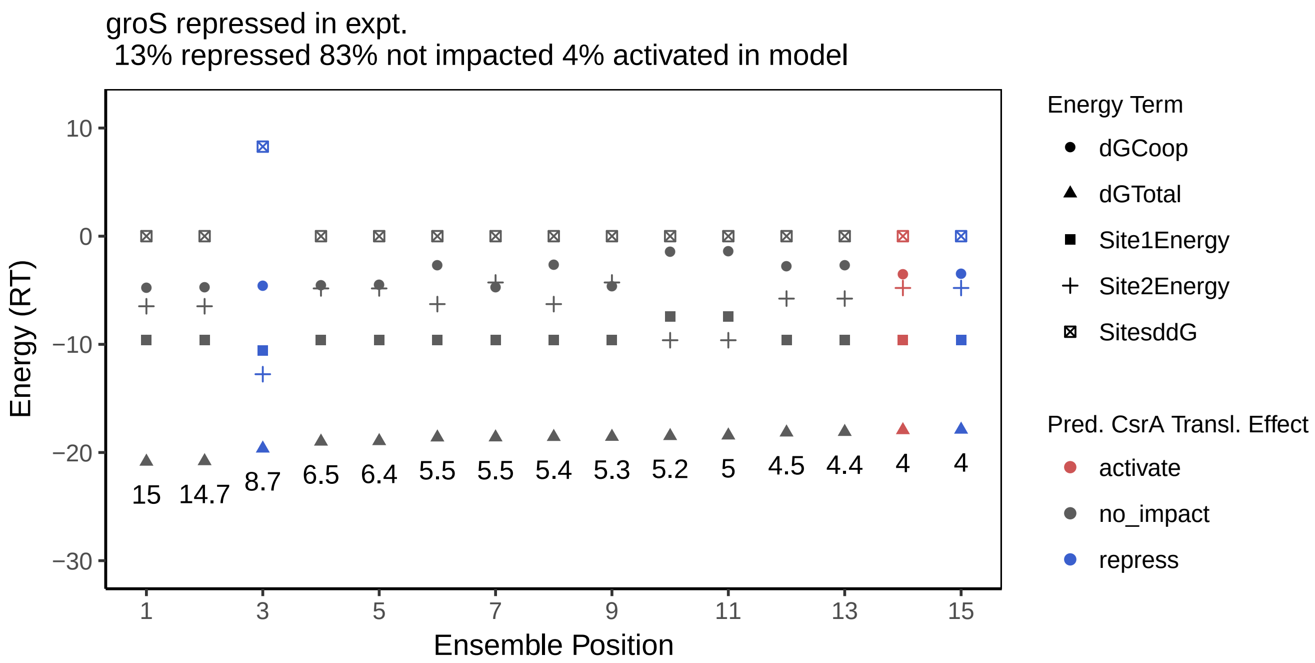
groS repressed in expt.
13% repressed 83% not impacted 4% activated in model
Energy (RT)
10
0
−10
−20
−30
15
14.7
8.7
6.5
6.4
5.5
5.5
5.4
5.3
5.2
5
4.5
4.4
4
4
1
3
5
7
9
11
13
15
Ensemble Position
Energy Term
dGCoop
dGTotal
Site1Energy
Site2Energy
SitesddG
Pred. CsrA Transl. Effect
activate
no_impact
repress

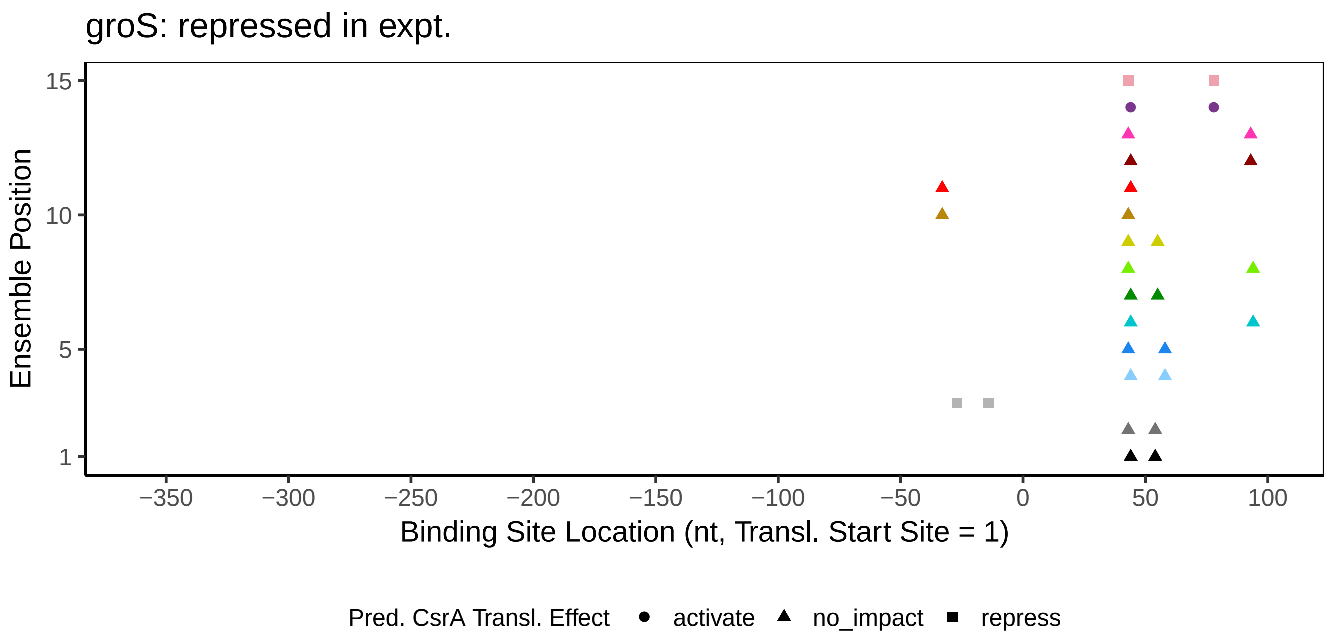
groS: repressed in expt.
Ensemble Position
15
10
5
1
−350
−300
−250
−200
−150
−100
−50
0
50
100
Binding Site Location (nt, Transl. Start Site = 1)
Pred. CsrA Transl. Effect
activate
no_impact
repress

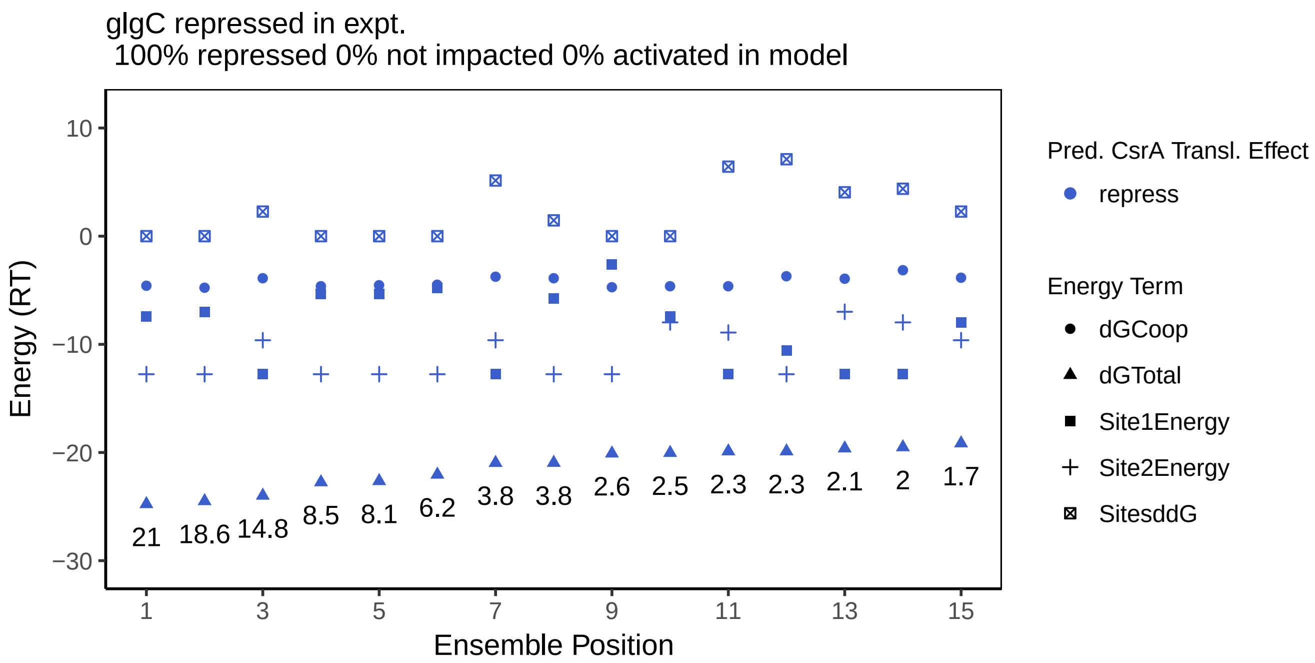
glgC repressed in expt.
100% repressed 0% not impacted 0% activated in model
Energy (RT)
10
0
−10
−20
−30
21
18.6
14.8
8.5
8.1
6.2
3.8
3.8
2.6
2.5
2.3
2.3
2.1
2
1.7
1
3
5
7
9
11
13
15
Ensemble Position
Pred. CsrA Transl. Effect
repress
Energy Term
dGCoop
dGTotal
Site1Energy
Site2Energy
SitesddG

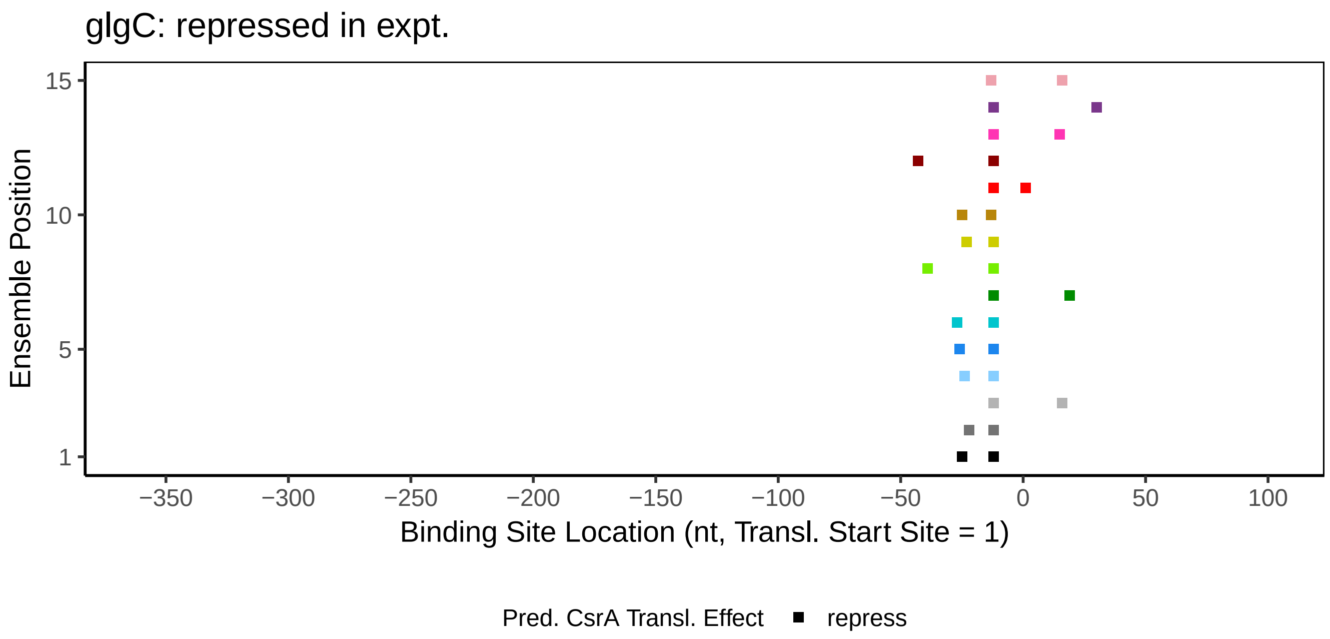
glgC: repressed in expt.
Ensemble Position
15
10
5
1
−350
−300
−250
−200
−150
−100
−50
0
50
100
Binding Site Location (nt, Transl. Start Site = 1)
Pred. CsrA Transl. Effect
repress

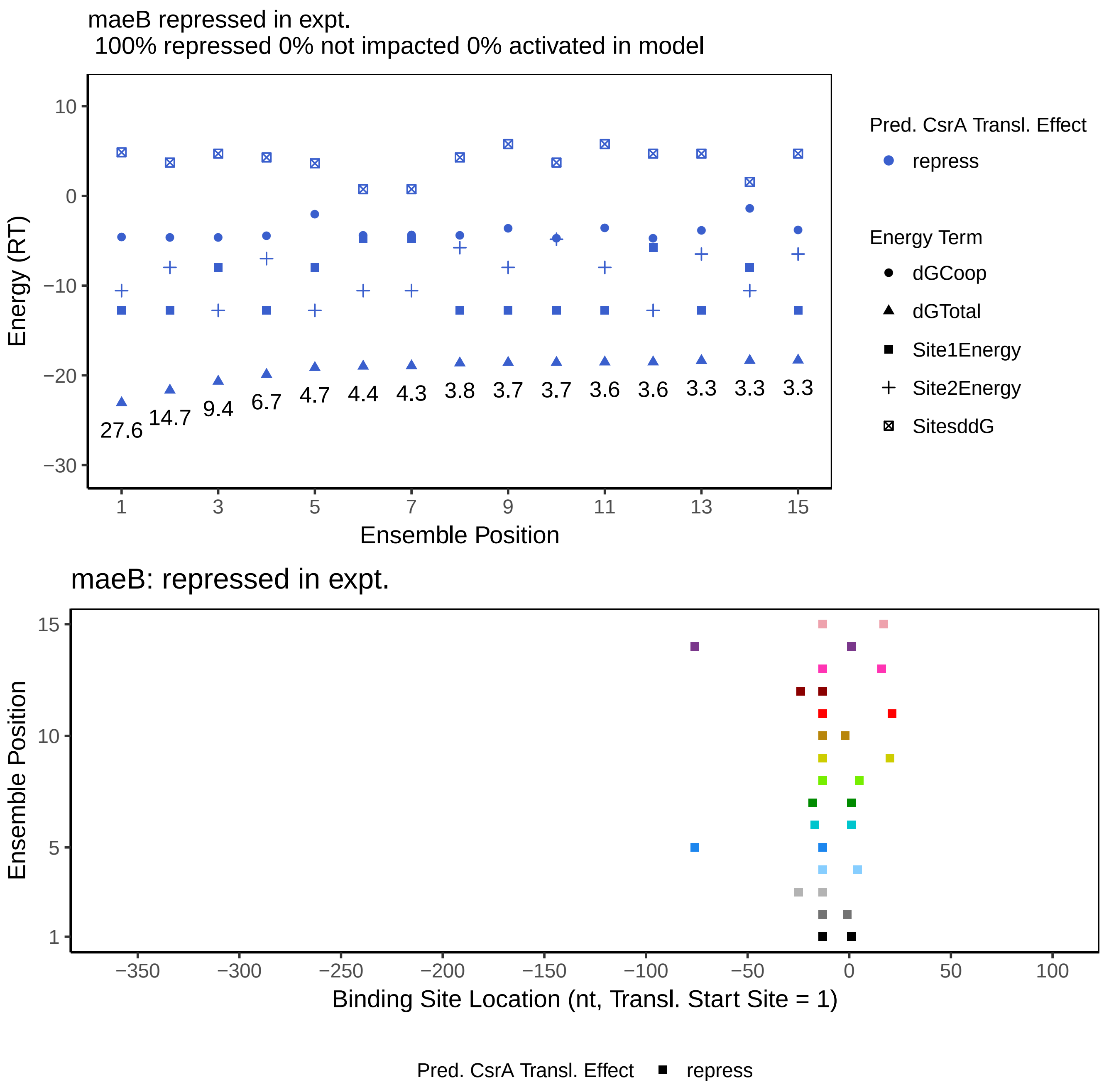

maeB repressed in expt.
100% repressed 0% not impacted 0% activated in model
Energy (RT)
10
0
−10
−20
−30
27.6
14.7
9.4
6.7
4.7
4.4
4.3
3.8
3.7
3.7
3.6
3.6
3.3
3.3
3.3
1
3
5
7
9
11
13
15
Ensemble Position
Pred. CsrA Transl. Effect
repress
Energy Term
dGCoop
dGTotal
Site1Energy
Site2Energy
SitesddG
maeB: repressed in expt.
Ensemble Position
15
10
5
1
−350
−300
−250
−200
−150
−100
−50
0
50
100
Binding Site Location (nt, Transl. Start Site = 1)
Pred. CsrA Transl. Effect
repress

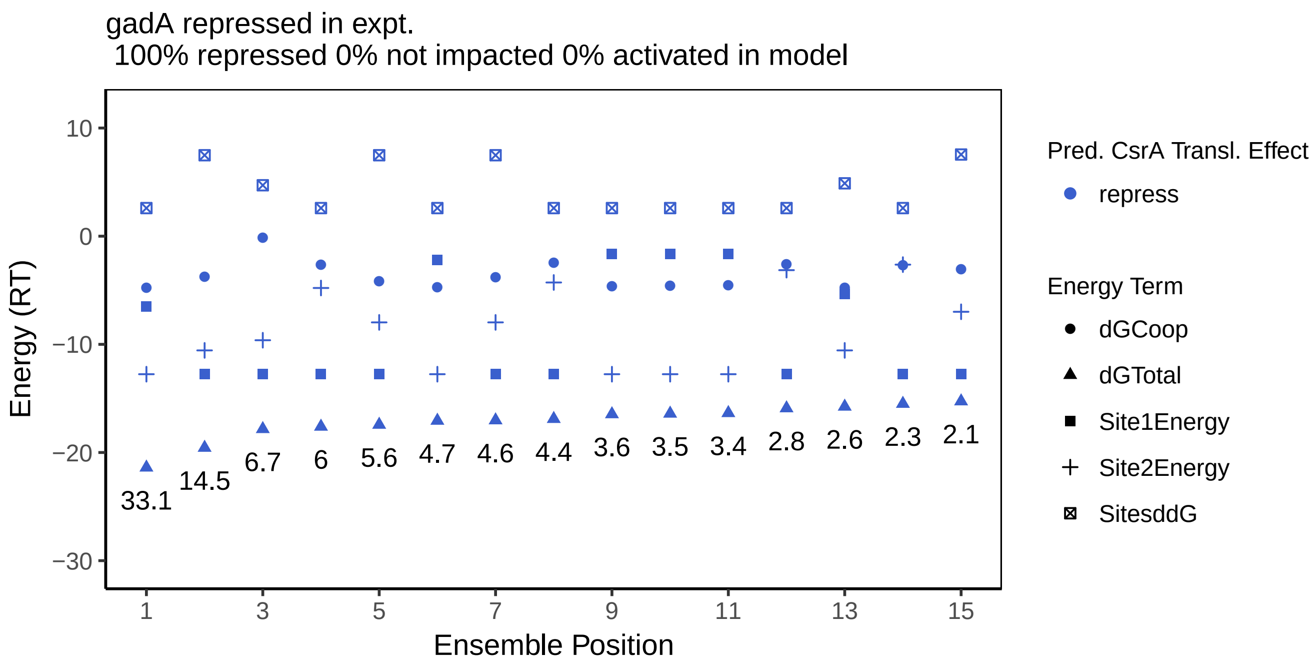
gadA repressed in expt.
100% repressed 0% not impacted 0% activated in model
Energy (RT)
Ensemble Position
33.1
14.5
6.7
6
5.6
4.7
4.6
4.4
3.6
3.5
3.4
2.8
2.6
2.3
2.1
Pred. CsrA Transl. Effect
repress
Energy Term
dGCoop
dGTotal
Site1Energy
Site2Energy
SitesddG

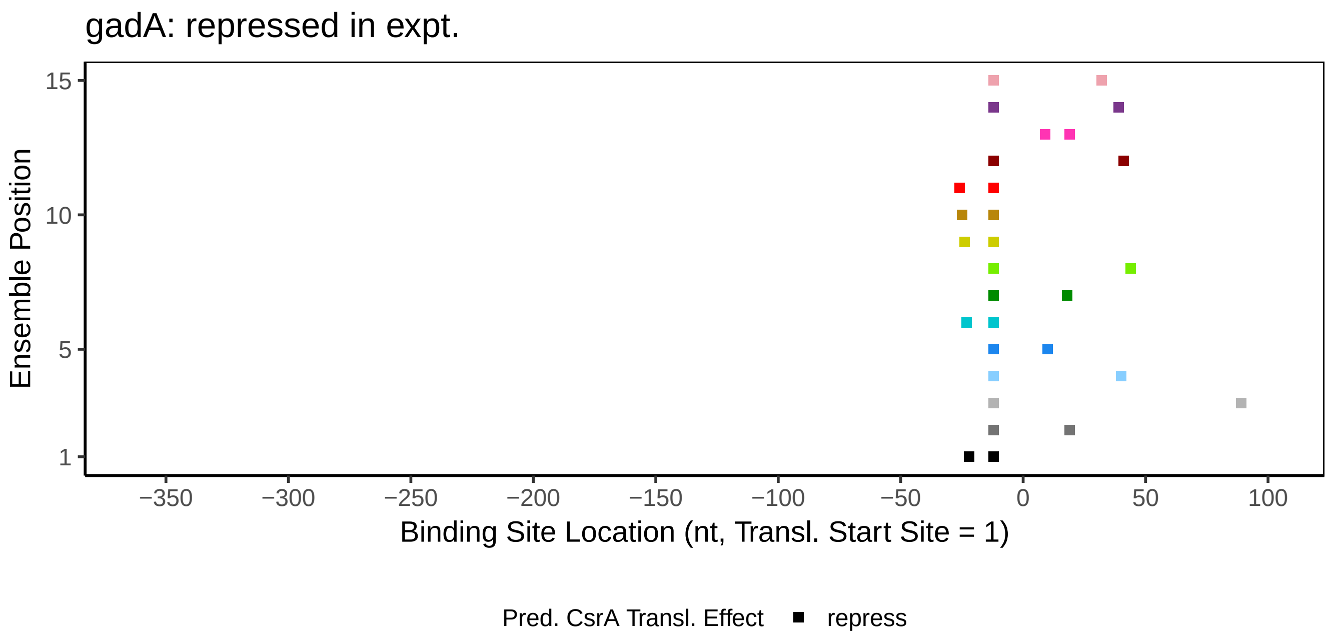
gadA: repressed in expt.
Ensemble Position
Binding Site Location (nt, Transl. Start Site = 1)
Pred. CsrA Transl. Effect
repress

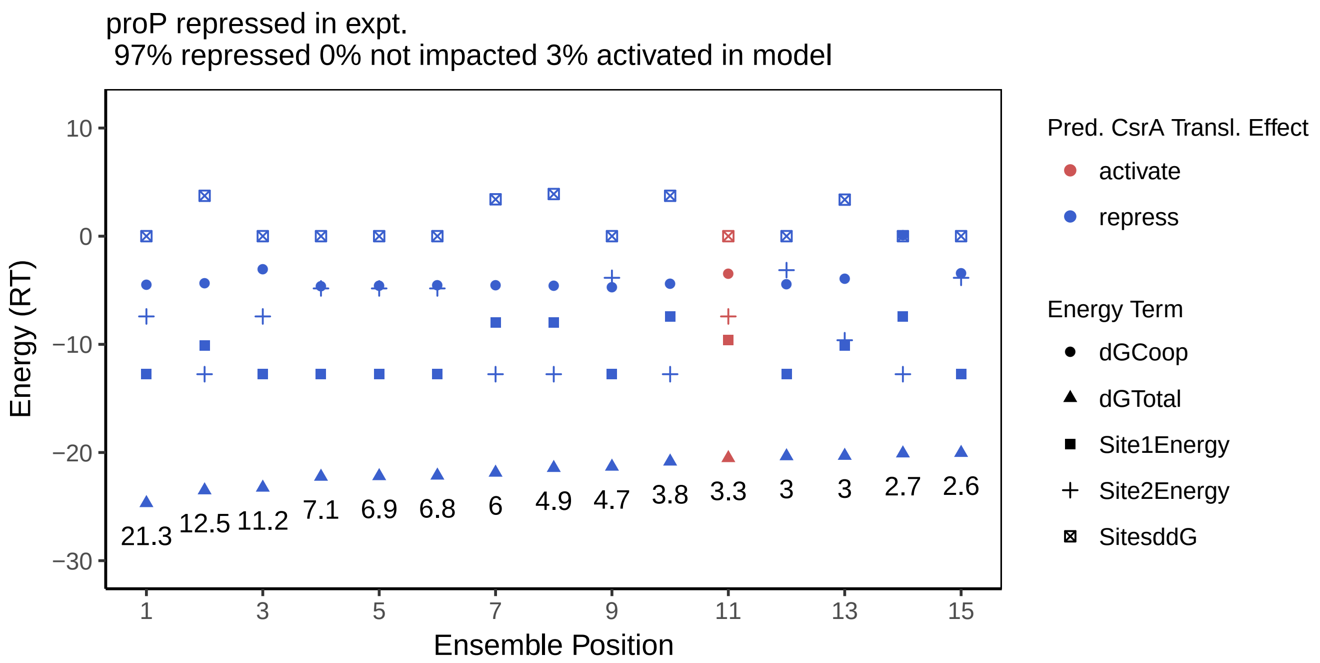

proP repressed in expt.
97% repressed 0% not impacted 3% activated in model
Energy (RT)
Ensemble Position
21.3
12.5
11.2
7.1
6.9
6.8
6
4.9
4.7
3.8
3.3
3
3
2.7
2.6
Pred. CsrA Transl. Effect
activate
repress
Energy Term
dGCoop
dGTotal
Site1Energy
Site2Energy
SitesddG

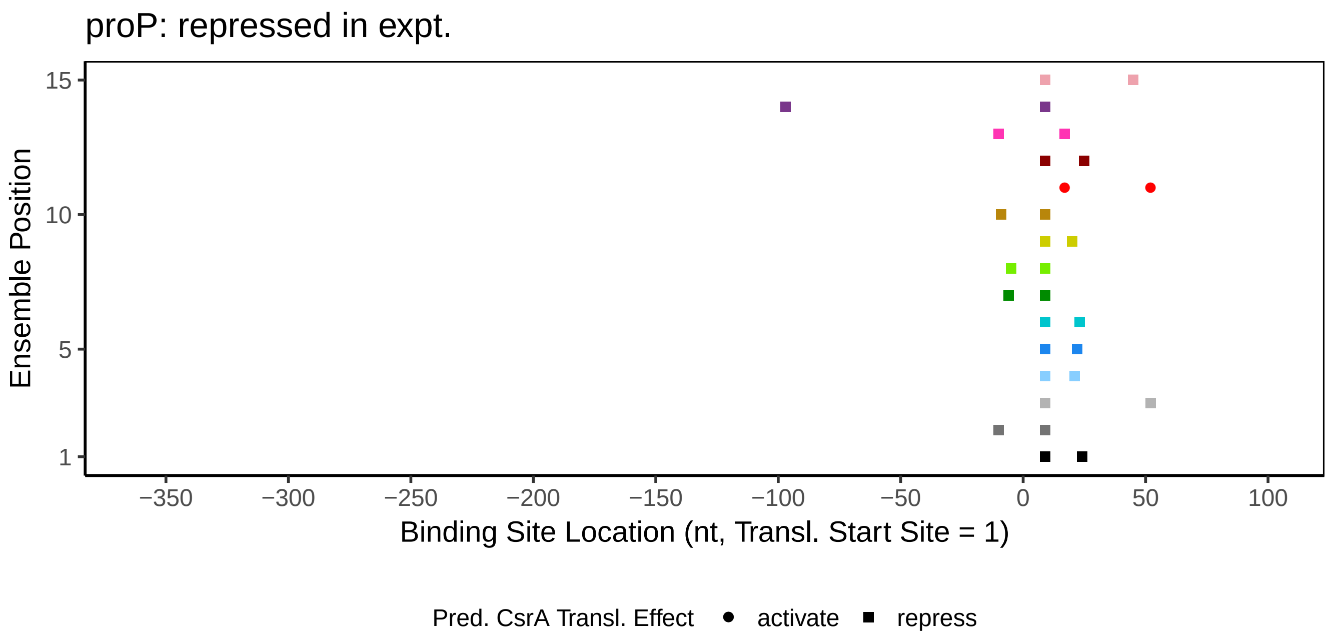

proP: repressed in expt.
Ensemble Position
Binding Site Location (nt, Transl. Start Site = 1)
Pred. CsrA Transl. Effect
activate
repress

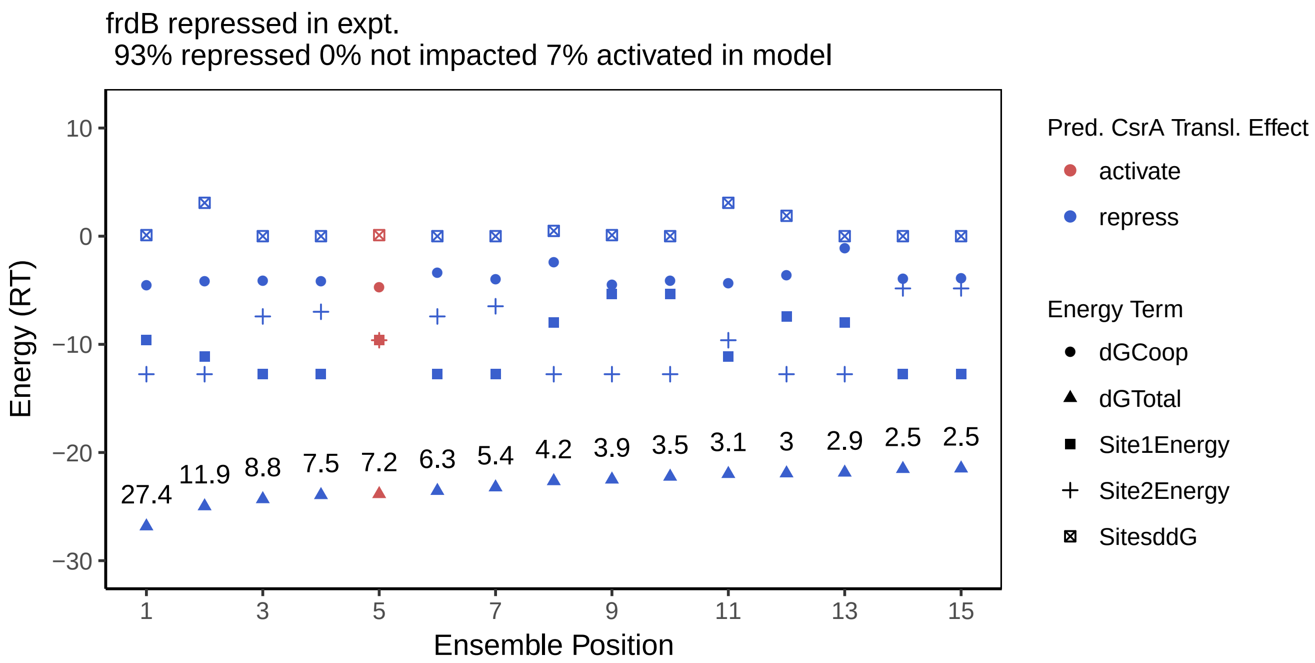
frdB repressed in expt.
93% repressed 0% not impacted 7% activated in model
Energy (RT)
10
0
−10
−20
−30
27.4
11.9
8.8
7.5
7.2
6.3
5.4
4.2
3.9
3.5
3.1
3
2.9
2.5
2.5
1
3
5
7
9
11
13
15
Ensemble Position
Pred. CsrA Transl. Effect
activate
repress
Energy Term
dGCoop
dGTotal
Site1Energy
Site2Energy
SitesddG

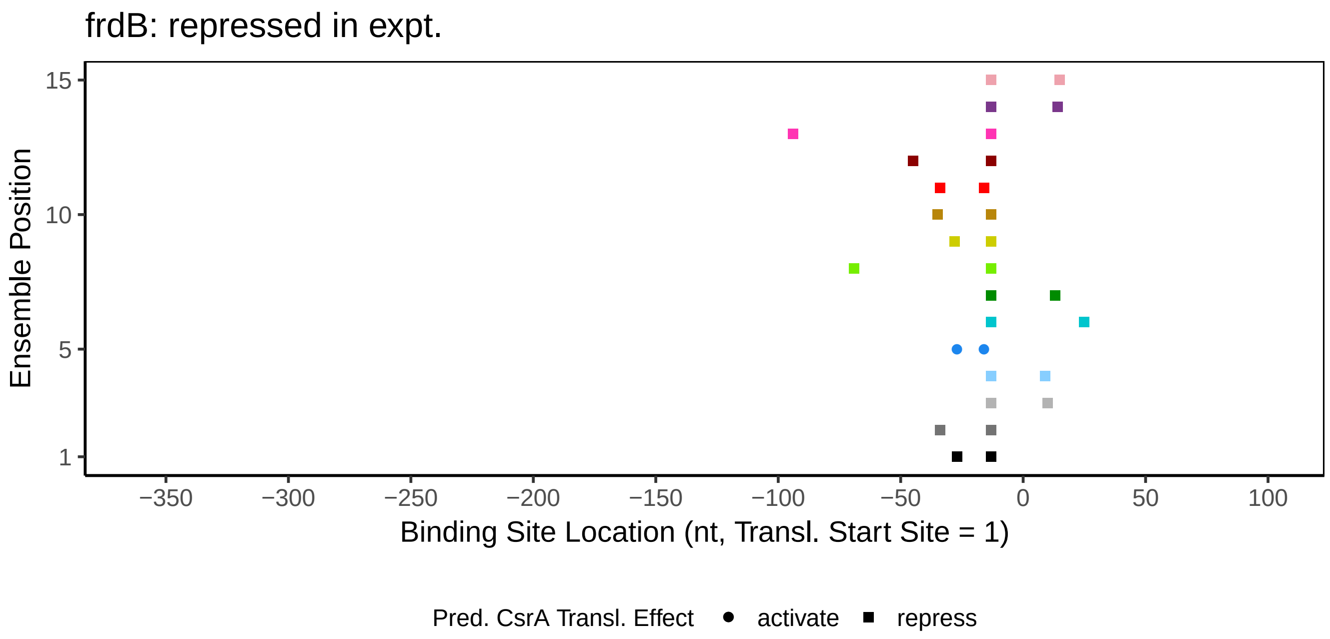
frdB: repressed in expt.
Ensemble Position
15
10
5
1
−350
−300
−250
−200
−150
−100
−50
0
50
100
Binding Site Location (nt, Transl. Start Site = 1)
Pred. CsrA Transl. Effect
activate
repress

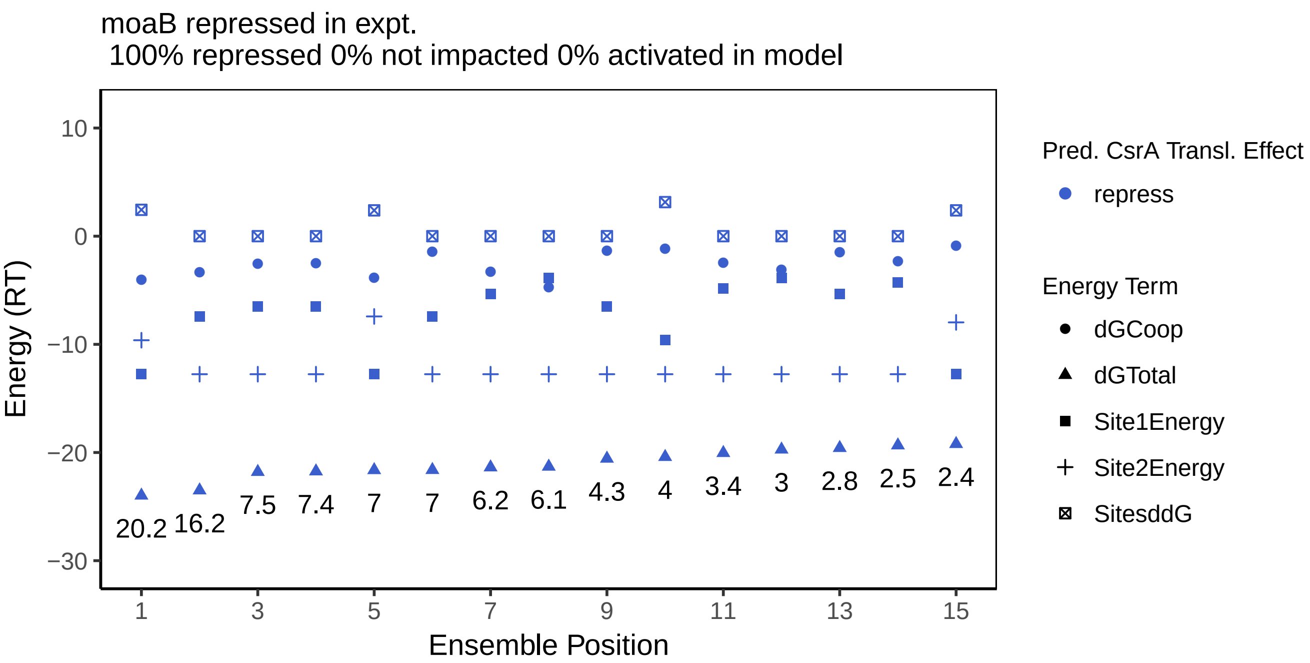
moaB repressed in expt.
100% repressed 0% not impacted 0% activated in model
Energy (RT)
10
0
−10
−20
−30
20.2
16.2
7.5
7.4
7
7
6.2
6.1
4.3
4
3.4
3
2.8
2.5
2.4
1
3
5
7
9
11
13
15
Ensemble Position
Pred. CsrA Transl. Effect
repress
Energy Term
dGCoop
dGTotal
Site1Energy
Site2Energy
SitesddG

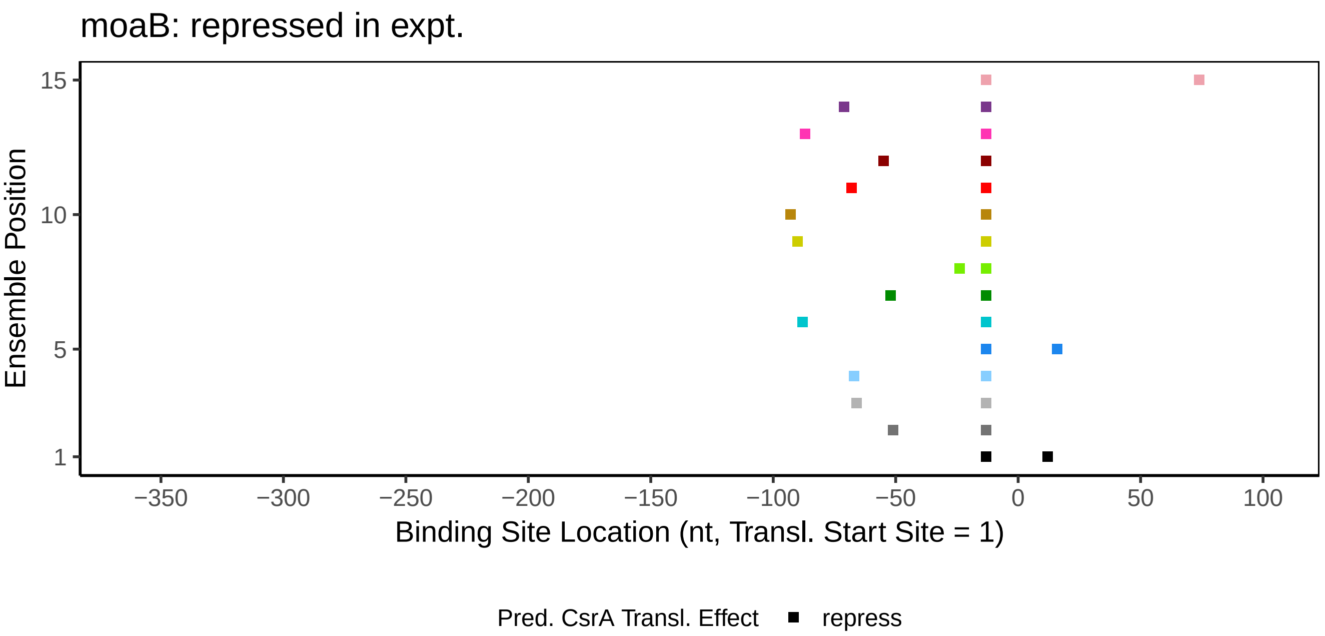
moaB: repressed in expt.
Ensemble Position
15
10
5
1
−350
−300
−250
−200
−150
−100
−50
0
50
100
Binding Site Location (nt, Transl. Start Site = 1)
Pred. CsrA Transl. Effect
repress

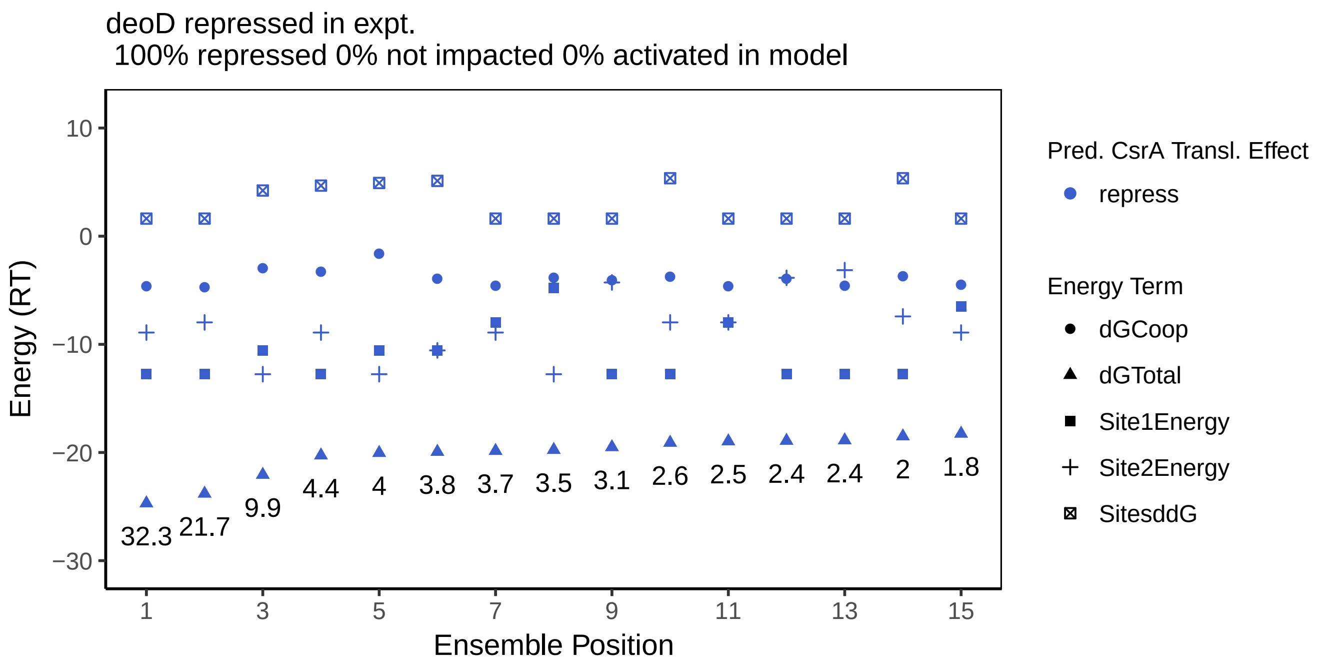
deoD repressed in expt.
100% repressed 0% not impacted 0% activated in model
Energy (RT)
10
0
−10
−20
−30
32.3
21.7
9.9
4.4
4
3.8
3.7
3.5
3.1
2.6
2.5
2.4
2.4
2
1.8
1
3
5
7
9
11
13
15
Ensemble Position
Pred. CsrA Transl. Effect
repress
Energy Term
dGCoop
dGTotal
Site1Energy
Site2Energy
SitesddG

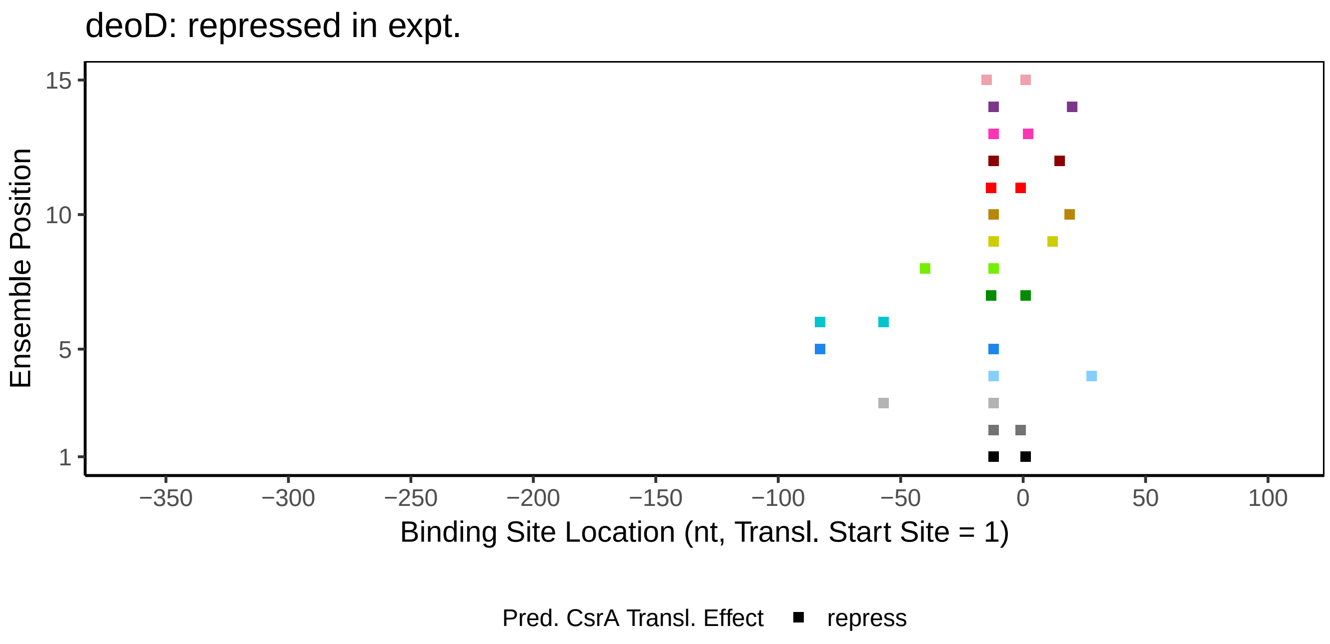
deoD: repressed in expt.
Ensemble Position
15
10
5
1
−350
−300
−250
−200
−150
−100
−50
0
50
100
Binding Site Location (nt, Transl. Start Site = 1)
Pred. CsrA Transl. Effect
repress

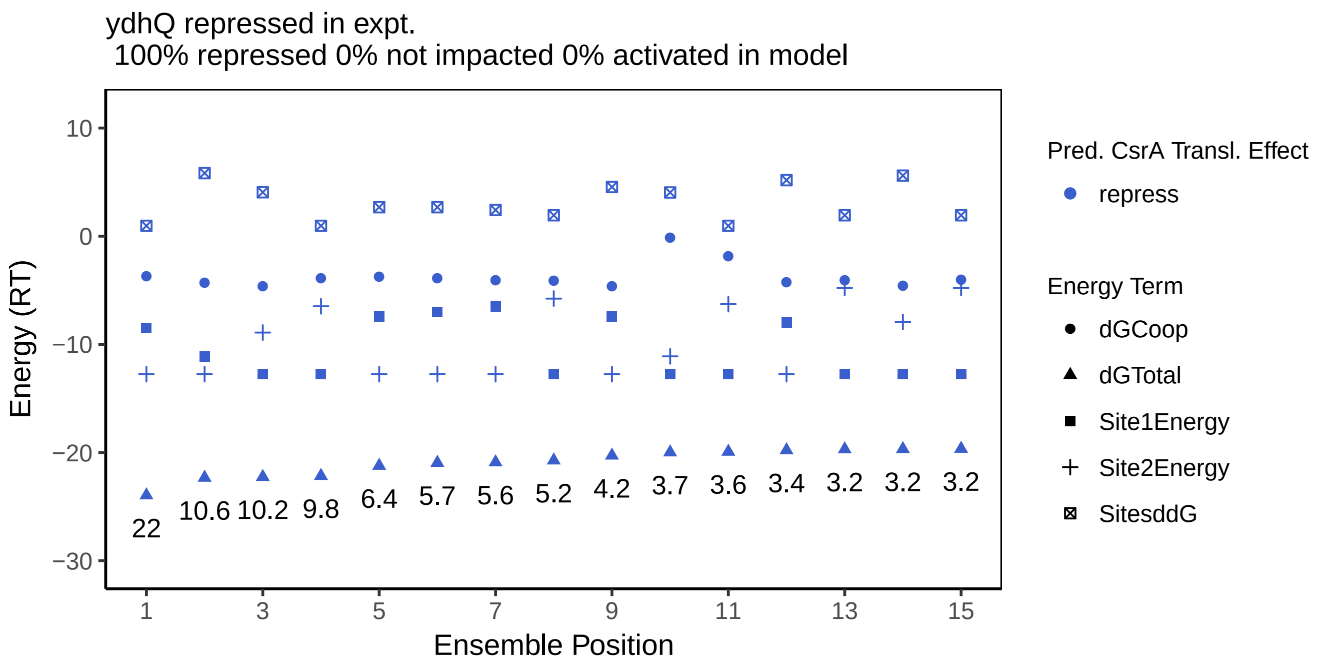

ydhQ repressed in expt.
100% repressed 0% not impacted 0% activated in model
Energy (RT)
10
0
−10
−20
−30
22
10.6
10.2
9.8
6.4
5.7
5.6
5.2
4.2
3.7
3.6
3.4
3.2
3.2
3.2
1
3
5
7
9
11
13
15
Ensemble Position
Pred. CsrA Transl. Effect
repress
Energy Term
dGCoop
dGTotal
Site1Energy
Site2Energy
SitesddG

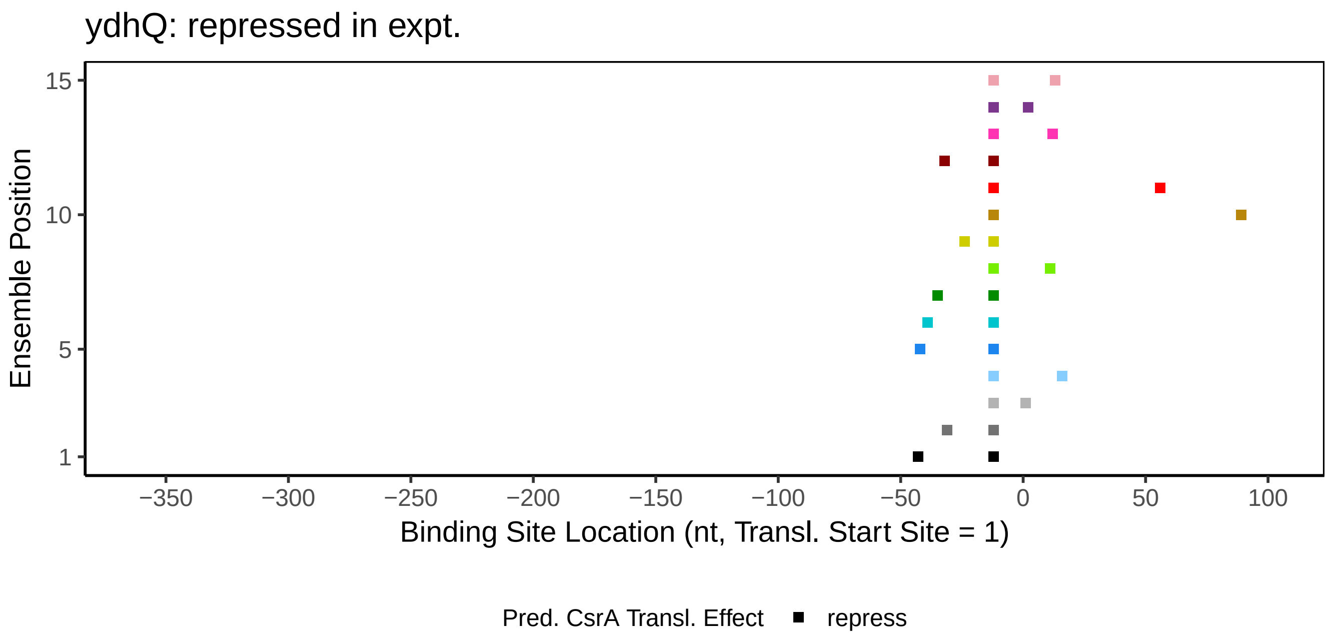

ydhQ: repressed in expt.
Ensemble Position
15
10
5
1
−350
−300
−250
−200
−150
−100
−50
0
50
100
Binding Site Location (nt, Transl. Start Site = 1)
Pred. CsrA Transl. Effect
repress

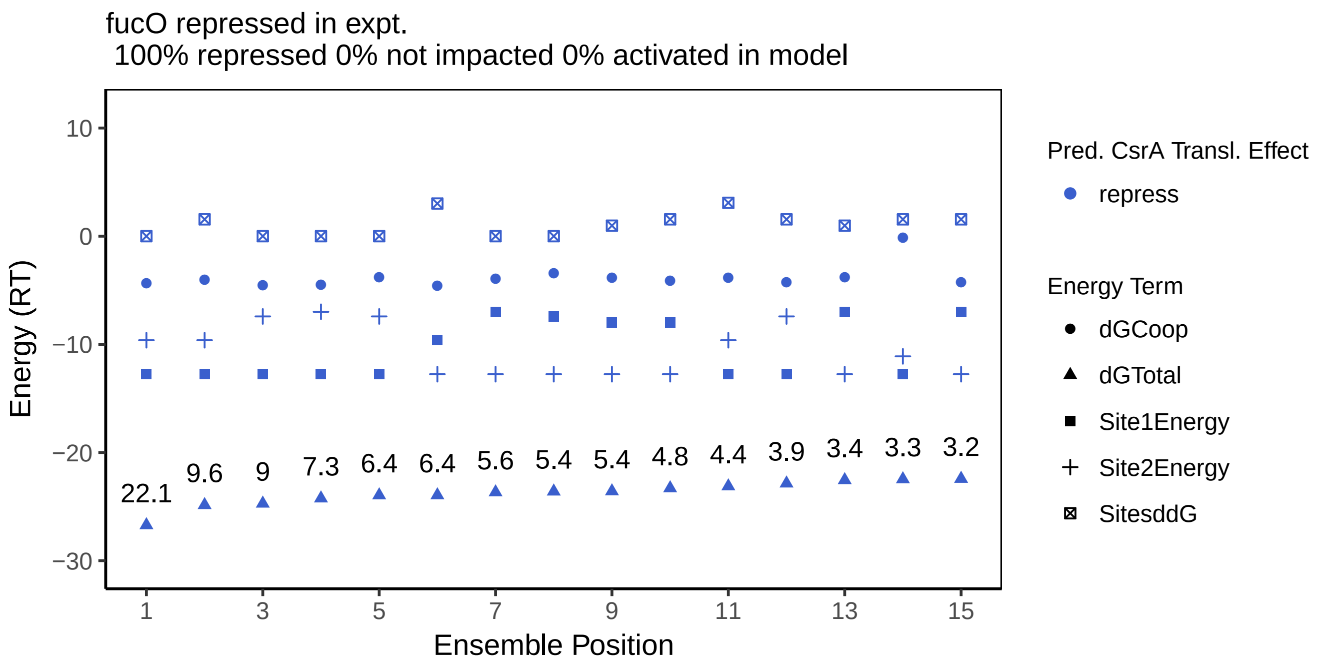
fucO repressed in expt.
100% repressed 0% not impacted 0% activated in model
Energy (RT)
Ensemble Position
Pred. CsrA Transl. Effect
repress
Energy Term
dGCoop
dGTotal
Site1Energy
Site2Energy
SitesddG
22.1
9.6
9
7.3
6.4
6.4
5.6
5.4
5.4
4.8
4.4
3.9
3.4
3.3
3.2

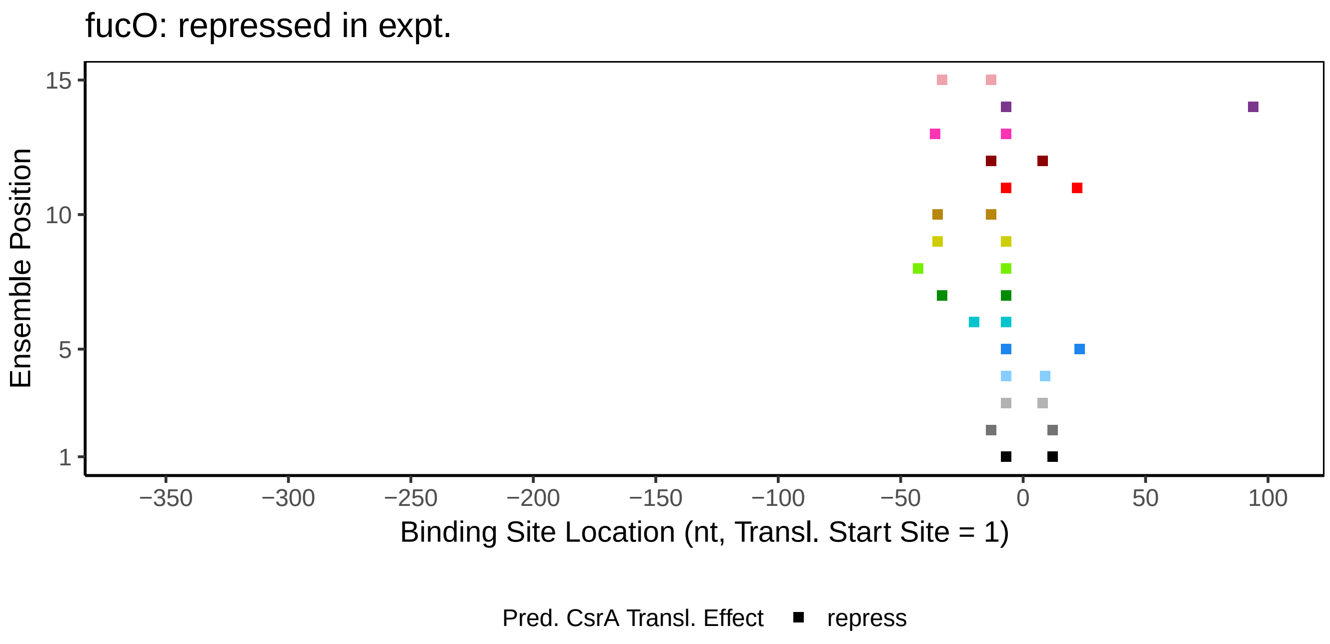
fucO: repressed in expt.
Ensemble Position
Binding Site Location (nt, Transl. Start Site = 1)
Pred. CsrA Transl. Effect
repress

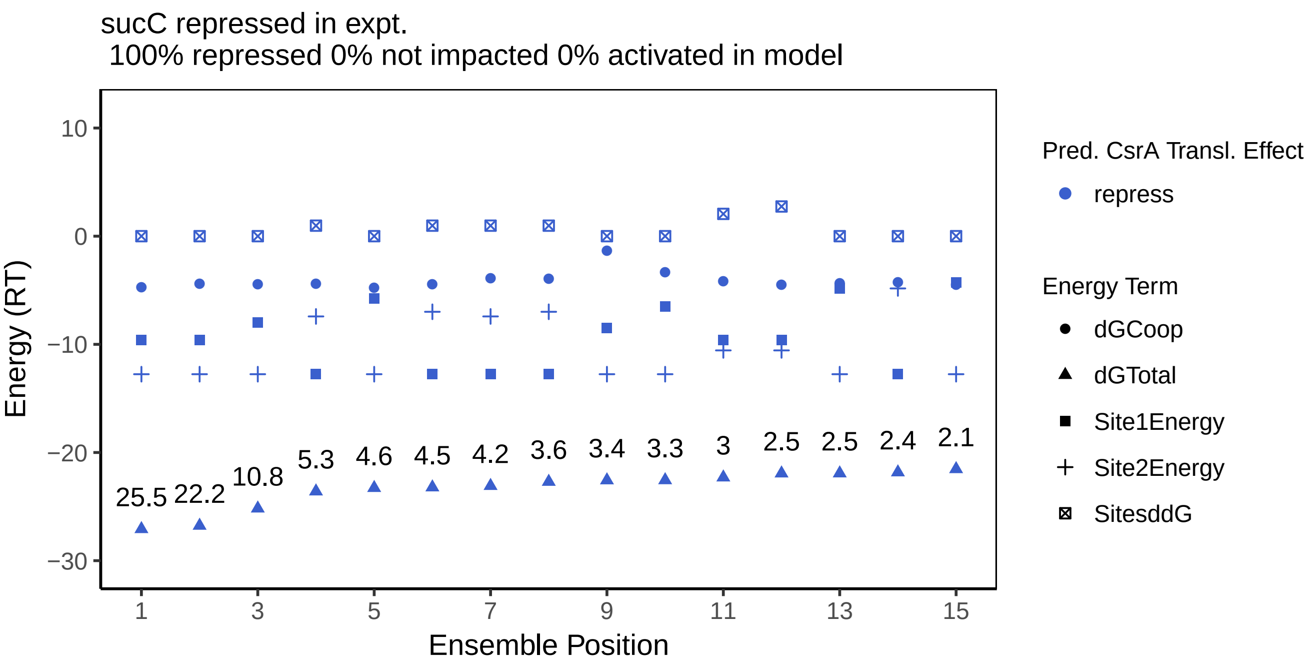

sucC repressed in expt.
100% repressed 0% not impacted 0% activated in model
Energy (RT)
Ensemble Position
25.5
22.2
10.8
5.3
4.6
4.5
4.2
3.6
3.4
3.3
3
2.5
2.5
2.4
2.1
Pred. CsrA Transl. Effect
repress
Energy Term
dGCoop
dGTotal
Site1Energy
Site2Energy
SitesddG

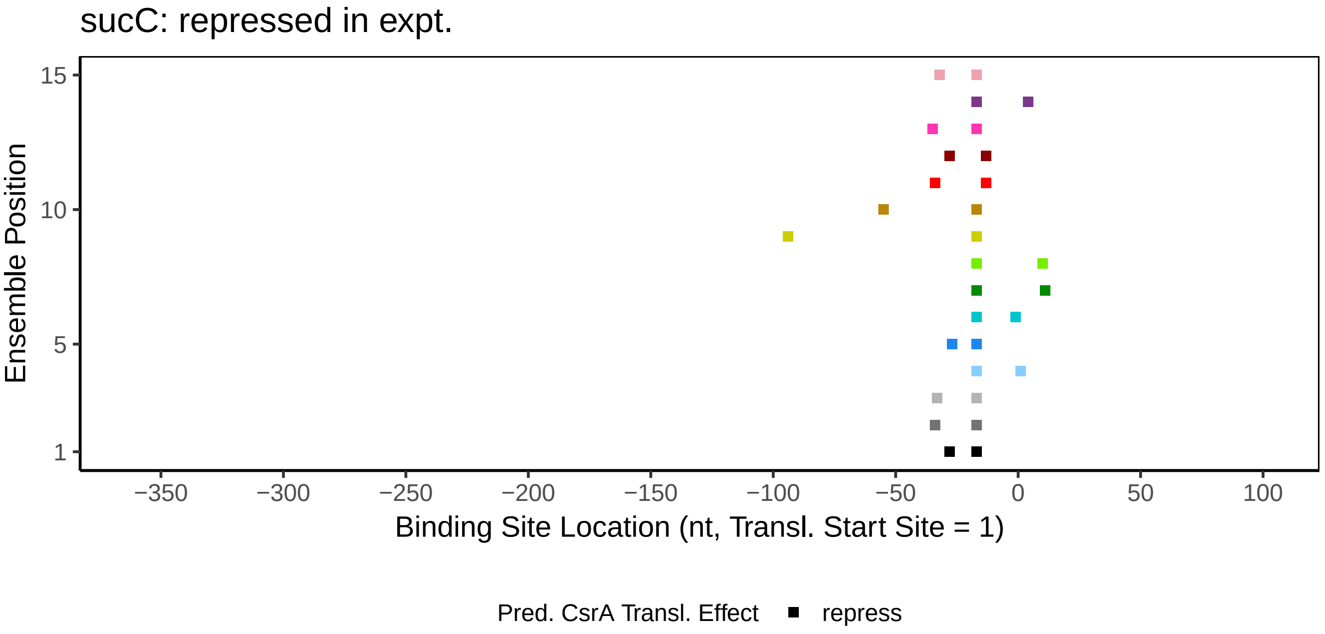

sucC: repressed in expt.
Ensemble Position
Binding Site Location (nt, Transl. Start Site = 1)
Pred. CsrA Transl. Effect
repress

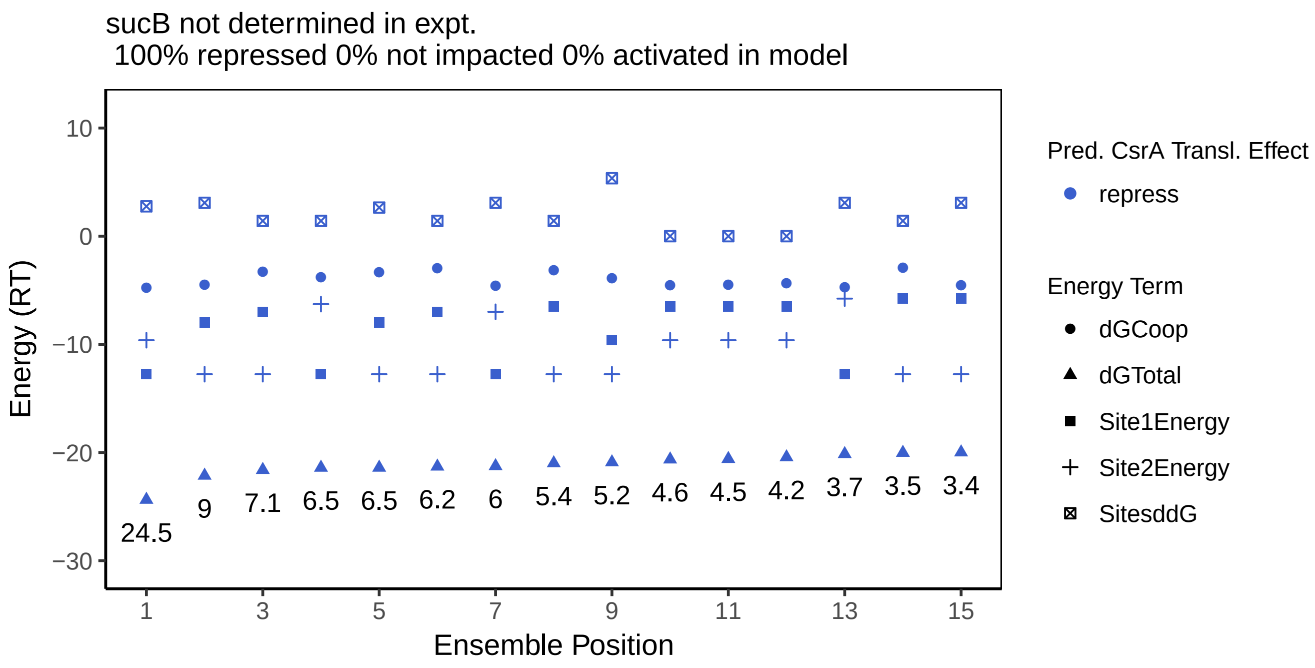
sucB not determined in expt.
100% repressed 0% not impacted 0% activated in model
Energy (RT)
Ensemble Position
Pred. CsrA Transl. Effect
repress
Energy Term
dGCoop
dGTotal
Site1Energy
Site2Energy
SitesddG
24.5
9
7.1
6.5
6.5
6.2
6
5.4
5.2
4.6
4.5
4.2
3.7
3.5
3.4

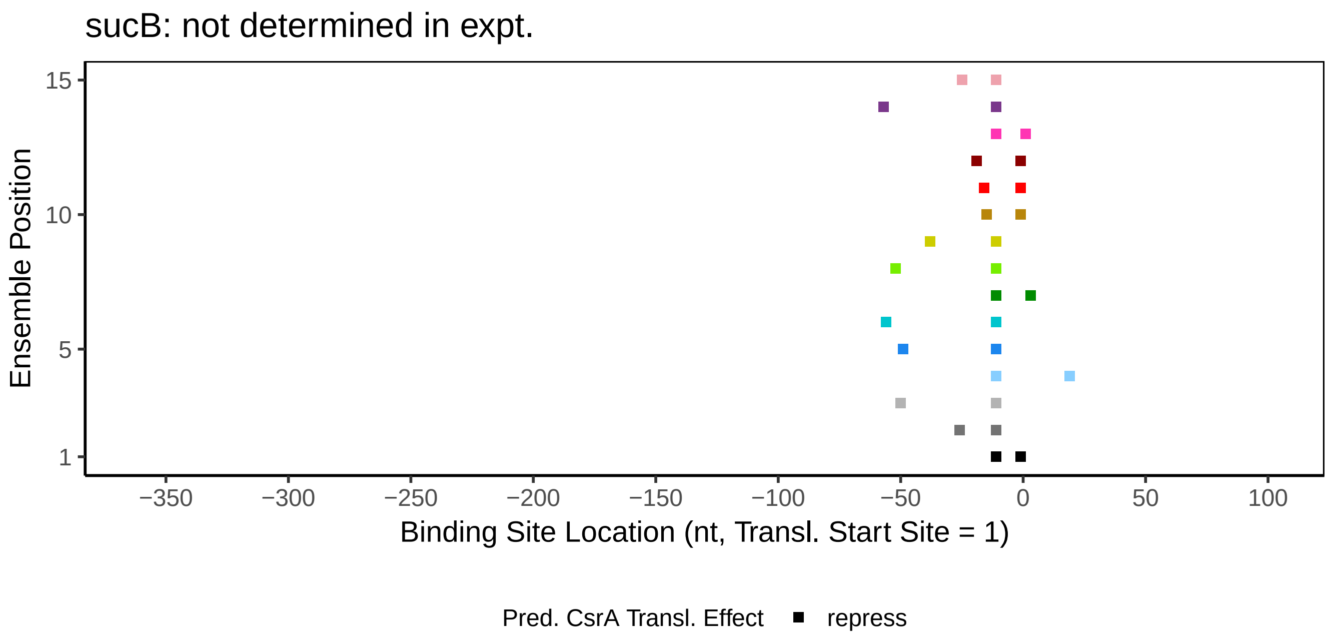
sucB: not determined in expt.
Ensemble Position
Binding Site Location (nt, Transl. Start Site = 1)
Pred. CsrA Transl. Effect
repress

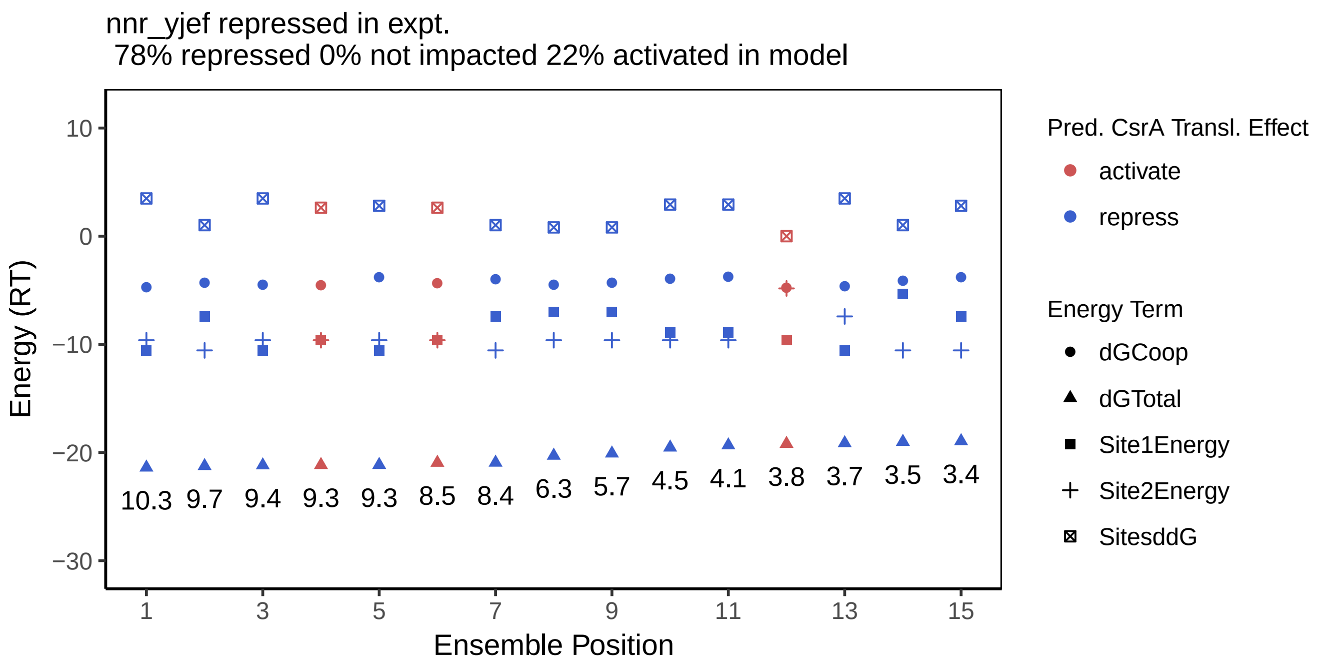

nnr_yjef repressed in expt.
78% repressed 0% not impacted 22% activated in model
Energy (RT)
Ensemble Position
10.3
9.7
9.4
9.3
9.3
8.5
8.4
6.3
5.7
4.5
4.1
3.8
3.7
3.5
3.4
Pred. CsrA Transl. Effect
activate
repress
Energy Term
dGCoop
dGTotal
Site1Energy
Site2Energy
SitesddG

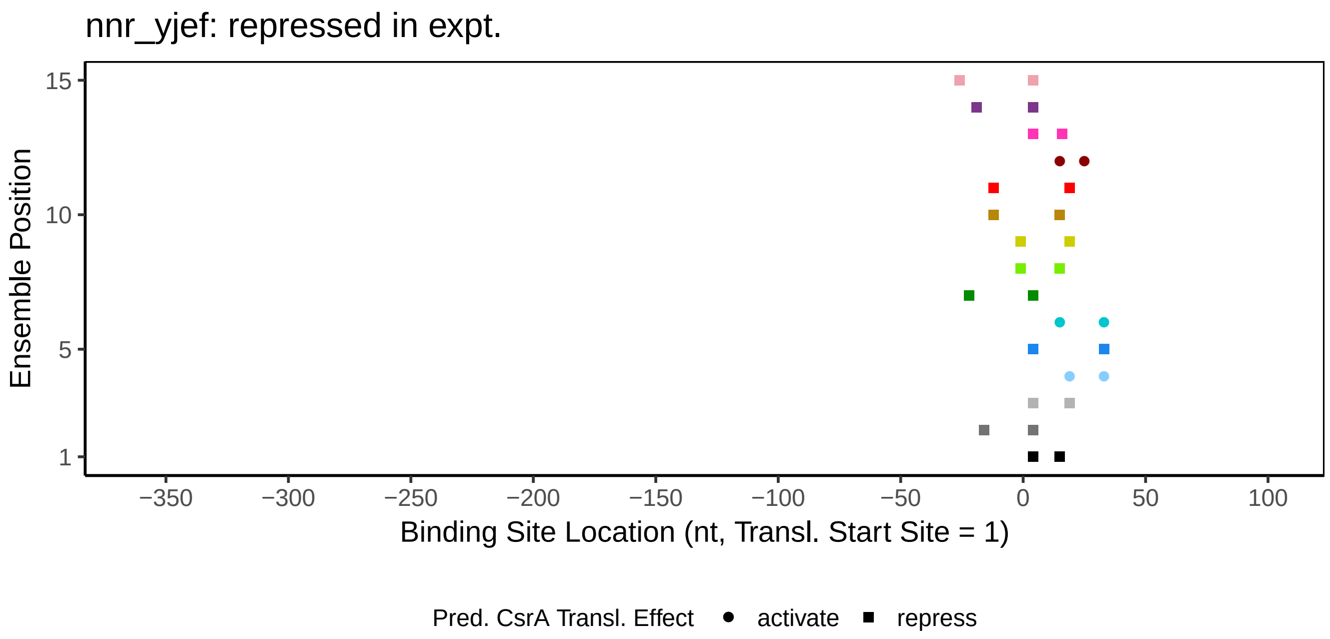

nnr_yjef: repressed in expt.
Ensemble Position
Binding Site Location (nt, Transl. Start Site = 1)
Pred. CsrA Transl. Effect
activate
repress

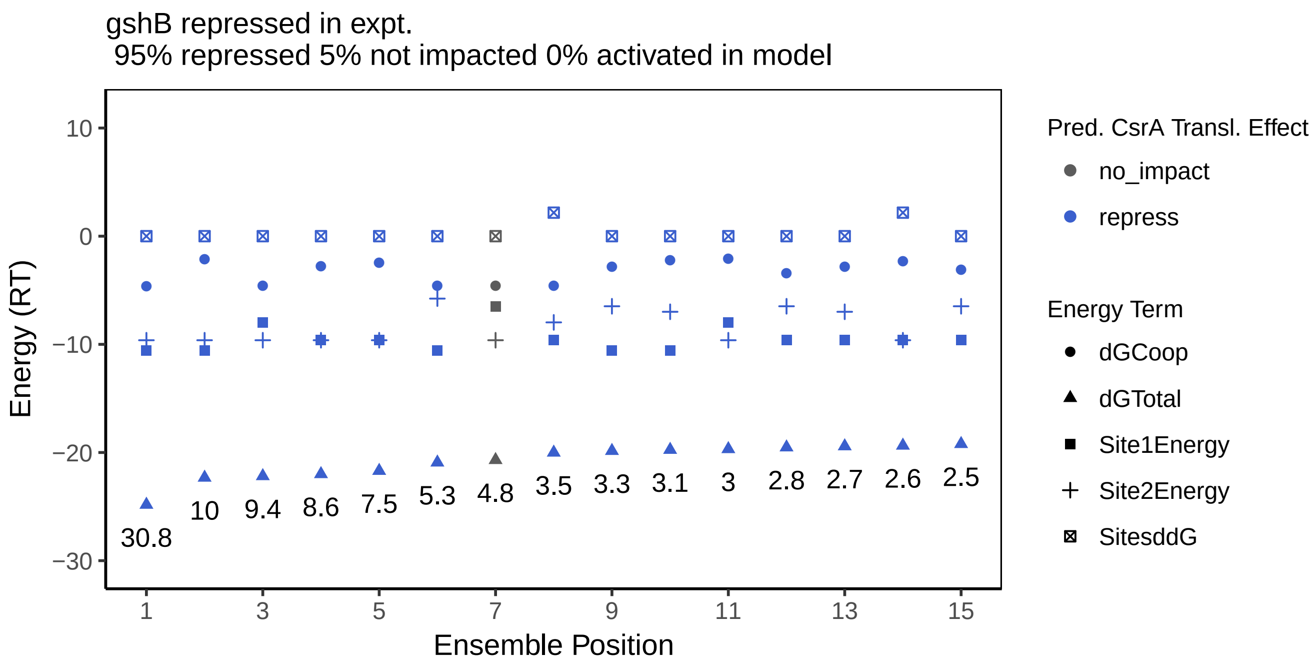
gshB repressed in expt.
95% repressed 5% not impacted 0% activated in model
Energy (RT)
10
0
−10
−20
−30
30.8
10
9.4
8.6
7.5
5.3
4.8
3.5
3.3
3.1
3
2.8
2.7
2.6
2.5
1
3
5
7
9
11
13
15
Ensemble Position
Pred. CsrA Transl. Effect
no_impact
repress
Energy Term
dGCoop
dGTotal
Site1Energy
Site2Energy
SitesddG

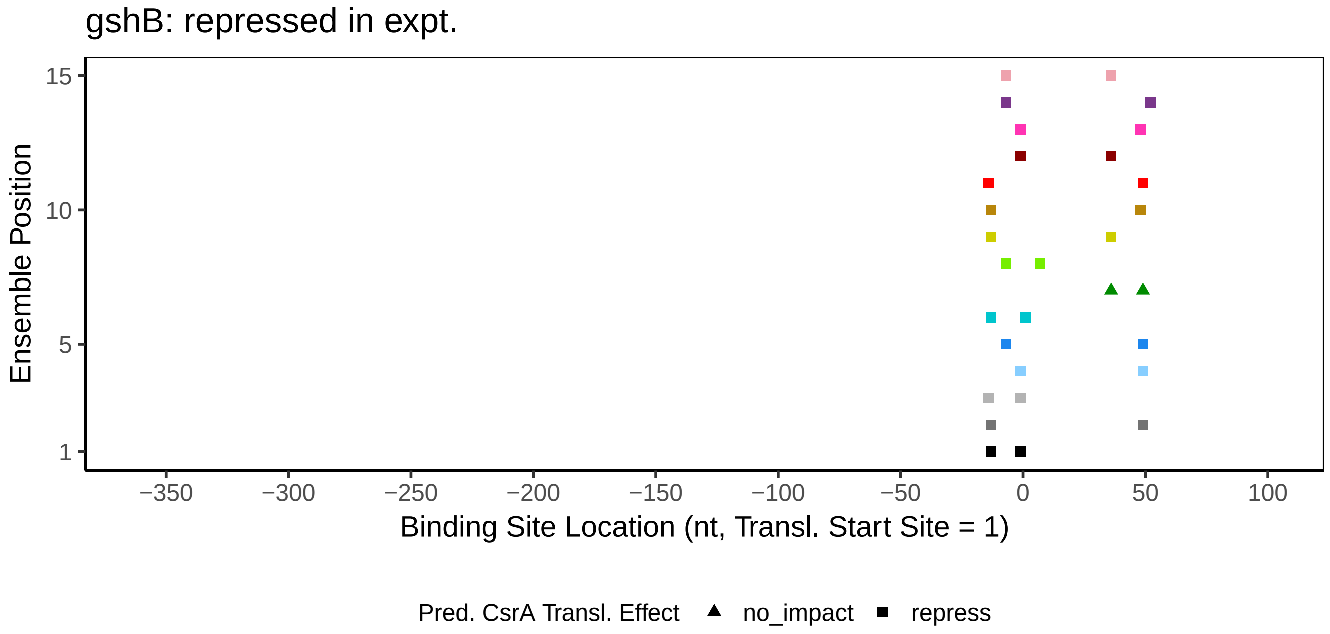
gshB: repressed in expt.
Ensemble Position
15
10
5
1
−350
−300
−250
−200
−150
−100
−50
0
50
100
Binding Site Location (nt, Transl. Start Site = 1)
Pred. CsrA Transl. Effect
no_impact
repress

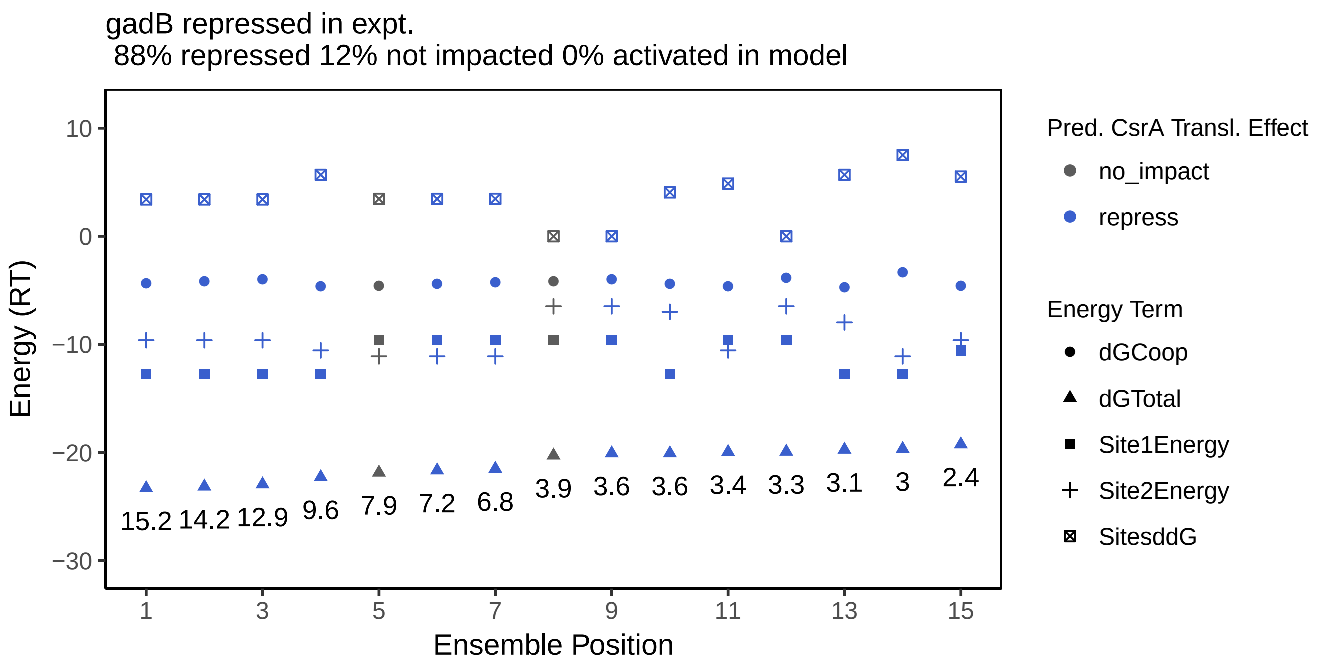
gadB repressed in expt.
88% repressed 12% not impacted 0% activated in model
Energy (RT)
10
0
−10
−20
−30
15.2
14.2
12.9
9.6
7.9
7.2
6.8
3.9
3.6
3.6
3.4
3.3
3.1
3
2.4
1
3
5
7
9
11
13
15
Ensemble Position
Pred. CsrA Transl. Effect
no_impact
repress
Energy Term
dGCoop
dGTotal
Site1Energy
Site2Energy
SitesddG

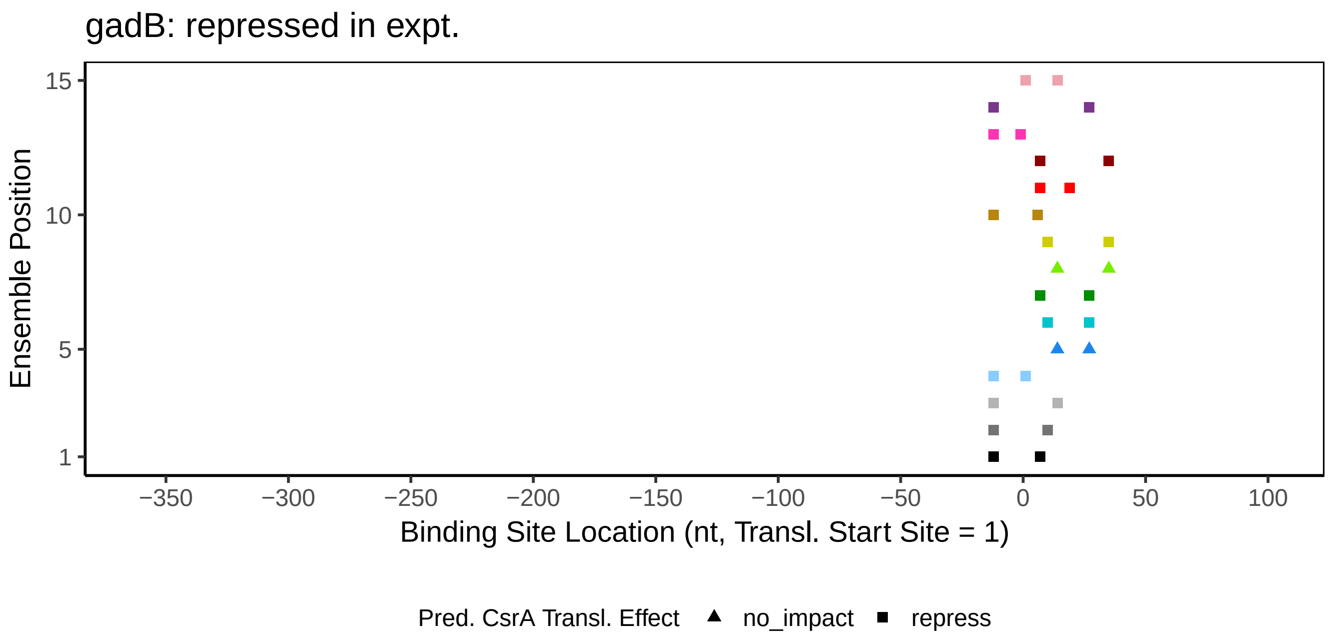
gadB: repressed in expt.
Ensemble Position
15
10
5
1
−350
−300
−250
−200
−150
−100
−50
0
50
100
Binding Site Location (nt, Transl. Start Site = 1)
Pred. CsrA Transl. Effect
no_impact
repress

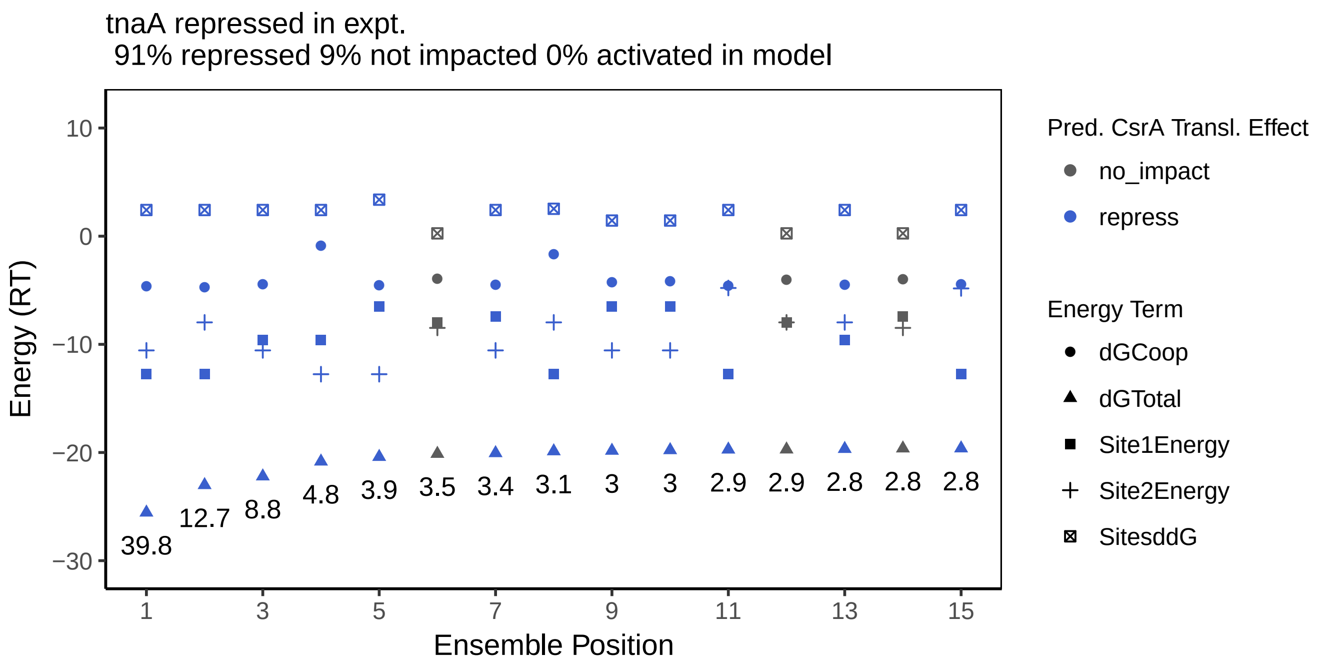
tnaA repressed in expt.
91% repressed 9% not impacted 0% activated in model
Energy (RT)
Ensemble Position
39.8
12.7
8.8
4.8
3.9
3.5
3.4
3.1
3
3
2.9
2.9
2.8
2.8
2.8
Pred. CsrA Transl. Effect
no_impact
repress
Energy Term
dGCoop
dGTotal
Site1Energy
Site2Energy
SitesddG

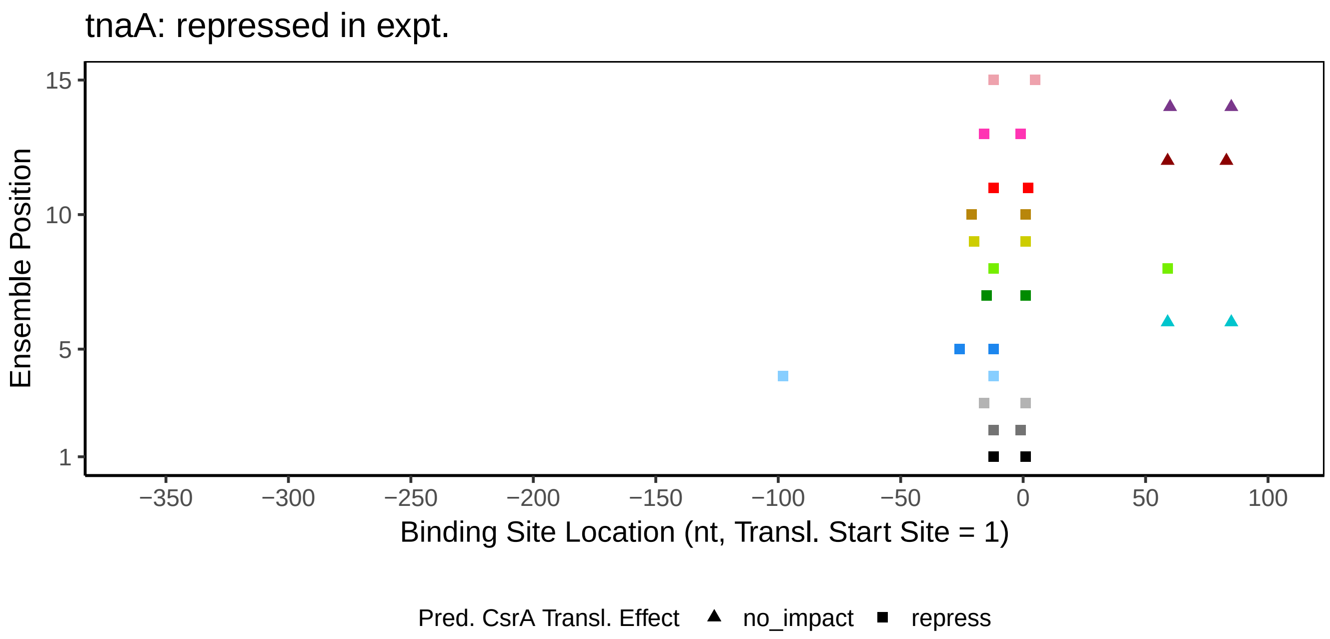
tnaA: repressed in expt.
Ensemble Position
Binding Site Location (nt, Transl. Start Site = 1)
Pred. CsrA Transl. Effect
no_impact
repress

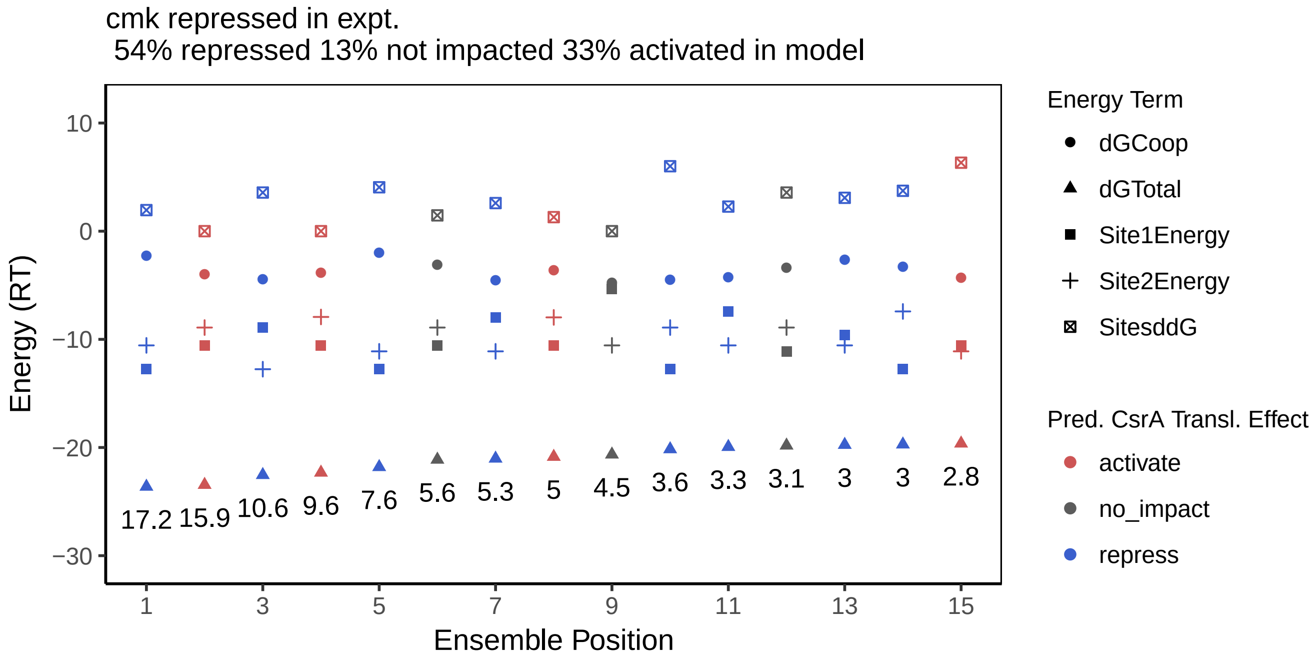

cmk repressed in expt.
54% repressed 13% not impacted 33% activated in model
Energy (RT)
Ensemble Position
17.2 15.9 10.6 9.6 7.6 5.6 5.3 5 4.5 3.6 3.3 3.1 3 3 2.8
Energy Term
dGCoop
dGTotal
Site1Energy
Site2Energy
SitesddG
Pred. CsrA Transl. Effect
activate
no_impact
repress

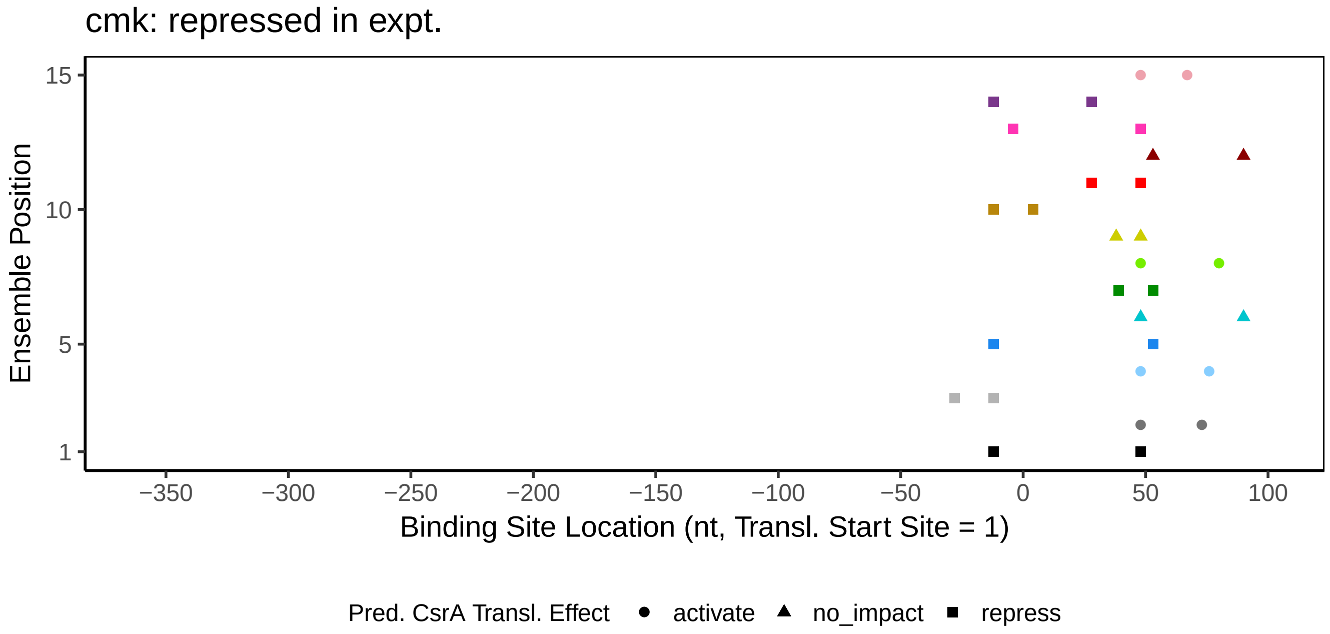

cmk: repressed in expt.
Ensemble Position
Binding Site Location (nt, Transl. Start Site = 1)
Pred. CsrA Transl. Effect
activate
no_impact
repress

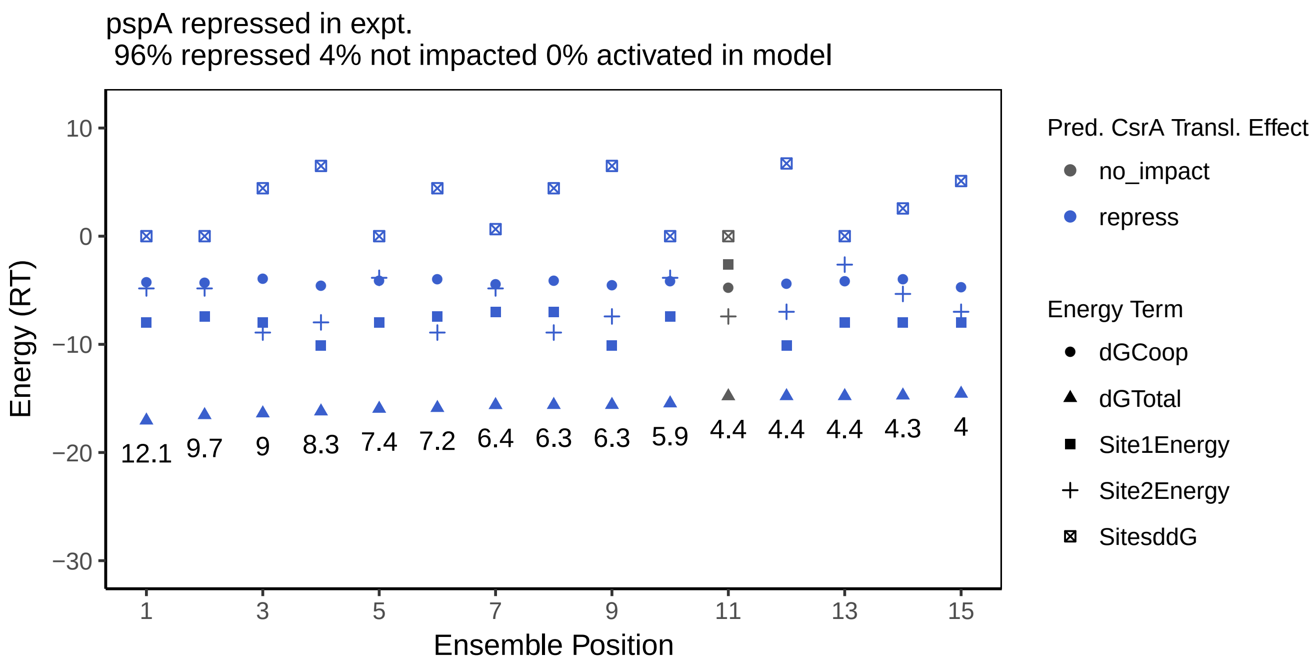

pspA repressed in expt.
96% repressed 4% not impacted 0% activated in model
Energy (RT)
10
0
−10
−20
−30
12.1 9.7 9 8.3 7.4 7.2 6.4 6.3 6.3 5.9 4.4 4.4 4.4 4.3 4
1 3 5 7 9 11 13 15
Ensemble Position
Pred. CsrA Transl. Effect
no_impact
repress
Energy Term
dGCoop
dGTotal
Site1Energy
Site2Energy
SitesddG

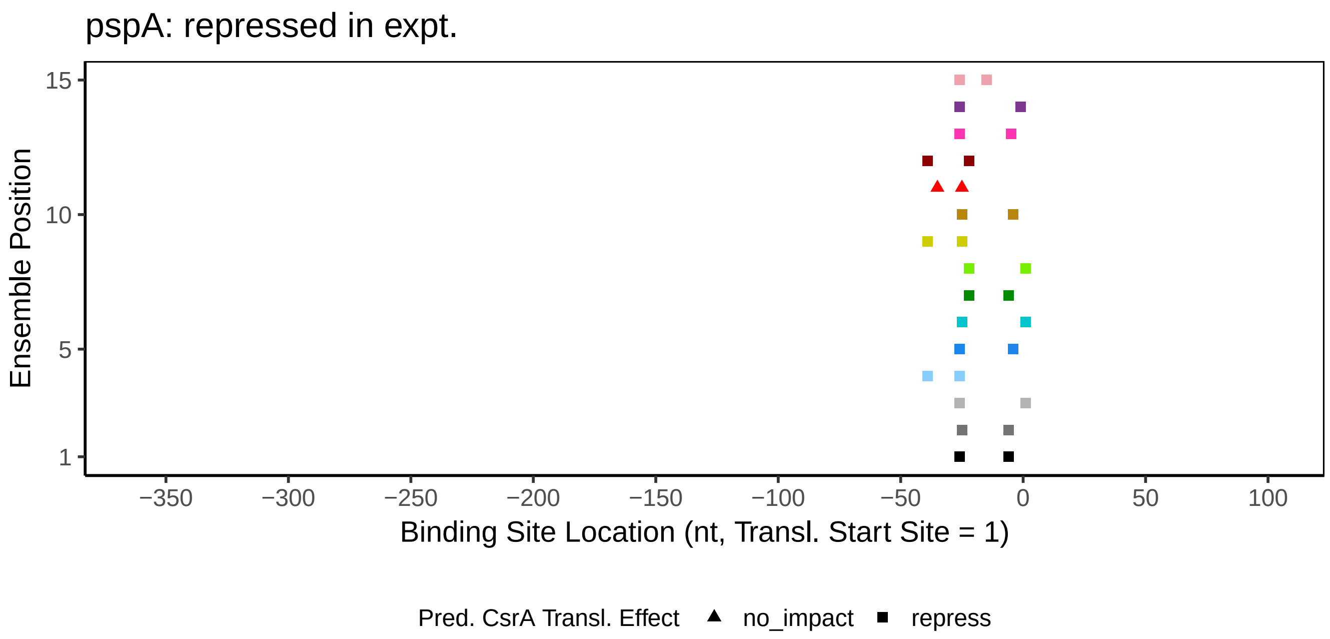

pspA: repressed in expt.
Ensemble Position
15
10
5
1
−350 −300 −250 −200 −150 −100 −50 0 50 100
Binding Site Location (nt, Transl. Start Site = 1)
Pred. CsrA Transl. Effect
no_impact
repress

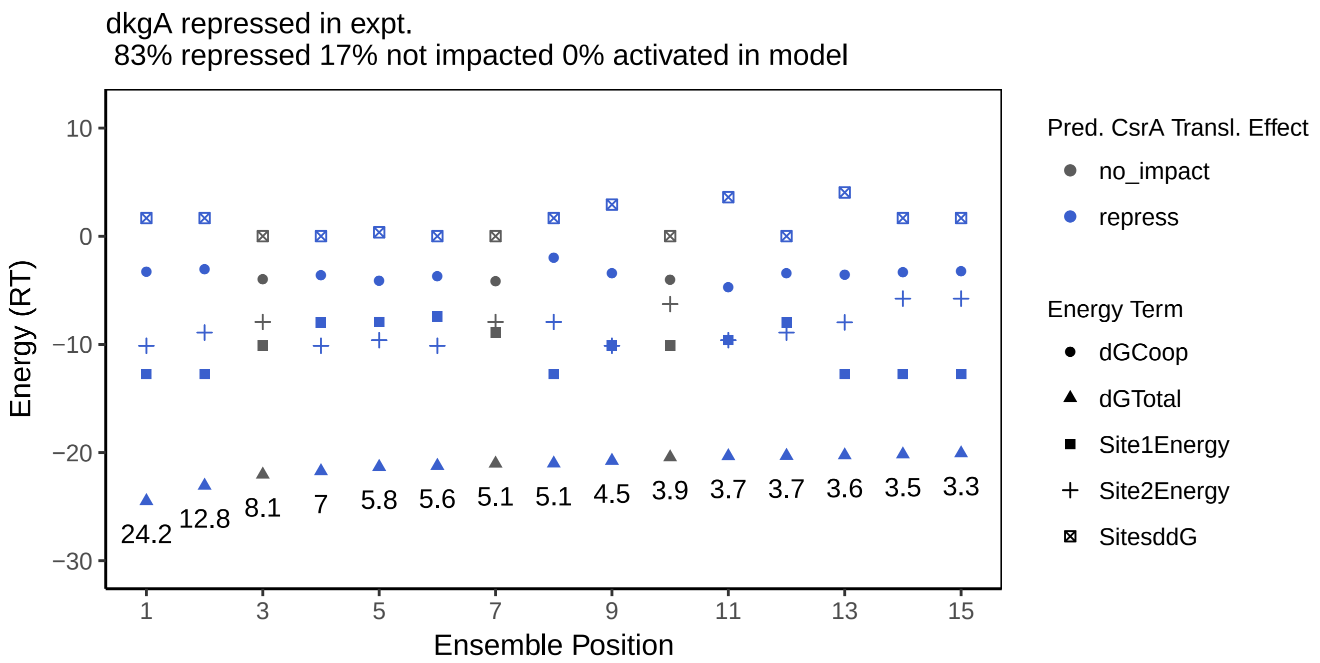

dkgA repressed in expt.
83% repressed 17% not impacted 0% activated in model
Energy (RT)
Ensemble Position
24.2
12.8
8.1
7
5.8
5.6
5.1
5.1
4.5
3.9
3.7
3.7
3.6
3.5
3.3
Pred. CsrA Transl. Effect
no_impact
repress
Energy Term
dGCoop
dGTotal
Site1Energy
Site2Energy
SitesddG

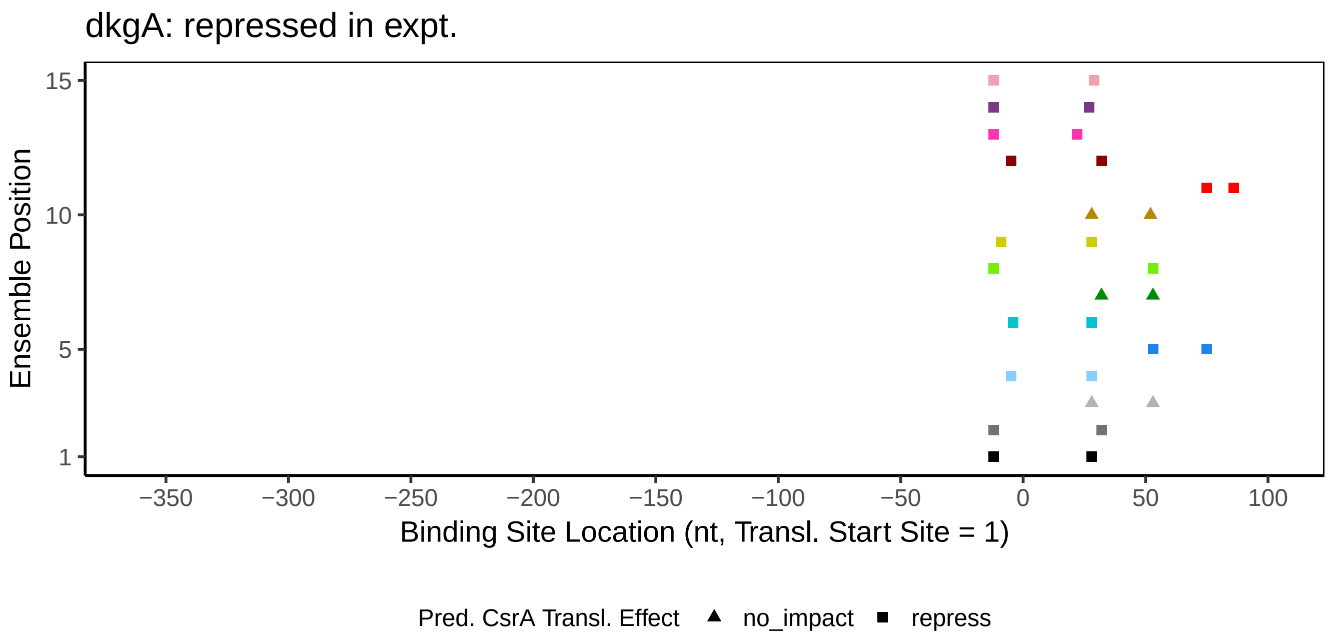

dkgA: repressed in expt.
Ensemble Position
Binding Site Location (nt, Transl. Start Site = 1)
Pred. CsrA Transl. Effect
no_impact
repress

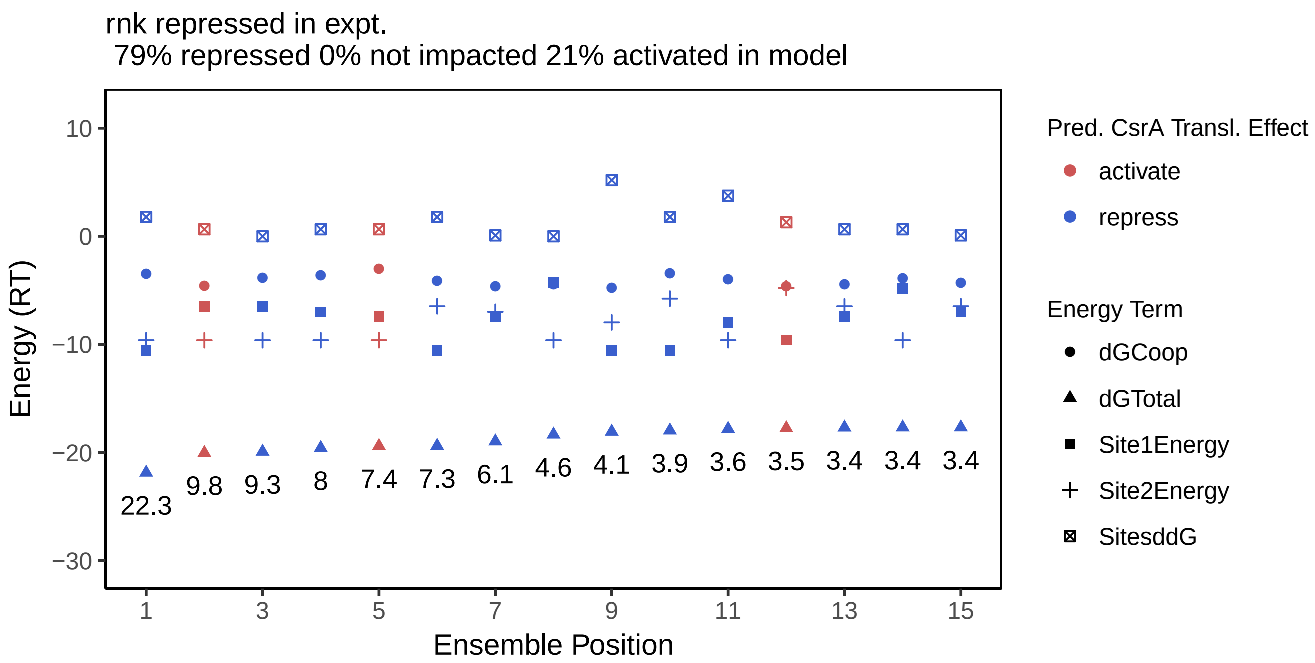

rnk repressed in expt.
79% repressed 0% not impacted 21% activated in model
Energy (RT)
10
0
−10
−20
−30
22.3
9.8
9.3
8
7.4
7.3
6.1
4.6
4.1
3.9
3.6
3.5
3.4
3.4
3.4
1
3
5
7
9
11
13
15
Ensemble Position
Pred. CsrA Transl. Effect
activate
repress
Energy Term
dGCoop
dGTotal
Site1Energy
Site2Energy
SitesddG

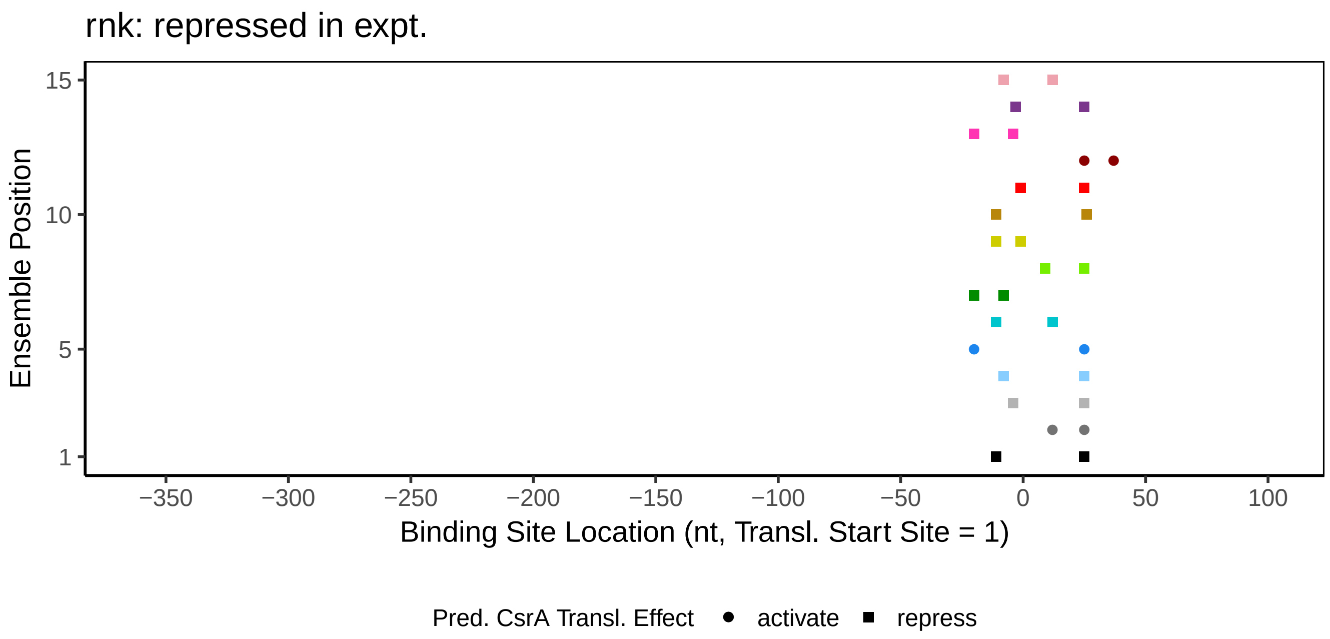

rnk: repressed in expt.
Ensemble Position
15
10
5
1
−350
−300
−250
−200
−150
−100
−50
0
50
100
Binding Site Location (nt, Transl. Start Site = 1)
Pred. CsrA Transl. Effect
activate
repress

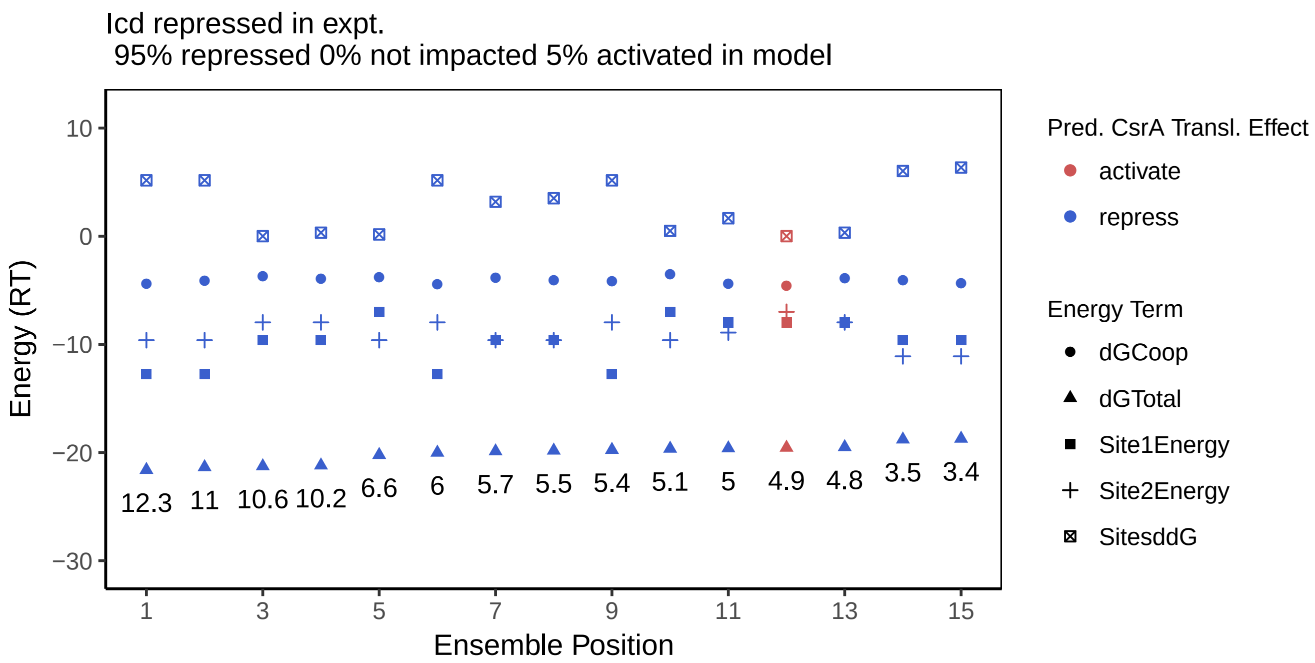
Icd repressed in expt.
95% repressed 0% not impacted 5% activated in model
Energy (RT)
10
0
−10
−20
−30
12.3
11
10.6
10.2
6.6
6
5.7
5.5
5.4
5.1
5
4.9
4.8
3.5
3.4
1
3
5
7
9
11
13
15
Ensemble Position
Pred. CsrA Transl. Effect
activate
repress
Energy Term
dGCoop
dGTotal
Site1Energy
Site2Energy
SitesddG

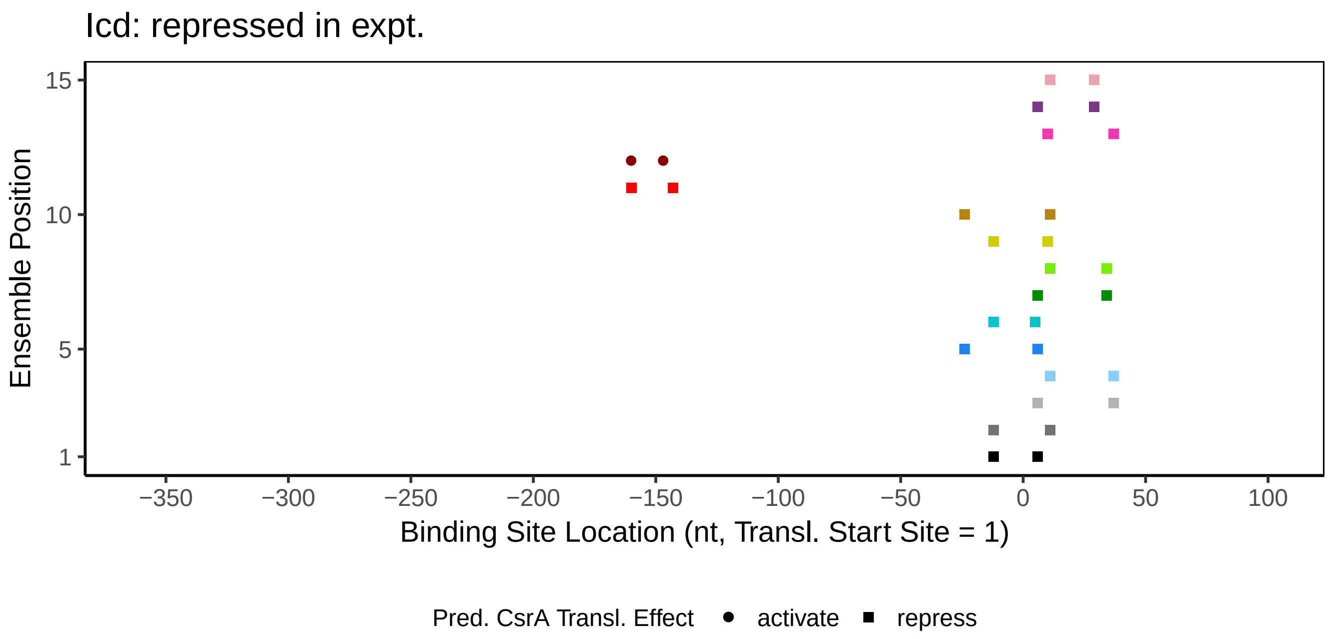
Icd: repressed in expt.
Ensemble Position
15
10
5
1
−350
−300
−250
−200
−150
−100
−50
0
50
100
Binding Site Location (nt, Transl. Start Site = 1)
Pred. CsrA Transl. Effect
activate
repress

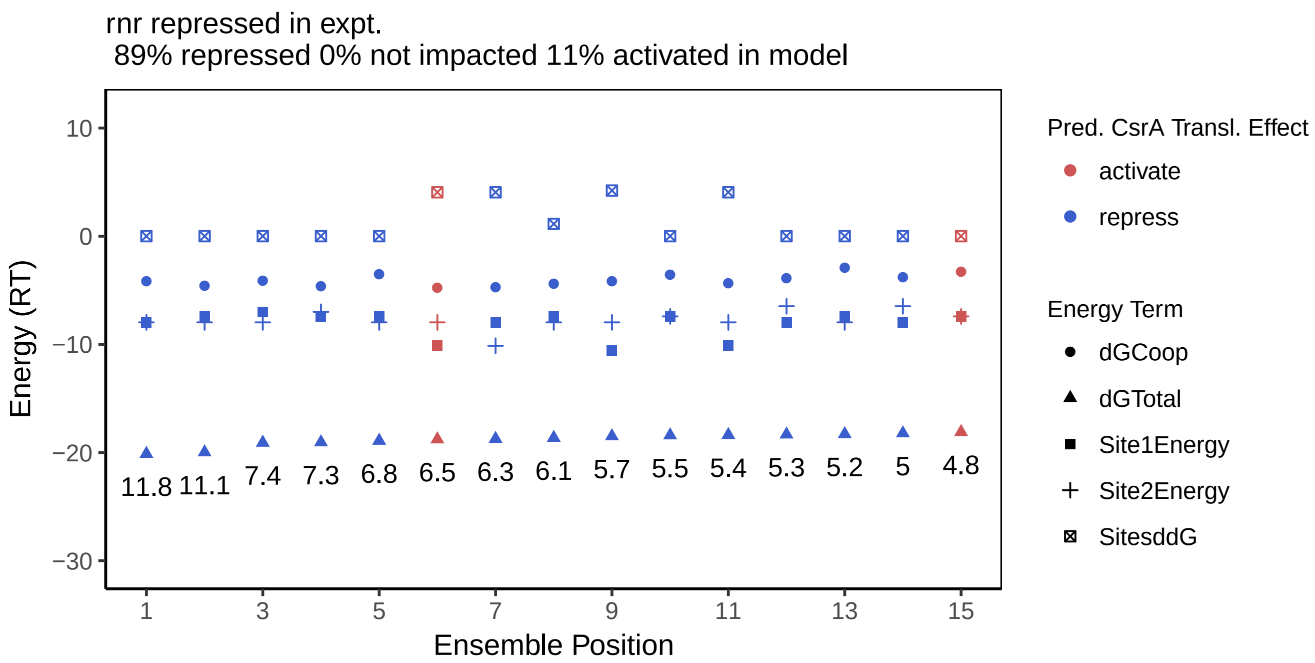
rnr repressed in expt.
89% repressed 0% not impacted 11% activated in model
Energy (RT)
Ensemble Position
11.8 11.1 7.4 7.3 6.8 6.5 6.3 6.1 5.7 5.5 5.4 5.3 5.2 5 4.8
Pred. CsrA Transl. Effect
activate
repress
Energy Term
dGCoop
dGTotal
Site1Energy
Site2Energy
SitesddG

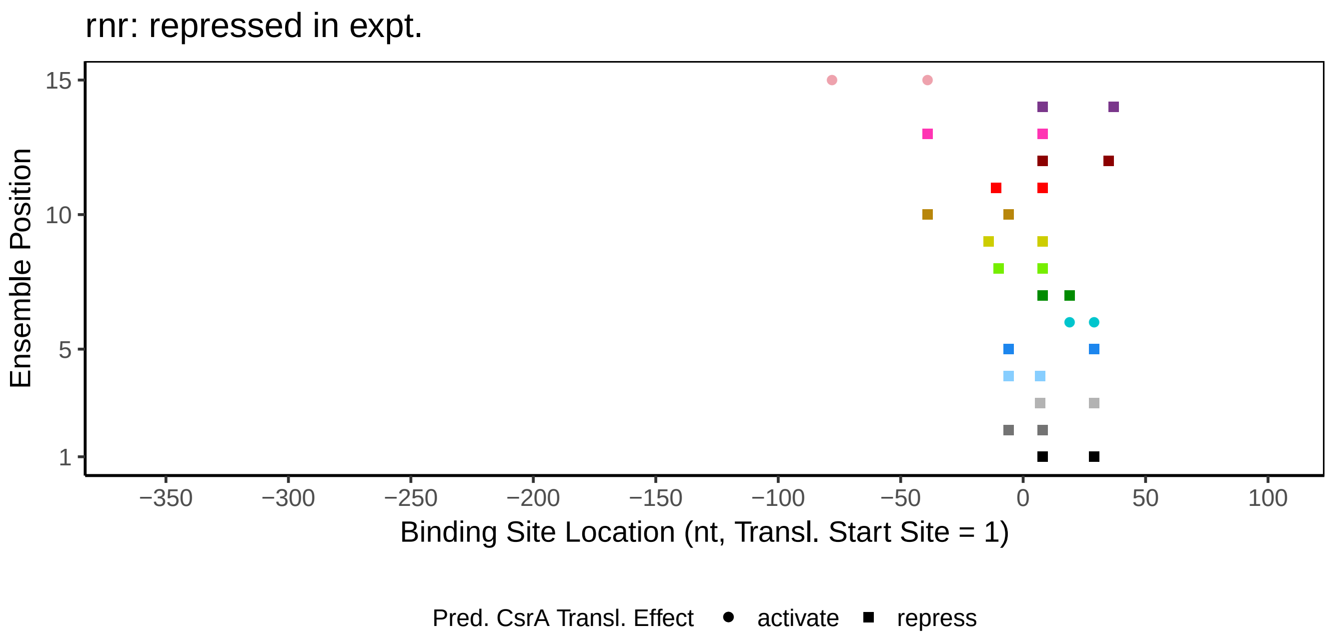
rnr: repressed in expt.
Ensemble Position
Binding Site Location (nt, Transl. Start Site = 1)
Pred. CsrA Transl. Effect
activate
repress

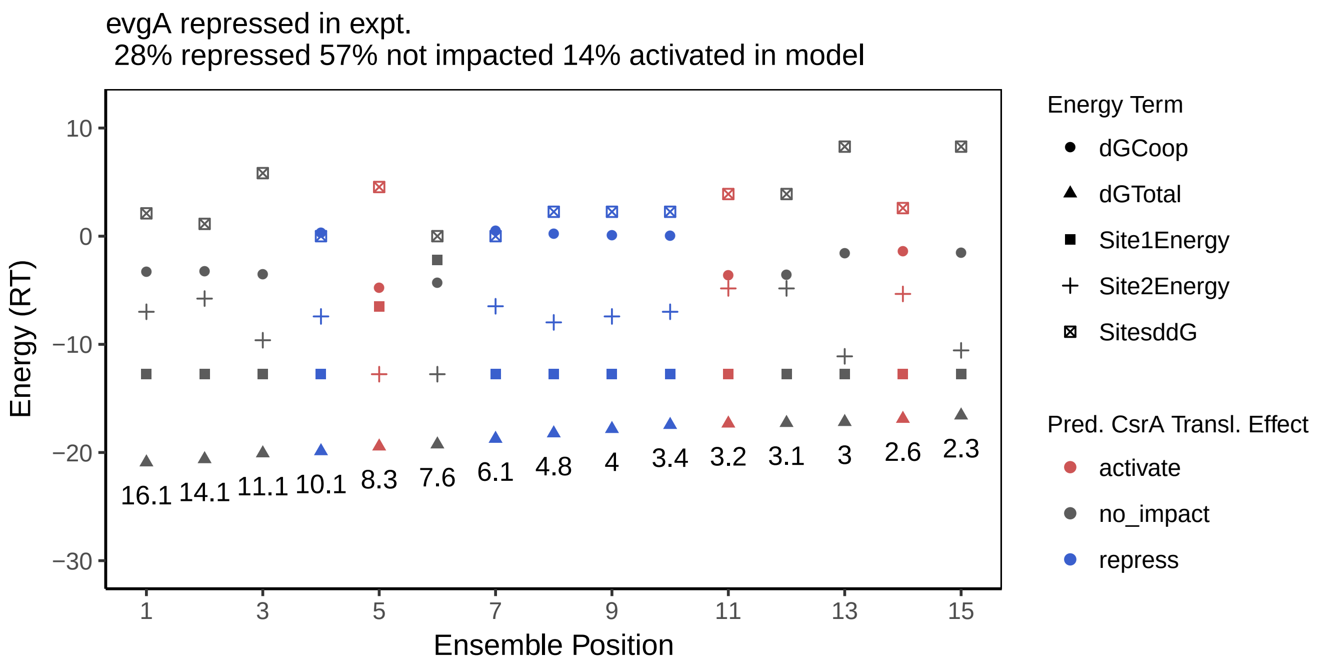
evgA repressed in expt.
28% repressed 57% not impacted 14% activated in model
Energy (RT)
Ensemble Position
16.1 14.1 11.1 10.1 8.3 7.6 6.1 4.8 4 3.4 3.2 3.1 3 2.6 2.3
Energy Term
dGCoop
dGTotal
Site1Energy
Site2Energy
SitesddG
Pred. CsrA Transl. Effect
activate
no_impact
repress

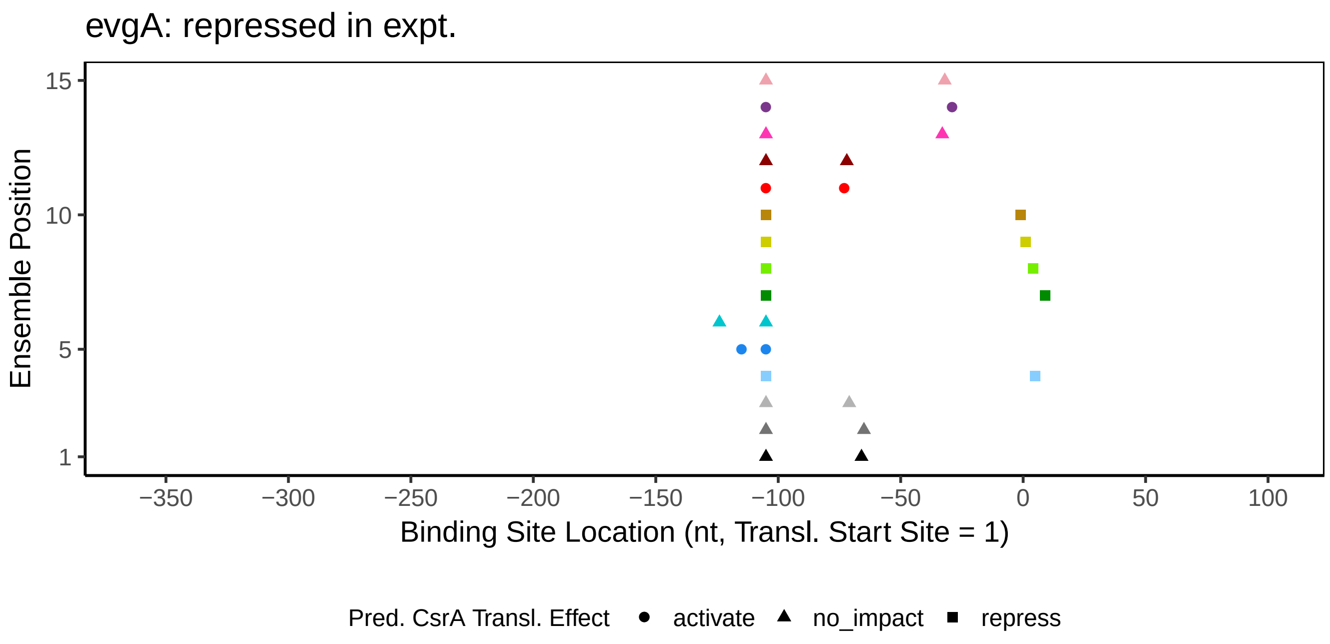
evgA: repressed in expt.
Ensemble Position
Binding Site Location (nt, Transl. Start Site = 1)
Pred. CsrA Transl. Effect
activate
no_impact
repress

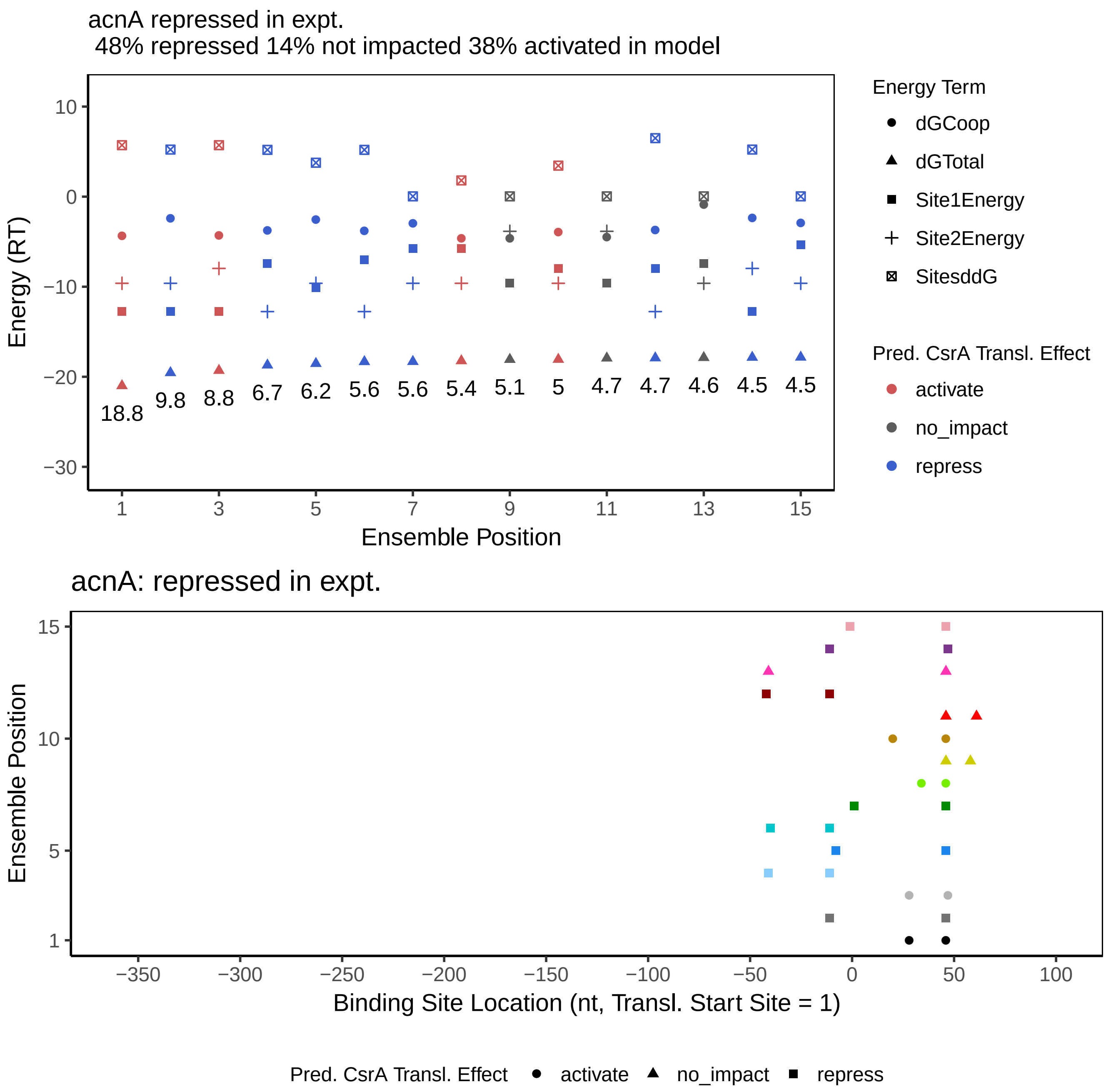
acnA repressed in expt.
48% repressed 14% not impacted 38% activated in model
Energy (RT)
18.8
9.8
8.8
6.7
6.2
5.6
5.6
5.4
5.1
5
4.7
4.7
4.6
4.5
4.5
Ensemble Position
Energy Term
dGCoop
dGTotal
Site1Energy
Site2Energy
SitesddG
Pred. CsrA Transl. Effect
activate
no_impact
repress
acnA: repressed in expt.
Ensemble Position
Binding Site Location (nt, Transl. Start Site = 1)
Pred. CsrA Transl. Effect
activate
no_impact
repress

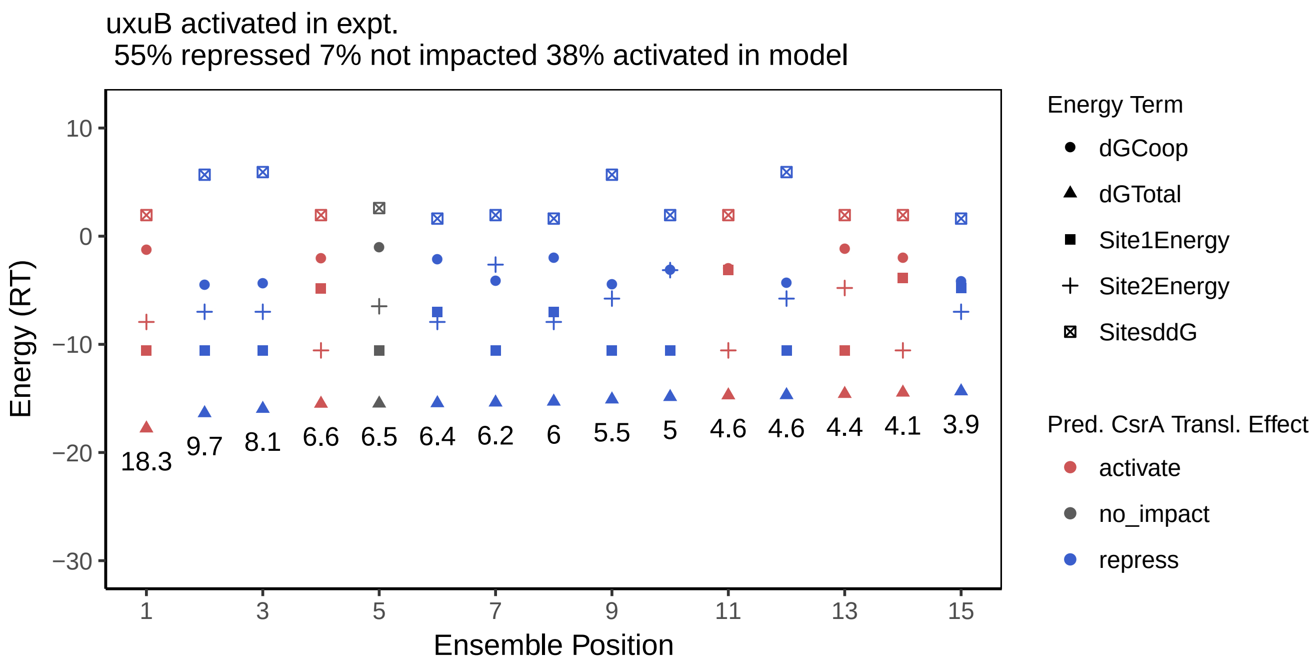
uxuB activated in expt.
55% repressed 7% not impacted 38% activated in model
Energy (RT)
Ensemble Position
18.3
9.7
8.1
6.6
6.5
6.4
6.2
6
5.5
5
4.6
4.6
4.4
4.1
3.9
Energy Term
dGCoop
dGTotal
Site1Energy
Site2Energy
SitesddG
Pred. CsrA Transl. Effect
activate
no_impact
repress

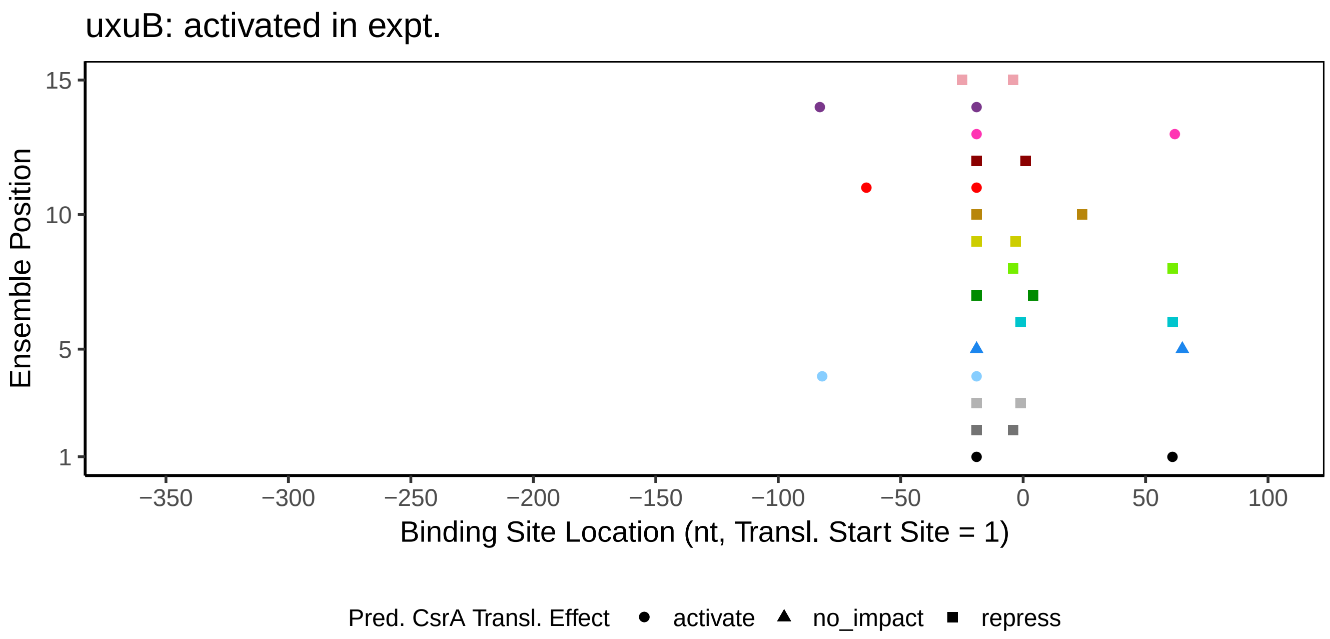
uxuB: activated in expt.
Ensemble Position
Binding Site Location (nt, Transl. Start Site = 1)
Pred. CsrA Transl. Effect
activate
no_impact
repress

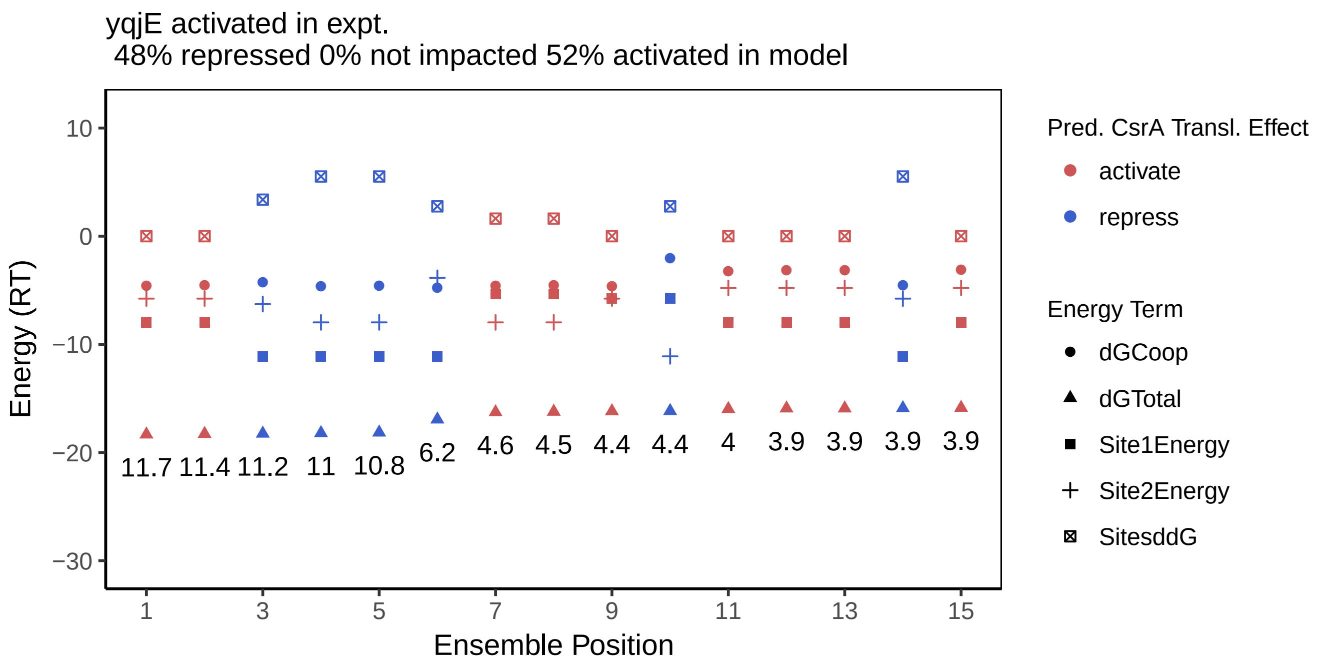

yqjE activated in expt.
48% repressed 0% not impacted 52% activated in model
Energy (RT)
10
0
−10
−20
−30
11.7
11.4
11.2
11
10.8
6.2
4.6
4.5
4.4
4.4
4
3.9
3.9
3.9
3.9
1
3
5
7
9
11
13
15
Ensemble Position
Pred. CsrA Transl. Effect
activate
repress
Energy Term
dGCoop
dGTotal
Site1Energy
Site2Energy
SitesddG

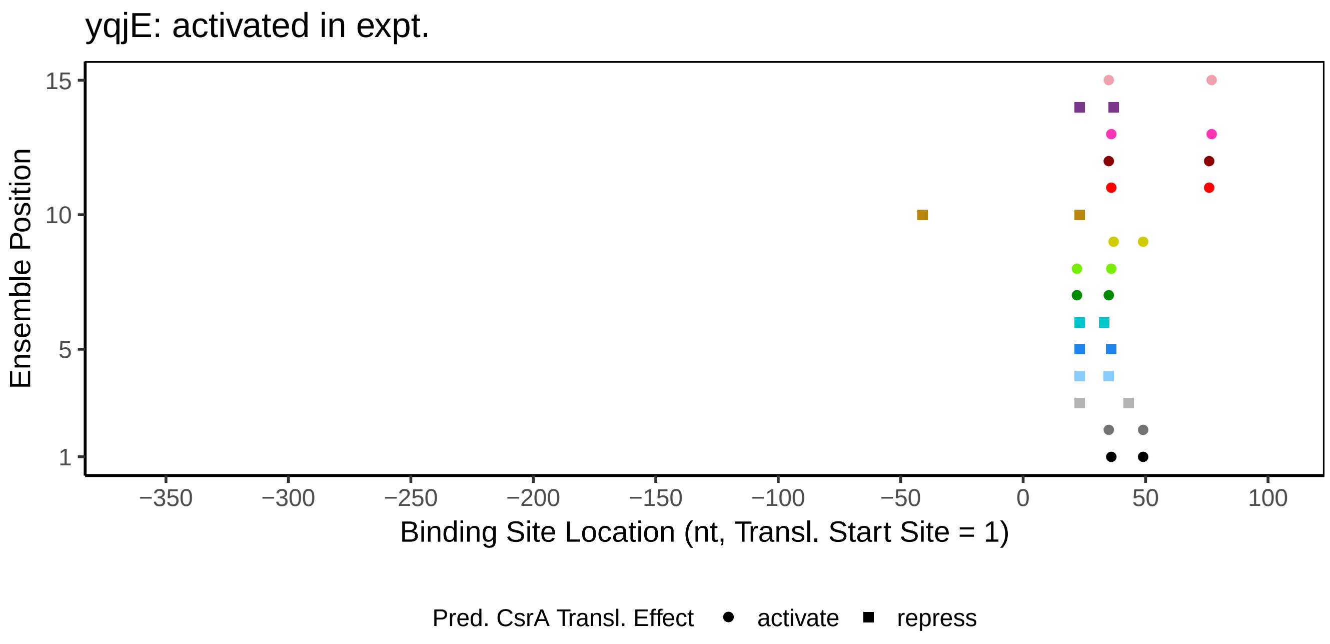

yqjE: activated in expt.
Ensemble Position
15
10
5
1
−350
−300
−250
−200
−150
−100
−50
0
50
100
Binding Site Location (nt, Transl. Start Site = 1)
Pred. CsrA Transl. Effect
activate
repress

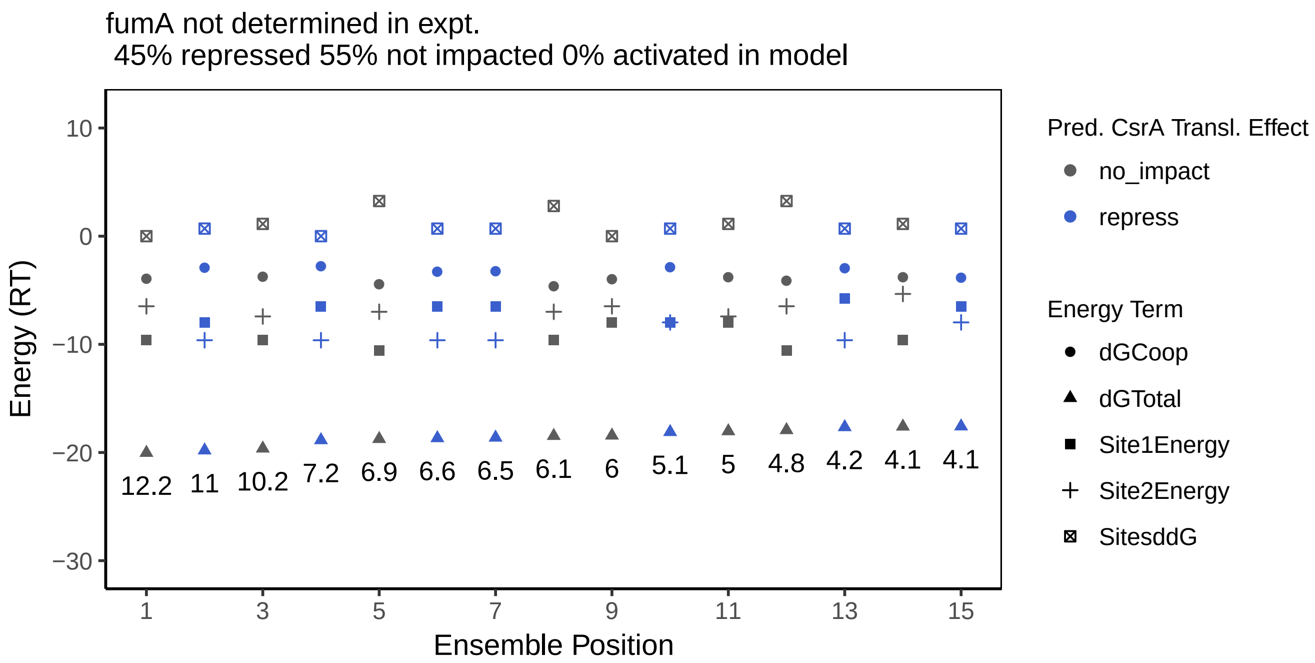

fumA not determined in expt.
45% repressed 55% not impacted 0% activated in model
Energy (RT)
10
0
−10
−20
−30
12.2
11
10.2
7.2
6.9
6.6
6.5
6.1
6
5.1
5
4.8
4.2
4.1
4.1
1
3
5
7
9
11
13
15
Ensemble Position
Pred. CsrA Transl. Effect
no_impact
repress
Energy Term
dGCoop
dGTotal
Site1Energy
Site2Energy
SitesddG

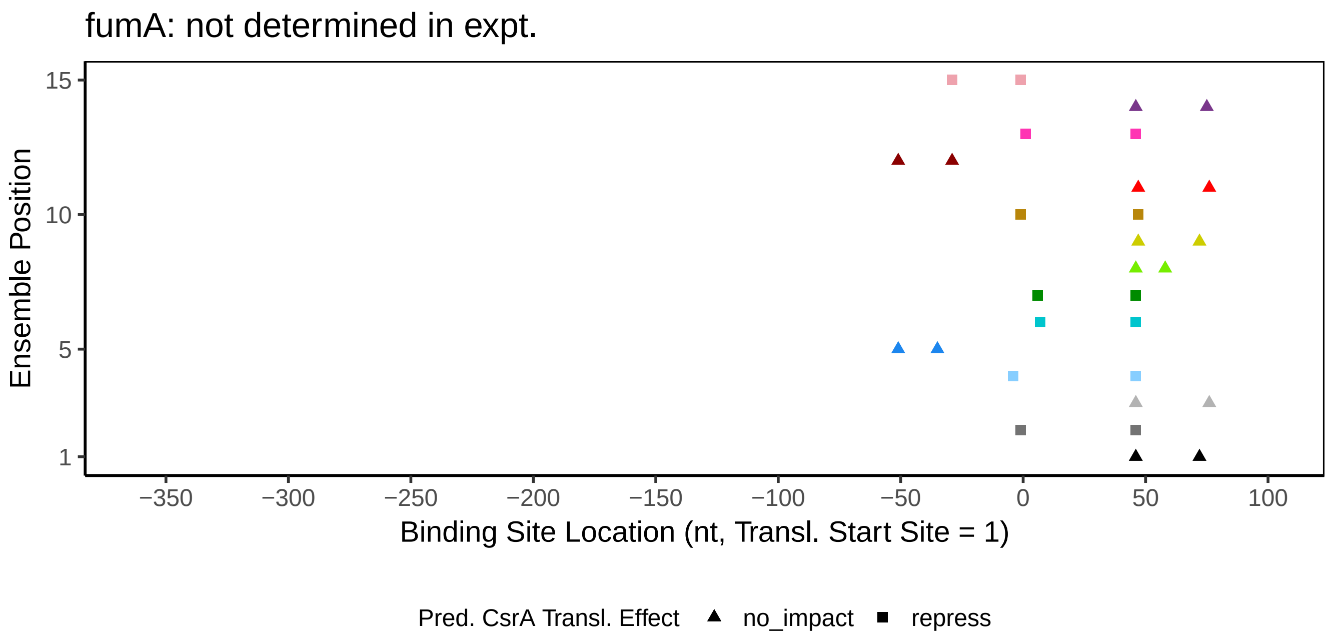

fumA: not determined in expt.
Ensemble Position
15
10
5
1
−350
−300
−250
−200
−150
−100
−50
0
50
100
Binding Site Location (nt, Transl. Start Site = 1)
Pred. CsrA Transl. Effect
no_impact
repress

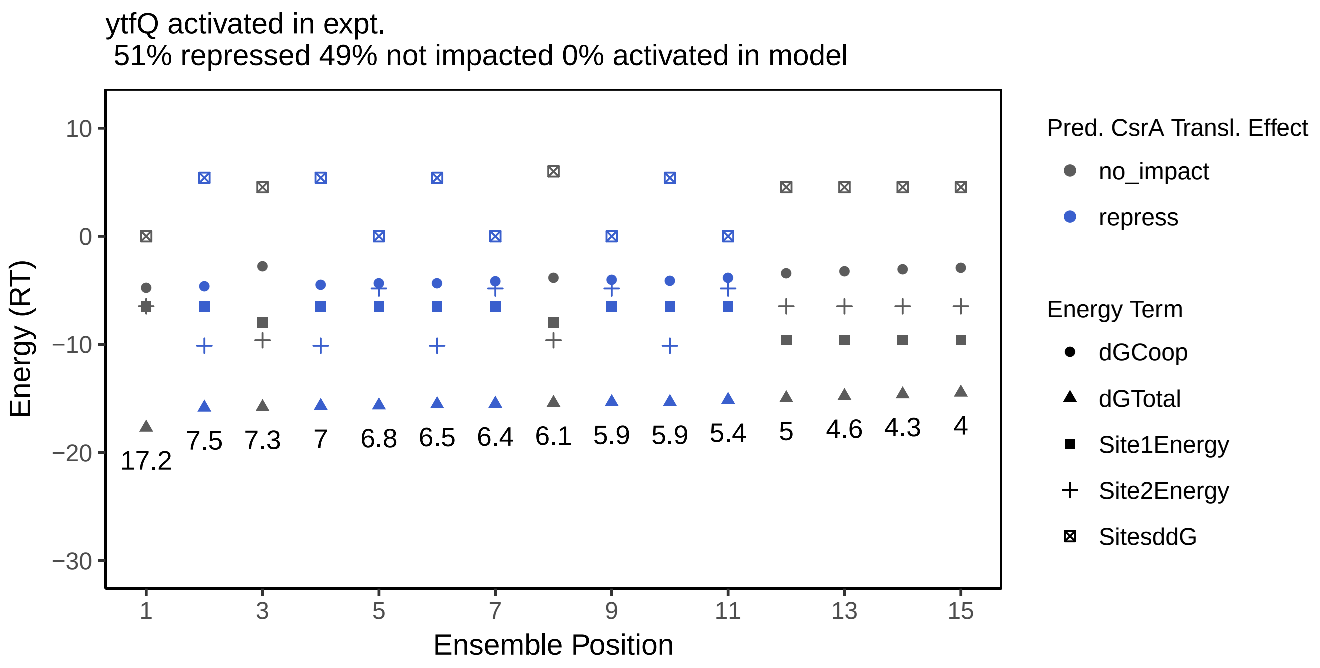
ytfQ activated in expt.
51% repressed 49% not impacted 0% activated in model
Energy (RT)
Ensemble Position
17.2
7.5
7.3
7
6.8
6.5
6.4
6.1
5.9
5.9
5.4
5
4.6
4.3
4
Pred. CsrA Transl. Effect
no_impact
repress
Energy Term
dGCoop
dGTotal
Site1Energy
Site2Energy
SitesddG

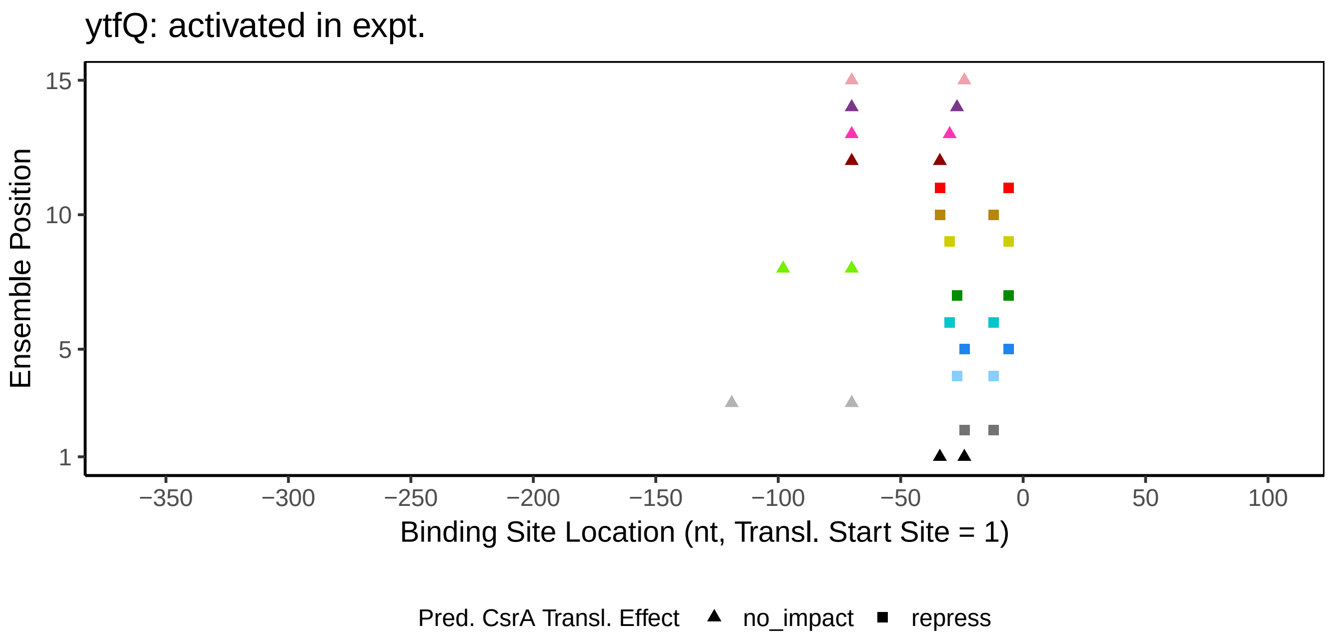
ytfQ: activated in expt.
Ensemble Position
Binding Site Location (nt, Transl. Start Site = 1)
Pred. CsrA Transl. Effect
no_impact
repress

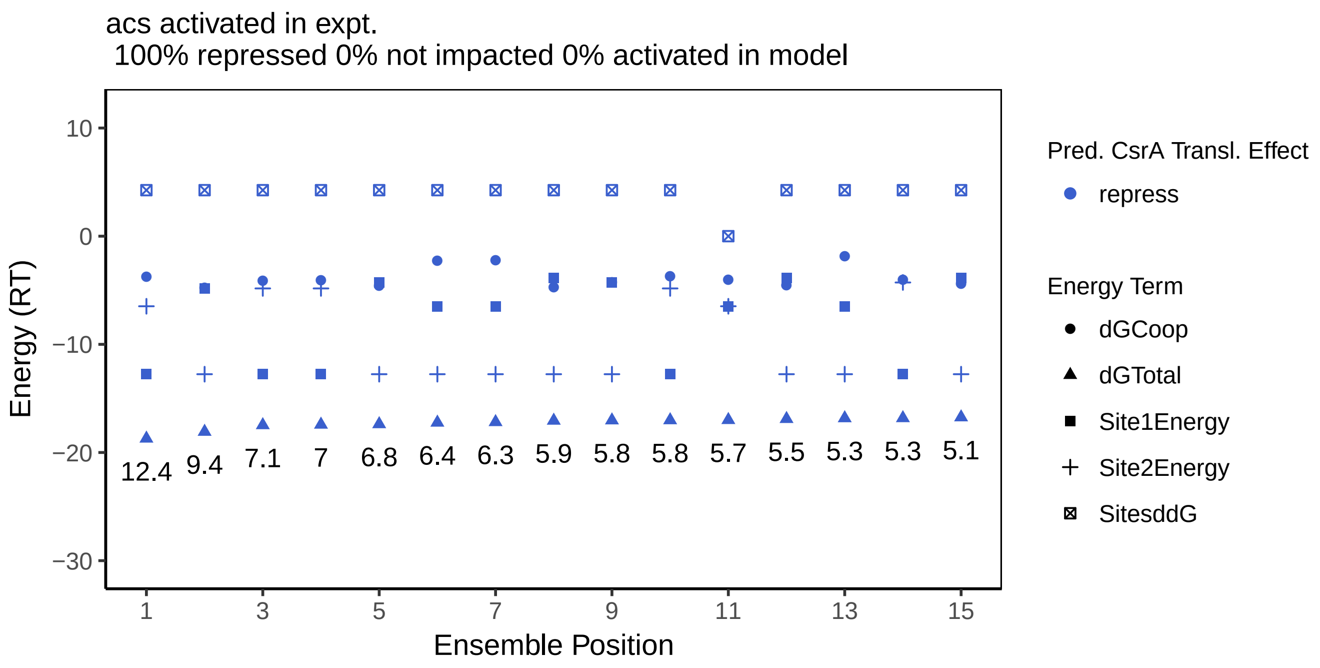
acs activated in expt.
100% repressed 0% not impacted 0% activated in model
Energy (RT)
10
0
−10
−20
−30
12.4
9.4
7.1
7
6.8
6.4
6.3
5.9
5.8
5.8
5.7
5.5
5.3
5.3
5.1
1
3
5
7
9
11
13
15
Ensemble Position
Pred. CsrA Transl. Effect
repress
Energy Term
dGCoop
dGTotal
Site1Energy
Site2Energy
SitesddG

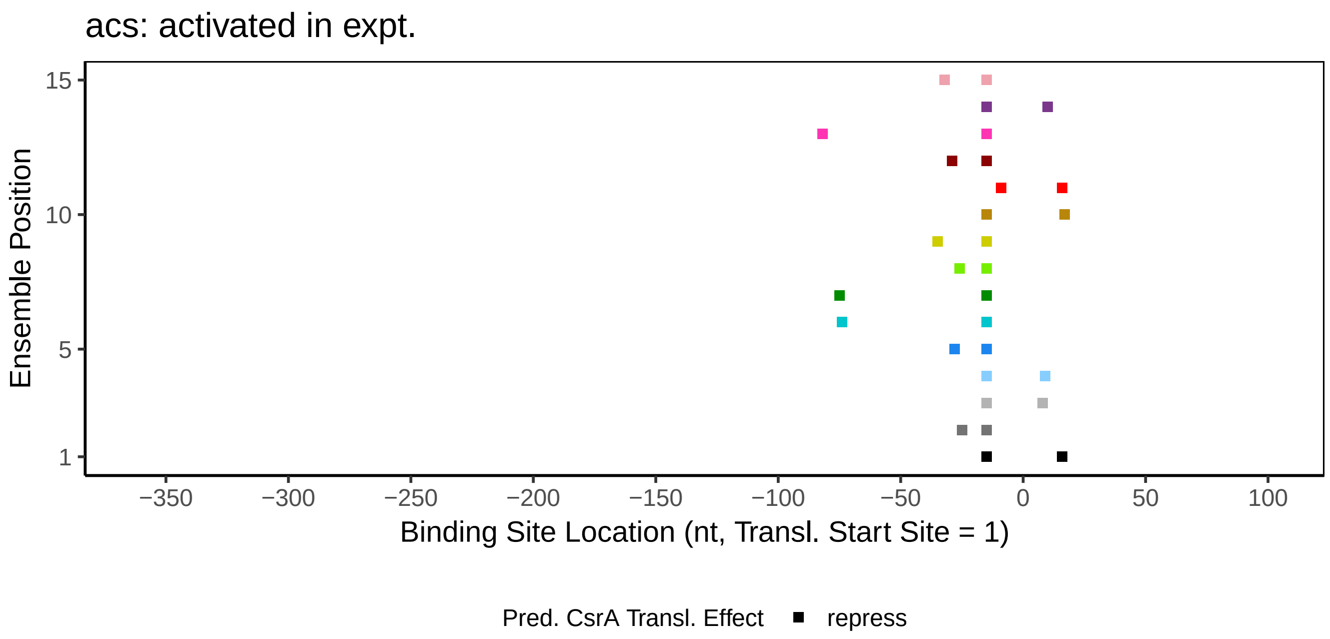
acs: activated in expt.
Ensemble Position
15
10
5
1
−350
−300
−250
−200
−150
−100
−50
0
50
100
Binding Site Location (nt, Transl. Start Site = 1)
Pred. CsrA Transl. Effect
repress

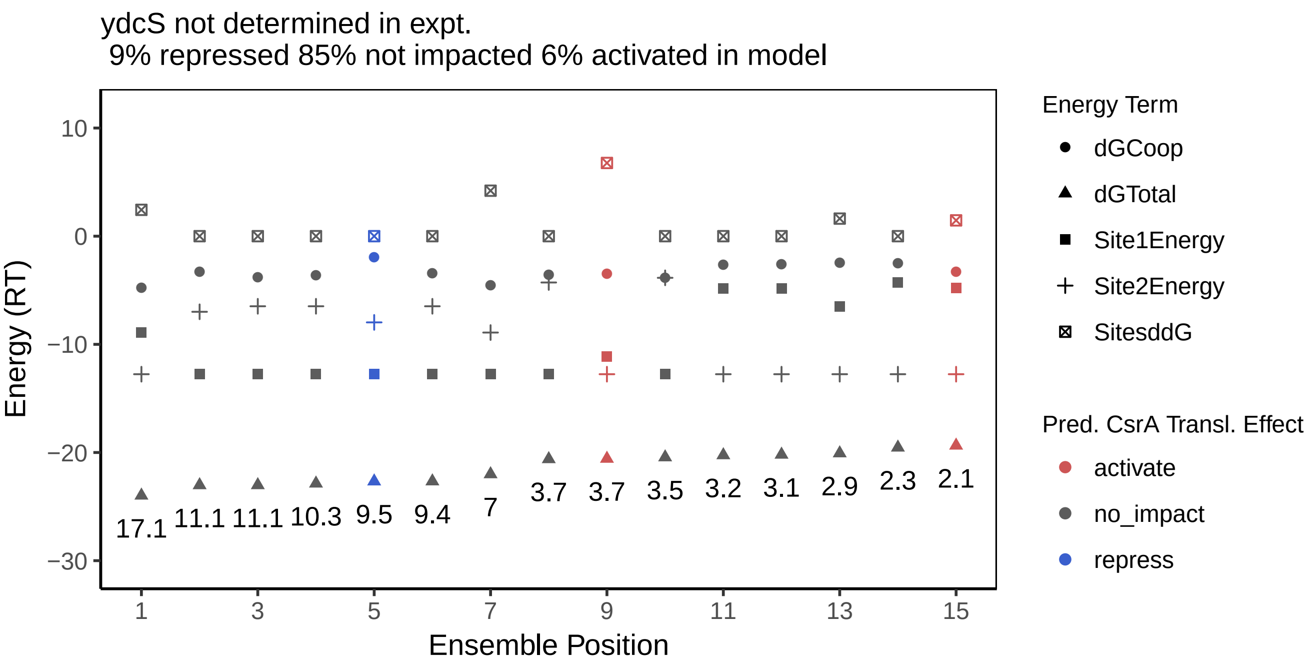

ydcS not determined in expt.
9% repressed 85% not impacted 6% activated in model
Energy (RT)
Ensemble Position
Energy Term
dGCoop
dGTotal
Site1Energy
Site2Energy
SitesddG
Pred. CsrA Transl. Effect
activate
no_impact
repress
17.1
11.1
11.1
10.3
9.5
9.4
7
3.7
3.7
3.5
3.2
3.1
2.9
2.3
2.1

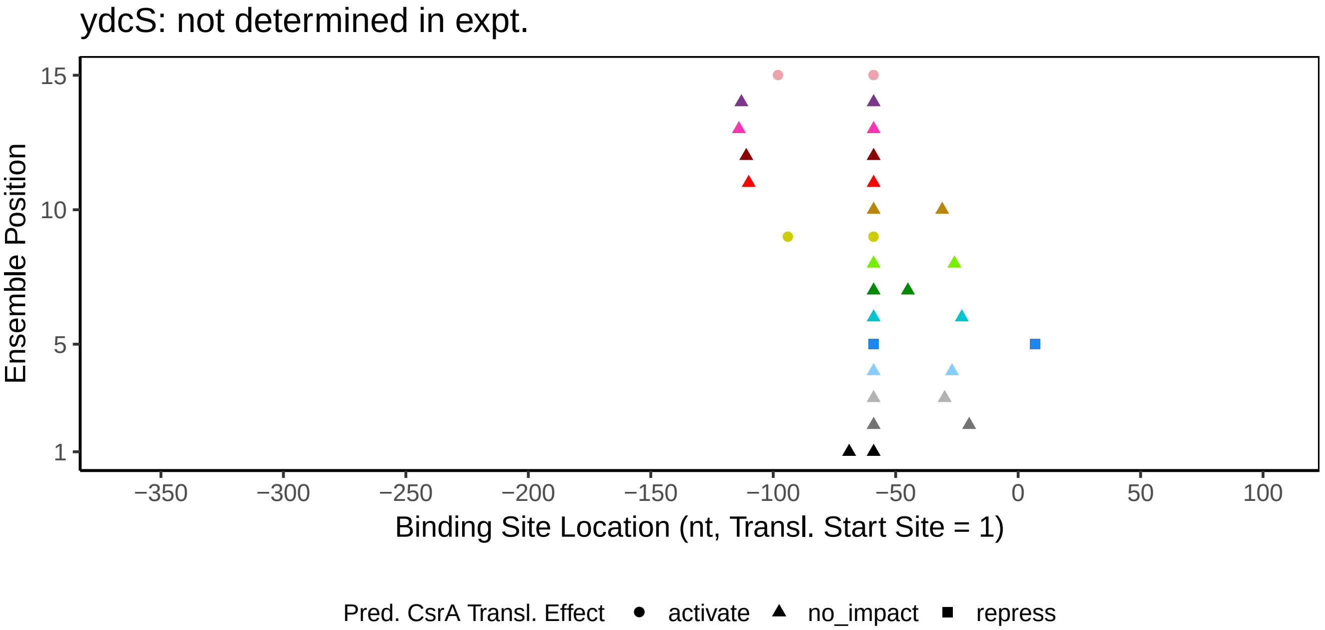

ydcS: not determined in expt.
Ensemble Position
Binding Site Location (nt, Transl. Start Site = 1)
Pred. CsrA Transl. Effect
activate
no_impact
repress

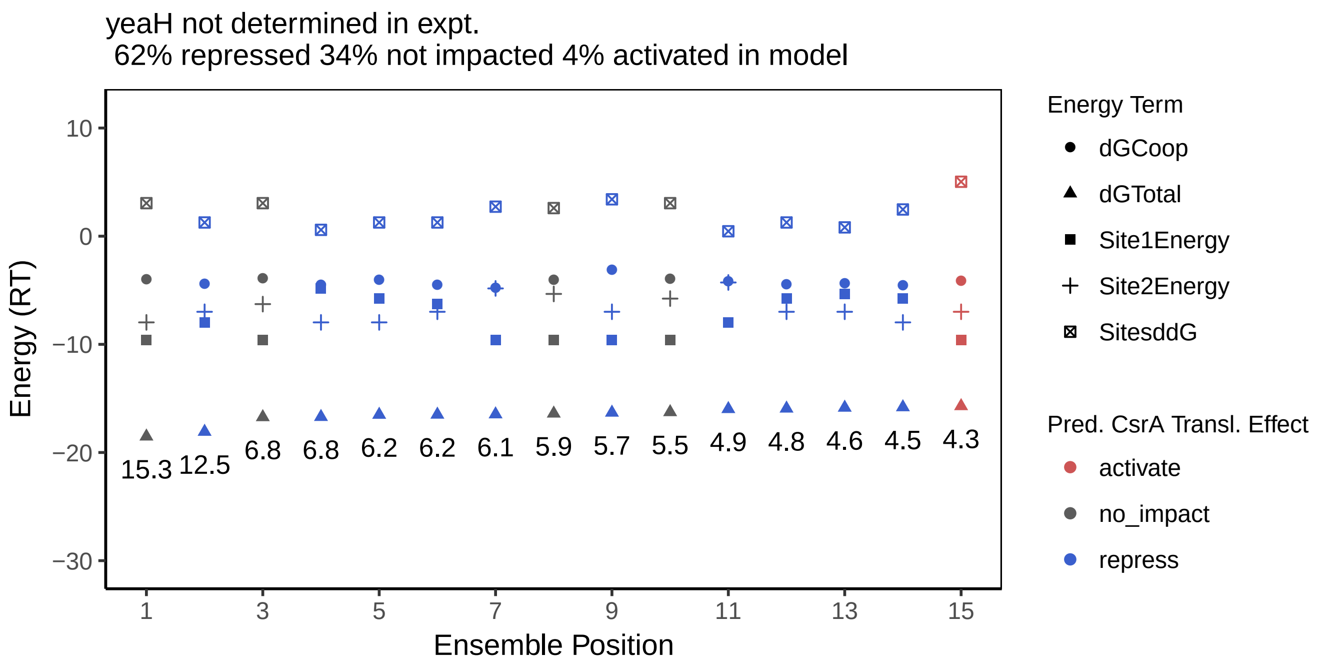

yeaH not determined in expt.
62% repressed 34% not impacted 4% activated in model
Energy (RT)
Ensemble Position
15.3 12.5 6.8 6.8 6.2 6.2 6.1 5.9 5.7 5.5 4.9 4.8 4.6 4.5 4.3
Energy Term
dGCoop
dGTotal
Site1Energy
Site2Energy
SitesddG
Pred. CsrA Transl. Effect
activate
no_impact
repress

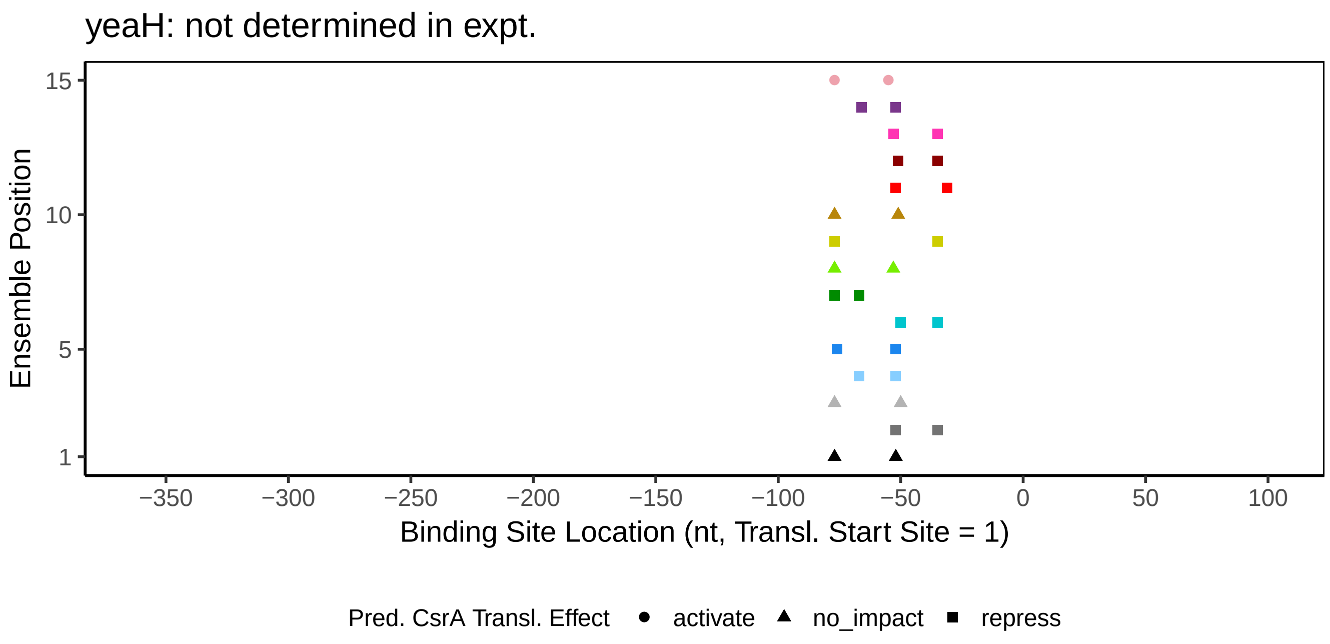

yeaH: not determined in expt.
Ensemble Position
Binding Site Location (nt, Transl. Start Site = 1)
Pred. CsrA Transl. Effect
activate
no_impact
repress

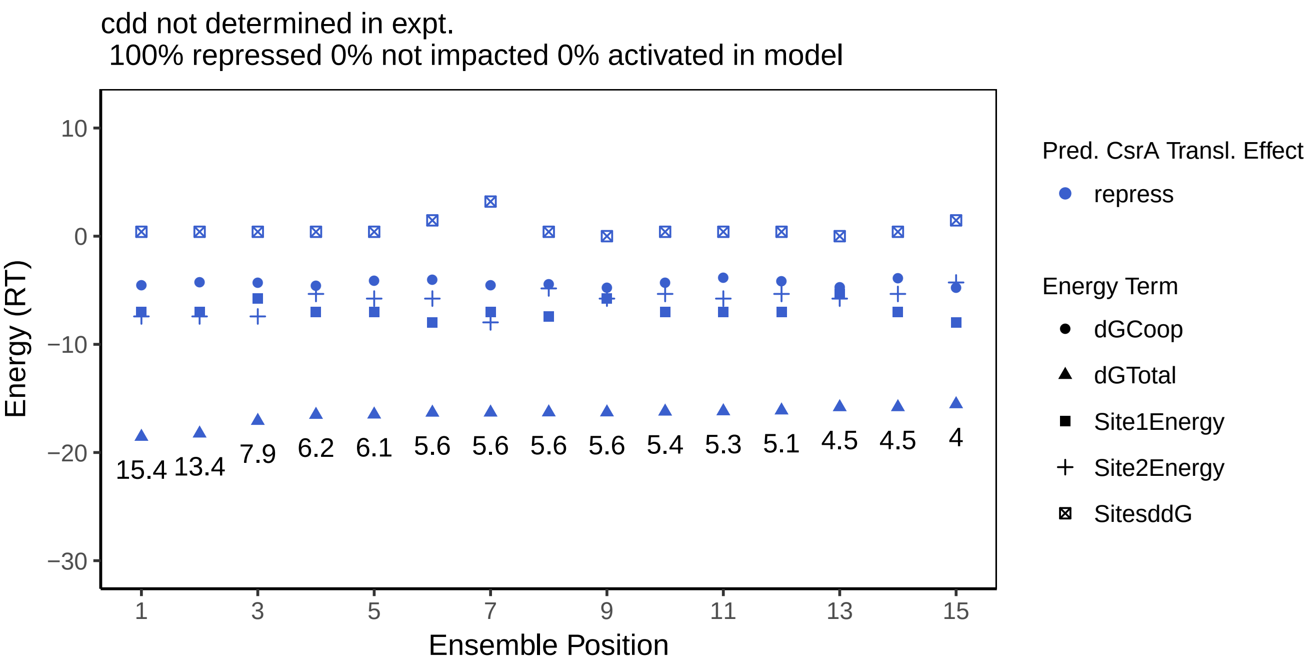

cdd not determined in expt.
100% repressed 0% not impacted 0% activated in model
Energy (RT)
Ensemble Position
15.4 13.4 7.9 6.2 6.1 5.6 5.6 5.6 5.6 5.4 5.3 5.1 4.5 4.5 4
Pred. CsrA Transl. Effect
repress
Energy Term
dGCoop
dGTotal
Site1Energy
Site2Energy
SitesddG

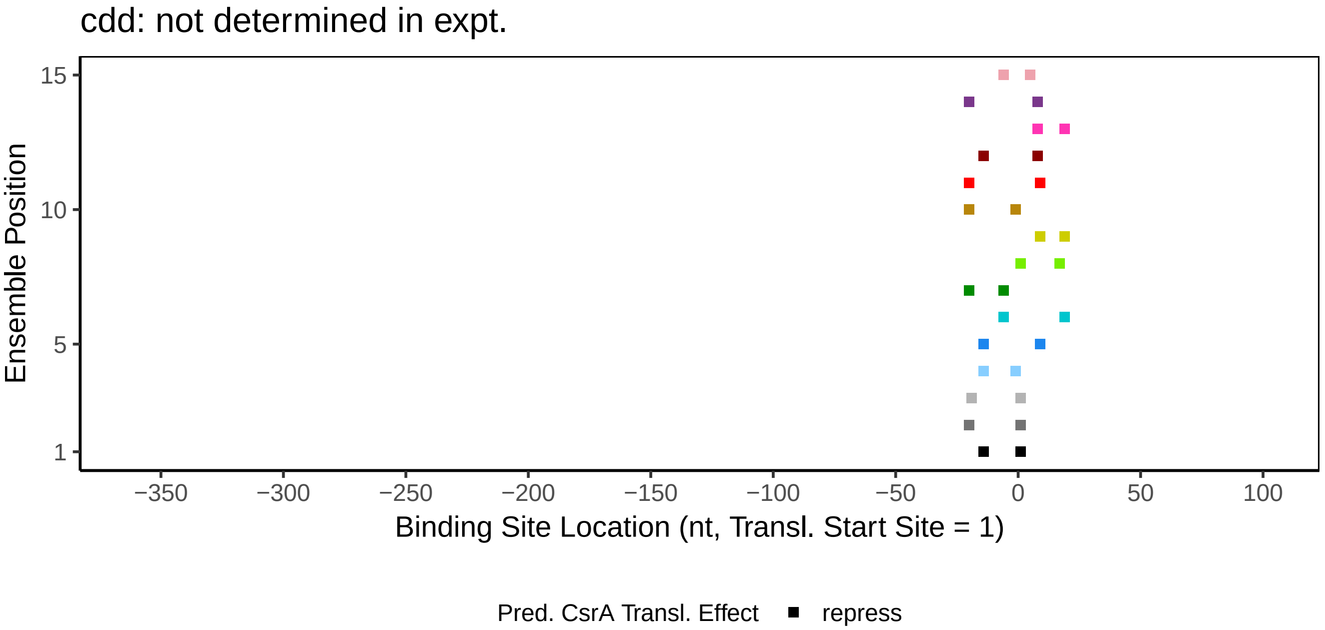

cdd: not determined in expt.
Ensemble Position
Binding Site Location (nt, Transl. Start Site = 1)
Pred. CsrA Transl. Effect
repress

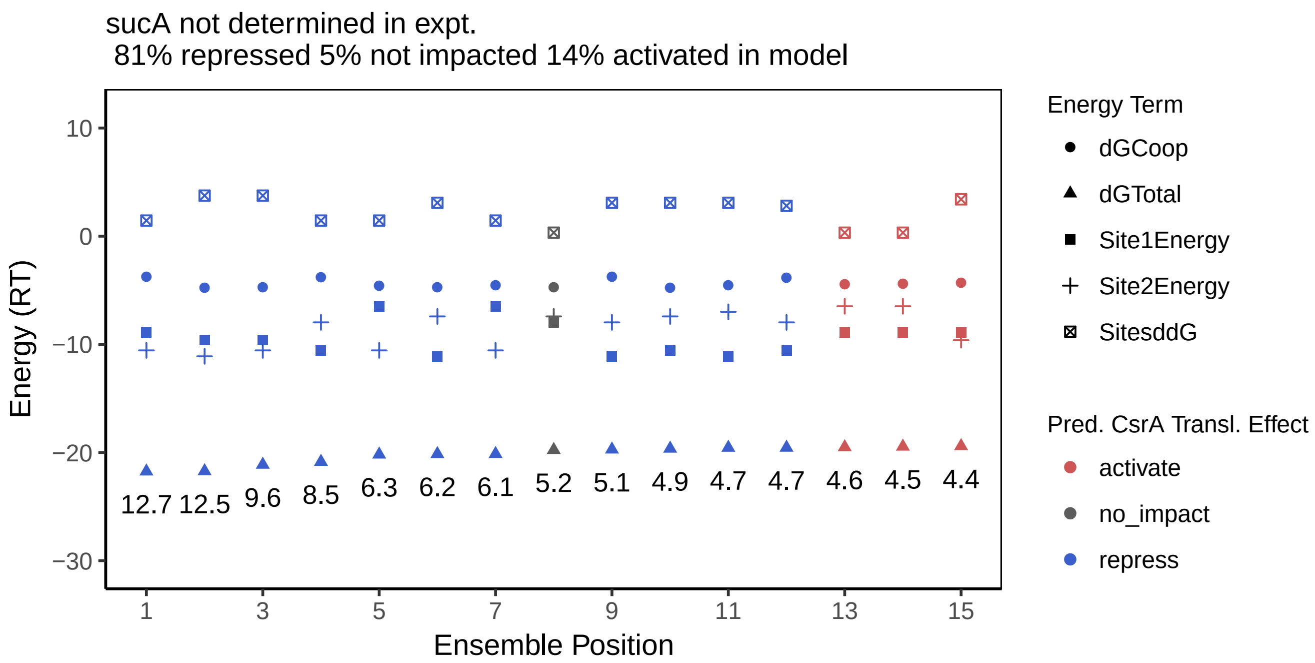
sucA not determined in expt.
81% repressed 5% not impacted 14% activated in model
Energy (RT)
Ensemble Position
12.7 12.5 9.6 8.5 6.3 6.2 6.1 5.2 5.1 4.9 4.7 4.7 4.6 4.5 4.4
Energy Term
dGCoop
dGTotal
Site1Energy
Site2Energy
SitesddG
Pred. CsrA Transl. Effect
activate
no_impact
repress

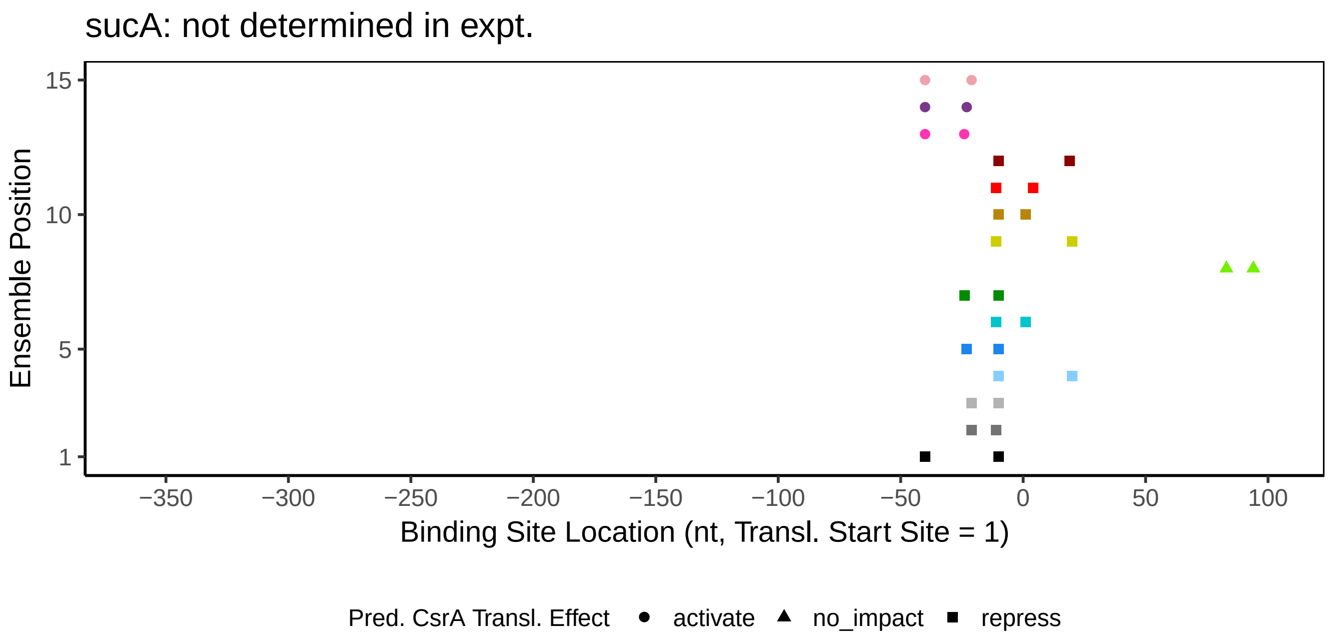
sucA: not determined in expt.
Ensemble Position
Binding Site Location (nt, Transl. Start Site = 1)
Pred. CsrA Transl. Effect
activate
no_impact
repress

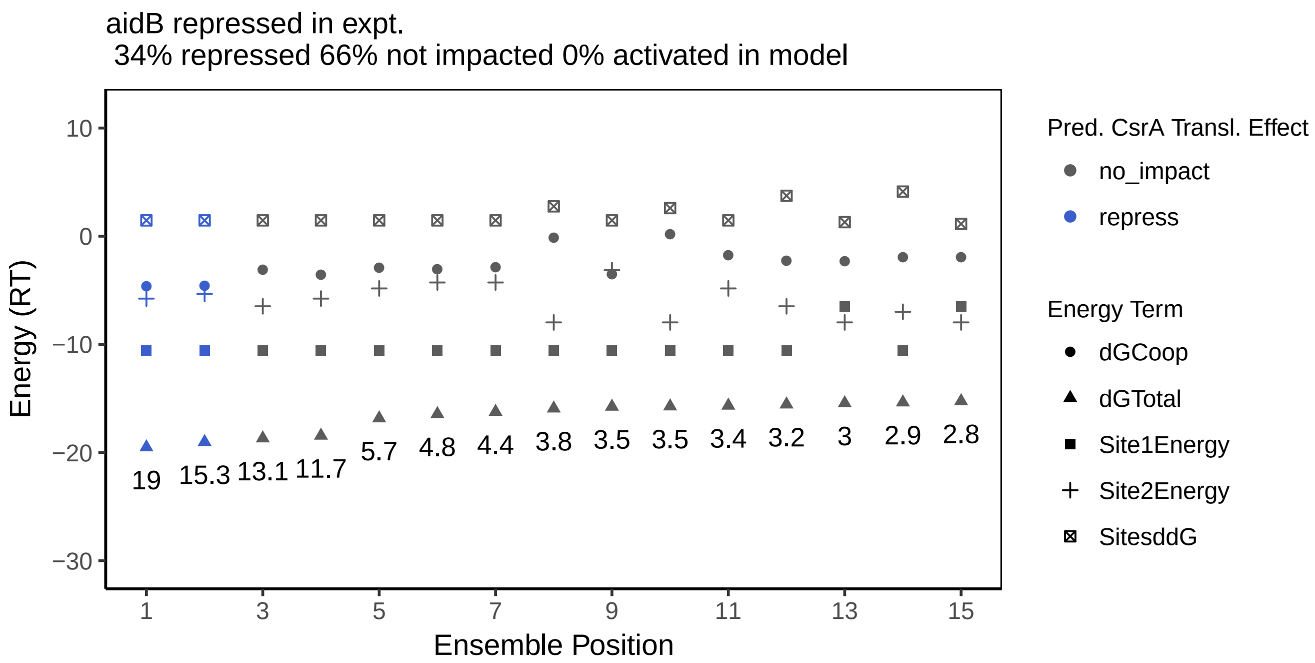
aidB repressed in expt.
34% repressed 66% not impacted 0% activated in model
Energy (RT)
10
0
−10
−20
−30
19
15.3
13.1
11.7
5.7
4.8
4.4
3.8
3.5
3.5
3.4
3.2
3
2.9
2.8
1
3
5
7
9
11
13
15
Ensemble Position
Pred. CsrA Transl. Effect
no_impact
repress
Energy Term
dGCoop
dGTotal
Site1Energy
Site2Energy
SitesddG

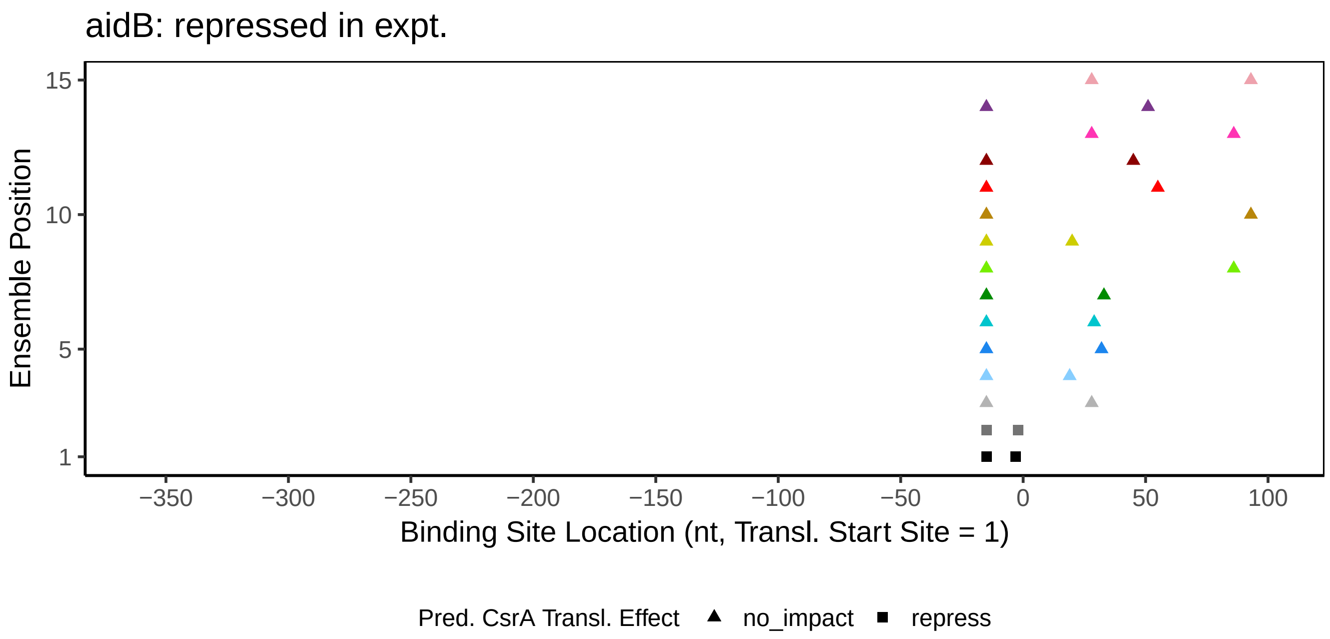
aidB: repressed in expt.
Ensemble Position
15
10
5
1
−350
−300
−250
−200
−150
−100
−50
0
50
100
Binding Site Location (nt, Transl. Start Site = 1)
Pred. CsrA Transl. Effect
no_impact
repress

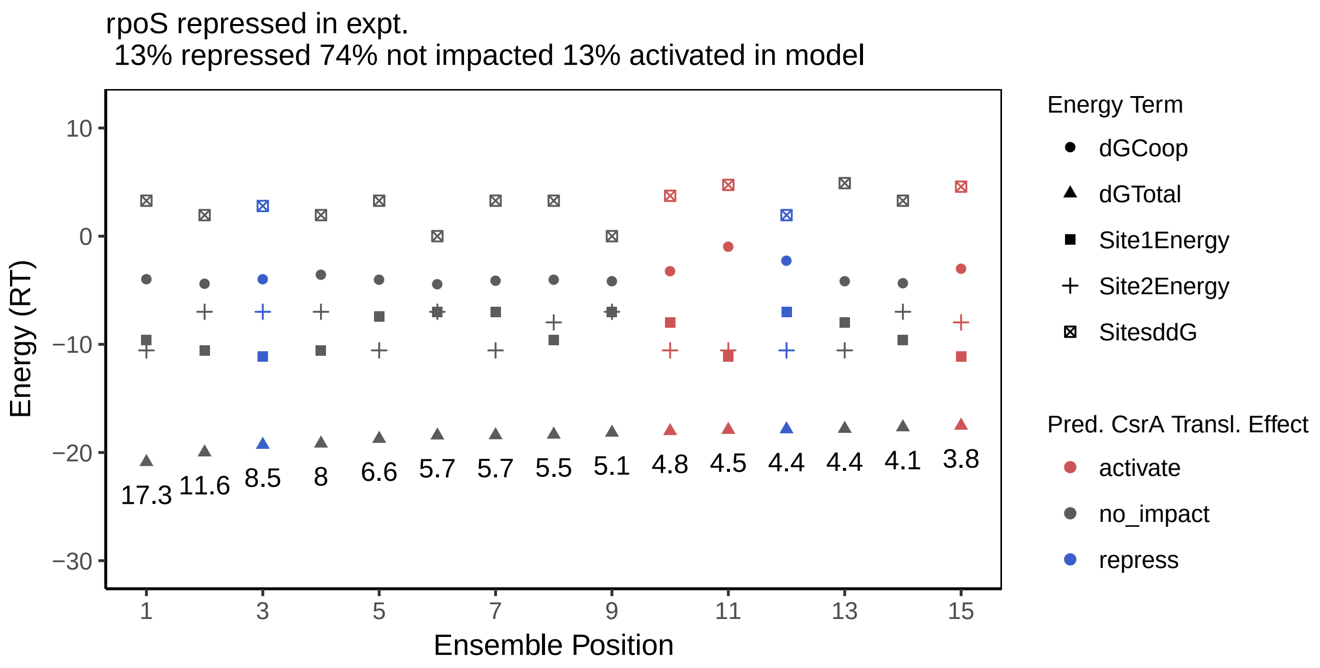

rpoS repressed in expt.
13% repressed 74% not impacted 13% activated in model
Energy (RT)
10
0
−10
−20
−30
17.3
11.6
8.5
8
6.6
5.7
5.7
5.5
5.1
4.8
4.5
4.4
4.4
4.1
3.8
1
3
5
7
9
11
13
15
Ensemble Position
Energy Term
dGCoop
dGTotal
Site1Energy
Site2Energy
SitesddG
Pred. CsrA Transl. Effect
activate
no_impact
repress

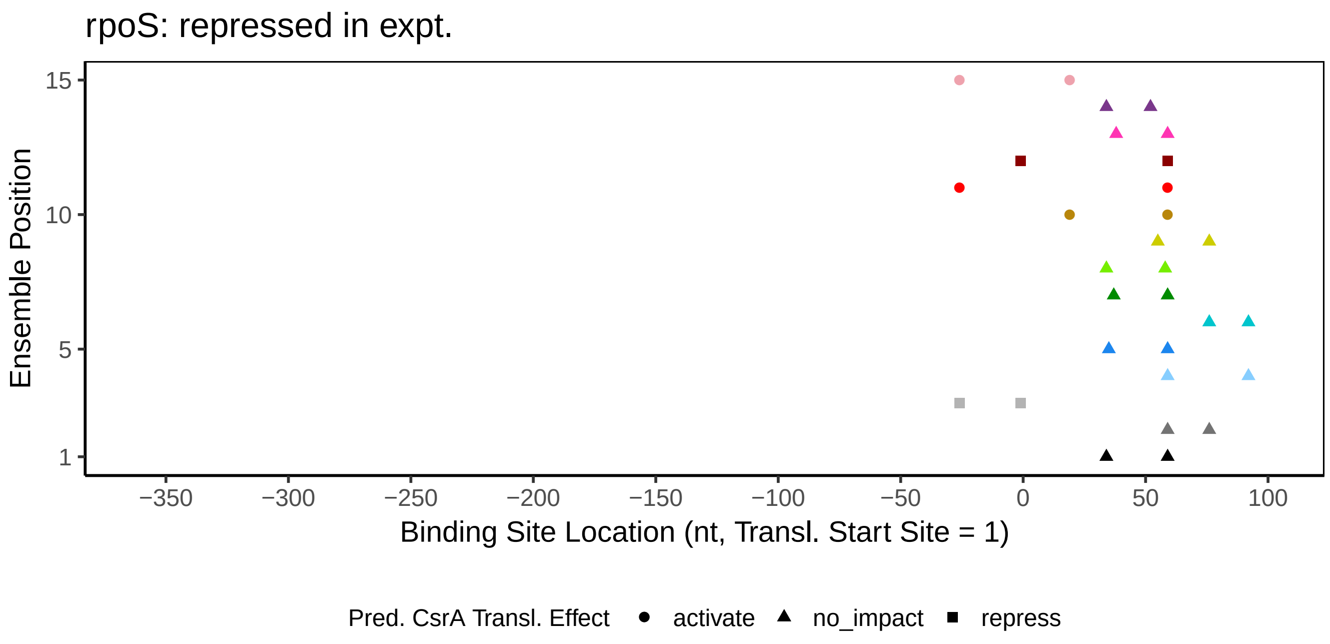

rpoS: repressed in expt.
Ensemble Position
15
10
5
1
−350
−300
−250
−200
−150
−100
−50
0
50
100
Binding Site Location (nt, Transl. Start Site = 1)
Pred. CsrA Transl. Effect
activate
no_impact
repress

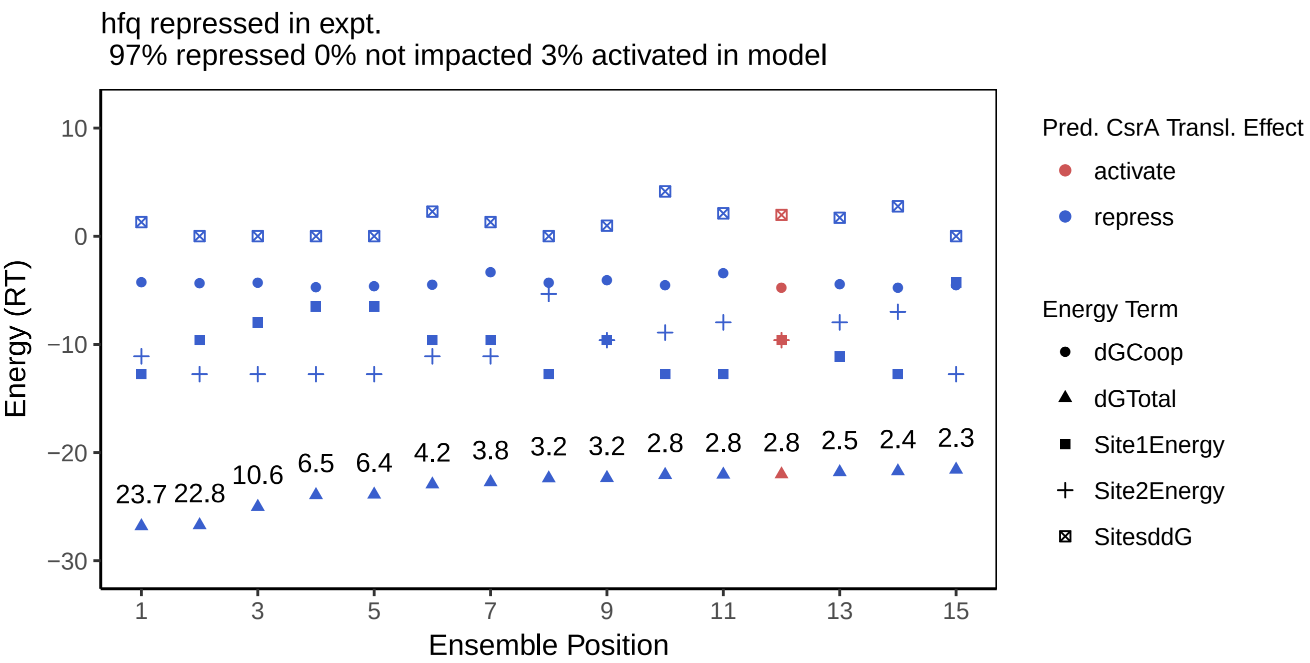
hfq repressed in expt.
97% repressed 0% not impacted 3% activated in model
Energy (RT)
Ensemble Position
23.7 22.8 10.6 6.5 6.4 4.2 3.8 3.2 3.2 2.8 2.8 2.8 2.5 2.4 2.3
Pred. CsrA Transl. Effect
activate
repress
Energy Term
dGCoop
dGTotal
Site1Energy
Site2Energy
SitesddG

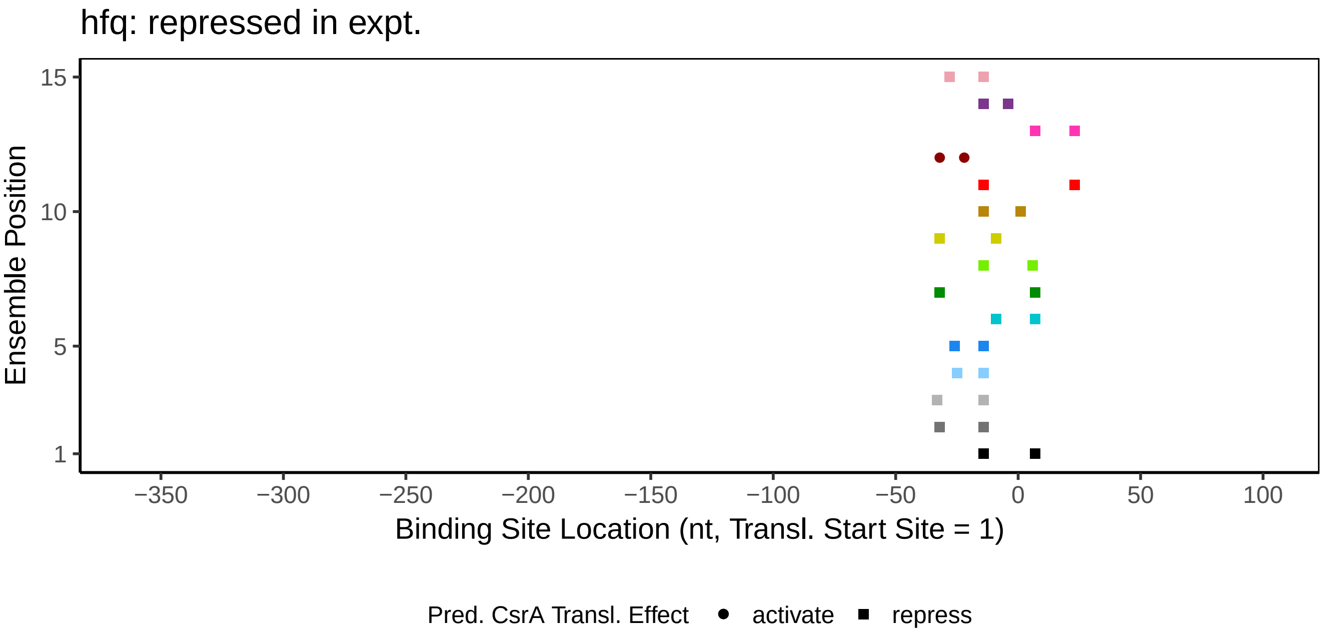
hfq: repressed in expt.
Ensemble Position
Binding Site Location (nt, Transl. Start Site = 1)
Pred. CsrA Transl. Effect
activate
repress

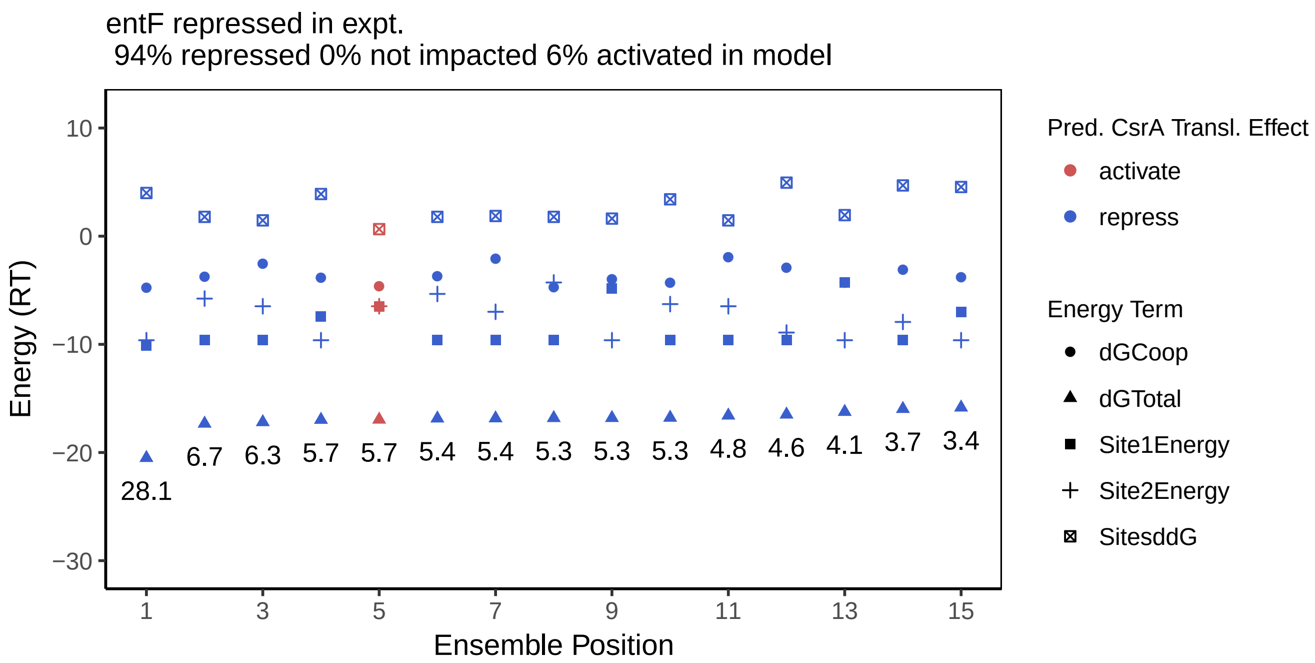
entF repressed in expt.
94% repressed 0% not impacted 6% activated in model
Energy (RT)
10
0
−10
−20
−30
28.1
6.7
6.3
5.7
5.7
5.4
5.4
5.3
5.3
5.3
4.8
4.6
4.1
3.7
3.4
1
3
5
7
9
11
13
15
Ensemble Position
Pred. CsrA Transl. Effect
activate
repress
Energy Term
dGCoop
dGTotal
Site1Energy
Site2Energy
SitesddG

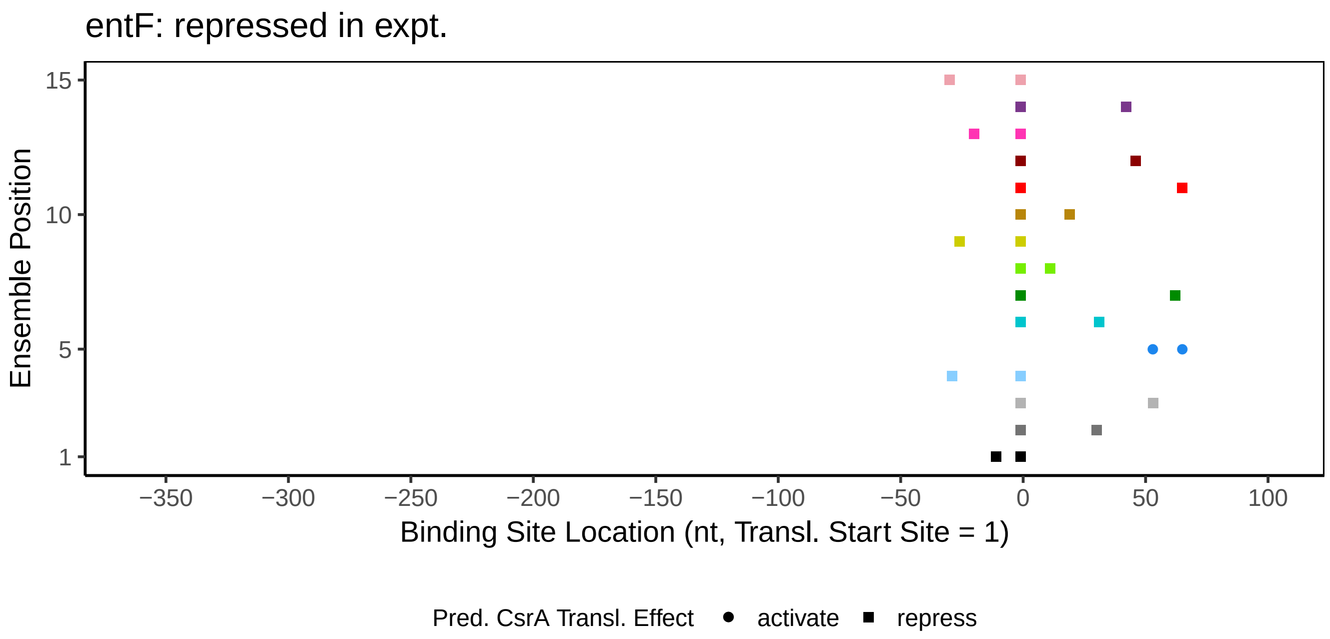
entF: repressed in expt.
Ensemble Position
15
10
5
1
−350
−300
−250
−200
−150
−100
−50
0
50
100
Binding Site Location (nt, Transl. Start Site = 1)
Pred. CsrA Transl. Effect
activate
repress

mscS repressed in expt.
100% repressed 0% not impacted 0% activated in model

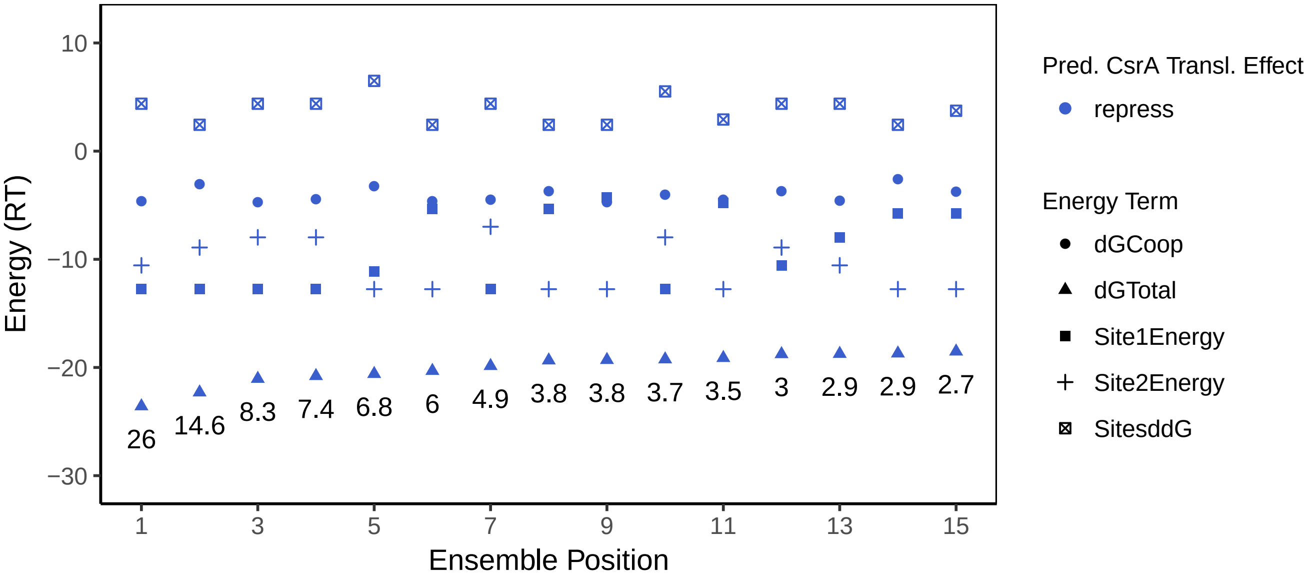

mscS: repressed in expt.

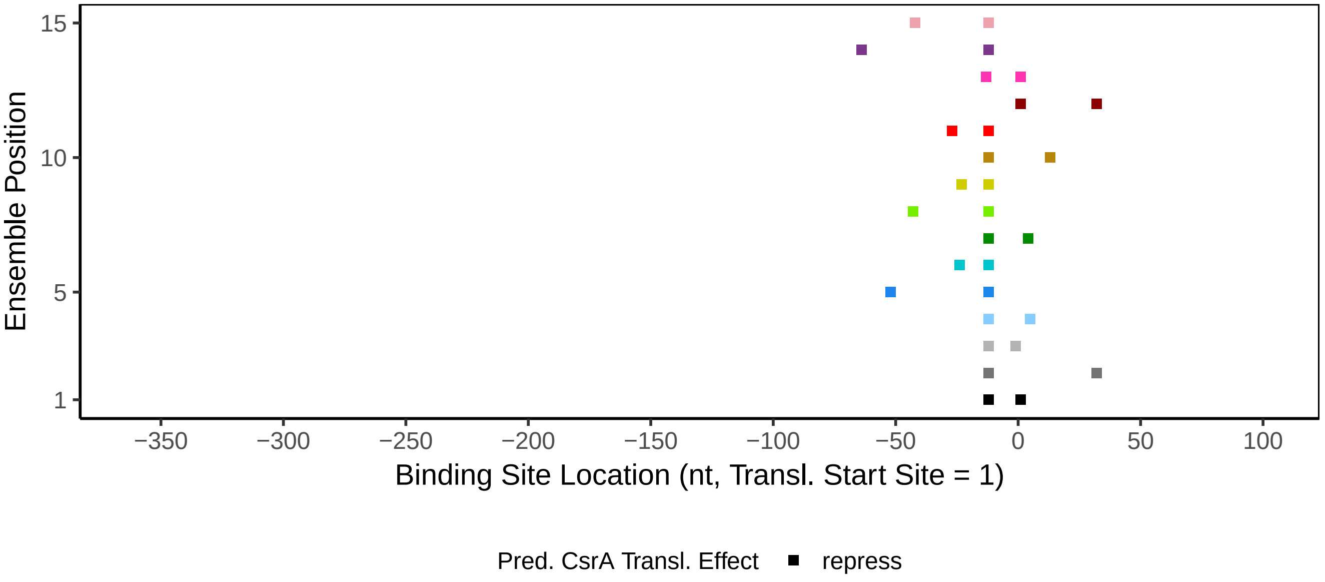

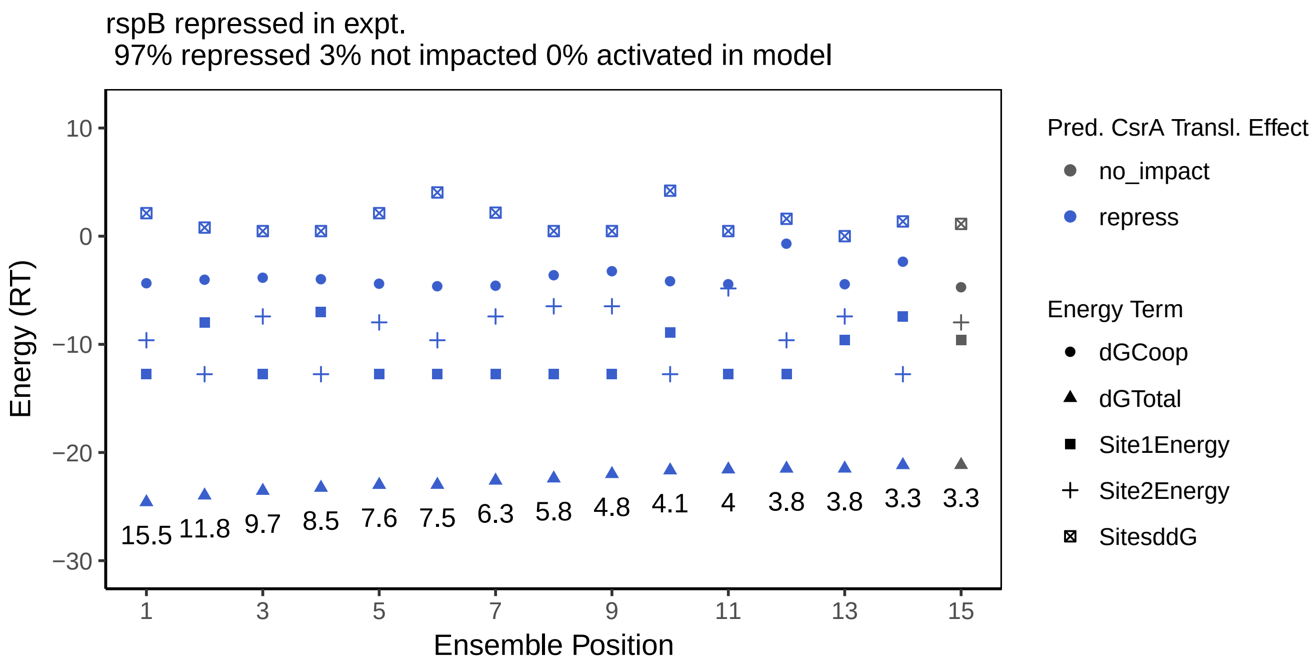
rspB repressed in expt.
97% repressed 3% not impacted 0% activated in model
Energy (RT)
10
0
−10
−20
−30
15.5
11.8
9.7
8.5
7.6
7.5
6.3
5.8
4.8
4.1
4
3.8
3.8
3.3
3.3
1
3
5
7
9
11
13
15
Ensemble Position
Pred. CsrA Transl. Effect
no_impact
repress
Energy Term
dGCoop
dGTotal
Site1Energy
Site2Energy
SitesddG

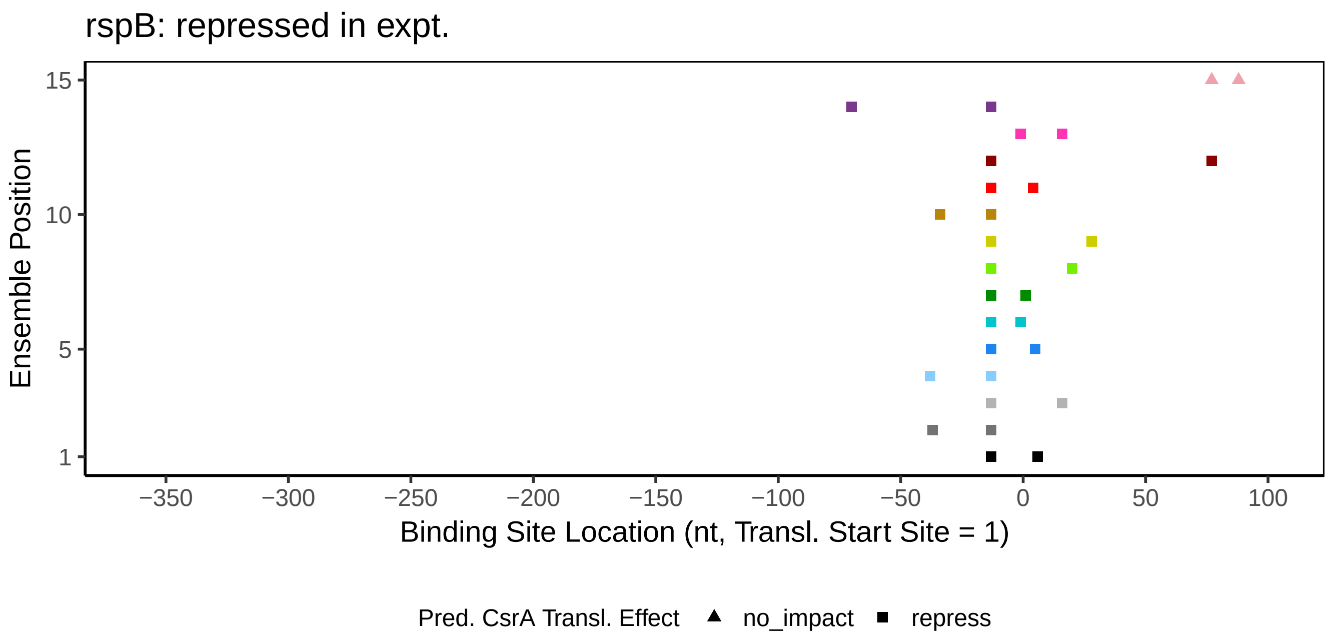
rspB: repressed in expt.
Ensemble Position
15
10
5
1
−350
−300
−250
−200
−150
−100
−50
0
50
100
Binding Site Location (nt, Transl. Start Site = 1)
Pred. CsrA Transl. Effect
no_impact
repress

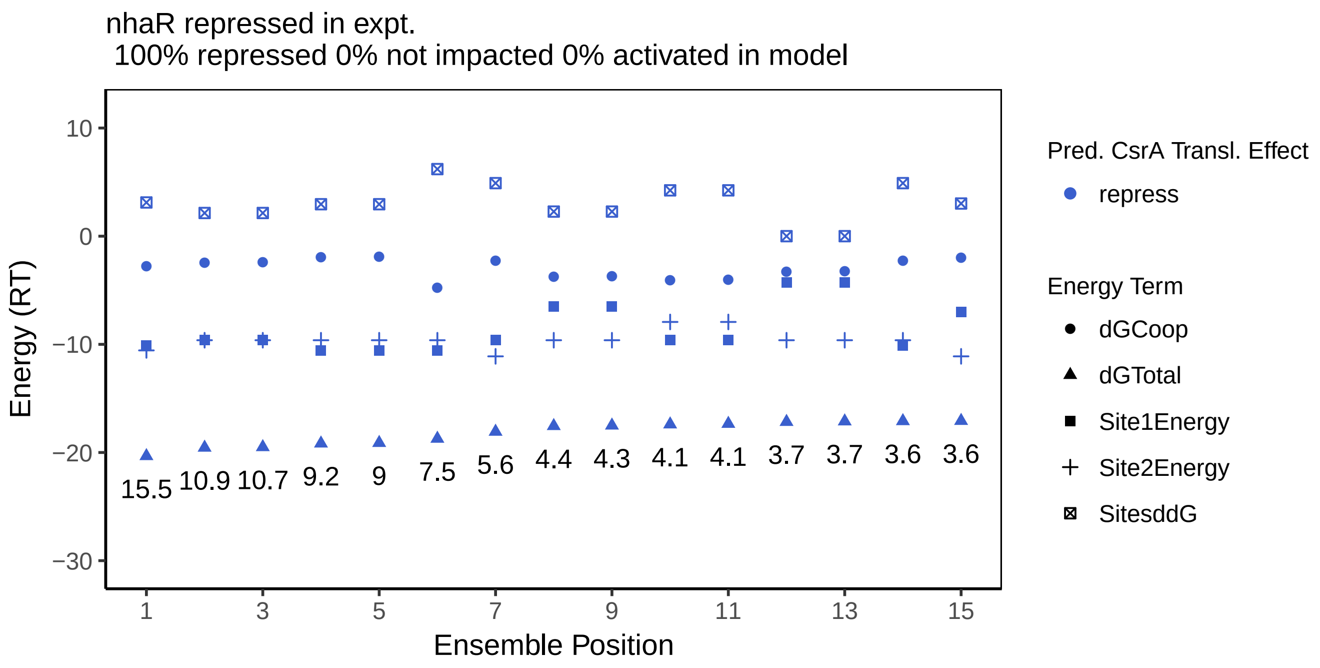
nhaR repressed in expt.
100% repressed 0% not impacted 0% activated in model
Energy (RT)
10
0
−10
−20
−30
15.5 10.9 10.7 9.2 9 7.5 5.6 4.4 4.3 4.1 4.1 3.7 3.7 3.6 3.6
1 3 5 7 9 11 13 15
Ensemble Position
Pred. CsrA Transl. Effect
repress
Energy Term
dGCoop
dGTotal
Site1Energy
Site2Energy
SitesddG

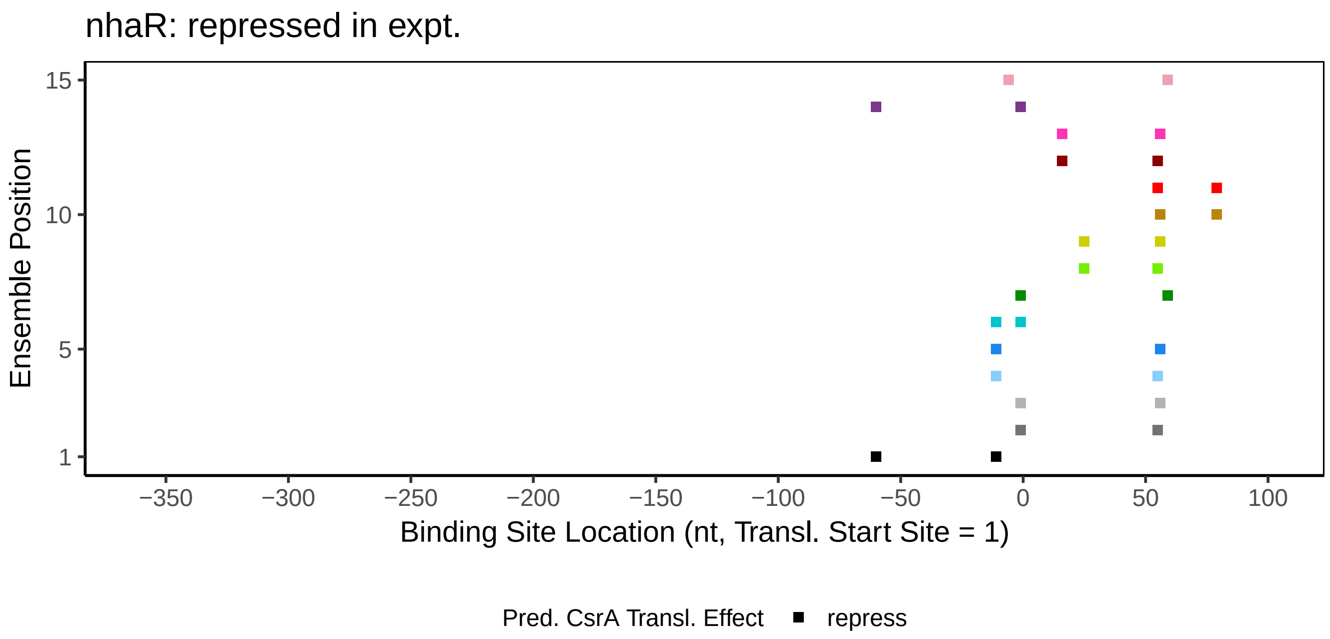
nhaR: repressed in expt.
Ensemble Position
15
10
5
1
−350 −300 −250 −200 −150 −100 −50 0 50 100
Binding Site Location (nt, Transl. Start Site = 1)
Pred. CsrA Transl. Effect
repress

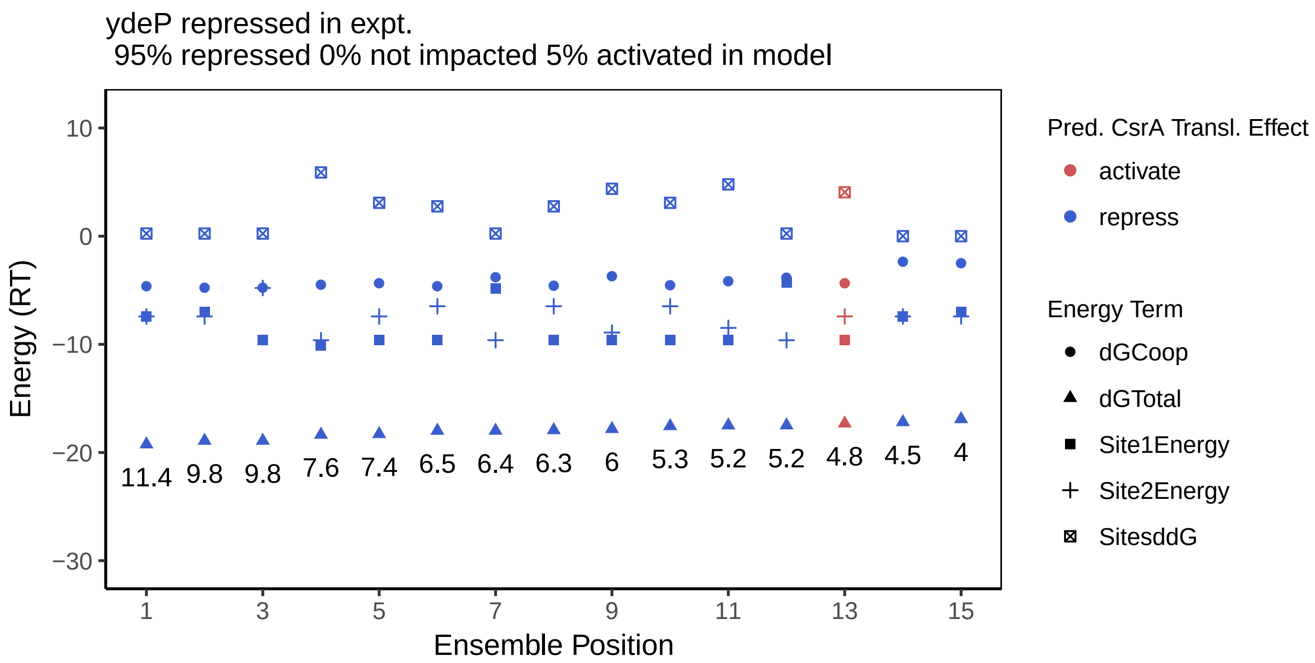
ydeP repressed in expt.
95% repressed 0% not impacted 5% activated in model
Energy (RT)
Ensemble Position
11.4
9.8
9.8
7.6
7.4
6.5
6.4
6.3
6
5.3
5.2
5.2
4.8
4.5
4
Pred. CsrA Transl. Effect
activate
repress
Energy Term
dGCoop
dGTotal
Site1Energy
Site2Energy
SitesddG

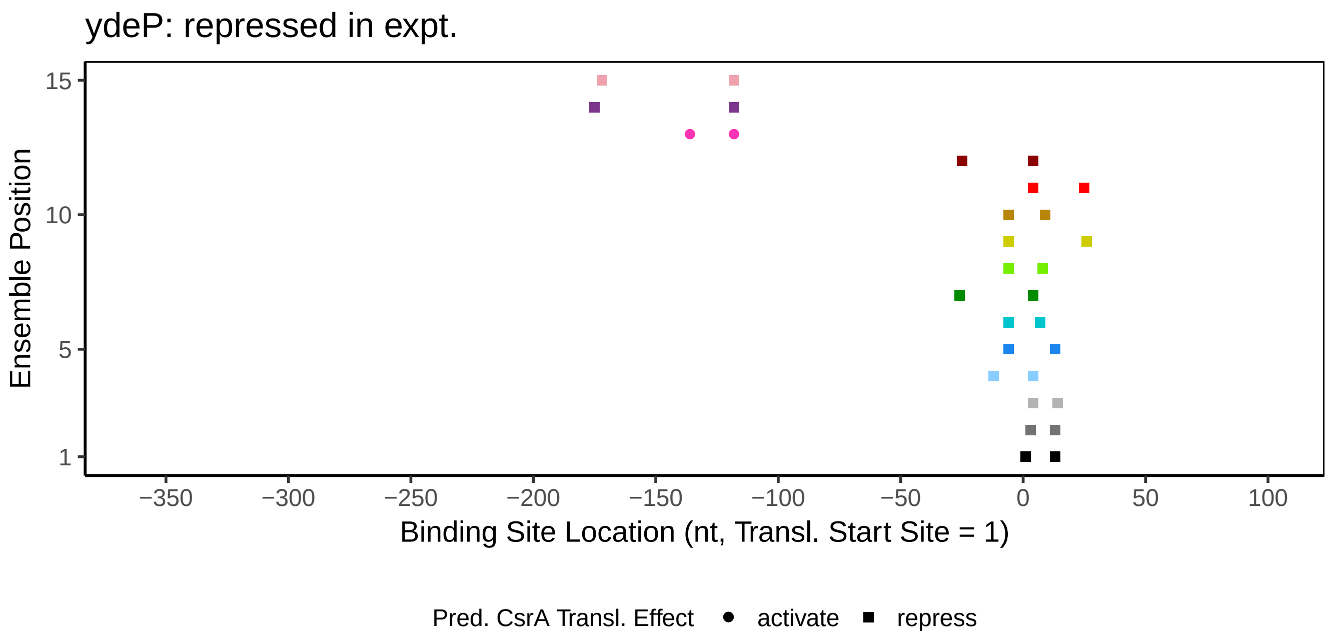
ydeP: repressed in expt.
Ensemble Position
Binding Site Location (nt, Transl. Start Site = 1)
Pred. CsrA Transl. Effect
activate
repress

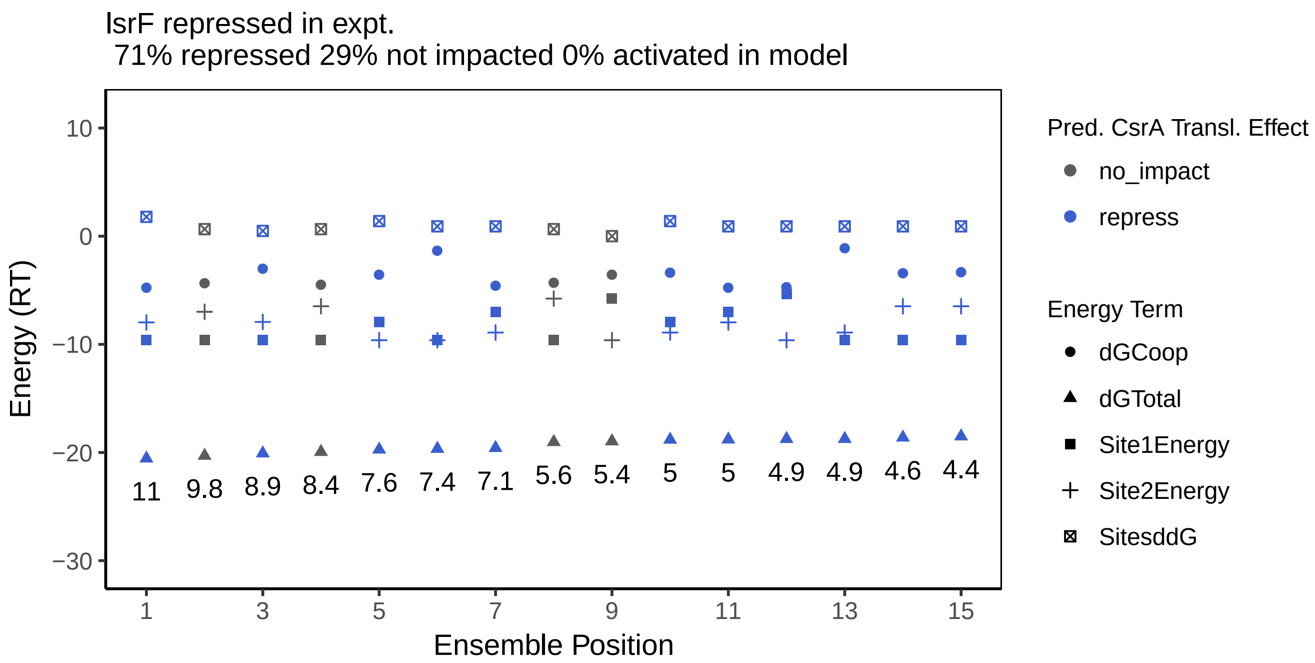
lsrF repressed in expt.
71% repressed 29% not impacted 0% activated in model
Energy (RT)
Ensemble Position
11
9.8
8.9
8.4
7.6
7.4
7.1
5.6
5.4
5
5
4.9
4.9
4.6
4.4
Pred. CsrA Transl. Effect
no_impact
repress
Energy Term
dGCoop
dGTotal
Site1Energy
Site2Energy
SitesddG

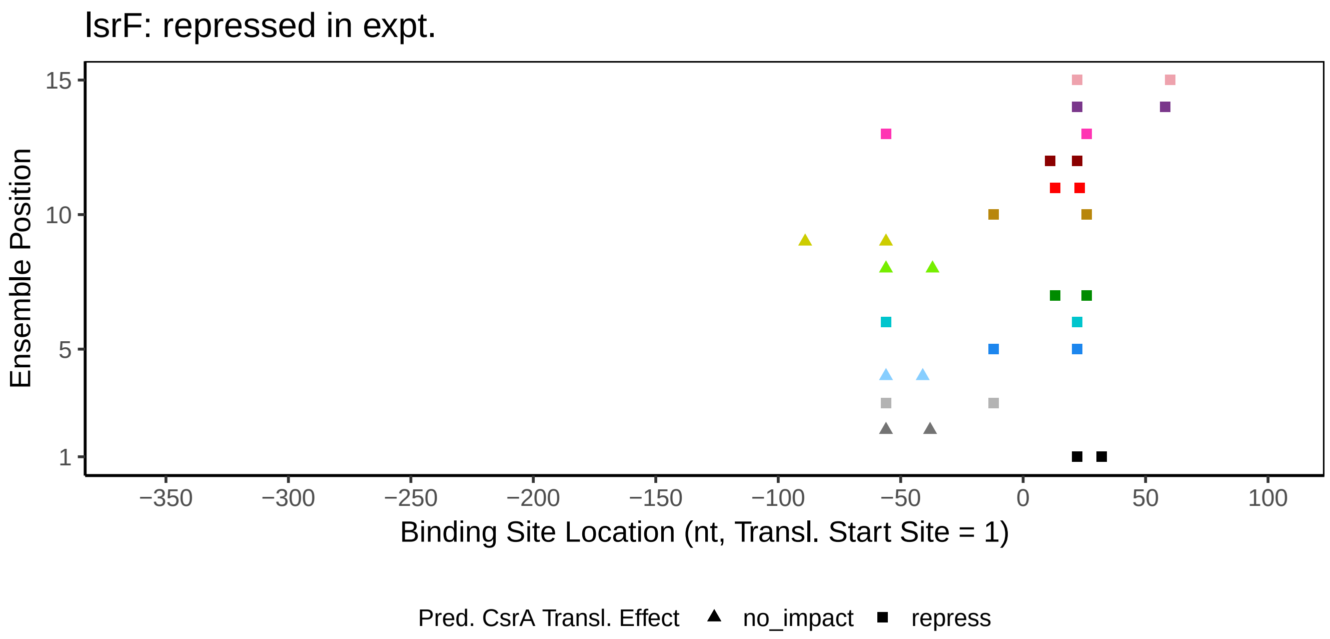
lsrF: repressed in expt.
Ensemble Position
Binding Site Location (nt, Transl. Start Site = 1)
Pred. CsrA Transl. Effect
no_impact
repress

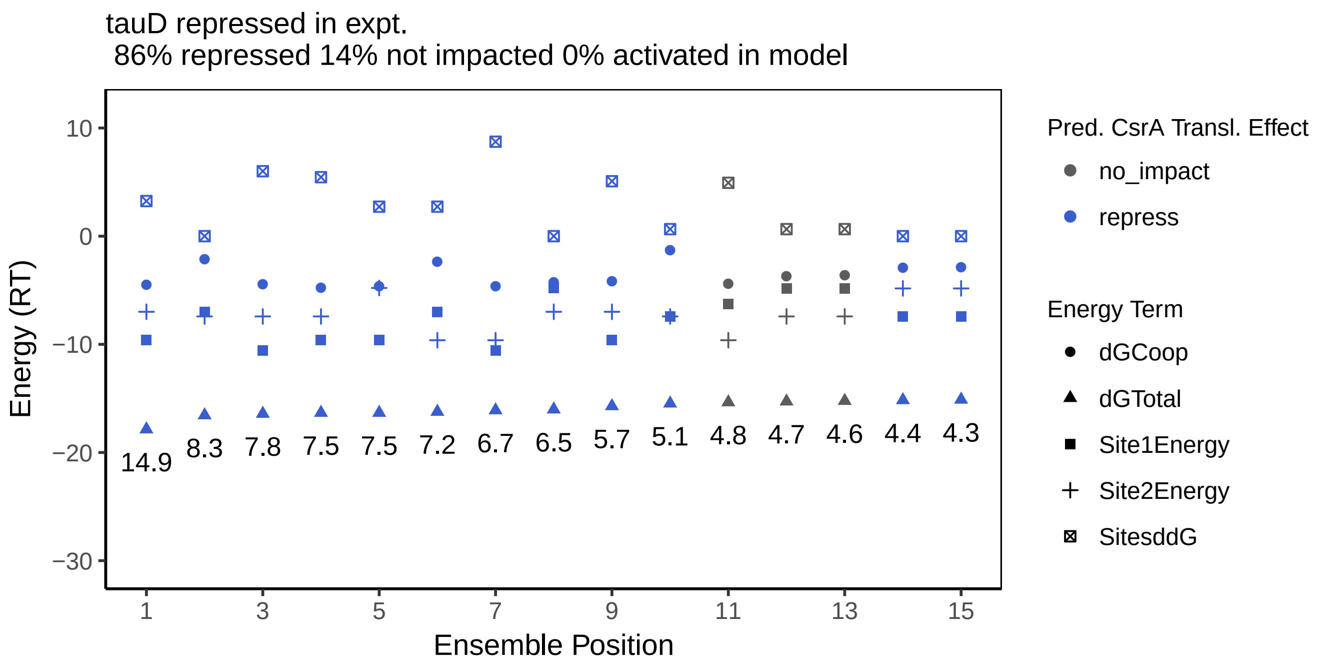

tauD repressed in expt.
86% repressed 14% not impacted 0% activated in model
Energy (RT)
10
0
−10
−20
−30
14.9
8.3
7.8
7.5
7.5
7.2
6.7
6.5
5.7
5.1
4.8
4.7
4.6
4.4
4.3
1
3
5
7
9
11
13
15
Ensemble Position
Pred. CsrA Transl. Effect
no_impact
repress
Energy Term
dGCoop
dGTotal
Site1Energy
Site2Energy
SitesddG

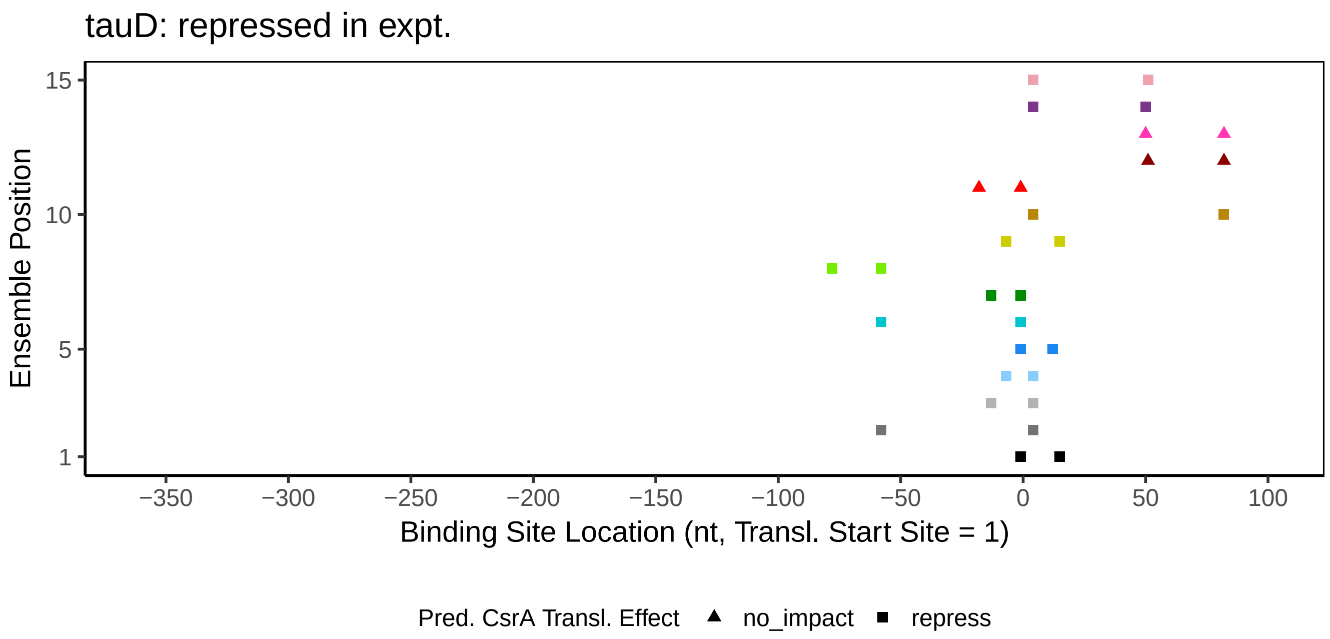

tauD: repressed in expt.
Ensemble Position
15
10
5
1
−350
−300
−250
−200
−150
−100
−50
0
50
100
Binding Site Location (nt, Transl. Start Site = 1)
Pred. CsrA Transl. Effect
no_impact
repress

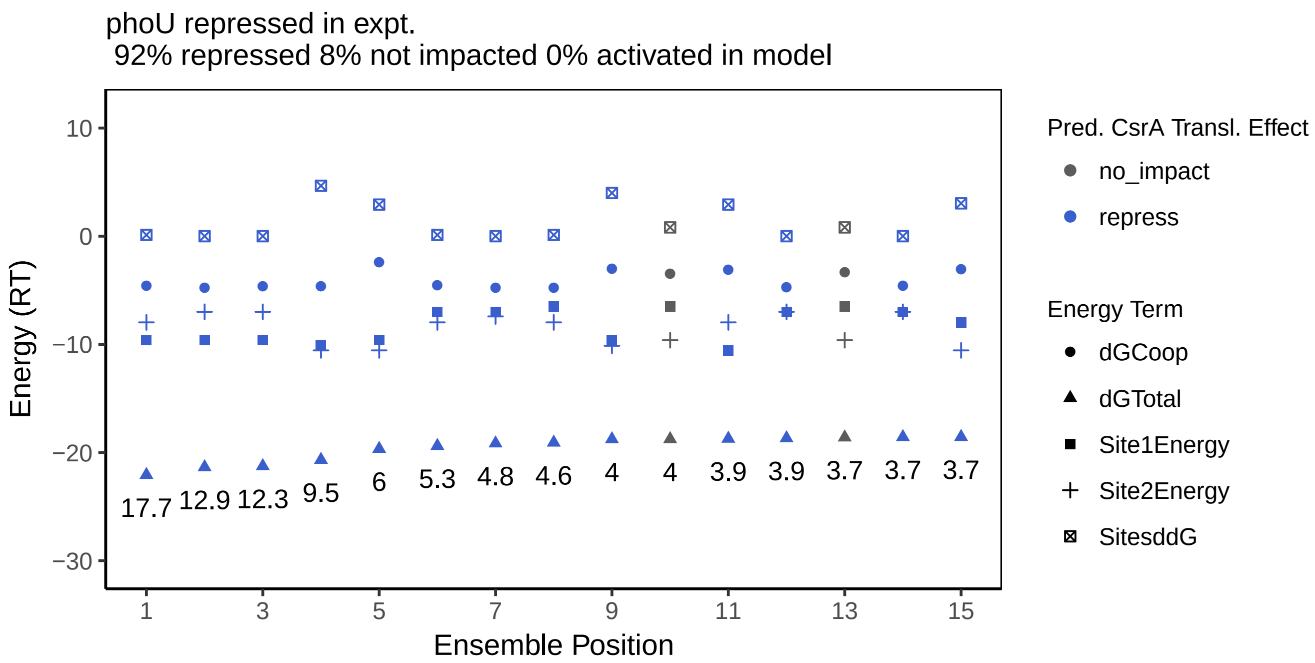
phoU repressed in expt.
92% repressed 8% not impacted 0% activated in model
Energy (RT)
Ensemble Position
17.7
12.9
12.3
9.5
6
5.3
4.8
4.6
4
4
3.9
3.9
3.7
3.7
3.7
Pred. CsrA Transl. Effect
no_impact
repress
Energy Term
dGCoop
dGTotal
Site1Energy
Site2Energy
SitesddG

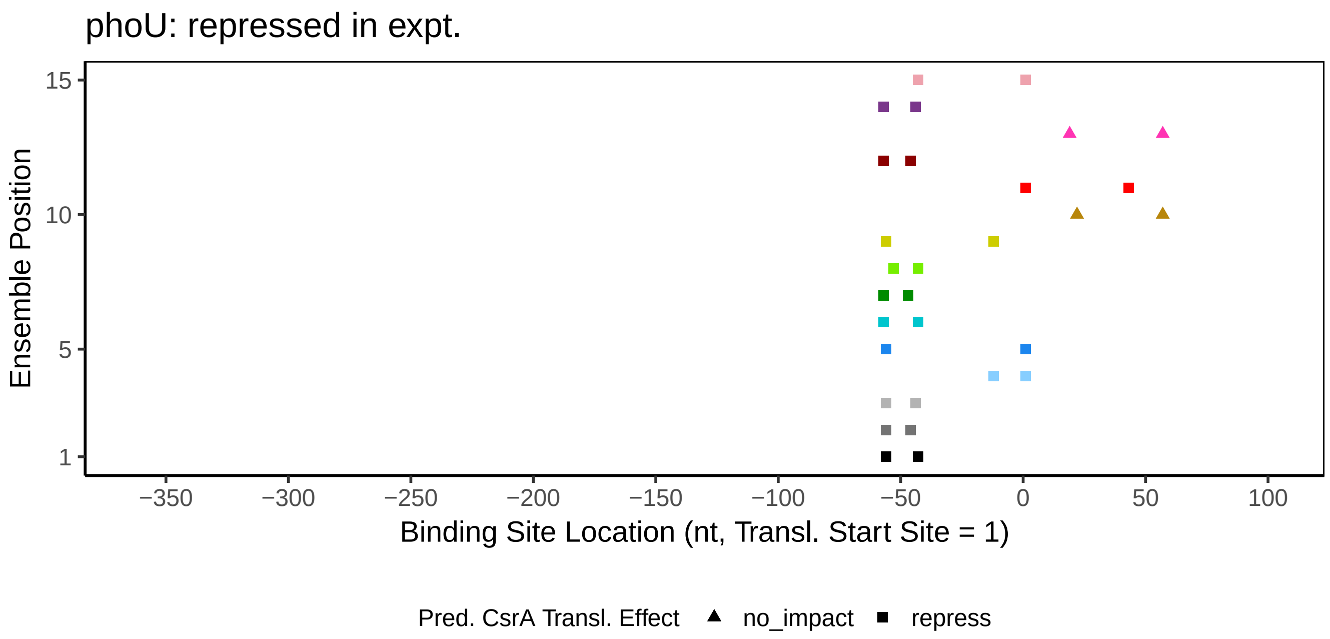
phoU: repressed in expt.
Ensemble Position
Binding Site Location (nt, Transl. Start Site = 1)
Pred. CsrA Transl. Effect
no_impact
repress

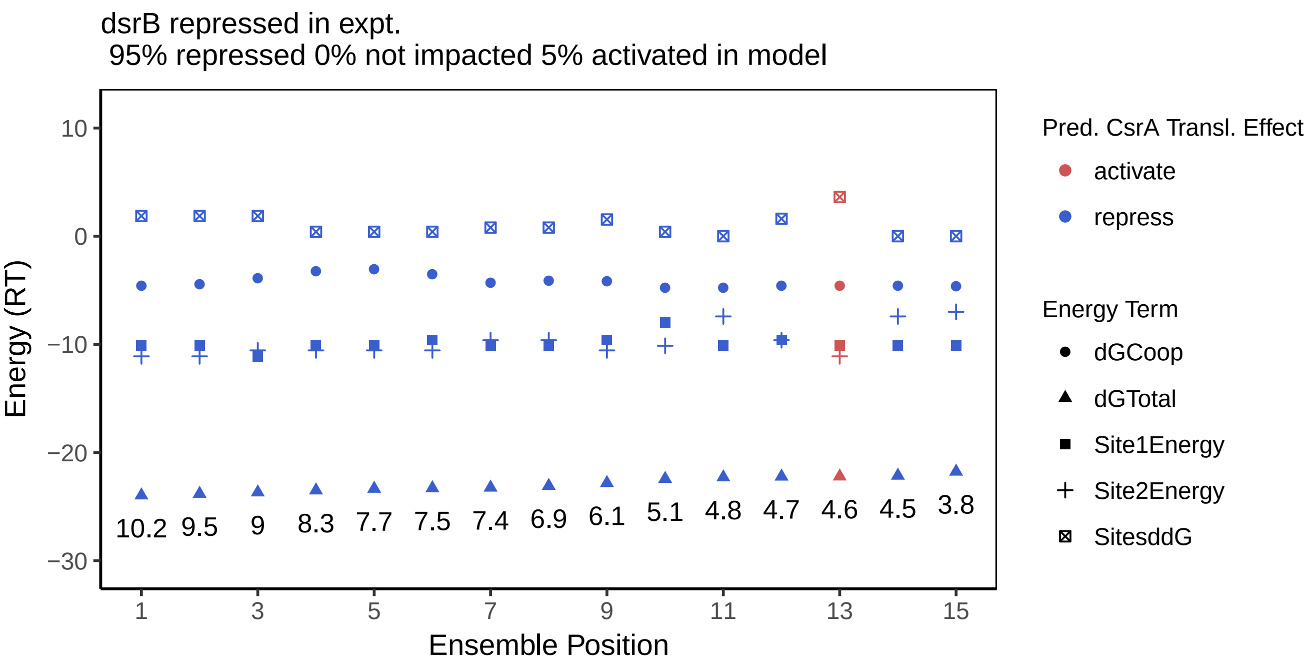
dsrB repressed in expt.
95% repressed 0% not impacted 5% activated in model
Energy (RT)
10
0
−10
−20
−30
10.2
9.5
9
8.3
7.7
7.5
7.4
6.9
6.1
5.1
4.8
4.7
4.6
4.5
3.8
1
3
5
7
9
11
13
15
Ensemble Position
Pred. CsrA Transl. Effect
activate
repress
Energy Term
dGCoop
dGTotal
Site1Energy
Site2Energy
SitesddG

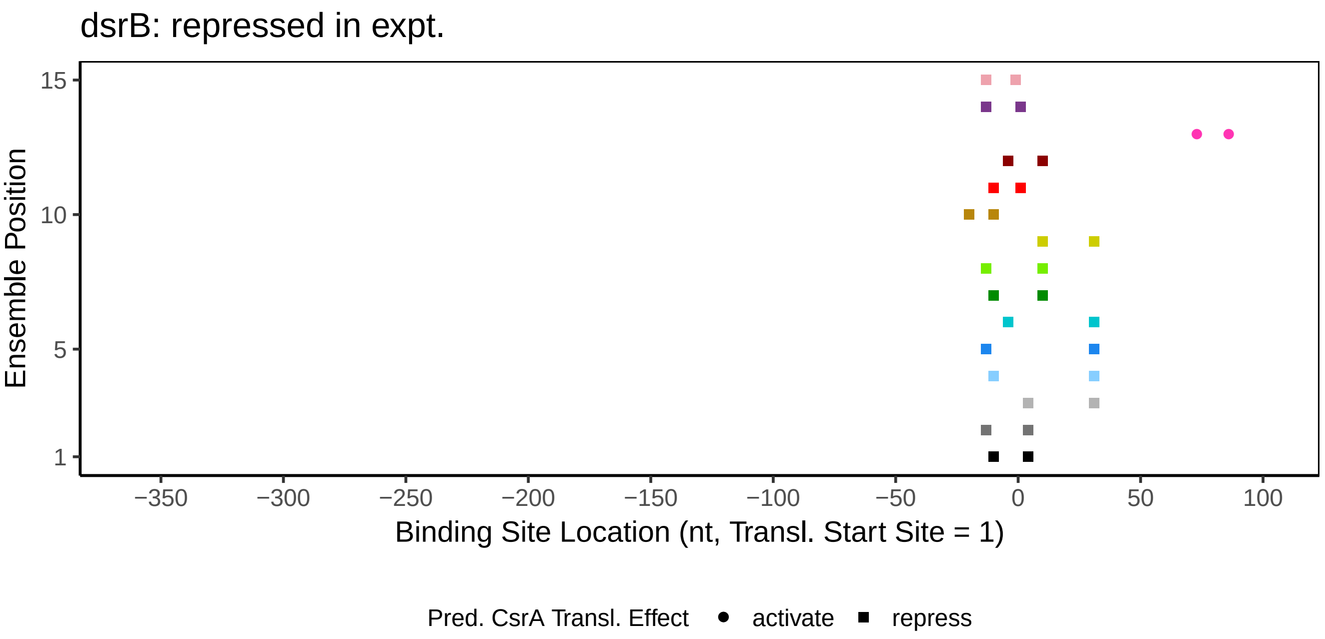
dsrB: repressed in expt.
Ensemble Position
15
10
5
1
−350
−300
−250
−200
−150
−100
−50
0
50
100
Binding Site Location (nt, Transl. Start Site = 1)
Pred. CsrA Transl. Effect
activate
repress

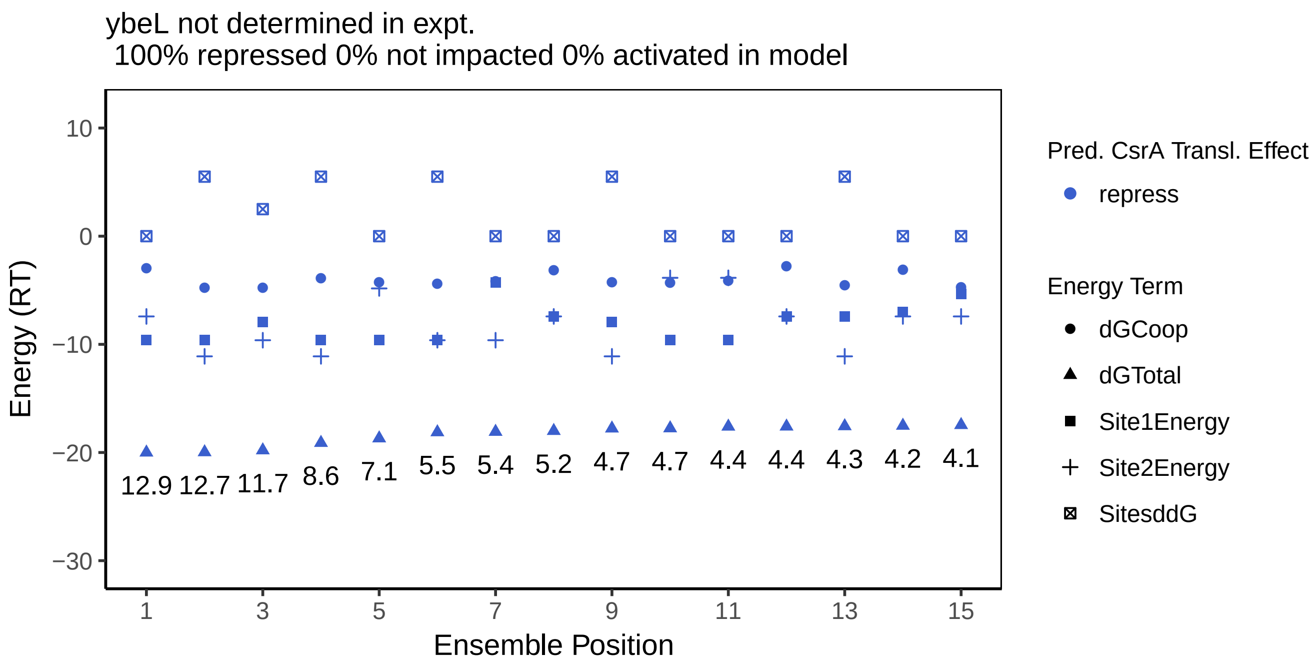

ybeL not determined in expt.
100% repressed 0% not impacted 0% activated in model
Energy (RT)
Ensemble Position
12.9 12.7 11.7 8.6 7.1 5.5 5.4 5.2 4.7 4.7 4.4 4.4 4.3 4.2 4.1
Pred. CsrA Transl. Effect
repress
Energy Term
dGCoop
dGTotal
Site1Energy
Site2Energy
SitesddG

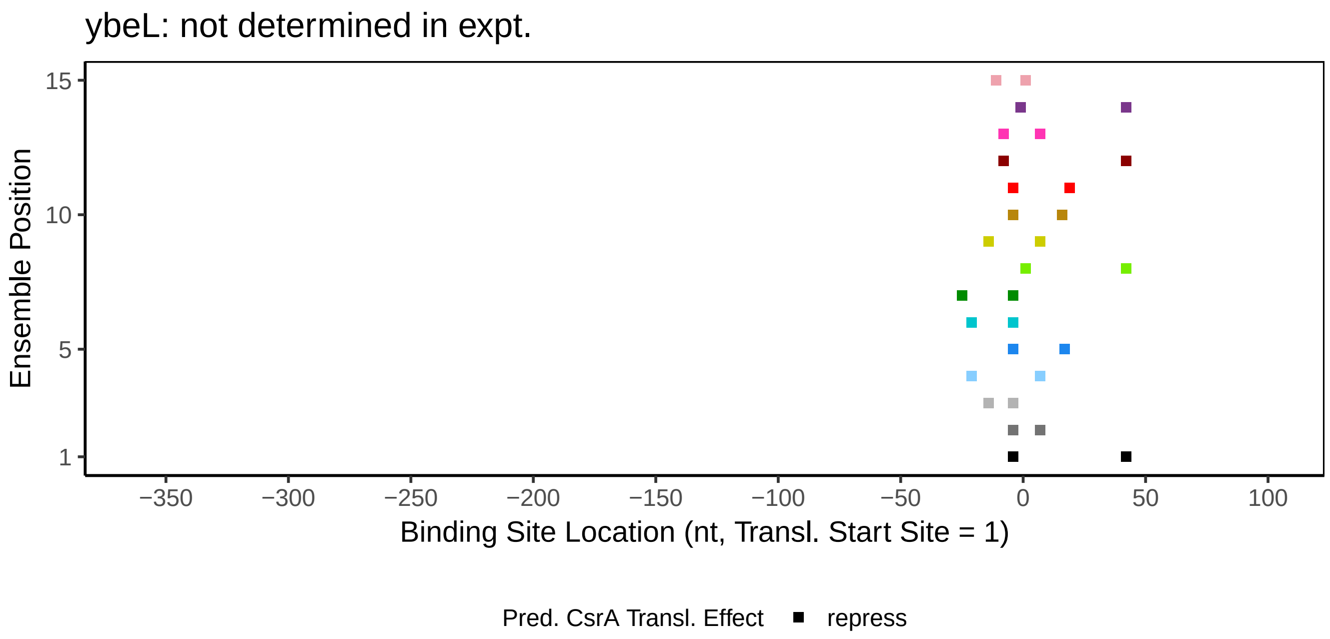

ybeL: not determined in expt.
Ensemble Position
Binding Site Location (nt, Transl. Start Site = 1)
Pred. CsrA Transl. Effect
repress

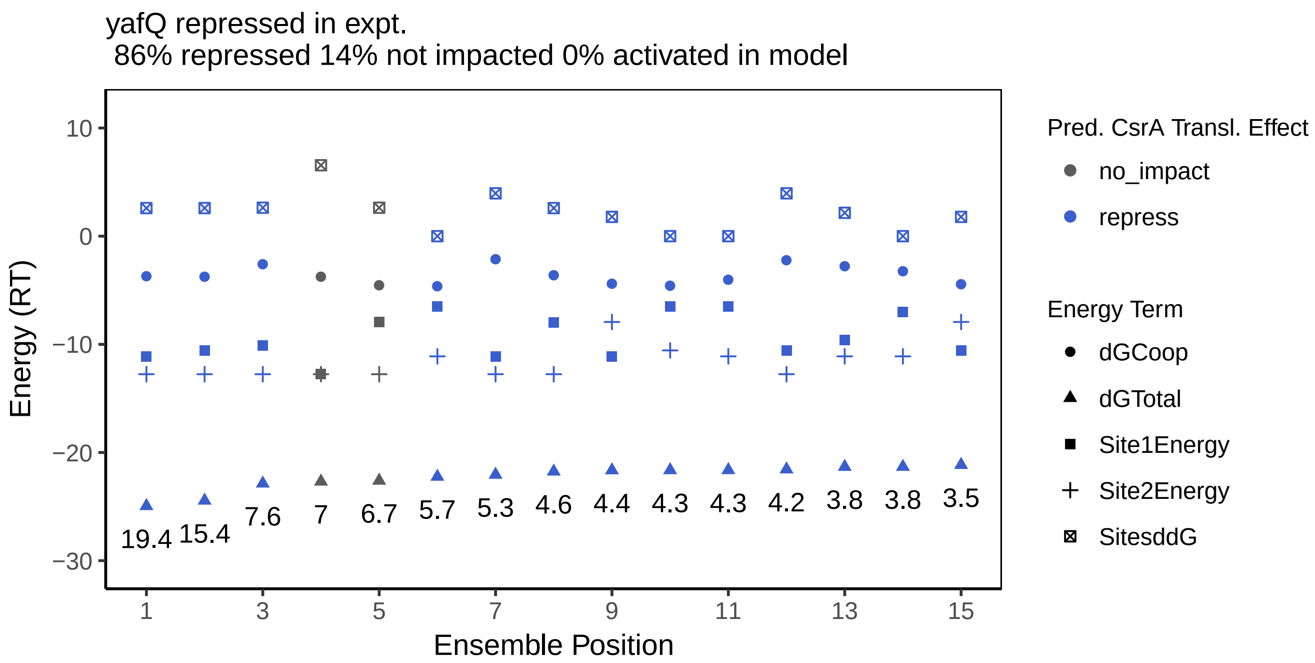

yafQ repressed in expt.
86% repressed 14% not impacted 0% activated in model
Energy (RT)
Ensemble Position
19.4
15.4
7.6
7
6.7
5.7
5.3
4.6
4.4
4.3
4.3
4.2
3.8
3.8
3.5
Pred. CsrA Transl. Effect
no_impact
repress
Energy Term
dGCoop
dGTotal
Site1Energy
Site2Energy
SitesddG

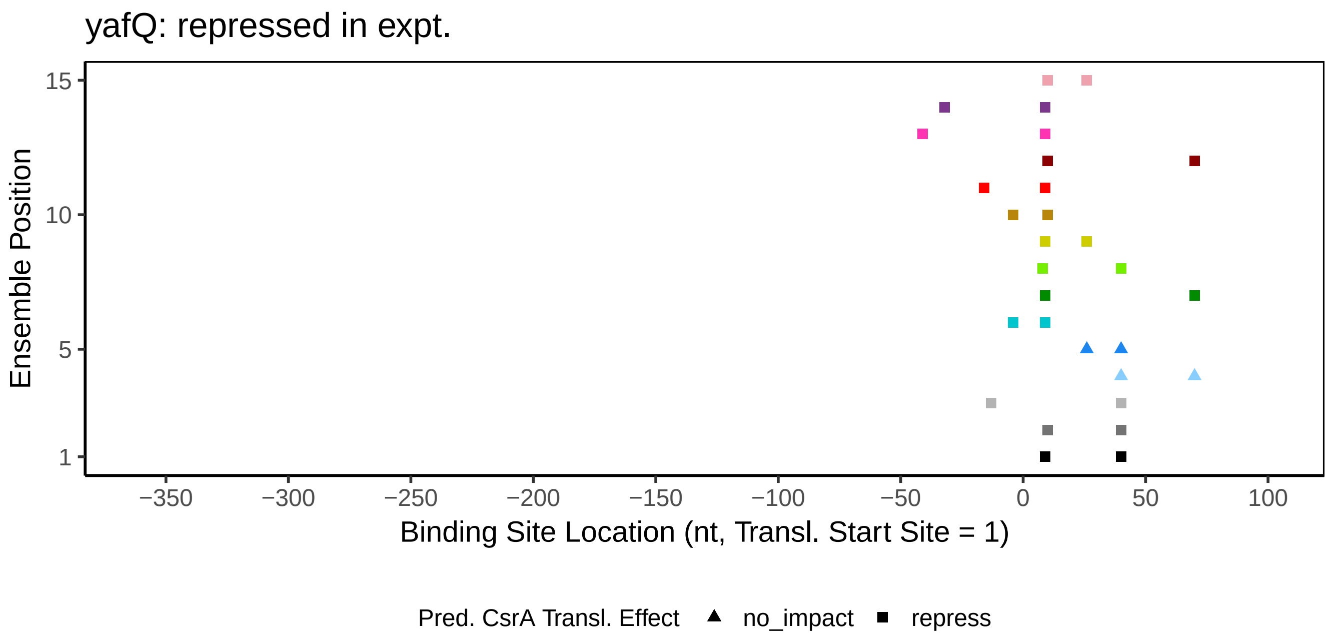

yafQ: repressed in expt.
Ensemble Position
Binding Site Location (nt, Transl. Start Site = 1)
Pred. CsrA Transl. Effect
no_impact
repress

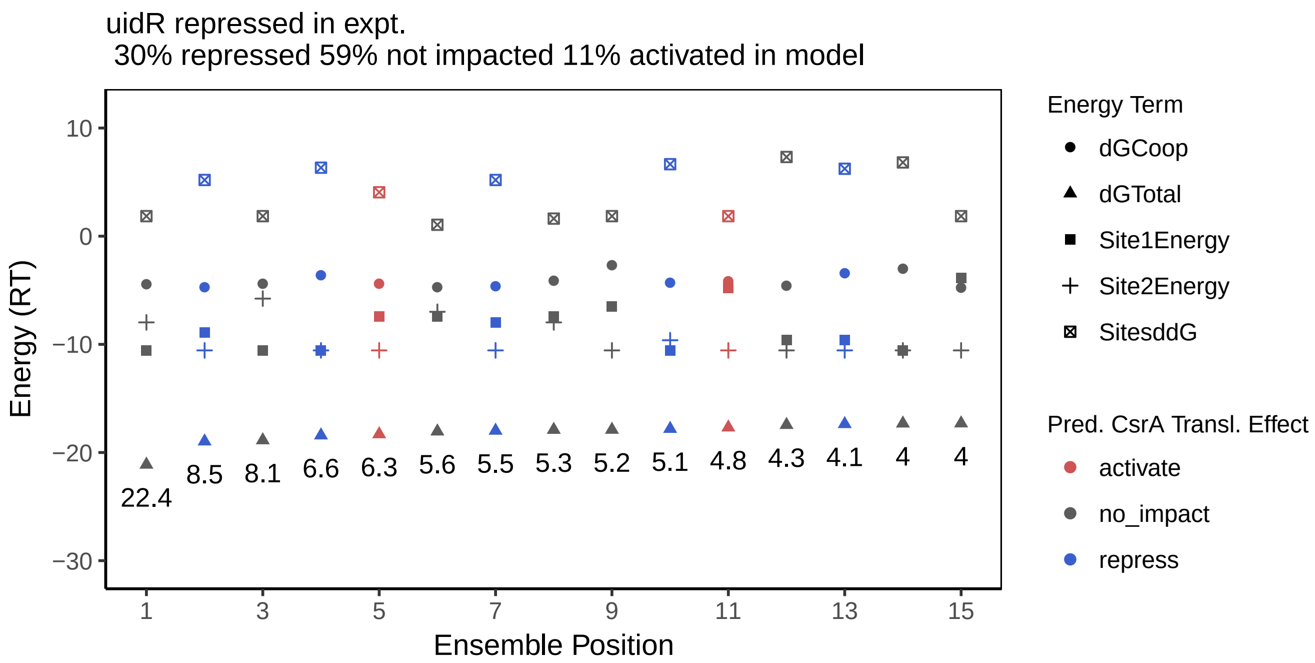

uidR repressed in expt.
30% repressed 59% not impacted 11% activated in model
Energy (RT)
10
0
−10
−20
−30
1
3
5
7
9
11
13
15
Ensemble Position
22.4
8.5
8.1
6.6
6.3
5.6
5.5
5.3
5.2
5.1
4.8
4.3
4.1
4
4
Energy Term
dGCoop
dGTotal
Site1Energy
Site2Energy
SitesddG
Pred. CsrA Transl. Effect
activate
no_impact
repress

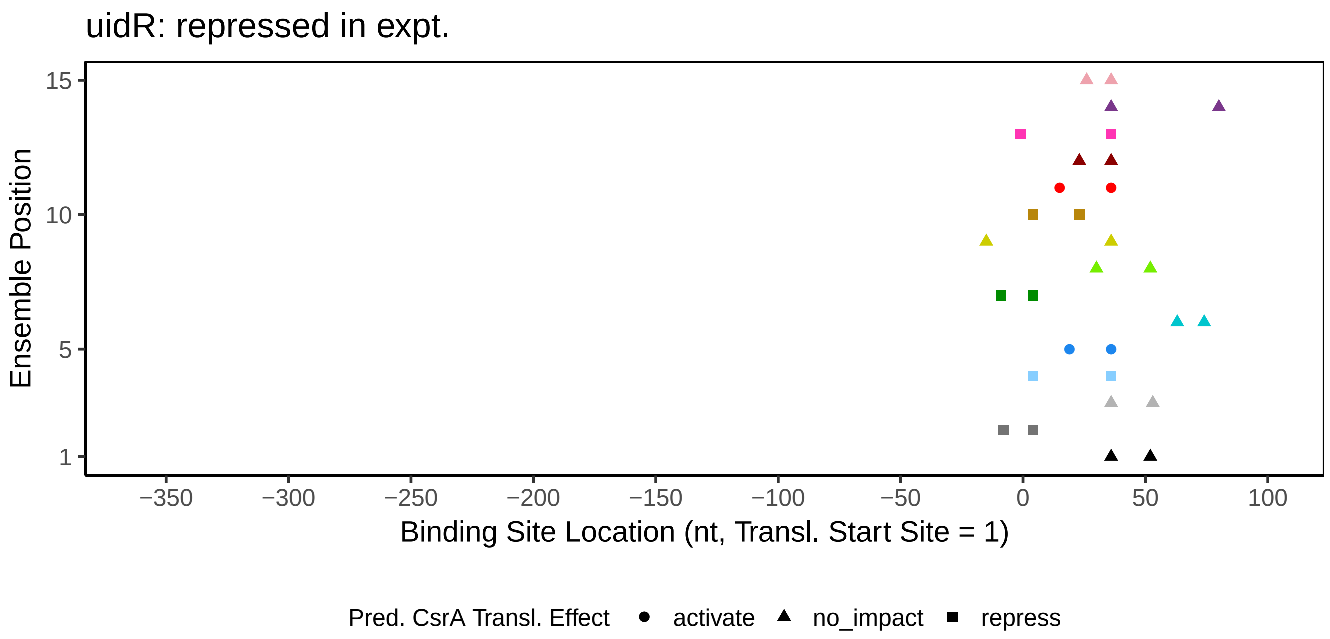

uidR: repressed in expt.
Ensemble Position
15
10
5
1
−350
−300
−250
−200
−150
−100
−50
0
50
100
Binding Site Location (nt, Transl. Start Site = 1)
Pred. CsrA Transl. Effect
activate
no_impact
repress

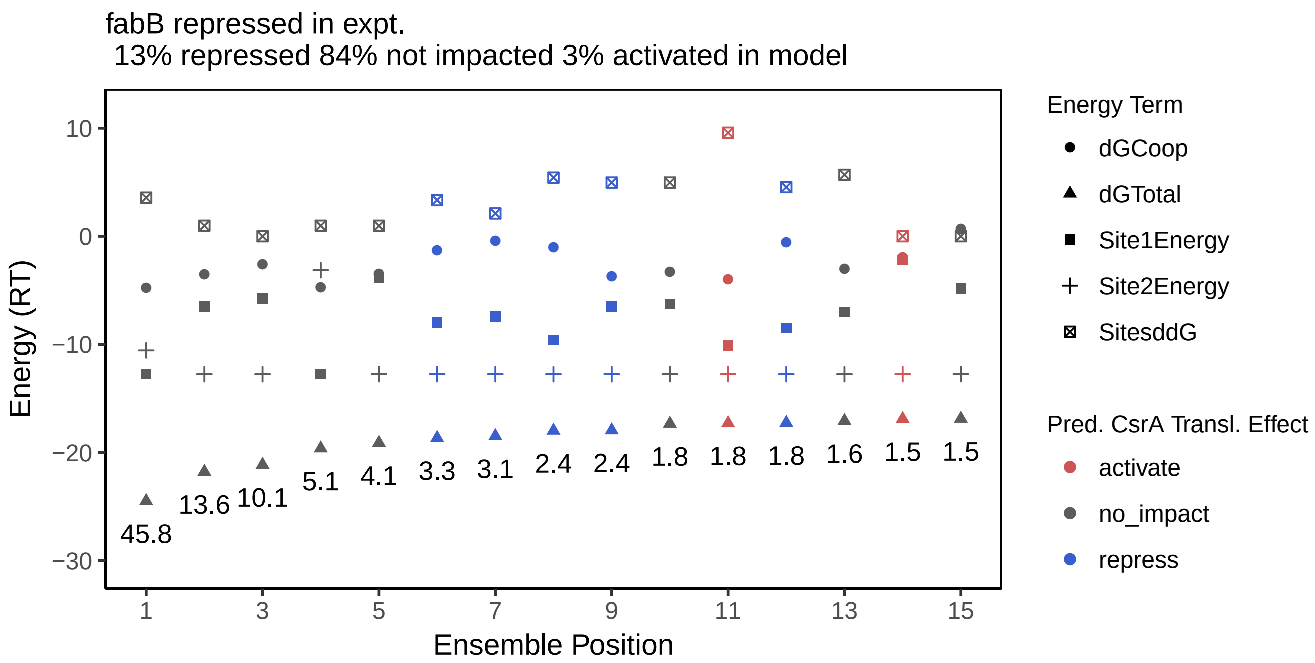

fabB repressed in expt.
13% repressed 84% not impacted 3% activated in model
Energy (RT)
Ensemble Position
45.8
13.6
10.1
5.1
4.1
3.3
3.1
2.4
2.4
1.8
1.8
1.8
1.6
1.5
1.5
Energy Term
dGCoop
dGTotal
Site1Energy
Site2Energy
SitesddG
Pred. CsrA Transl. Effect
activate
no_impact
repress

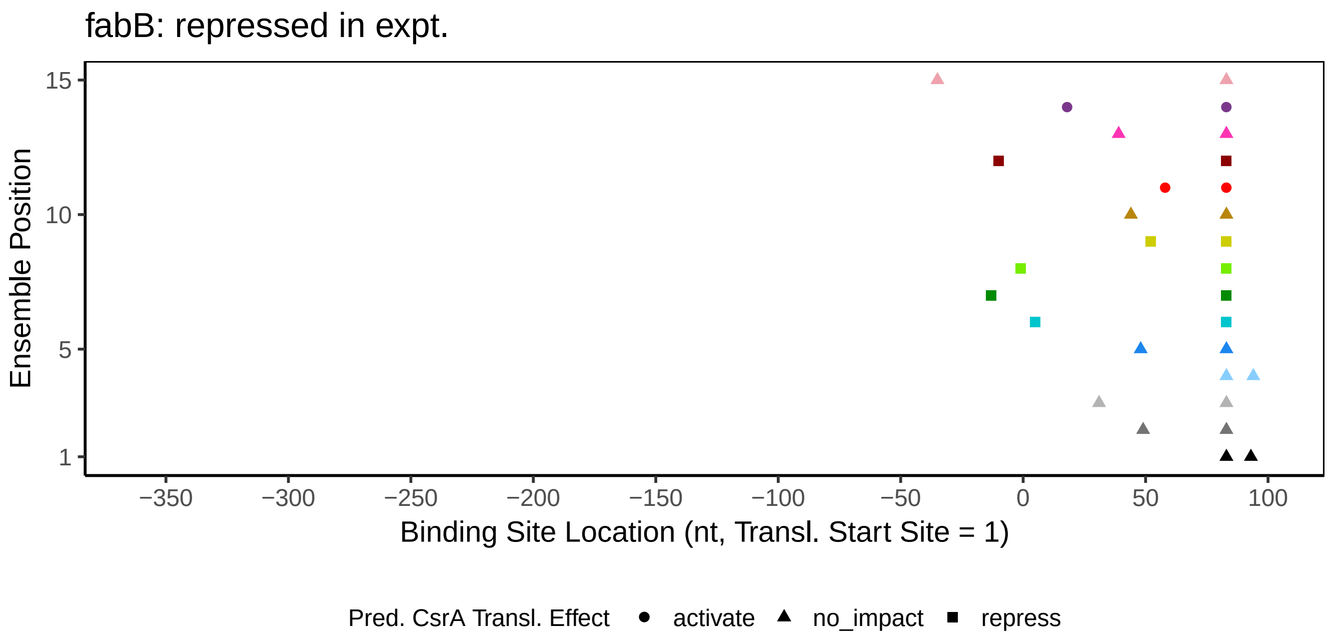

fabB: repressed in expt.
Ensemble Position
Binding Site Location (nt, Transl. Start Site = 1)
Pred. CsrA Transl. Effect
activate
no_impact
repress

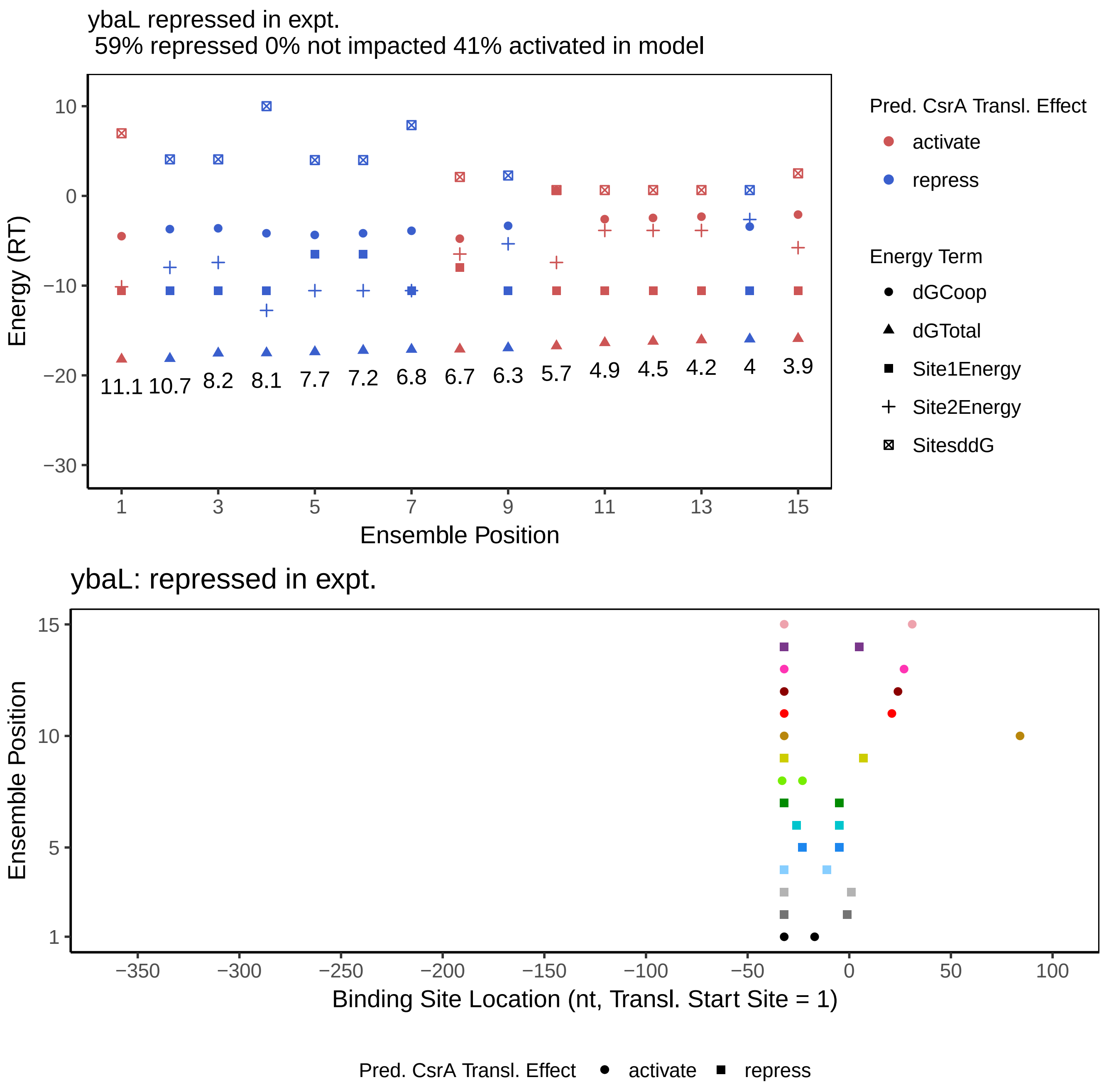
ybaL repressed in expt.
59% repressed 0% not impacted 41% activated in model
Energy (RT)
10
0
−10
−20
−30
11.1 10.7 8.2 8.1 7.7 7.2 6.8 6.7 6.3 5.7 4.9 4.5 4.2 4 3.9
1 3 5 7 9 11 13 15
Ensemble Position
Pred. CsrA Transl. Effect
activate
repress
Energy Term
dGCoop
dGTotal
Site1Energy
Site2Energy
SitesddG
ybaL: repressed in expt.
Ensemble Position
15
10
5
1
−350 −300 −250 −200 −150 −100 −50 0 50 100
Binding Site Location (nt, Transl. Start Site = 1)
Pred. CsrA Transl. Effect
activate
repress

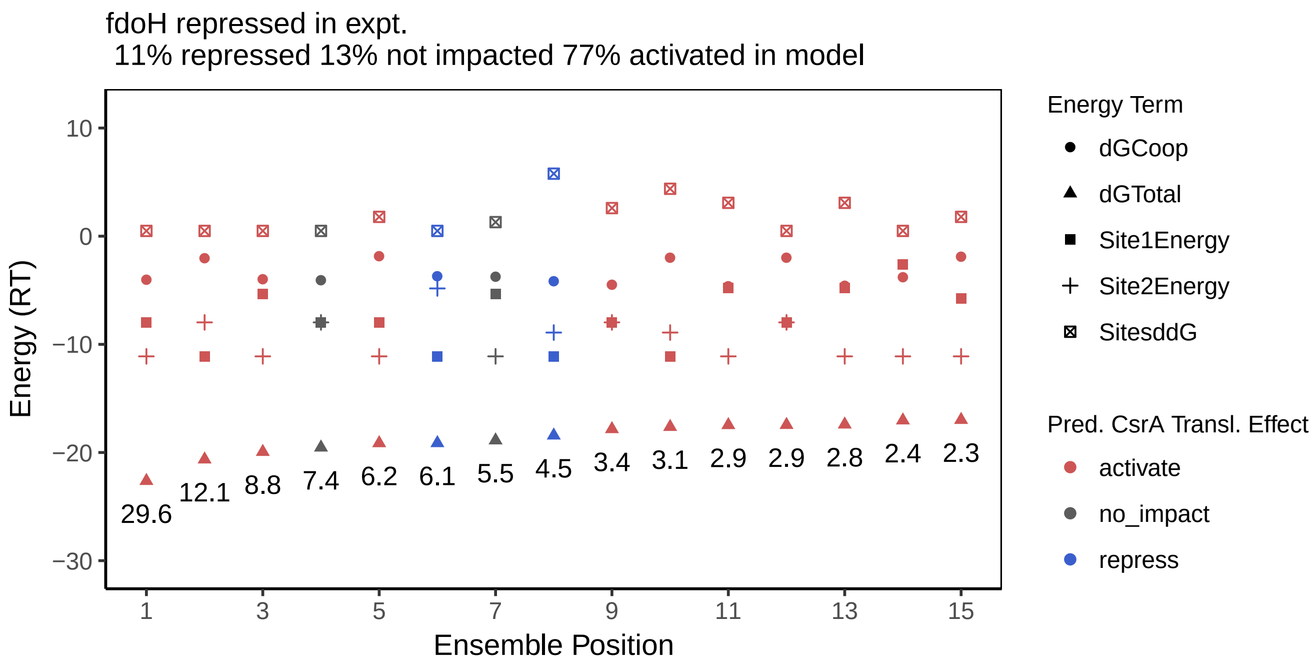
fdoH repressed in expt.
11% repressed 13% not impacted 77% activated in model
Energy (RT)
10
0
−10
−20
−30
29.6
12.1
8.8
7.4
6.2
6.1
5.5
4.5
3.4
3.1
2.9
2.9
2.8
2.4
2.3
1
3
5
7
9
11
13
15
Ensemble Position
Energy Term
dGCoop
dGTotal
Site1Energy
Site2Energy
SitesddG
Pred. CsrA Transl. Effect
activate
no_impact
repress

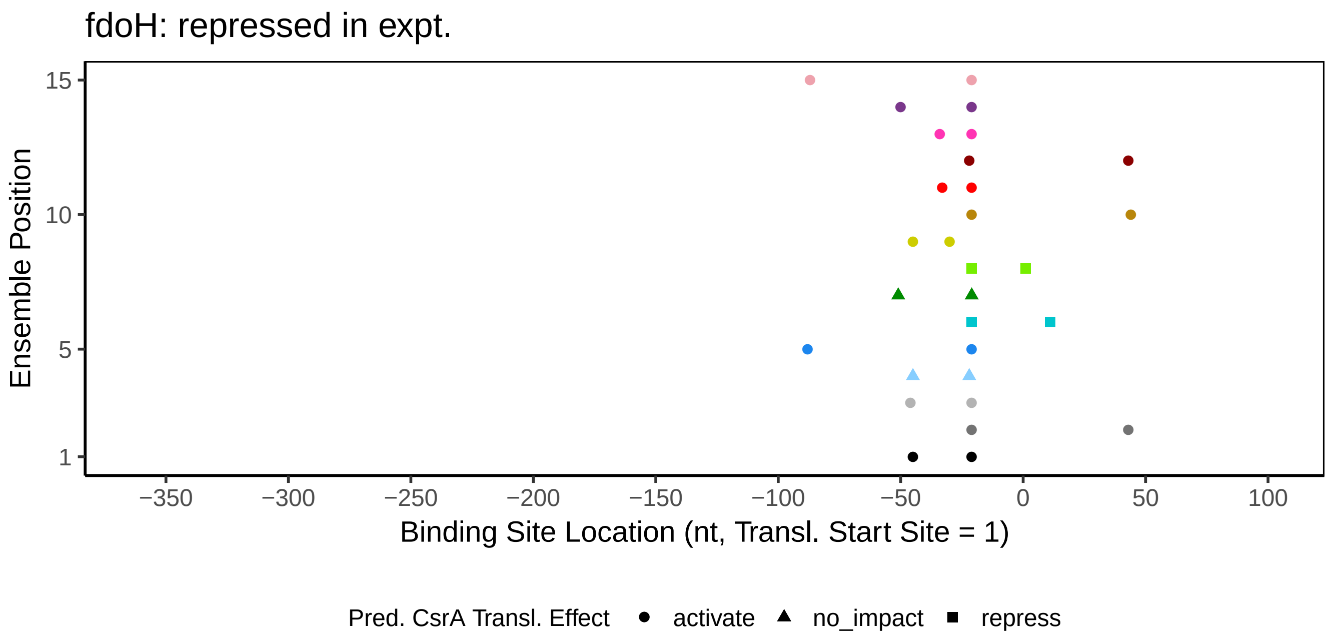
fdoH: repressed in expt.
Ensemble Position
15
10
5
1
−350
−300
−250
−200
−150
−100
−50
0
50
100
Binding Site Location (nt, Transl. Start Site = 1)
Pred. CsrA Transl. Effect
activate
no_impact
repress

CsiD repressed in expt.
28% repressed 0% not impacted 72% activated in model

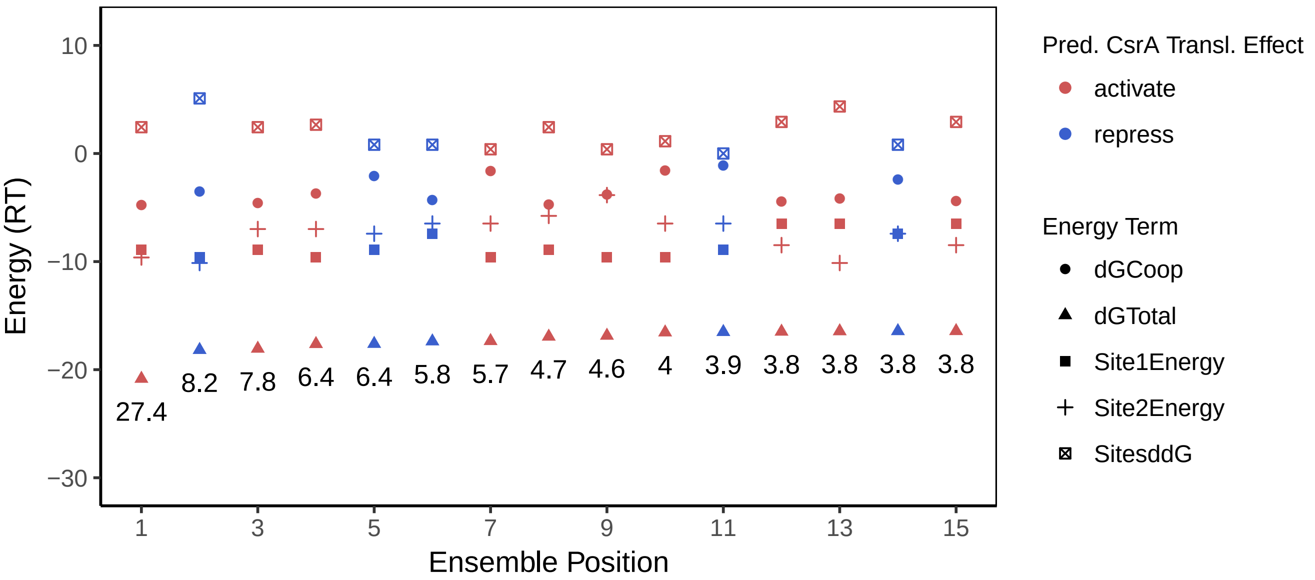

CsiD: repressed in expt.

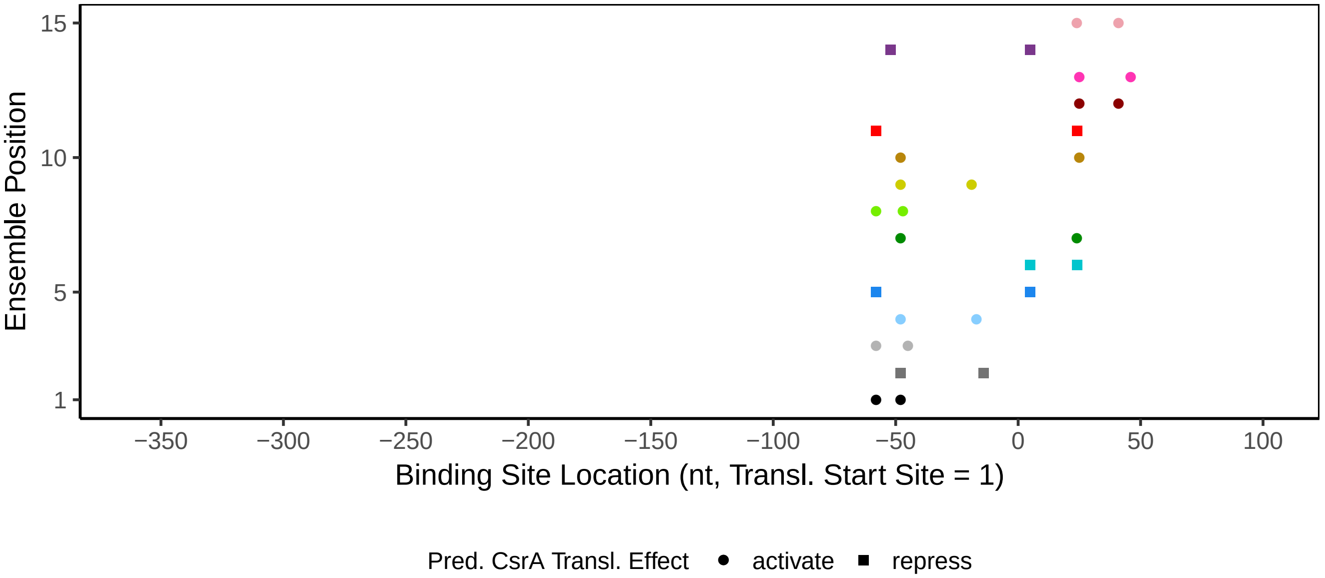

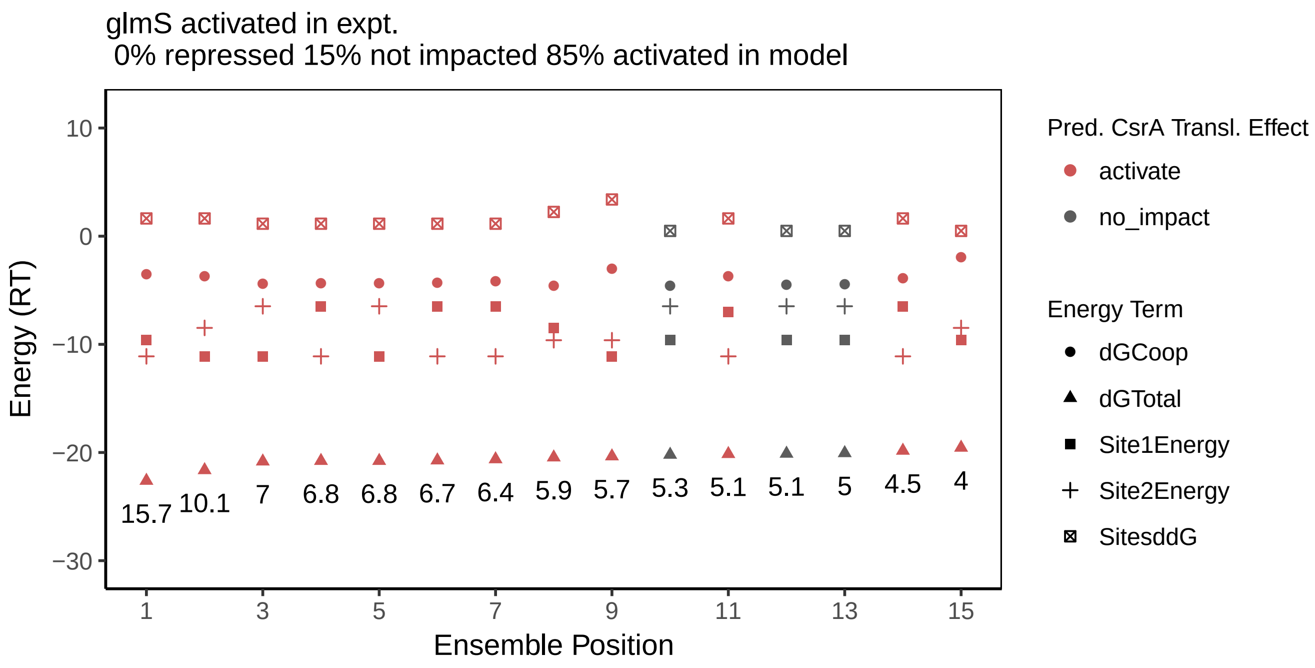
glmS activated in expt.
0% repressed 15% not impacted 85% activated in model
Energy (RT)
10
0
−10
−20
−30
15.7
10.1
7
6.8
6.8
6.7
6.4
5.9
5.7
5.3
5.1
5.1
5
4.5
4
1
3
5
7
9
11
13
15
Ensemble Position
Pred. CsrA Transl. Effect
activate
no_impact
Energy Term
dGCoop
dGTotal
Site1Energy
Site2Energy
SitesddG

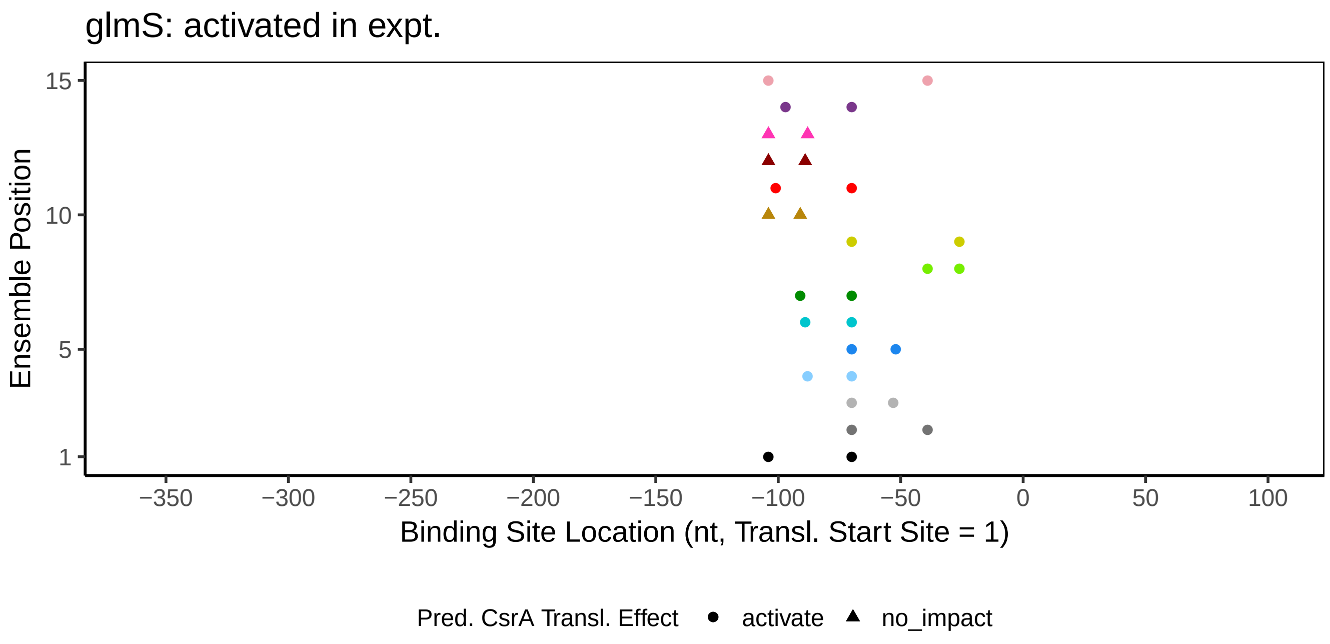
glmS: activated in expt.
Ensemble Position
15
10
5
1
−350
−300
−250
−200
−150
−100
−50
0
50
100
Binding Site Location (nt, Transl. Start Site = 1)
Pred. CsrA Transl. Effect
activate
no_impact

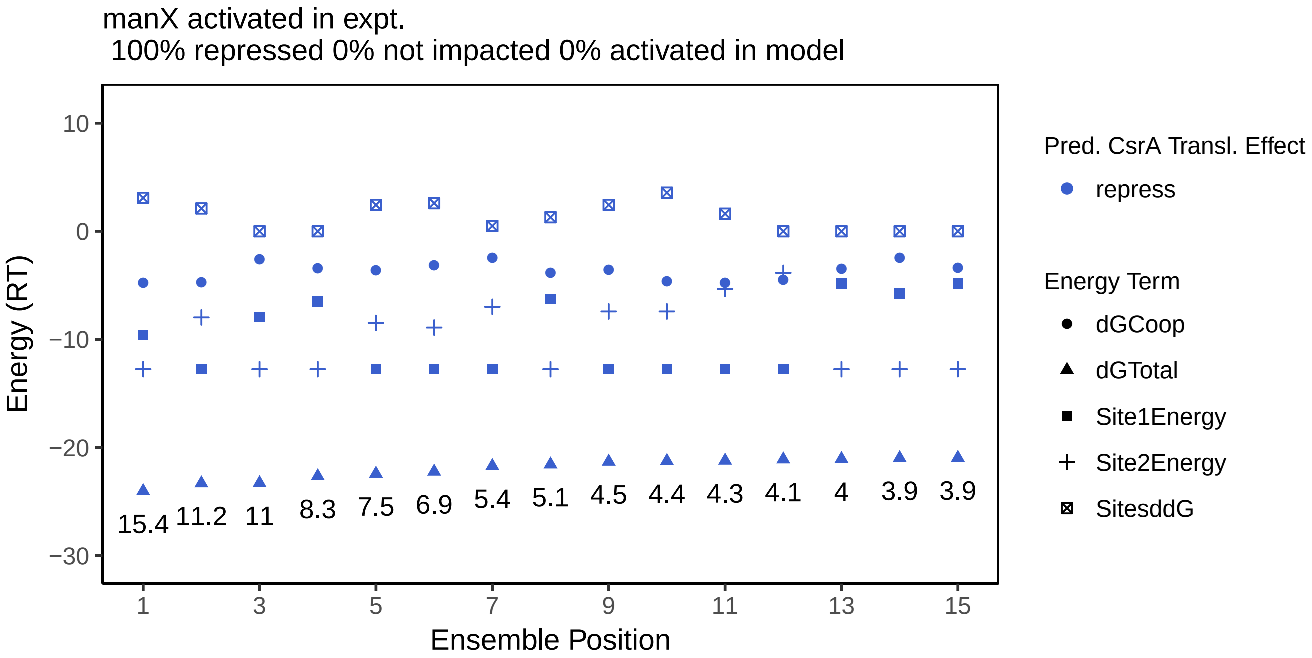

manX activated in expt.
100% repressed 0% not impacted 0% activated in model
Energy (RT)
10
0
−10
−20
−30
15.4
11.2
11
8.3
7.5
6.9
5.4
5.1
4.5
4.4
4.3
4.1
4
3.9
3.9
1
3
5
7
9
11
13
15
Ensemble Position
Pred. CsrA Transl. Effect
repress
Energy Term
dGCoop
dGTotal
Site1Energy
Site2Energy
SitesddG

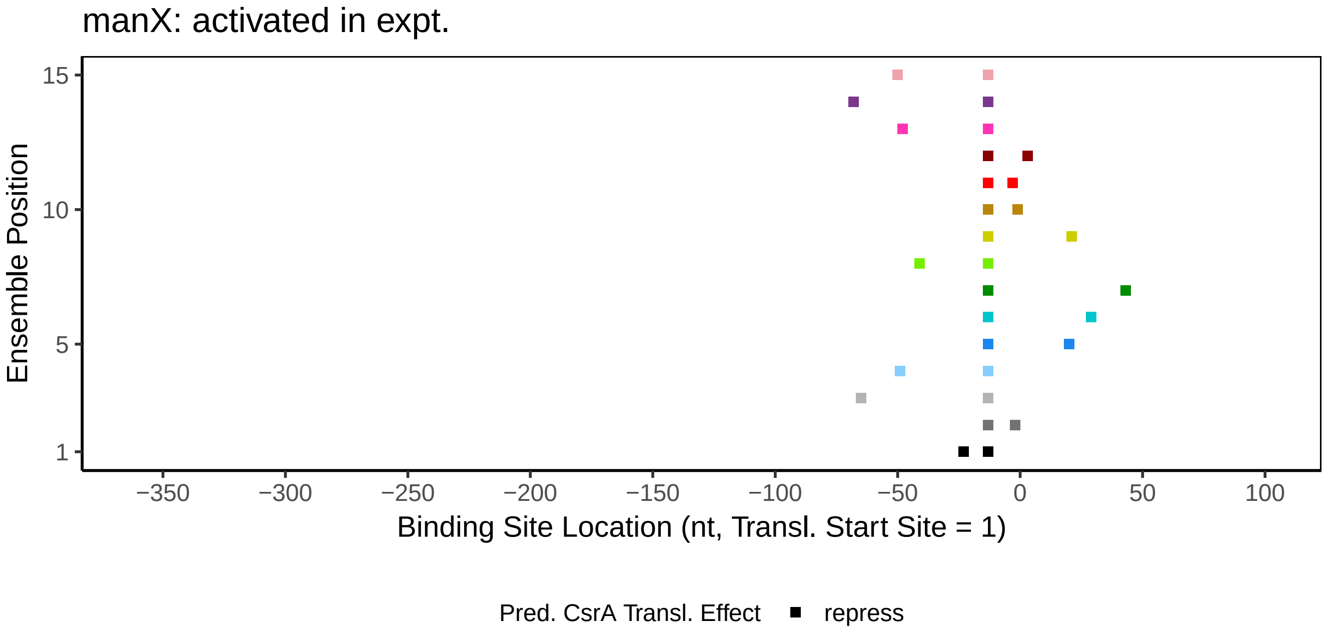

manX: activated in expt.
Ensemble Position
15
10
5
1
−350
−300
−250
−200
−150
−100
−50
0
50
100
Binding Site Location (nt, Transl. Start Site = 1)
Pred. CsrA Transl. Effect
repress

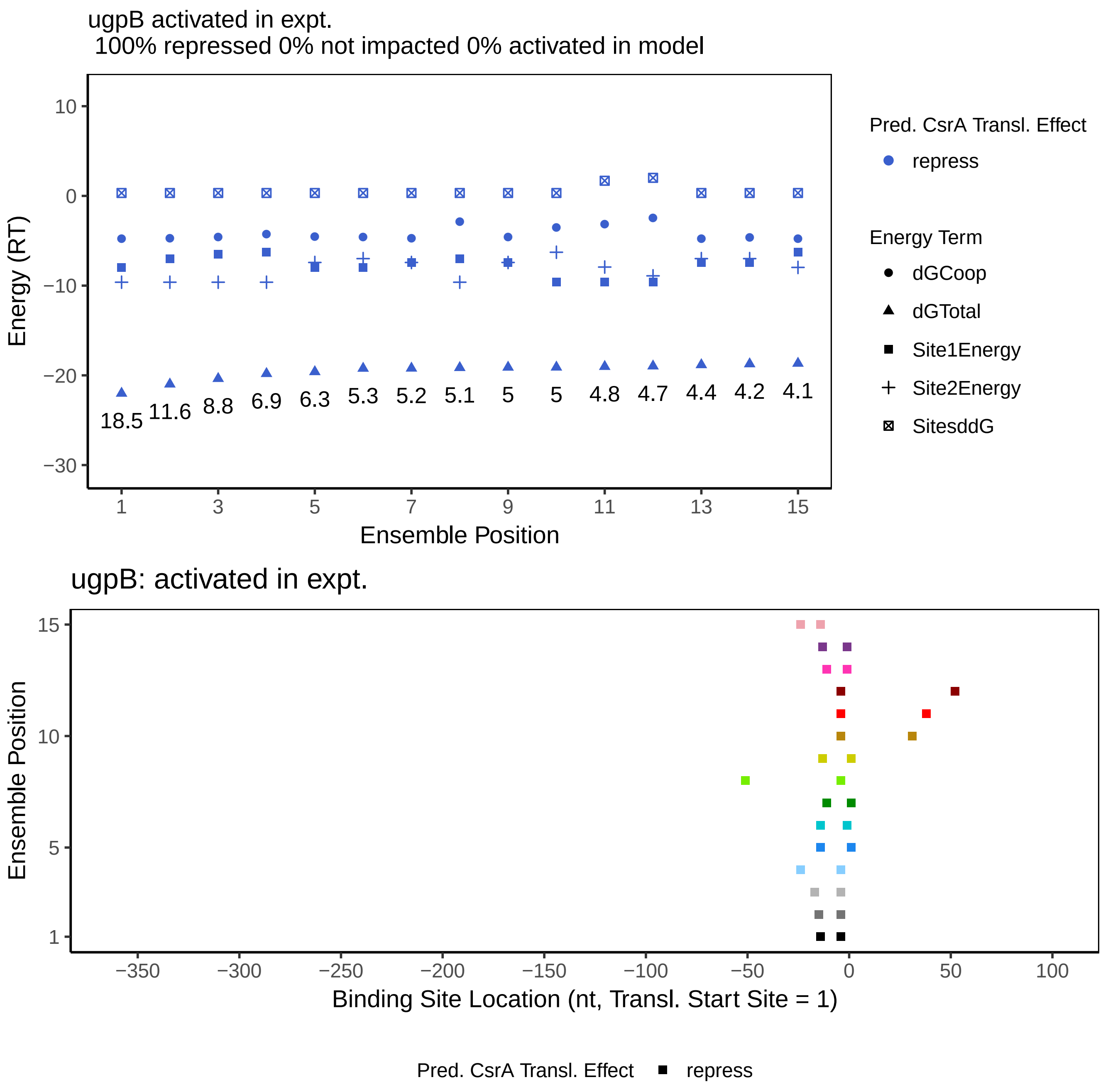

ugpB activated in expt.
100% repressed 0% not impacted 0% activated in model
Energy (RT)
Ensemble Position
18.5
11.6
8.8
6.9
6.3
5.3
5.2
5.1
5
5
4.8
4.7
4.4
4.2
4.1
Pred. CsrA Transl. Effect
repress
Energy Term
dGCoop
dGTotal
Site1Energy
Site2Energy
SitesddG
ugpB: activated in expt.
Ensemble Position
Binding Site Location (nt, Transl. Start Site = 1)
Pred. CsrA Transl. Effect
repress

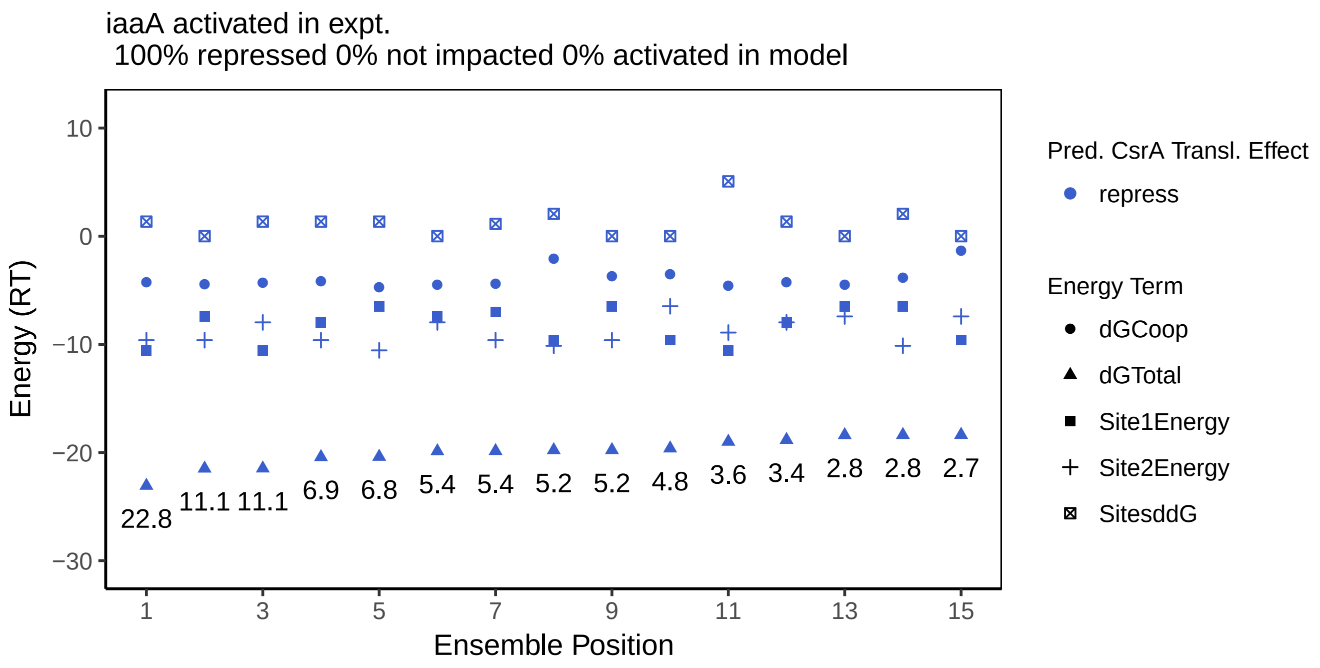

iaaA activated in expt.
100% repressed 0% not impacted 0% activated in model
Energy (RT)
10
0
−10
−20
−30
22.8
11.1
11.1
6.9
6.8
5.4
5.4
5.2
5.2
4.8
3.6
3.4
2.8
2.8
2.7
1
3
5
7
9
11
13
15
Ensemble Position
Pred. CsrA Transl. Effect
repress
Energy Term
dGCoop
dGTotal
Site1Energy
Site2Energy
SitesddG

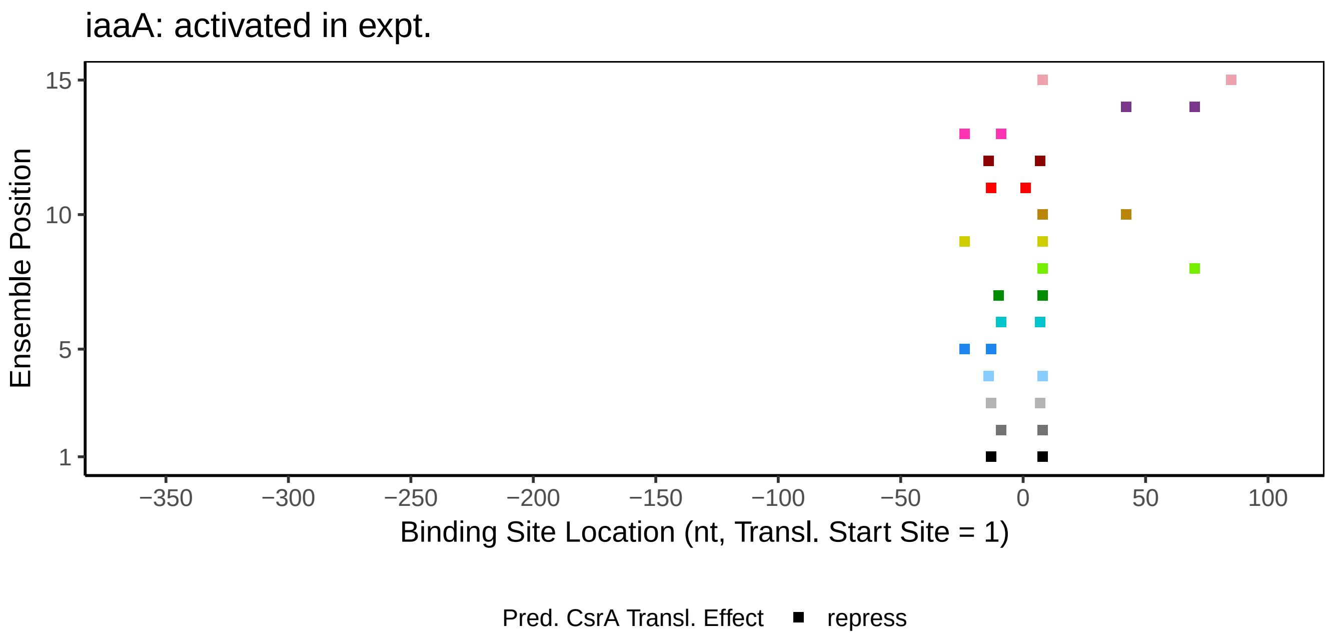

iaaA: activated in expt.
Ensemble Position
15
10
5
1
−350
−300
−250
−200
−150
−100
−50
0
50
100
Binding Site Location (nt, Transl. Start Site = 1)
Pred. CsrA Transl. Effect
repress

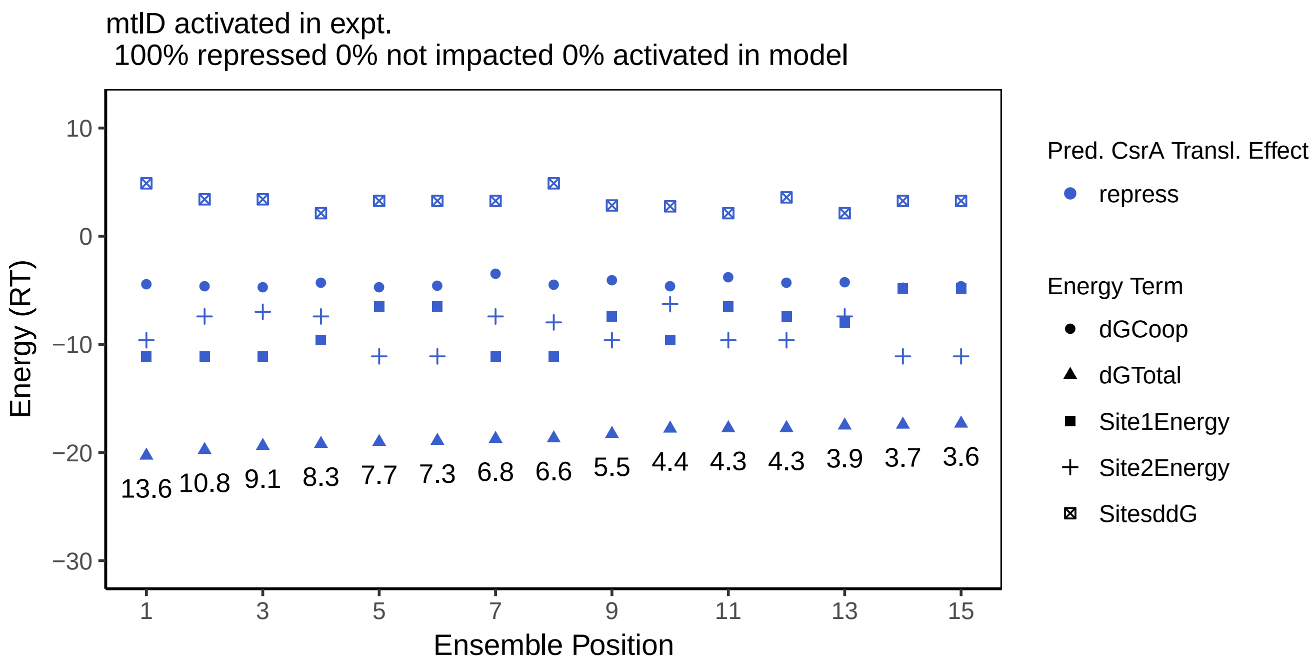
mtlD activated in expt.
100% repressed 0% not impacted 0% activated in model
Energy (RT)
Ensemble Position
13.6 10.8 9.1 8.3 7.7 7.3 6.8 6.6 5.5 4.4 4.3 4.3 3.9 3.7 3.6
Pred. CsrA Transl. Effect
repress
Energy Term
dGCoop
dGTotal
Site1Energy
Site2Energy
SitesddG

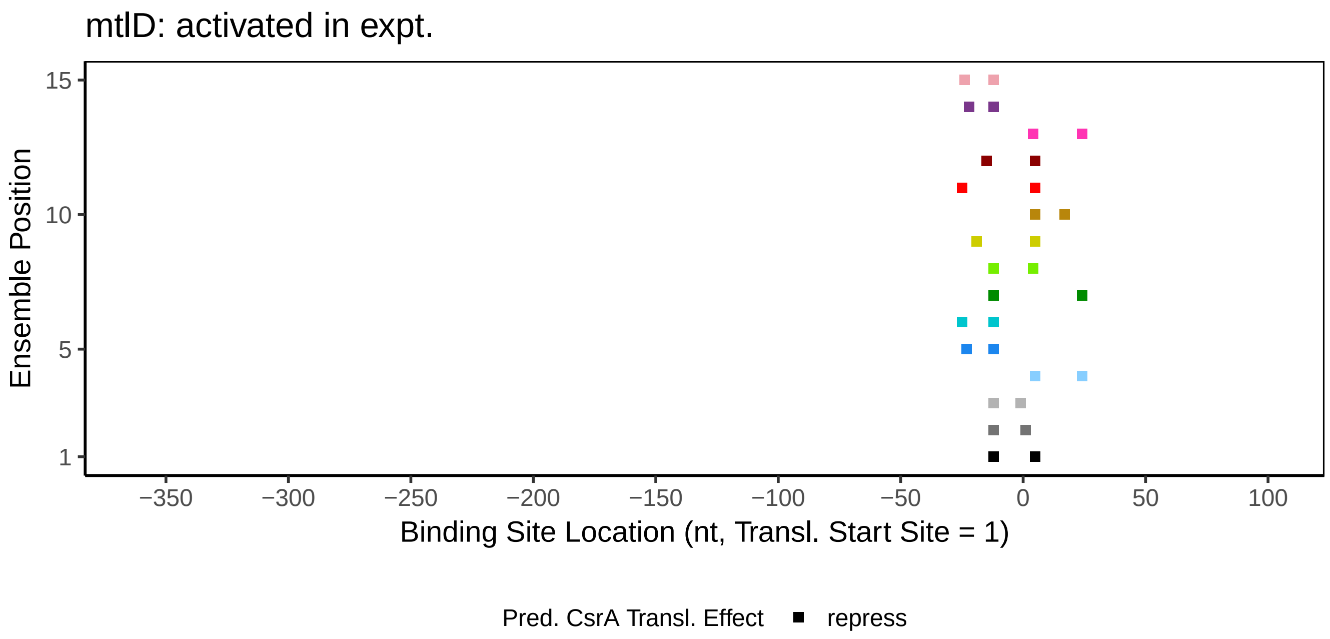
mtlD: activated in expt.
Ensemble Position
Binding Site Location (nt, Transl. Start Site = 1)
Pred. CsrA Transl. Effect
repress

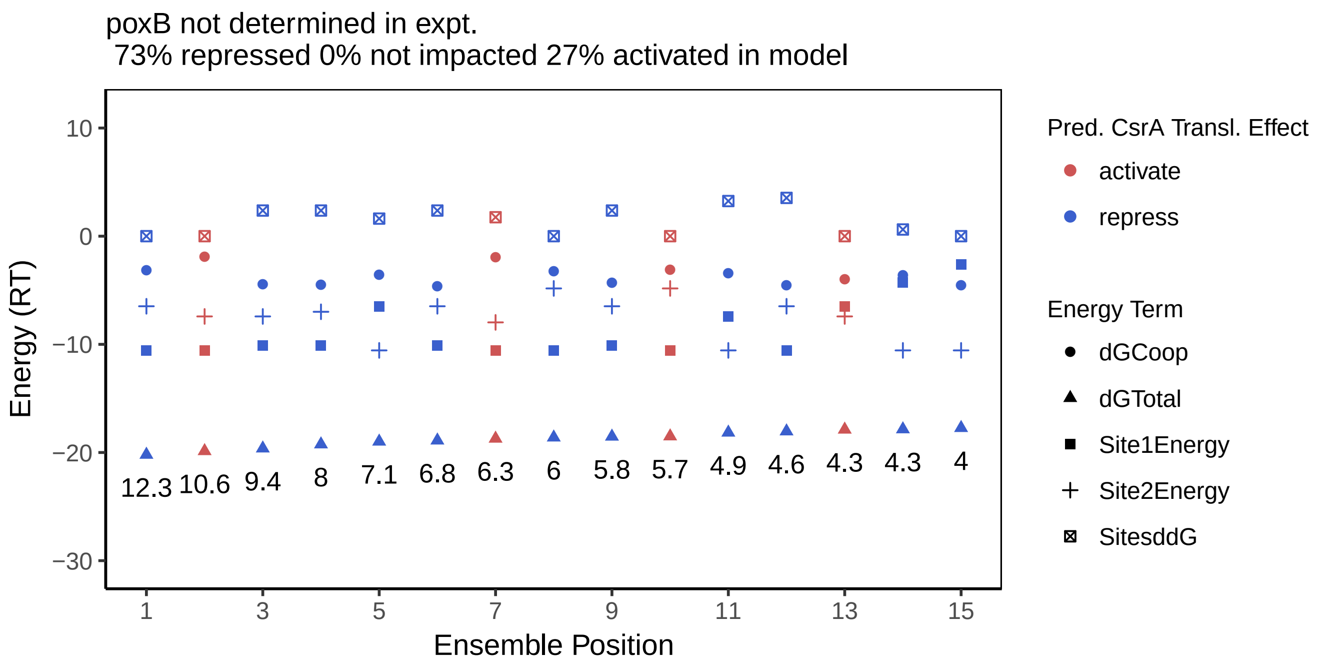
poxB not determined in expt.
73% repressed 0% not impacted 27% activated in model
Energy (RT)
10
0
−10
−20
−30
12.3 10.6 9.4 8 7.1 6.8 6.3 6 5.8 5.7 4.9 4.6 4.3 4.3 4
1
3
5
7
9
11
13
15
Ensemble Position
Pred. CsrA Transl. Effect
activate
repress
Energy Term
dGCoop
dGTotal
Site1Energy
Site2Energy
SitesddG

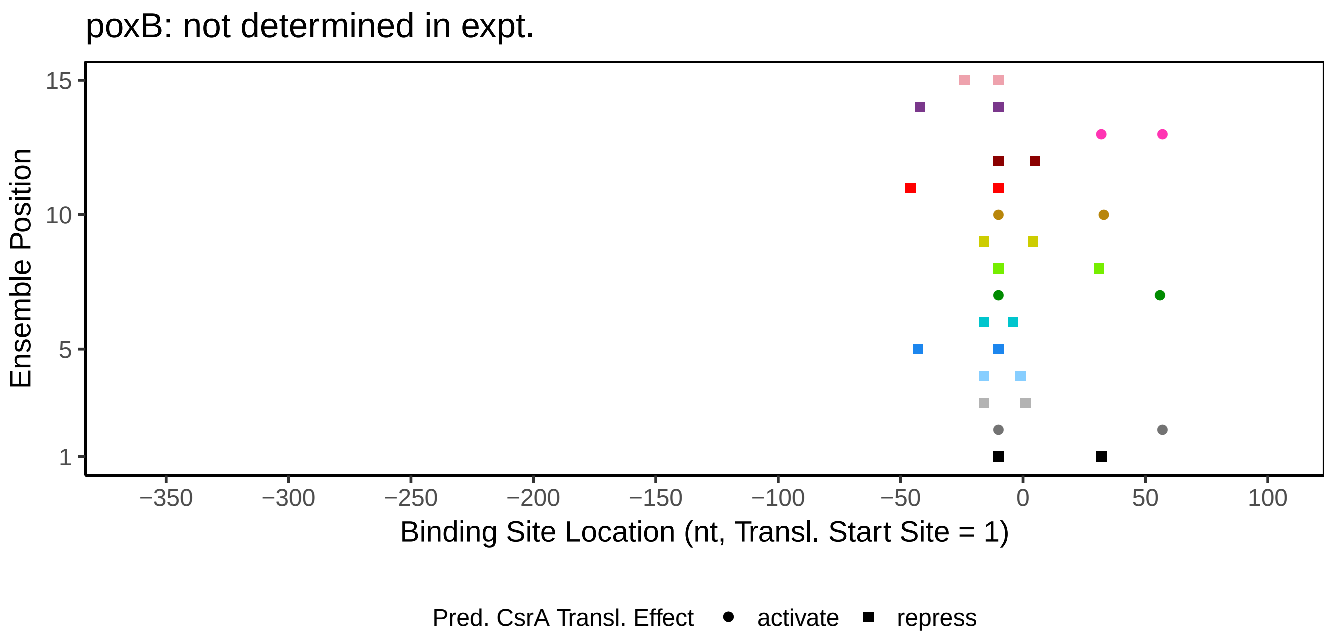
poxB: not determined in expt.
Ensemble Position
15
10
5
1
−350
−300
−250
−200
−150
−100
−50
0
50
100
Binding Site Location (nt, Transl. Start Site = 1)
Pred. CsrA Transl. Effect
activate
repress

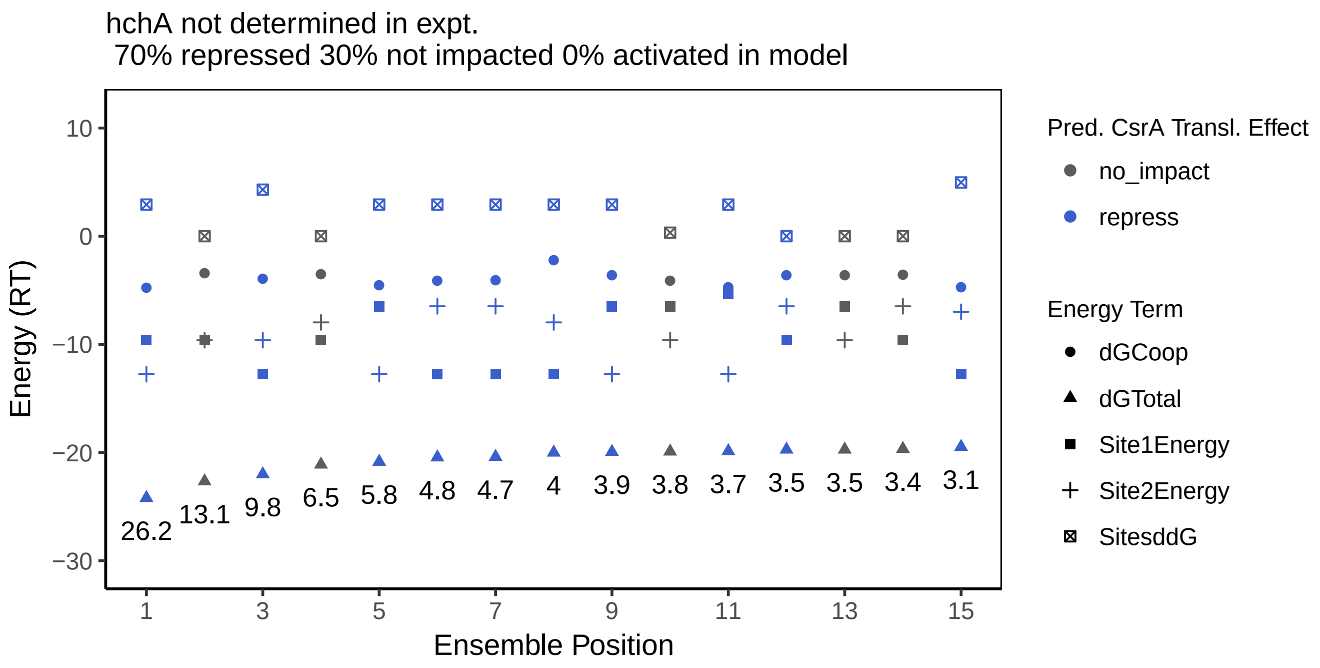

hchA not determined in expt.
70% repressed 30% not impacted 0% activated in model
Energy (RT)
Ensemble Position
26.2
13.1
9.8
6.5
5.8
4.8
4.7
4
3.9
3.8
3.7
3.5
3.5
3.4
3.1
Pred. CsrA Transl. Effect
no_impact
repress
Energy Term
dGCoop
dGTotal
Site1Energy
Site2Energy
SitesddG

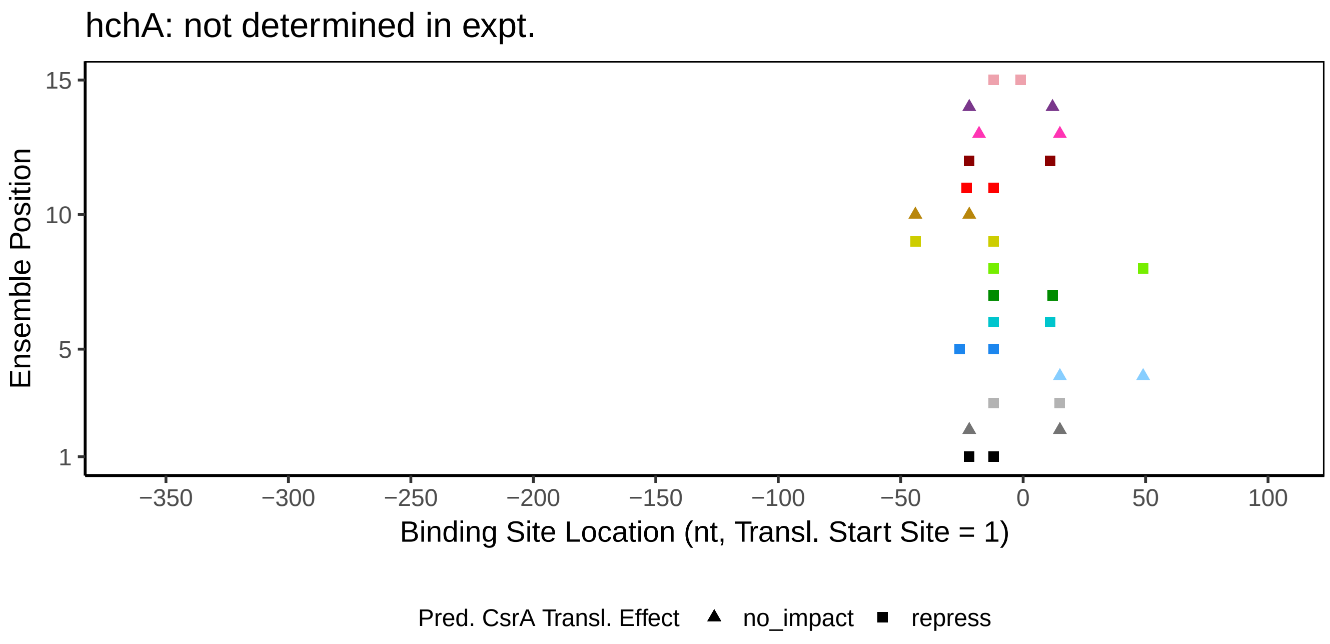

hchA: not determined in expt.
Ensemble Position
Binding Site Location (nt, Transl. Start Site = 1)
Pred. CsrA Transl. Effect
no_impact
repress

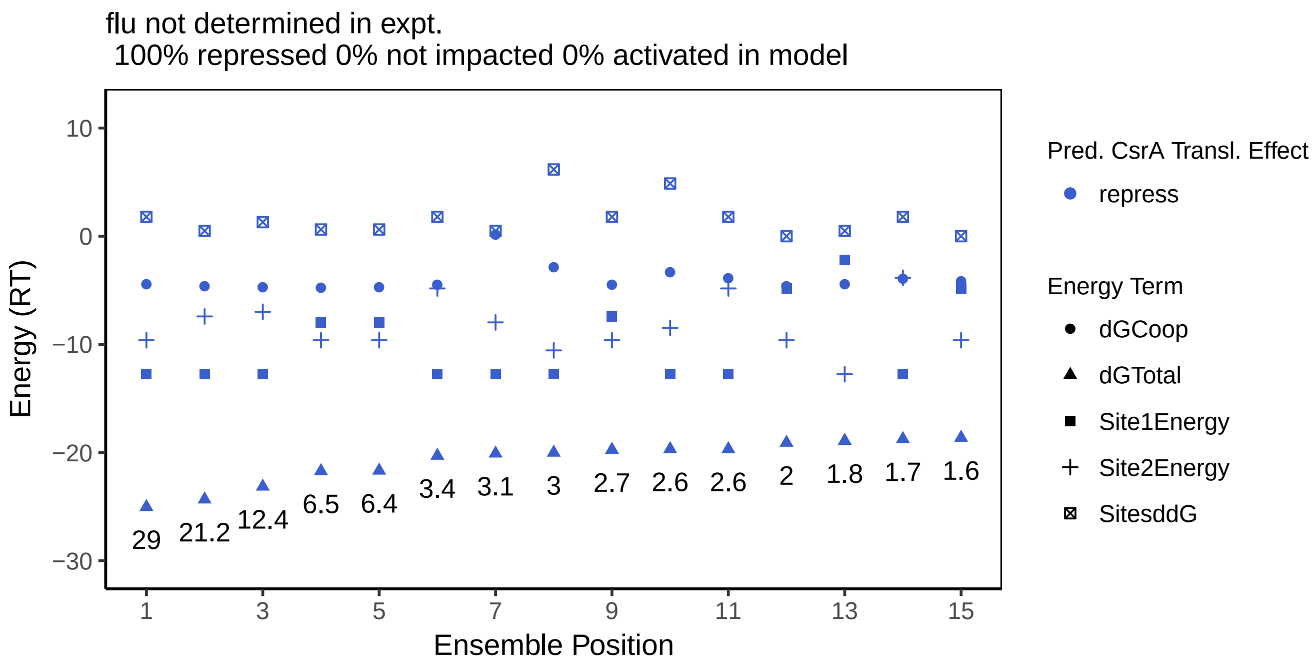
flu not determined in expt.
100% repressed 0% not impacted 0% activated in model
Energy (RT)
10
0
−10
−20
−30
29
21.2
12.4
6.5
6.4
3.4
3.1
3
2.7
2.6
2.6
2
1.8
1.7
1.6
1
3
5
7
9
11
13
15
Ensemble Position
Pred. CsrA Transl. Effect
repress
Energy Term
dGCoop
dGTotal
Site1Energy
Site2Energy
SitesddG

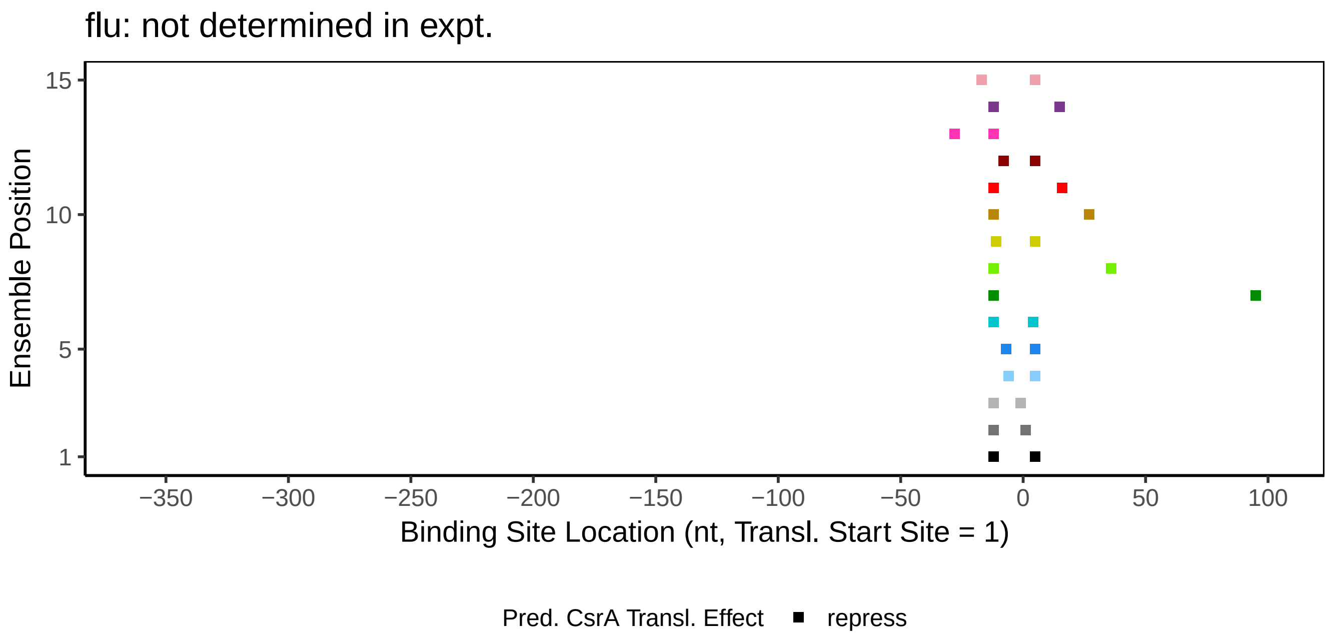
flu: not determined in expt.
Ensemble Position
15
10
5
1
−350
−300
−250
−200
−150
−100
−50
0
50
100
Binding Site Location (nt, Transl. Start Site = 1)
Pred. CsrA Transl. Effect
repress

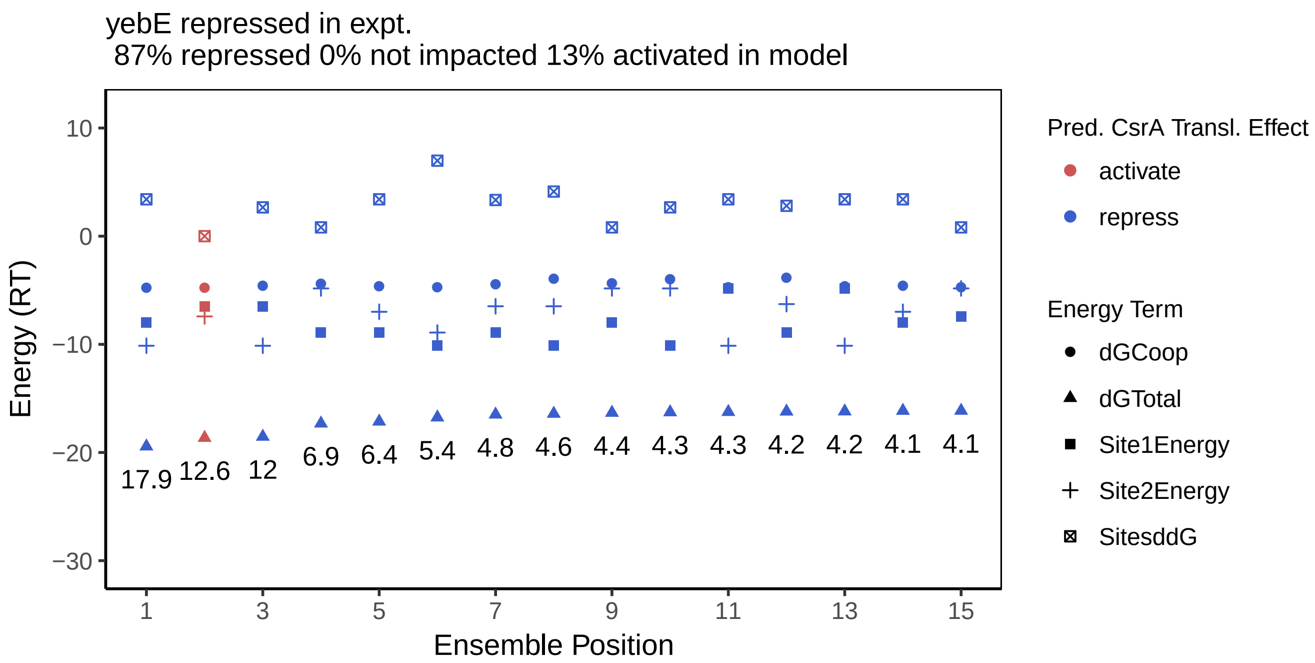

yebE repressed in expt.
87% repressed 0% not impacted 13% activated in model
Energy (RT)
Ensemble Position
17.9
12.6
12
6.9
6.4
5.4
4.8
4.6
4.4
4.3
4.3
4.2
4.2
4.1
4.1
Pred. CsrA Transl. Effect
activate
repress
Energy Term
dGCoop
dGTotal
Site1Energy
Site2Energy
SitesddG

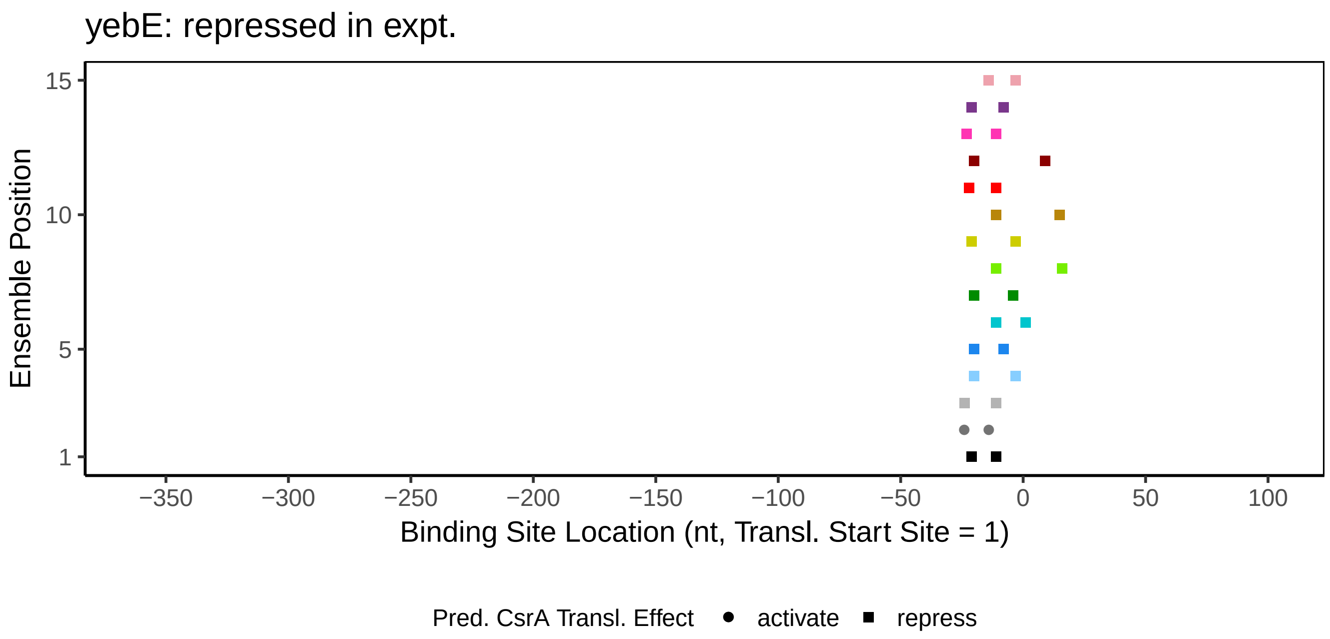

yebE: repressed in expt.
Ensemble Position
Binding Site Location (nt, Transl. Start Site = 1)
Pred. CsrA Transl. Effect
activate
repress

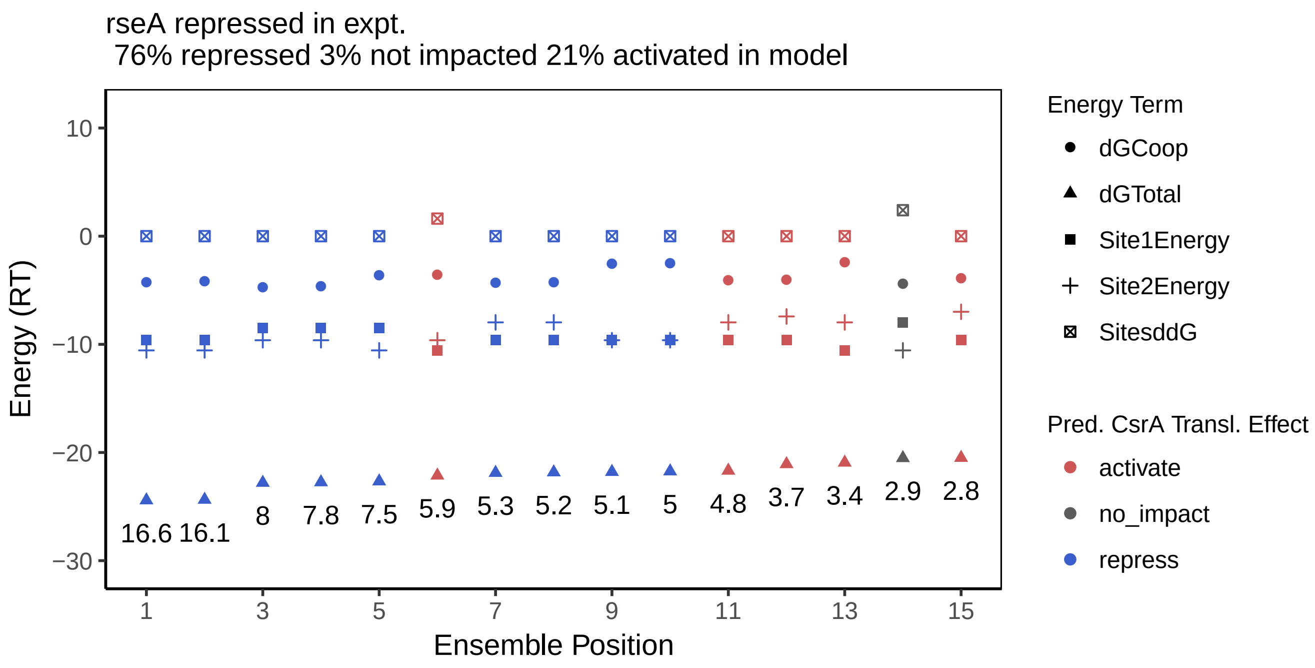

rseA repressed in expt.
76% repressed 3% not impacted 21% activated in model
Energy (RT)
Ensemble Position
16.6 16.1 8 7.8 7.5 5.9 5.3 5.2 5.1 5 4.8 3.7 3.4 2.9 2.8
Energy Term
dGCoop
dGTotal
Site1Energy
Site2Energy
SitesddG
Pred. CsrA Transl. Effect
activate
no_impact
repress

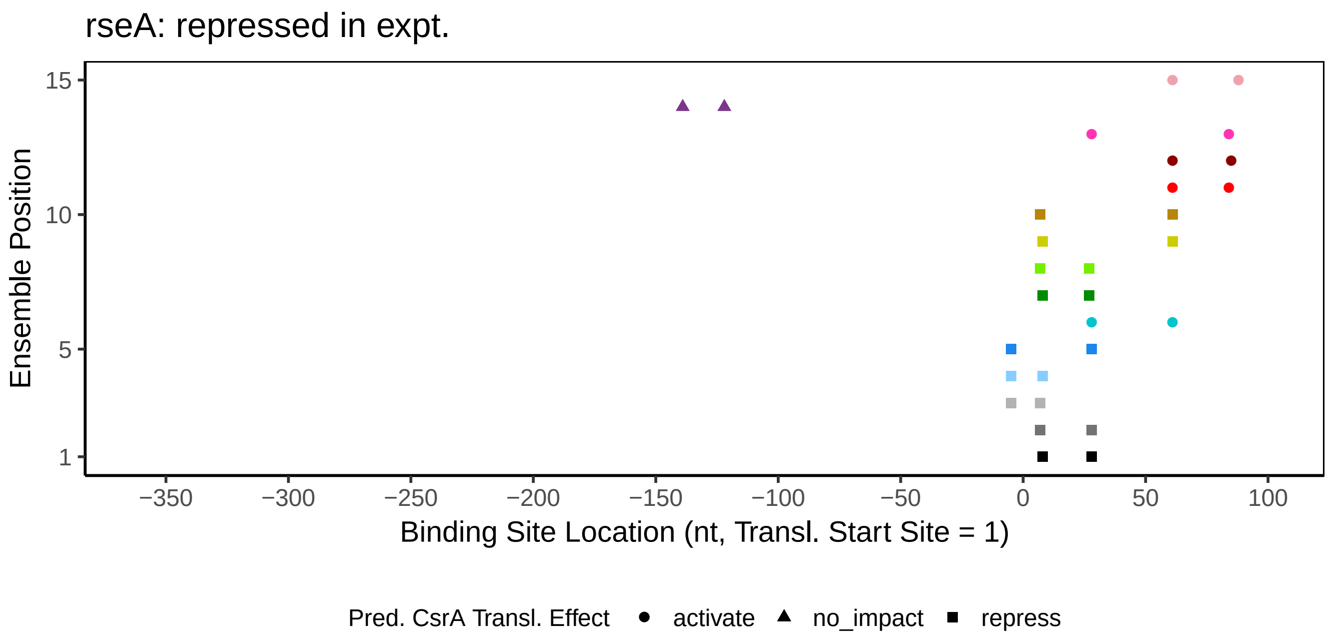

rseA: repressed in expt.
Ensemble Position
Binding Site Location (nt, Transl. Start Site = 1)
Pred. CsrA Transl. Effect
activate
no_impact
repress

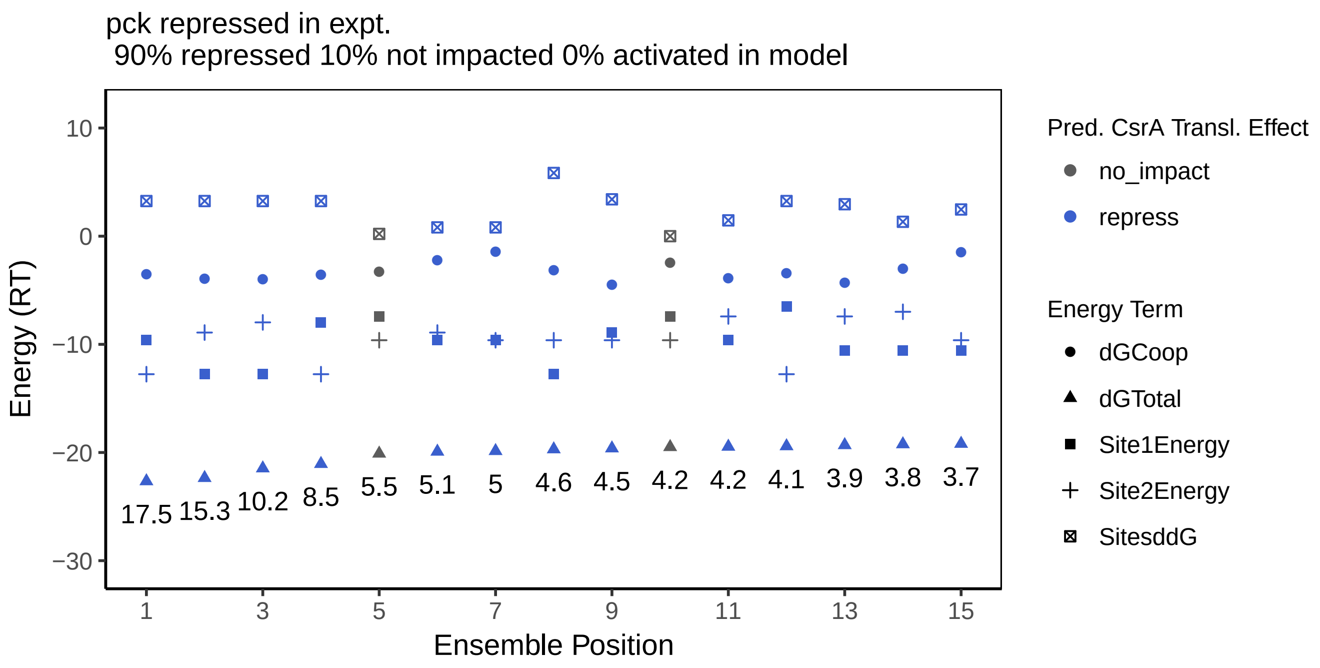

pck repressed in expt.
90% repressed 10% not impacted 0% activated in model
Energy (RT)
Ensemble Position
17.5 15.3 10.2 8.5 5.5 5.1 5 4.6 4.5 4.2 4.2 4.1 3.9 3.8 3.7
Pred. CsrA Transl. Effect
no_impact
repress
Energy Term
dGCoop
dGTotal
Site1Energy
Site2Energy
SitesddG

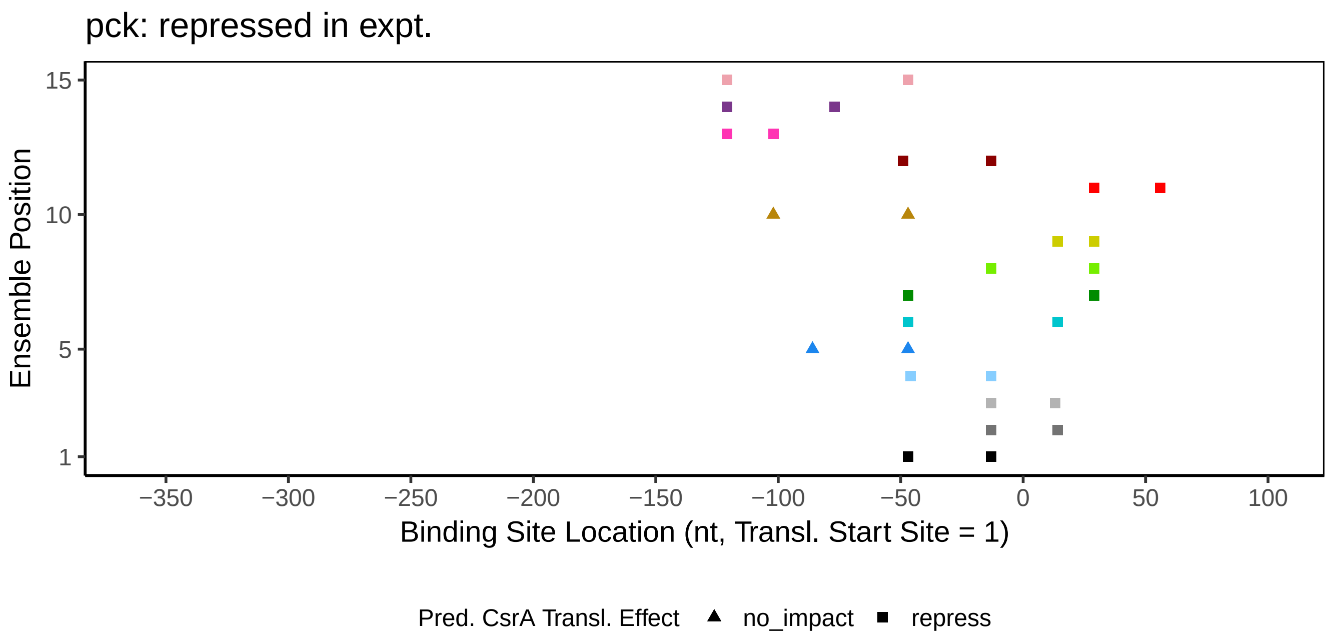

pck: repressed in expt.
Ensemble Position
Binding Site Location (nt, Transl. Start Site = 1)
Pred. CsrA Transl. Effect
no_impact
repress

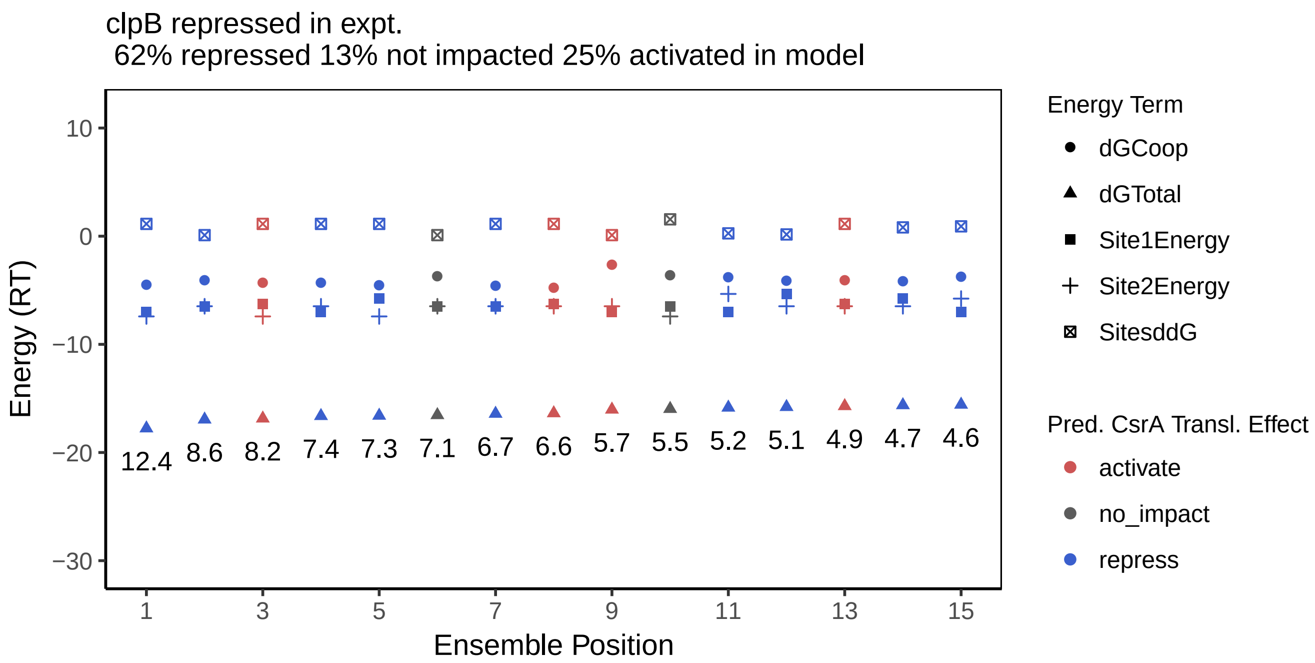
clpB repressed in expt.
62% repressed 13% not impacted 25% activated in model
Energy (RT)
10
0
−10
−20
−30
12.4 8.6 8.2 7.4 7.3 7.1 6.7 6.6 5.7 5.5 5.2 5.1 4.9 4.7 4.6
1 3 5 7 9 11 13 15
Ensemble Position
Energy Term
dGCoop
dGTotal
Site1Energy
Site2Energy
SitesddG
Pred. CsrA Transl. Effect
activate
no_impact
repress

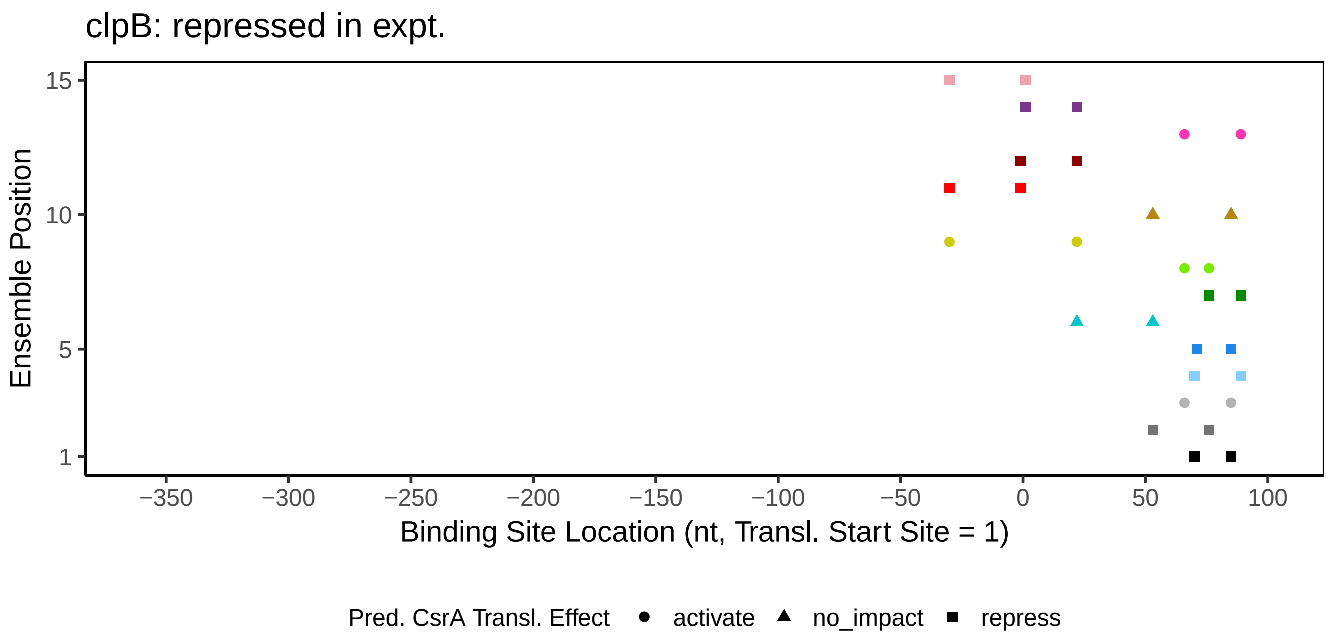
clpB: repressed in expt.
Ensemble Position
15
10
5
1
−350 −300 −250 −200 −150 −100 −50 0 50 100
Binding Site Location (nt, Transl. Start Site = 1)
Pred. CsrA Transl. Effect
activate
no_impact
repress

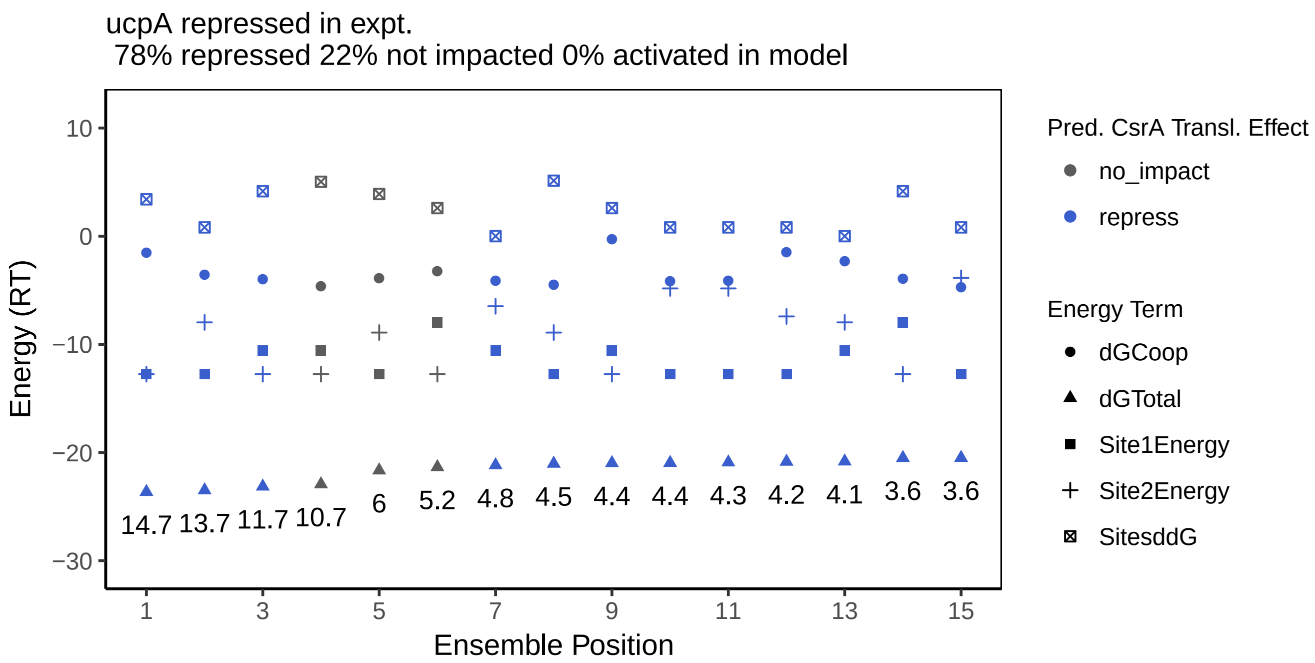

ucpA repressed in expt.
78% repressed 22% not impacted 0% activated in model
Energy (RT)
10
0
−10
−20
−30
14.7 13.7 11.7 10.7 6 5.2 4.8 4.5 4.4 4.4 4.3 4.2 4.1 3.6 3.6
1 3 5 7 9 11 13 15
Ensemble Position
Pred. CsrA Transl. Effect
no_impact
repress
Energy Term
dGCoop
dGTotal
Site1Energy
Site2Energy
SitesddG

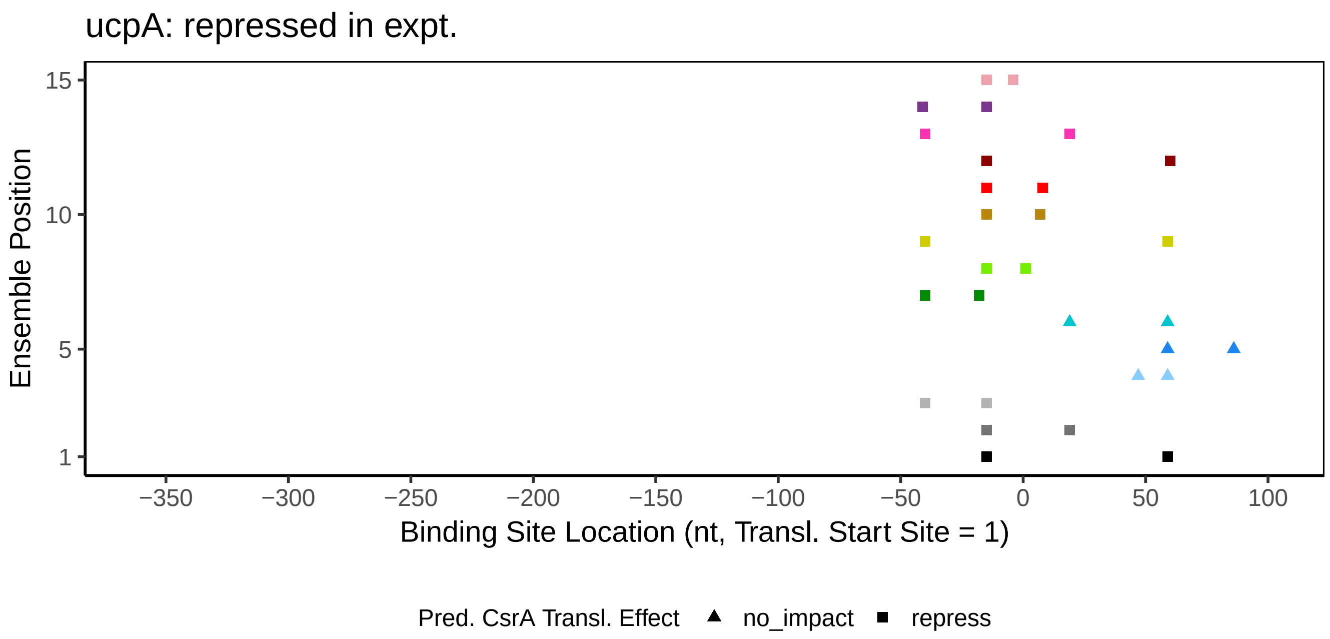

ucpA: repressed in expt.
Ensemble Position
15
10
5
1
−350 −300 −250 −200 −150 −100 −50 0 50 100
Binding Site Location (nt, Transl. Start Site = 1)
Pred. CsrA Transl. Effect
no_impact
repress

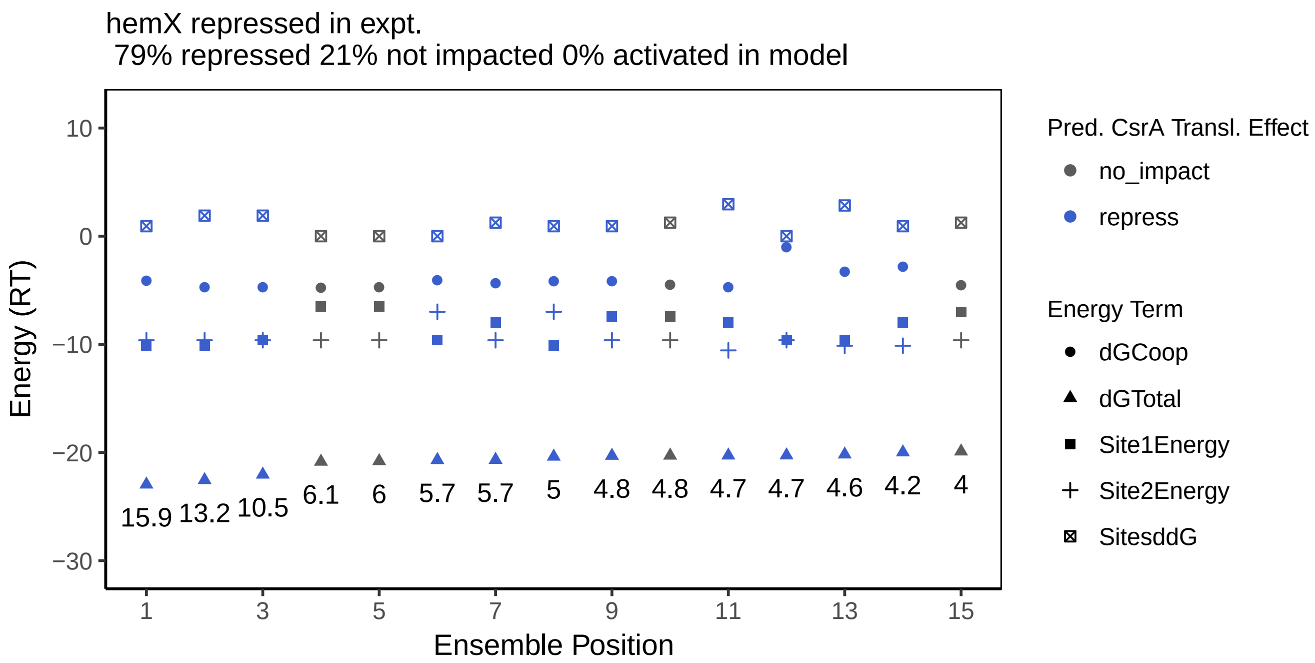
hemX repressed in expt.
79% repressed 21% not impacted 0% activated in model
Energy (RT)
10
0
−10
−20
−30
15.9 13.2 10.5 6.1 6 5.7 5.7 5 4.8 4.8 4.7 4.7 4.6 4.2 4
1 3 5 7 9 11 13 15
Ensemble Position
Pred. CsrA Transl. Effect
no_impact
repress
Energy Term
dGCoop
dGTotal
Site1Energy
Site2Energy
SitesddG

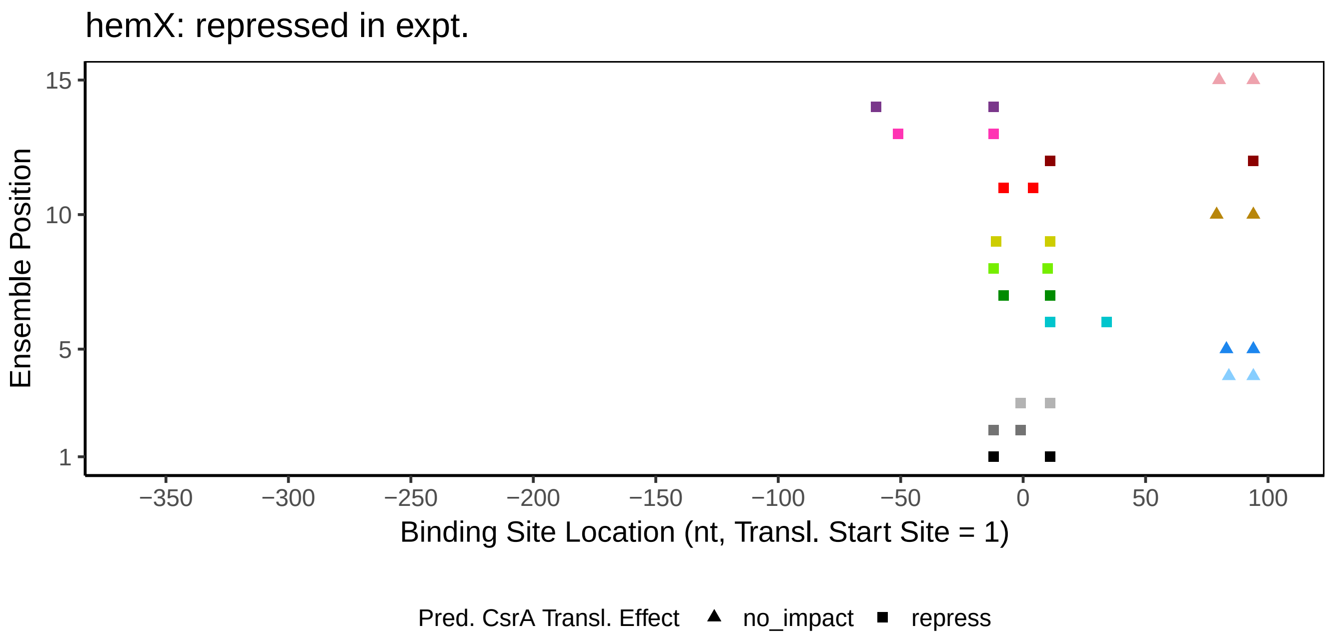
hemX: repressed in expt.
Ensemble Position
15
10
5
1
−350 −300 −250 −200 −150 −100 −50 0 50 100
Binding Site Location (nt, Transl. Start Site = 1)
Pred. CsrA Transl. Effect
no_impact
repress

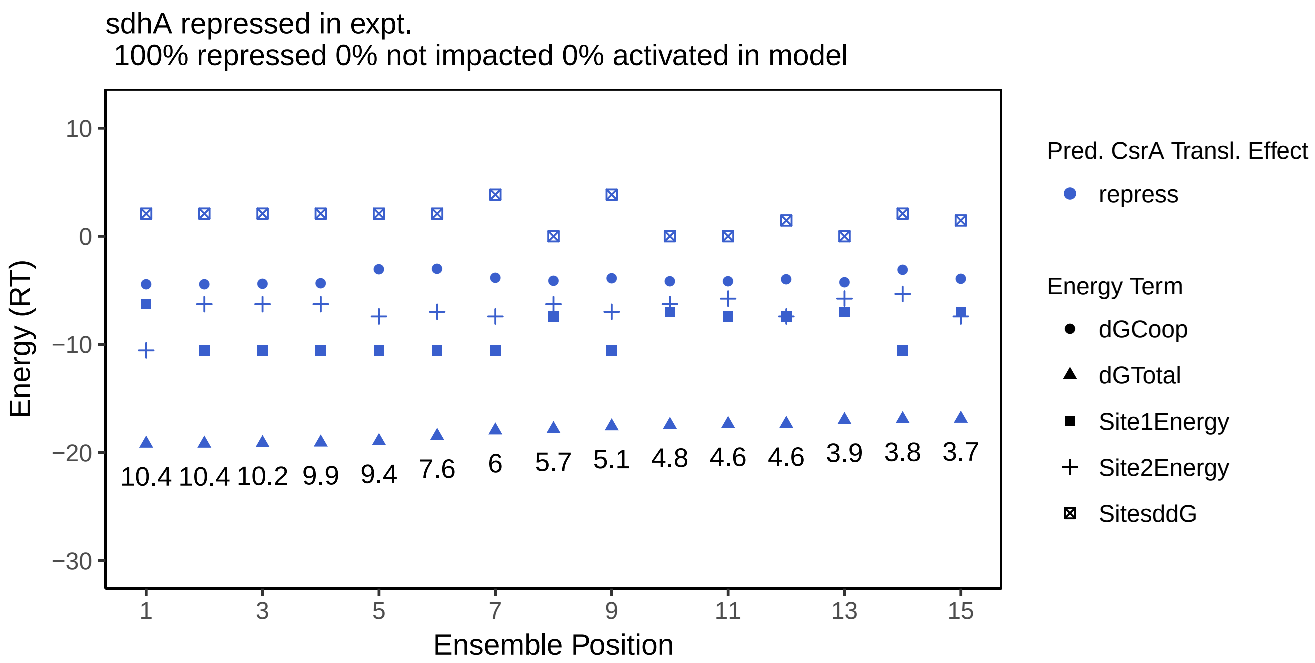
sdhA repressed in expt.
100% repressed 0% not impacted 0% activated in model
Energy (RT)
10
0
−10
−20
−30
10.4 10.4 10.2 9.9 9.4 7.6 6 5.7 5.1 4.8 4.6 4.6 3.9 3.8 3.7
1 3 5 7 9 11 13 15
Ensemble Position
Pred. CsrA Transl. Effect
repress
Energy Term
dGCoop
dGTotal
Site1Energy
Site2Energy
SitesddG

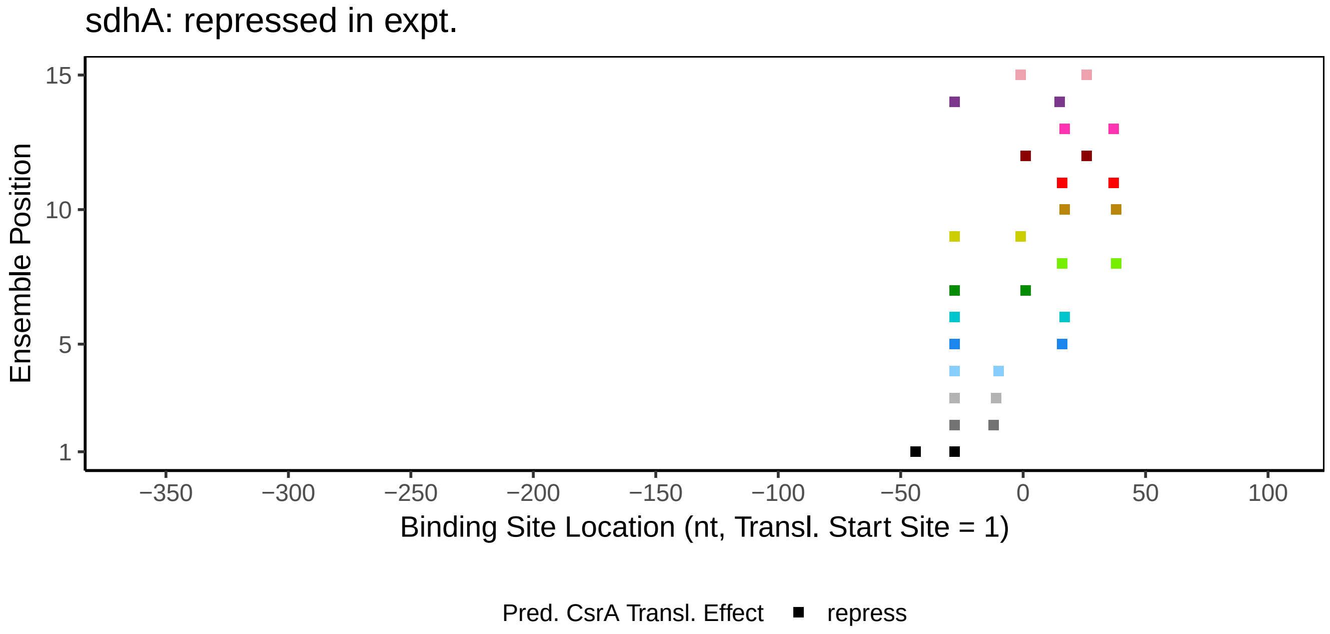
sdhA: repressed in expt.
Ensemble Position
15
10
5
1
−350 −300 −250 −200 −150 −100 −50 0 50 100
Binding Site Location (nt, Transl. Start Site = 1)
Pred. CsrA Transl. Effect
repress

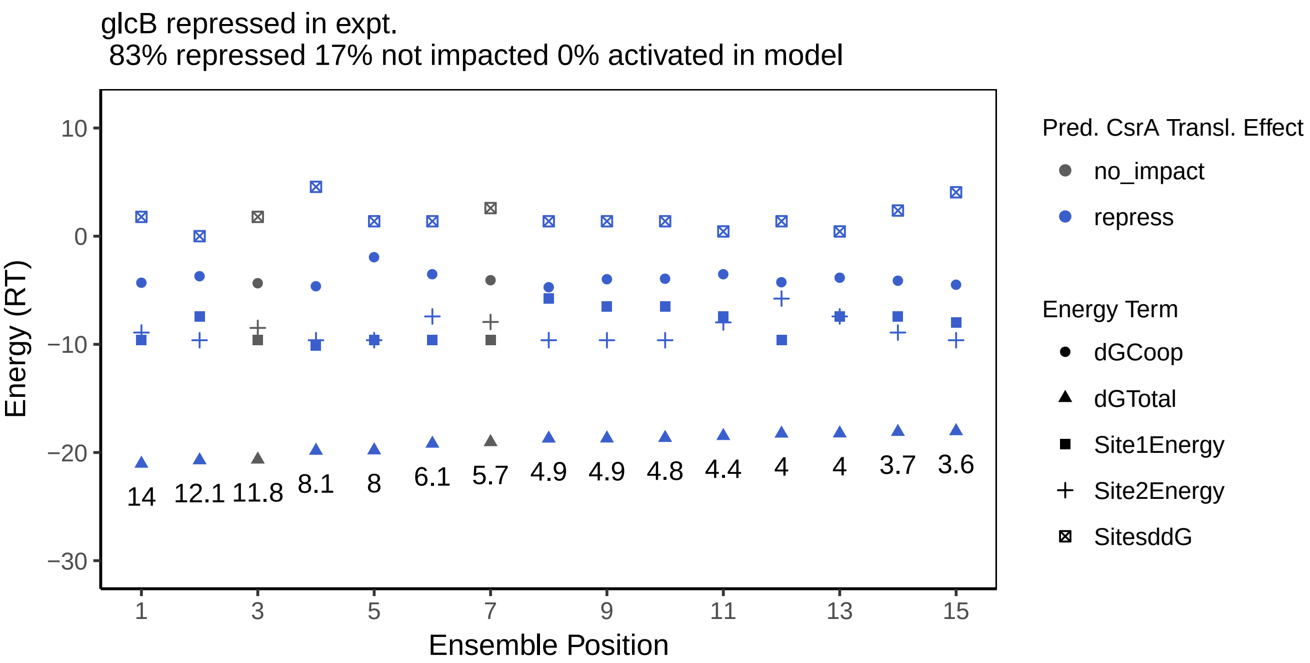

glcB repressed in expt.
83% repressed 17% not impacted 0% activated in model
Energy (RT)
10
0
−10
−20
−30
14
12.1
11.8
8.1
8
6.1
5.7
4.9
4.9
4.8
4.4
4
4
3.7
3.6
1
3
5
7
9
11
13
15
Ensemble Position
Pred. CsrA Transl. Effect
no_impact
repress
Energy Term
dGCoop
dGTotal
Site1Energy
Site2Energy
SitesddG

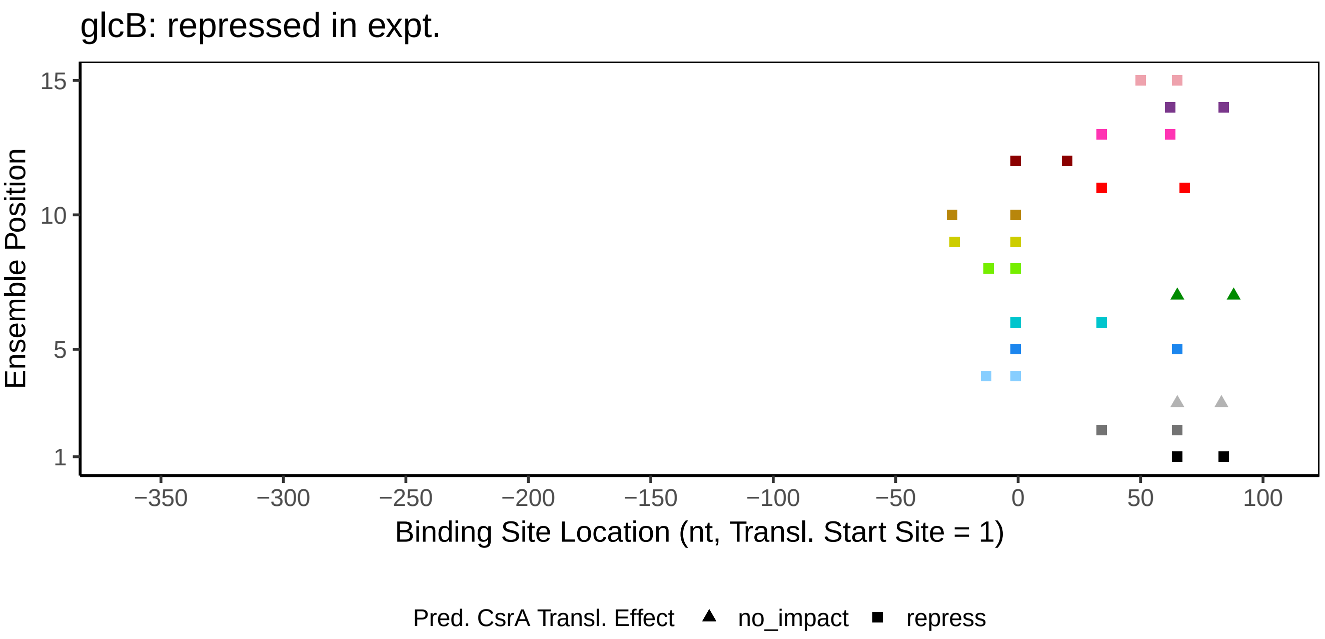

glcB: repressed in expt.
Ensemble Position
15
10
5
1
−350
−300
−250
−200
−150
−100
−50
0
50
100
Binding Site Location (nt, Transl. Start Site = 1)
Pred. CsrA Transl. Effect
no_impact
repress

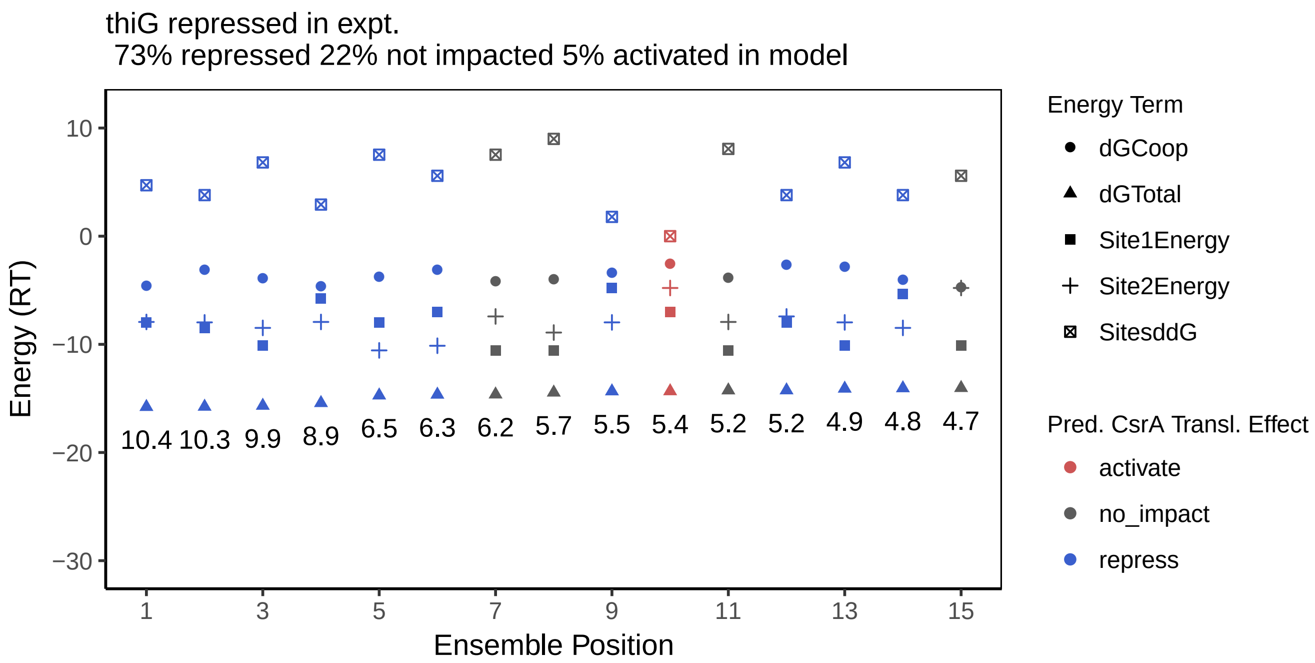
thiG repressed in expt.
73% repressed 22% not impacted 5% activated in model
Energy (RT)
Ensemble Position
10.4 10.3 9.9 8.9 6.5 6.3 6.2 5.7 5.5 5.4 5.2 5.2 4.9 4.8 4.7
Energy Term
dGCoop
dGTotal
Site1Energy
Site2Energy
SitesddG
Pred. CsrA Transl. Effect
activate
no_impact
repress

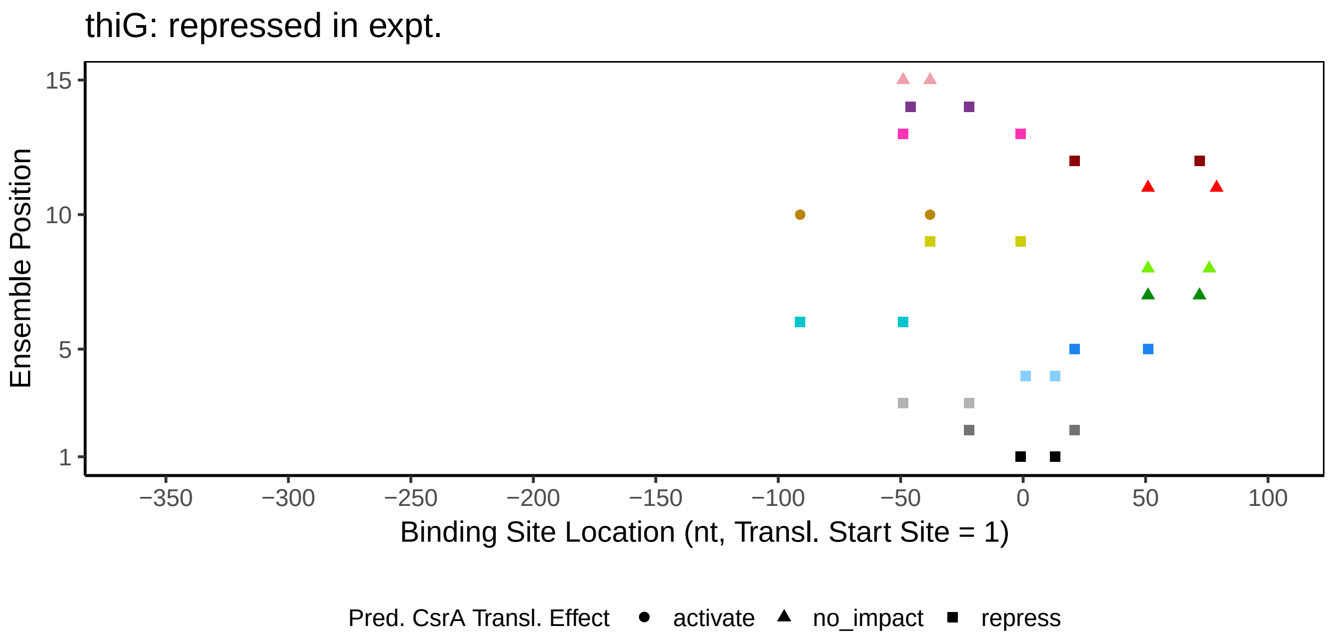
thiG: repressed in expt.
Ensemble Position
Binding Site Location (nt, Transl. Start Site = 1)
Pred. CsrA Transl. Effect
activate
no_impact
repress

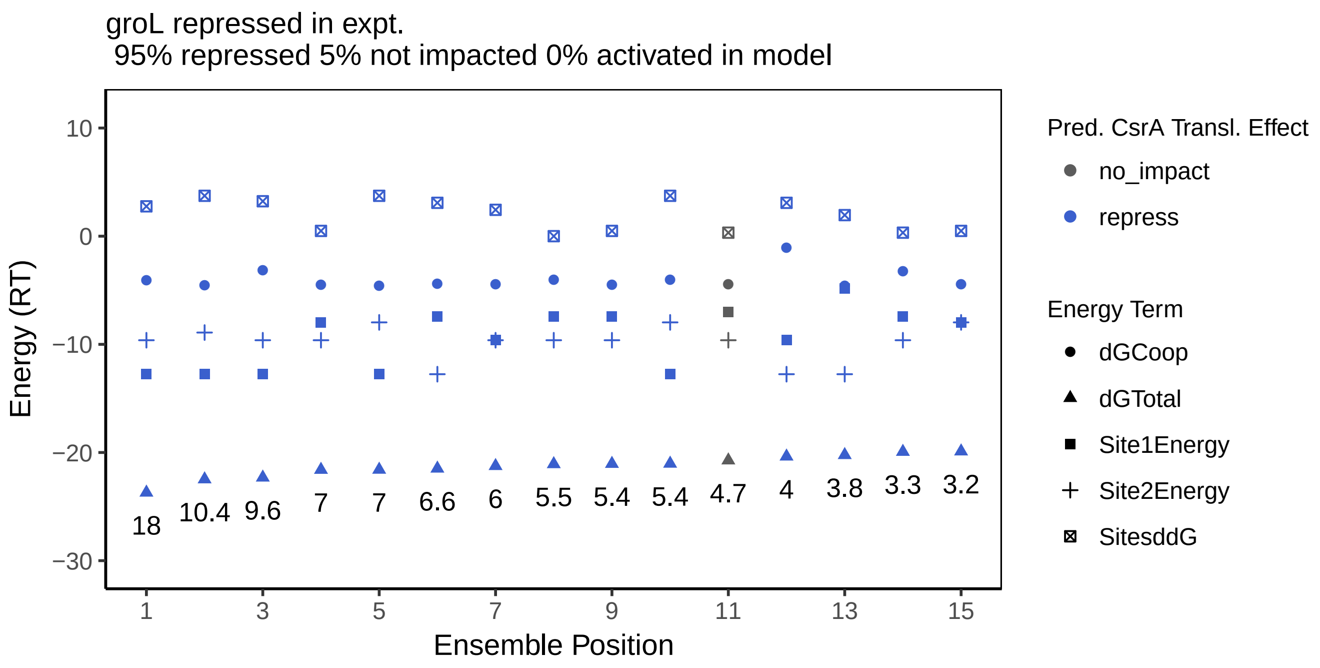
groL repressed in expt.
95% repressed 5% not impacted 0% activated in model
Energy (RT)
Ensemble Position
18 10.4 9.6 7 7 6.6 6 5.5 5.4 5.4 4.7 4 3.8 3.3 3.2
Pred. CsrA Transl. Effect
no_impact
repress
Energy Term
dGCoop
dGTotal
Site1Energy
Site2Energy
SitesddG

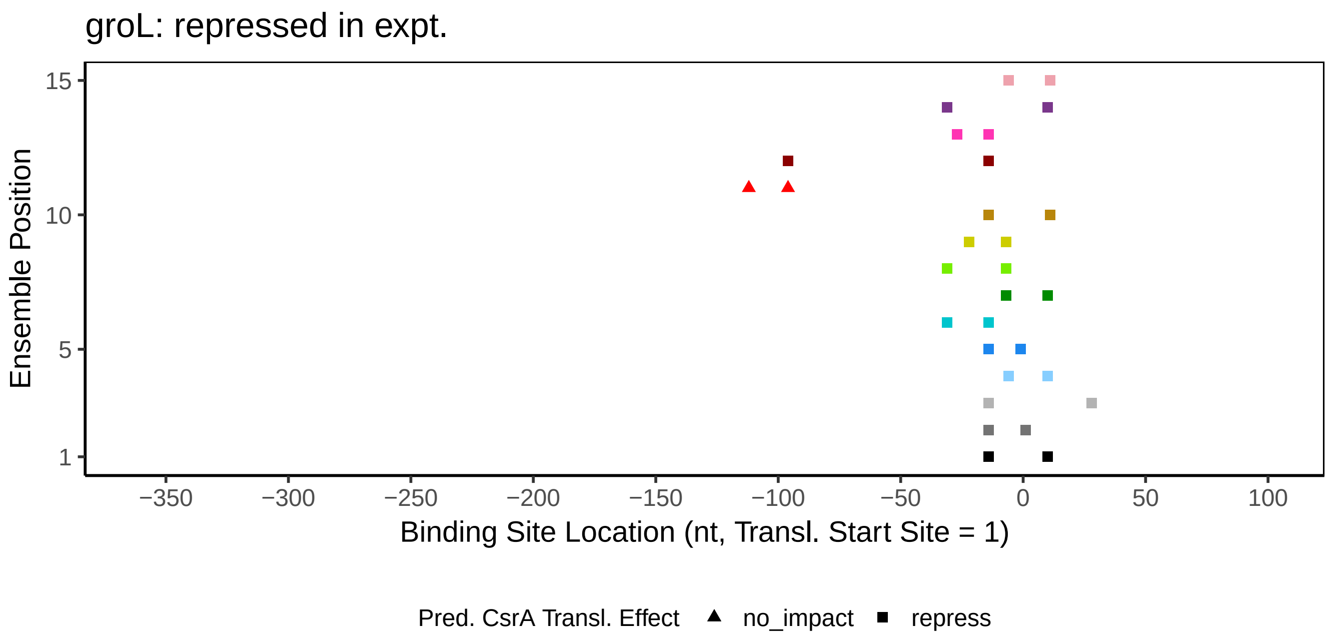
groL: repressed in expt.
Ensemble Position
Binding Site Location (nt, Transl. Start Site = 1)
Pred. CsrA Transl. Effect
no_impact
repress

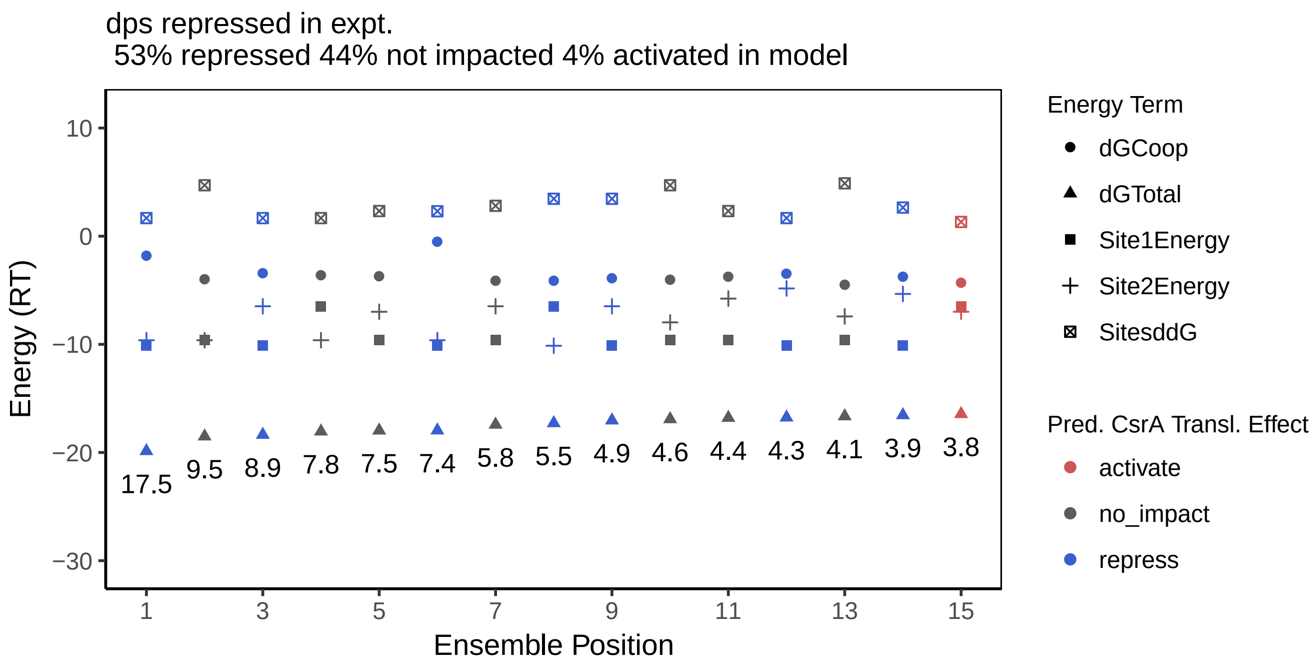

dps repressed in expt.
53% repressed 44% not impacted 4% activated in model
Energy (RT)
10
0
−10
−20
−30
17.5
9.5
8.9
7.8
7.5
7.4
5.8
5.5
4.9
4.6
4.4
4.3
4.1
3.9
3.8
1
3
5
7
9
11
13
15
Ensemble Position
Energy Term
dGCoop
dGTotal
Site1Energy
Site2Energy
SitesddG
Pred. CsrA Transl. Effect
activate
no_impact
repress

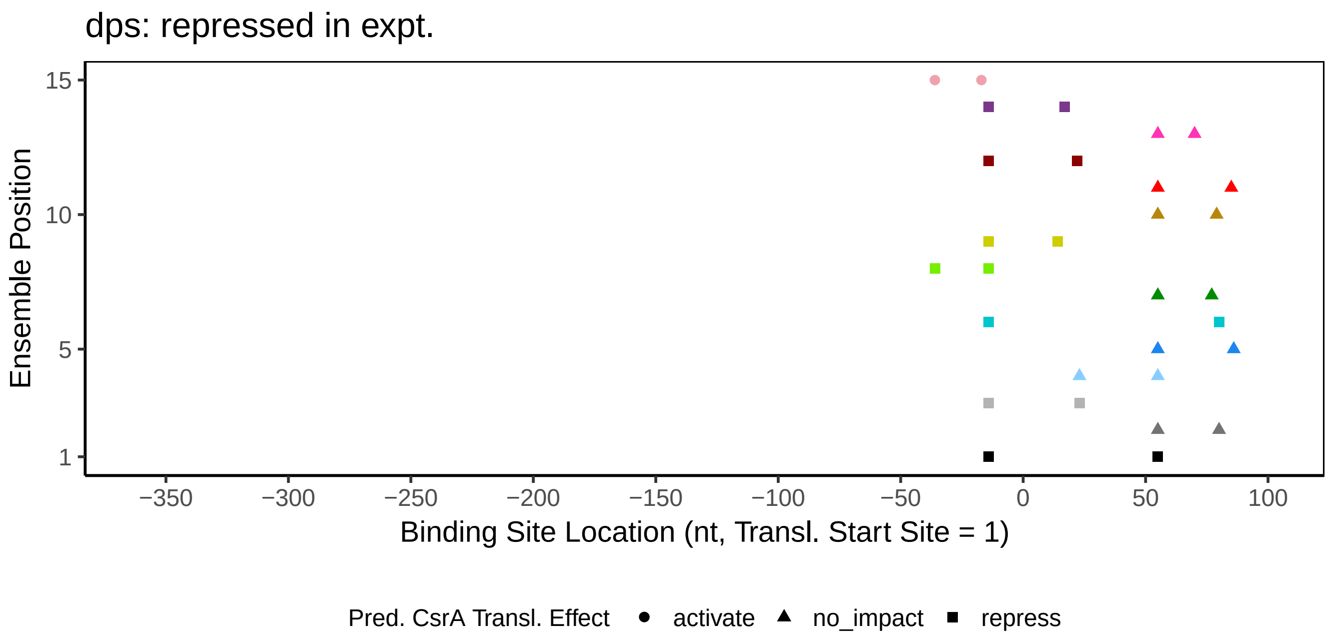

dps: repressed in expt.
Ensemble Position
15
10
5
1
−350
−300
−250
−200
−150
−100
−50
0
50
100
Binding Site Location (nt, Transl. Start Site = 1)
Pred. CsrA Transl. Effect
activate
no_impact
repress

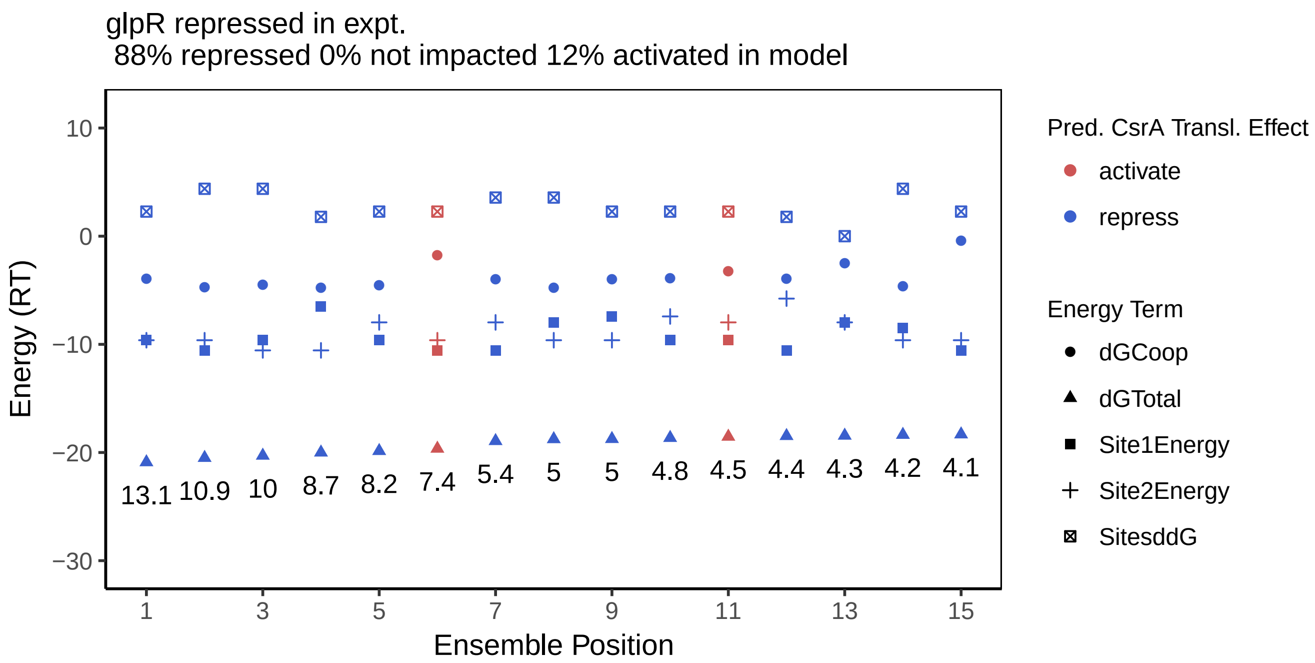

glpR repressed in expt.
88% repressed 0% not impacted 12% activated in model
Energy (RT)
10
0
−10
−20
−30
13.1 10.9 10 8.7 8.2 7.4 5.4 5 5 4.8 4.5 4.4 4.3 4.2 4.1
1 3 5 7 9 11 13 15
Ensemble Position
Pred. CsrA Transl. Effect
activate
repress
Energy Term
dGCoop
dGTotal
Site1Energy
Site2Energy
SitesddG

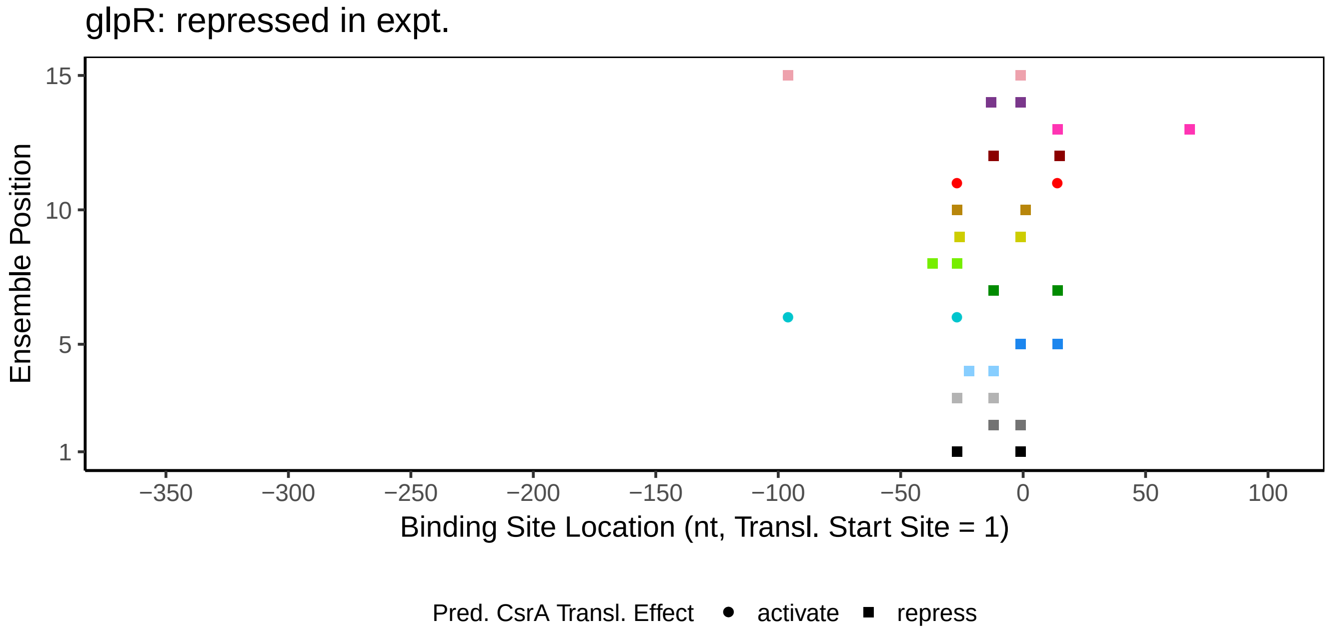

glpR: repressed in expt.
Ensemble Position
15
10
5
1
−350 −300 −250 −200 −150 −100 −50 0 50 100
Binding Site Location (nt, Transl. Start Site = 1)
Pred. CsrA Transl. Effect
activate
repress

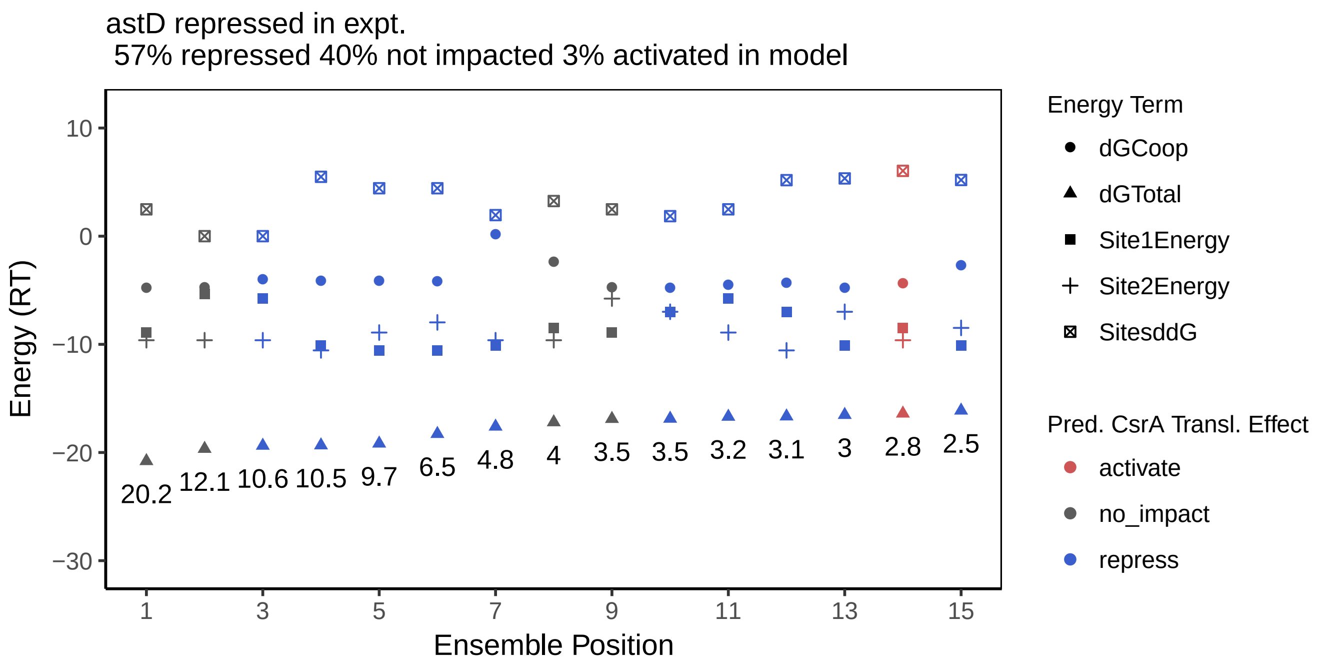

astD repressed in expt.
57% repressed 40% not impacted 3% activated in model
Energy (RT)
10
0
−10
−20
−30
20.2
12.1
10.6
10.5
9.7
6.5
4.8
4
3.5
3.5
3.2
3.1
3
2.8
2.5
1
3
5
7
9
11
13
15
Ensemble Position
Energy Term
dGCoop
dGTotal
Site1Energy
Site2Energy
SitesddG
Pred. CsrA Transl. Effect
activate
no_impact
repress

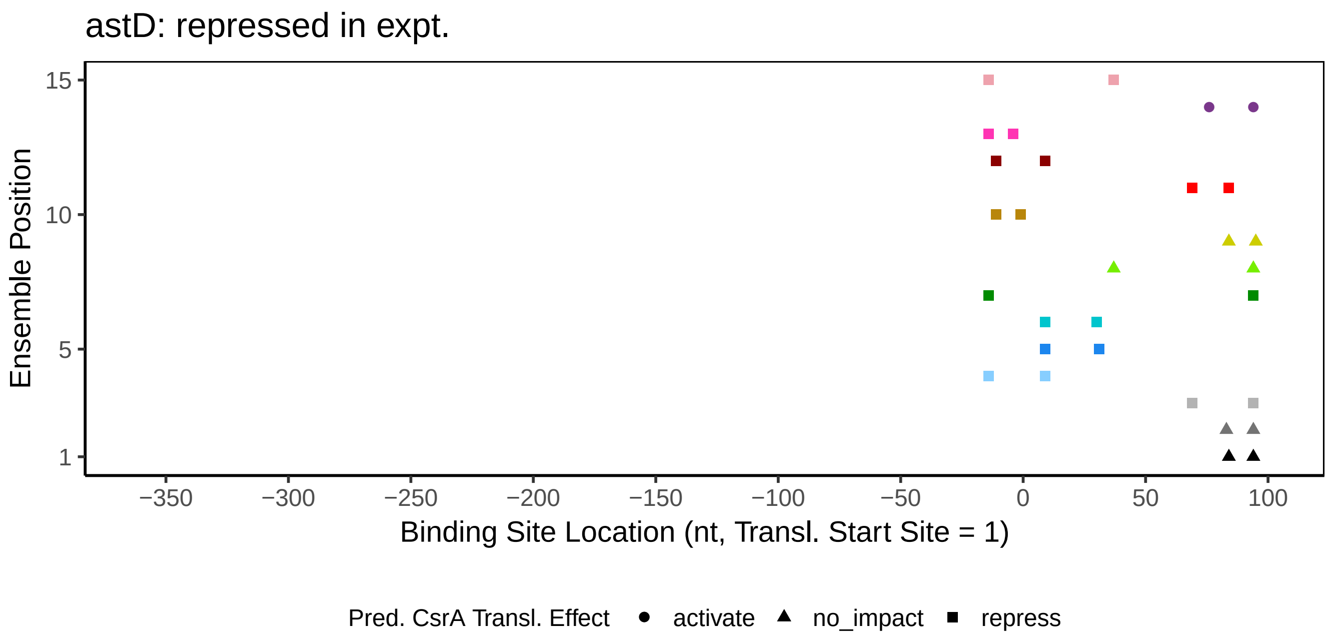

astD: repressed in expt.
Ensemble Position
15
10
5
1
−350
−300
−250
−200
−150
−100
−50
0
50
100
Binding Site Location (nt, Transl. Start Site = 1)
Pred. CsrA Transl. Effect
activate
no_impact
repress

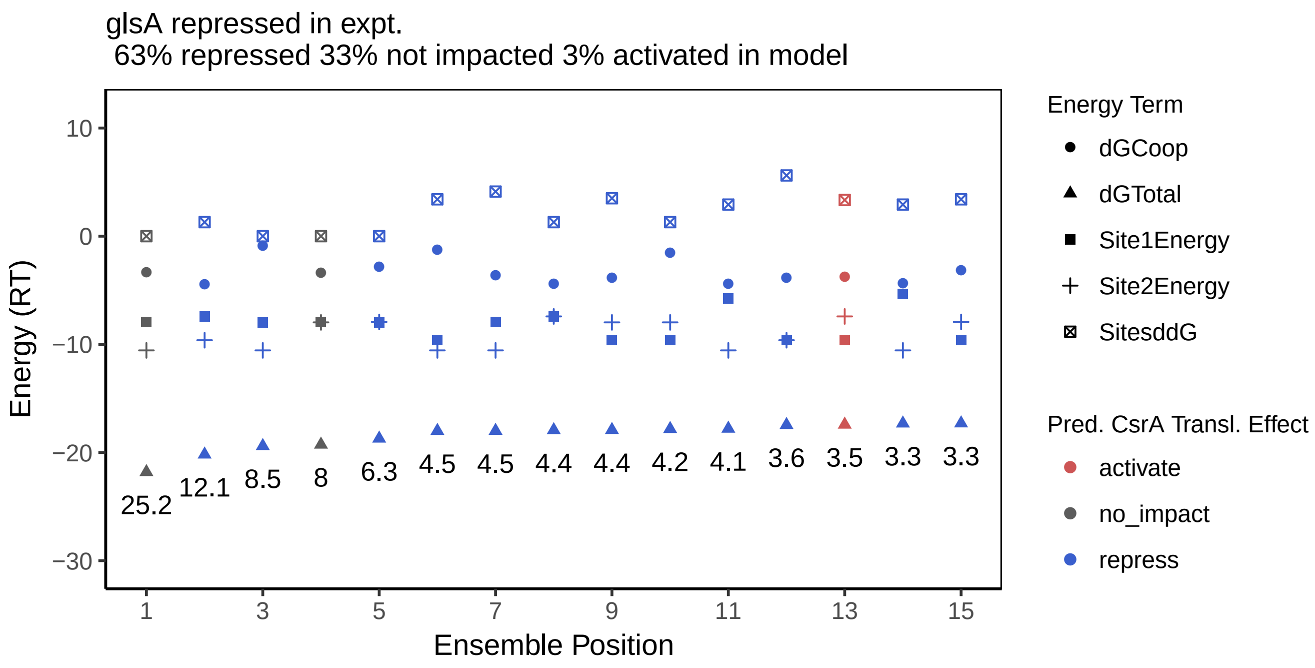

glsA repressed in expt.
63% repressed 33% not impacted 3% activated in model
Energy (RT)
10
0
−10
−20
−30
25.2
12.1
8.5
8
6.3
4.5
4.5
4.4
4.4
4.2
4.1
3.6
3.5
3.3
3.3
1
3
5
7
9
11
13
15
Ensemble Position
Energy Term
dGCoop
dGTotal
Site1Energy
Site2Energy
SitesddG
Pred. CsrA Transl. Effect
activate
no_impact
repress

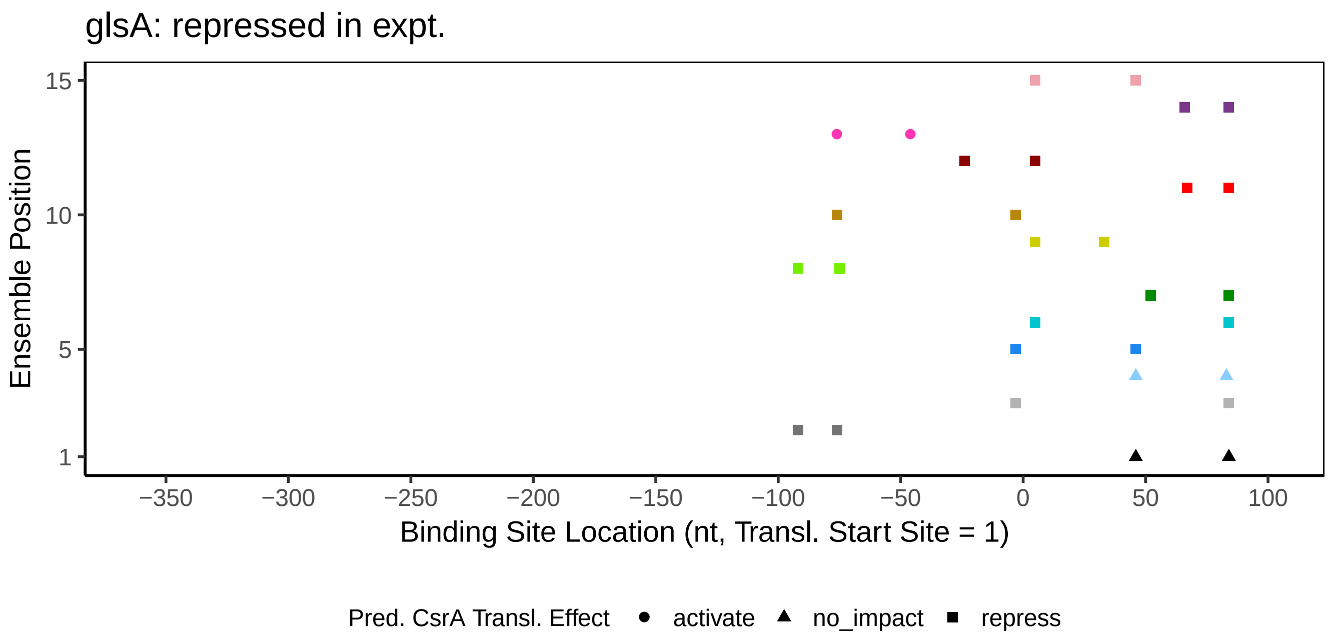

glsA: repressed in expt.
Ensemble Position
15
10
5
1
−350
−300
−250
−200
−150
−100
−50
0
50
100
Binding Site Location (nt, Transl. Start Site = 1)
Pred. CsrA Transl. Effect
activate
no_impact
repress

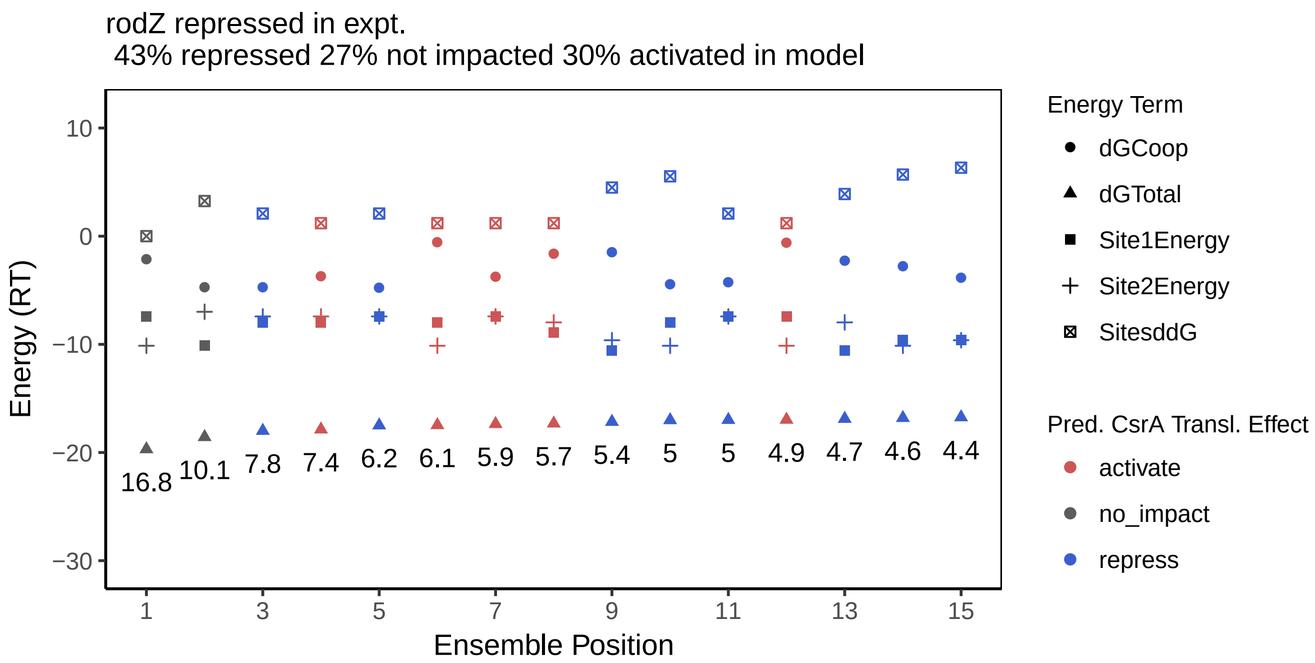
rodZ repressed in expt.
43% repressed 27% not impacted 30% activated in model
Energy (RT)
10
0
−10
−20
−30
16.8
10.1
7.8
7.4
6.2
6.1
5.9
5.7
5.4
5
5
4.9
4.7
4.6
4.4
1
3
5
7
9
11
13
15
Ensemble Position
Energy Term
dGCoop
dGTotal
Site1Energy
Site2Energy
SitesddG
Pred. CsrA Transl. Effect
activate
no_impact
repress

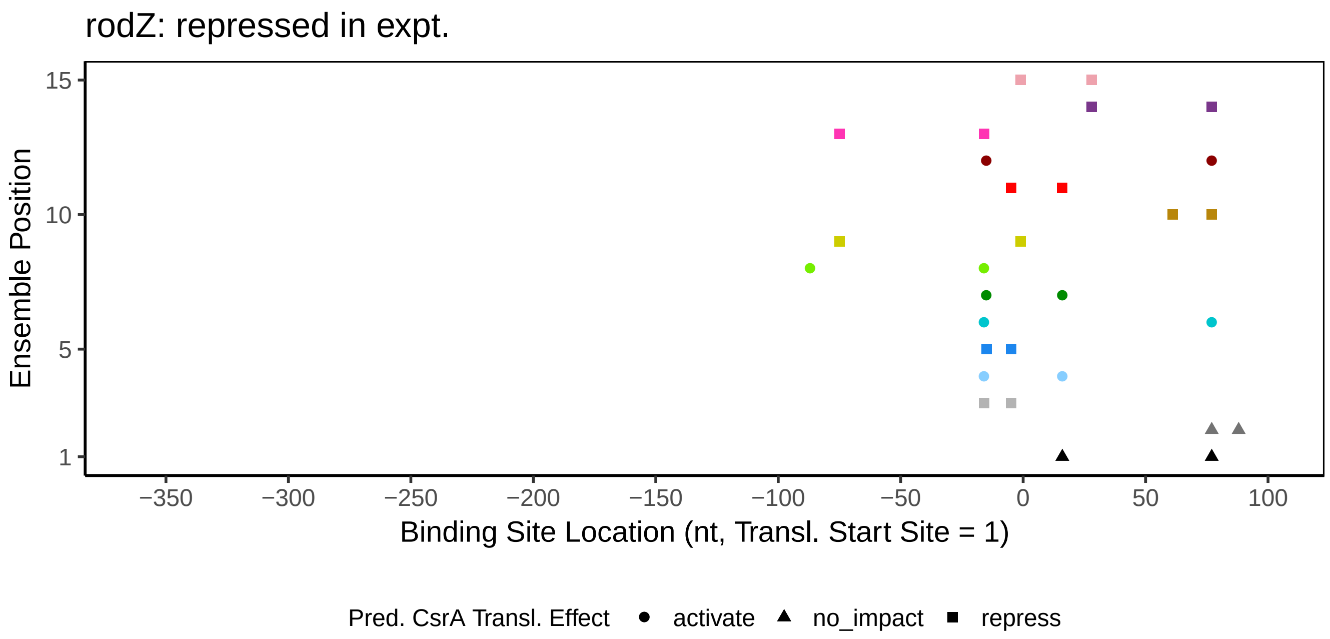
rodZ: repressed in expt.
Ensemble Position
15
10
5
1
−350
−300
−250
−200
−150
−100
−50
0
50
100
Binding Site Location (nt, Transl. Start Site = 1)
Pred. CsrA Transl. Effect
activate
no_impact
repress

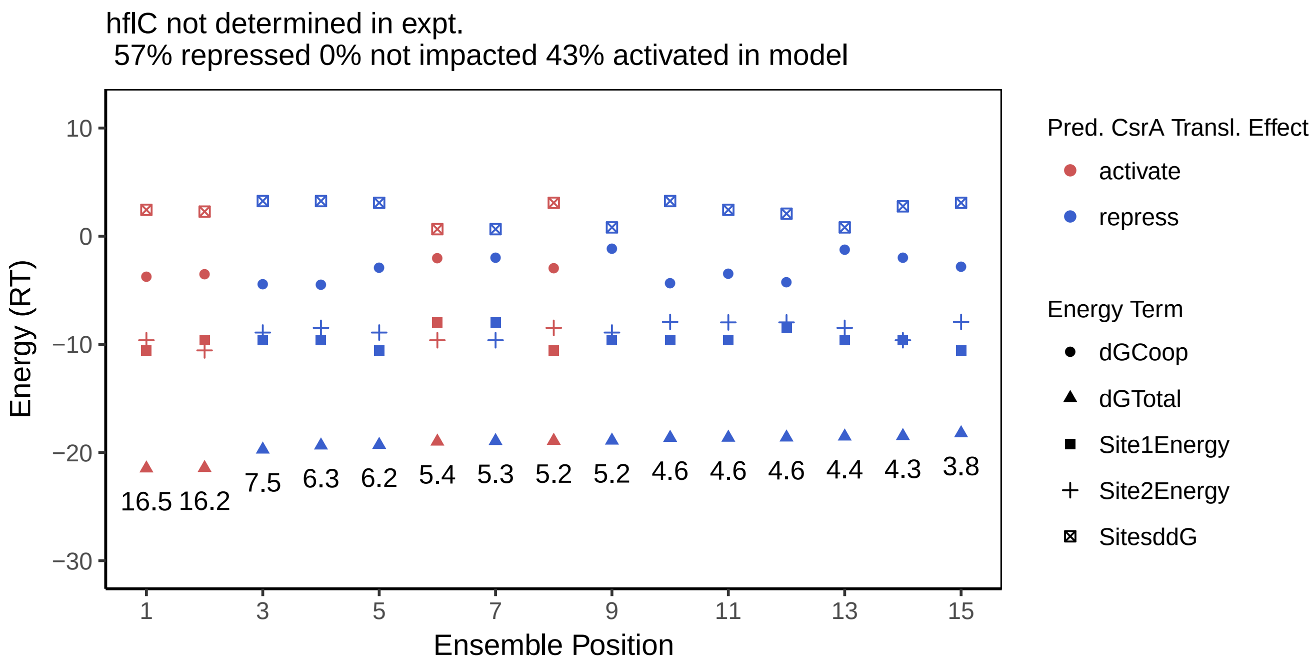

hflC not determined in expt.
57% repressed 0% not impacted 43% activated in model
Energy (RT)
10
0
−10
−20
−30
16.5 16.2 7.5 6.3 6.2 5.4 5.3 5.2 5.2 4.6 4.6 4.6 4.4 4.3 3.8
1
3
5
7
9
11
13
15
Ensemble Position
Pred. CsrA Transl. Effect
activate
repress
Energy Term
dGCoop
dGTotal
Site1Energy
Site2Energy
SitesddG

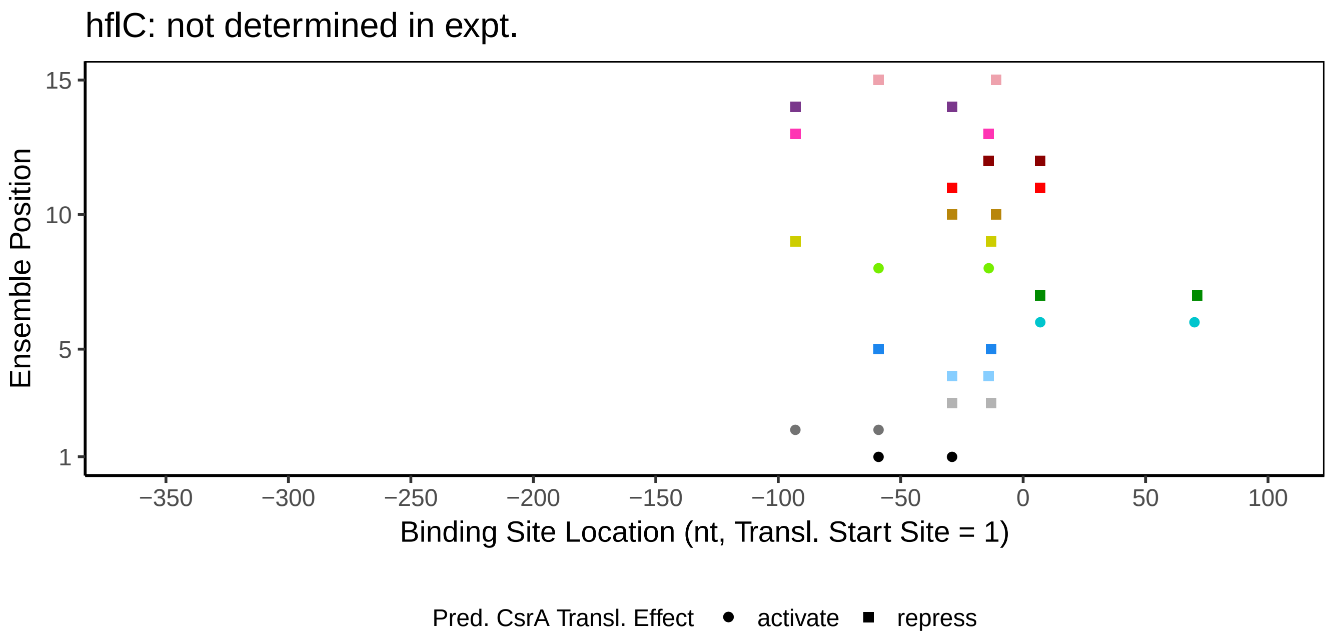

hflC: not determined in expt.
Ensemble Position
15
10
5
1
−350
−300
−250
−200
−150
−100
−50
0
50
100
Binding Site Location (nt, Transl. Start Site = 1)
Pred. CsrA Transl. Effect
activate
repress

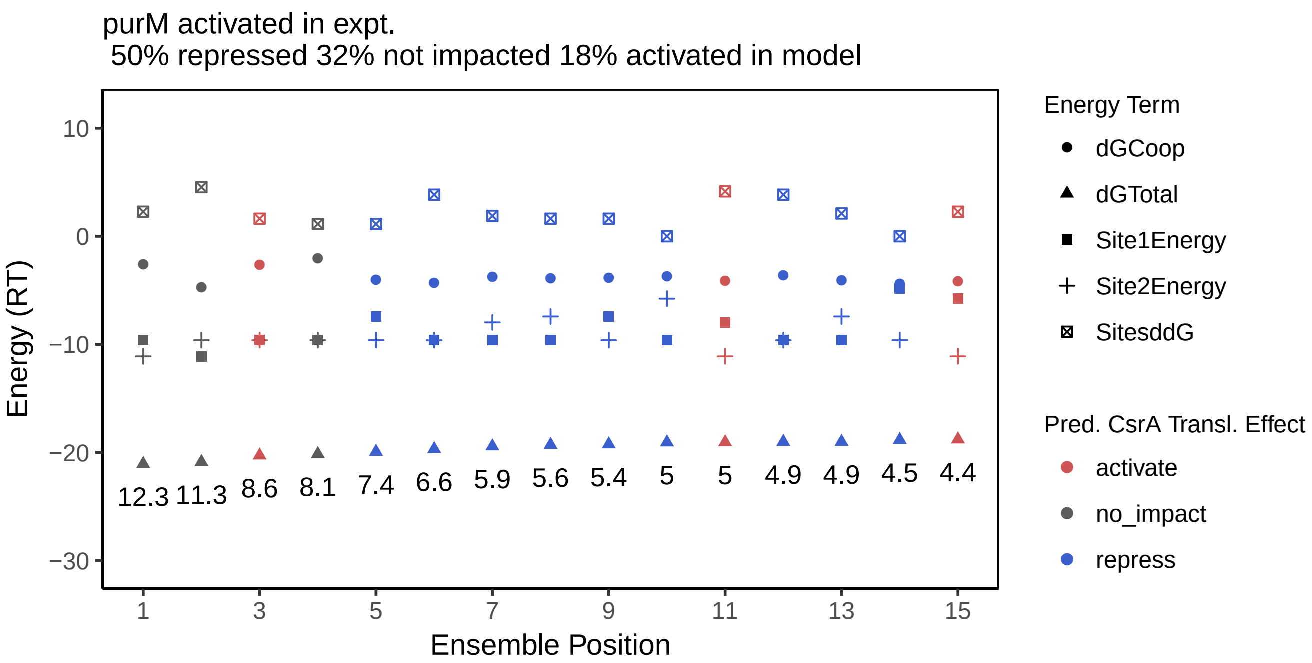
purM activated in expt.
50% repressed 32% not impacted 18% activated in model
Energy (RT)
10
0
−10
−20
−30
12.3 11.3 8.6 8.1 7.4 6.6 5.9 5.6 5.4 5 5 4.9 4.9 4.5 4.4
1 3 5 7 9 11 13 15
Ensemble Position
Energy Term
dGCoop
dGTotal
Site1Energy
Site2Energy
SitesddG
Pred. CsrA Transl. Effect
activate
no_impact
repress

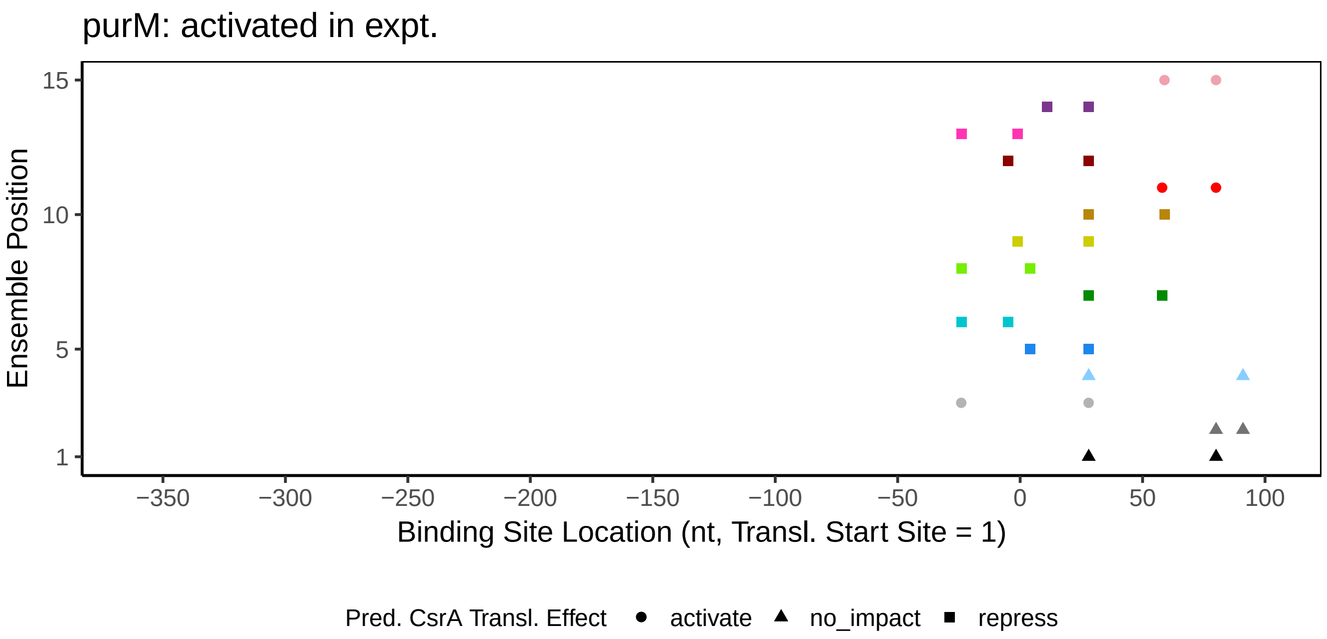
purM: activated in expt.
Ensemble Position
15
10
5
1
−350 −300 −250 −200 −150 −100 −50 0 50 100
Binding Site Location (nt, Transl. Start Site = 1)
Pred. CsrA Transl. Effect
activate
no_impact
repress

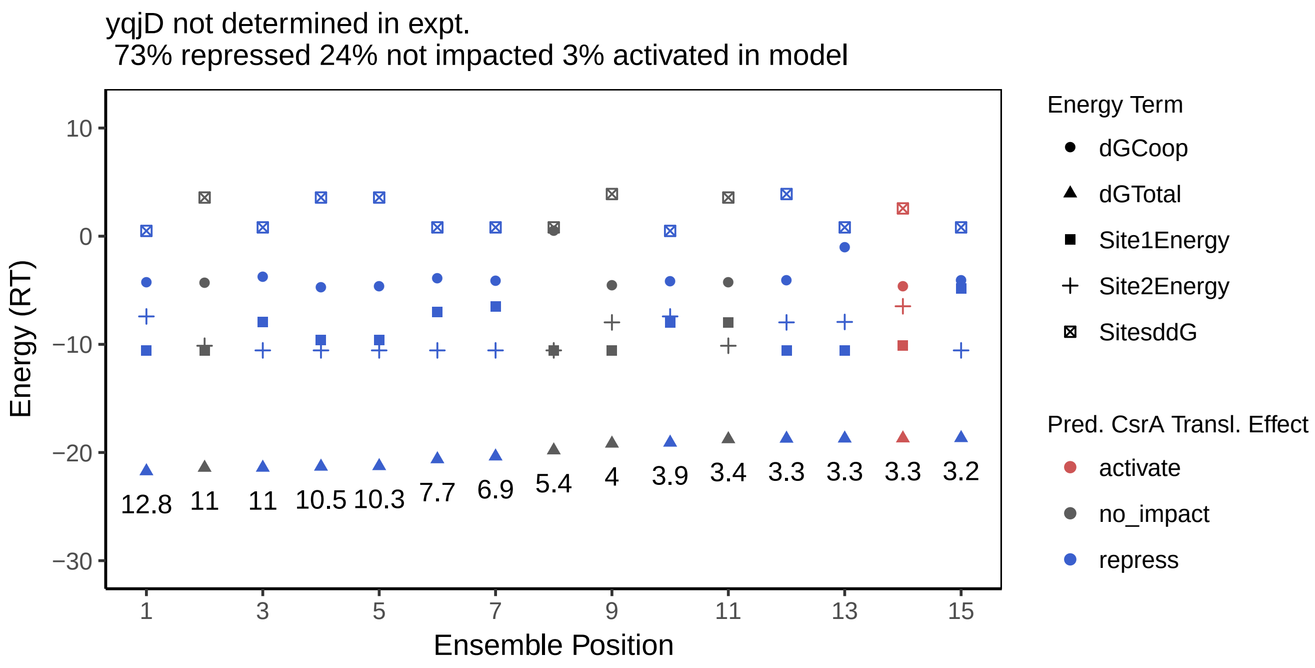
yqjD not determined in expt.
73% repressed 24% not impacted 3% activated in model
Energy (RT)
Ensemble Position
12.8
11
11
10.5
10.3
7.7
6.9
5.4
4
3.9
3.4
3.3
3.3
3.3
3.2
Energy Term
dGCoop
dGTotal
Site1Energy
Site2Energy
SitesddG
Pred. CsrA Transl. Effect
activate
no_impact
repress

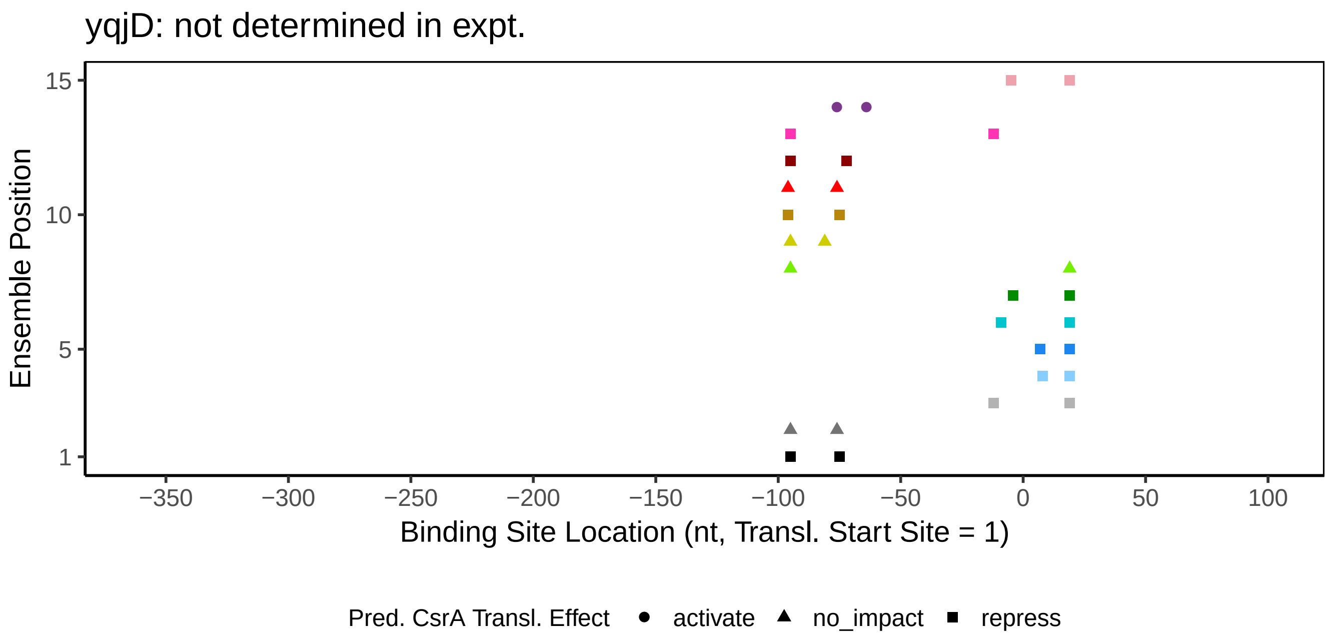
yqjD: not determined in expt.
Ensemble Position
Binding Site Location (nt, Transl. Start Site = 1)
Pred. CsrA Transl. Effect
activate
no_impact
repress

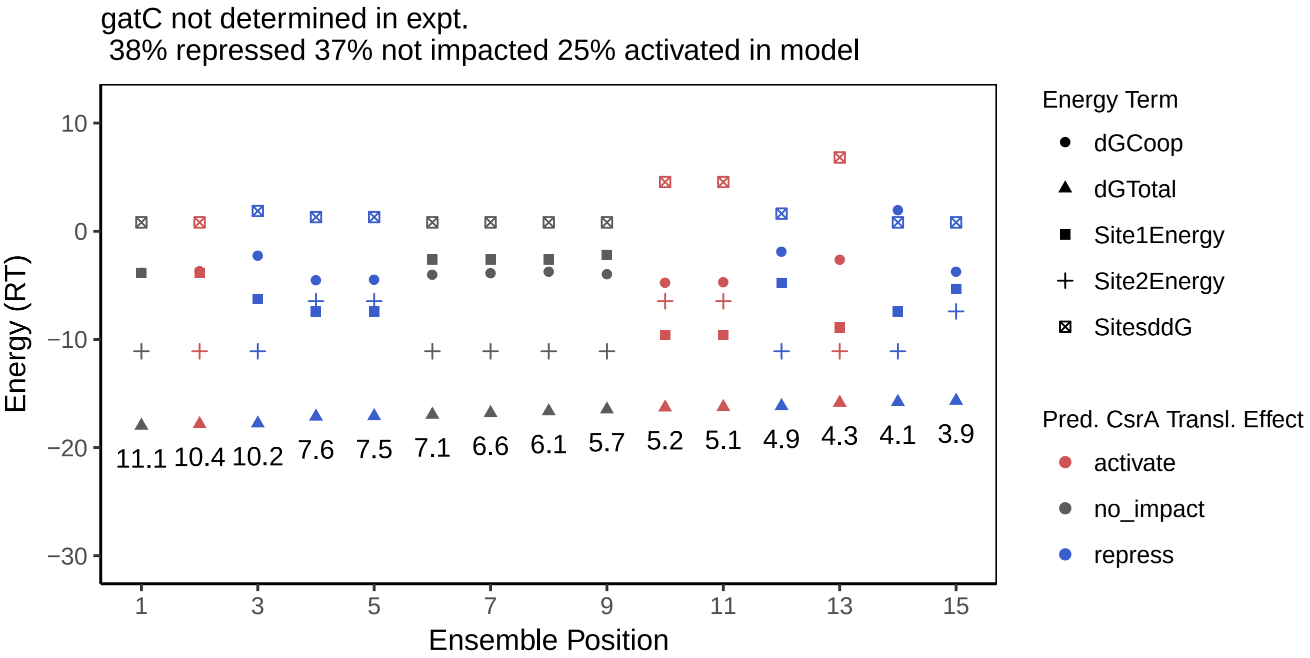

gatC not determined in expt.
38% repressed 37% not impacted 25% activated in model
Energy (RT)
10
0
−10
−20
−30
11.1 10.4 10.2 7.6 7.5 7.1 6.6 6.1 5.7 5.2 5.1 4.9 4.3 4.1 3.9
1 3 5 7 9 11 13 15
Ensemble Position
Energy Term
dGCoop
dGTotal
Site1Energy
Site2Energy
SitesddG
Pred. CsrA Transl. Effect
activate
no_impact
repress

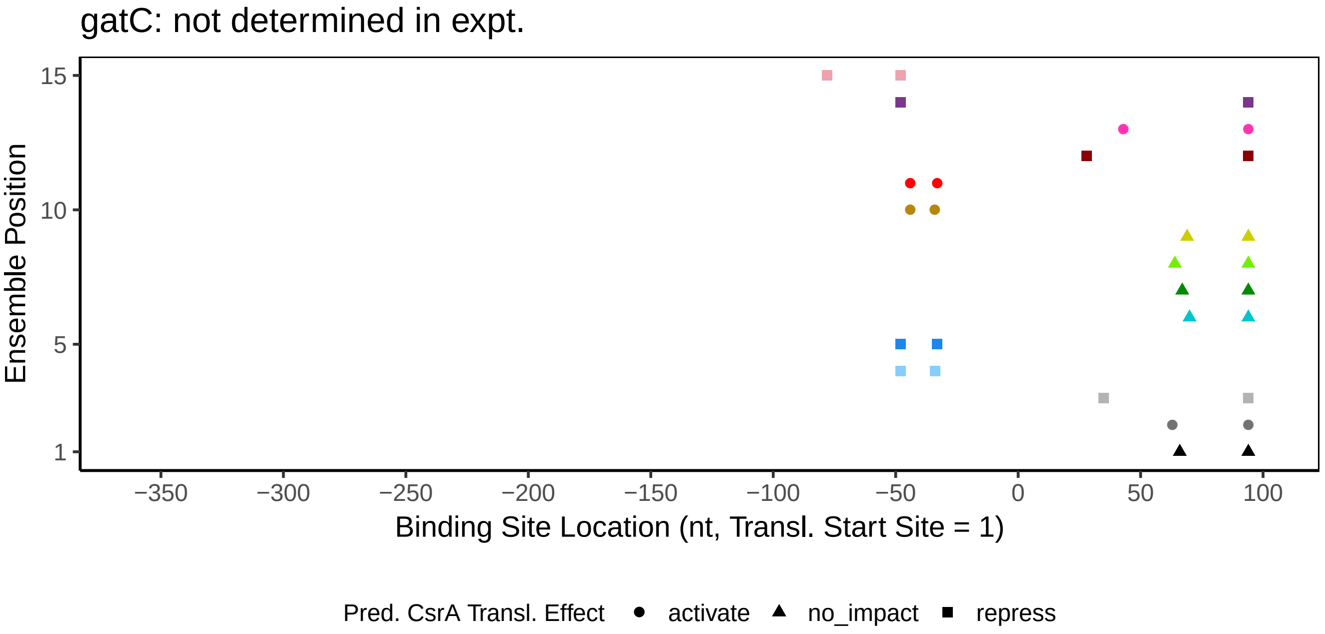

gatC: not determined in expt.
Ensemble Position
15
10
5
1
−350 −300 −250 −200 −150 −100 −50 0 50 100
Binding Site Location (nt, Transl. Start Site = 1)
Pred. CsrA Transl. Effect
activate
no_impact
repress

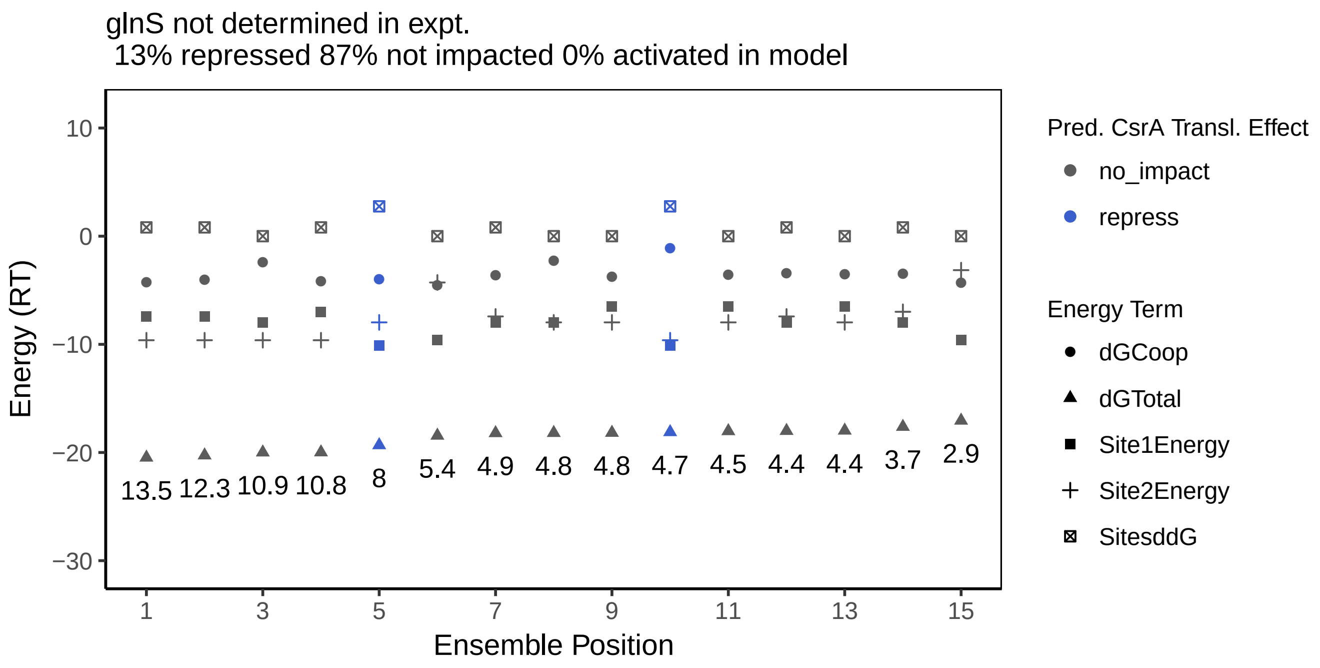

glnS not determined in expt.
13% repressed 87% not impacted 0% activated in model
Energy (RT)
Ensemble Position
13.5 12.3 10.9 10.8 8 5.4 4.9 4.8 4.8 4.7 4.5 4.4 4.4 3.7 2.9
Pred. CsrA Transl. Effect
no_impact
repress
Energy Term
dGCoop
dGTotal
Site1Energy
Site2Energy
SitesddG

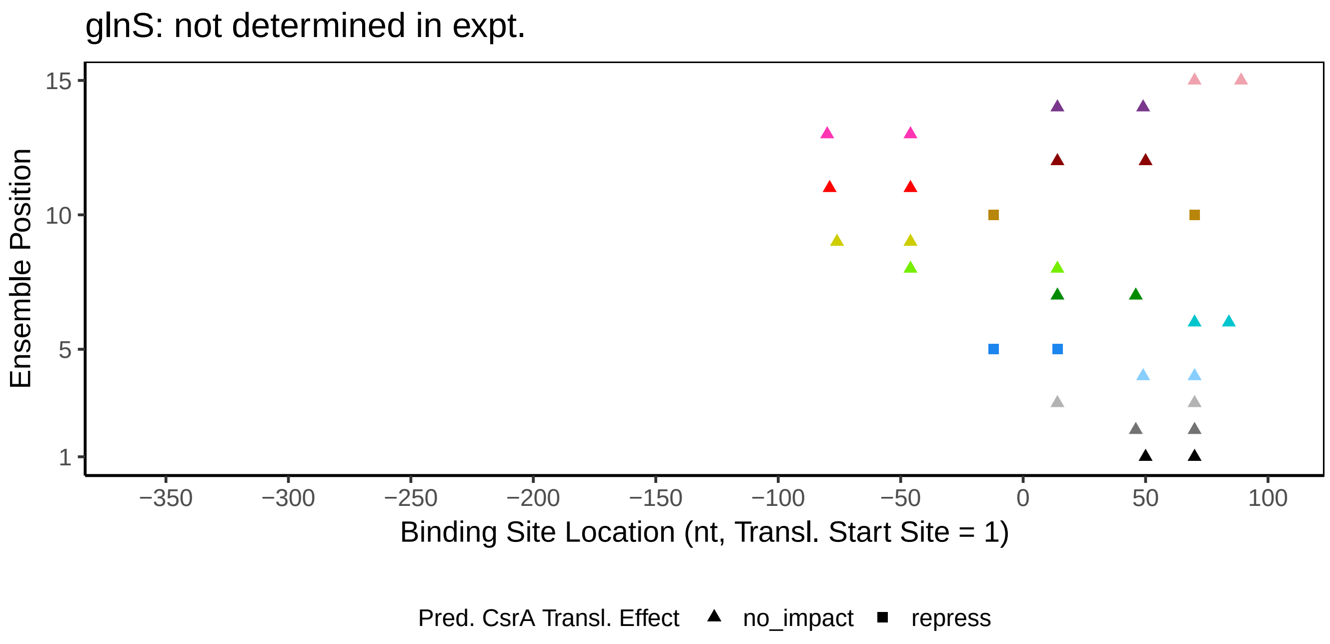

glnS: not determined in expt.
Ensemble Position
Binding Site Location (nt, Transl. Start Site = 1)
Pred. CsrA Transl. Effect
no_impact
repress

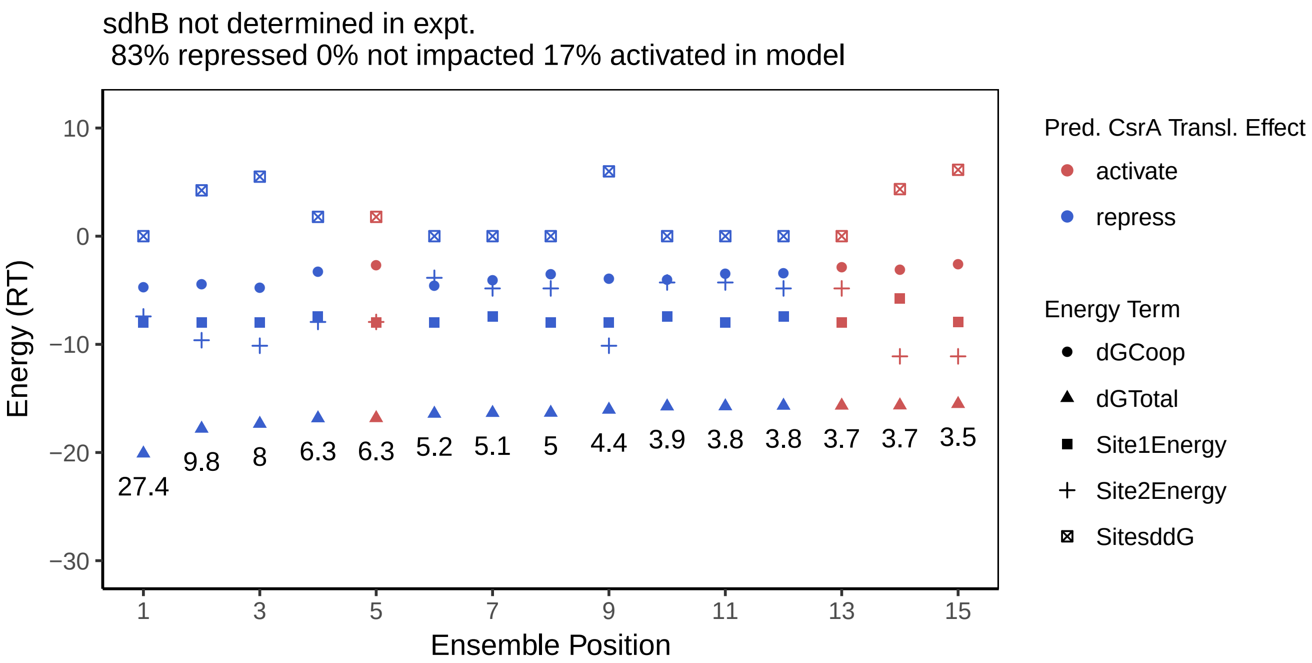
sdhB not determined in expt.
83% repressed 0% not impacted 17% activated in model
Energy (RT)
Ensemble Position
27.4
9.8
8
6.3
6.3
5.2
5.1
5
4.4
3.9
3.8
3.8
3.7
3.7
3.5
Pred. CsrA Transl. Effect
activate
repress
Energy Term
dGCoop
dGTotal
Site1Energy
Site2Energy
SitesddG

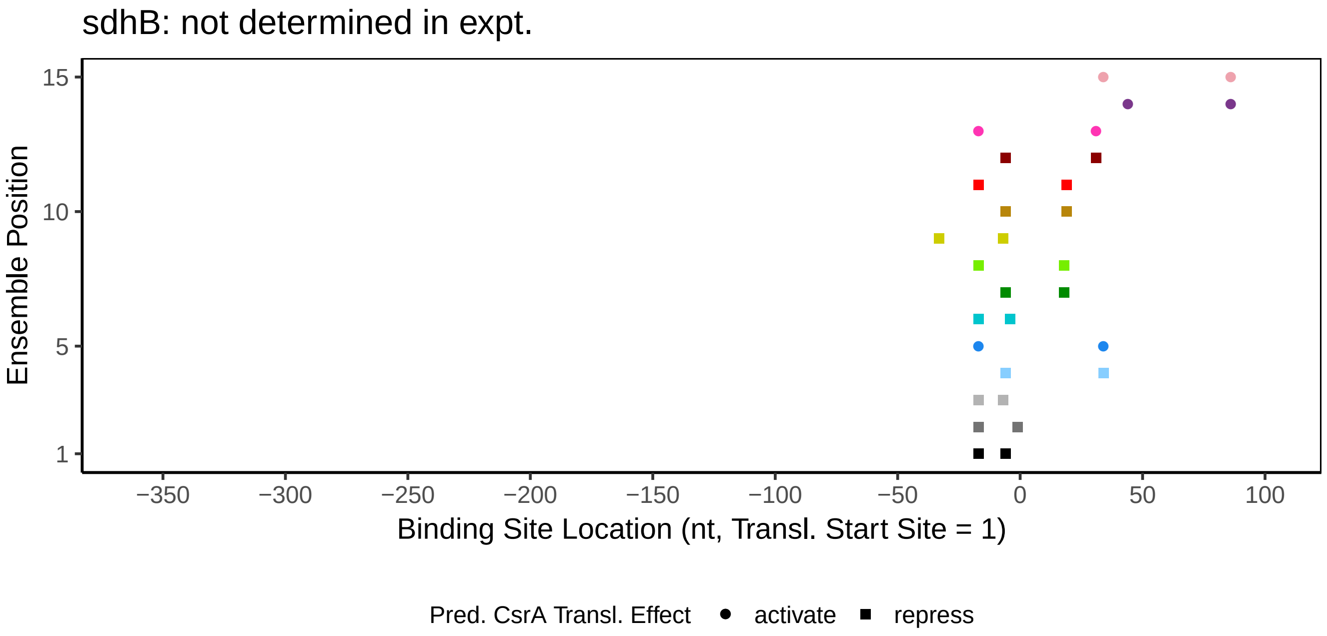
sdhB: not determined in expt.
Ensemble Position
Binding Site Location (nt, Transl. Start Site = 1)
Pred. CsrA Transl. Effect
activate
repress

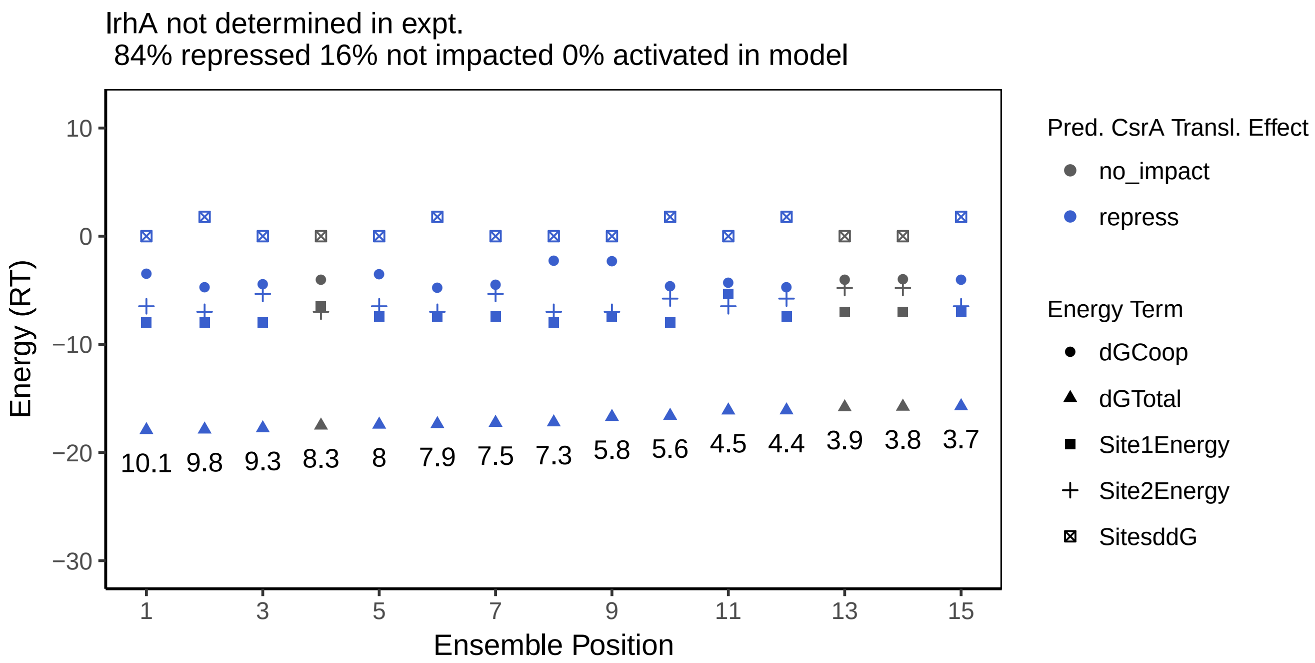

IrhA not determined in expt.
84% repressed 16% not impacted 0% activated in model
Energy (RT)
Ensemble Position
10.1 9.8 9.3 8.3 8 7.9 7.5 7.3 5.8 5.6 4.5 4.4 3.9 3.8 3.7
Pred. CsrA Transl. Effect
no_impact
repress
Energy Term
dGCoop
dGTotal
Site1Energy
Site2Energy
SitesddG

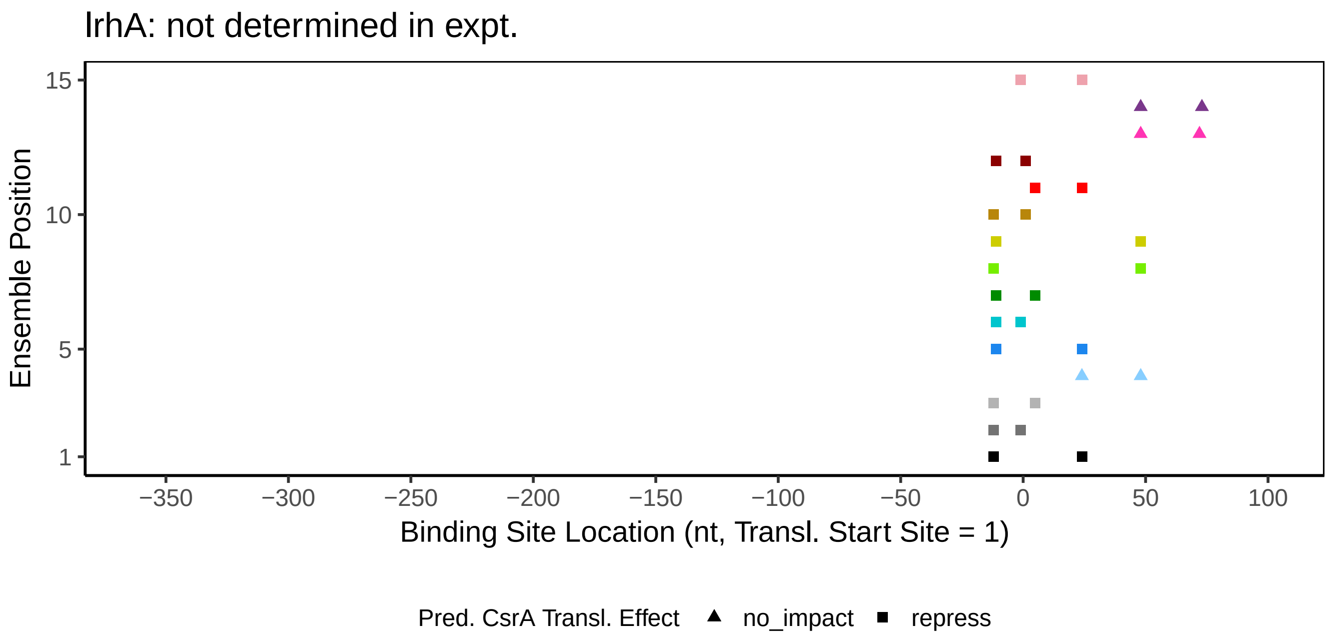

IrhA: not determined in expt.
Ensemble Position
Binding Site Location (nt, Transl. Start Site = 1)
Pred. CsrA Transl. Effect
no_impact
repress

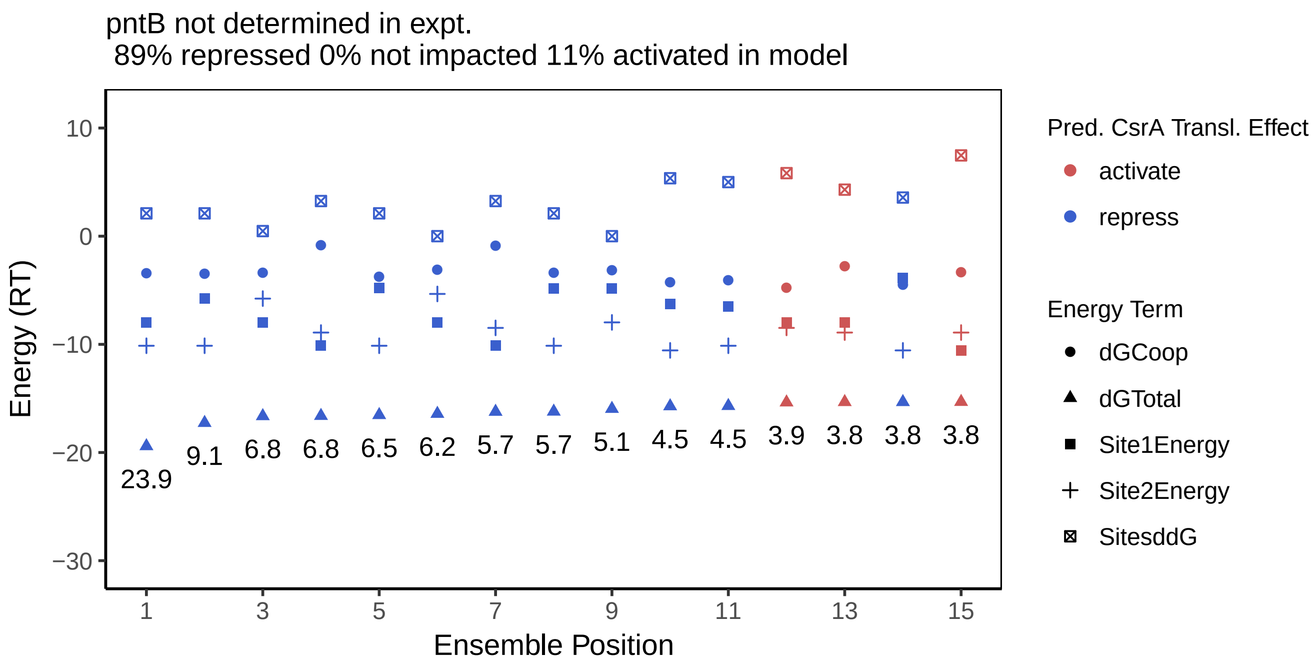
pntB not determined in expt.
89% repressed 0% not impacted 11% activated in model
Energy (RT)
10
0
−10
−20
−30
23.9
9.1
6.8
6.8
6.5
6.2
5.7
5.7
5.1
4.5
4.5
3.9
3.8
3.8
3.8
1
3
5
7
9
11
13
15
Ensemble Position
Pred. CsrA Transl. Effect
activate
repress
Energy Term
dGCoop
dGTotal
Site1Energy
Site2Energy
SitesddG

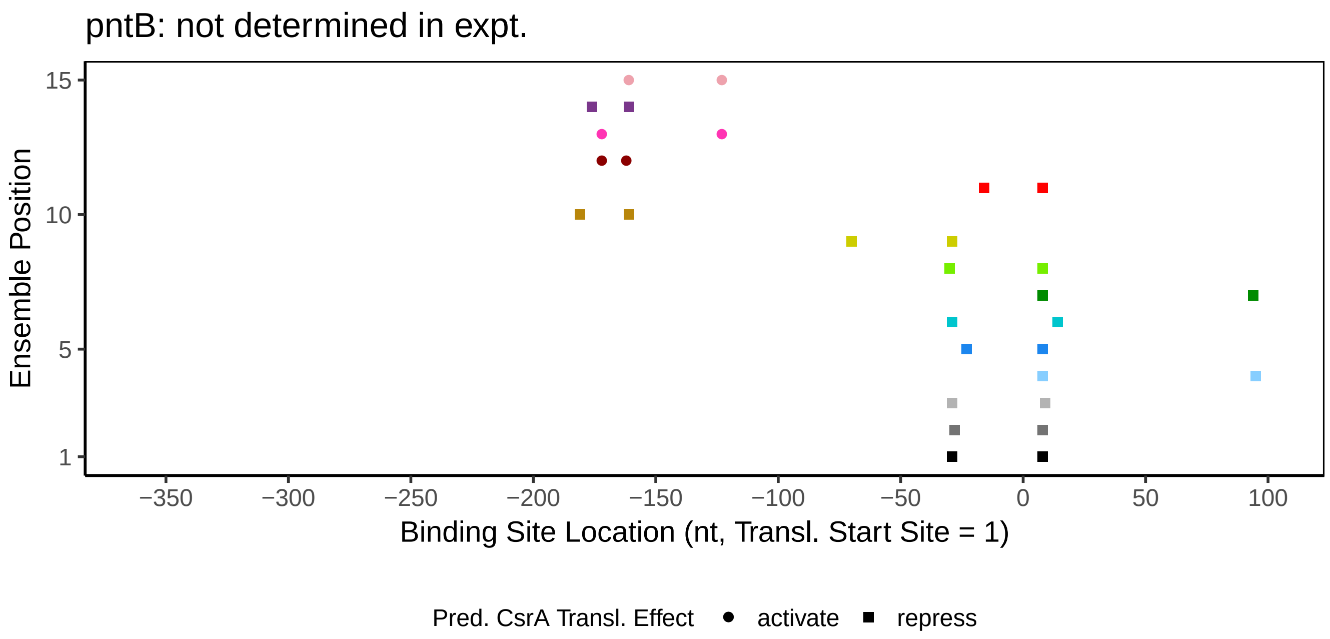
pntB: not determined in expt.
Ensemble Position
15
10
5
1
−350
−300
−250
−200
−150
−100
−50
0
50
100
Binding Site Location (nt, Transl. Start Site = 1)
Pred. CsrA Transl. Effect
activate
repress

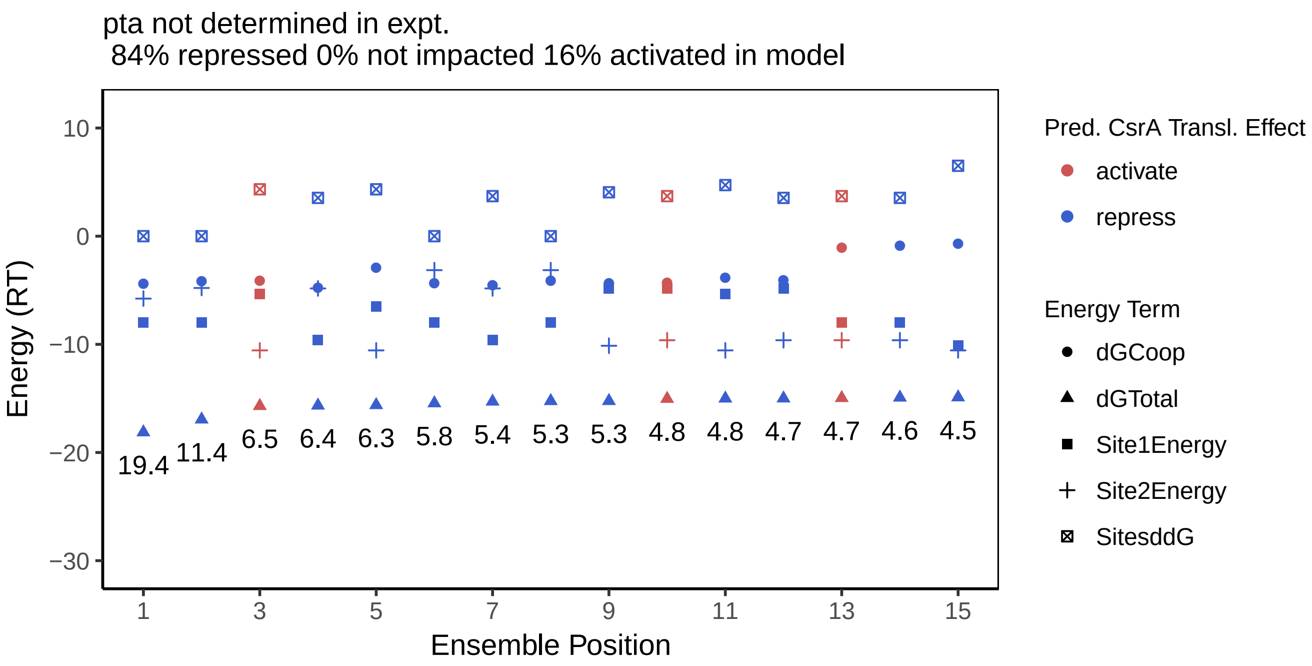
pta not determined in expt.
84% repressed 0% not impacted 16% activated in model
Energy (RT)
Ensemble Position
19.4
11.4
6.5
6.4
6.3
5.8
5.4
5.3
5.3
4.8
4.8
4.7
4.7
4.6
4.5
Pred. CsrA Transl. Effect
activate
repress
Energy Term
dGCoop
dGTotal
Site1Energy
Site2Energy
SitesddG

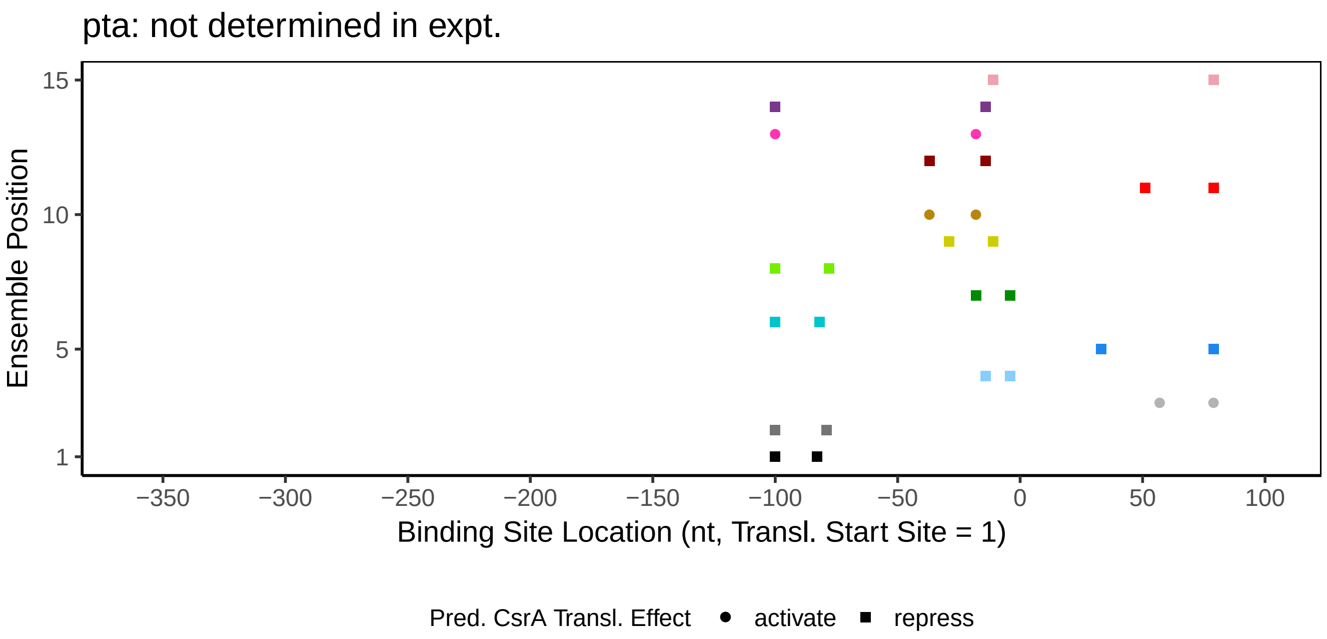
pta: not determined in expt.
Ensemble Position
Binding Site Location (nt, Transl. Start Site = 1)
Pred. CsrA Transl. Effect
activate
repress

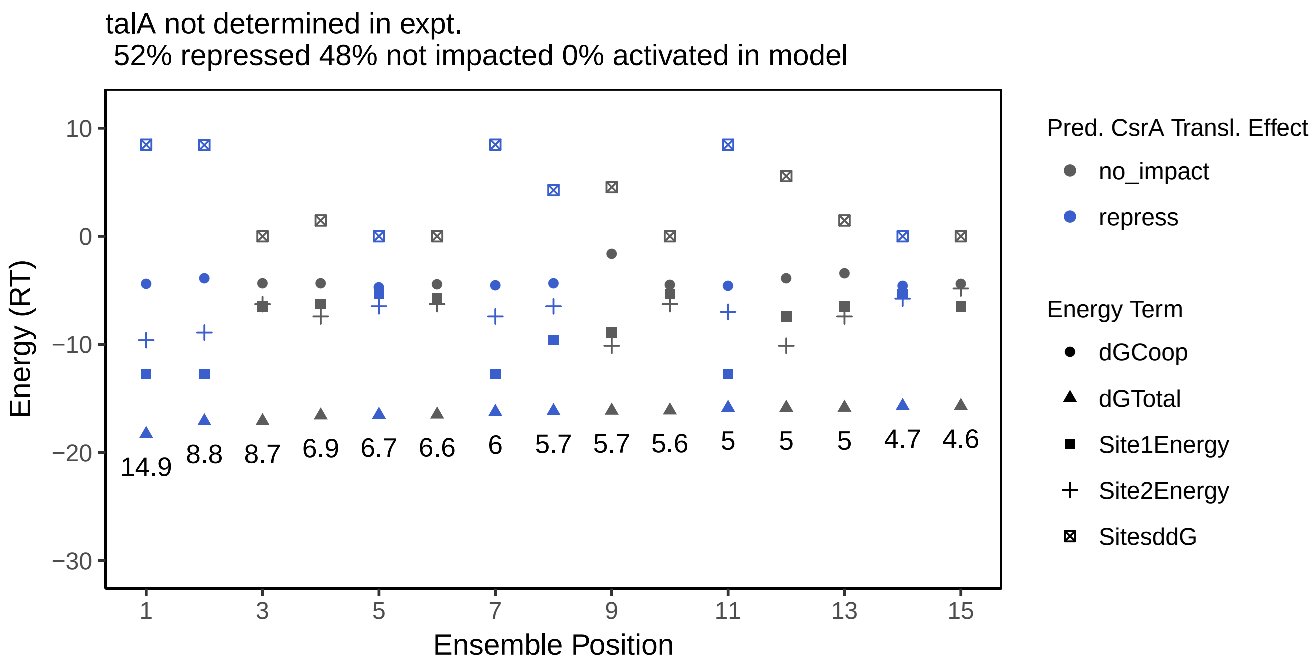
talA not determined in expt.
52% repressed 48% not impacted 0% activated in model
Energy (RT)
Ensemble Position
14.9
8.8
8.7
6.9
6.7
6.6
6
5.7
5.7
5.6
5
5
5
4.7
4.6
Pred. CsrA Transl. Effect
no_impact
repress
Energy Term
dGCoop
dGTotal
Site1Energy
Site2Energy
SitesddG

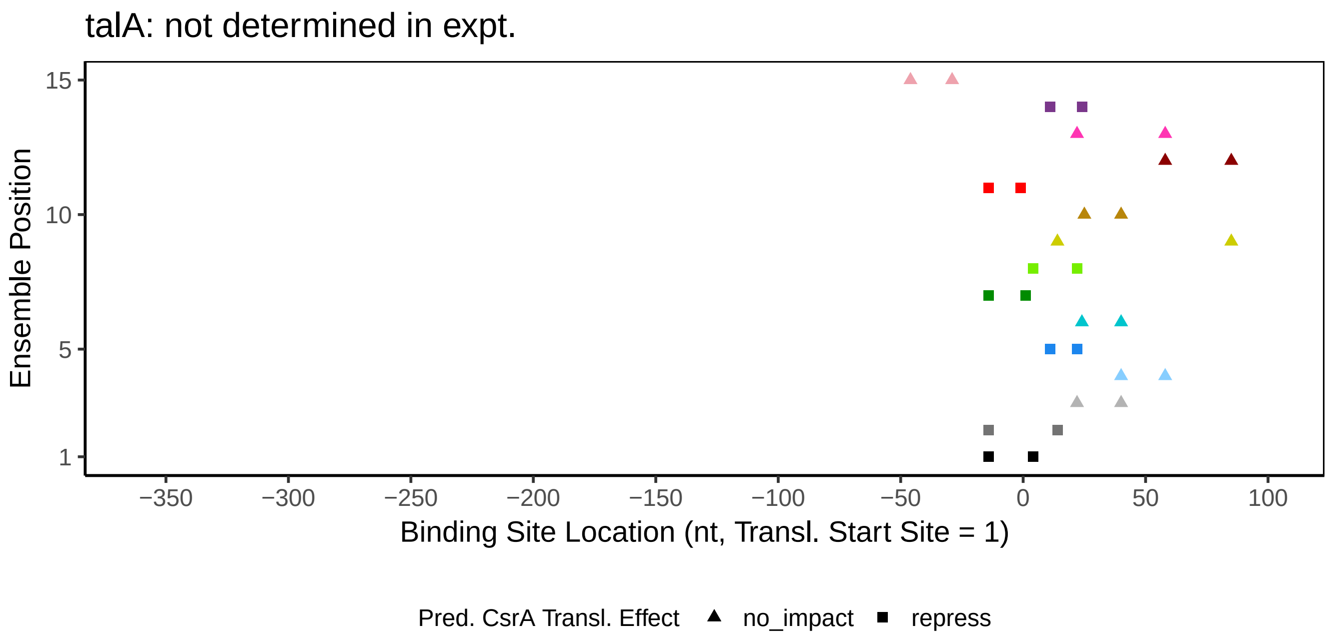
talA: not determined in expt.
Ensemble Position
Binding Site Location (nt, Transl. Start Site = 1)
Pred. CsrA Transl. Effect
no_impact
repress

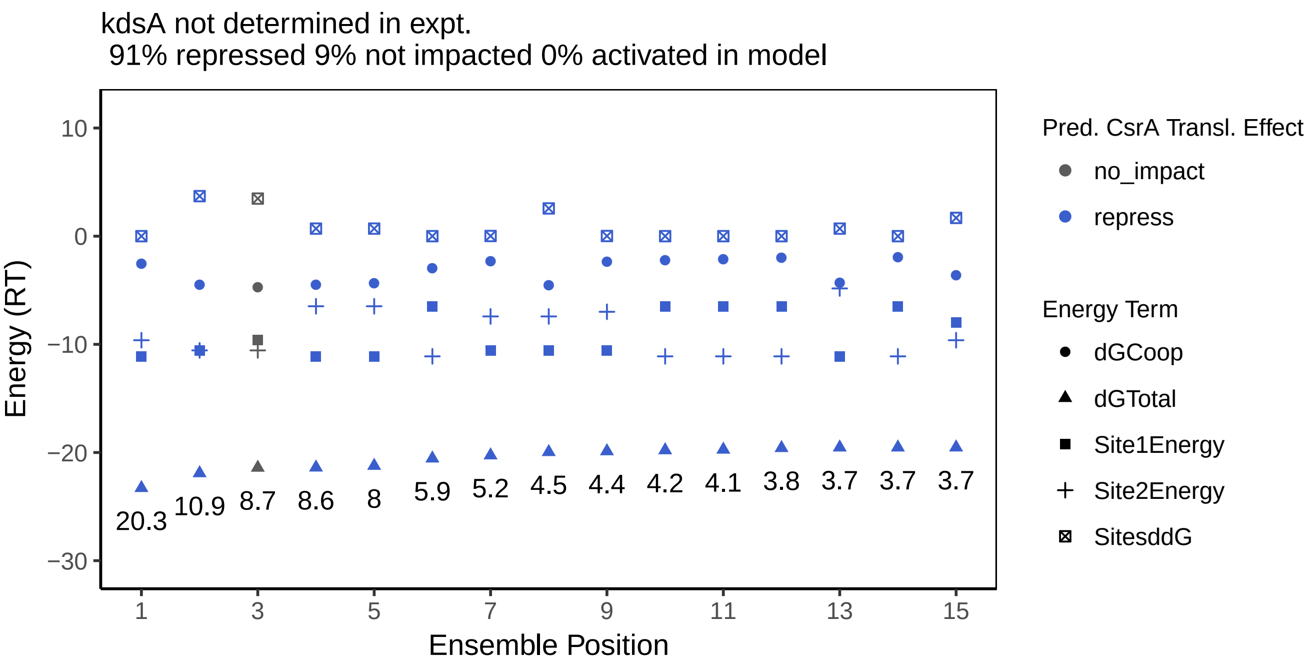

kdsA not determined in expt.
91% repressed 9% not impacted 0% activated in model
Energy (RT)
10
0
−10
−20
−30
20.3
10.9
8.7
8.6
8
5.9
5.2
4.5
4.4
4.2
4.1
3.8
3.7
3.7
3.7
1
3
5
7
9
11
13
15
Ensemble Position
Pred. CsrA Transl. Effect
no_impact
repress
Energy Term
dGCoop
dGTotal
Site1Energy
Site2Energy
SitesddG

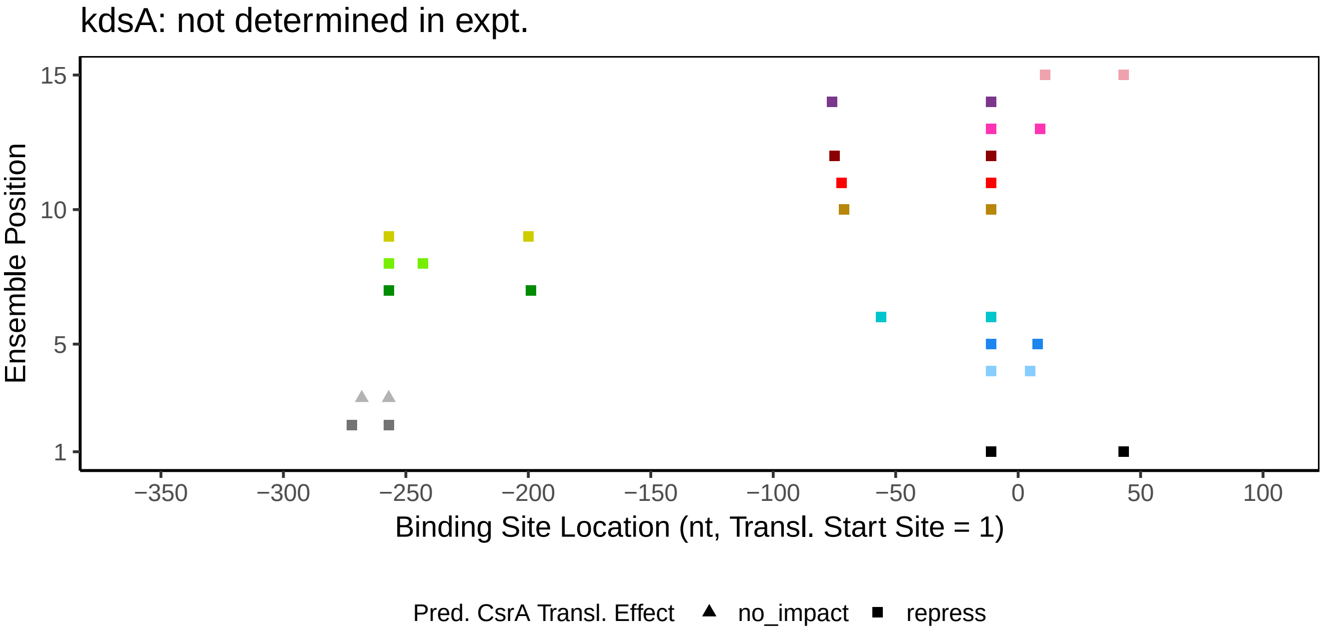

kdsA: not determined in expt.
Ensemble Position
15
10
5
1
−350
−300
−250
−200
−150
−100
−50
0
50
100
Binding Site Location (nt, Transl. Start Site = 1)
Pred. CsrA Transl. Effect
no_impact
repress

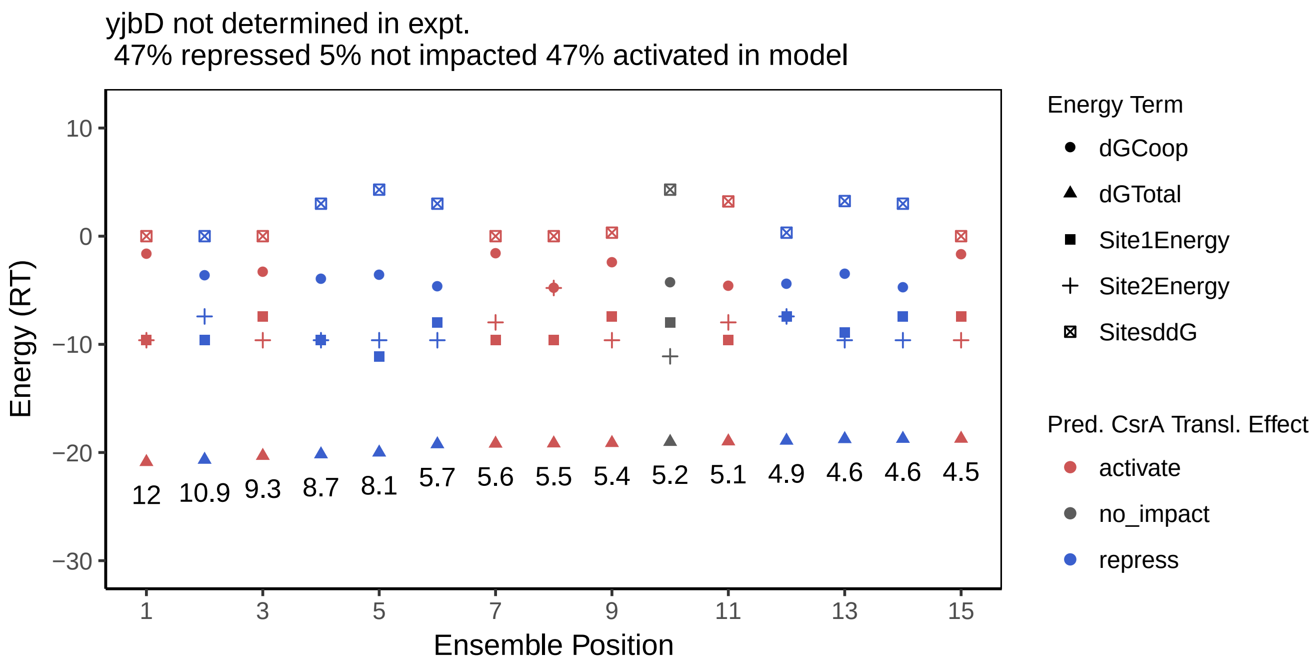

yjbD not determined in expt.
47% repressed 5% not impacted 47% activated in model
Energy (RT)
Ensemble Position
12 10.9 9.3 8.7 8.1 5.7 5.6 5.5 5.4 5.2 5.1 4.9 4.6 4.6 4.5
Energy Term
dGCoop
dGTotal
Site1Energy
Site2Energy
SitesddG
Pred. CsrA Transl. Effect
activate
no_impact
repress

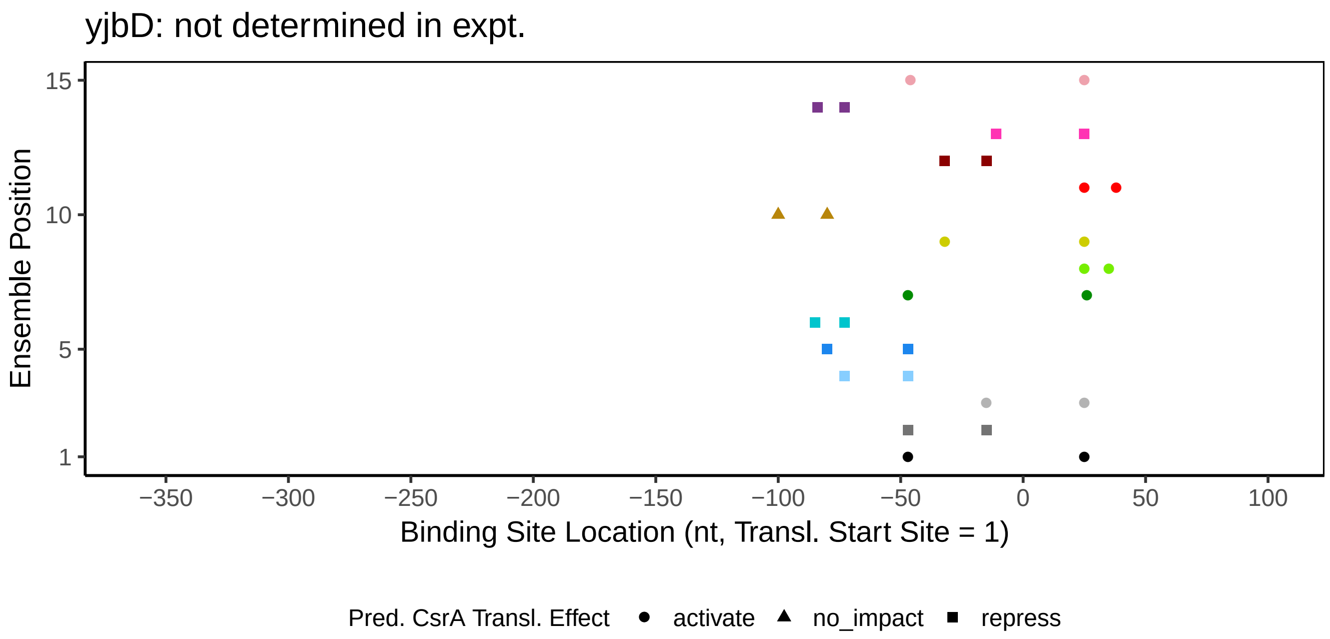

yjbD: not determined in expt.
Ensemble Position
Binding Site Location (nt, Transl. Start Site = 1)
Pred. CsrA Transl. Effect
activate
no_impact
repress

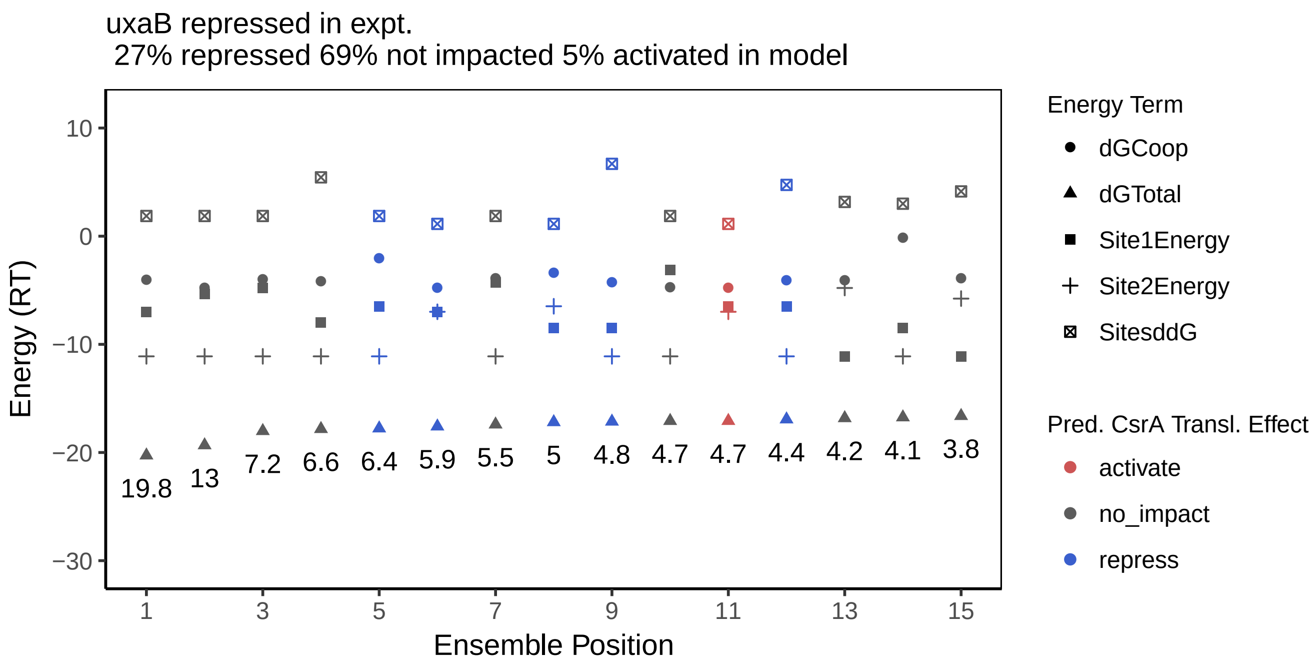
uxaB repressed in expt.
27% repressed 69% not impacted 5% activated in model
Energy (RT)
Ensemble Position
19.8
13
7.2
6.6
6.4
5.9
5.5
5
4.8
4.7
4.7
4.4
4.2
4.1
3.8
Energy Term
dGCoop
dGTotal
Site1Energy
Site2Energy
SitesddG
Pred. CsrA Transl. Effect
activate
no_impact
repress

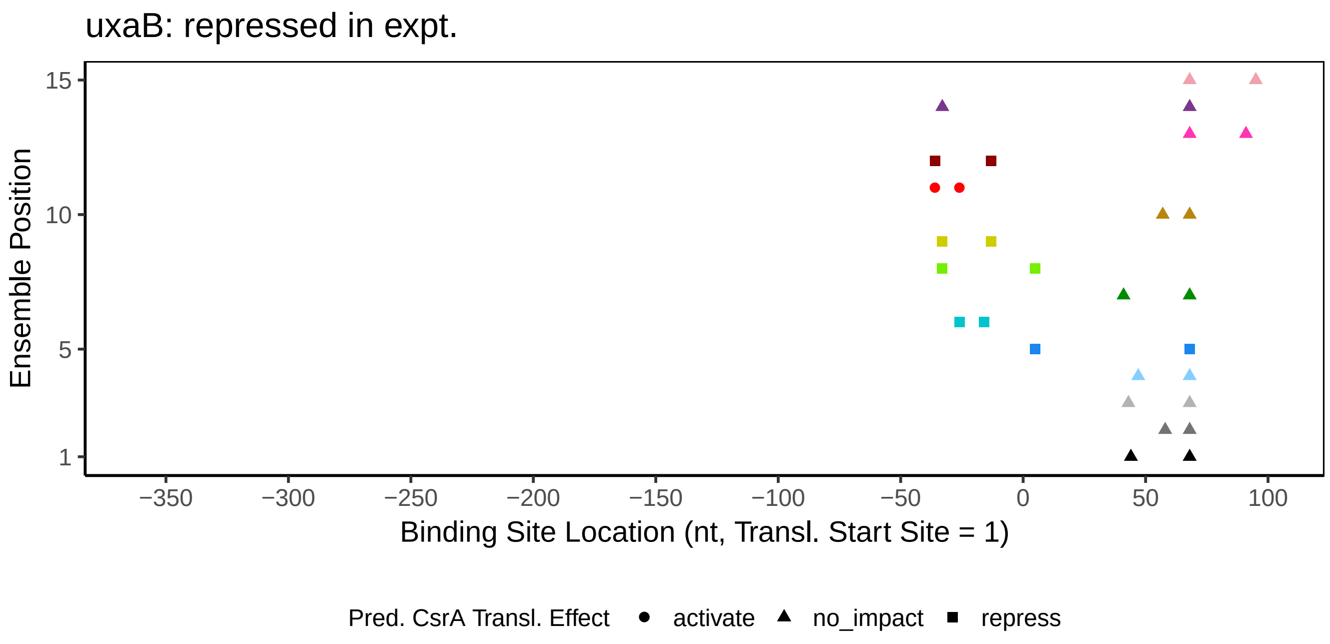
uxaB: repressed in expt.
Ensemble Position
Binding Site Location (nt, Transl. Start Site = 1)
Pred. CsrA Transl. Effect
activate
no_impact
repress

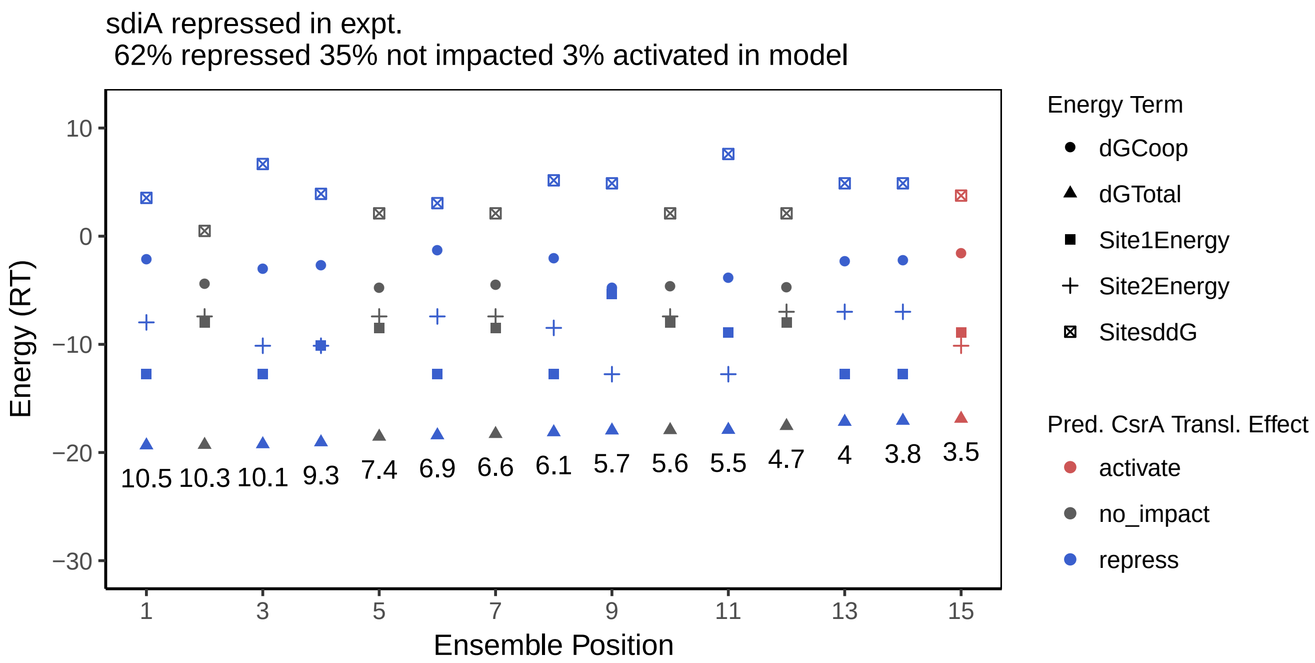
sdiA repressed in expt.
62% repressed 35% not impacted 3% activated in model
Energy (RT)
10
0
−10
−20
−30
10.5 10.3 10.1 9.3 7.4 6.9 6.6 6.1 5.7 5.6 5.5 4.7 4 3.8 3.5
1 3 5 7 9 11 13 15
Ensemble Position
Energy Term
dGCoop
dGTotal
Site1Energy
Site2Energy
SitesddG
Pred. CsrA Transl. Effect
activate
no_impact
repress

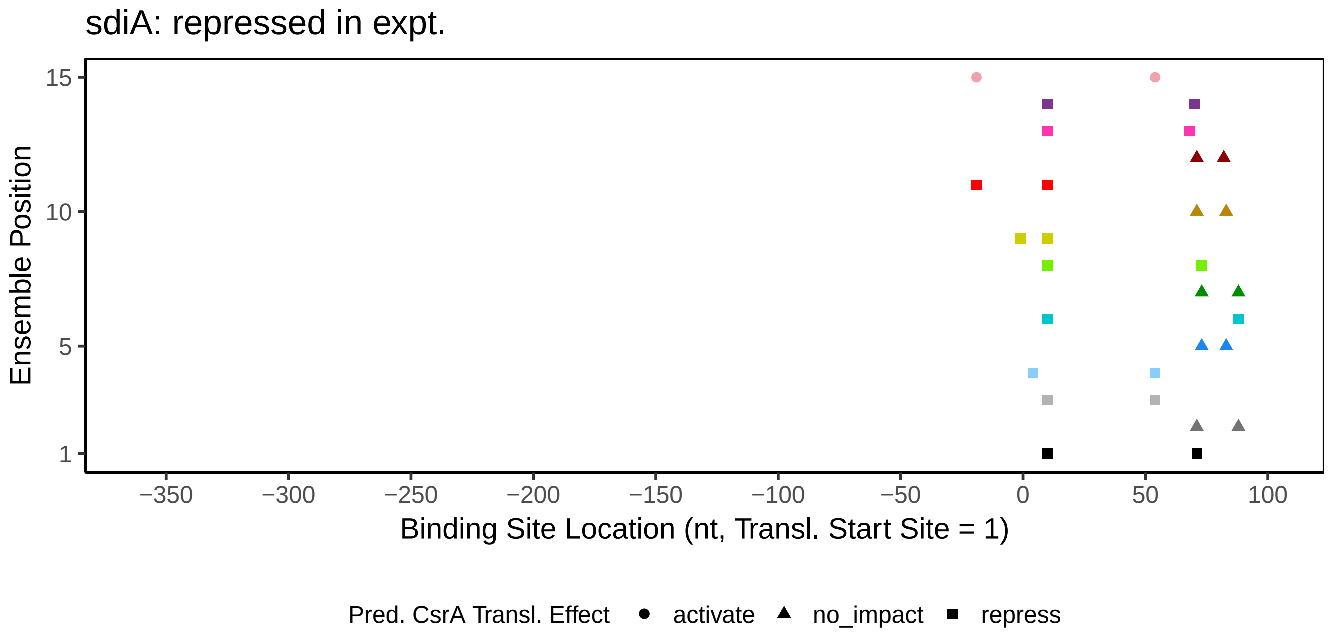
sdiA: repressed in expt.
Ensemble Position
15
10
5
1
−350 −300 −250 −200 −150 −100 −50 0 50 100
Binding Site Location (nt, Transl. Start Site = 1)
Pred. CsrA Transl. Effect
activate
no_impact
repress

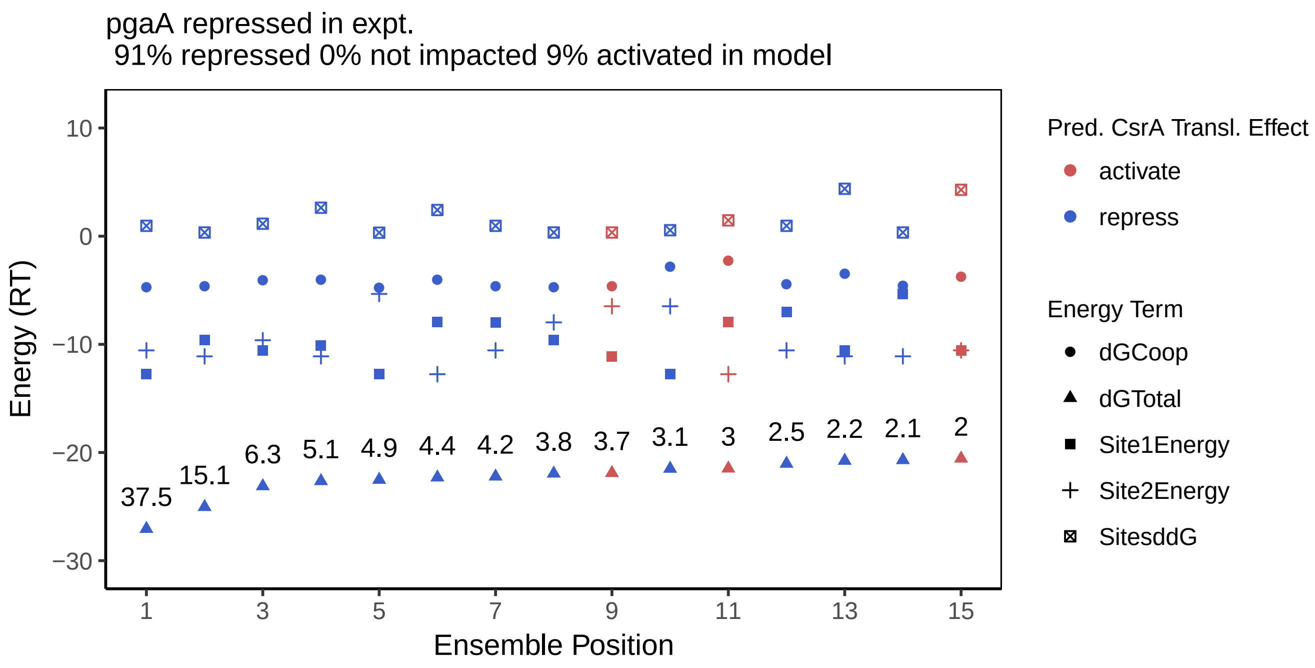
pgaA repressed in expt.
91% repressed 0% not impacted 9% activated in model
Energy (RT)
Ensemble Position
37.5
15.1
6.3
5.1
4.9
4.4
4.2
3.8
3.7
3.1
3
2.5
2.2
2.1
2
Pred. CsrA Transl. Effect
activate
repress
Energy Term
dGCoop
dGTotal
Site1Energy
Site2Energy
SitesddG

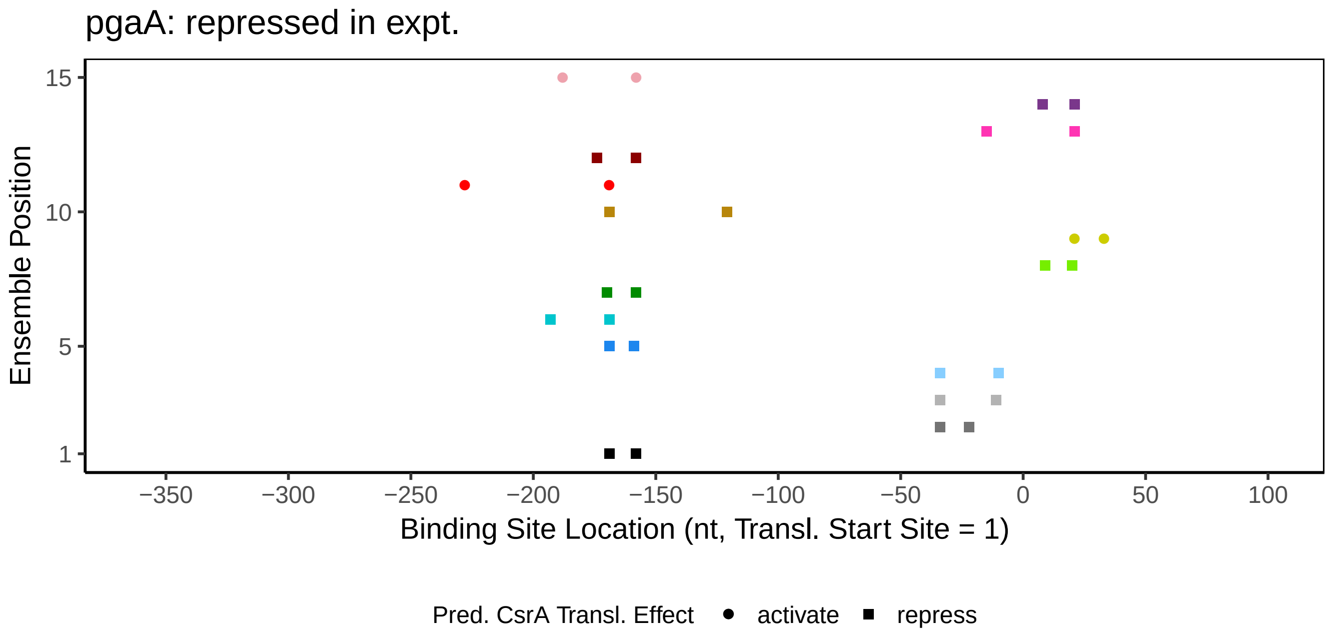
pgaA: repressed in expt.
Ensemble Position
Binding Site Location (nt, Transl. Start Site = 1)
Pred. CsrA Transl. Effect
activate
repress

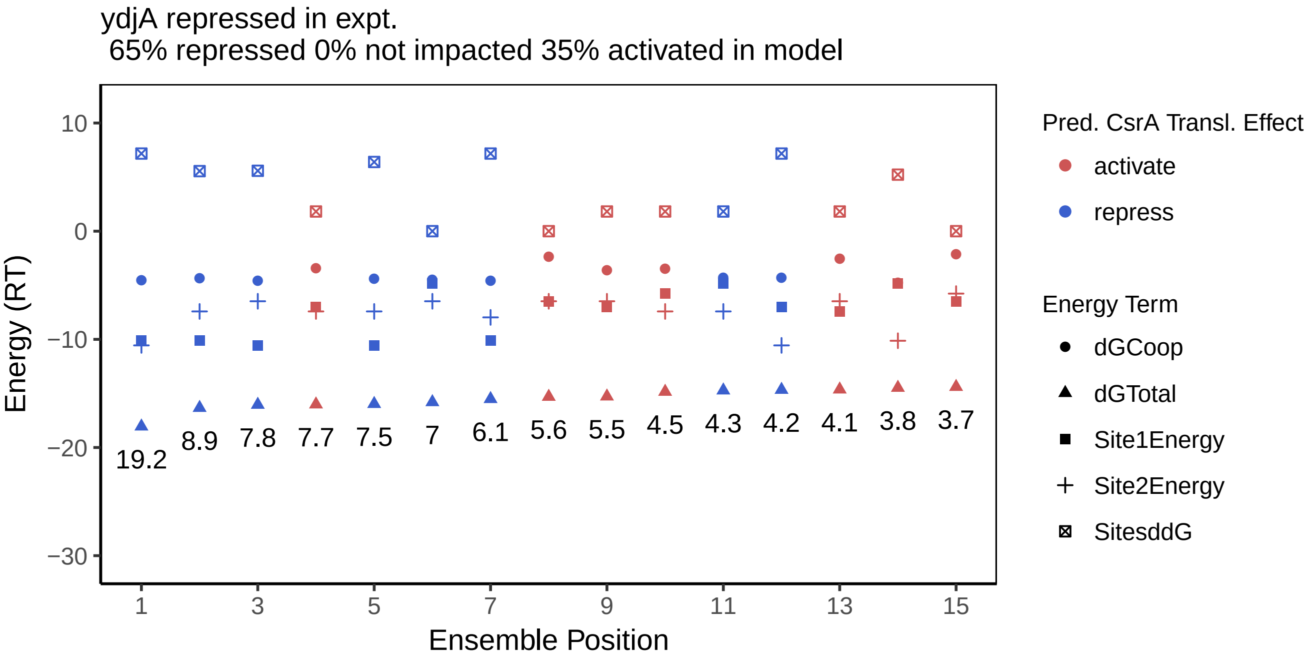
ydjA repressed in expt.
65% repressed 0% not impacted 35% activated in model
Energy (RT)
10
0
−10
−20
−30
19.2
8.9
7.8
7.7
7.5
7
6.1
5.6
5.5
4.5
4.3
4.2
4.1
3.8
3.7
1
3
5
7
9
11
13
15
Ensemble Position
Pred. CsrA Transl. Effect
activate
repress
Energy Term
dGCoop
dGTotal
Site1Energy
Site2Energy
SitesddG

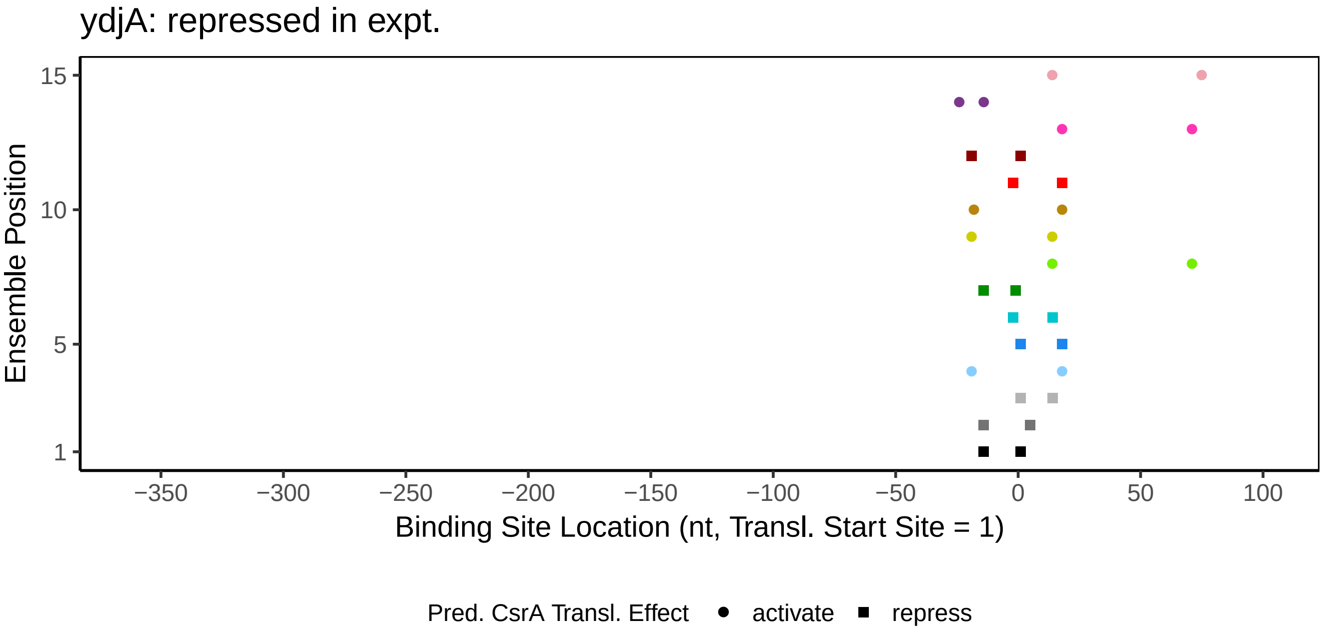
ydjA: repressed in expt.
Ensemble Position
15
10
5
1
−350
−300
−250
−200
−150
−100
−50
0
50
100
Binding Site Location (nt, Transl. Start Site = 1)
Pred. CsrA Transl. Effect
activate
repress

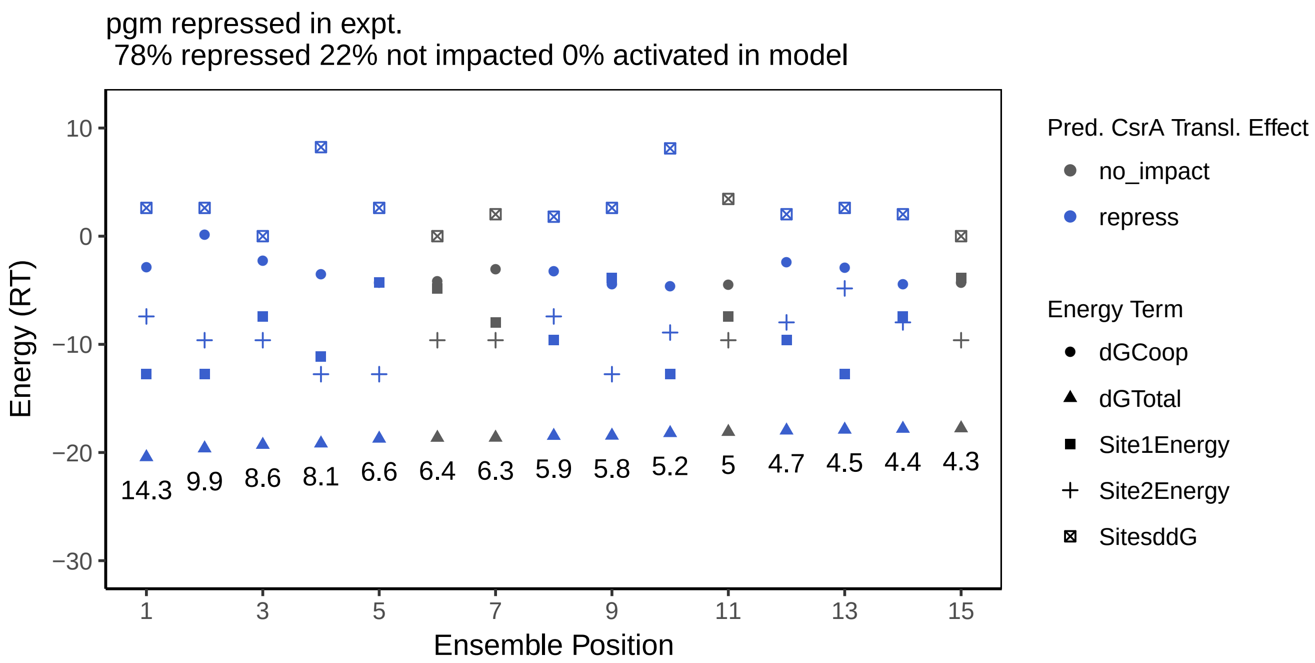
pgm repressed in expt.
78% repressed 22% not impacted 0% activated in model
Energy (RT)
Ensemble Position
14.3 9.9 8.6 8.1 6.6 6.4 6.3 5.9 5.8 5.2 5 4.7 4.5 4.4 4.3
Pred. CsrA Transl. Effect
no_impact
repress
Energy Term
dGCoop
dGTotal
Site1Energy
Site2Energy
SitesddG

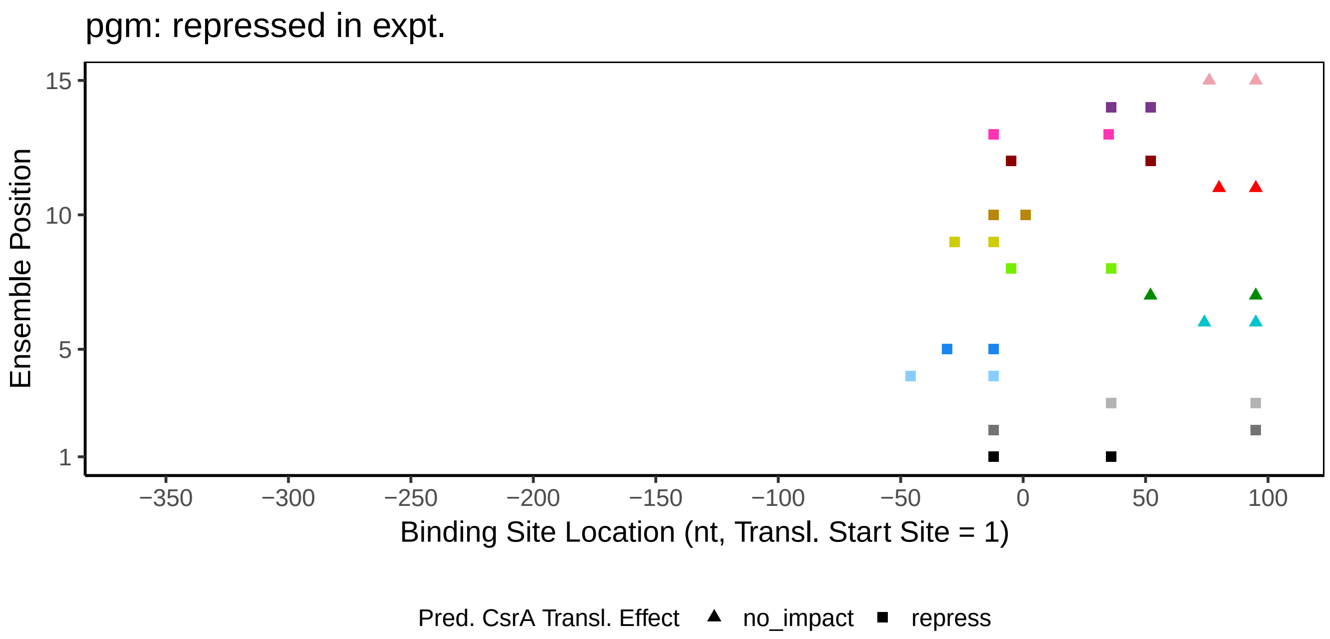
pgm: repressed in expt.
Ensemble Position
Binding Site Location (nt, Transl. Start Site = 1)
Pred. CsrA Transl. Effect
no_impact
repress

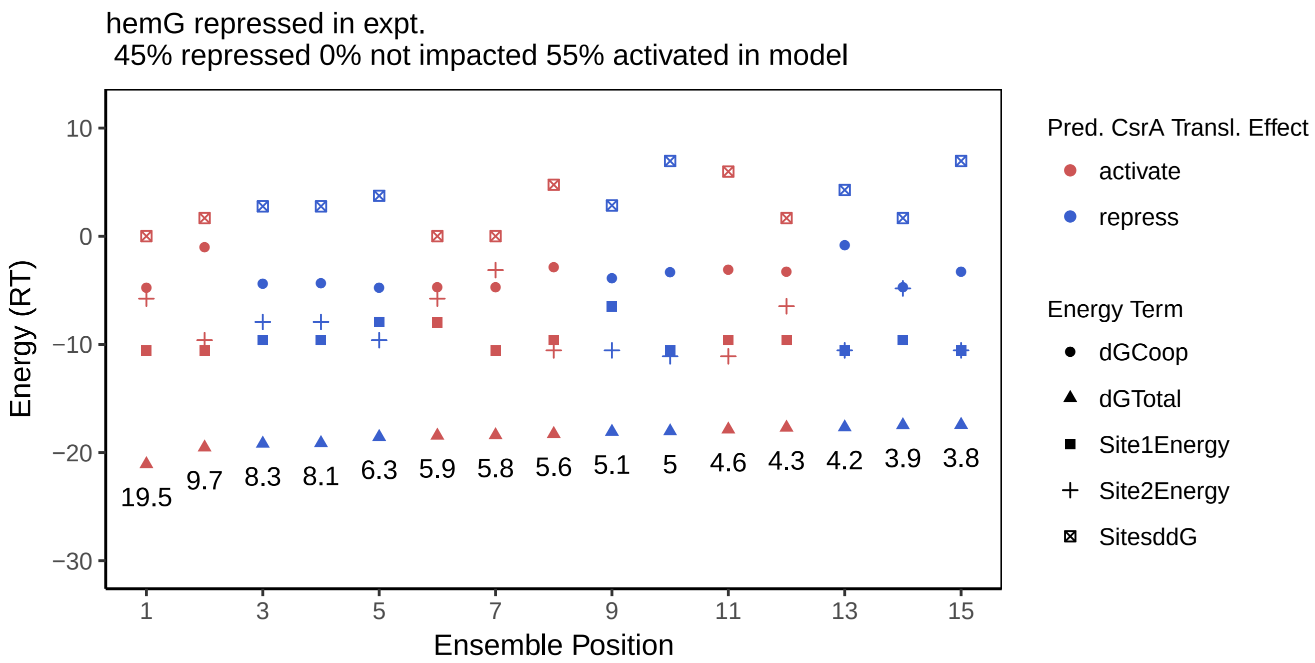
hemG repressed in expt.
45% repressed 0% not impacted 55% activated in model
Energy (RT)
Ensemble Position
Pred. CsrA Transl. Effect
activate
repress
Energy Term
dGCoop
dGTotal
Site1Energy
Site2Energy
SitesddG
19.5
9.7
8.3
8.1
6.3
5.9
5.8
5.6
5.1
5
4.6
4.3
4.2
3.9
3.8

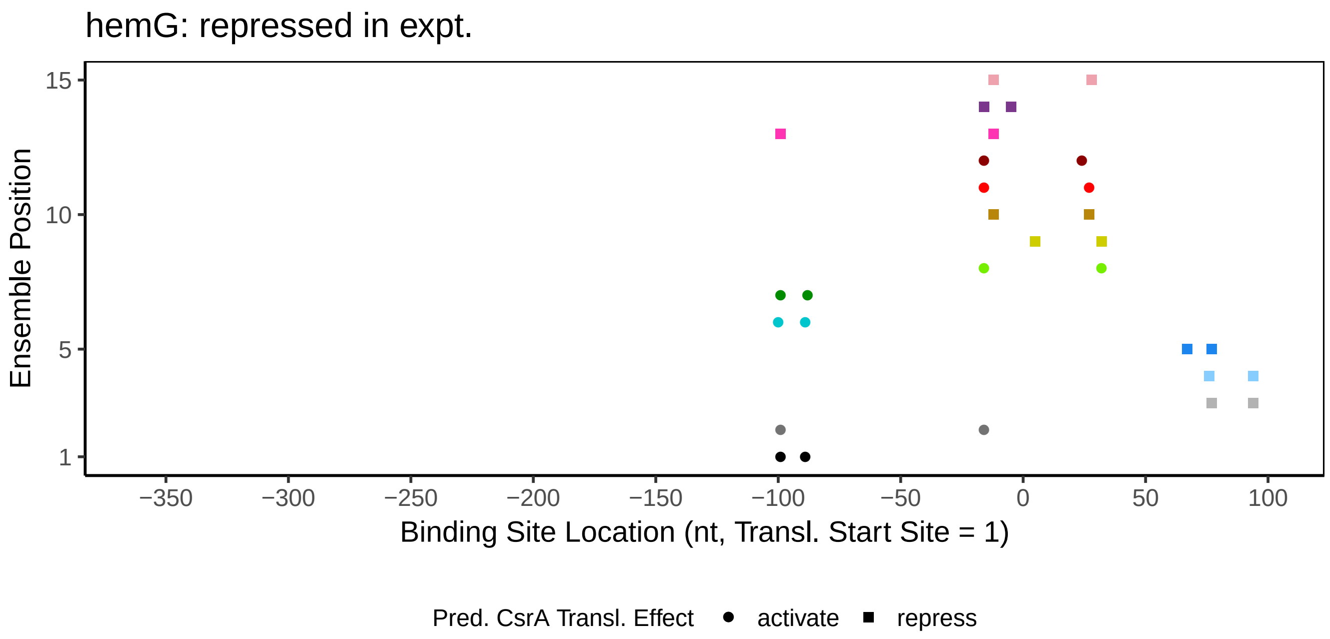
hemG: repressed in expt.
Ensemble Position
Binding Site Location (nt, Transl. Start Site = 1)
Pred. CsrA Transl. Effect
activate
repress

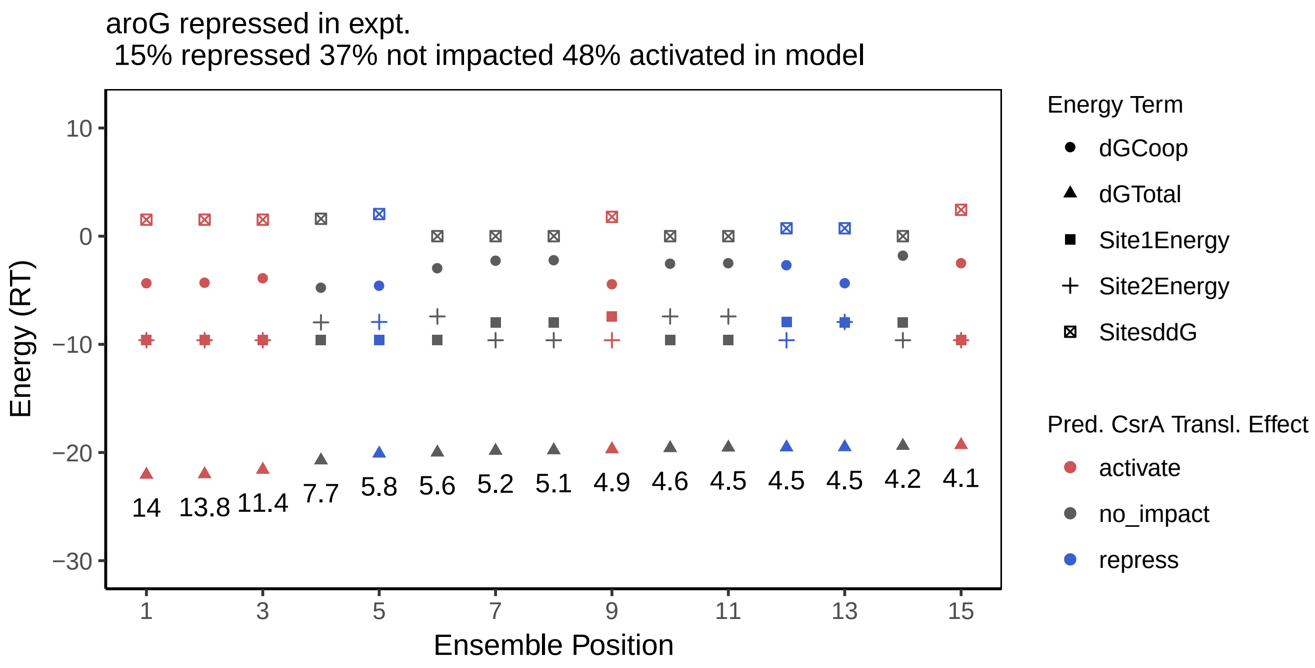
aroG repressed in expt.
15% repressed 37% not impacted 48% activated in model
Energy (RT)
10
0
−10
−20
−30
14
13.8
11.4
7.7
5.8
5.6
5.2
5.1
4.9
4.6
4.5
4.5
4.5
4.2
4.1
1
3
5
7
9
11
13
15
Ensemble Position
Energy Term
dGCoop
dGTotal
Site1Energy
Site2Energy
SitesddG
Pred. CsrA Transl. Effect
activate
no_impact
repress

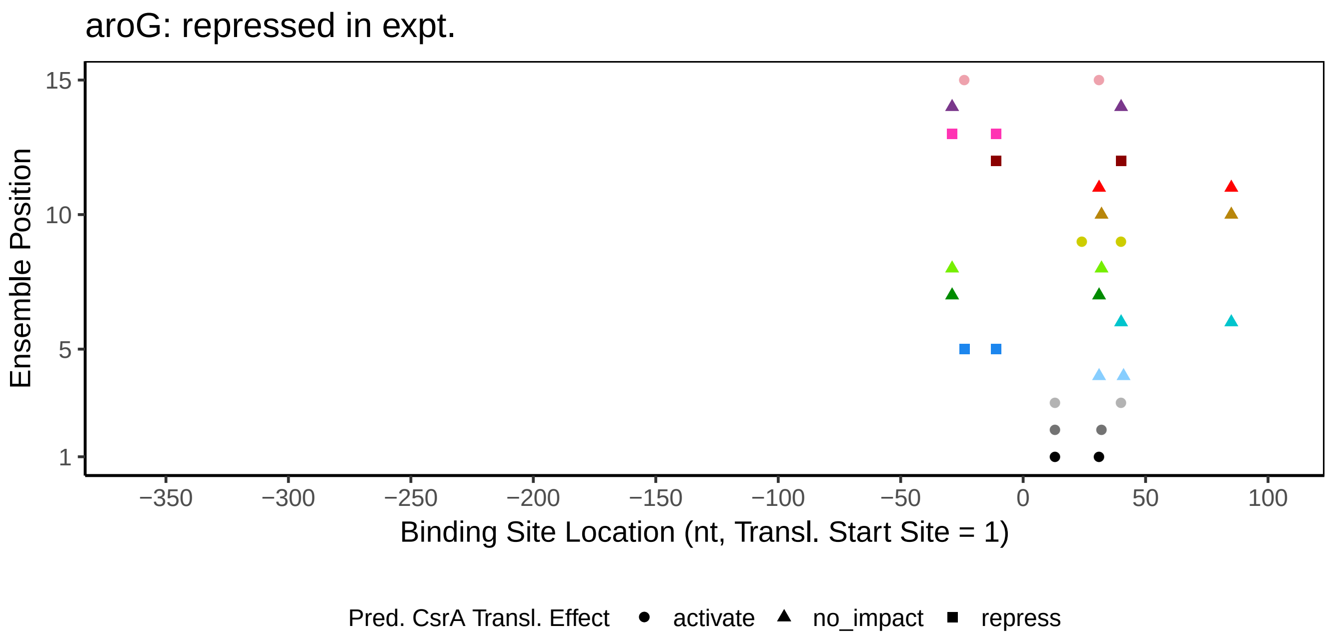
aroG: repressed in expt.
Ensemble Position
15
10
5
1
−350
−300
−250
−200
−150
−100
−50
0
50
100
Binding Site Location (nt, Transl. Start Site = 1)
Pred. CsrA Transl. Effect
activate
no_impact
repress

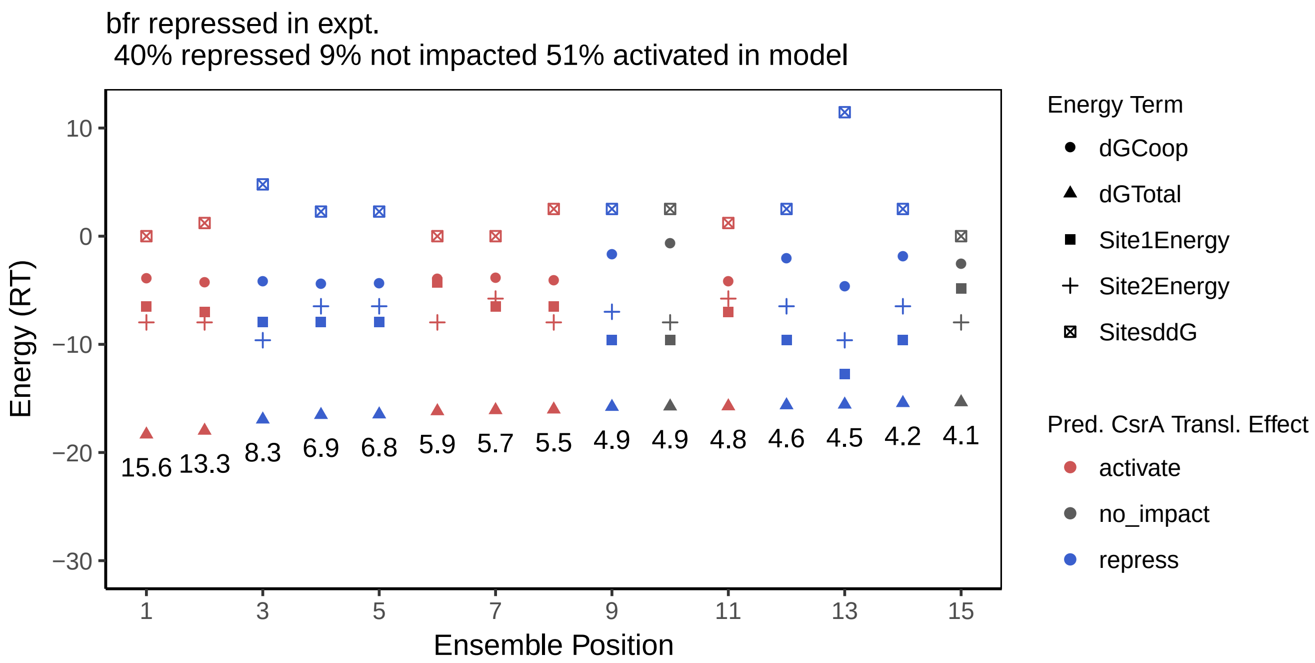

bfr repressed in expt.
40% repressed 9% not impacted 51% activated in model
Energy (RT)
10
0
−10
−20
−30
15.6 13.3 8.3 6.9 6.8 5.9 5.7 5.5 4.9 4.9 4.8 4.6 4.5 4.2 4.1
1 3 5 7 9 11 13 15
Ensemble Position
Energy Term
dGCoop
dGTotal
Site1Energy
Site2Energy
SitesddG
Pred. CsrA Transl. Effect
activate
no_impact
repress

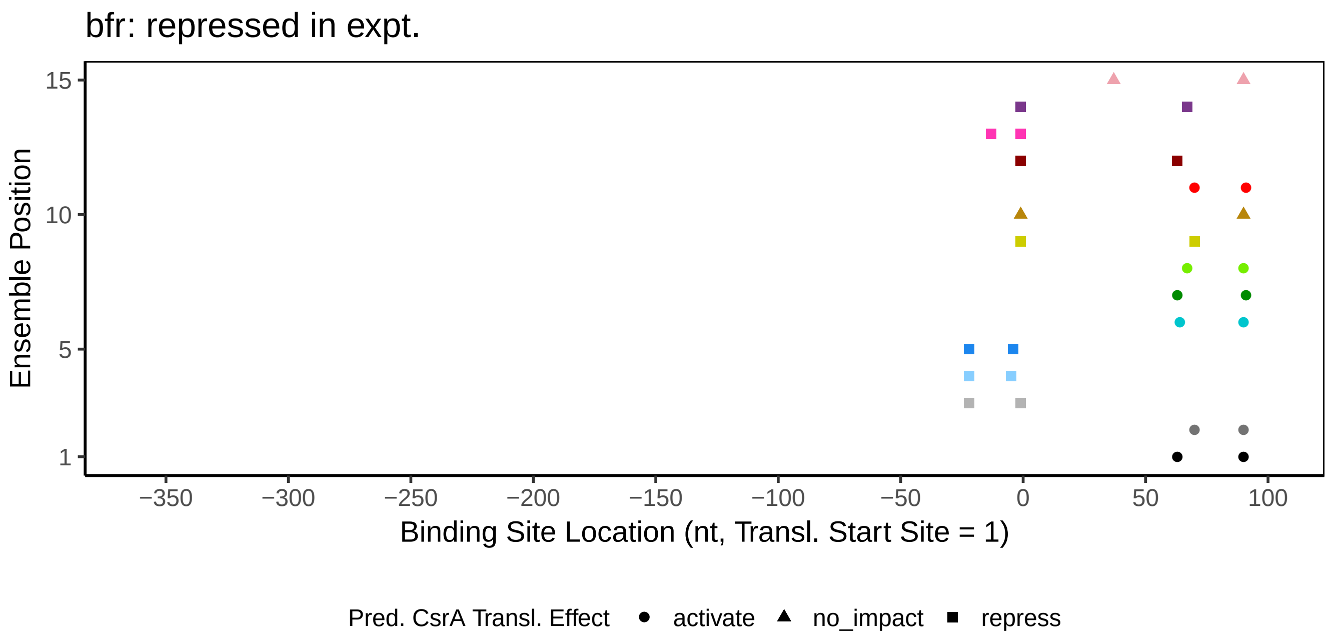

bfr: repressed in expt.
Ensemble Position
15
10
5
1
−350 −300 −250 −200 −150 −100 −50 0 50 100
Binding Site Location (nt, Transl. Start Site = 1)
Pred. CsrA Transl. Effect
activate
no_impact
repress

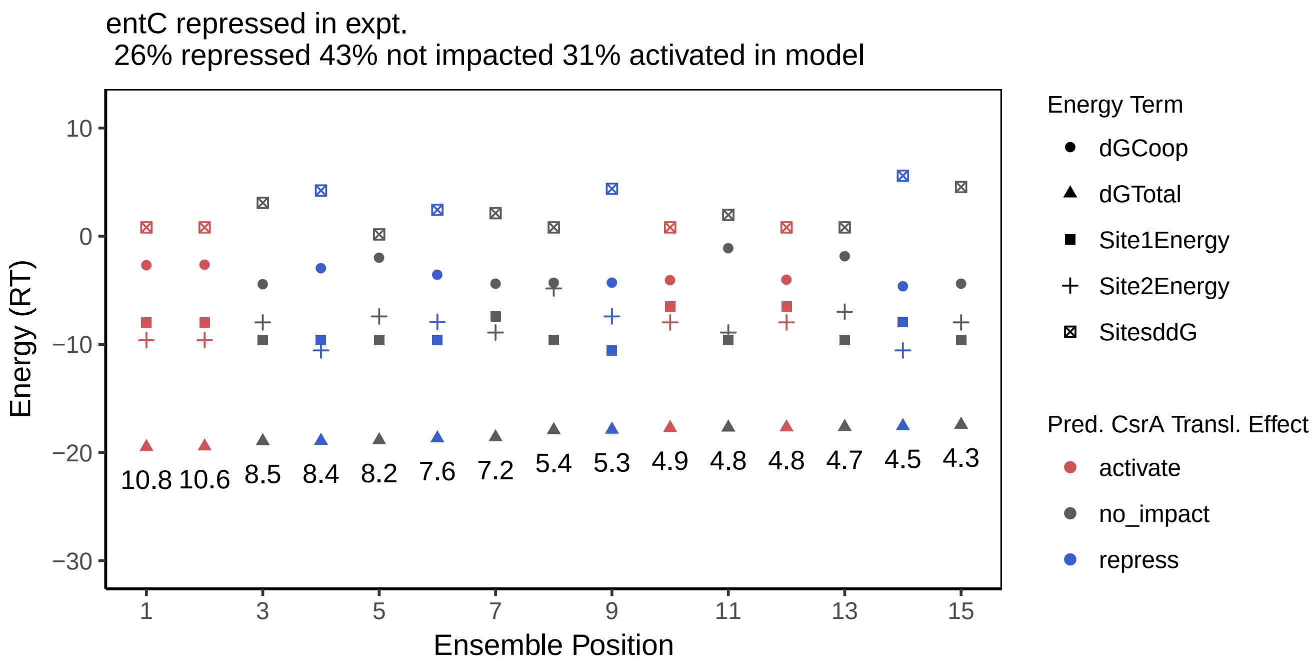

entC repressed in expt.
26% repressed 43% not impacted 31% activated in model
Energy (RT)
10
0
−10
−20
−30
10.8 10.6 8.5 8.4 8.2 7.6 7.2 5.4 5.3 4.9 4.8 4.8 4.7 4.5 4.3
1
3
5
7
9
11
13
15
Ensemble Position
Energy Term
dGCoop
dGTotal
Site1Energy
Site2Energy
SitesddG
Pred. CsrA Transl. Effect
activate
no_impact
repress

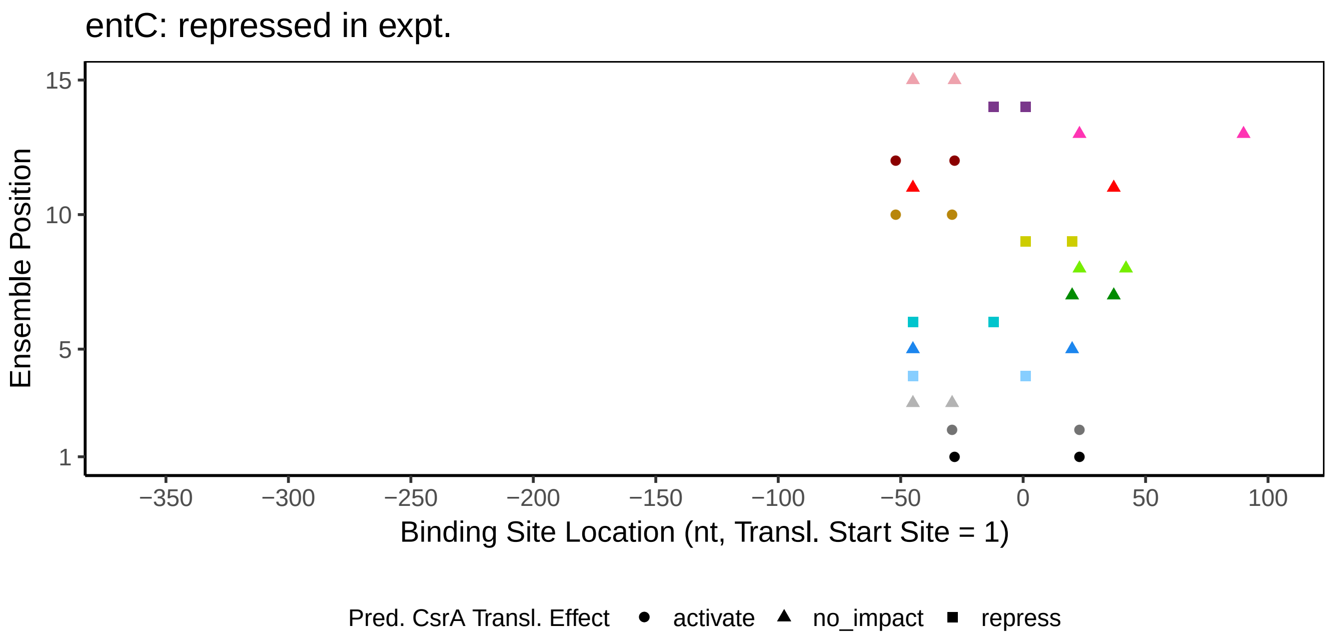

entC: repressed in expt.
Ensemble Position
15
10
5
1
−350
−300
−250
−200
−150
−100
−50
0
50
100
Binding Site Location (nt, Transl. Start Site = 1)
Pred. CsrA Transl. Effect
activate
no_impact
repress

uxaA repressed in expt.
32% repressed 6% not impacted 62% activated in model

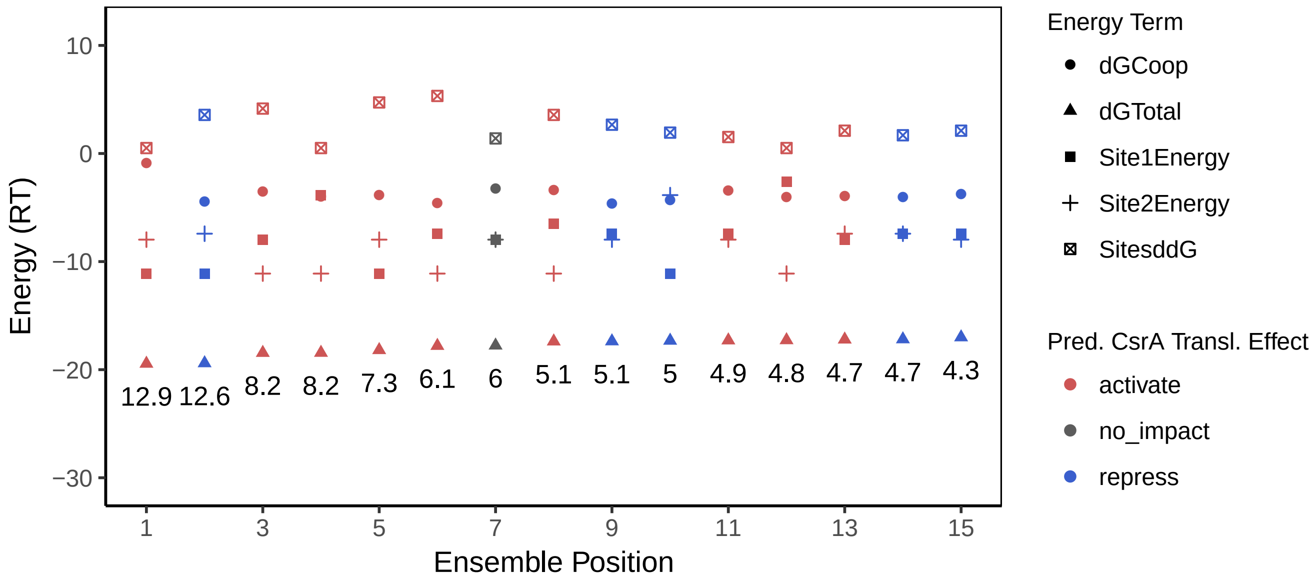

uxaA: repressed in expt.

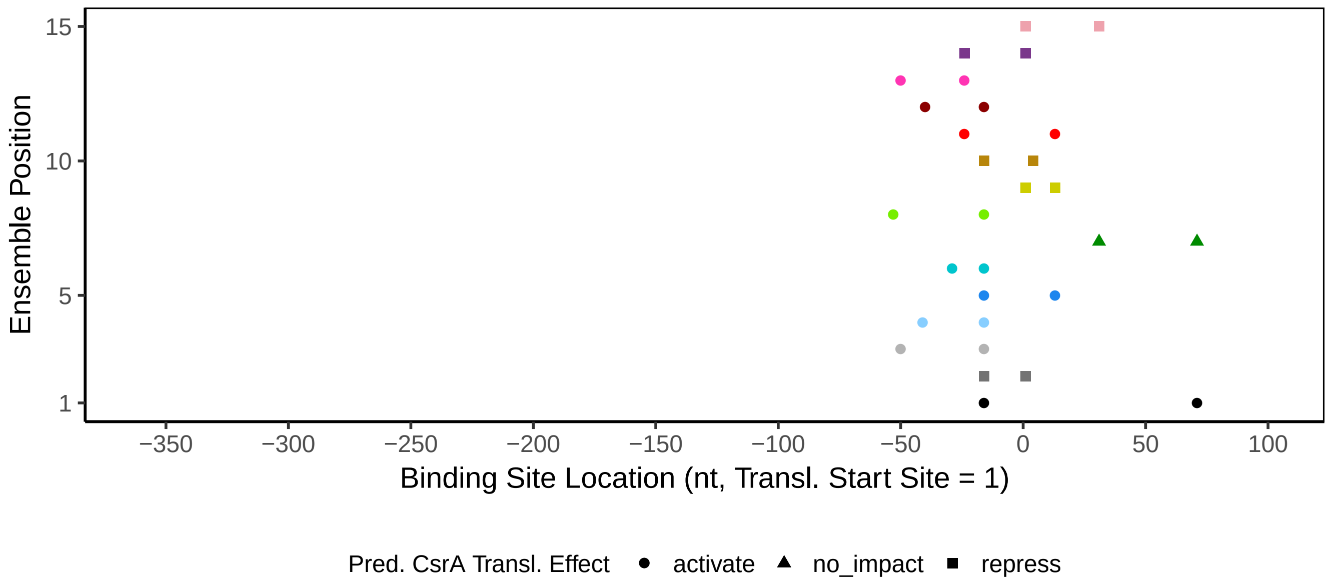

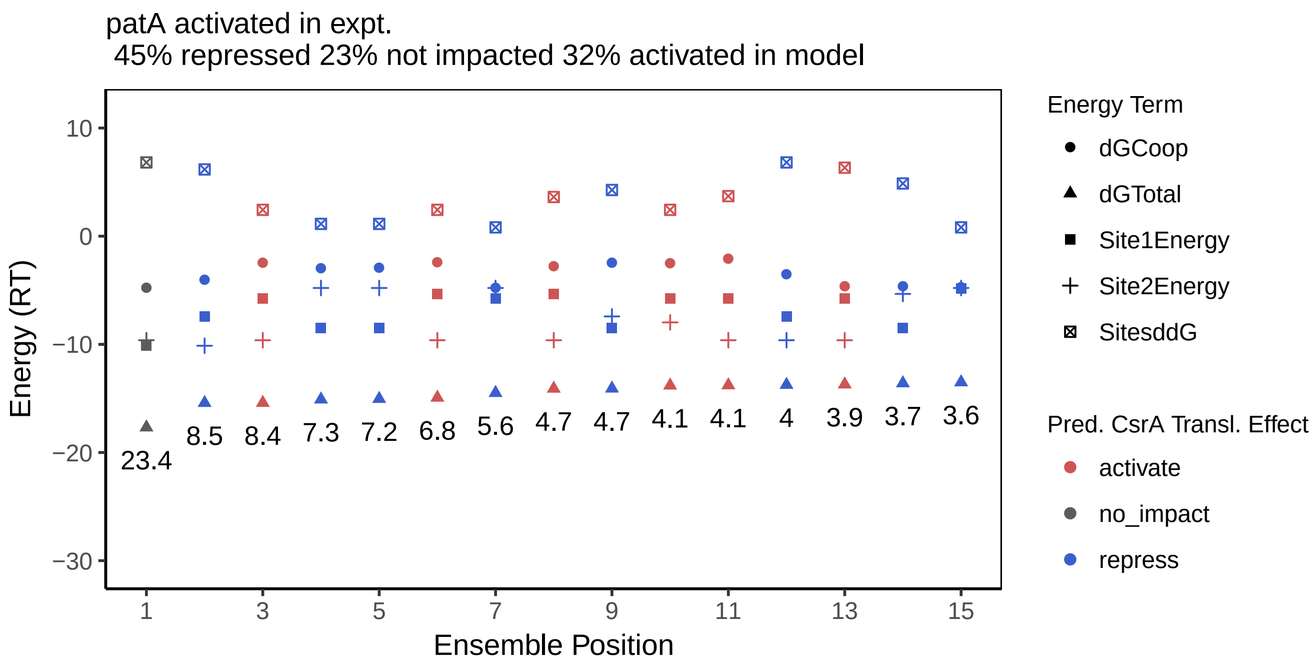
patA activated in expt.
45% repressed 23% not impacted 32% activated in model
Energy (RT)
10
0
−10
−20
−30
23.4
8.5
8.4
7.3
7.2
6.8
5.6
4.7
4.7
4.1
4.1
4
3.9
3.7
3.6
1
3
5
7
9
11
13
15
Ensemble Position
Energy Term
dGCoop
dGTotal
Site1Energy
Site2Energy
SitesddG
Pred. CsrA Transl. Effect
activate
no_impact
repress

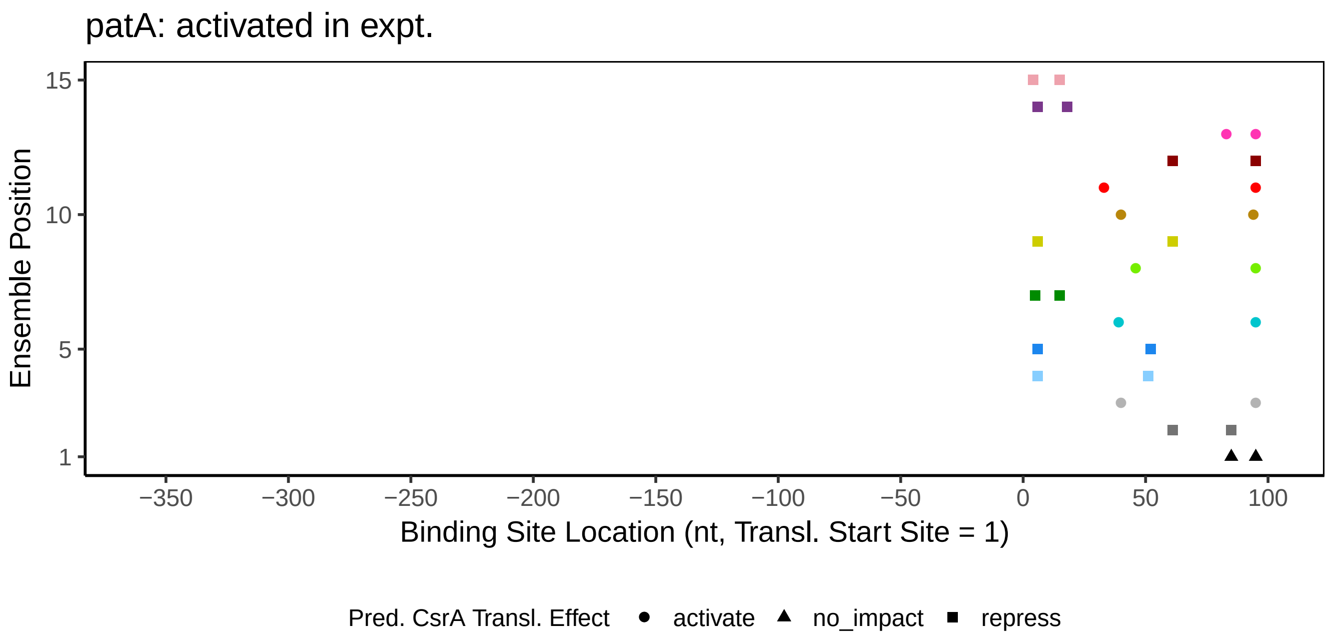
patA: activated in expt.
Ensemble Position
15
10
5
1
−350
−300
−250
−200
−150
−100
−50
0
50
100
Binding Site Location (nt, Transl. Start Site = 1)
Pred. CsrA Transl. Effect
activate
no_impact
repress

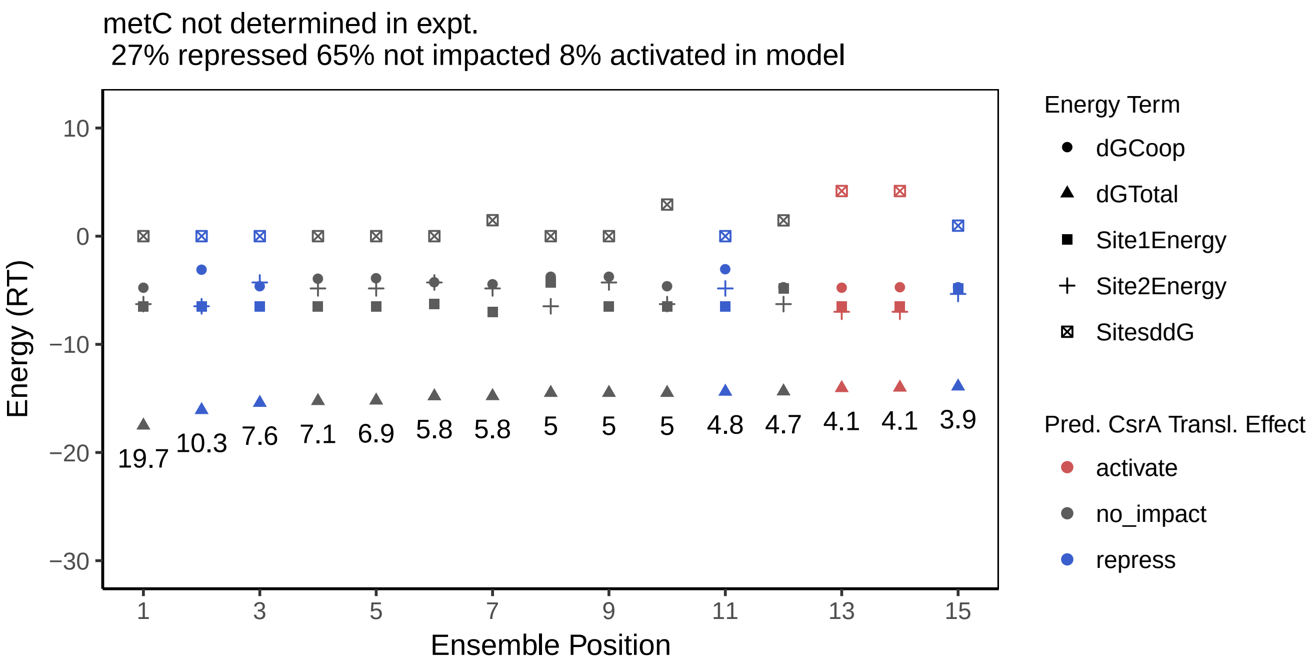
metC not determined in expt.
27% repressed 65% not impacted 8% activated in model
Energy (RT)
Ensemble Position
19.7
10.3
7.6
7.1
6.9
5.8
5.8
5
5
5
4.8
4.7
4.1
4.1
3.9
Energy Term
dGCoop
dGTotal
Site1Energy
Site2Energy
SitesddG
Pred. CsrA Transl. Effect
activate
no_impact
repress

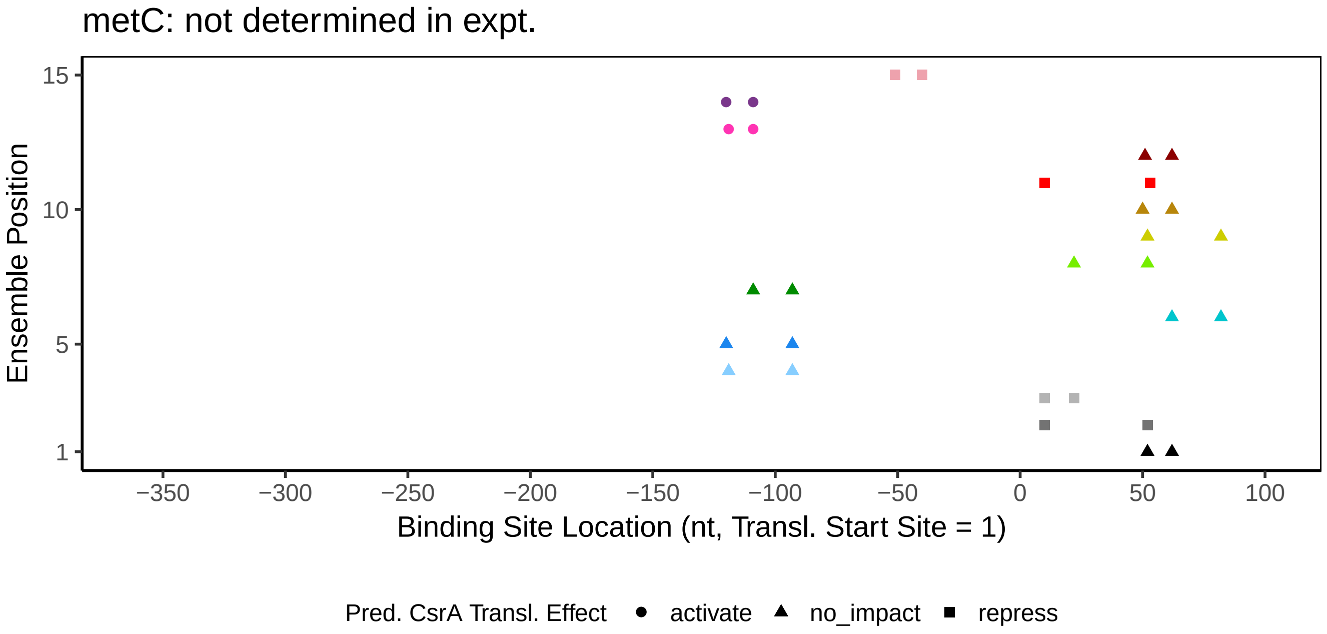
metC: not determined in expt.
Ensemble Position
Binding Site Location (nt, Transl. Start Site = 1)
Pred. CsrA Transl. Effect
activate
no_impact
repress

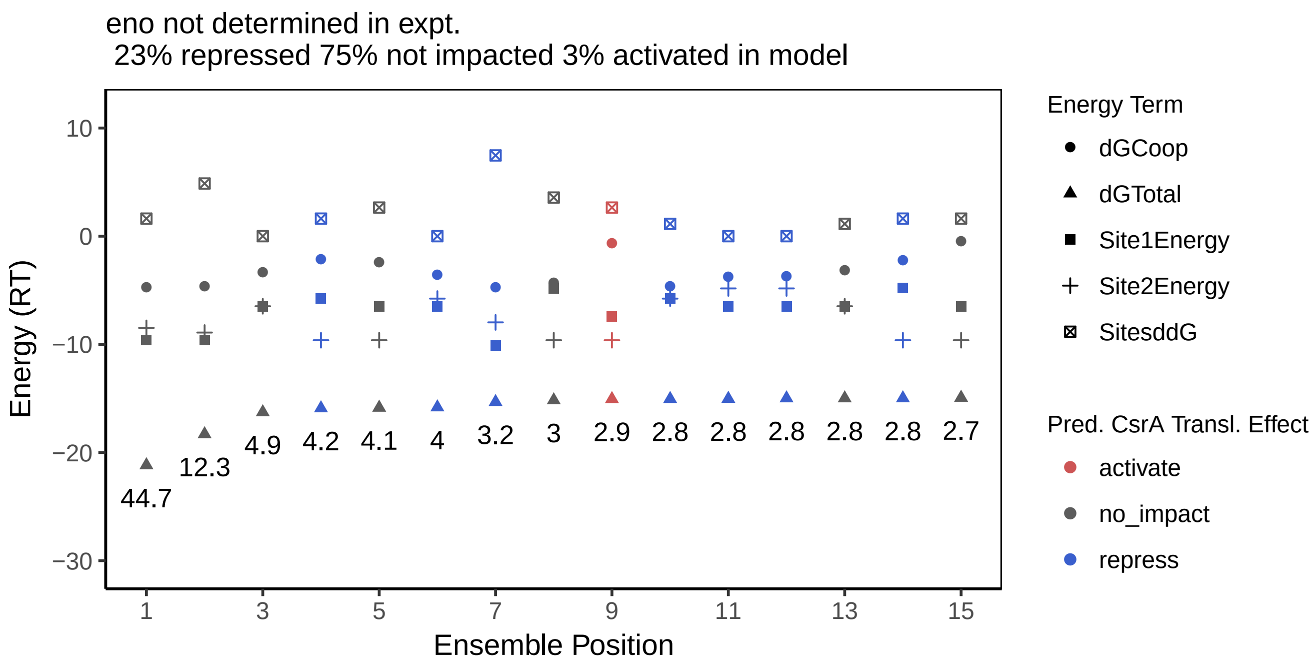
eno not determined in expt.
23% repressed 75% not impacted 3% activated in model
Energy (RT)
Ensemble Position
44.7
12.3
4.9
4.2
4.1
4
3.2
3
2.9
2.8
2.8
2.8
2.8
2.8
2.7
Energy Term
dGCoop
dGTotal
Site1Energy
Site2Energy
SitesddG
Pred. CsrA Transl. Effect
activate
no_impact
repress

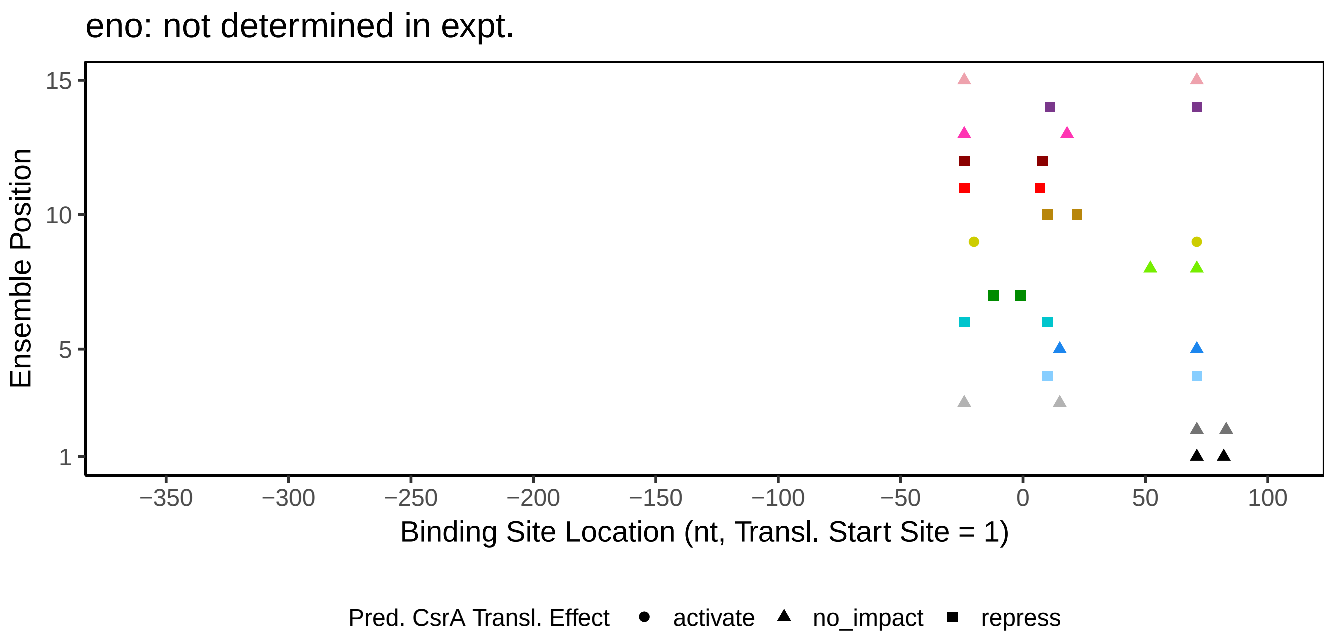
eno: not determined in expt.
Ensemble Position
Binding Site Location (nt, Transl. Start Site = 1)
Pred. CsrA Transl. Effect
activate
no_impact
repress

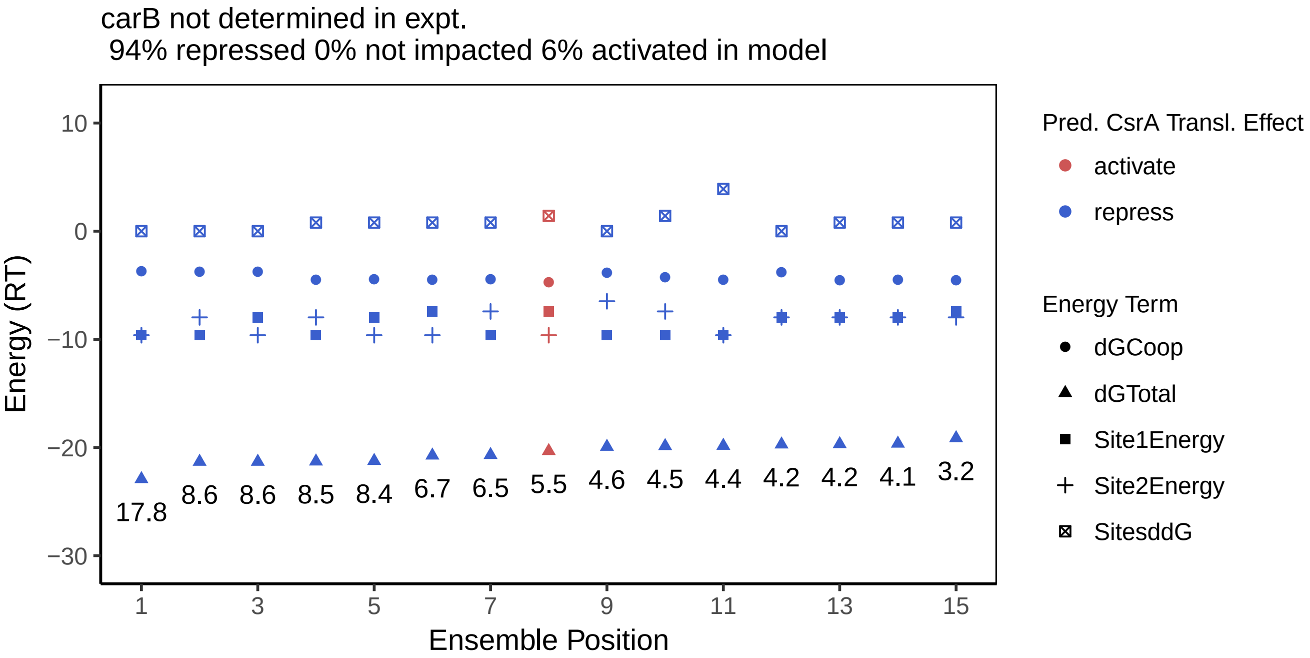
carB not determined in expt.
94% repressed 0% not impacted 6% activated in model
Energy (RT)
10
0
−10
−20
−30
17.8
8.6
8.6
8.5
8.4
6.7
6.5
5.5
4.6
4.5
4.4
4.2
4.2
4.1
3.2
1
3
5
7
9
11
13
15
Ensemble Position
Pred. CsrA Transl. Effect
activate
repress
Energy Term
dGCoop
dGTotal
Site1Energy
Site2Energy
SitesddG

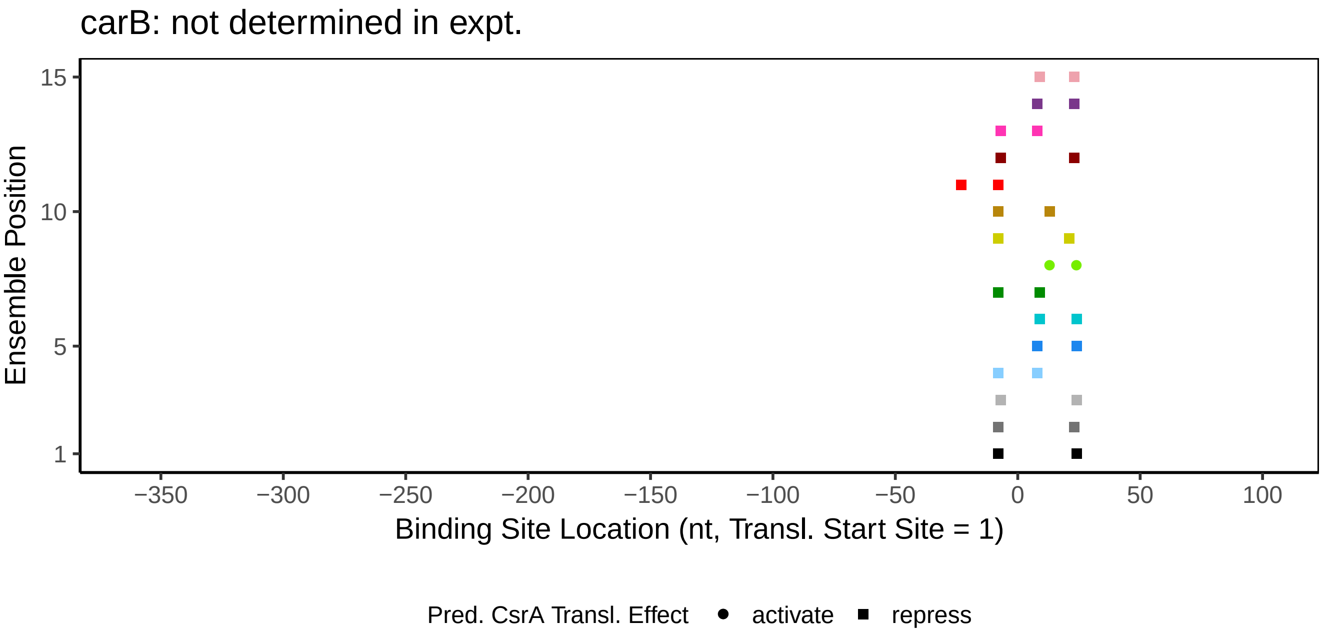
carB: not determined in expt.
Ensemble Position
15
10
5
1
−350
−300
−250
−200
−150
−100
−50
0
50
100
Binding Site Location (nt, Transl. Start Site = 1)
Pred. CsrA Transl. Effect
activate
repress

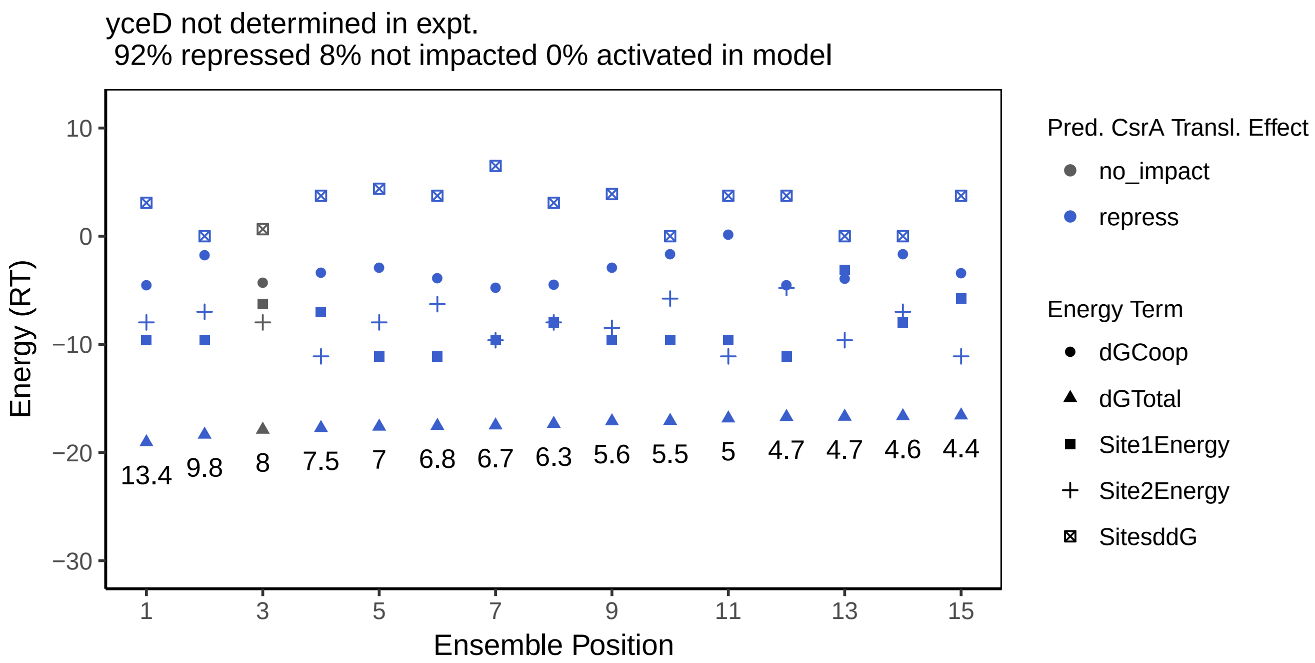
yceD not determined in expt.
92% repressed 8% not impacted 0% activated in model
Energy (RT)
Ensemble Position
13.4
9.8
8
7.5
7
6.8
6.7
6.3
5.6
5.5
5
4.7
4.7
4.6
4.4
Pred. CsrA Transl. Effect
no_impact
repress
Energy Term
dGCoop
dGTotal
Site1Energy
Site2Energy
SitesddG

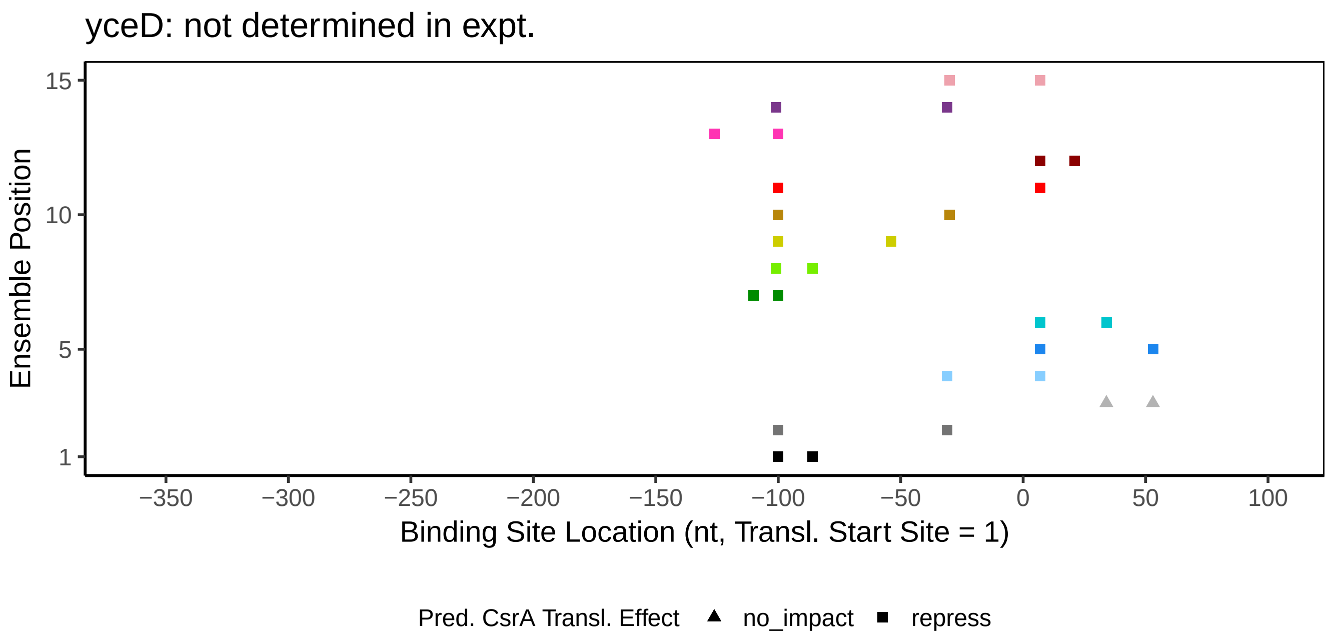
yceD: not determined in expt.
Ensemble Position
Binding Site Location (nt, Transl. Start Site = 1)
Pred. CsrA Transl. Effect
no_impact
repress

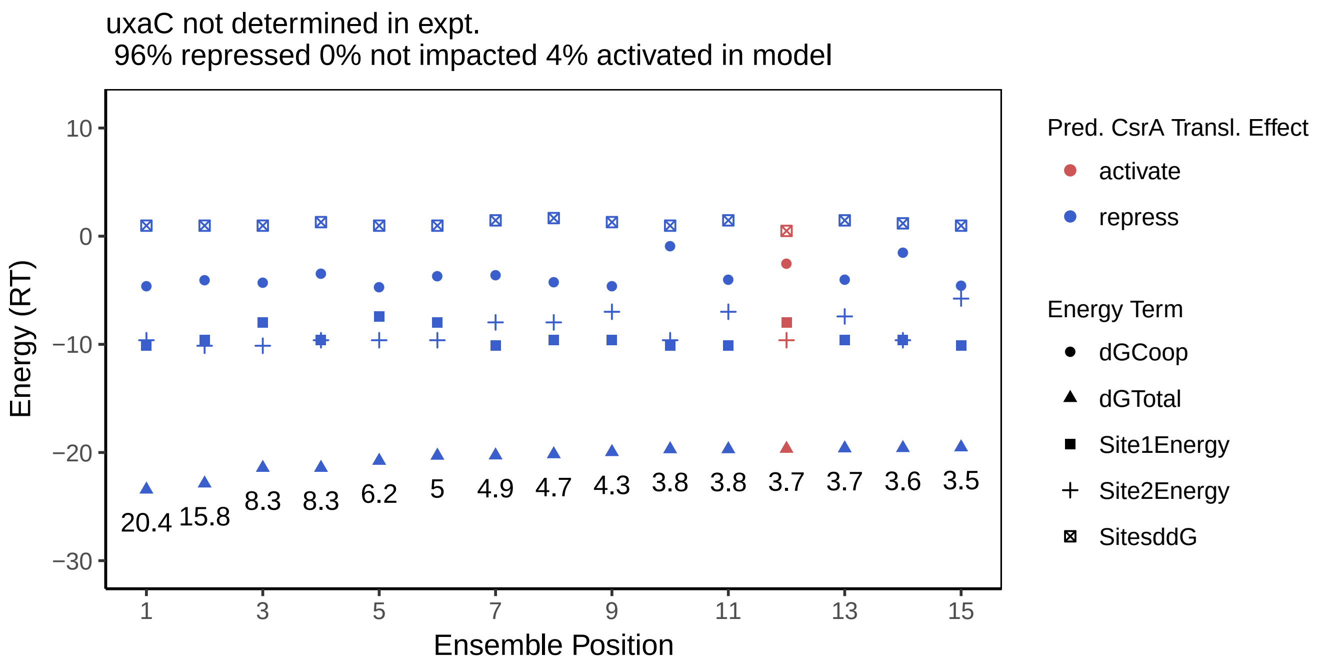
uxaC not determined in expt.
96% repressed 0% not impacted 4% activated in model
Energy (RT)
10
0
−10
−20
−30
20.4 15.8 8.3 8.3 6.2 5 4.9 4.7 4.3 3.8 3.8 3.7 3.7 3.6 3.5
1 3 5 7 9 11 13 15
Ensemble Position
Pred. CsrA Transl. Effect
activate
repress
Energy Term
dGCoop
dGTotal
Site1Energy
Site2Energy
SitesddG

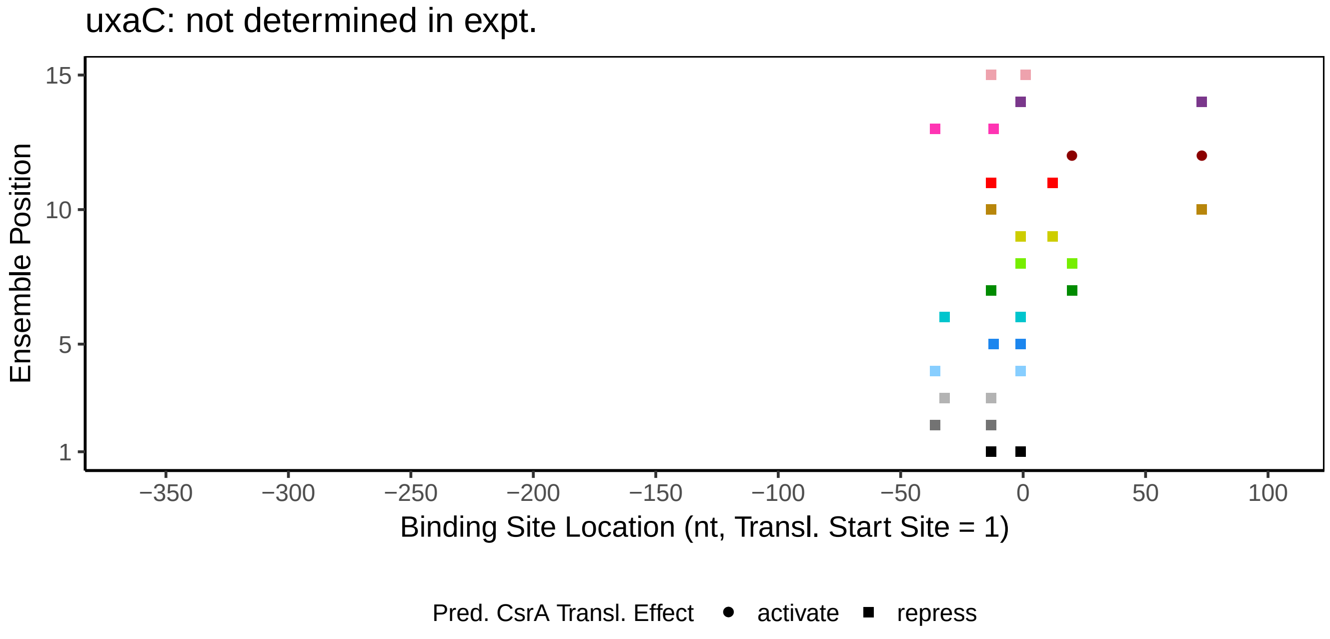
uxaC: not determined in expt.
Ensemble Position
15
10
5
1
−350 −300 −250 −200 −150 −100 −50 0 50 100
Binding Site Location (nt, Transl. Start Site = 1)
Pred. CsrA Transl. Effect
activate
repress

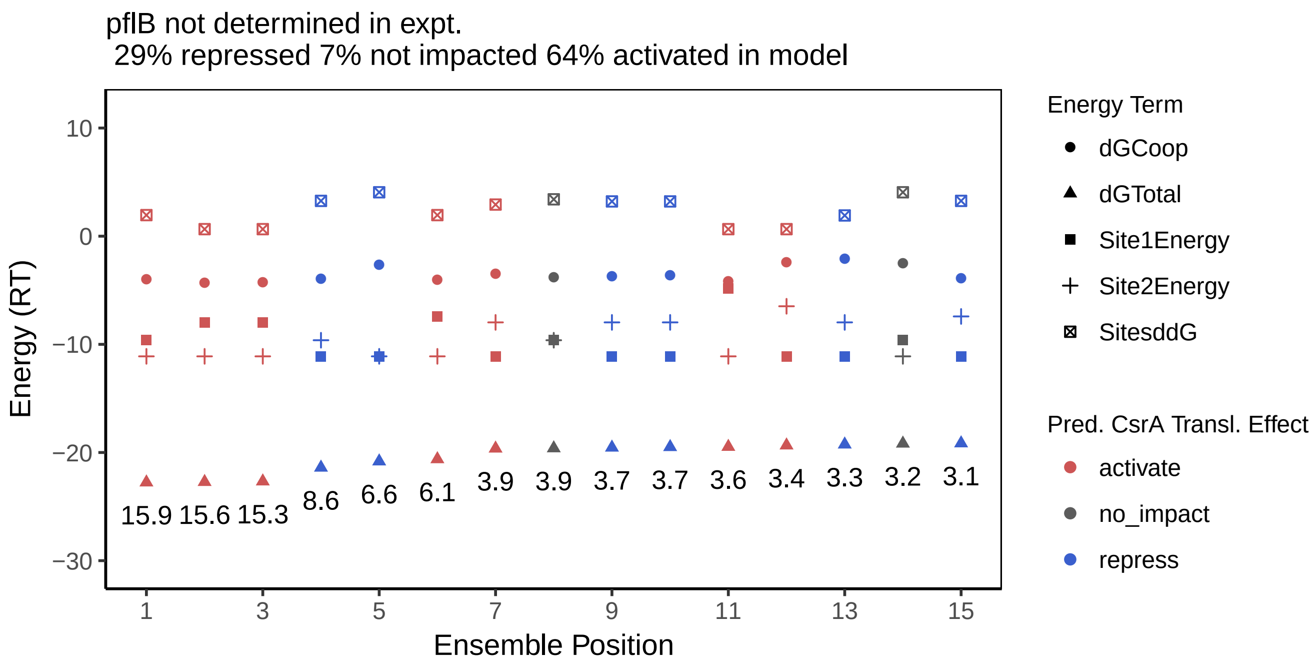
pflB not determined in expt.
29% repressed 7% not impacted 64% activated in model
Energy (RT)
Ensemble Position
15.9 15.6 15.3 8.6 6.6 6.1 3.9 3.9 3.7 3.7 3.6 3.4 3.3 3.2 3.1
Energy Term
dGCoop
dGTotal
Site1Energy
Site2Energy
SitesddG
Pred. CsrA Transl. Effect
activate
no_impact
repress

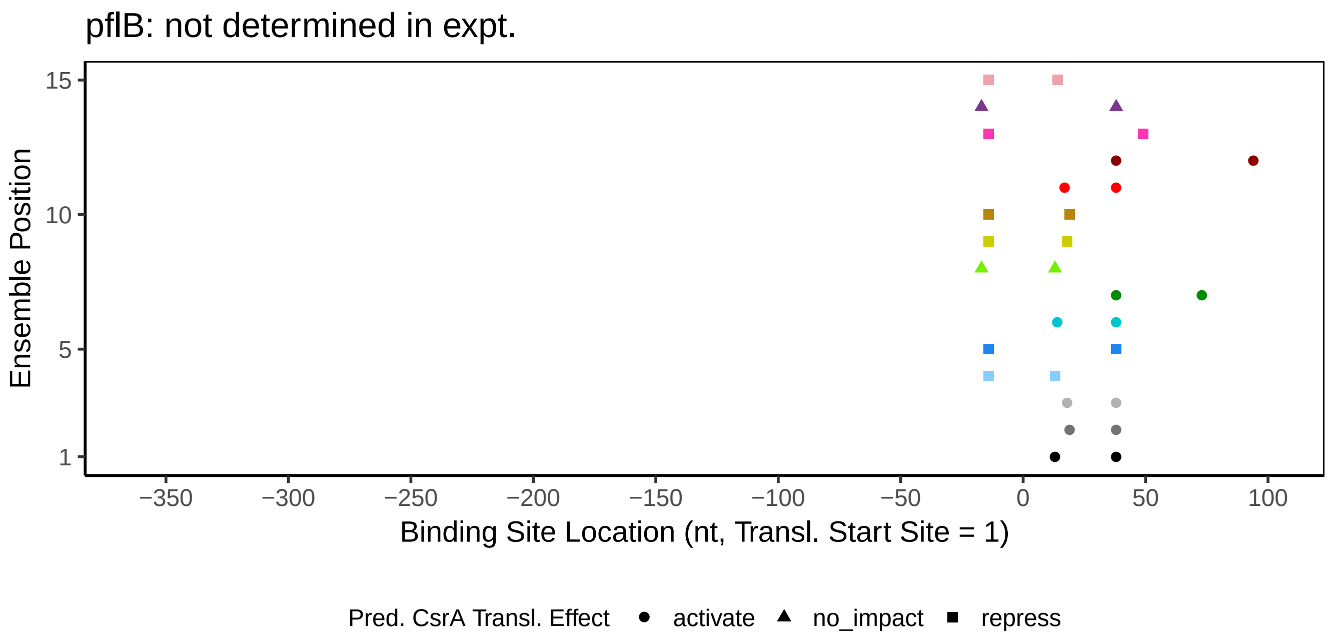
pflB: not determined in expt.
Ensemble Position
Binding Site Location (nt, Transl. Start Site = 1)
Pred. CsrA Transl. Effect
activate
no_impact
repress

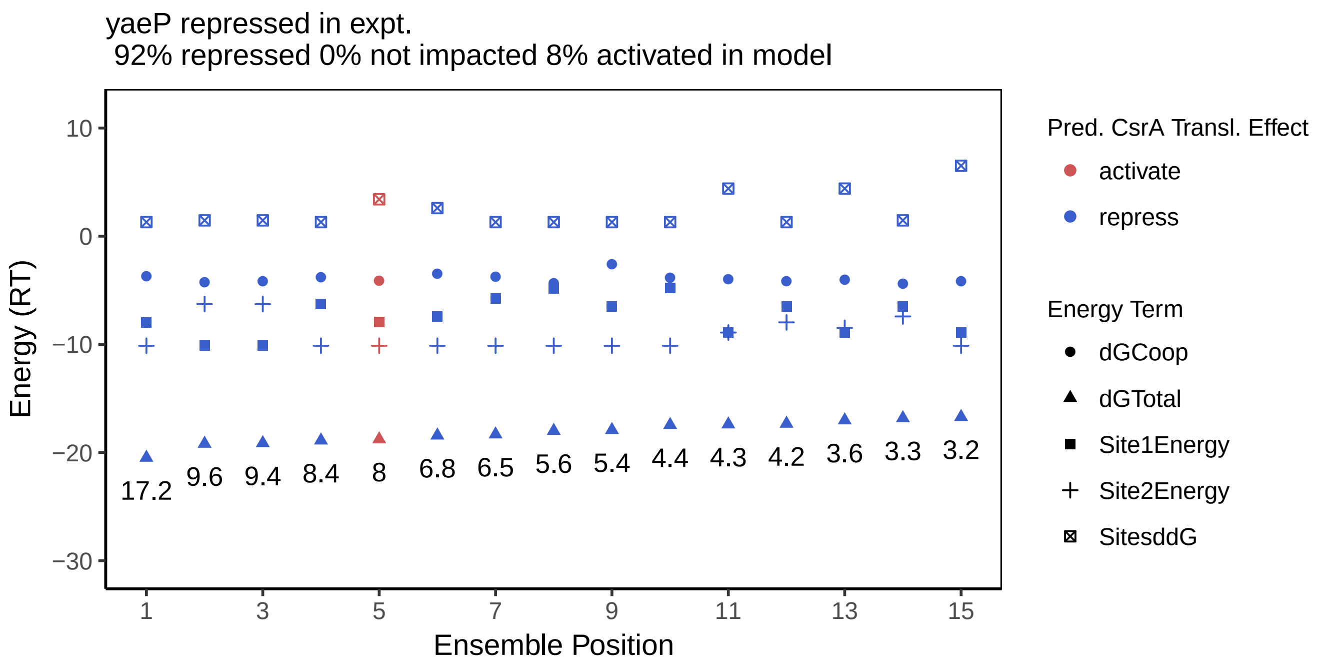

yaeP repressed in expt.
92% repressed 0% not impacted 8% activated in model
Energy (RT)
10
0
−10
−20
−30
17.2
9.6
9.4
8.4
8
6.8
6.5
5.6
5.4
4.4
4.3
4.2
3.6
3.3
3.2
1
3
5
7
9
11
13
15
Ensemble Position
Pred. CsrA Transl. Effect
activate
repress
Energy Term
dGCoop
dGTotal
Site1Energy
Site2Energy
SitesddG

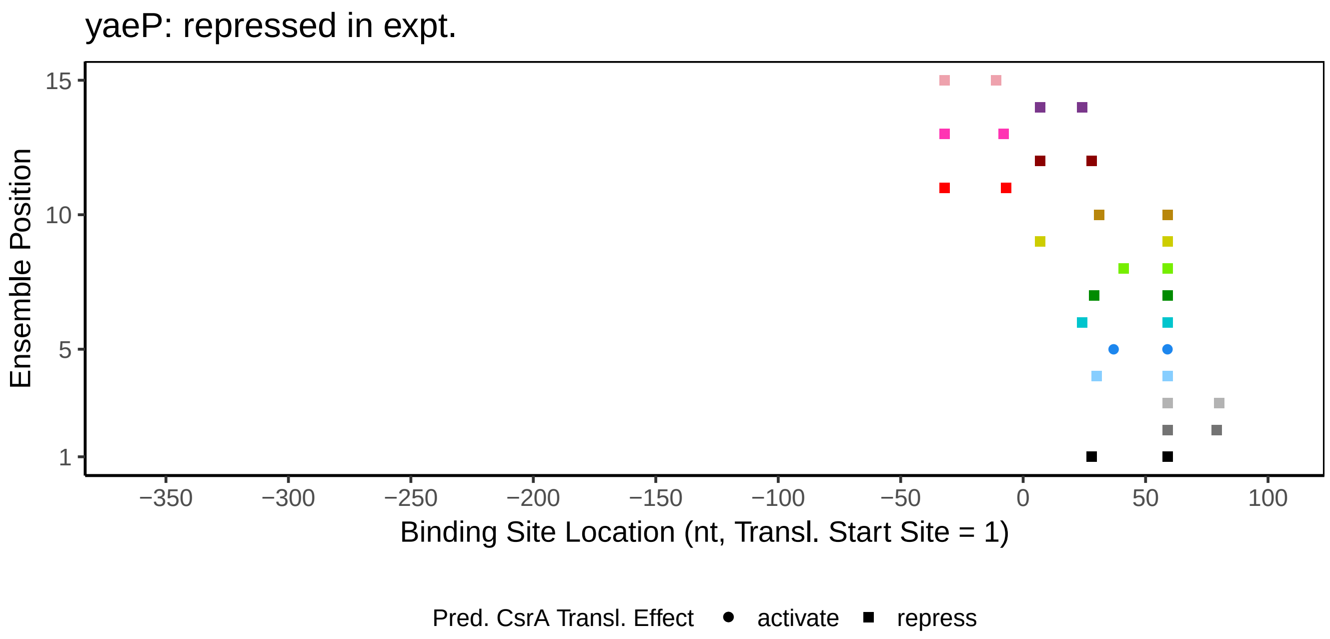

yaeP: repressed in expt.
Ensemble Position
15
10
5
1
−350
−300
−250
−200
−150
−100
−50
0
50
100
Binding Site Location (nt, Transl. Start Site = 1)
Pred. CsrA Transl. Effect
activate
repress

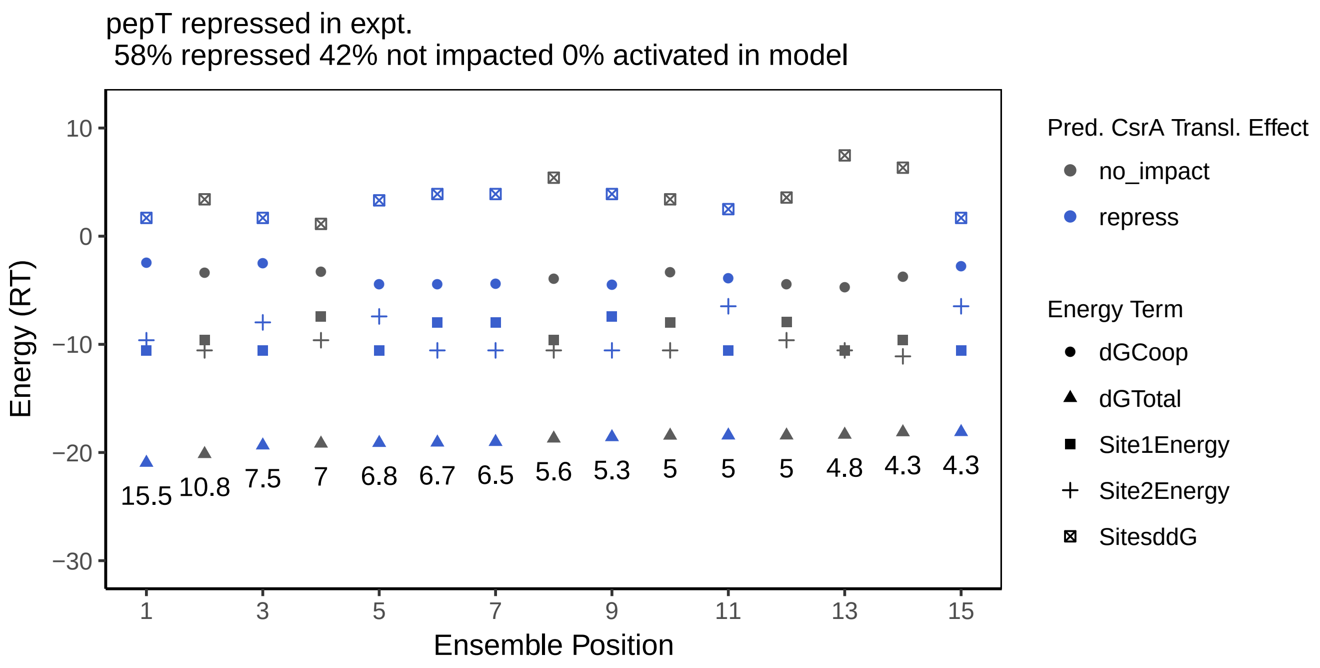
pepT repressed in expt.
58% repressed 42% not impacted 0% activated in model
Energy (RT)
10
0
−10
−20
−30
15.5
10.8
7.5
7
6.8
6.7
6.5
5.6
5.3
5
5
5
4.8
4.3
4.3
1
3
5
7
9
11
13
15
Ensemble Position
Pred. CsrA Transl. Effect
no_impact
repress
Energy Term
dGCoop
dGTotal
Site1Energy
Site2Energy
SitesddG

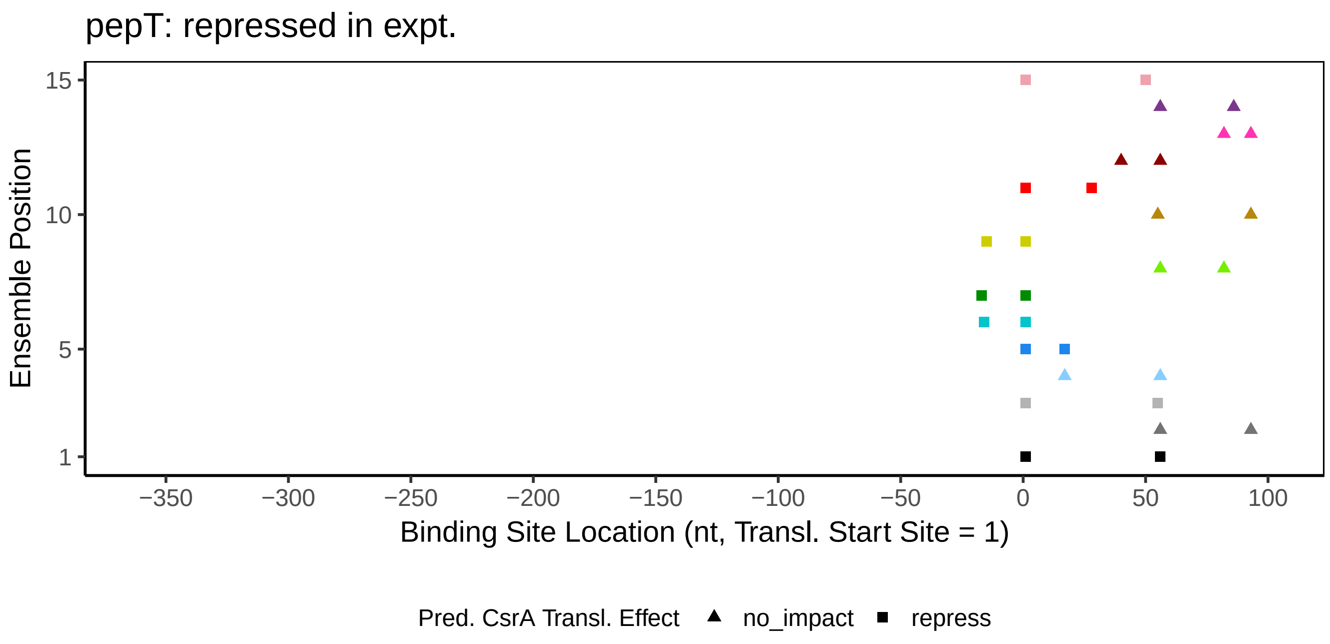
pepT: repressed in expt.
Ensemble Position
15
10
5
1
−350
−300
−250
−200
−150
−100
−50
0
50
100
Binding Site Location (nt, Transl. Start Site = 1)
Pred. CsrA Transl. Effect
no_impact
repress

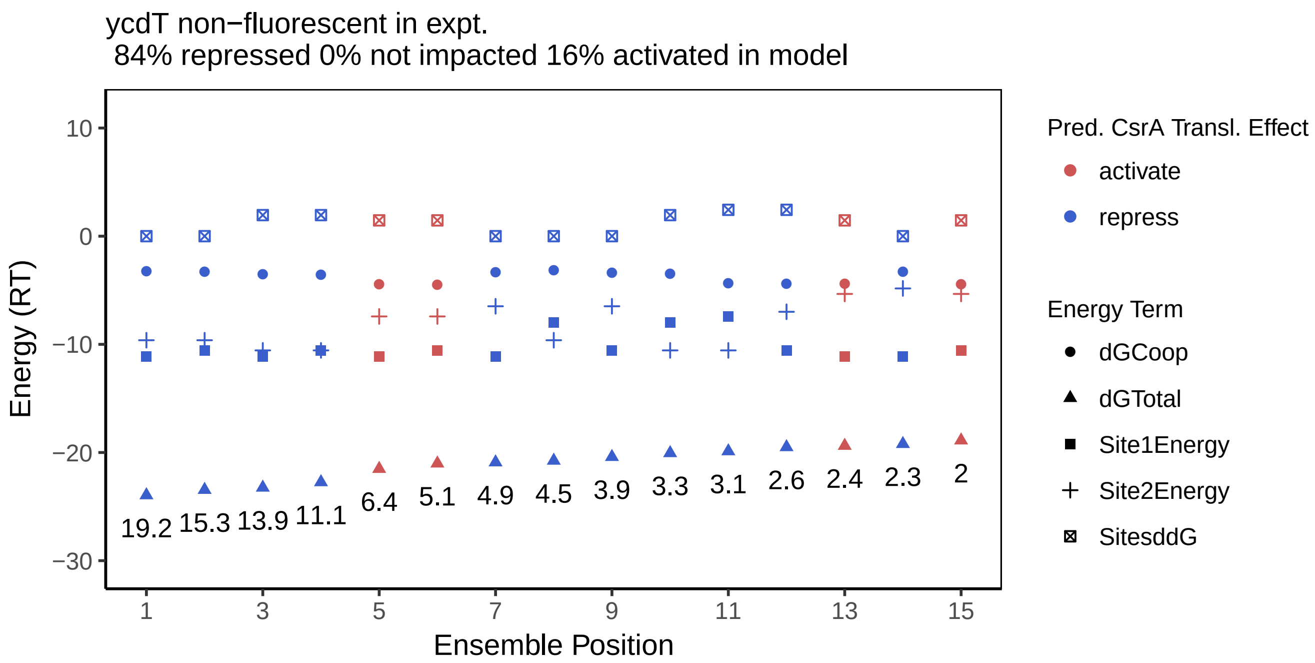
ycdT non−fluorescent in expt.
84% repressed 0% not impacted 16% activated in model
Energy (RT)
10
0
−10
−20
−30
19.2 15.3 13.9 11.1 6.4 5.1 4.9 4.5 3.9 3.3 3.1 2.6 2.4 2.3 2
1 3 5 7 9 11 13 15
Ensemble Position
Pred. CsrA Transl. Effect
activate
repress
Energy Term
dGCoop
dGTotal
Site1Energy
Site2Energy
SitesddG

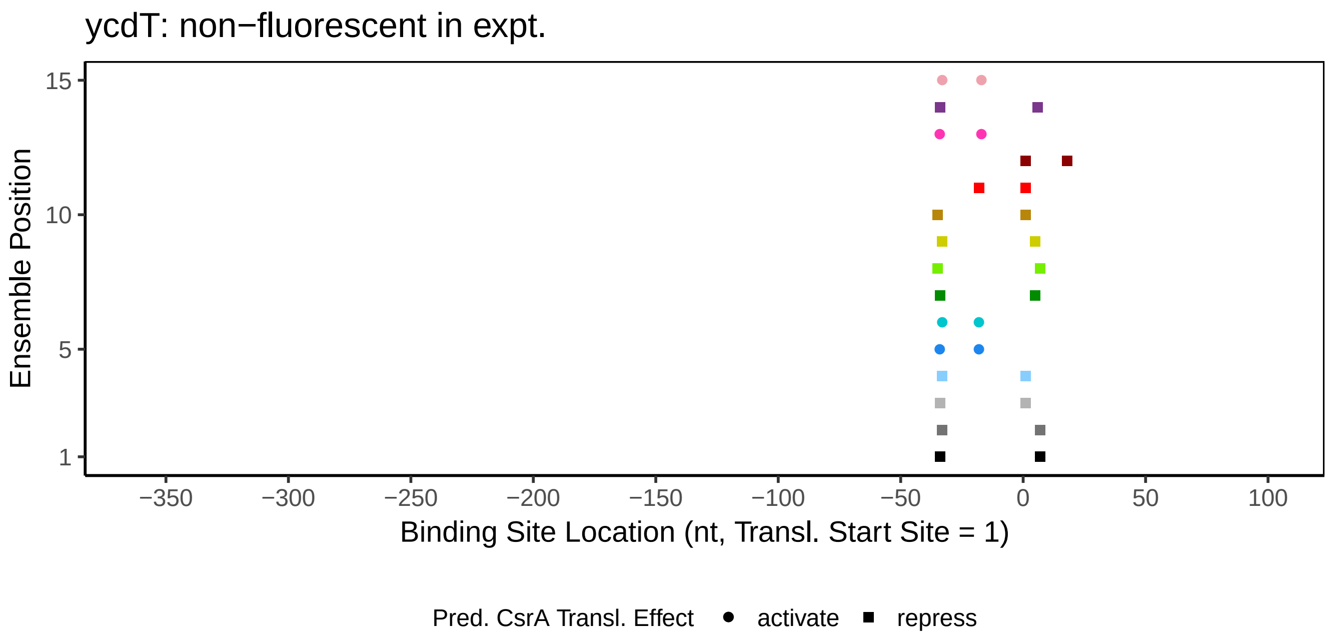
ycdT: non−fluorescent in expt.
Ensemble Position
15
10
5
1
−350 −300 −250 −200 −150 −100 −50 0 50 100
Binding Site Location (nt, Transl. Start Site = 1)
Pred. CsrA Transl. Effect
activate
repress

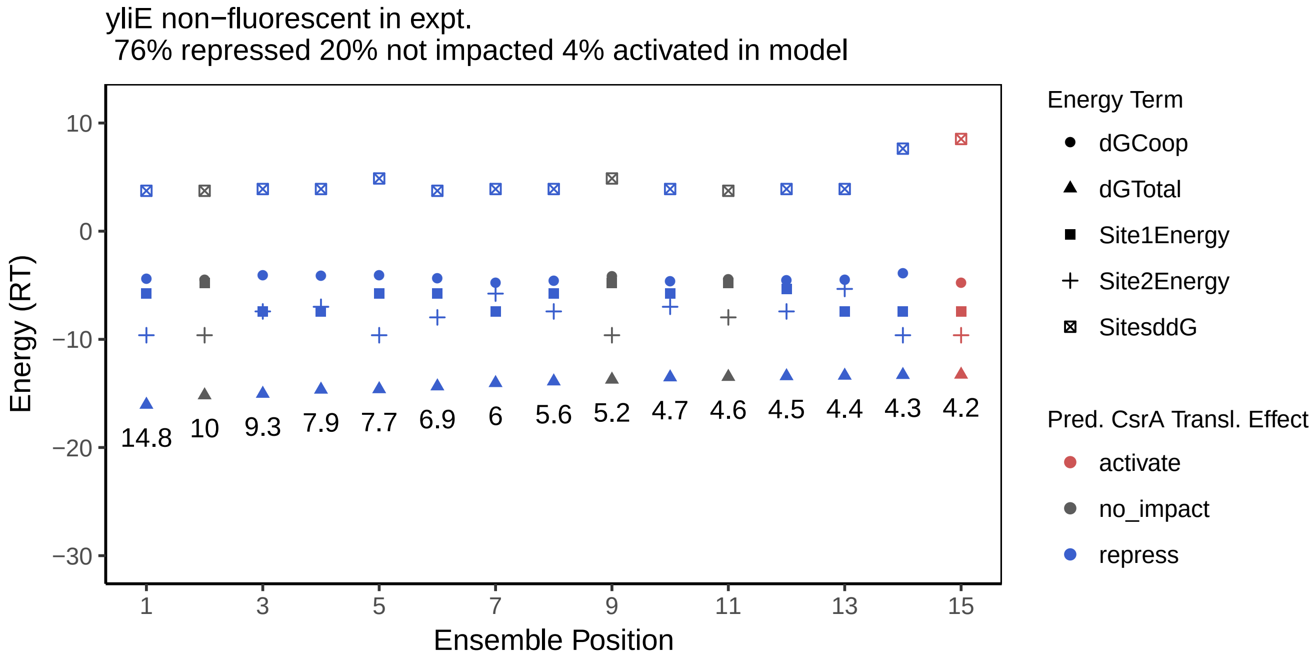

yliE non−fluorescent in expt.
76% repressed 20% not impacted 4% activated in model
Energy (RT)
10
0
−10
−20
−30
14.8
10
9.3
7.9
7.7
6.9
6
5.6
5.2
4.7
4.6
4.5
4.4
4.3
4.2
1
3
5
7
9
11
13
15
Ensemble Position
Energy Term
dGCoop
dGTotal
Site1Energy
Site2Energy
SitesddG
Pred. CsrA Transl. Effect
activate
no_impact
repress

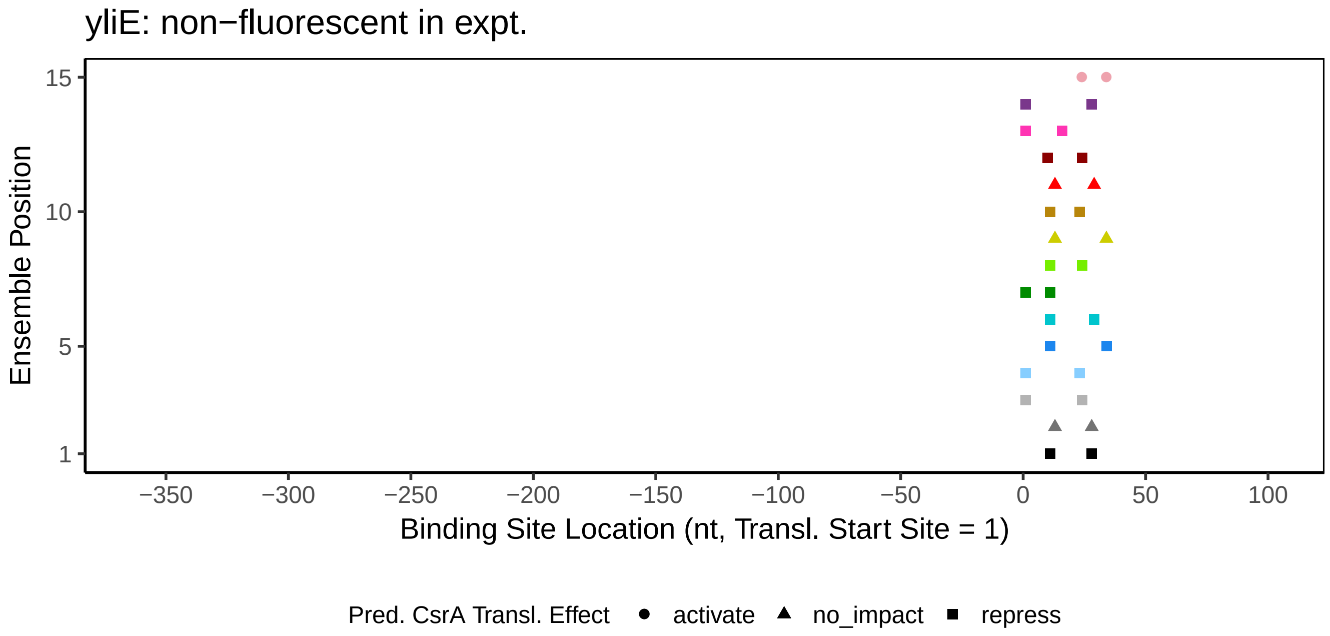

yliE: non−fluorescent in expt.
Ensemble Position
15
10
5
1
−350
−300
−250
−200
−150
−100
−50
0
50
100
Binding Site Location (nt, Transl. Start Site = 1)
Pred. CsrA Transl. Effect
activate
no_impact
repress

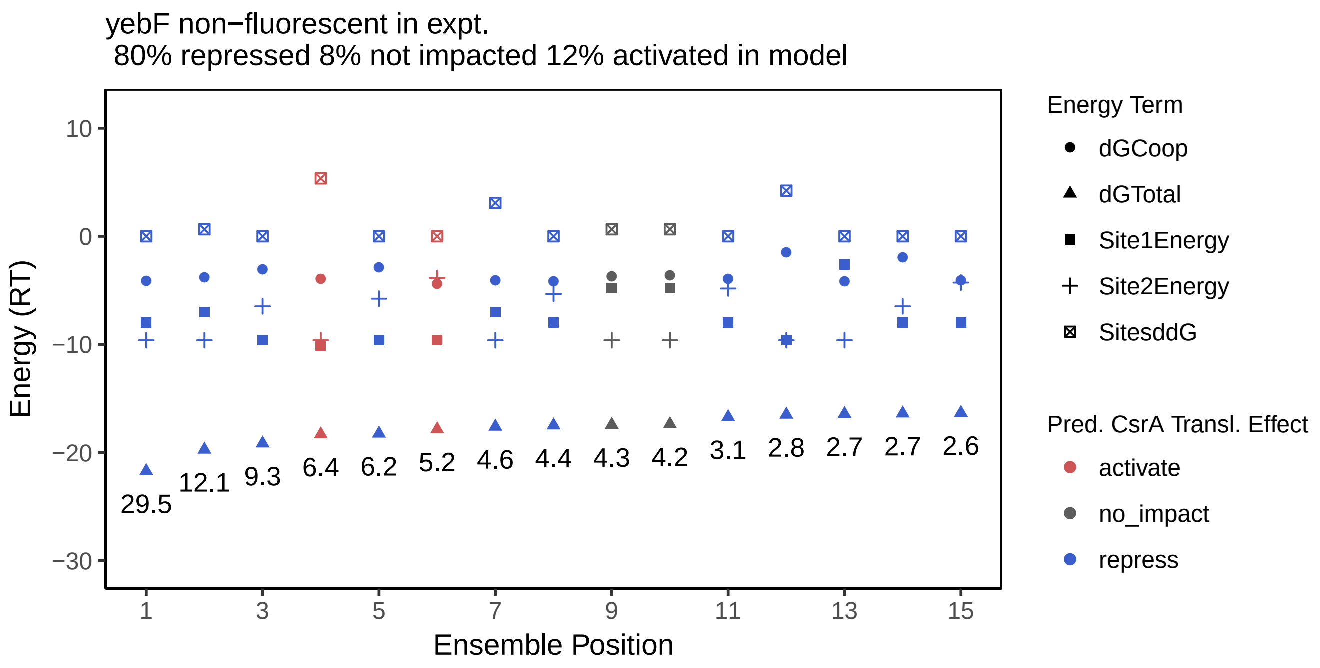

yebF non−fluorescent in expt.
80% repressed 8% not impacted 12% activated in model
Energy (RT)
10
0
−10
−20
−30
29.5
12.1
9.3
6.4
6.2
5.2
4.6
4.4
4.3
4.2
3.1
2.8
2.7
2.7
2.6
1
3
5
7
9
11
13
15
Ensemble Position
Energy Term
dGCoop
dGTotal
Site1Energy
Site2Energy
SitesddG
Pred. CsrA Transl. Effect
activate
no_impact
repress

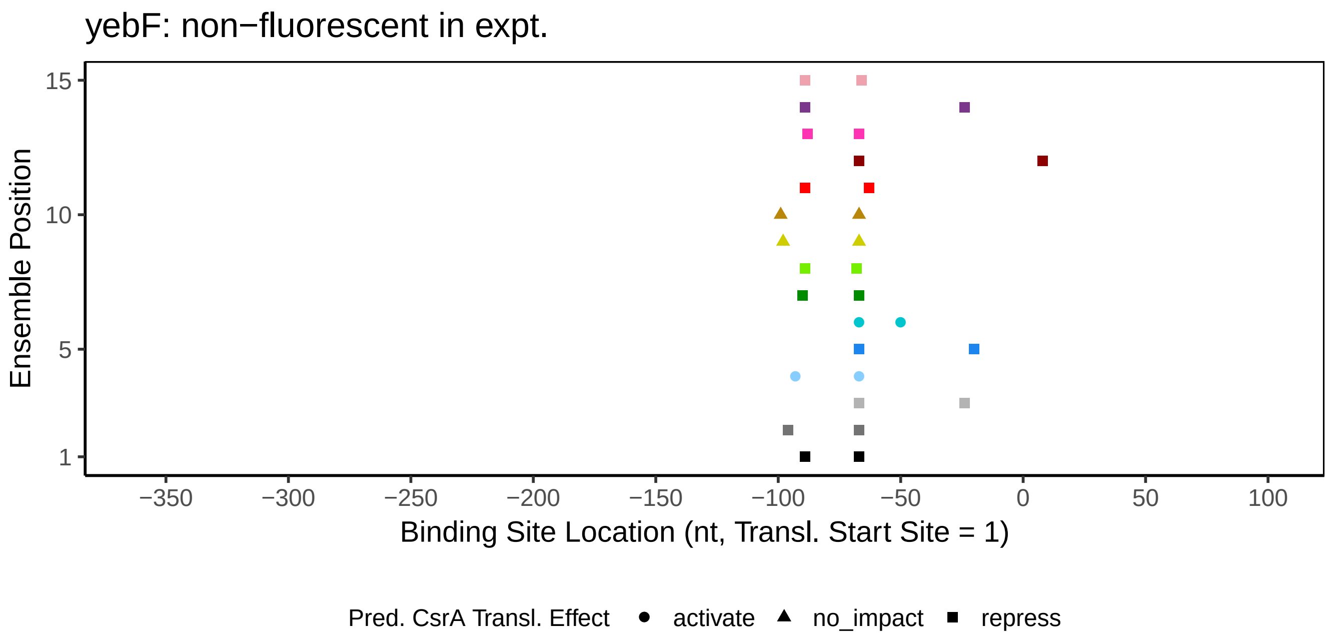

yebF: non−fluorescent in expt.
Ensemble Position
15
10
5
1
−350
−300
−250
−200
−150
−100
−50
0
50
100
Binding Site Location (nt, Transl. Start Site = 1)
Pred. CsrA Transl. Effect
activate
no_impact
repress

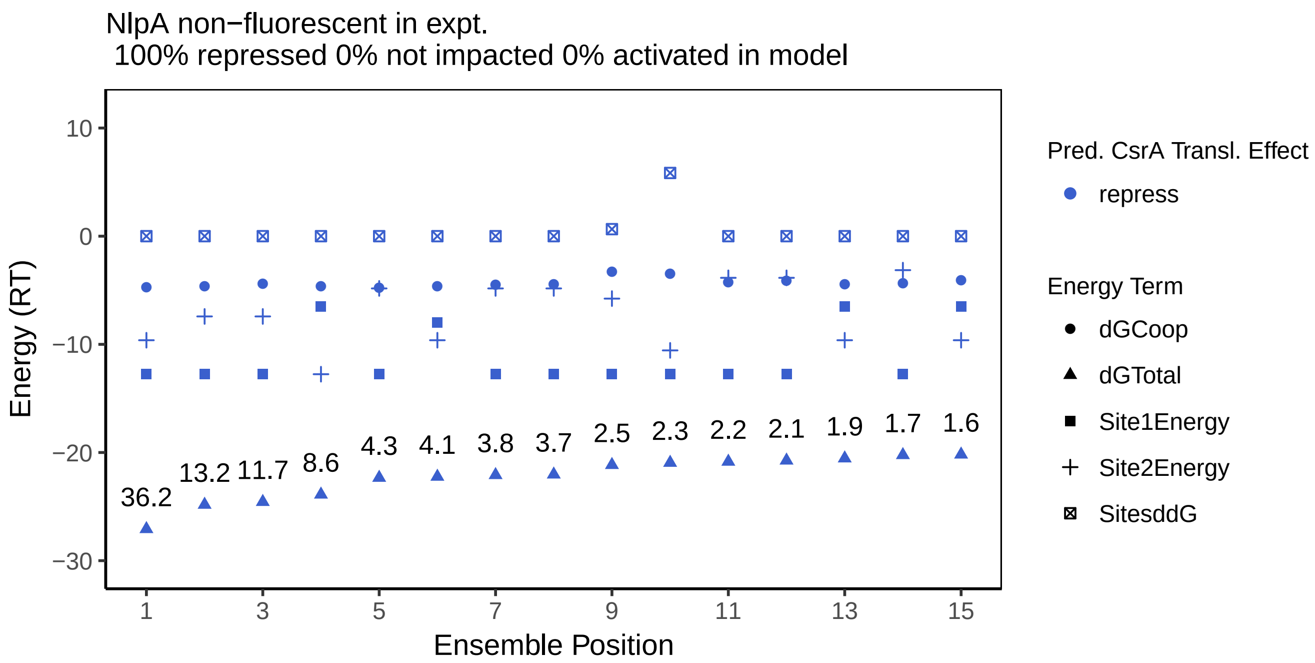

NlpA non−fluorescent in expt.
100% repressed 0% not impacted 0% activated in model
Energy (RT)
Ensemble Position
36.2
13.2
11.7
8.6
4.3
4.1
3.8
3.7
2.5
2.3
2.2
2.1
1.9
1.7
1.6
Pred. CsrA Transl. Effect
repress
Energy Term
dGCoop
dGTotal
Site1Energy
Site2Energy
SitesddG

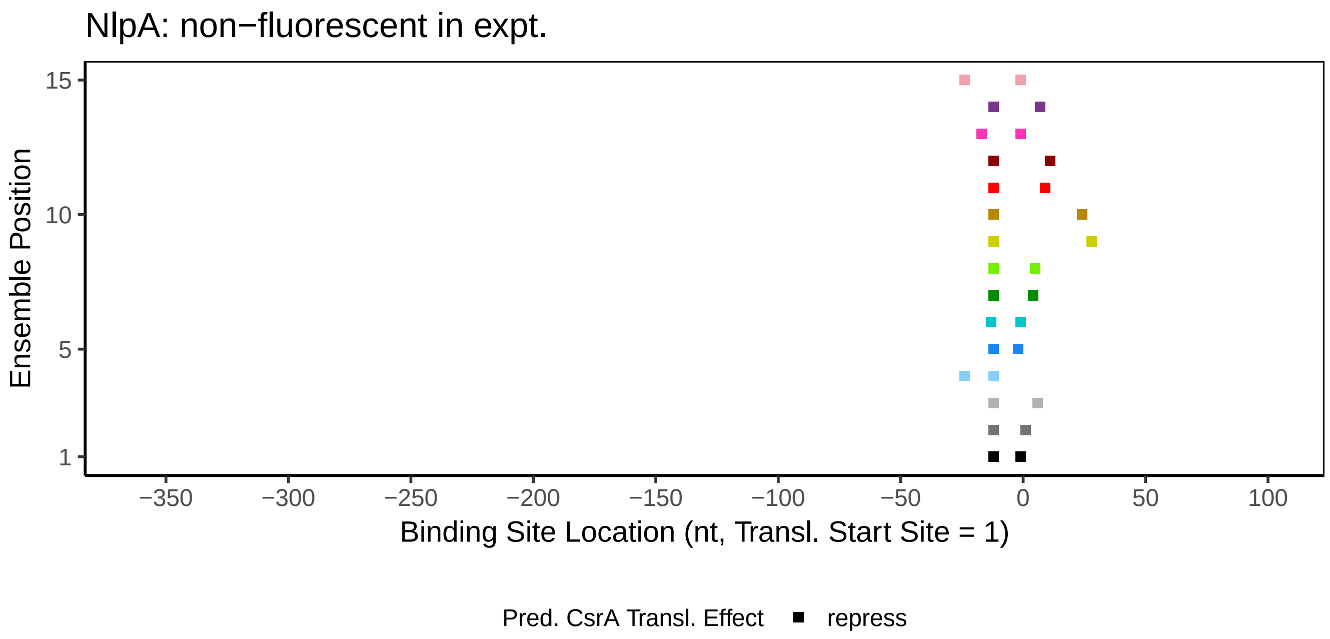

NlpA: non−fluorescent in expt.
Ensemble Position
Binding Site Location (nt, Transl. Start Site = 1)
Pred. CsrA Transl. Effect
repress

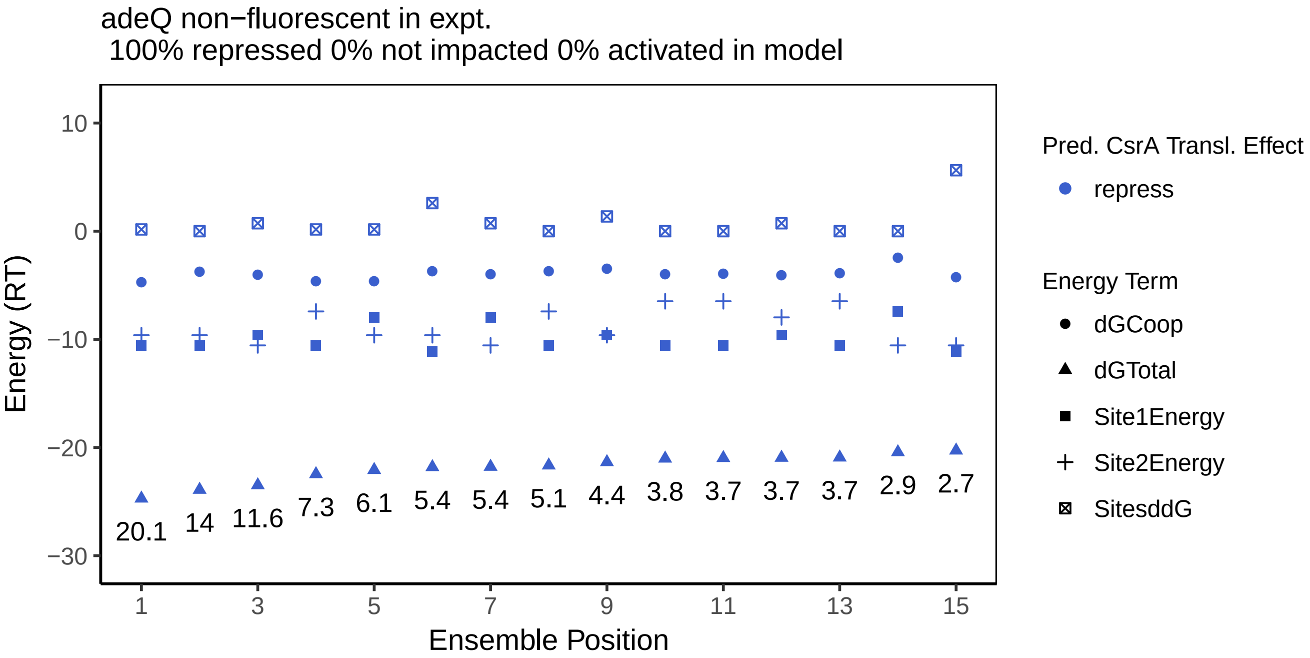

adeQ non−fluorescent in expt.
100% repressed 0% not impacted 0% activated in model
Energy (RT)
10
0
−10
−20
−30
20.1
14
11.6
7.3
6.1
5.4
5.4
5.1
4.4
3.8
3.7
3.7
3.7
2.9
2.7
1
3
5
7
9
11
13
15
Ensemble Position
Pred. CsrA Transl. Effect
repress
Energy Term
dGCoop
dGTotal
Site1Energy
Site2Energy
SitesddG

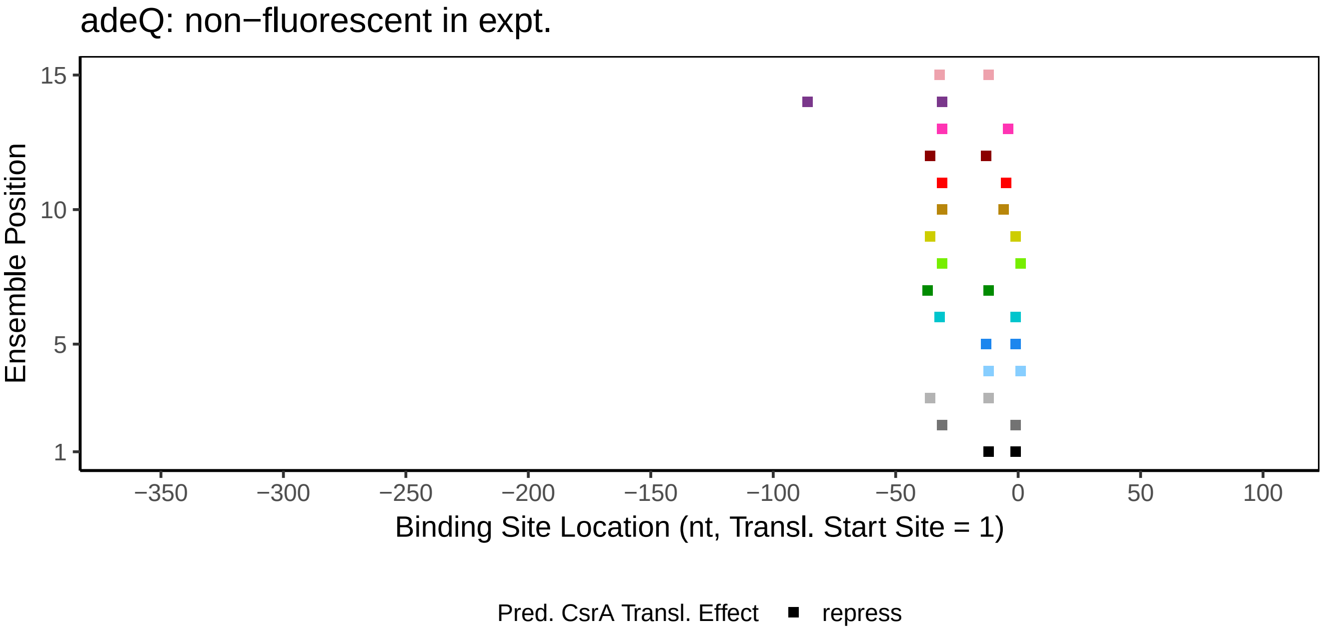

adeQ: non−fluorescent in expt.
Ensemble Position
15
10
5
1
−350
−300
−250
−200
−150
−100
−50
0
50
100
Binding Site Location (nt, Transl. Start Site = 1)
Pred. CsrA Transl. Effect
repress

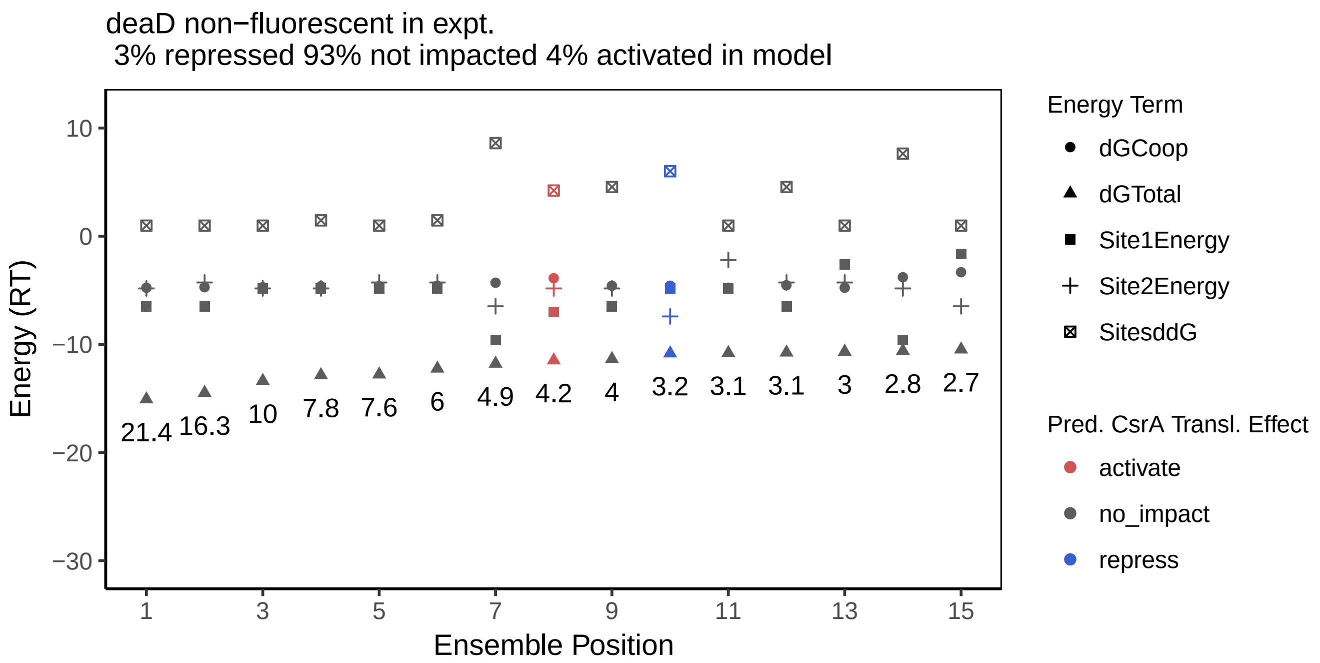
deaD non−fluorescent in expt.
3% repressed 93% not impacted 4% activated in model
Energy (RT)
Ensemble Position
21.4 16.3 10 7.8 7.6 6 4.9 4.2 4 3.2 3.1 3.1 3 2.8 2.7
Energy Term
dGCoop
dGTotal
Site1Energy
Site2Energy
SitesddG
Pred. CsrA Transl. Effect
activate
no_impact
repress

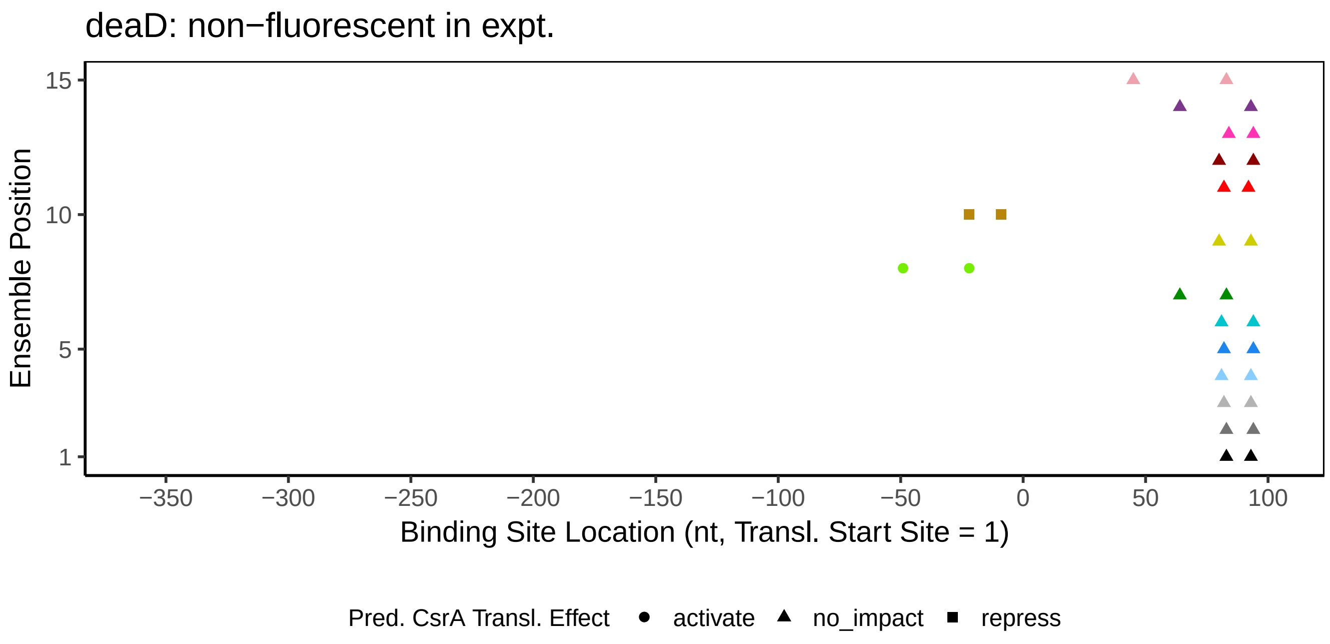
deaD: non−fluorescent in expt.
Ensemble Position
Binding Site Location (nt, Transl. Start Site = 1)
Pred. CsrA Transl. Effect
activate
no_impact
repress

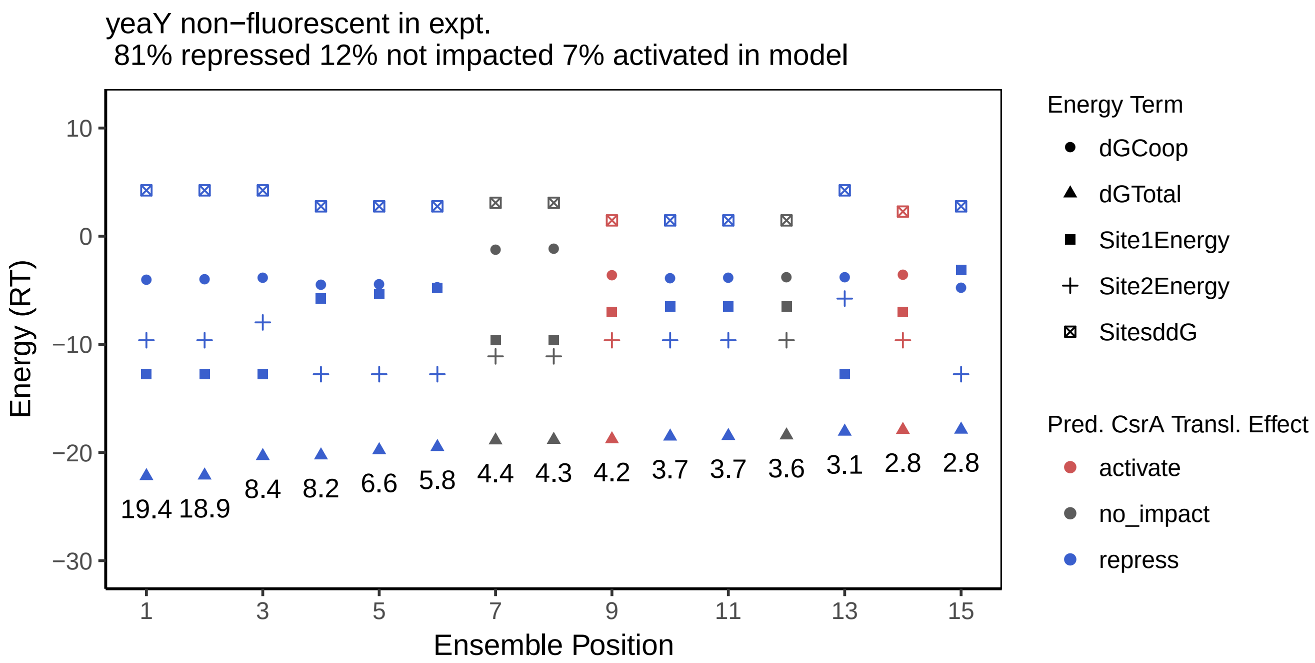
yeaY non−fluorescent in expt.
81% repressed 12% not impacted 7% activated in model
Energy (RT)
10
0
−10
−20
−30
19.4 18.9 8.4 8.2 6.6 5.8 4.4 4.3 4.2 3.7 3.7 3.6 3.1 2.8 2.8
1 3 5 7 9 11 13 15
Ensemble Position
Energy Term
dGCoop
dGTotal
Site1Energy
Site2Energy
SitesddG
Pred. CsrA Transl. Effect
activate
no_impact
repress

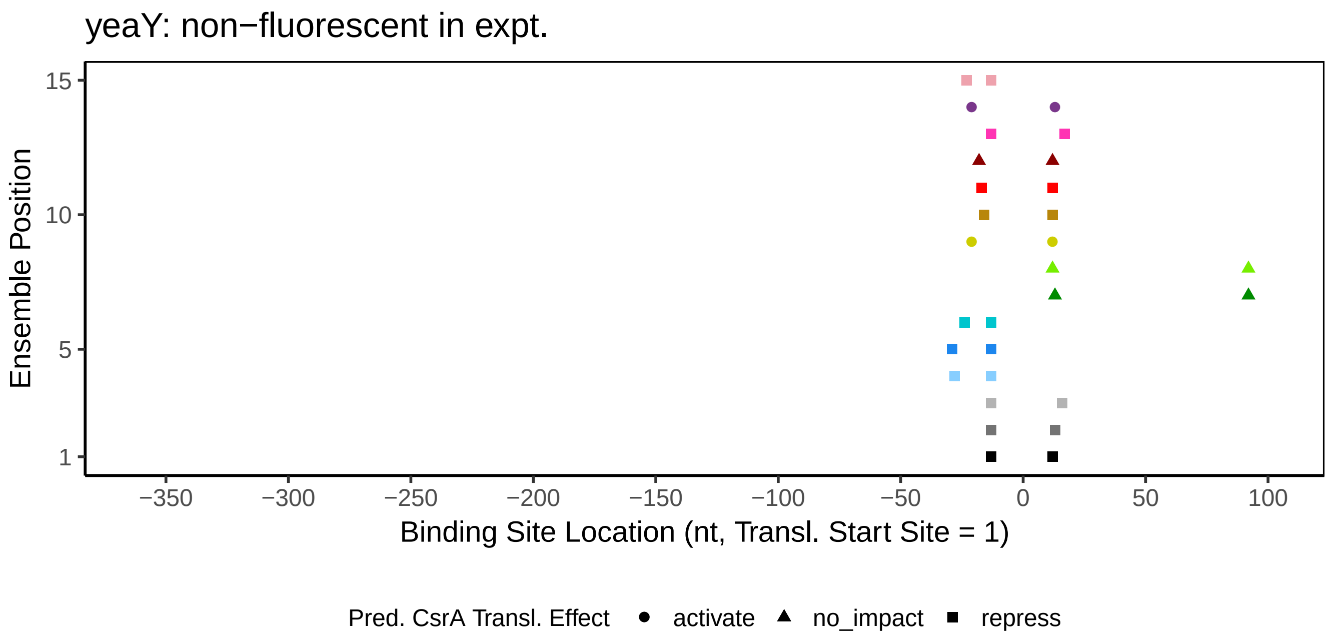
yeaY: non−fluorescent in expt.
Ensemble Position
15
10
5
1
−350 −300 −250 −200 −150 −100 −50 0 50 100
Binding Site Location (nt, Transl. Start Site = 1)
Pred. CsrA Transl. Effect
activate
no_impact
repress

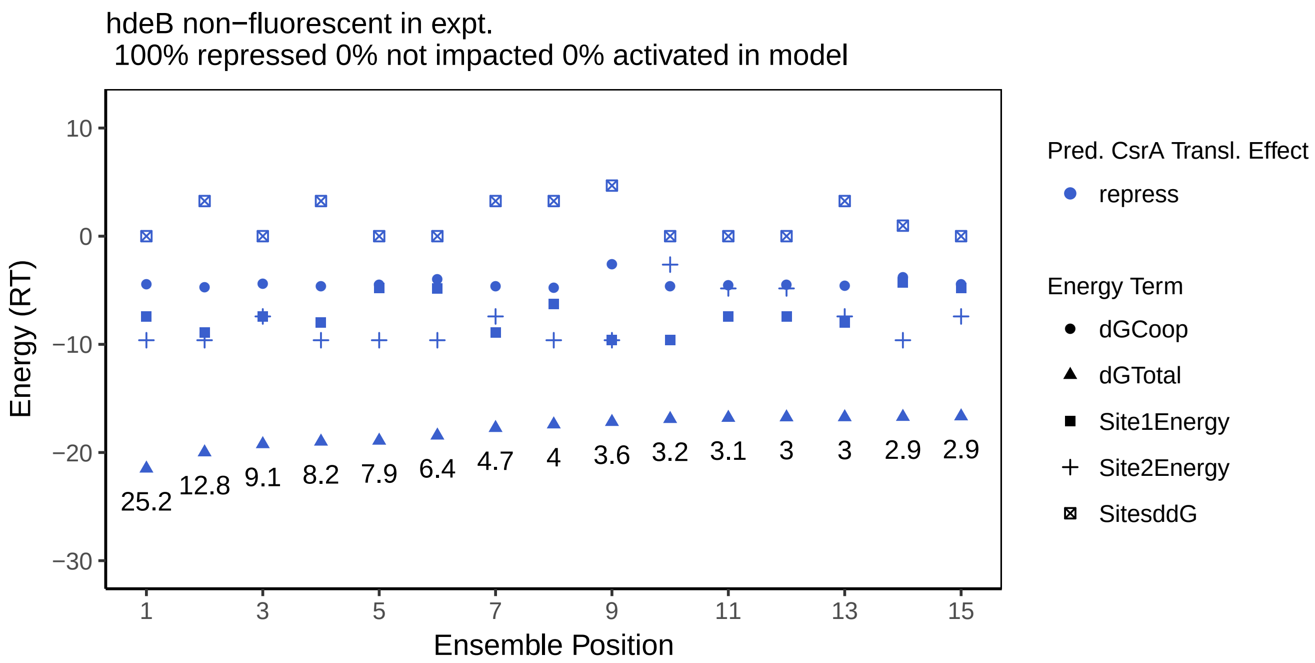
hdeB non−fluorescent in expt.
100% repressed 0% not impacted 0% activated in model
Energy (RT)
10
0
−10
−20
−30
25.2
12.8
9.1
8.2
7.9
6.4
4.7
4
3.6
3.2
3.1
3
3
2.9
2.9
1
3
5
7
9
11
13
15
Ensemble Position
Pred. CsrA Transl. Effect
repress
Energy Term
dGCoop
dGTotal
Site1Energy
Site2Energy
SitesddG

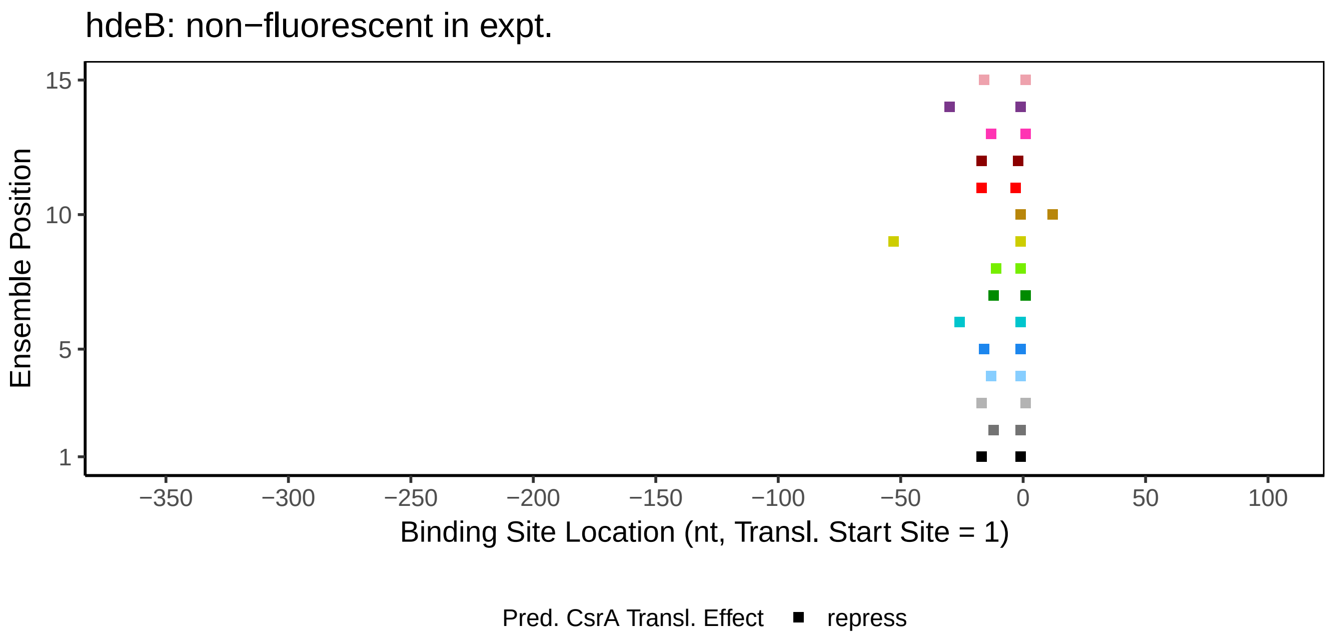
hdeB: non−fluorescent in expt.
Ensemble Position
15
10
5
1
−350
−300
−250
−200
−150
−100
−50
0
50
100
Binding Site Location (nt, Transl. Start Site = 1)
Pred. CsrA Transl. Effect
repress

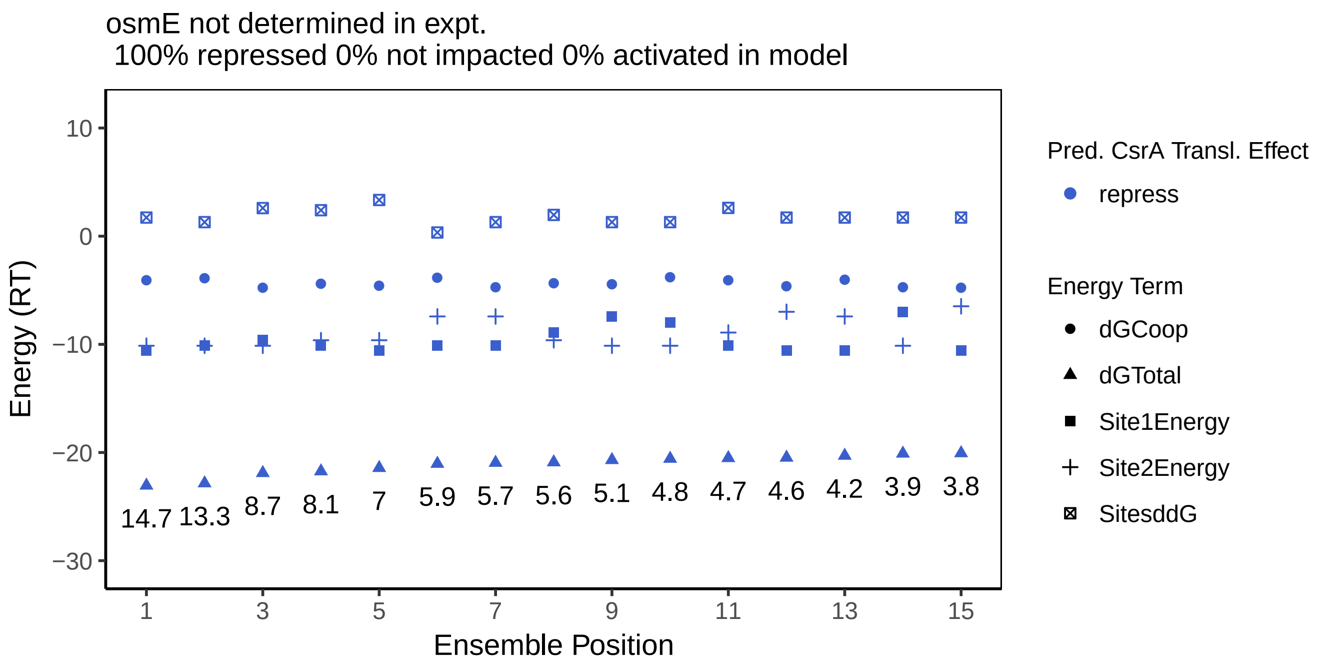

osmE not determined in expt.
100% repressed 0% not impacted 0% activated in model
Energy (RT)
Ensemble Position
14.7 13.3 8.7 8.1 7 5.9 5.7 5.6 5.1 4.8 4.7 4.6 4.2 3.9 3.8
Pred. CsrA Transl. Effect
repress
Energy Term
dGCoop
dGTotal
Site1Energy
Site2Energy
SitesddG

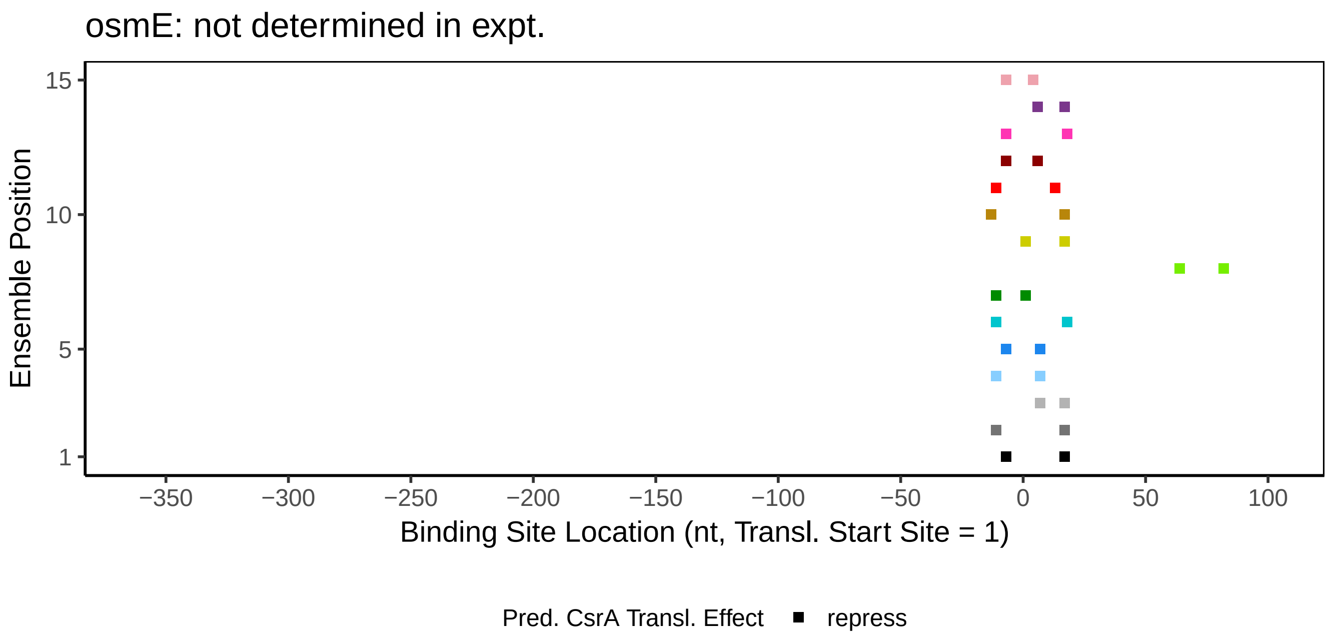

osmE: not determined in expt.
Ensemble Position
Binding Site Location (nt, Transl. Start Site = 1)
Pred. CsrA Transl. Effect
repress

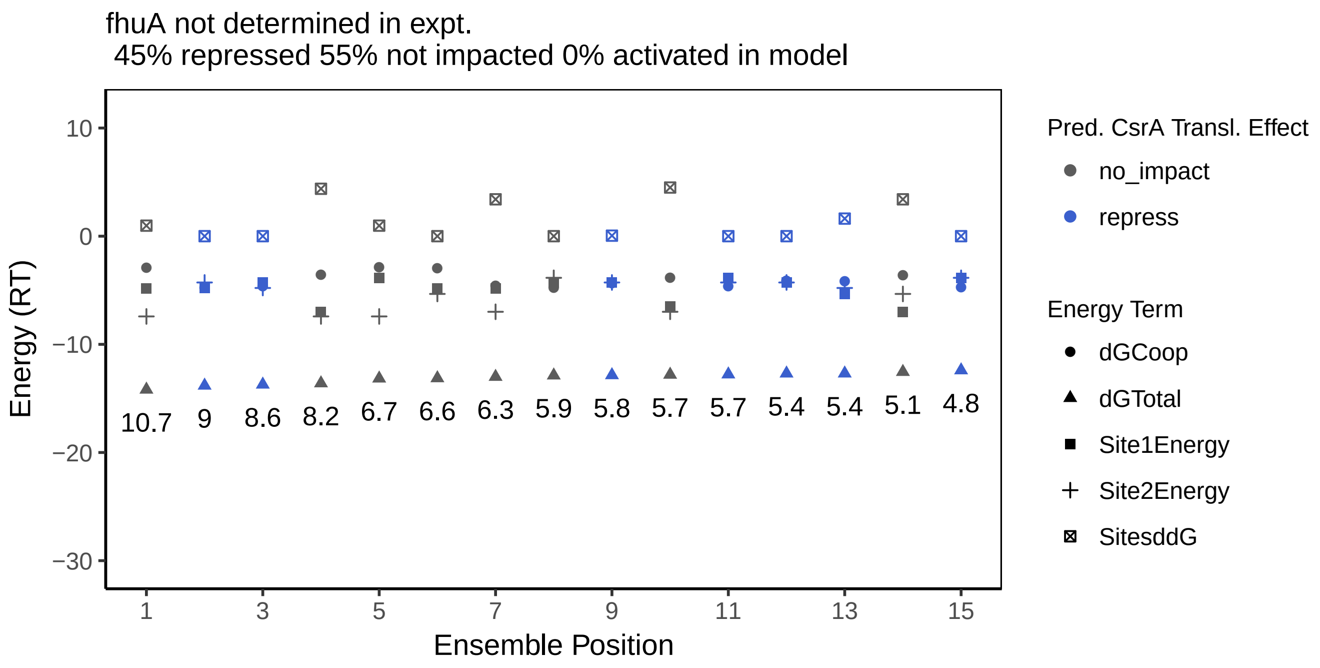
fhuA not determined in expt.
45% repressed 55% not impacted 0% activated in model
Energy (RT)
10
0
−10
−20
−30
10.7
9
8.6
8.2
6.7
6.6
6.3
5.9
5.8
5.7
5.7
5.4
5.4
5.1
4.8
1
3
5
7
9
11
13
15
Ensemble Position
Pred. CsrA Transl. Effect
no_impact
repress
Energy Term
dGCoop
dGTotal
Site1Energy
Site2Energy
SitesddG

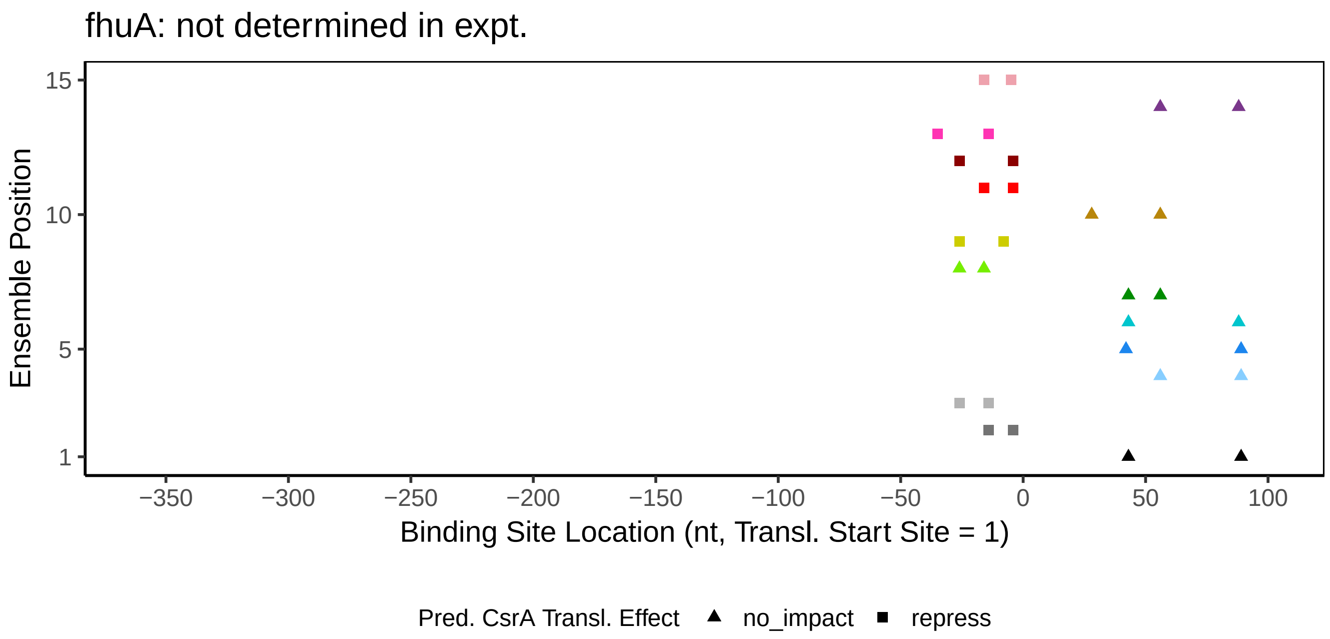
fhuA: not determined in expt.
Ensemble Position
15
10
5
1
−350
−300
−250
−200
−150
−100
−50
0
50
100
Binding Site Location (nt, Transl. Start Site = 1)
Pred. CsrA Transl. Effect
no_impact
repress

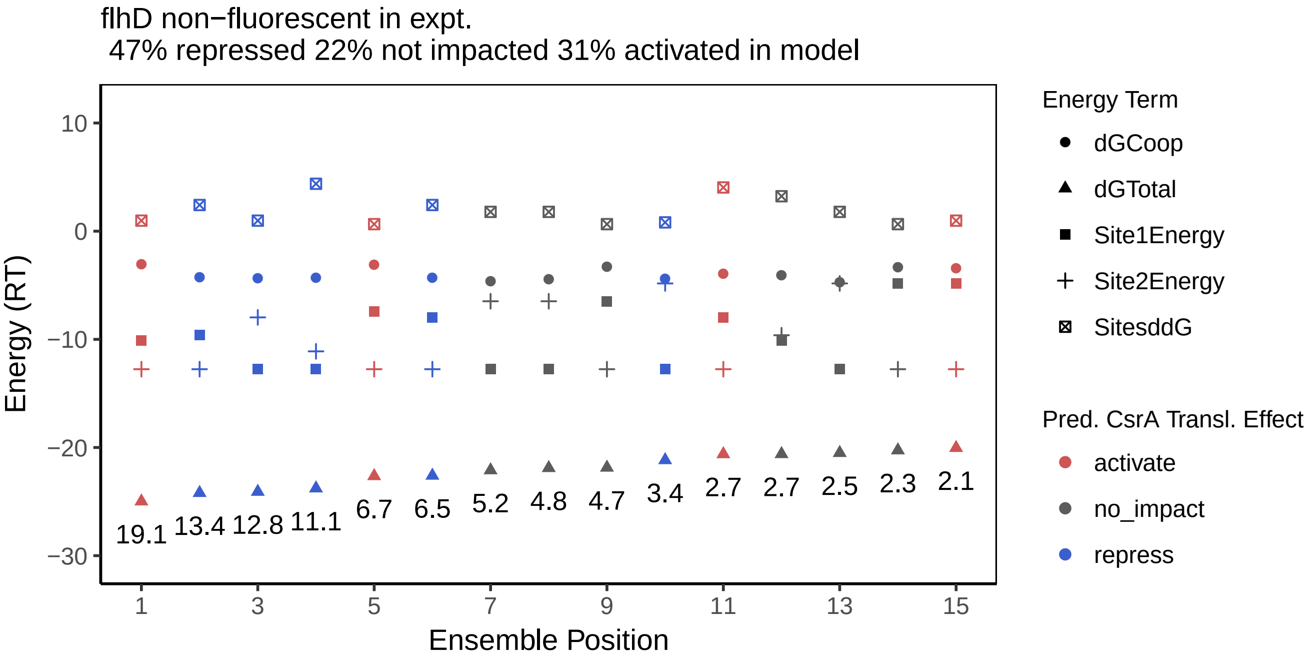

flhD non−fluorescent in expt.
47% repressed 22% not impacted 31% activated in model
Energy (RT)
Ensemble Position
19.1 13.4 12.8 11.1 6.7 6.5 5.2 4.8 4.7 3.4 2.7 2.7 2.5 2.3 2.1
Energy Term
dGCoop
dGTotal
Site1Energy
Site2Energy
SitesddG
Pred. CsrA Transl. Effect
activate
no_impact
repress

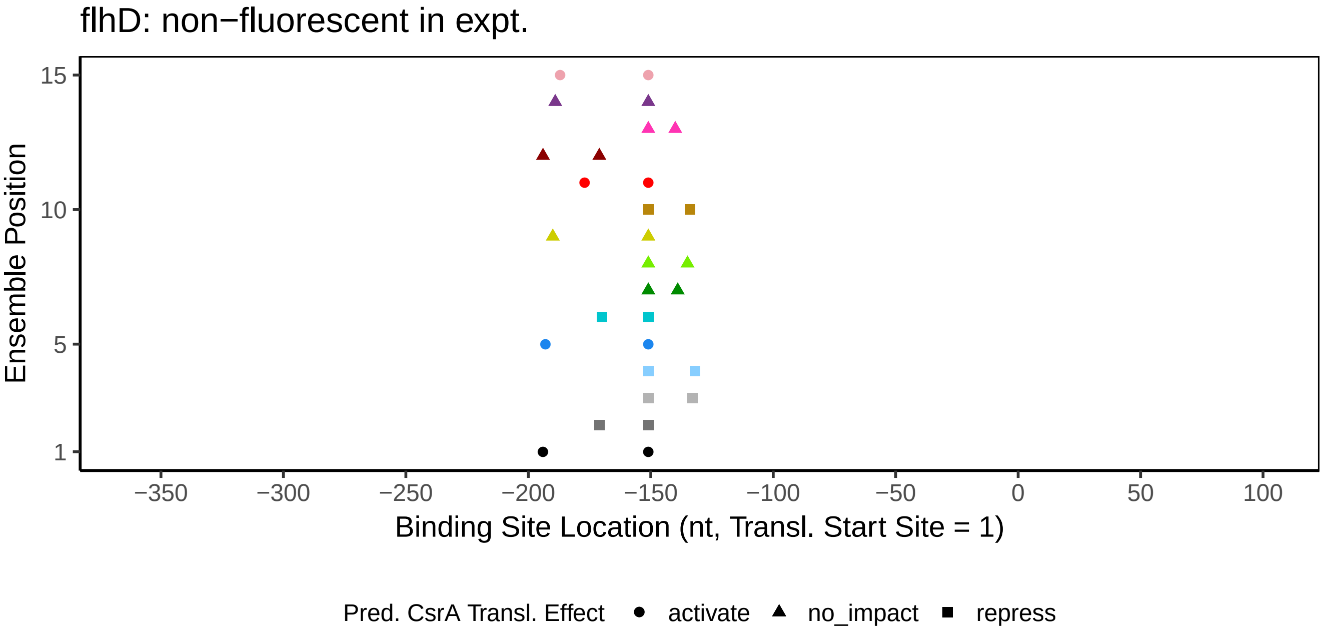

flhD: non−fluorescent in expt.
Ensemble Position
Binding Site Location (nt, Transl. Start Site = 1)
Pred. CsrA Transl. Effect
activate
no_impact
repress

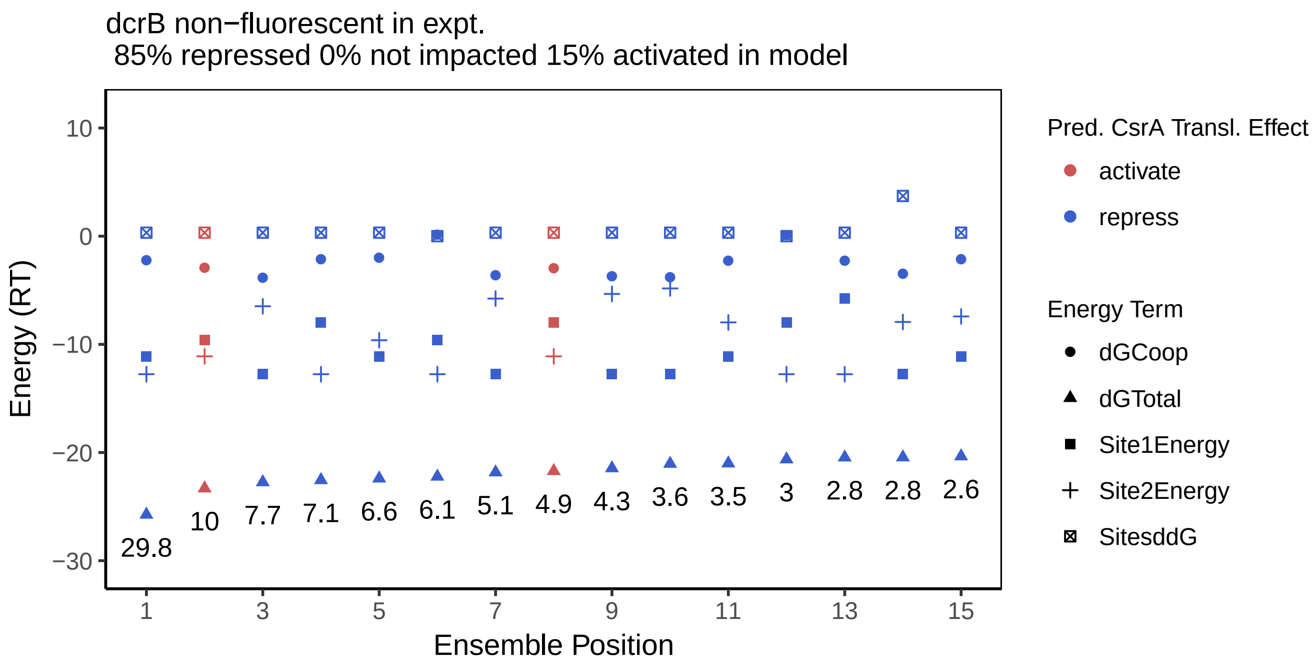
dcrB non−fluorescent in expt.
85% repressed 0% not impacted 15% activated in model
Energy (RT)
10
0
−10
−20
−30
29.8
10
7.7
7.1
6.6
6.1
5.1
4.9
4.3
3.6
3.5
3
2.8
2.8
2.6
1
3
5
7
9
11
13
15
Ensemble Position
Pred. CsrA Transl. Effect
activate
repress
Energy Term
dGCoop
dGTotal
Site1Energy
Site2Energy
SitesddG

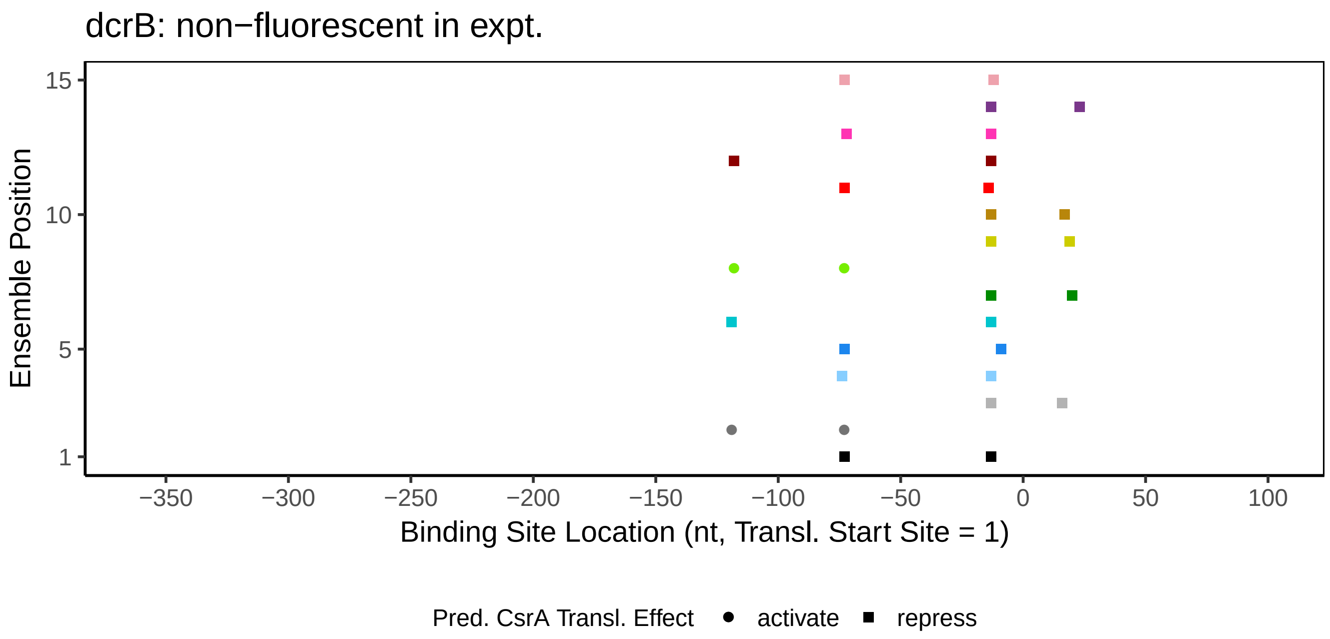
dcrB: non−fluorescent in expt.
Ensemble Position
15
10
5
1
−350
−300
−250
−200
−150
−100
−50
0
50
100
Binding Site Location (nt, Transl. Start Site = 1)
Pred. CsrA Transl. Effect
activate
repress

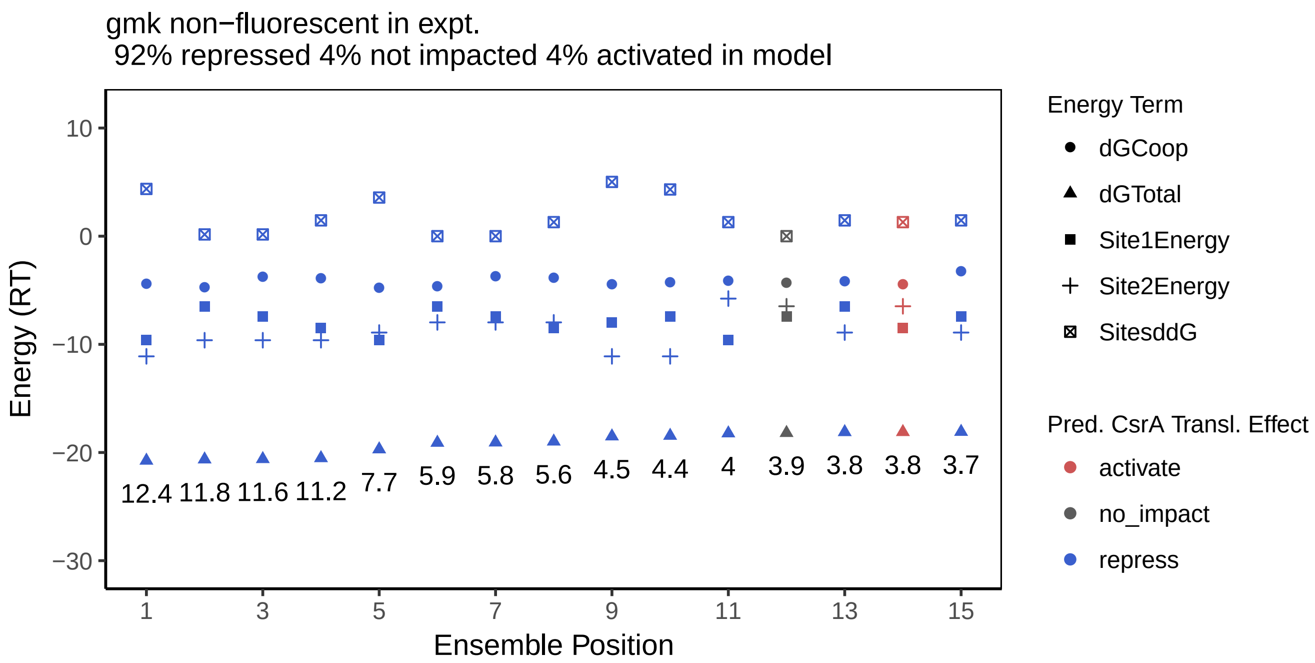
gmk non−fluorescent in expt.
92% repressed 4% not impacted 4% activated in model
Energy (RT)
10
0
−10
−20
−30
12.4 11.8 11.6 11.2 7.7 5.9 5.8 5.6 4.5 4.4 4 3.9 3.8 3.8 3.7
1 3 5 7 9 11 13 15
Ensemble Position
Energy Term
dGCoop
dGTotal
Site1Energy
Site2Energy
SitesddG
Pred. CsrA Transl. Effect
activate
no_impact
repress

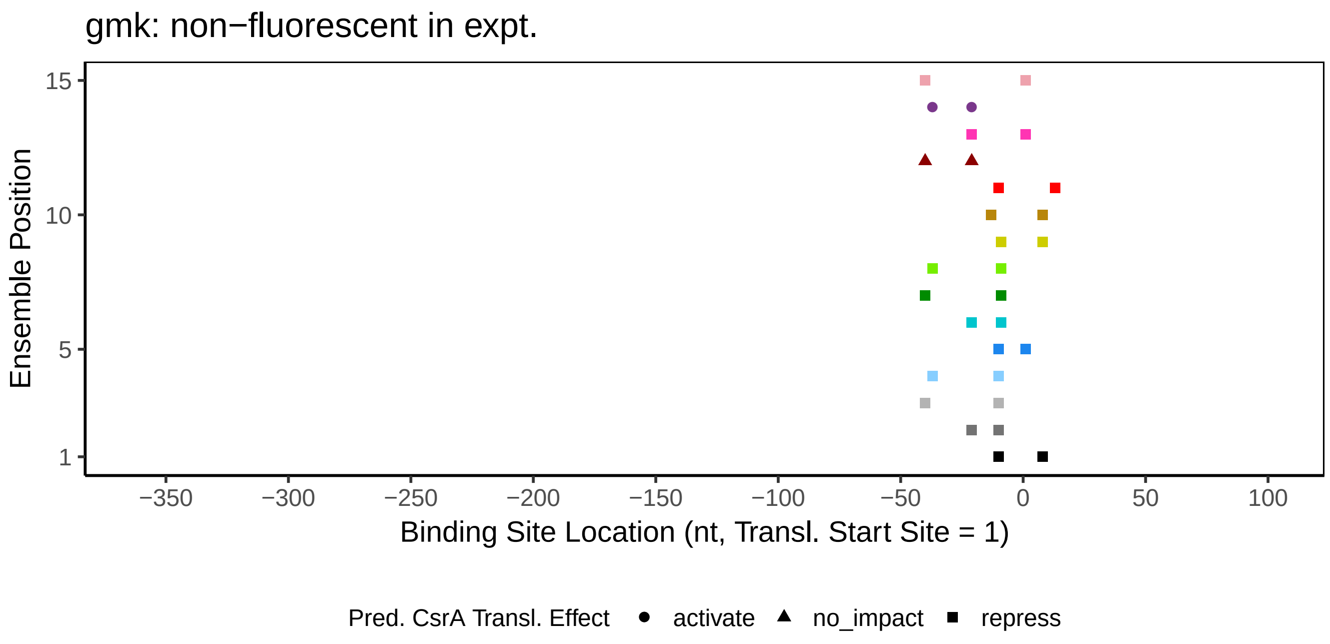
gmk: non−fluorescent in expt.
Ensemble Position
15
10
5
1
−350 −300 −250 −200 −150 −100 −50 0 50 100
Binding Site Location (nt, Transl. Start Site = 1)
Pred. CsrA Transl. Effect
activate
no_impact
repress

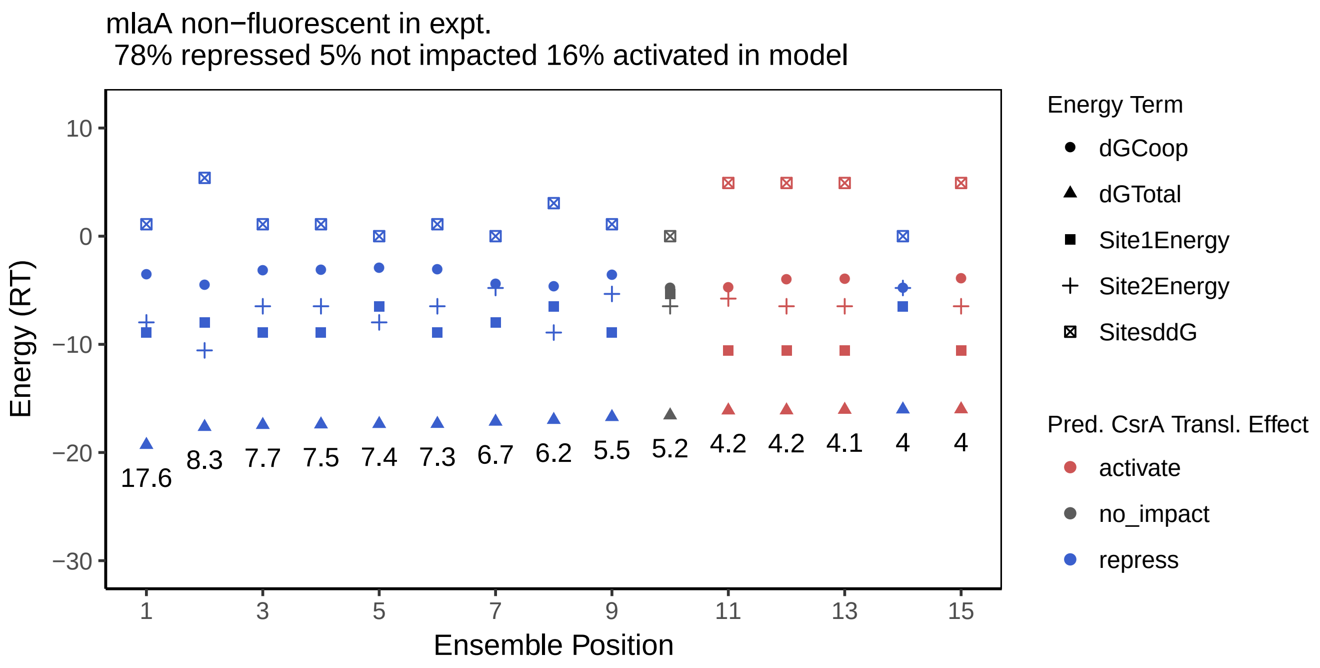

mlaA non−fluorescent in expt.
78% repressed 5% not impacted 16% activated in model
Energy (RT)
Ensemble Position
17.6
8.3
7.7
7.5
7.4
7.3
6.7
6.2
5.5
5.2
4.2
4.2
4.1
4
4
Energy Term
dGCoop
dGTotal
Site1Energy
Site2Energy
SitesddG
Pred. CsrA Transl. Effect
activate
no_impact
repress

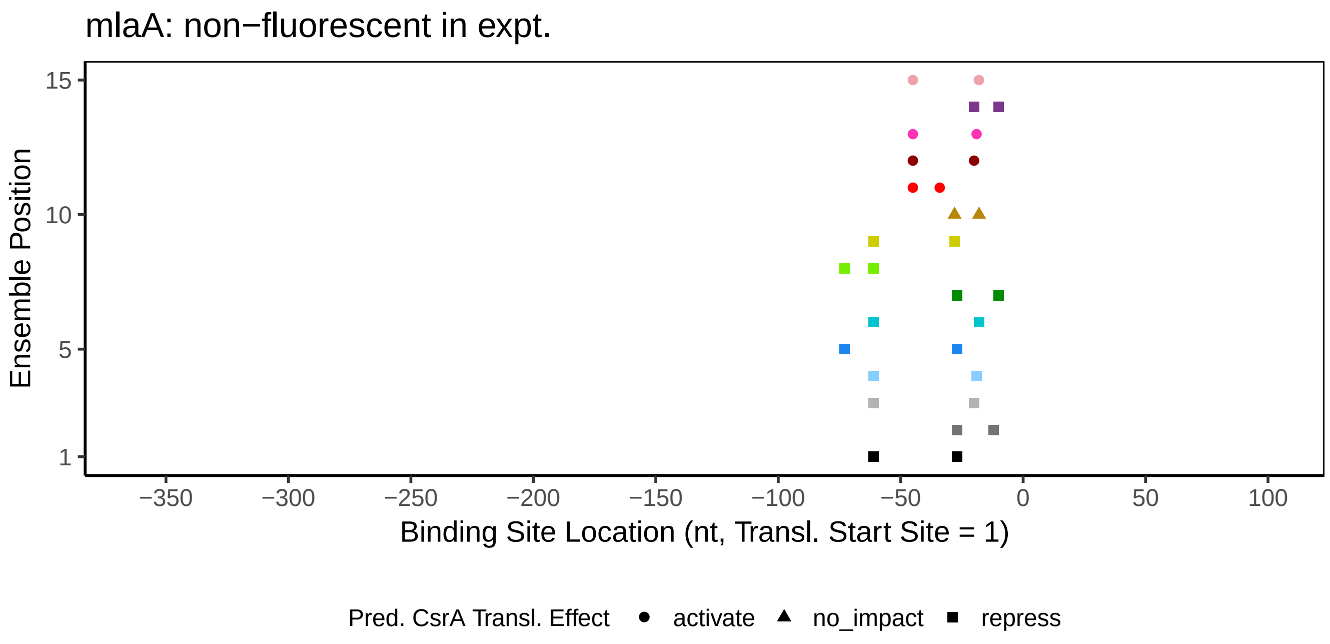

mlaA: non−fluorescent in expt.
Ensemble Position
Binding Site Location (nt, Transl. Start Site = 1)
Pred. CsrA Transl. Effect
activate
no_impact
repress

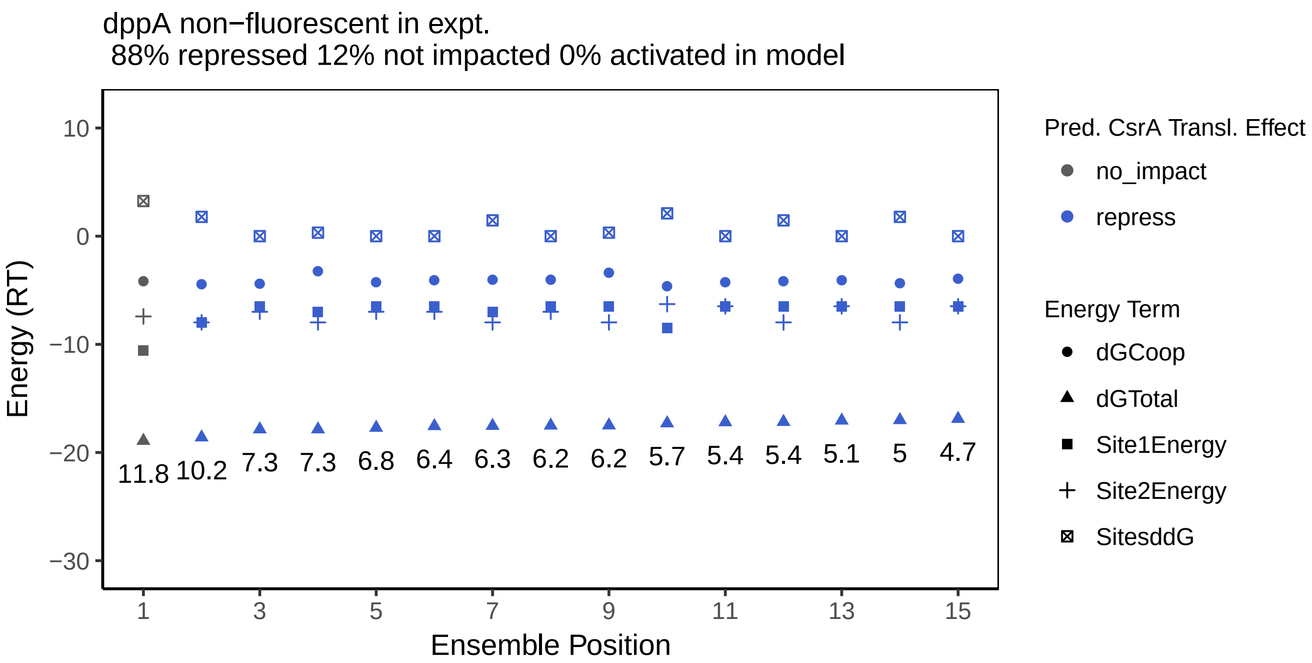
dppA non−fluorescent in expt.
88% repressed 12% not impacted 0% activated in model
Energy (RT)
10
0
−10
−20
−30
11.8 10.2 7.3 7.3 6.8 6.4 6.3 6.2 6.2 5.7 5.4 5.4 5.1 5 4.7
1 3 5 7 9 11 13 15
Ensemble Position
Pred. CsrA Transl. Effect
no_impact
repress
Energy Term
dGCoop
dGTotal
Site1Energy
Site2Energy
SitesddG

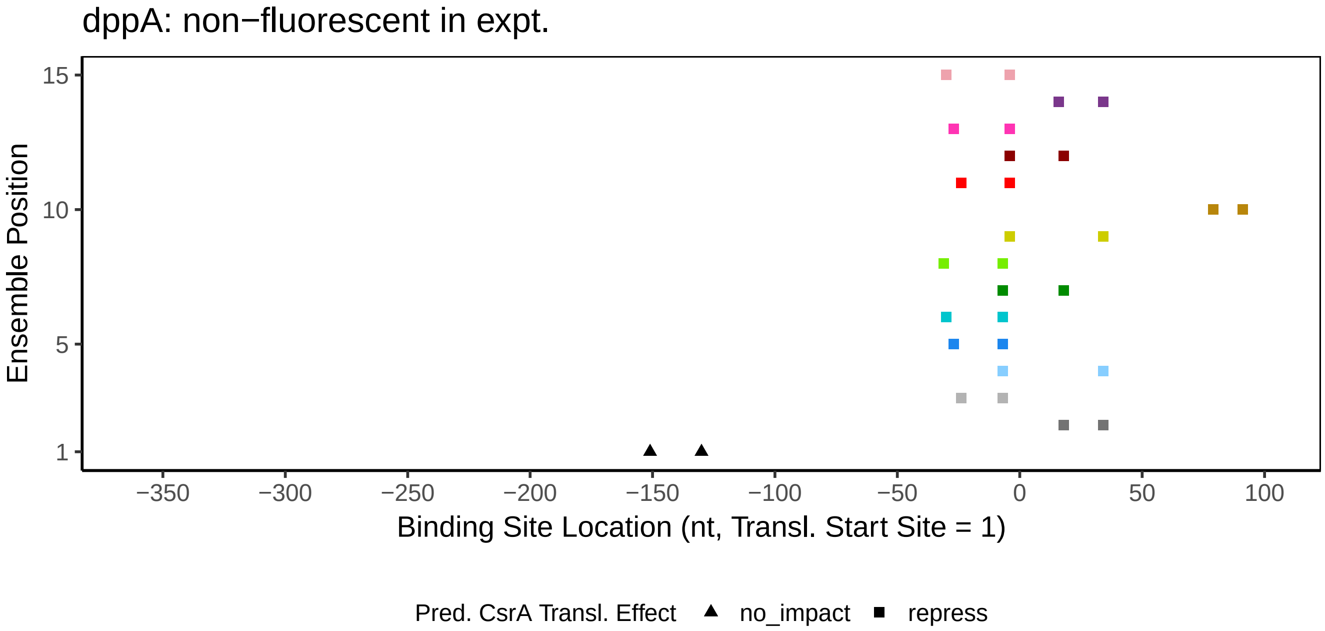
dppA: non−fluorescent in expt.
Ensemble Position
15
10
5
1
−350 −300 −250 −200 −150 −100 −50 0 50 100
Binding Site Location (nt, Transl. Start Site = 1)
Pred. CsrA Transl. Effect
no_impact
repress

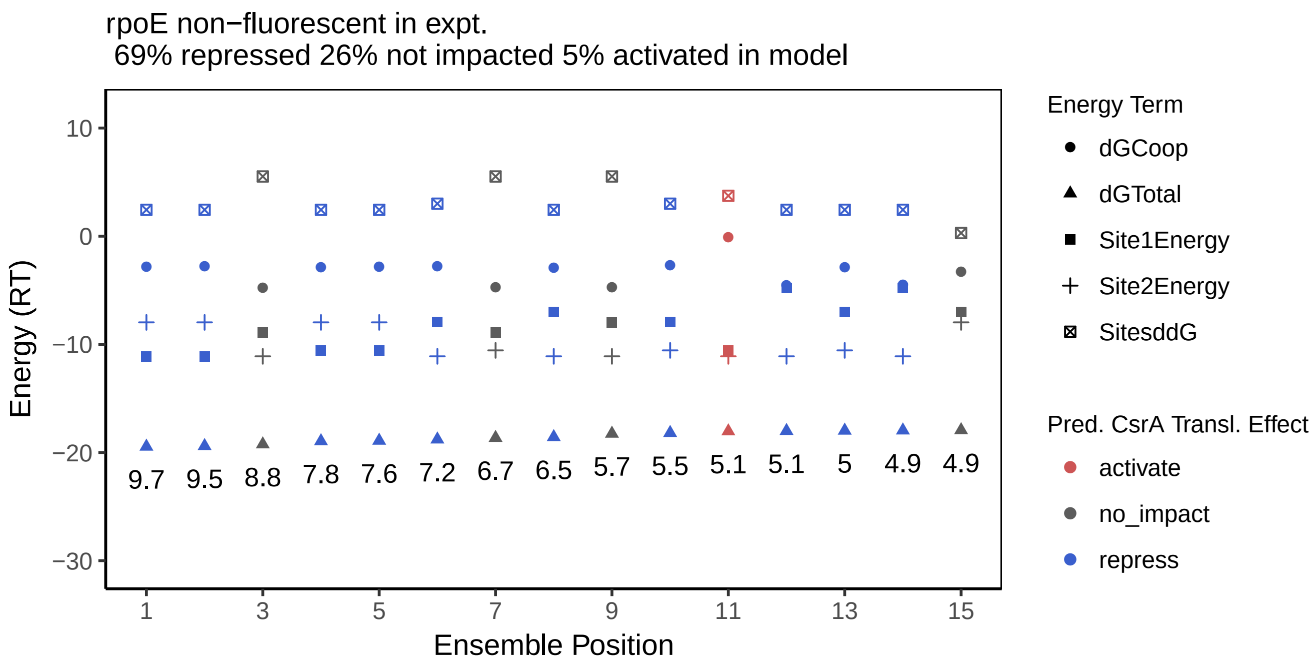
rpoE non−fluorescent in expt.
69% repressed 26% not impacted 5% activated in model
Energy (RT)
10
0
−10
−20
−30
9.7 9.5 8.8 7.8 7.6 7.2 6.7 6.5 5.7 5.5 5.1 5.1 5 4.9 4.9
1 3 5 7 9 11 13 15
Ensemble Position
Energy Term
dGCoop
dGTotal
Site1Energy
Site2Energy
SitesddG
Pred. CsrA Transl. Effect
activate
no_impact
repress

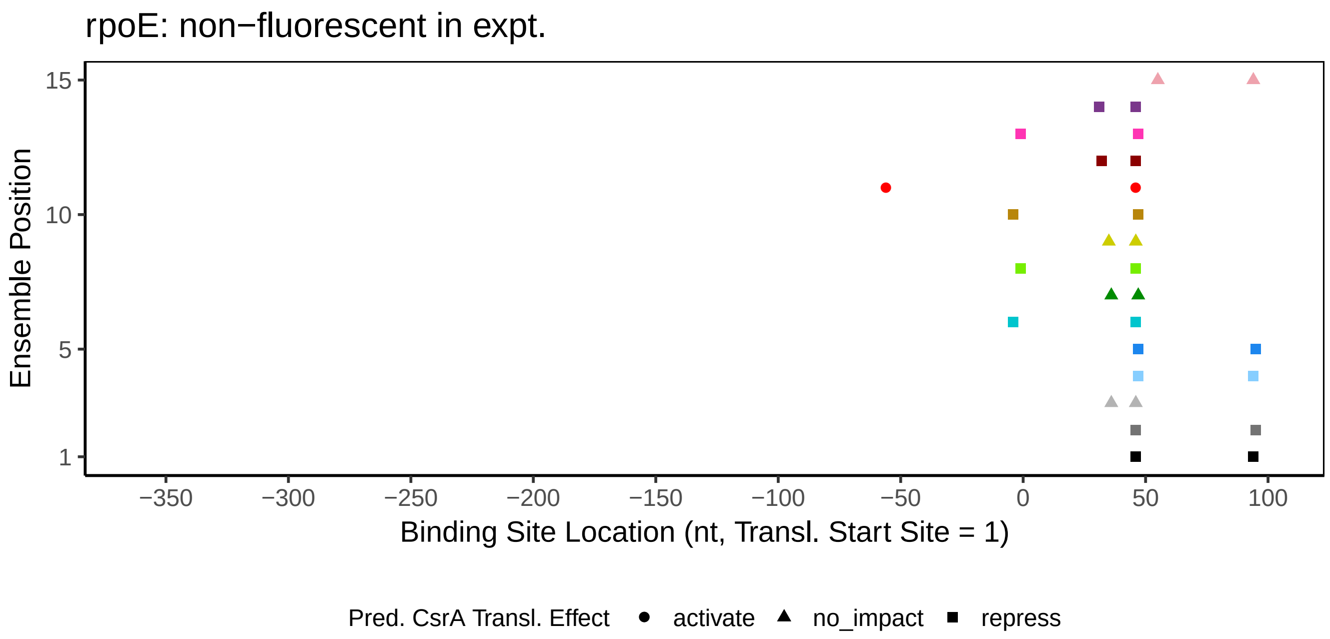
rpoE: non−fluorescent in expt.
Ensemble Position
15
10
5
1
−350 −300 −250 −200 −150 −100 −50 0 50 100
Binding Site Location (nt, Transl. Start Site = 1)
Pred. CsrA Transl. Effect
activate
no_impact
repress

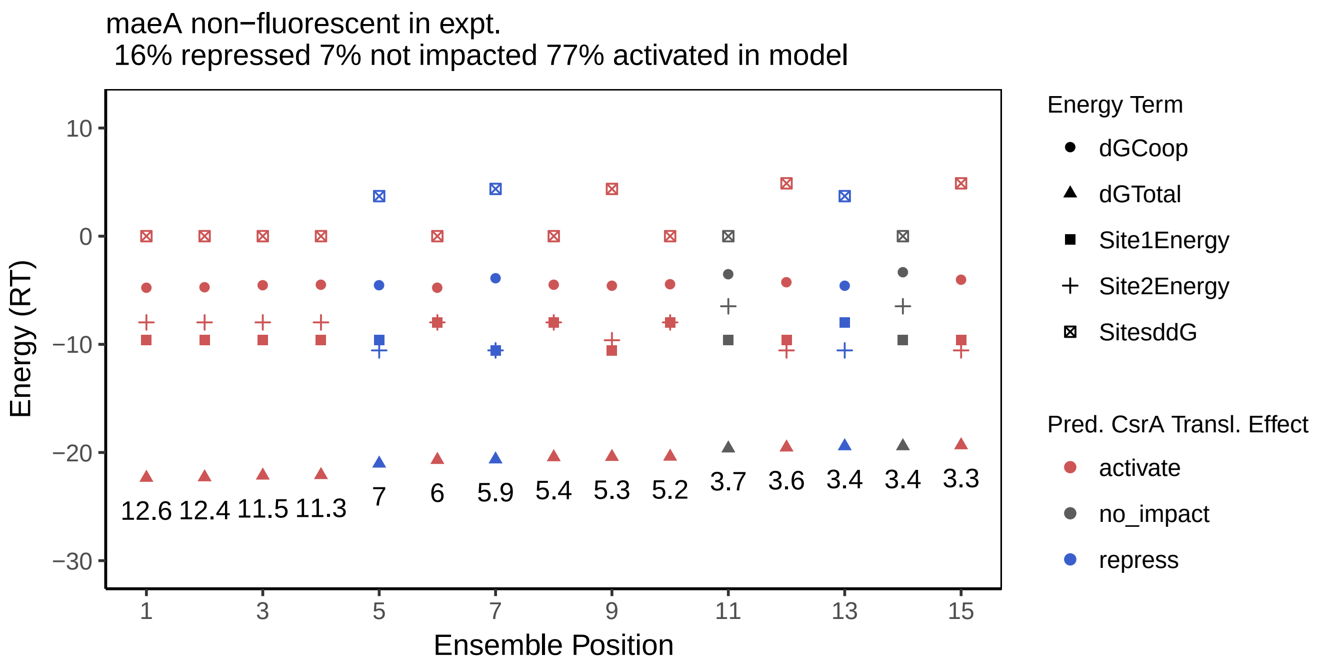
maeA non−fluorescent in expt.
16% repressed 7% not impacted 77% activated in model
Energy (RT)
Ensemble Position
12.6 12.4 11.5 11.3 7 6 5.9 5.4 5.3 5.2 3.7 3.6 3.4 3.4 3.3
Energy Term
dGCoop
dGTotal
Site1Energy
Site2Energy
SitesddG
Pred. CsrA Transl. Effect
activate
no_impact
repress

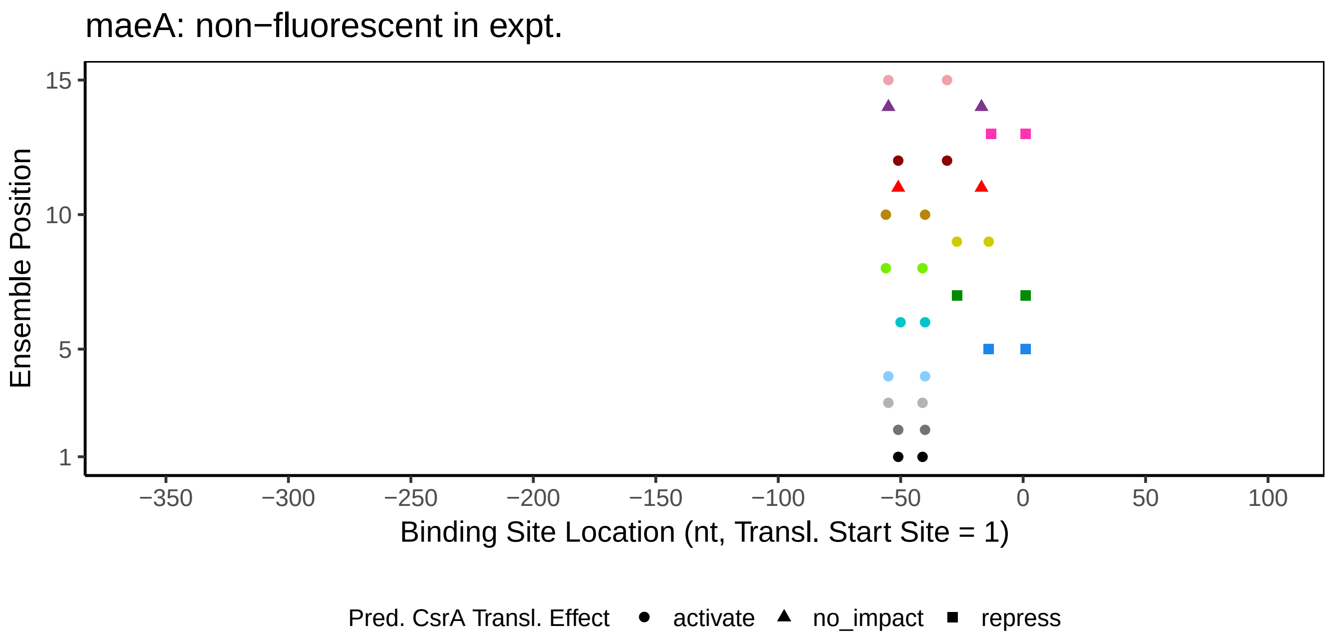
maeA: non−fluorescent in expt.
Ensemble Position
Binding Site Location (nt, Transl. Start Site = 1)
Pred. CsrA Transl. Effect
activate
no_impact
repress

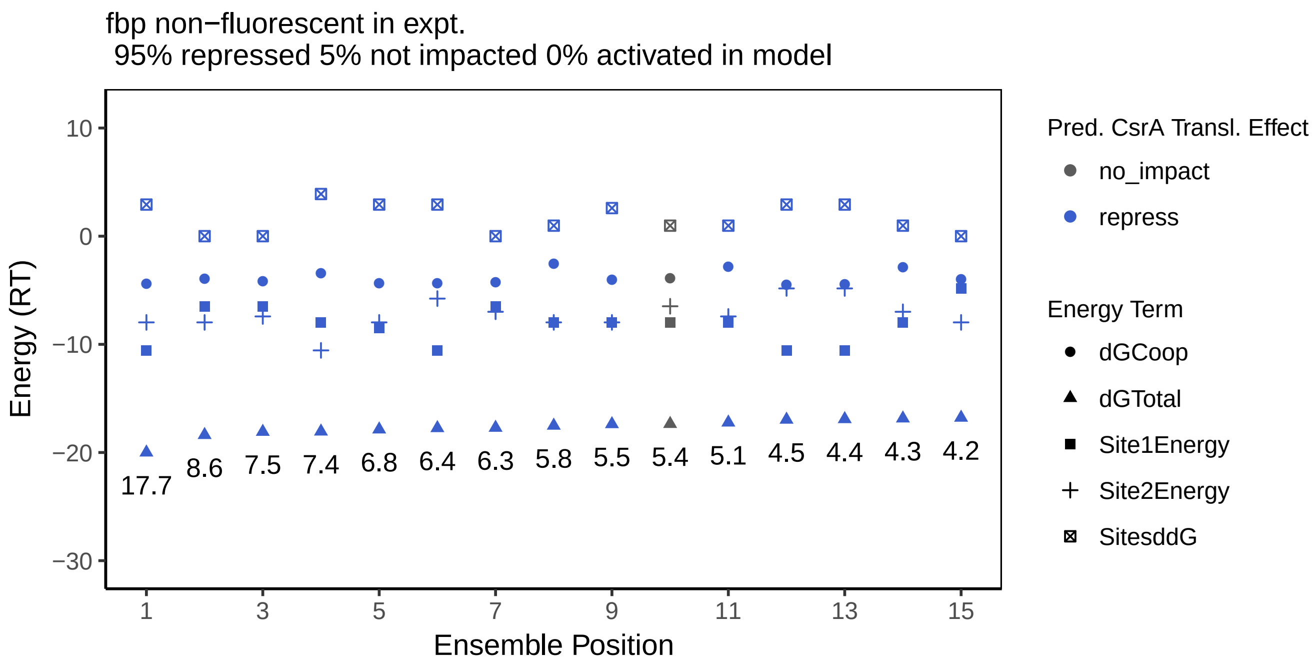
fbp non−fluorescent in expt.
95% repressed 5% not impacted 0% activated in model
Energy (RT)
Ensemble Position
17.7
8.6
7.5
7.4
6.8
6.4
6.3
5.8
5.5
5.4
5.1
4.5
4.4
4.3
4.2
Pred. CsrA Transl. Effect
no_impact
repress
Energy Term
dGCoop
dGTotal
Site1Energy
Site2Energy
SitesddG

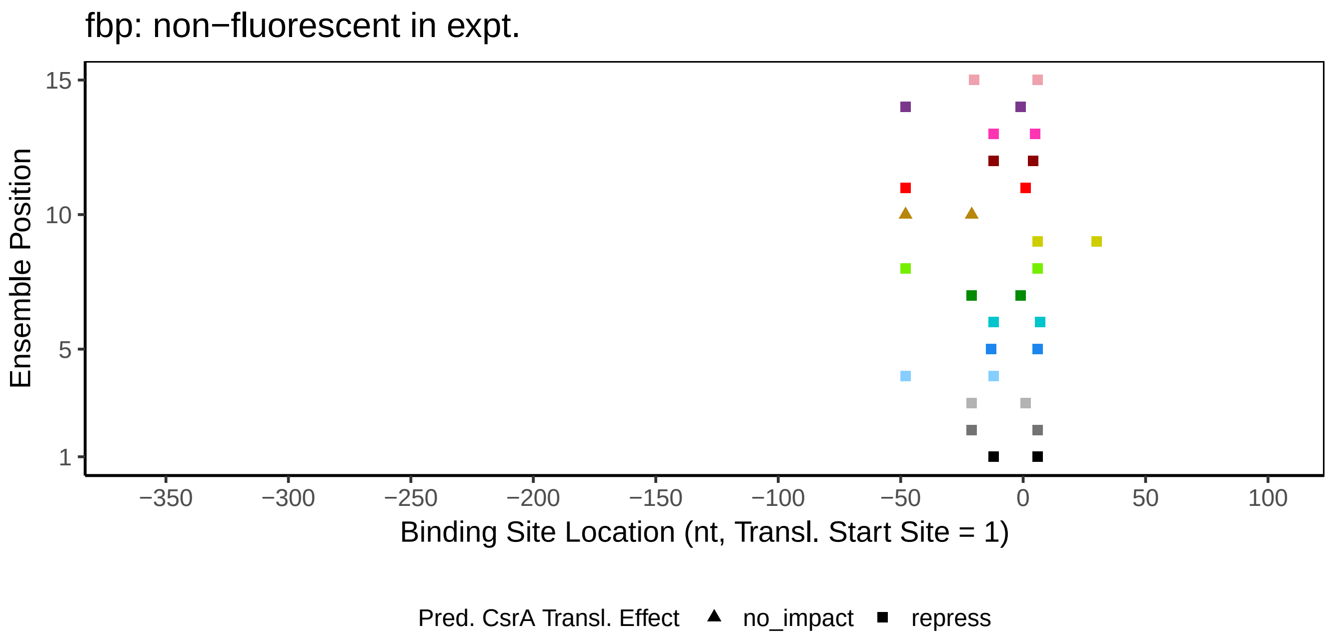
fbp: non−fluorescent in expt.
Ensemble Position
Binding Site Location (nt, Transl. Start Site = 1)
Pred. CsrA Transl. Effect
no_impact
repress

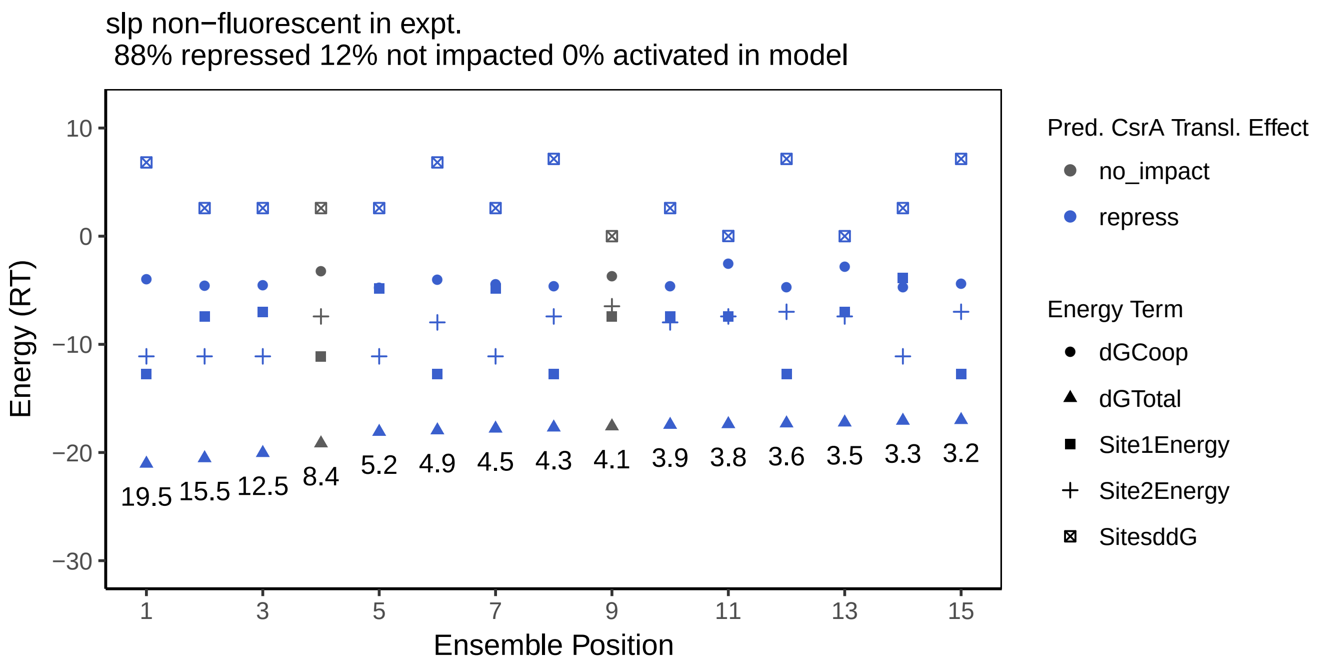
slp non−fluorescent in expt.
88% repressed 12% not impacted 0% activated in model
Energy (RT)
Ensemble Position
19.5
15.5
12.5
8.4
5.2
4.9
4.5
4.3
4.1
3.9
3.8
3.6
3.5
3.3
3.2
Pred. CsrA Transl. Effect
no_impact
repress
Energy Term
dGCoop
dGTotal
Site1Energy
Site2Energy
SitesddG

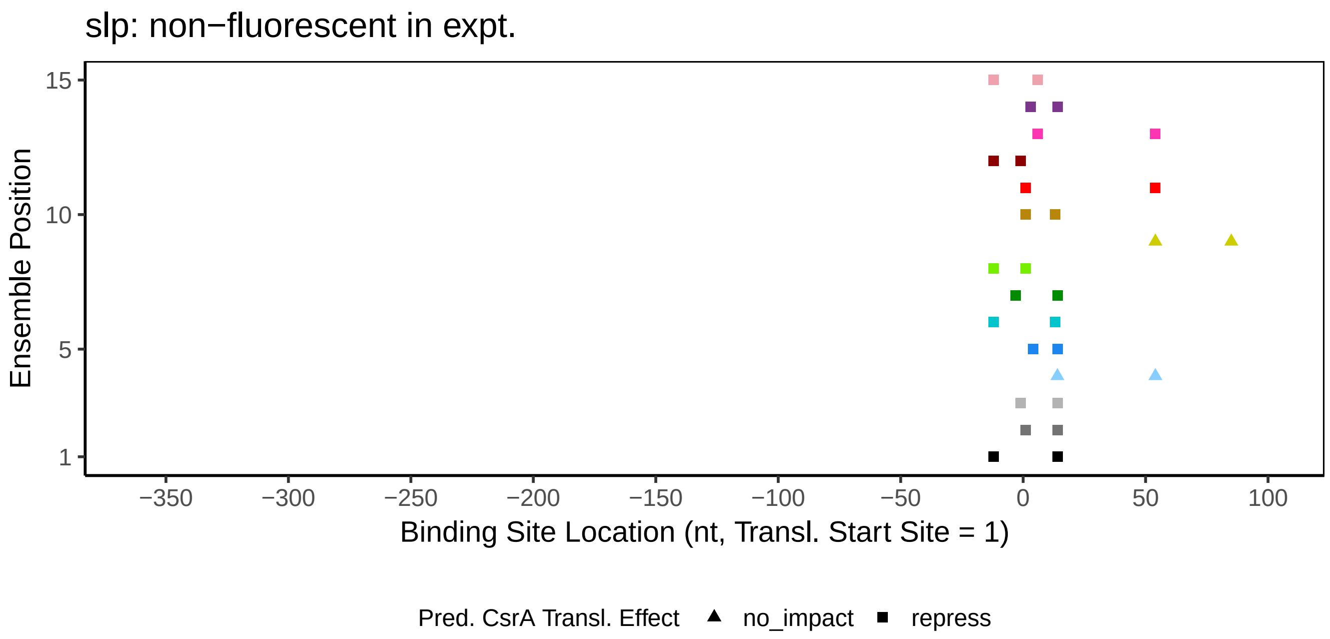
slp: non−fluorescent in expt.
Ensemble Position
Binding Site Location (nt, Transl. Start Site = 1)
Pred. CsrA Transl. Effect
no_impact
repress

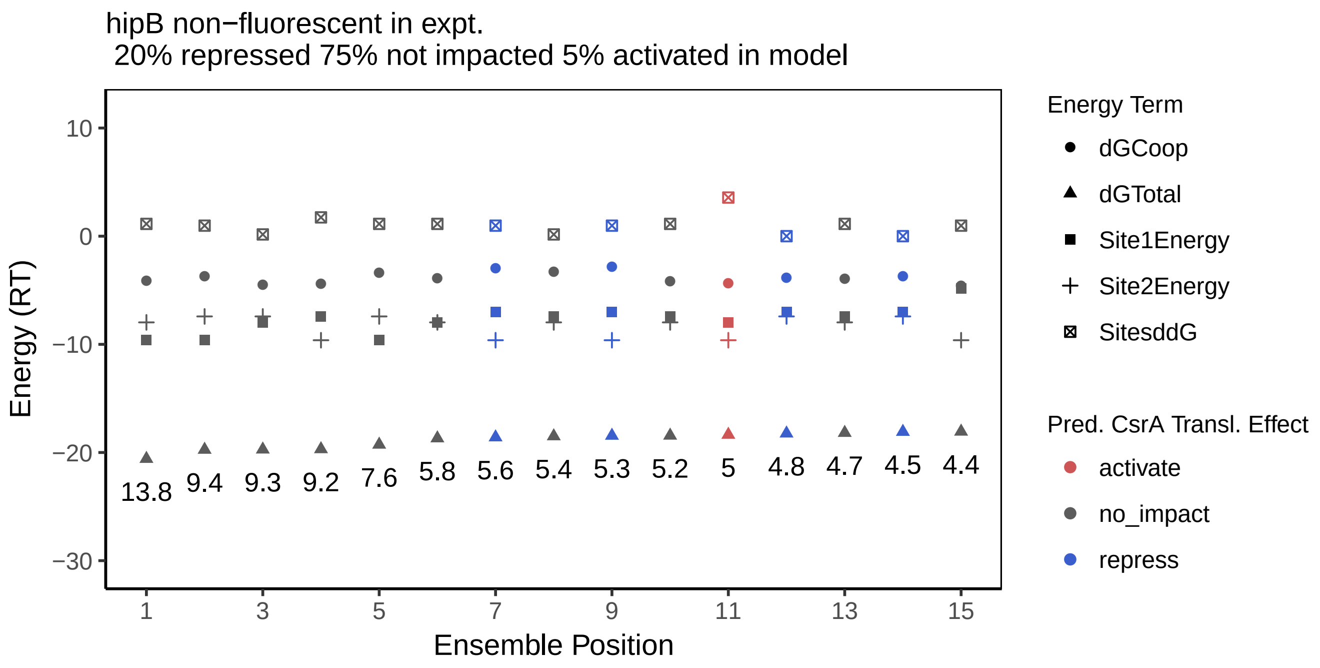

hipB non−fluorescent in expt.
20% repressed 75% not impacted 5% activated in model
Energy (RT)
10
0
−10
−20
−30
13.8
9.4
9.3
9.2
7.6
5.8
5.6
5.4
5.3
5.2
5
4.8
4.7
4.5
4.4
1
3
5
7
9
11
13
15
Ensemble Position
Energy Term
dGCoop
dGTotal
Site1Energy
Site2Energy
SitesddG
Pred. CsrA Transl. Effect
activate
no_impact
repress

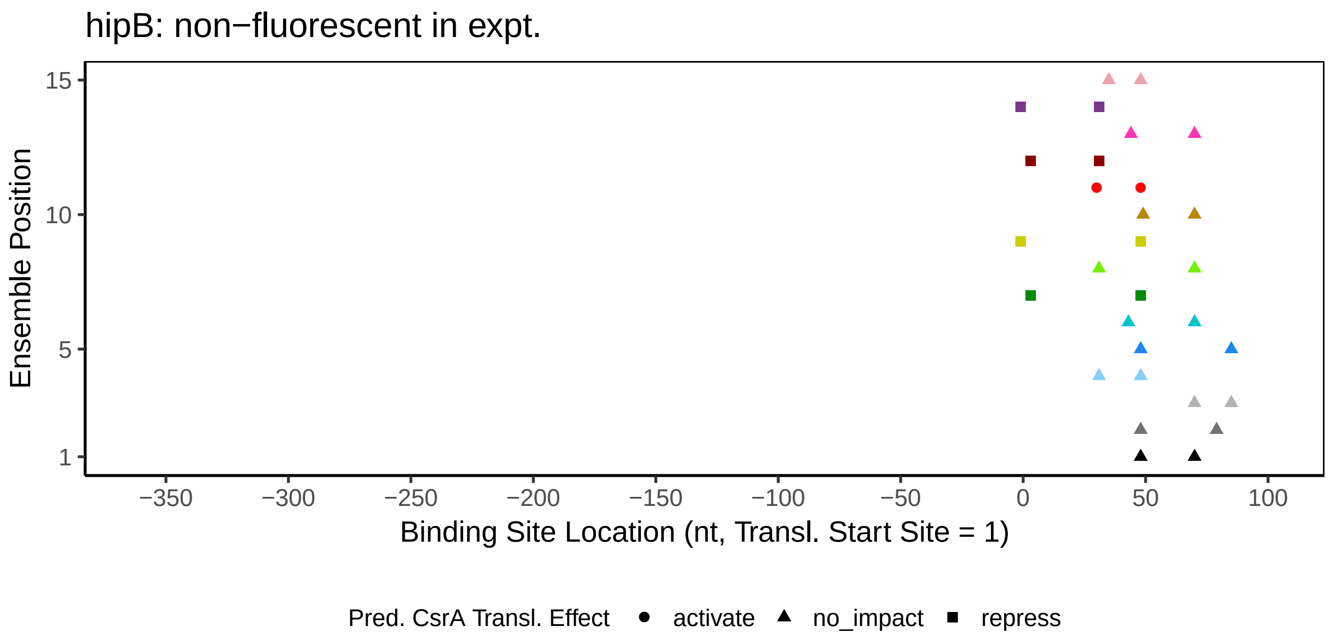

hipB: non−fluorescent in expt.
Ensemble Position
15
10
5
1
−350
−300
−250
−200
−150
−100
−50
0
50
100
Binding Site Location (nt, Transl. Start Site = 1)
Pred. CsrA Transl. Effect
activate
no_impact
repress

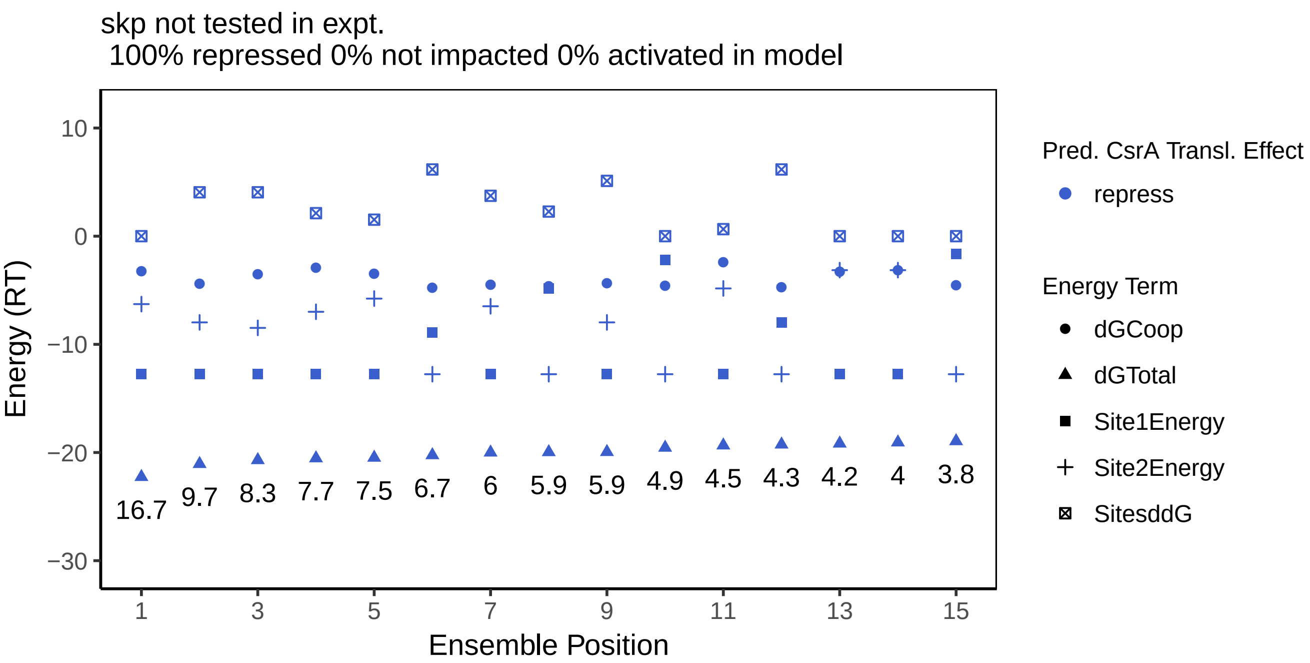
skp not tested in expt.
100% repressed 0% not impacted 0% activated in model
Energy (RT)
Ensemble Position
16.7
9.7
8.3
7.7
7.5
6.7
6
5.9
5.9
4.9
4.5
4.3
4.2
4
3.8
Pred. CsrA Transl. Effect
repress
Energy Term
dGCoop
dGTotal
Site1Energy
Site2Energy
SitesddG

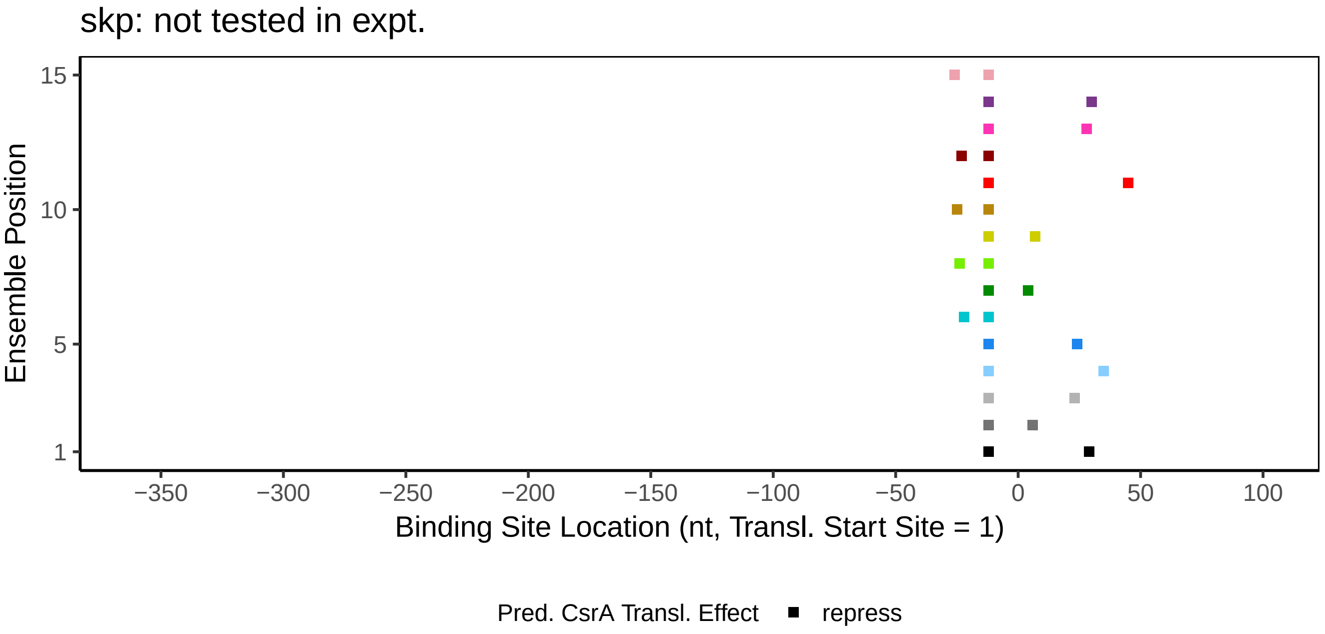
skp: not tested in expt.
Ensemble Position
Binding Site Location (nt, Transl. Start Site = 1)
Pred. CsrA Transl. Effect
repress

hyfR non−fluorescent in expt.
94% repressed 3% not impacted 3% activated in model

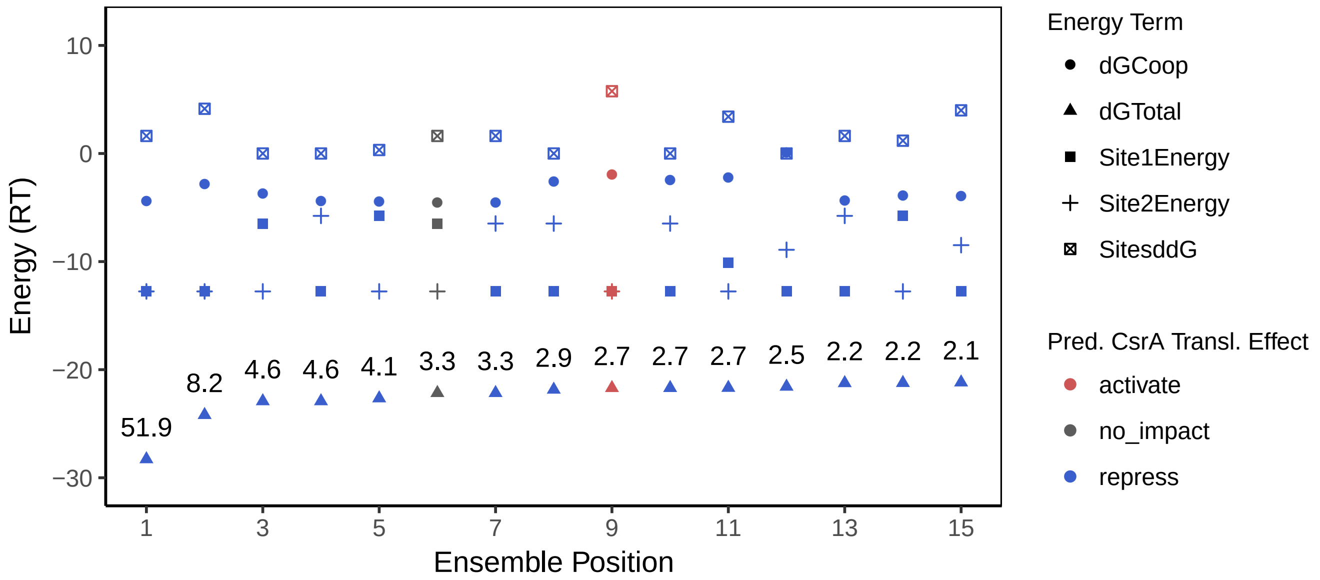

hyfR: non−fluorescent in expt.

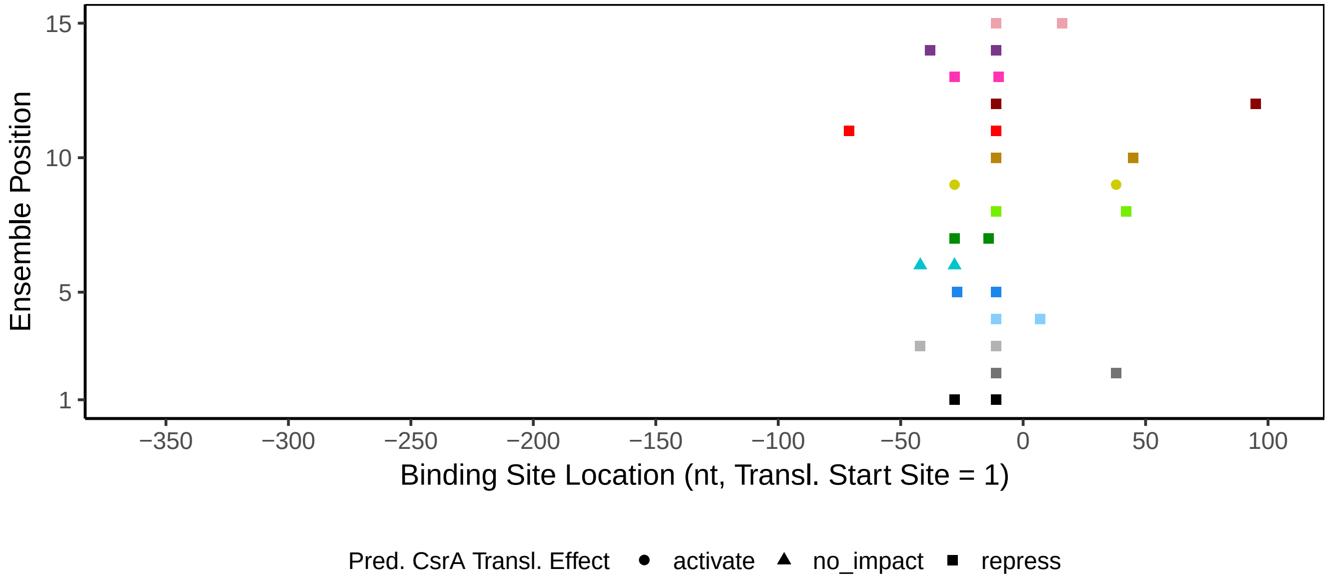

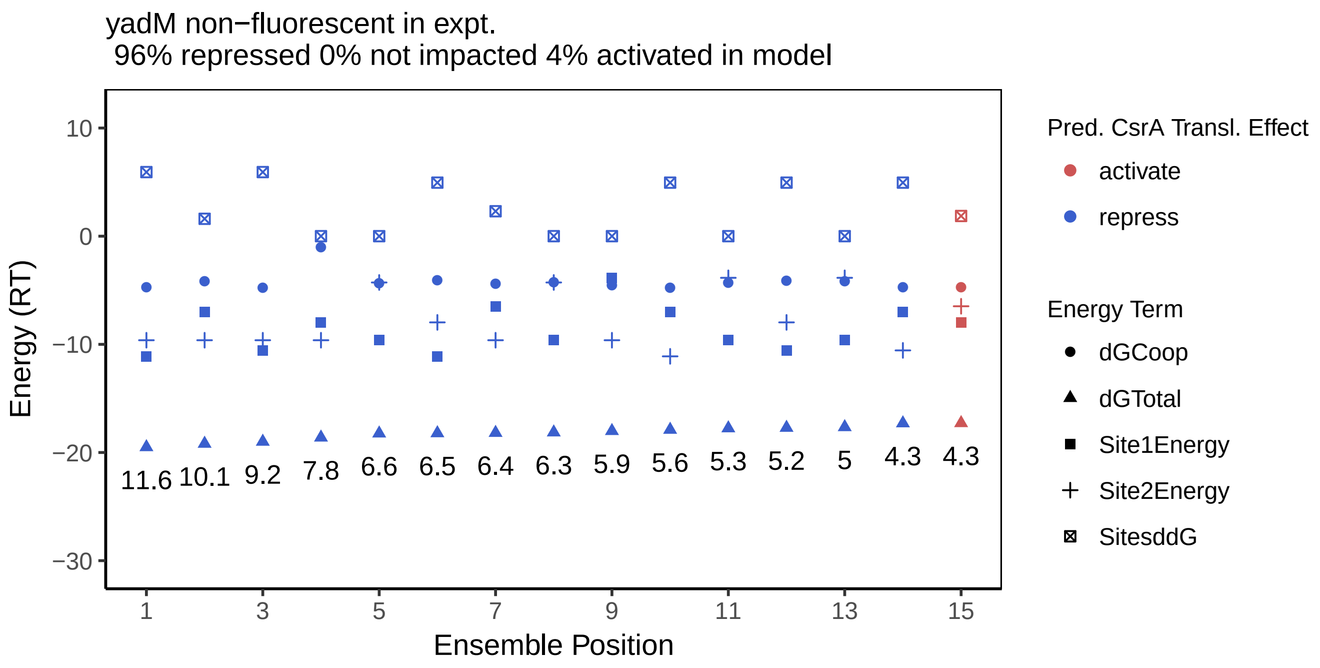
yadM non−fluorescent in expt.
96% repressed 0% not impacted 4% activated in model
Energy (RT)
10
0
−10
−20
−30
11.6
10.1
9.2
7.8
6.6
6.5
6.4
6.3
5.9
5.6
5.3
5.2
5
4.3
4.3
1
3
5
7
9
11
13
15
Ensemble Position
Pred. CsrA Transl. Effect
activate
repress
Energy Term
dGCoop
dGTotal
Site1Energy
Site2Energy
SitesddG

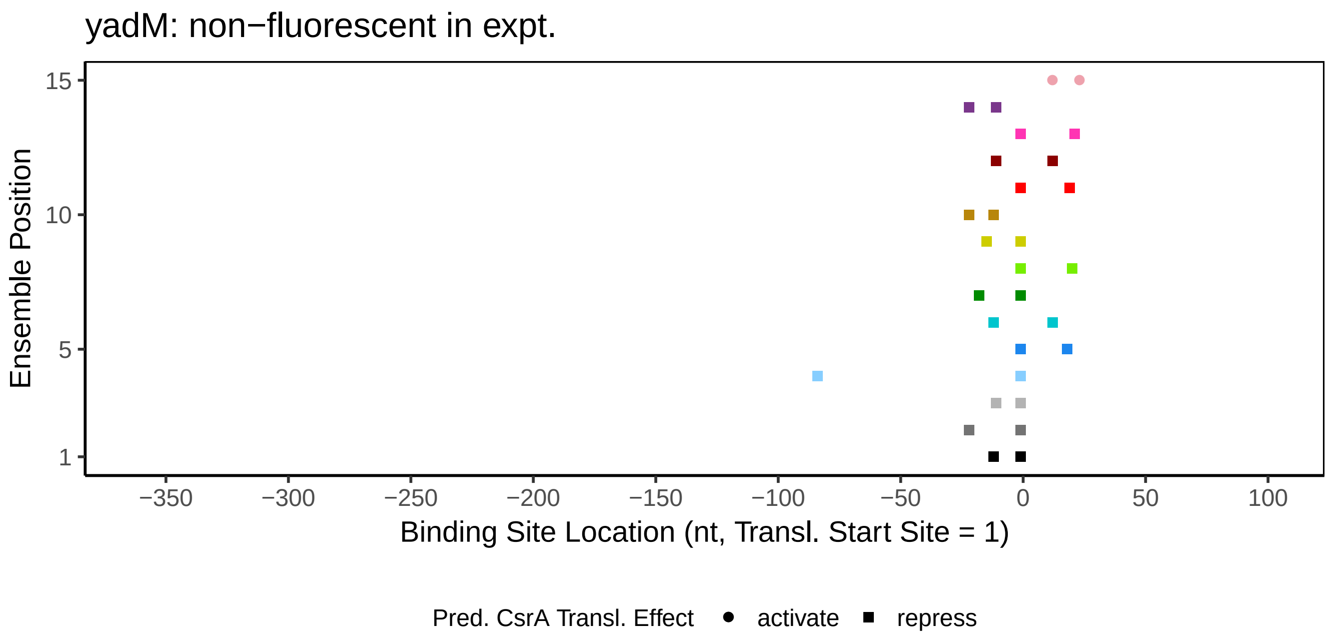
yadM: non−fluorescent in expt.
Ensemble Position
15
10
5
1
−350
−300
−250
−200
−150
−100
−50
0
50
100
Binding Site Location (nt, Transl. Start Site = 1)
Pred. CsrA Transl. Effect
activate
repress

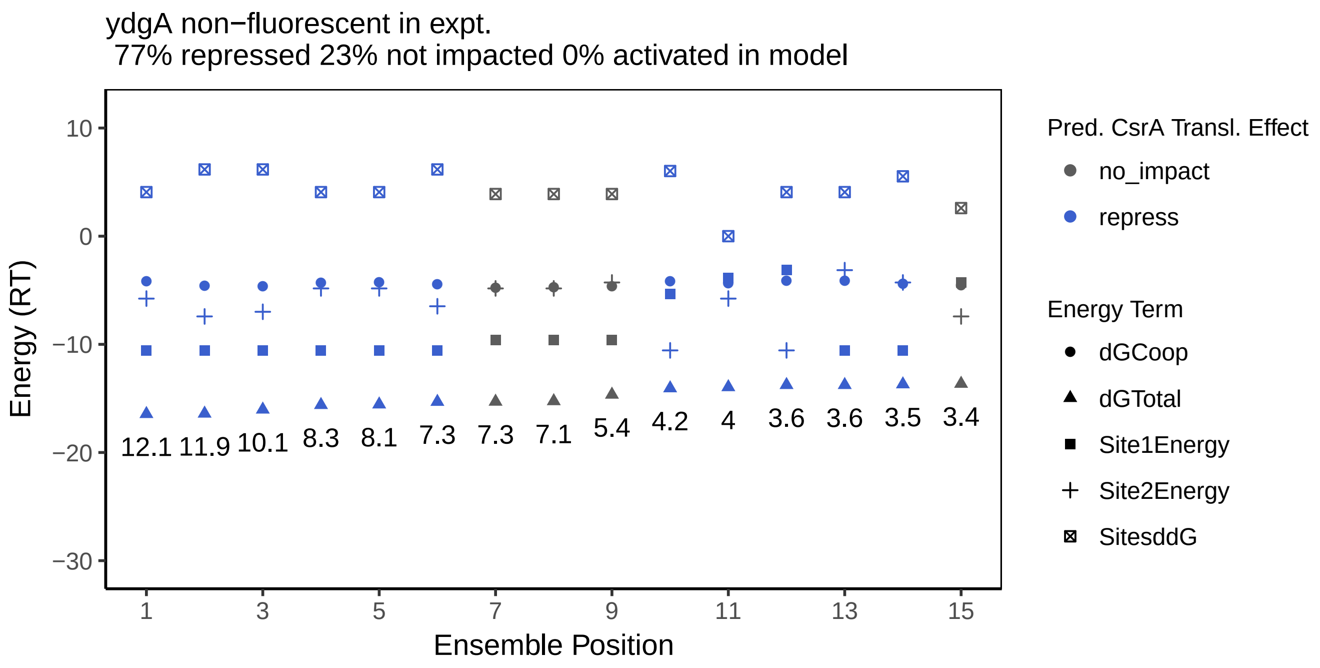
ydgA non−fluorescent in expt.
77% repressed 23% not impacted 0% activated in model
Energy (RT)
Ensemble Position
12.1 11.9 10.1 8.3 8.1 7.3 7.3 7.1 5.4 4.2 4 3.6 3.6 3.5 3.4
Pred. CsrA Transl. Effect
no_impact
repress
Energy Term
dGCoop
dGTotal
Site1Energy
Site2Energy
SitesddG

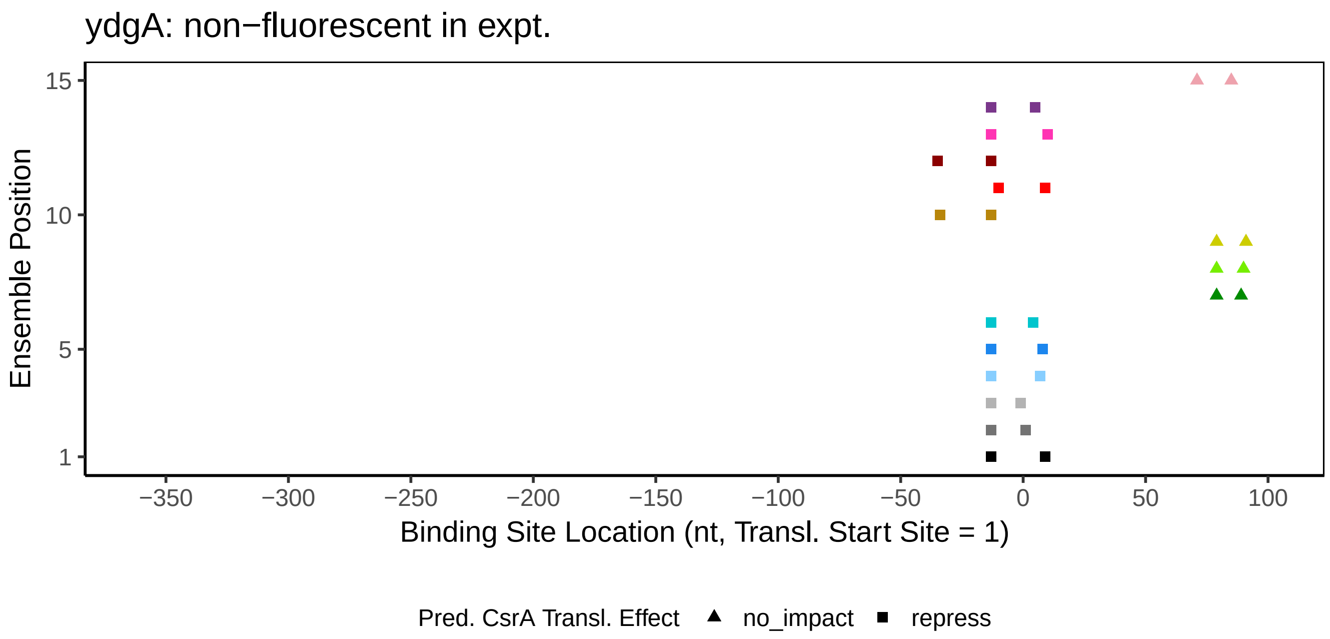
ydgA: non−fluorescent in expt.
Ensemble Position
Binding Site Location (nt, Transl. Start Site = 1)
Pred. CsrA Transl. Effect
no_impact
repress

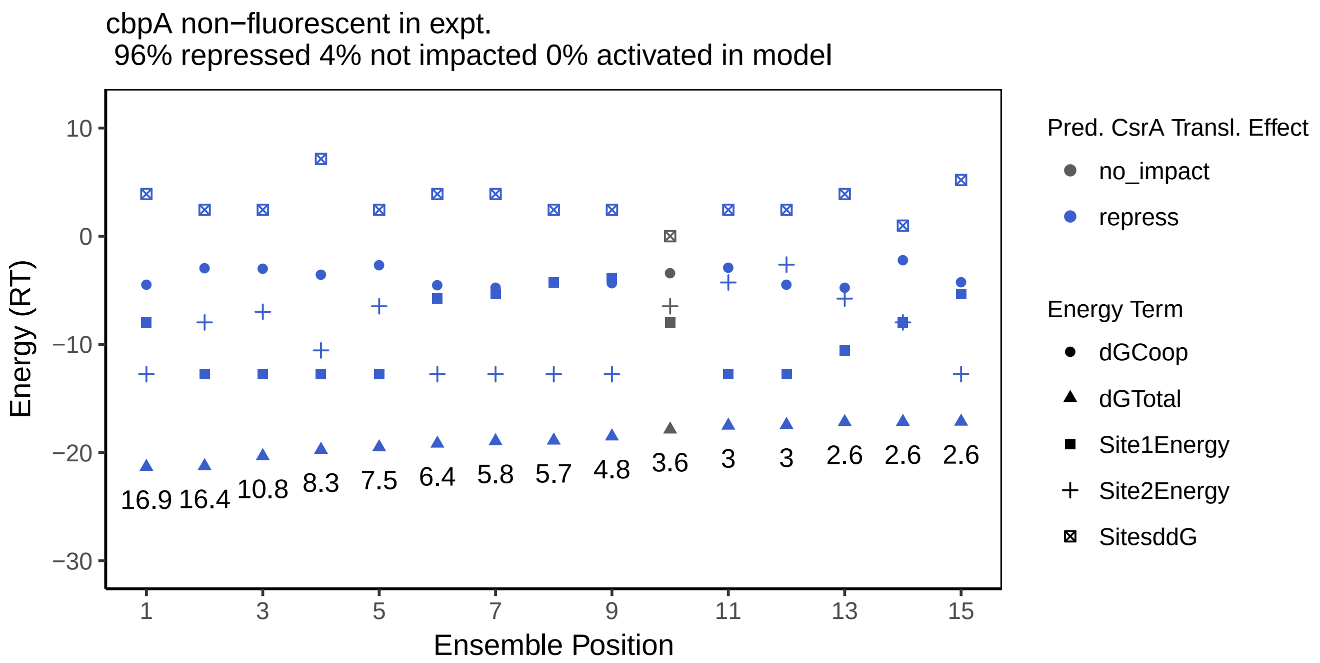

cbpA non−fluorescent in expt.
96% repressed 4% not impacted 0% activated in model
Energy (RT)
Ensemble Position
16.9 16.4 10.8 8.3 7.5 6.4 5.8 5.7 4.8 3.6 3 3 2.6 2.6 2.6
Pred. CsrA Transl. Effect
no_impact
repress
Energy Term
dGCoop
dGTotal
Site1Energy
Site2Energy
SitesddG

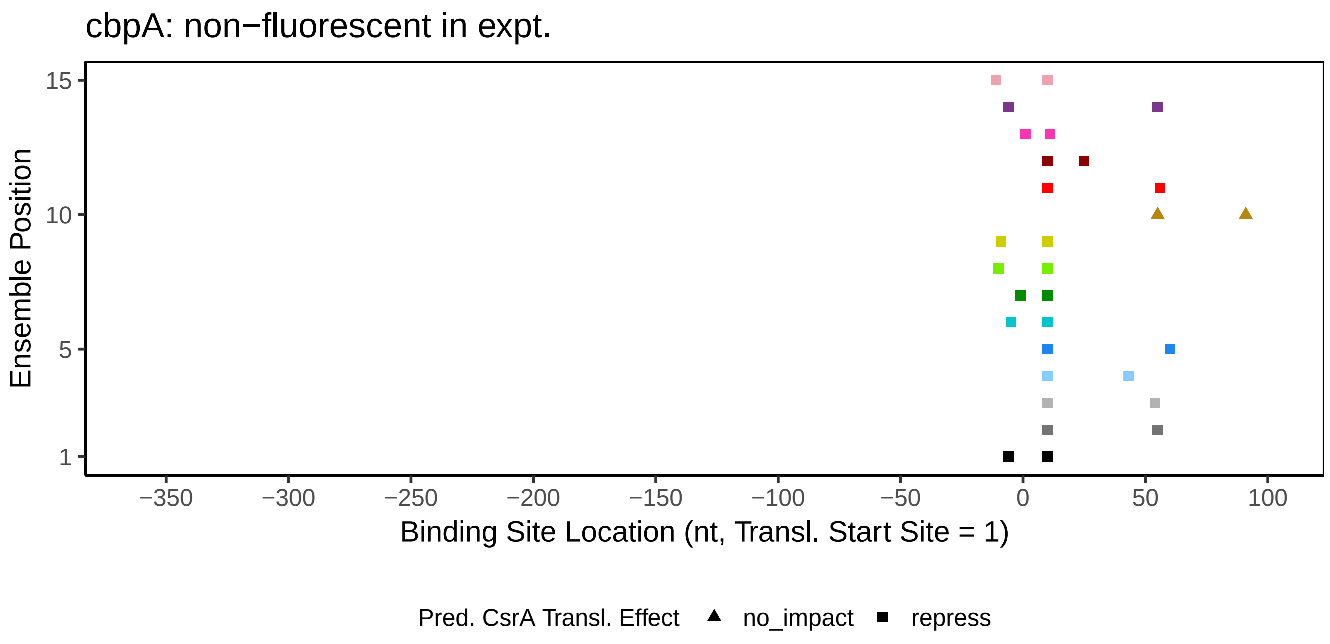

cbpA: non−fluorescent in expt.
Ensemble Position
Binding Site Location (nt, Transl. Start Site = 1)
Pred. CsrA Transl. Effect
no_impact
repress

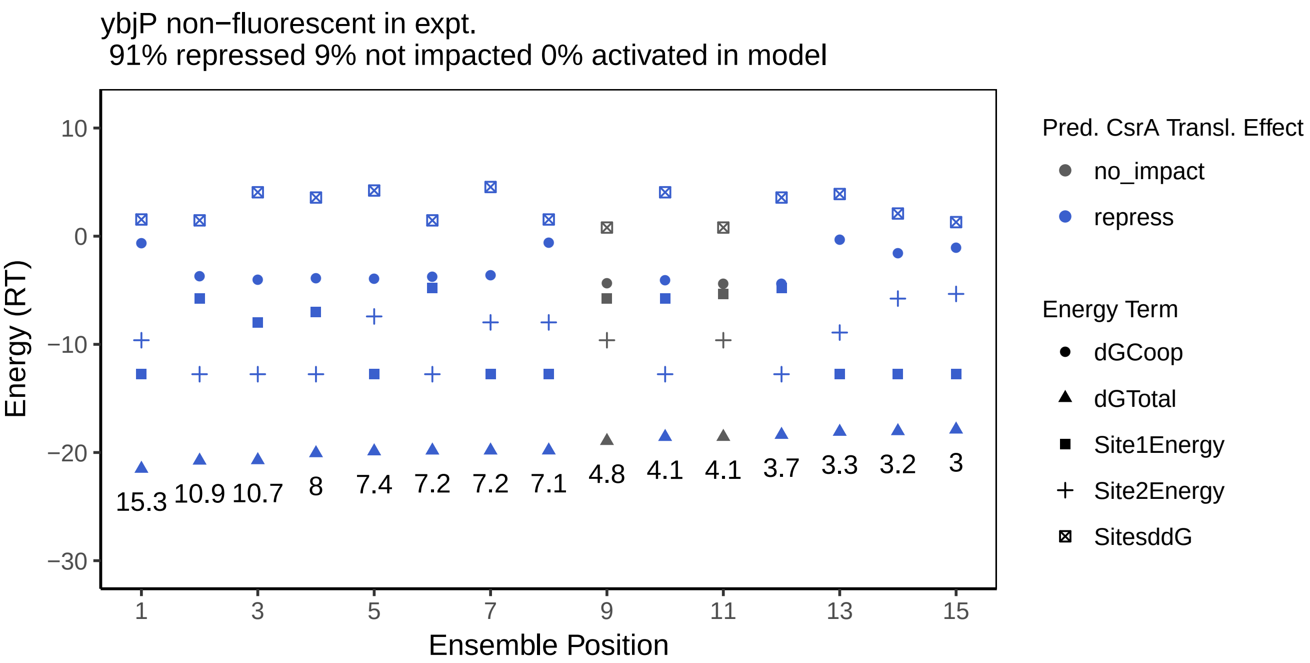
ybjP non−fluorescent in expt.
91% repressed 9% not impacted 0% activated in model
Energy (RT)
10
0
−10
−20
−30
15.3 10.9 10.7 8 7.4 7.2 7.2 7.1 4.8 4.1 4.1 3.7 3.3 3.2 3
1 3 5 7 9 11 13 15
Ensemble Position
Pred. CsrA Transl. Effect
no_impact
repress
Energy Term
dGCoop
dGTotal
Site1Energy
Site2Energy
SitesddG

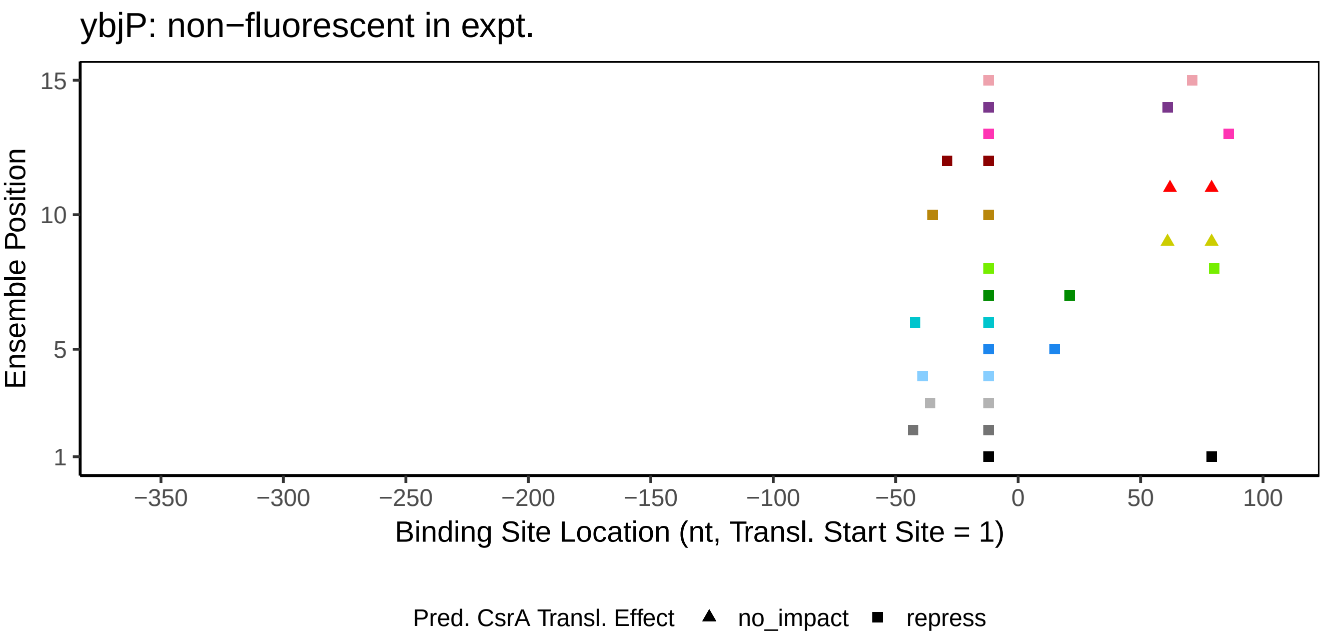
ybjP: non−fluorescent in expt.
Ensemble Position
15
10
5
1
−350 −300 −250 −200 −150 −100 −50 0 50 100
Binding Site Location (nt, Transl. Start Site = 1)
Pred. CsrA Transl. Effect
no_impact
repress

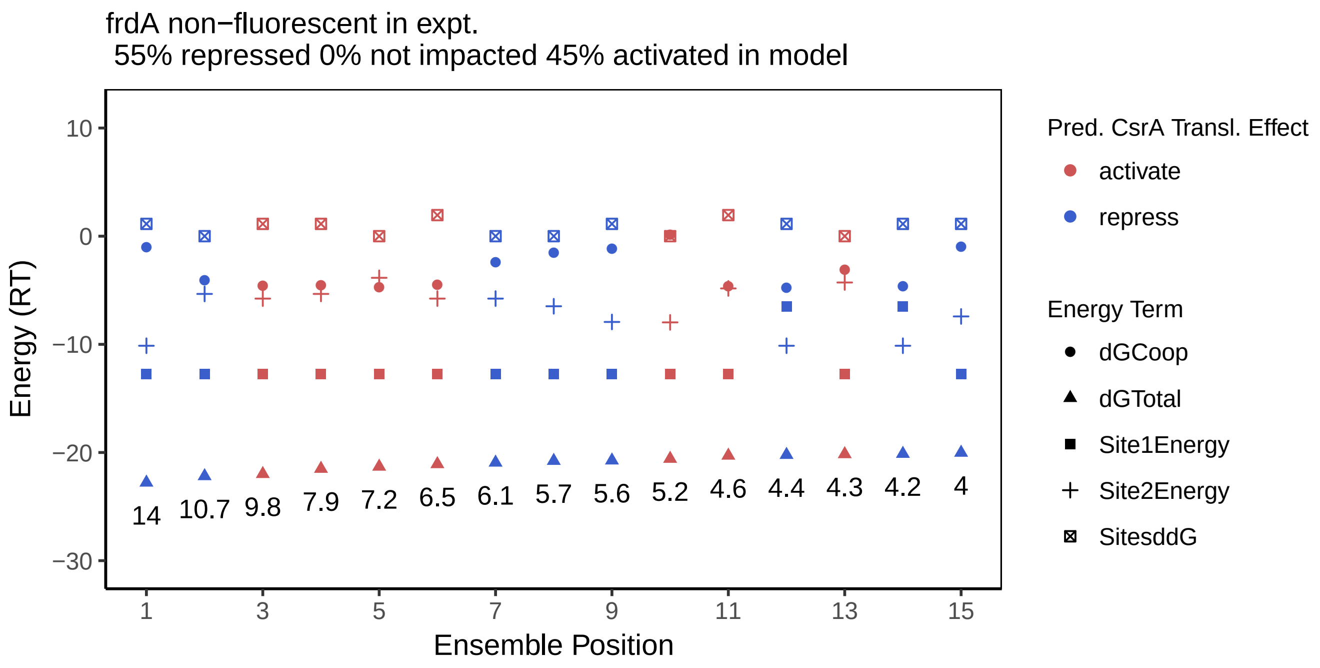

frdA non−fluorescent in expt.
55% repressed 0% not impacted 45% activated in model
Energy (RT)
10
0
−10
−20
−30
14
10.7
9.8
7.9
7.2
6.5
6.1
5.7
5.6
5.2
4.6
4.4
4.3
4.2
4
1
3
5
7
9
11
13
15
Ensemble Position
Pred. CsrA Transl. Effect
activate
repress
Energy Term
dGCoop
dGTotal
Site1Energy
Site2Energy
SitesddG

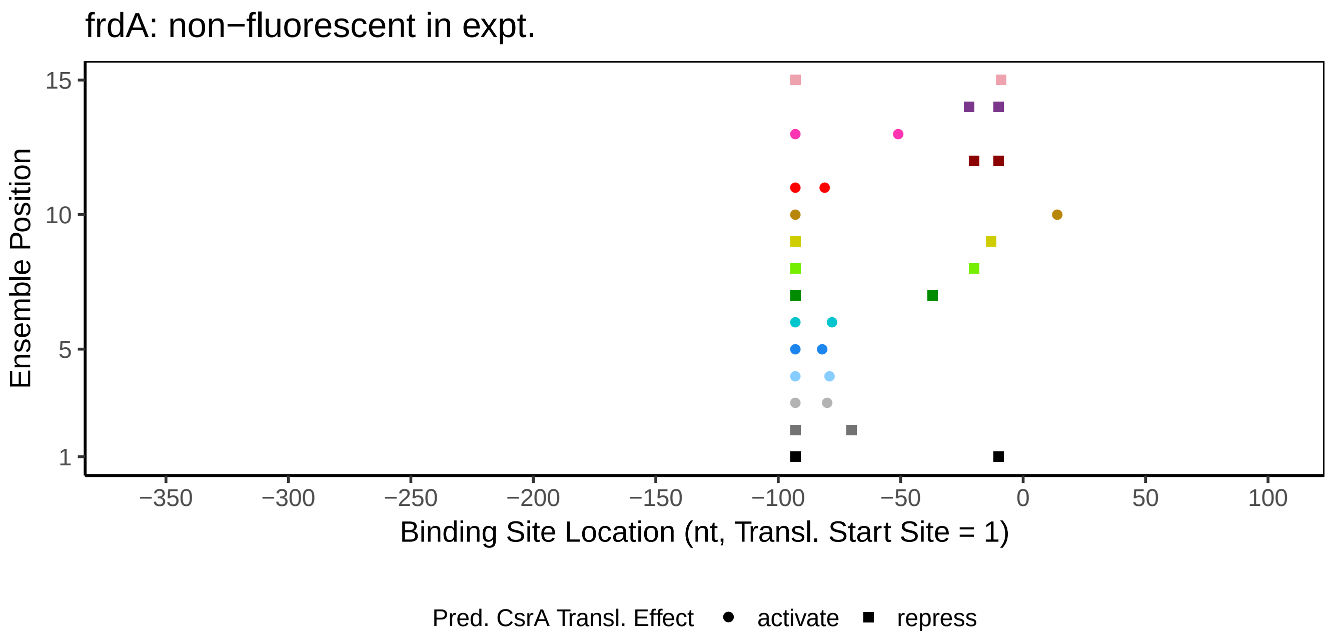

frdA: non−fluorescent in expt.
Ensemble Position
15
10
5
1
−350
−300
−250
−200
−150
−100
−50
0
50
100
Binding Site Location (nt, Transl. Start Site = 1)
Pred. CsrA Transl. Effect
activate
repress

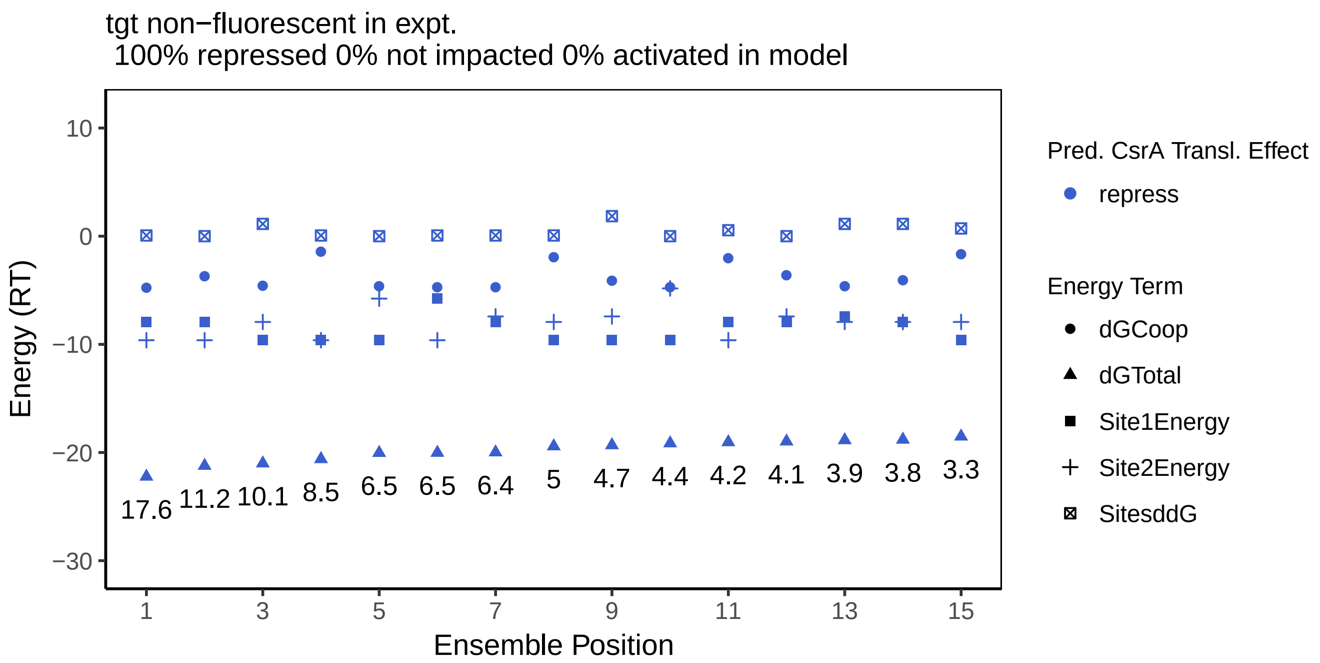
tgt non−fluorescent in expt.
100% repressed 0% not impacted 0% activated in model
Energy (RT)
Ensemble Position
17.6
11.2
10.1
8.5
6.5
6.5
6.4
5
4.7
4.4
4.2
4.1
3.9
3.8
3.3
Pred. CsrA Transl. Effect
repress
Energy Term
dGCoop
dGTotal
Site1Energy
Site2Energy
SitesddG

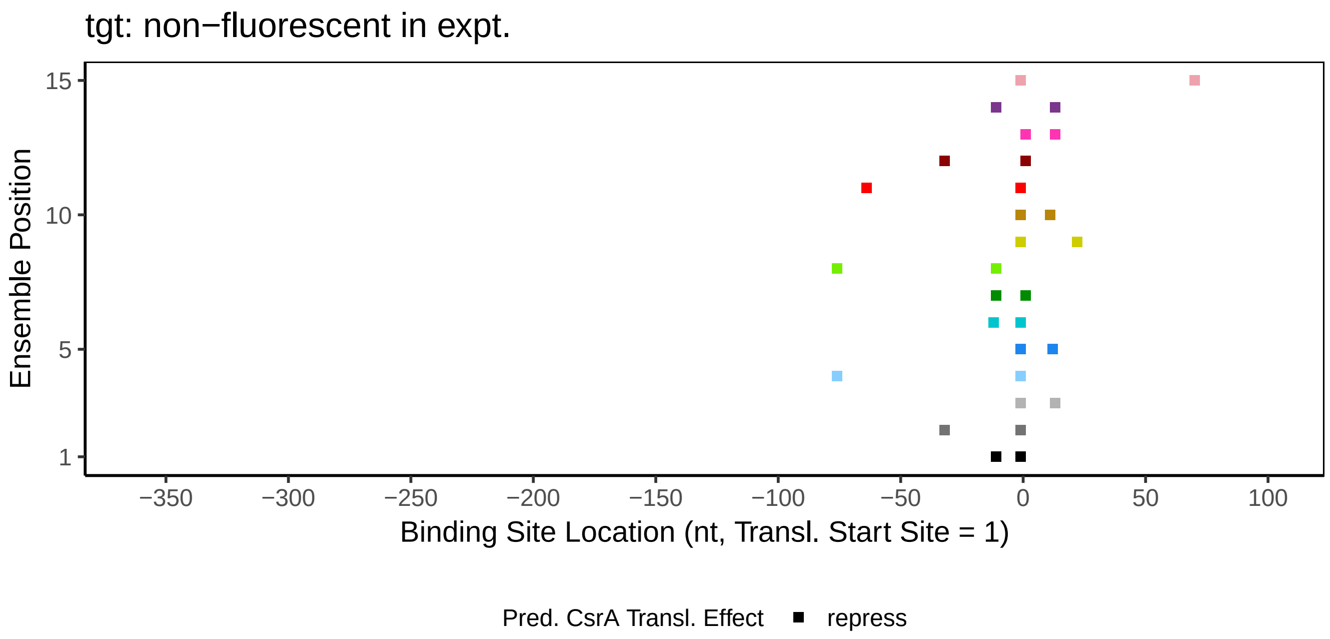
tgt: non−fluorescent in expt.
Ensemble Position
Binding Site Location (nt, Transl. Start Site = 1)
Pred. CsrA Transl. Effect
repress

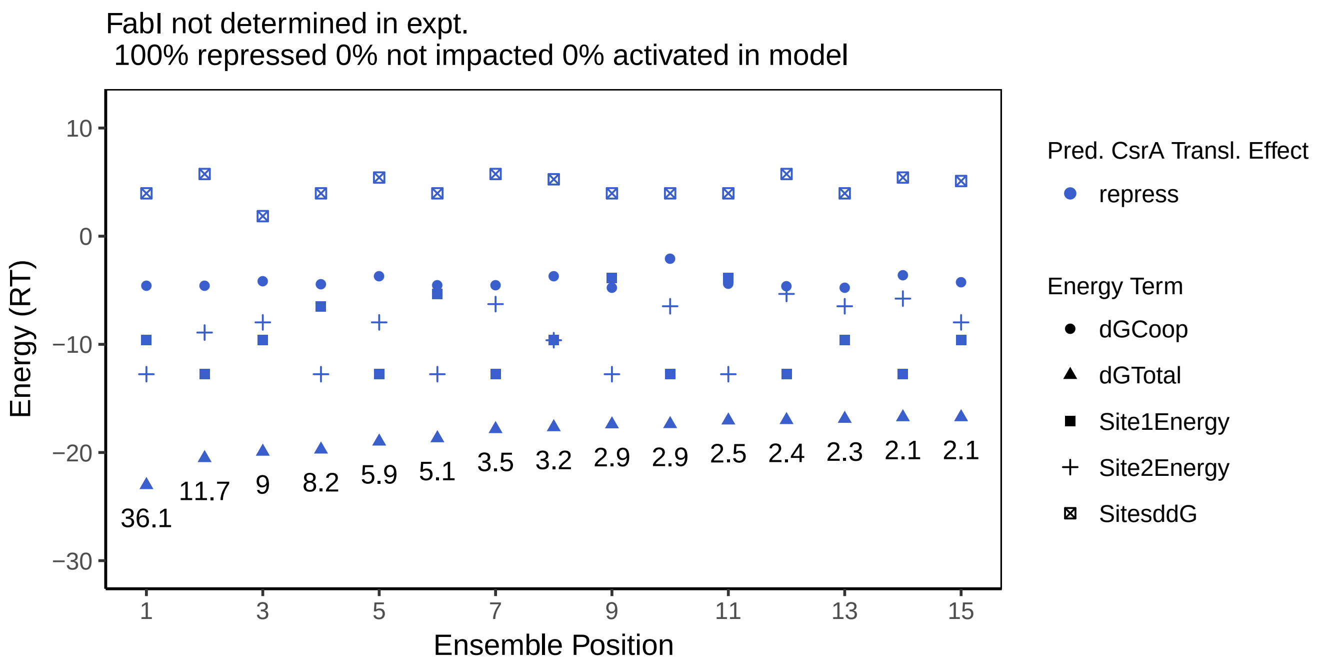
FabI not determined in expt.
100% repressed 0% not impacted 0% activated in model
Energy (RT)
10
0
−10
−20
−30
36.1
11.7
9
8.2
5.9
5.1
3.5
3.2
2.9
2.9
2.5
2.4
2.3
2.1
2.1
1
3
5
7
9
11
13
15
Ensemble Position
Pred. CsrA Transl. Effect
repress
Energy Term
dGCoop
dGTotal
Site1Energy
Site2Energy
SitesddG

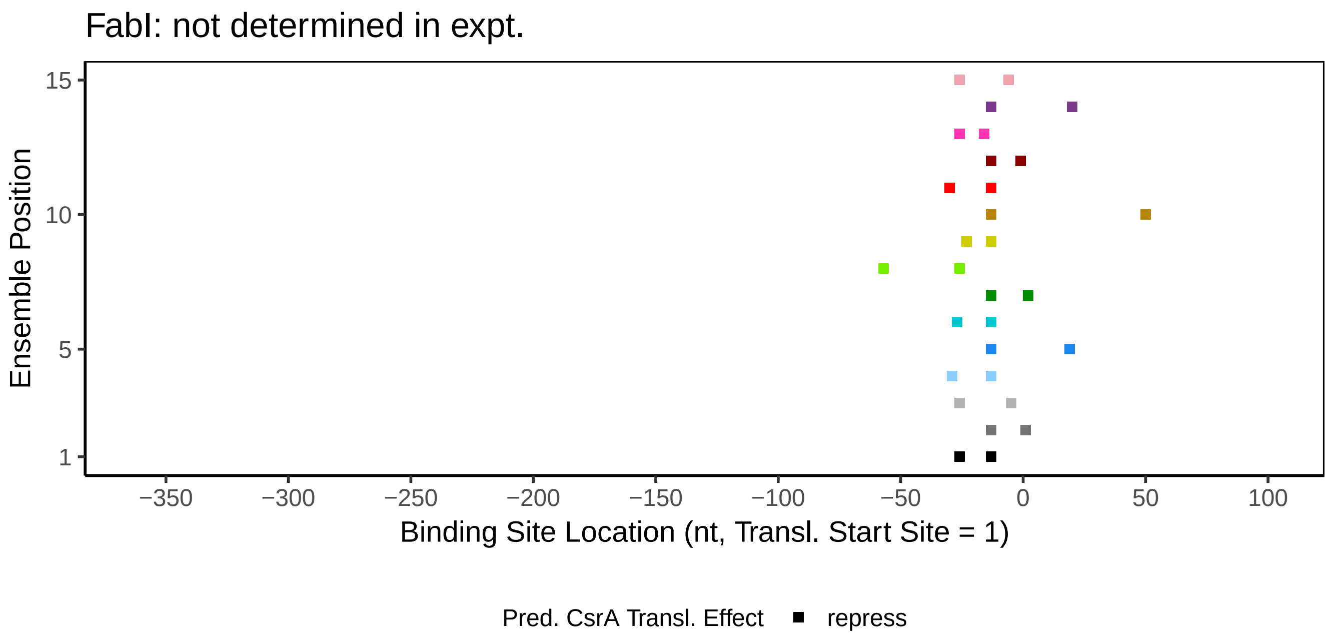
FabI: not determined in expt.
Ensemble Position
15
10
5
1
−350
−300
−250
−200
−150
−100
−50
0
50
100
Binding Site Location (nt, Transl. Start Site = 1)
Pred. CsrA Transl. Effect
repress

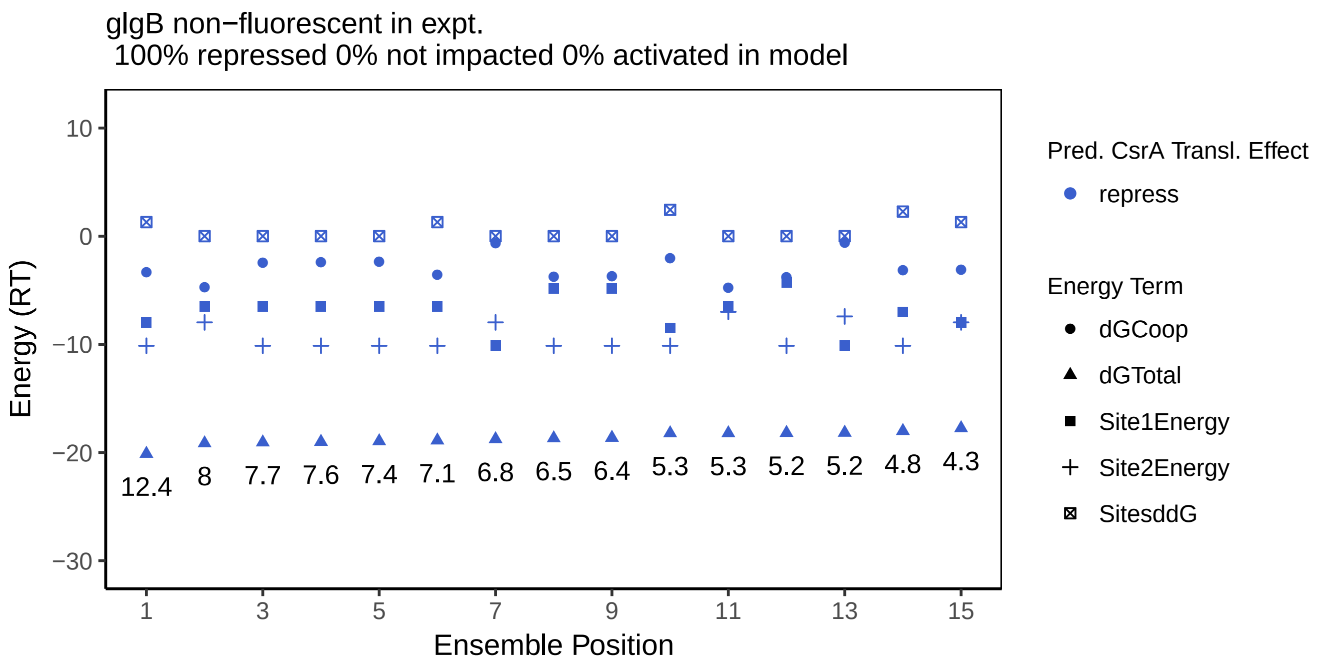
glgB non−fluorescent in expt.
100% repressed 0% not impacted 0% activated in model
Energy (RT)
10
0
−10
−20
−30
12.4
8
7.7
7.6
7.4
7.1
6.8
6.5
6.4
5.3
5.3
5.2
5.2
4.8
4.3
1
3
5
7
9
11
13
15
Ensemble Position
Pred. CsrA Transl. Effect
repress
Energy Term
dGCoop
dGTotal
Site1Energy
Site2Energy
SitesddG

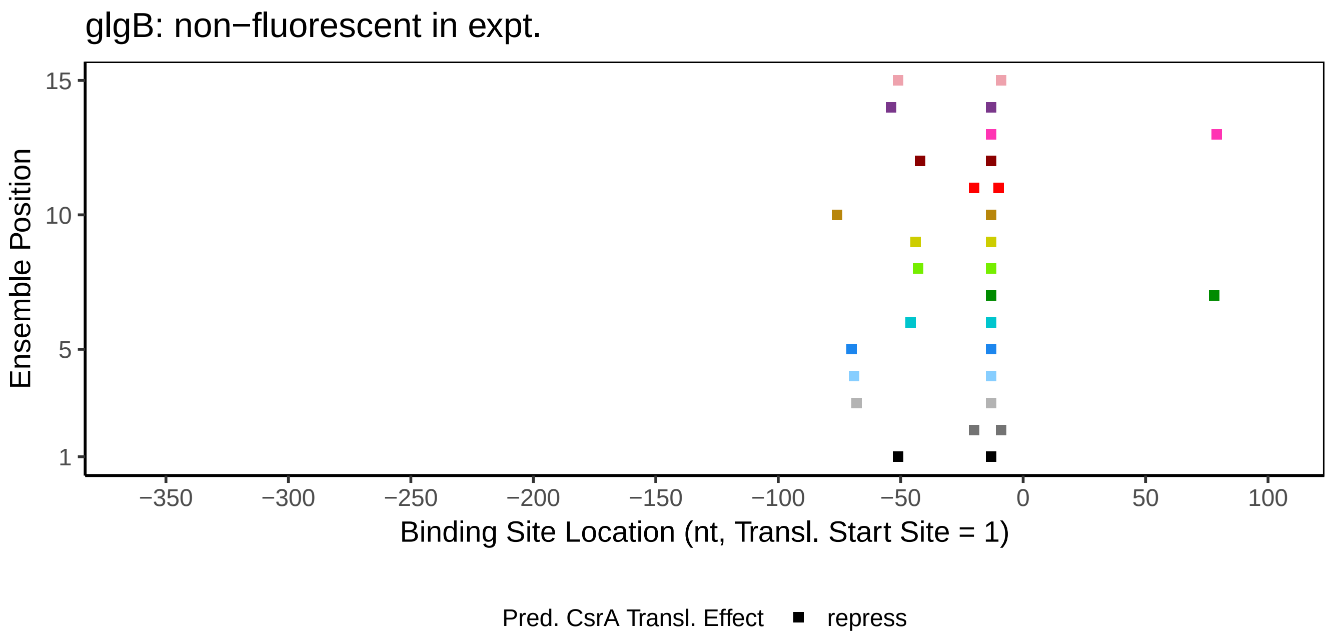
glgB: non−fluorescent in expt.
Ensemble Position
15
10
5
1
−350
−300
−250
−200
−150
−100
−50
0
50
100
Binding Site Location (nt, Transl. Start Site = 1)
Pred. CsrA Transl. Effect
repress

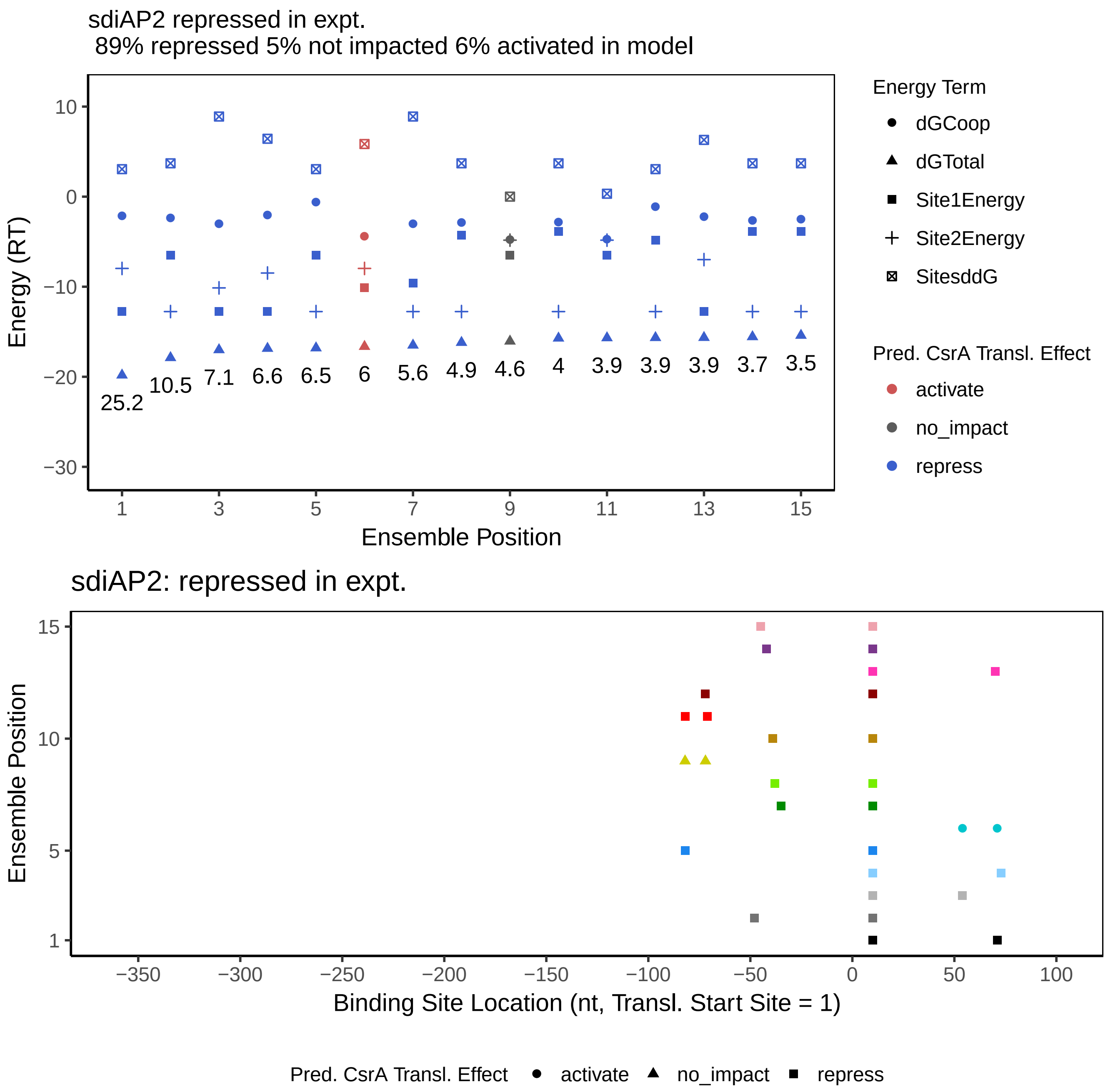
sdiAP2 repressed in expt.
89% repressed 5% not impacted 6% activated in model
Energy (RT)
Ensemble Position
25.2
10.5
7.1
6.6
6.5
6
5.6
4.9
4.6
4
3.9
3.9
3.9
3.7
3.5
Energy Term
dGCoop
dGTotal
Site1Energy
Site2Energy
SitesddG
Pred. CsrA Transl. Effect
activate
no_impact
repress
sdiAP2: repressed in expt.
Ensemble Position
Binding Site Location (nt, Transl. Start Site = 1)
Pred. CsrA Transl. Effect
activate
no_impact
repress

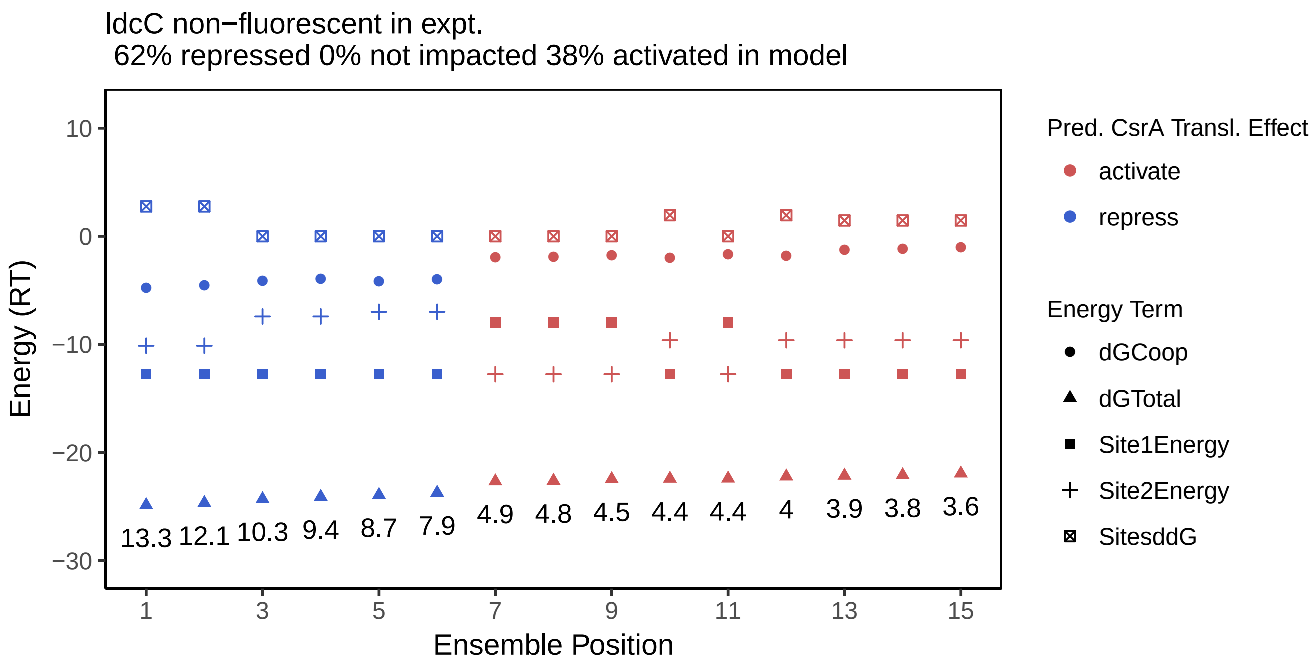
ldcC non−fluorescent in expt.
62% repressed 0% not impacted 38% activated in model
Energy (RT)
10
0
−10
−20
−30
13.3 12.1 10.3 9.4 8.7 7.9 4.9 4.8 4.5 4.4 4.4 4 3.9 3.8 3.6
1 3 5 7 9 11 13 15
Ensemble Position
Pred. CsrA Transl. Effect
activate
repress
Energy Term
dGCoop
dGTotal
Site1Energy
Site2Energy
SitesddG

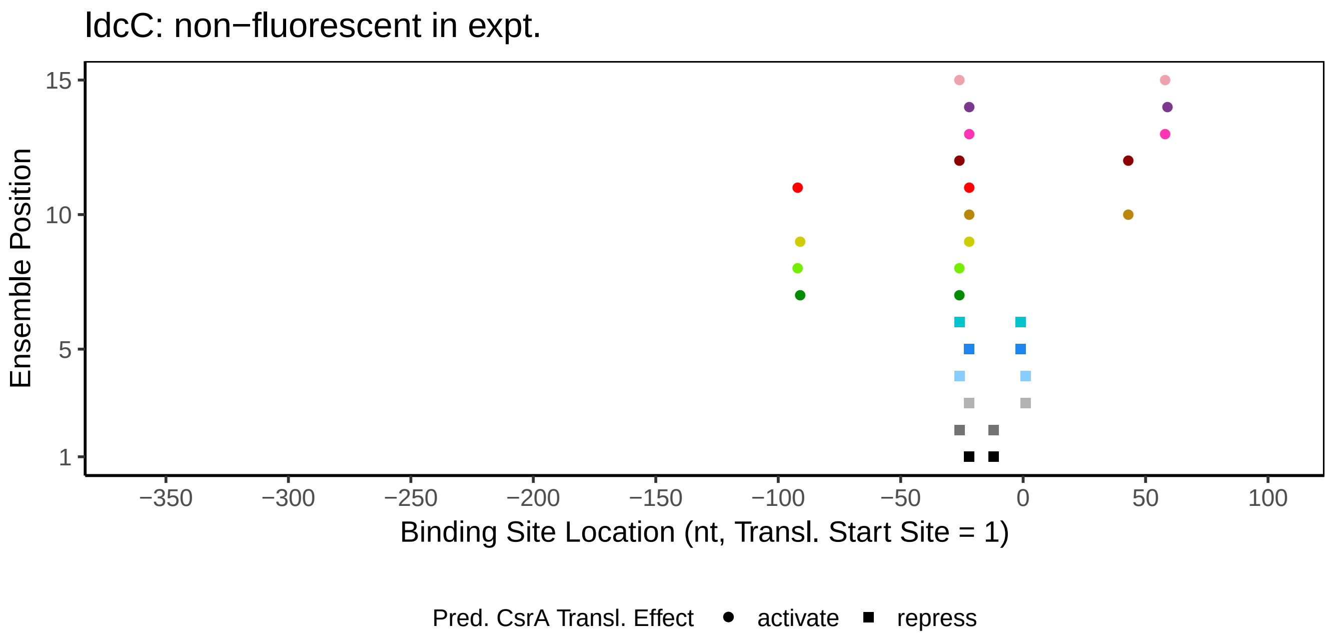
ldcC: non−fluorescent in expt.
Ensemble Position
15
10
5
1
−350 −300 −250 −200 −150 −100 −50 0 50 100
Binding Site Location (nt, Transl. Start Site = 1)
Pred. CsrA Transl. Effect
activate
repress

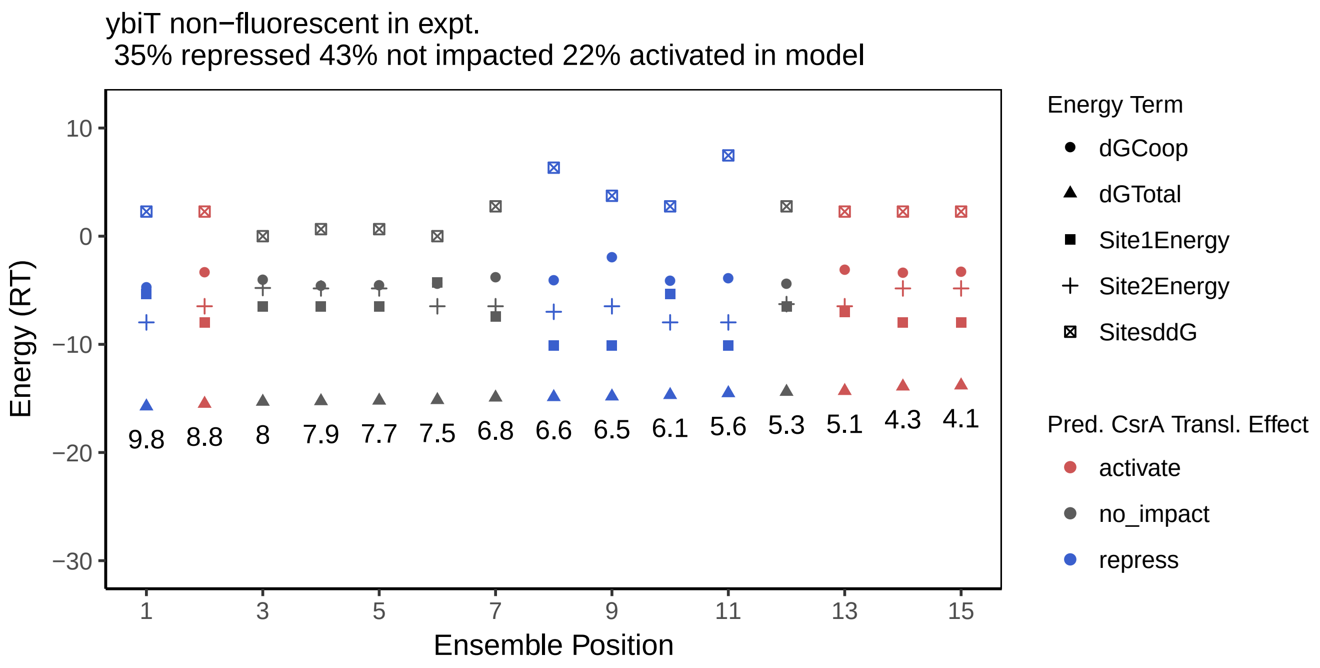
ybiT non−fluorescent in expt.
35% repressed 43% not impacted 22% activated in model
Energy (RT)
10
0
−10
−20
−30
9.8
8.8
8
7.9
7.7
7.5
6.8
6.6
6.5
6.1
5.6
5.3
5.1
4.3
4.1
1
3
5
7
9
11
13
15
Ensemble Position
Energy Term
dGCoop
dGTotal
Site1Energy
Site2Energy
SitesddG
Pred. CsrA Transl. Effect
activate
no_impact
repress

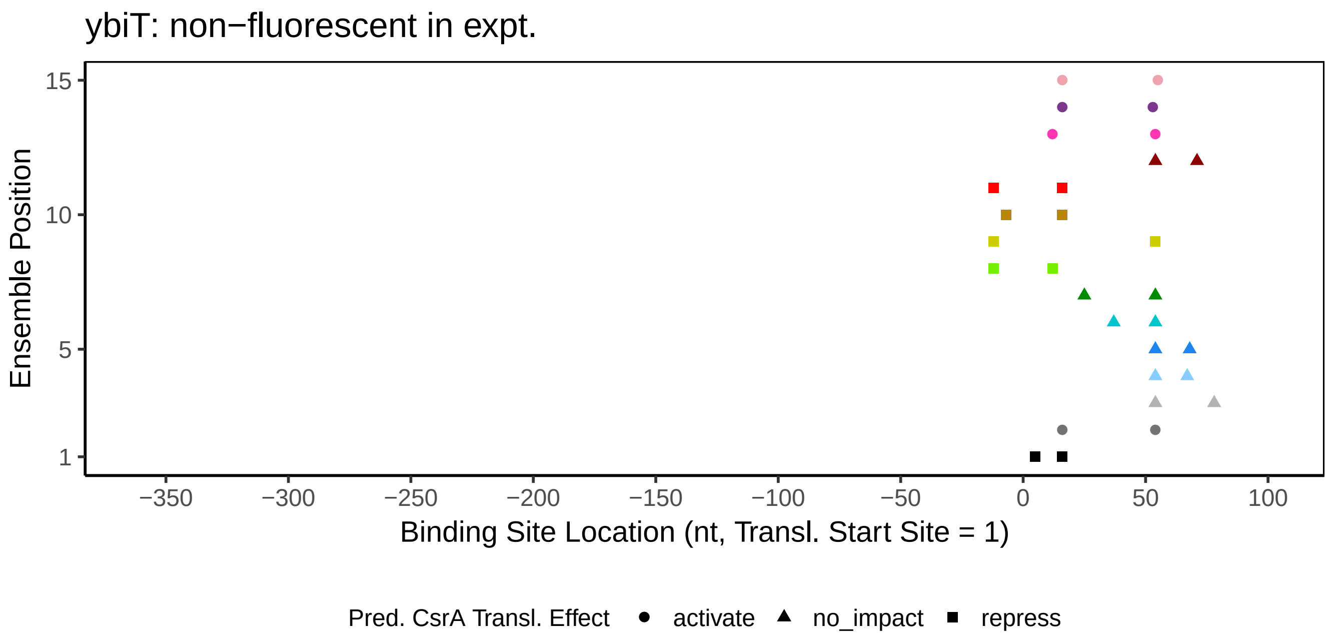
ybiT: non−fluorescent in expt.
Ensemble Position
15
10
5
1
−350
−300
−250
−200
−150
−100
−50
0
50
100
Binding Site Location (nt, Transl. Start Site = 1)
Pred. CsrA Transl. Effect
activate
no_impact
repress

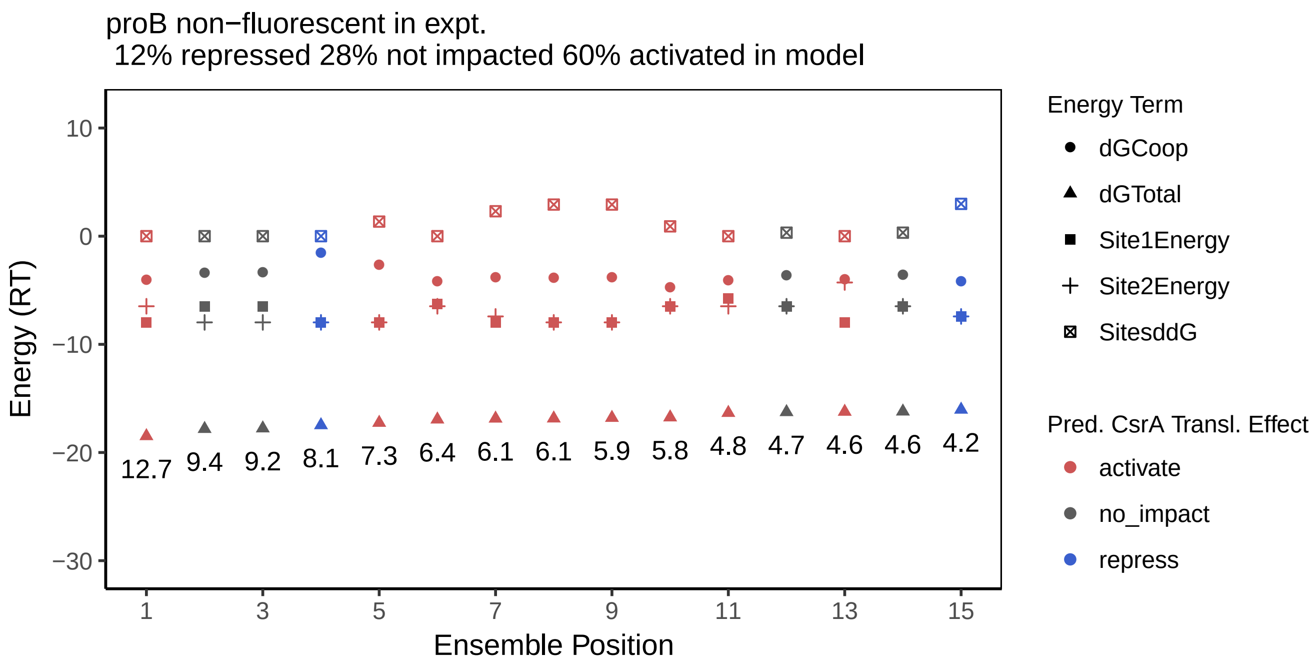

proB non−fluorescent in expt.
12% repressed 28% not impacted 60% activated in model
Energy (RT)
10
0
−10
−20
−30
12.7
9.4
9.2
8.1
7.3
6.4
6.1
6.1
5.9
5.8
4.8
4.7
4.6
4.6
4.2
1
3
5
7
9
11
13
15
Ensemble Position
Energy Term
dGCoop
dGTotal
Site1Energy
Site2Energy
SitesddG
Pred. CsrA Transl. Effect
activate
no_impact
repress

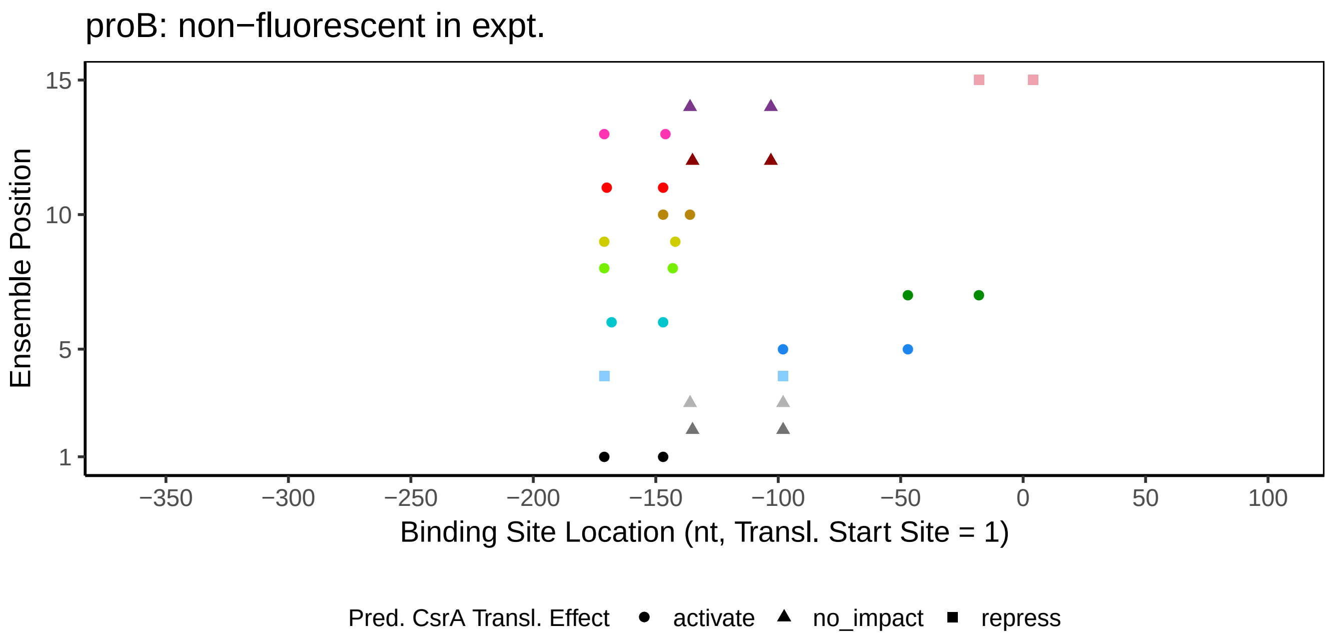

proB: non−fluorescent in expt.
Ensemble Position
15
10
5
1
−350
−300
−250
−200
−150
−100
−50
0
50
100
Binding Site Location (nt, Transl. Start Site = 1)
Pred. CsrA Transl. Effect
activate
no_impact
repress

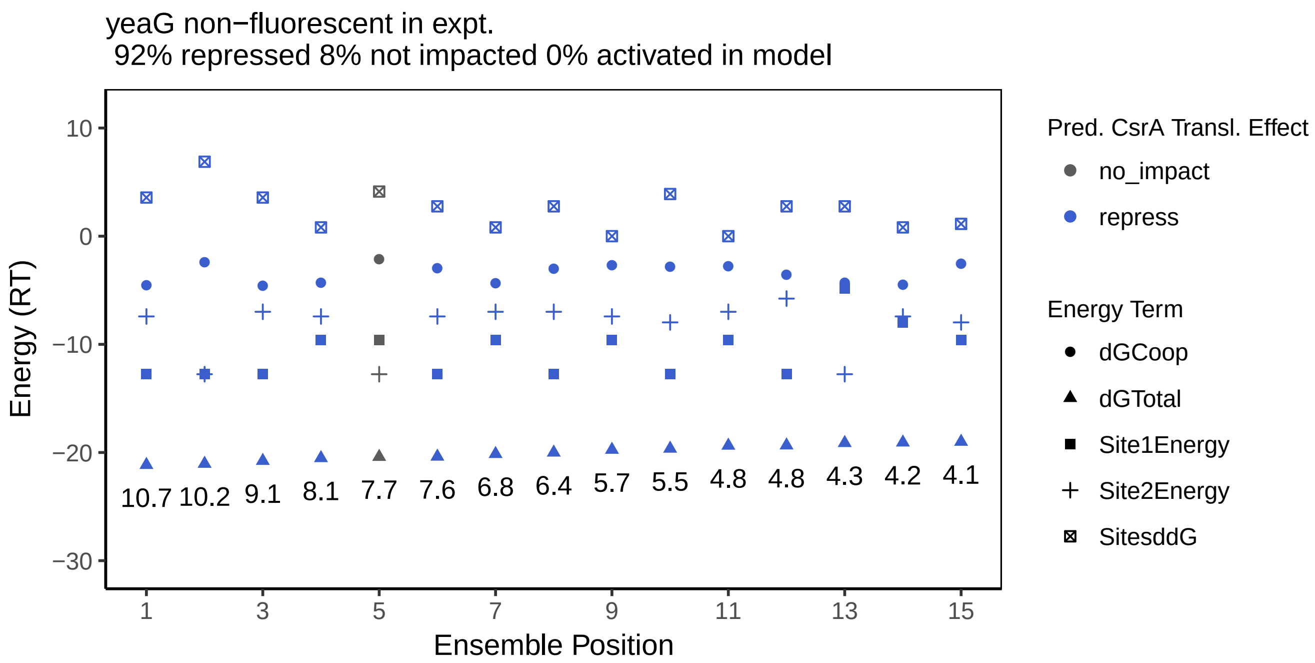
yeaG non−fluorescent in expt.
92% repressed 8% not impacted 0% activated in model
Energy (RT)
10
0
−10
−20
−30
10.7 10.2 9.1 8.1 7.7 7.6 6.8 6.4 5.7 5.5 4.8 4.8 4.3 4.2 4.1
1
3
5
7
9
11
13
15
Ensemble Position
Pred. CsrA Transl. Effect
no_impact
repress
Energy Term
dGCoop
dGTotal
Site1Energy
Site2Energy
SitesddG

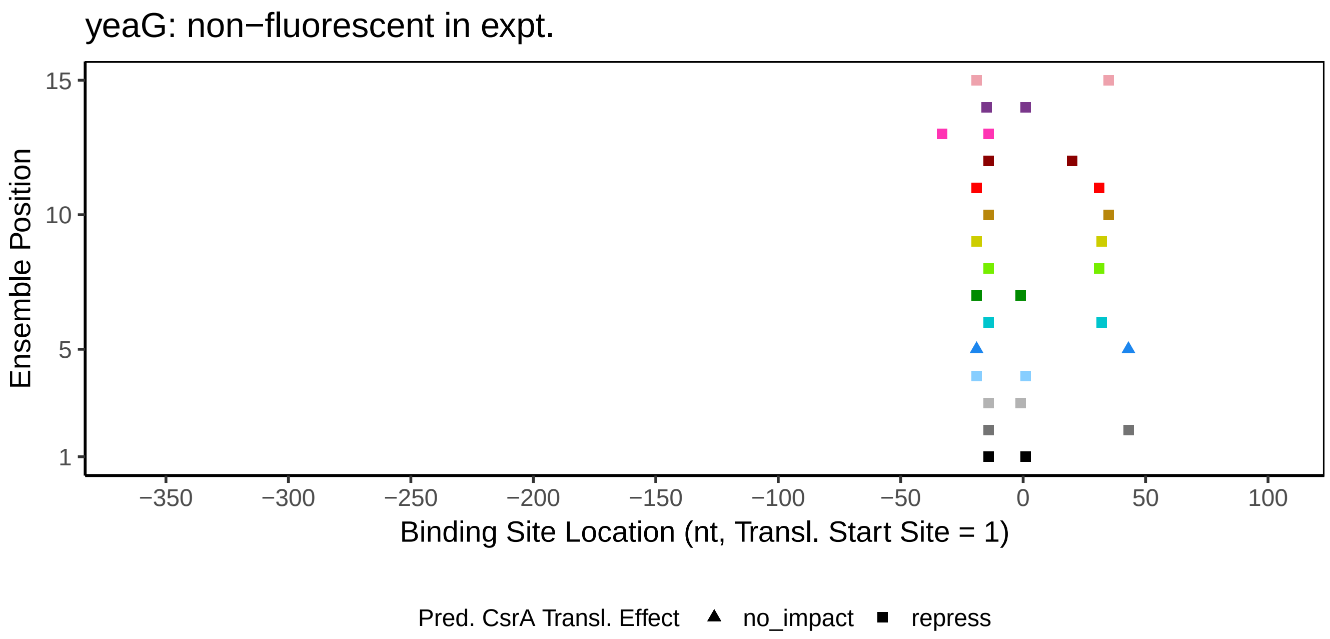
yeaG: non−fluorescent in expt.
Ensemble Position
15
10
5
1
−350
−300
−250
−200
−150
−100
−50
0
50
100
Binding Site Location (nt, Transl. Start Site = 1)
Pred. CsrA Transl. Effect
no_impact
repress

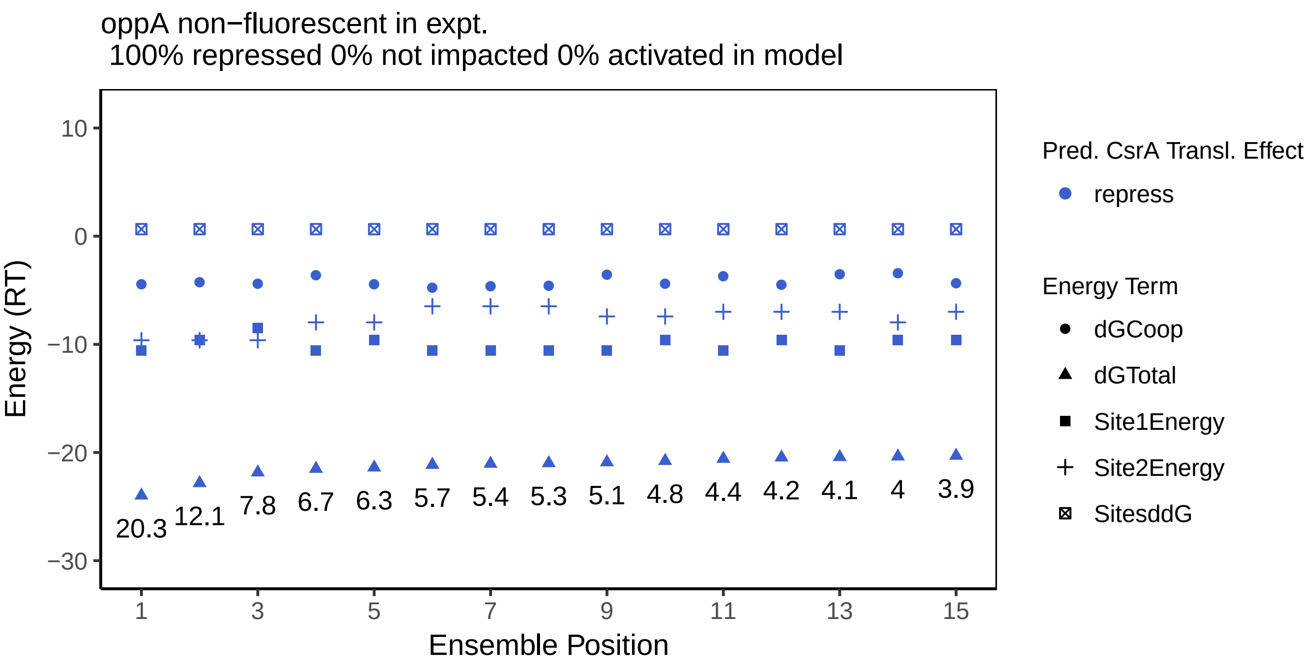

oppA non−fluorescent in expt.
100% repressed 0% not impacted 0% activated in model
Energy (RT)
10
0
−10
−20
−30
20.3
12.1
7.8
6.7
6.3
5.7
5.4
5.3
5.1
4.8
4.4
4.2
4.1
4
3.9
1
3
5
7
9
11
13
15
Ensemble Position
Pred. CsrA Transl. Effect
repress
Energy Term
dGCoop
dGTotal
Site1Energy
Site2Energy
SitesddG

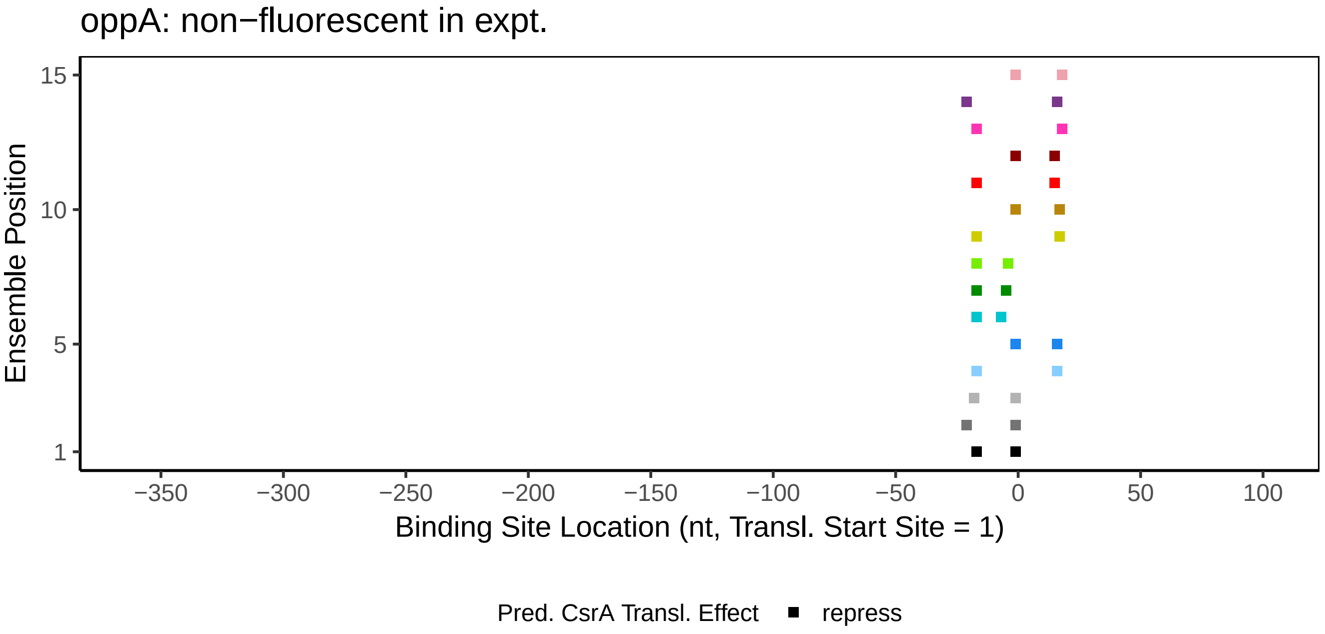

oppA: non−fluorescent in expt.
Ensemble Position
15
10
5
1
−350
−300
−250
−200
−150
−100
−50
0
50
100
Binding Site Location (nt, Transl. Start Site = 1)
Pred. CsrA Transl. Effect
repress

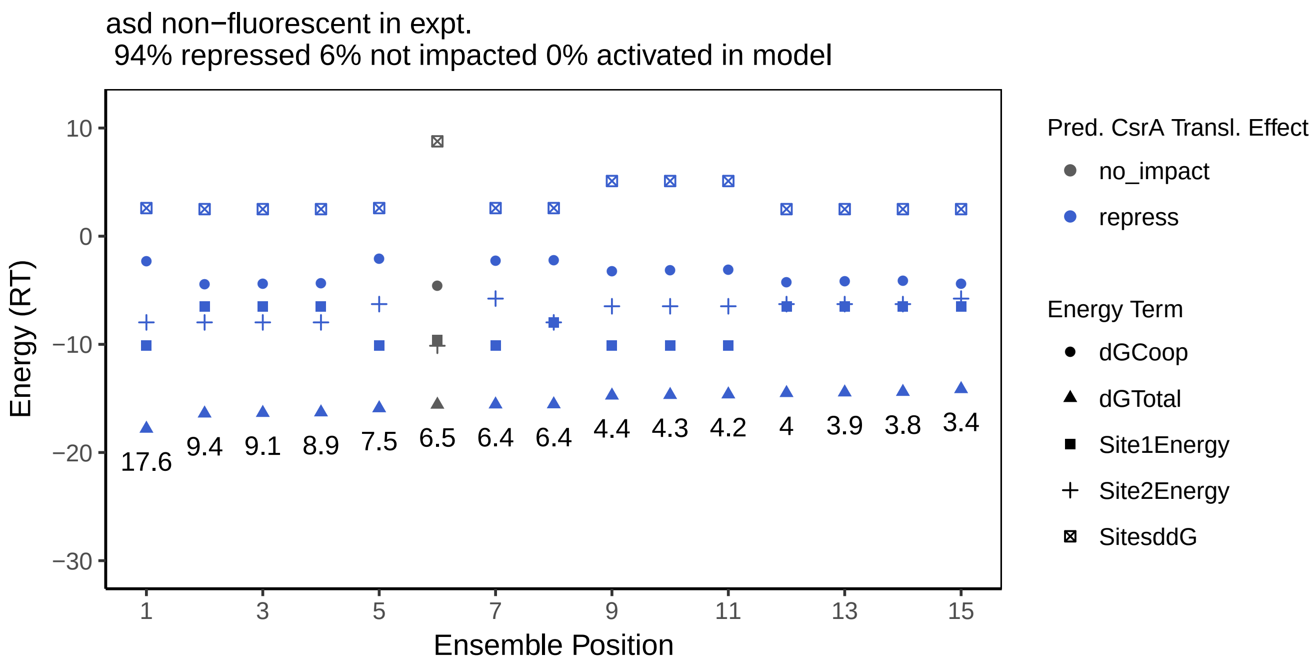

asd non−fluorescent in expt.
94% repressed 6% not impacted 0% activated in model
Energy (RT)
10
0
−10
−20
−30
17.6
9.4
9.1
8.9
7.5
6.5
6.4
6.4
4.4
4.3
4.2
4
3.9
3.8
3.4
1
3
5
7
9
11
13
15
Ensemble Position
Pred. CsrA Transl. Effect
no_impact
repress
Energy Term
dGCoop
dGTotal
Site1Energy
Site2Energy
SitesddG

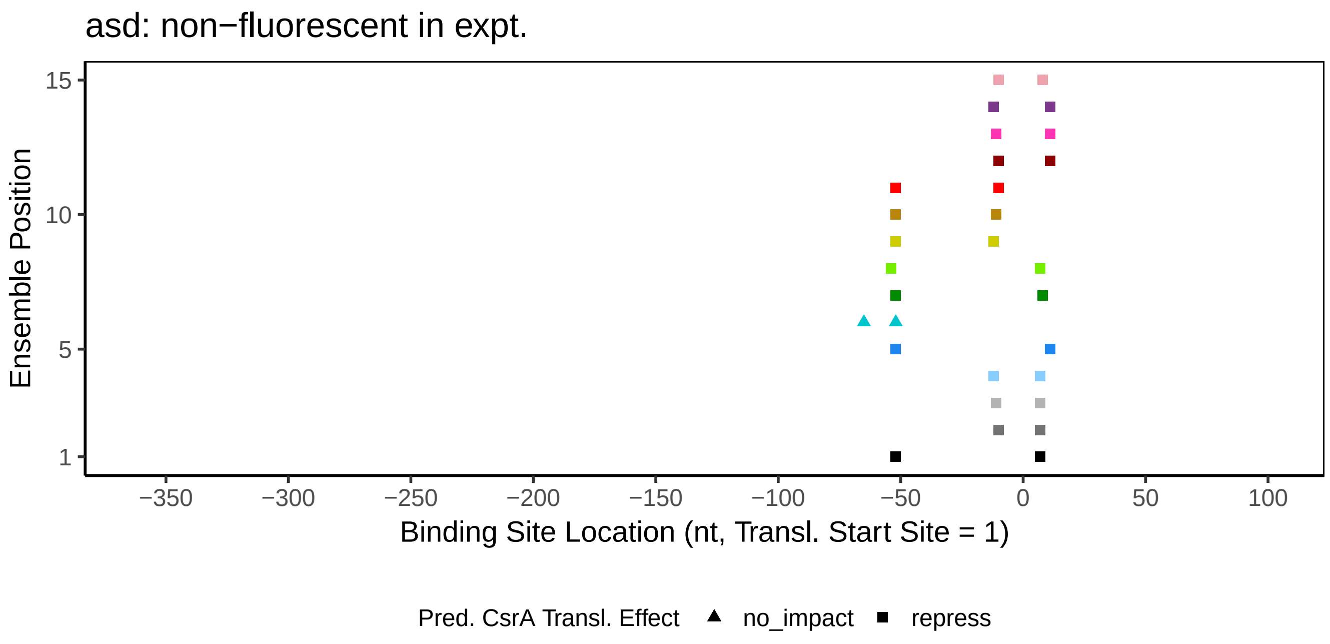

asd: non−fluorescent in expt.
Ensemble Position
15
10
5
1
−350
−300
−250
−200
−150
−100
−50
0
50
100
Binding Site Location (nt, Transl. Start Site = 1)
Pred. CsrA Transl. Effect
no_impact
repress

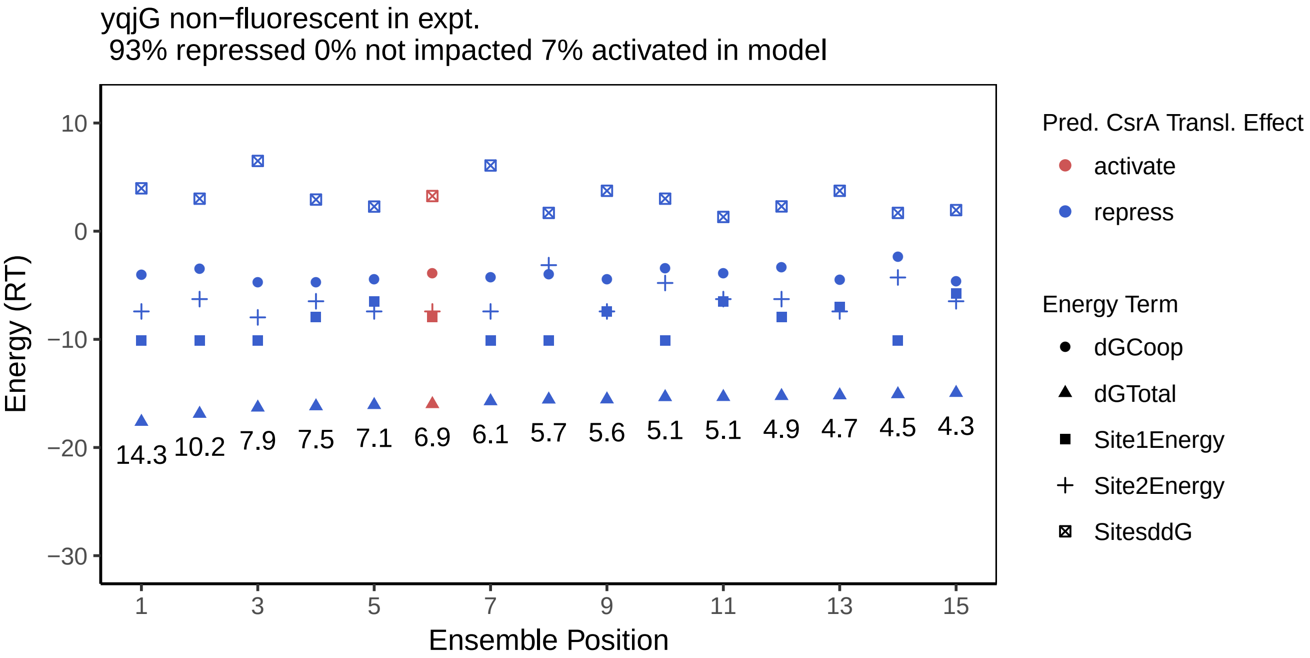

yqjG non−fluorescent in expt.
93% repressed 0% not impacted 7% activated in model
Energy (RT)
Ensemble Position
14.3 10.2 7.9 7.5 7.1 6.9 6.1 5.7 5.6 5.1 5.1 4.9 4.7 4.5 4.3
Pred. CsrA Transl. Effect
activate
repress
Energy Term
dGCoop
dGTotal
Site1Energy
Site2Energy
SitesddG

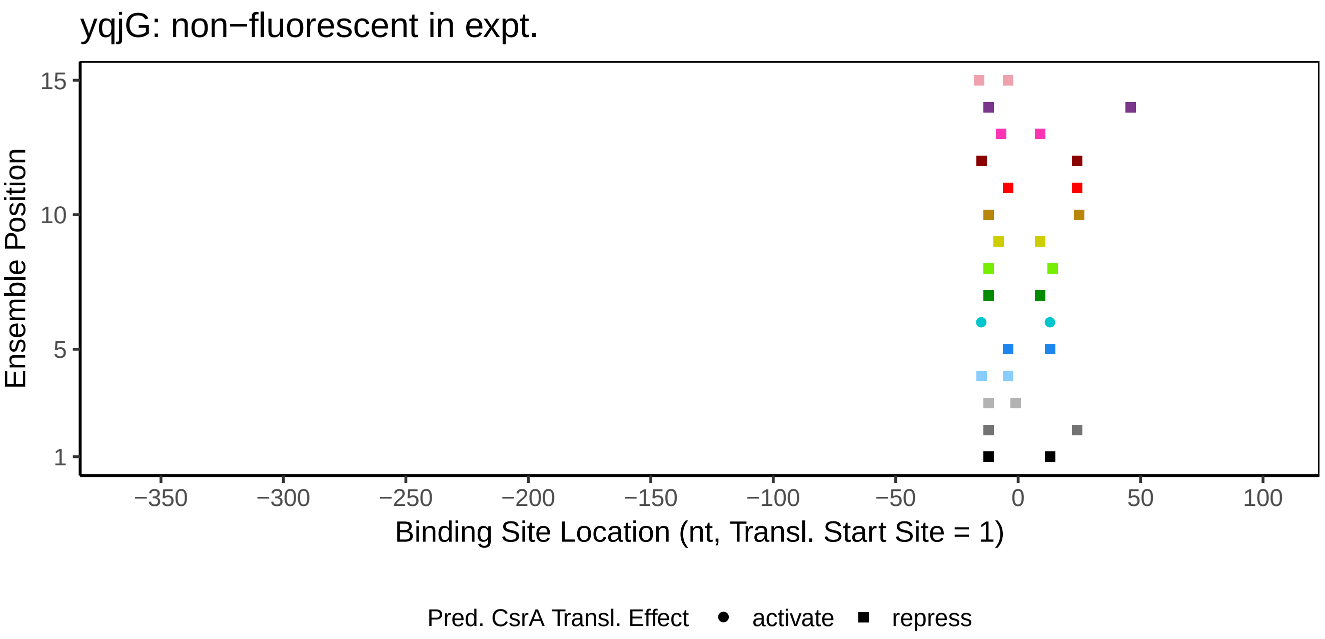

yqjG: non−fluorescent in expt.
Ensemble Position
Binding Site Location (nt, Transl. Start Site = 1)
Pred. CsrA Transl. Effect
activate
repress

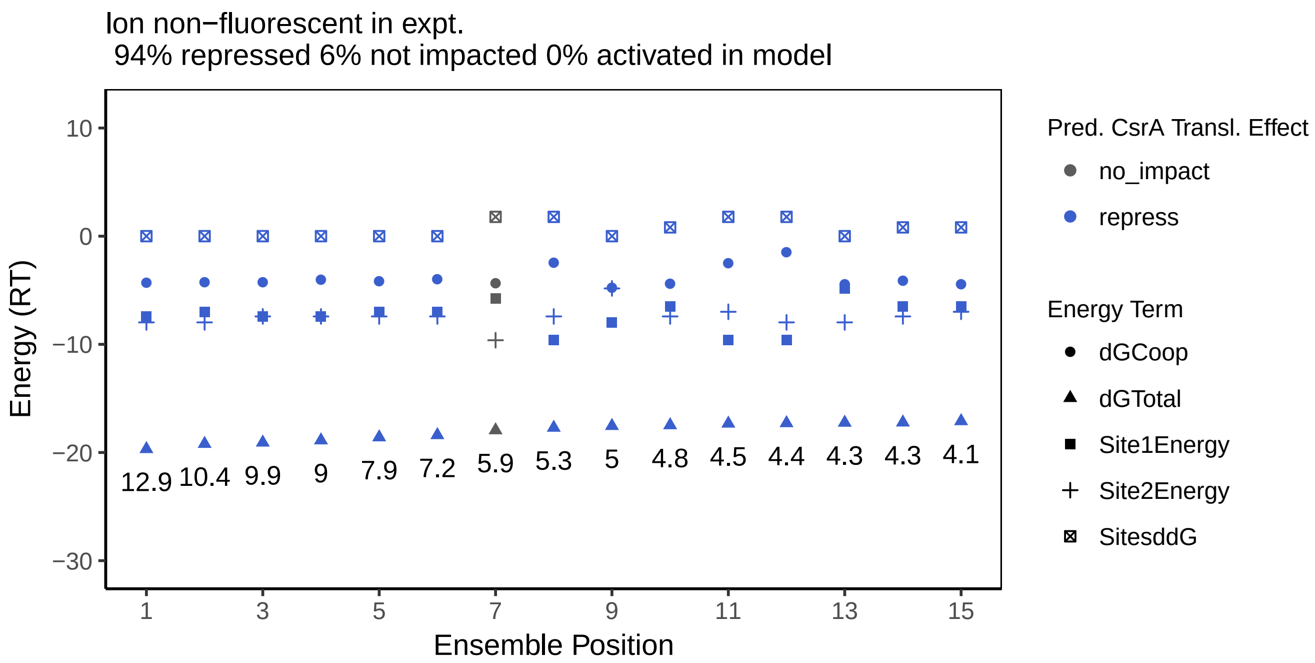

lon non−fluorescent in expt.
94% repressed 6% not impacted 0% activated in model
Energy (RT)
Ensemble Position
12.9 10.4 9.9 9 7.9 7.2 5.9 5.3 5 4.8 4.5 4.4 4.3 4.3 4.1
Pred. CsrA Transl. Effect
no_impact
repress
Energy Term
dGCoop
dGTotal
Site1Energy
Site2Energy
SitesddG

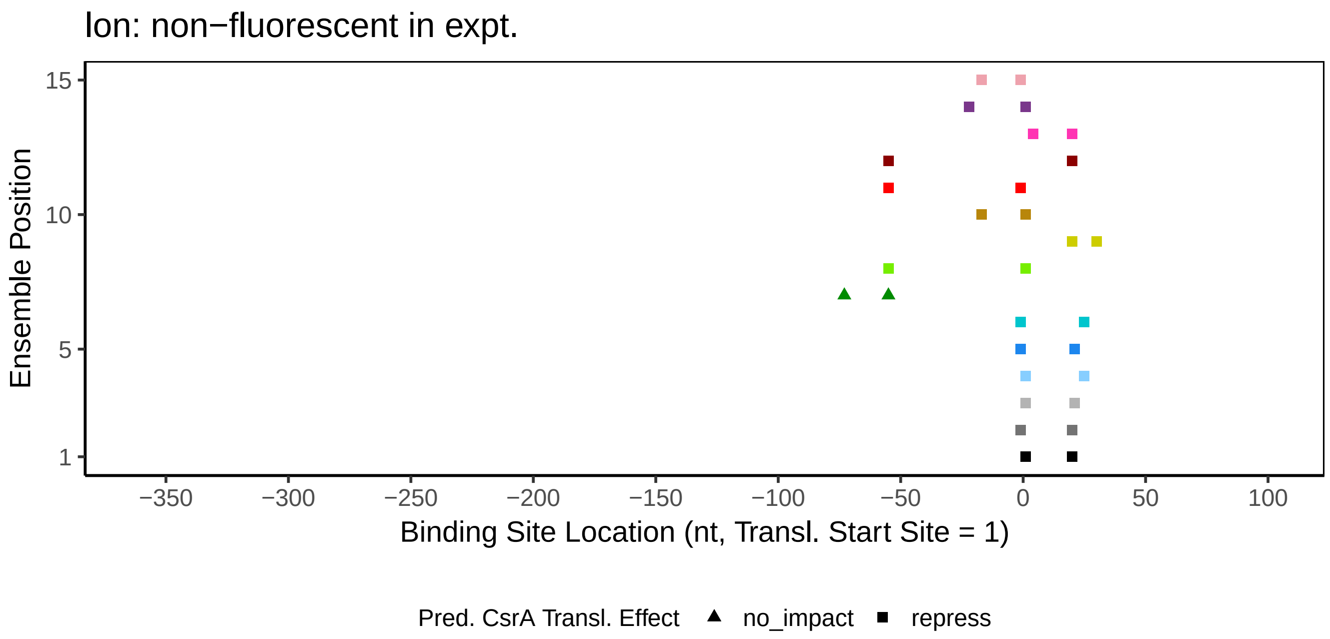

lon: non−fluorescent in expt.
Ensemble Position
Binding Site Location (nt, Transl. Start Site = 1)
Pred. CsrA Transl. Effect no_impact repress

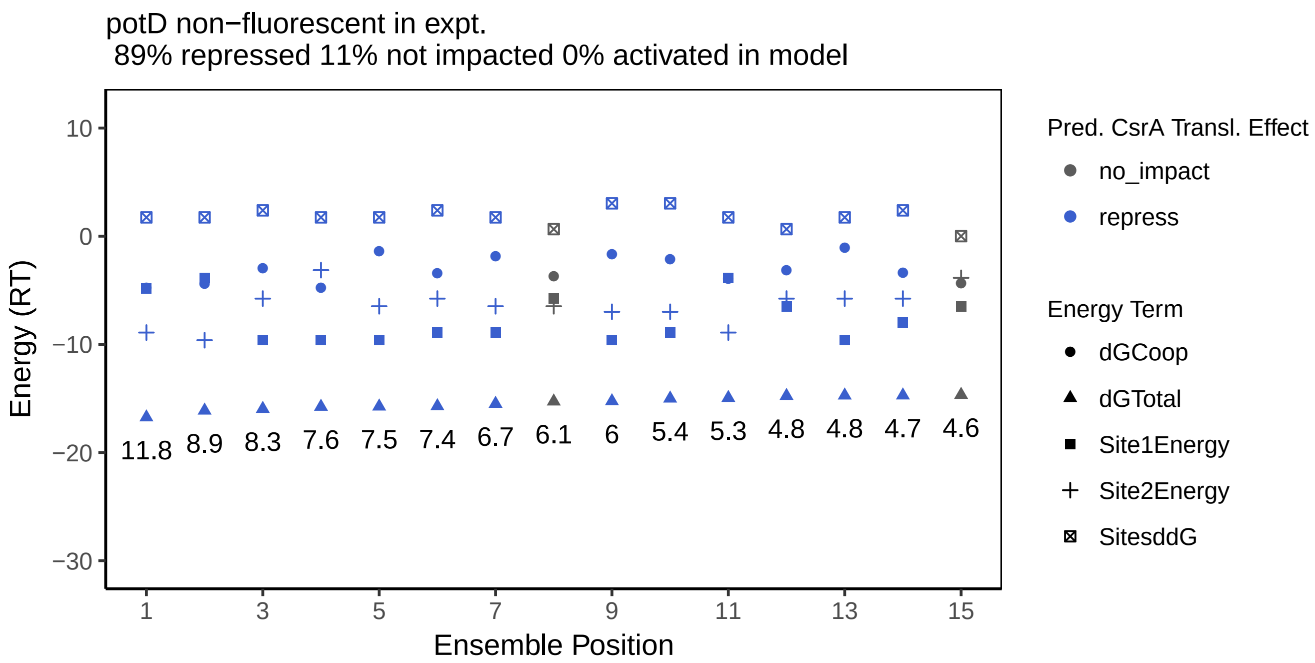
potD non−fluorescent in expt.
89% repressed 11% not impacted 0% activated in model
Energy (RT)
Ensemble Position
11.8
8.9
8.3
7.6
7.5
7.4
6.7
6.1
6
5.4
5.3
4.8
4.8
4.7
4.6
Pred. CsrA Transl. Effect
no_impact
repress
Energy Term
dGCoop
dGTotal
Site1Energy
Site2Energy
SitesddG

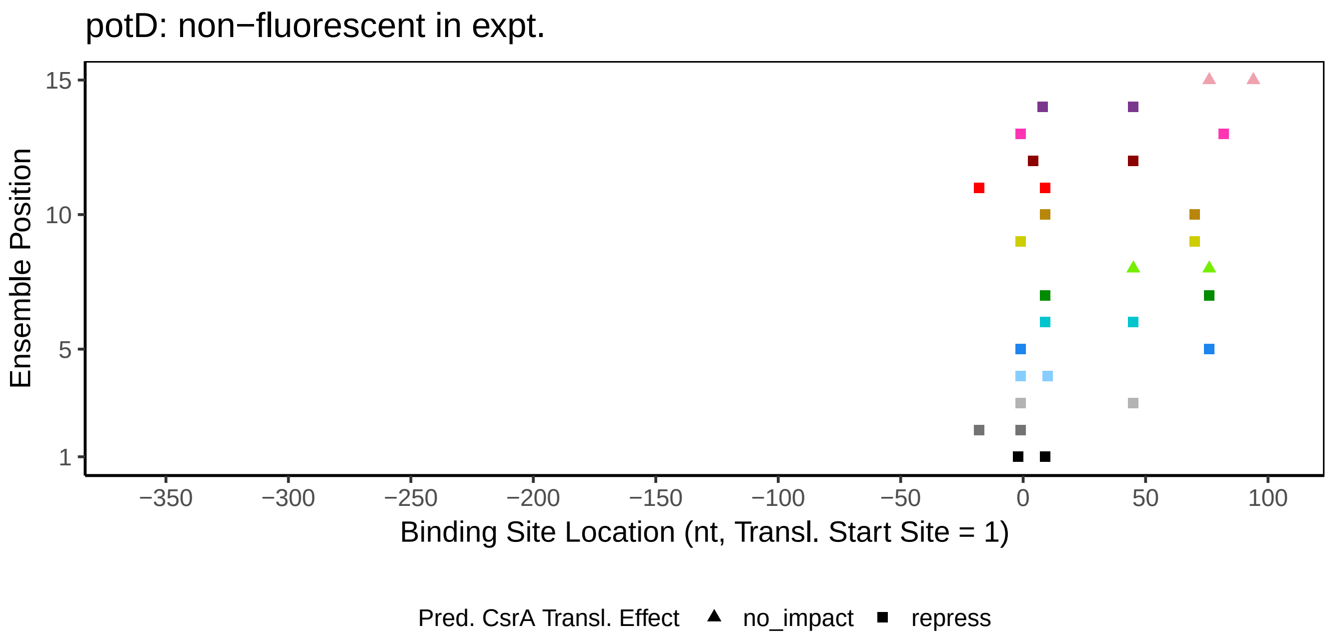
potD: non−fluorescent in expt.
Ensemble Position
Binding Site Location (nt, Transl. Start Site = 1)
Pred. CsrA Transl. Effect
no_impact
repress

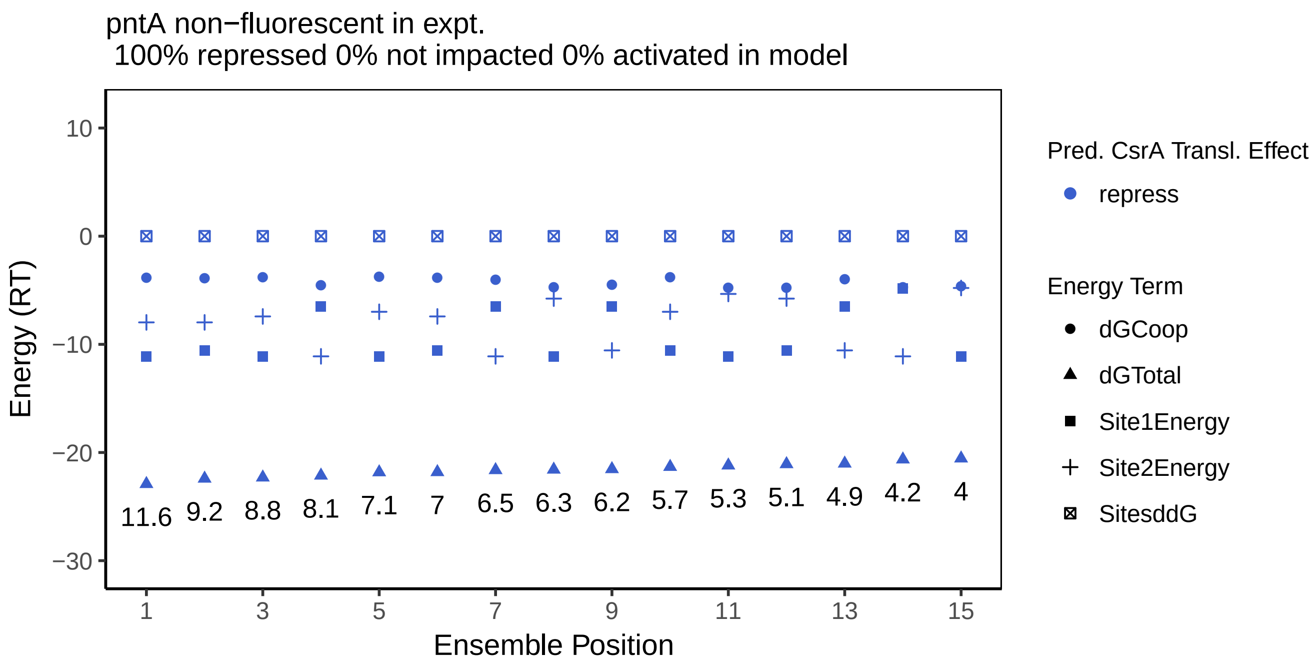

pntA non−fluorescent in expt.
100% repressed 0% not impacted 0% activated in model
Energy (RT)
10
0
−10
−20
−30
11.6
9.2
8.8
8.1
7.1
7
6.5
6.3
6.2
5.7
5.3
5.1
4.9
4.2
4
1
3
5
7
9
11
13
15
Ensemble Position
Pred. CsrA Transl. Effect
repress
Energy Term
dGCoop
dGTotal
Site1Energy
Site2Energy
SitesddG

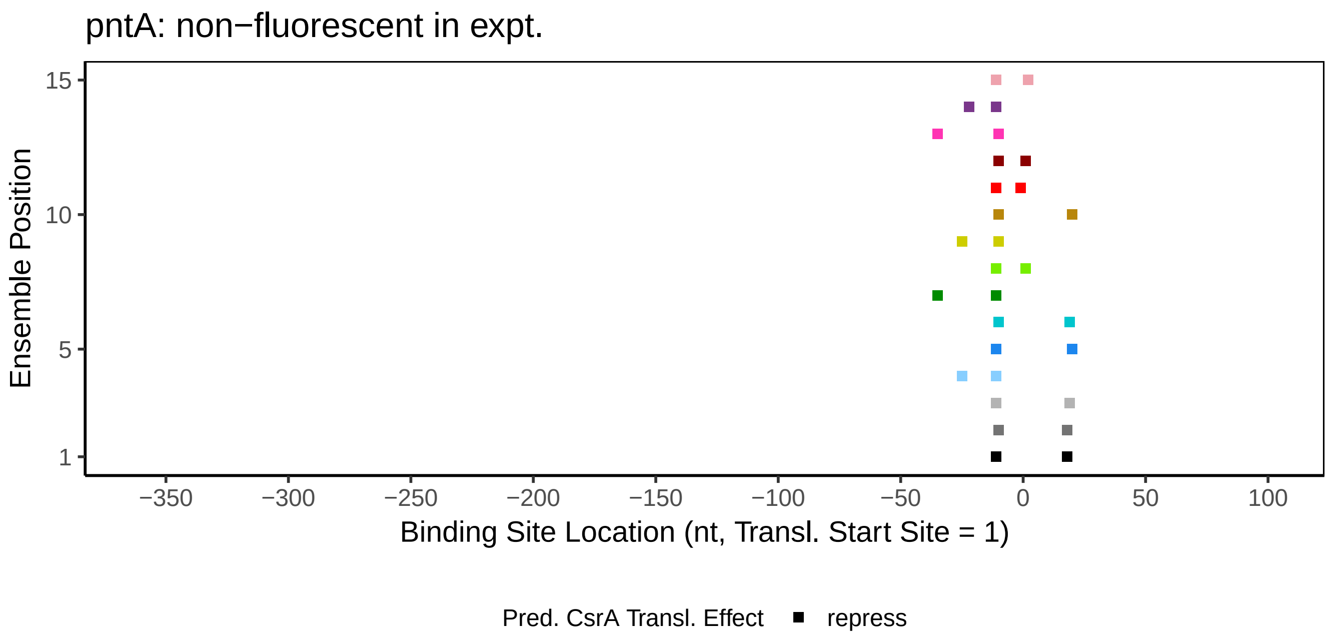

pntA: non−fluorescent in expt.
Ensemble Position
15
10
5
1
−350
−300
−250
−200
−150
−100
−50
0
50
100
Binding Site Location (nt, Transl. Start Site = 1)
Pred. CsrA Transl. Effect
repress

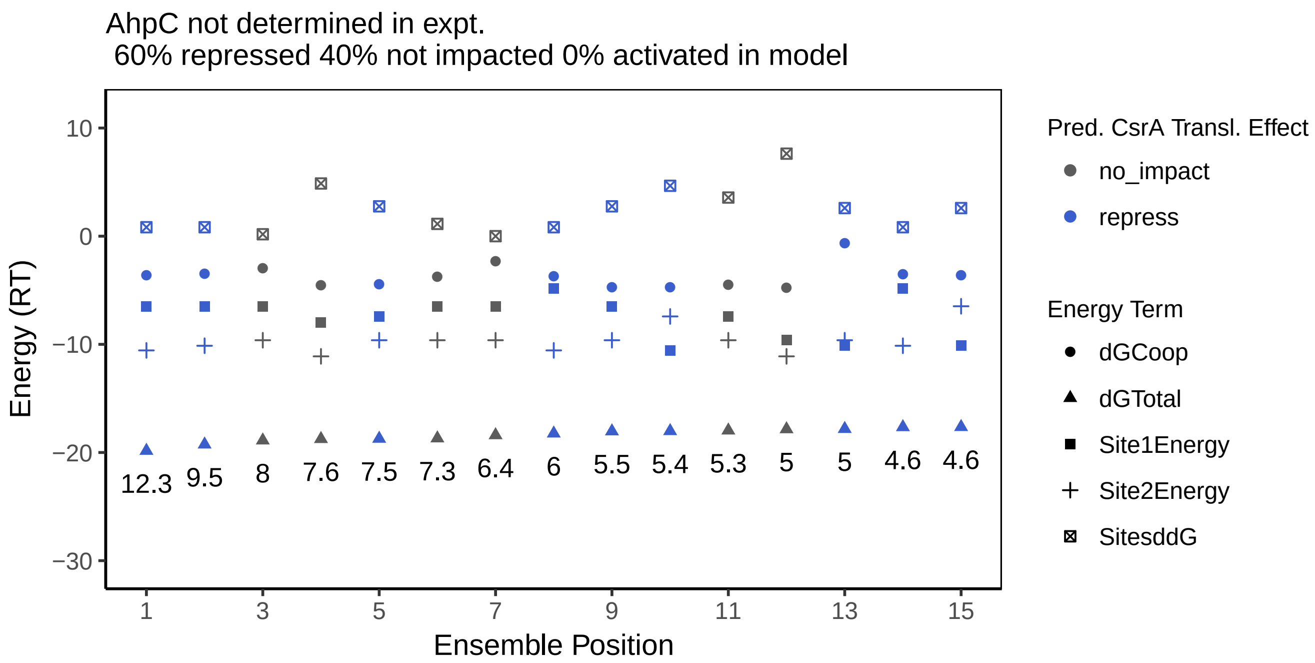
AhpC not determined in expt.
60% repressed 40% not impacted 0% activated in model
Energy (RT)
Ensemble Position
12.3 9.5 8 7.6 7.5 7.3 6.4 6 5.5 5.4 5.3 5 5 4.6 4.6
Pred. CsrA Transl. Effect
no_impact
repress
Energy Term
dGCoop
dGTotal
Site1Energy
Site2Energy
SitesddG

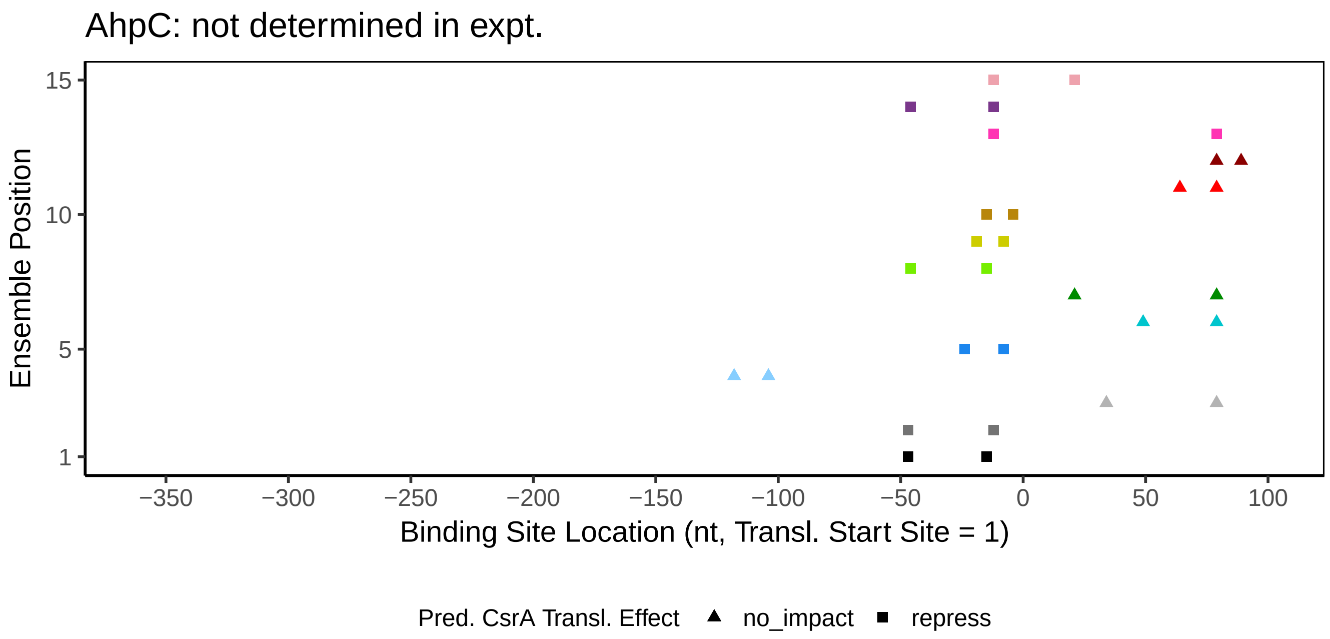
AhpC: not determined in expt.
Ensemble Position
Binding Site Location (nt, Transl. Start Site = 1)
Pred. CsrA Transl. Effect
no_impact
repress

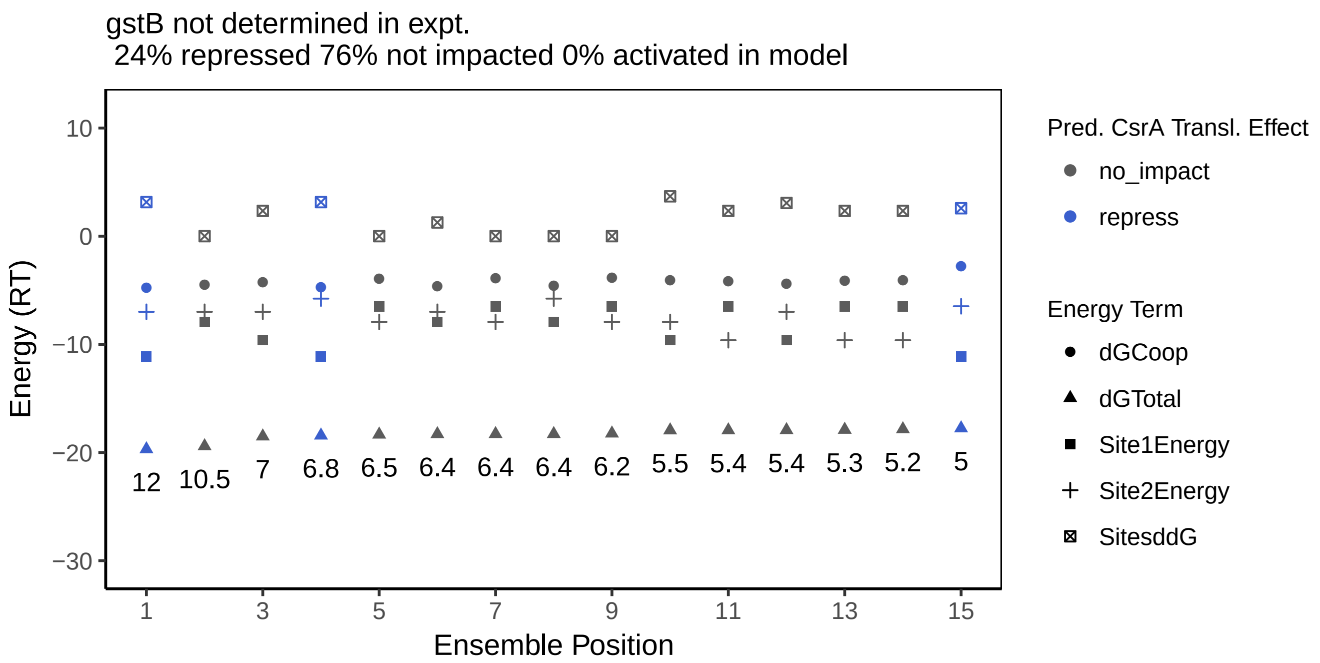
gstB not determined in expt.
24% repressed 76% not impacted 0% activated in model
Energy (RT)
Ensemble Position
12
10.5
7
6.8
6.5
6.4
6.4
6.4
6.2
5.5
5.4
5.4
5.3
5.2
5
Pred. CsrA Transl. Effect
no_impact
repress
Energy Term
dGCoop
dGTotal
Site1Energy
Site2Energy
SitesddG

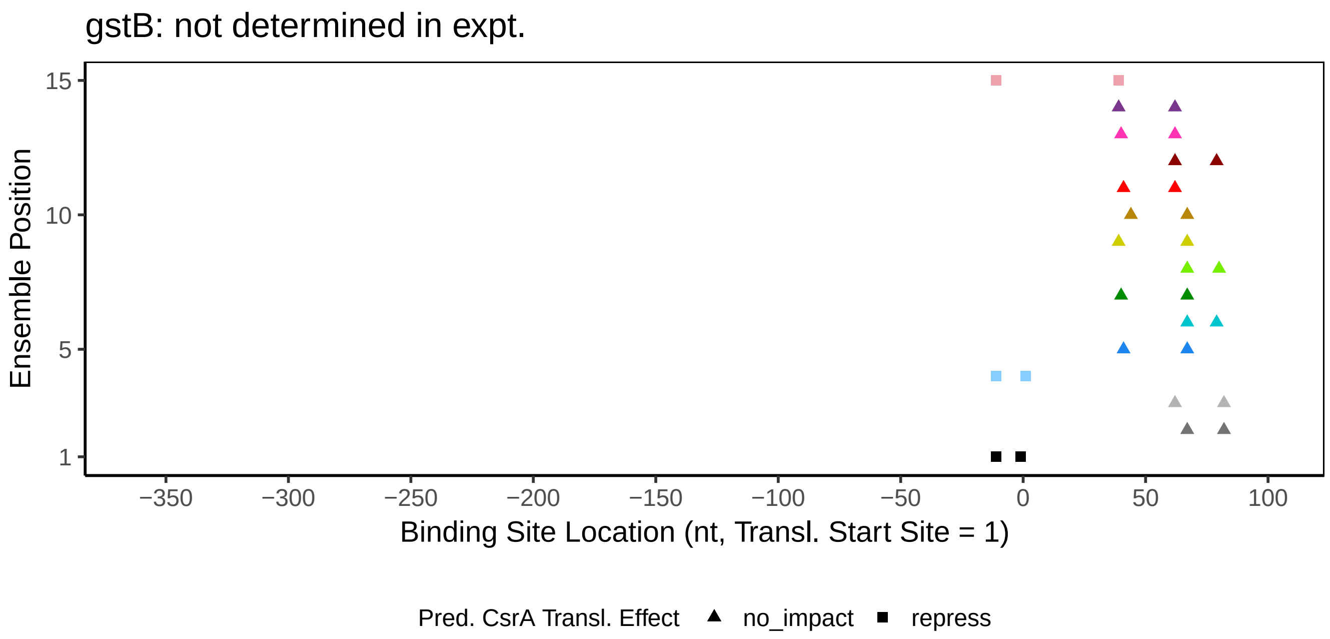
gstB: not determined in expt.
Ensemble Position
Binding Site Location (nt, Transl. Start Site = 1)
Pred. CsrA Transl. Effect
no_impact
repress

ackA not determined in expt.
100% repressed 0% not impacted 0% activated in model

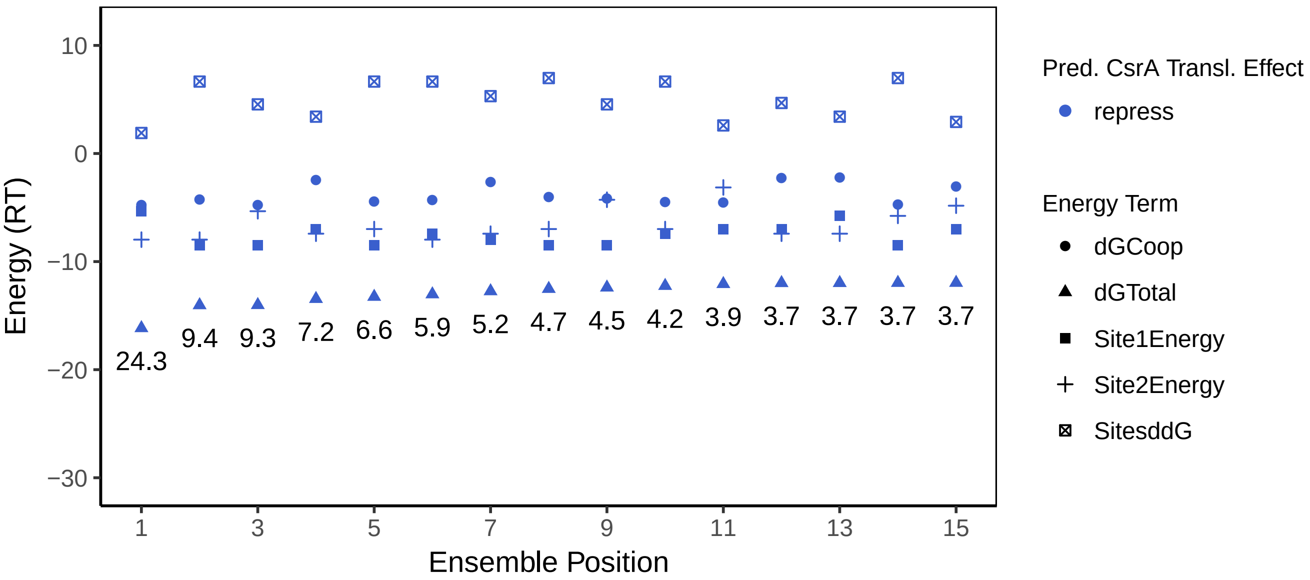

ackA: not determined in expt.

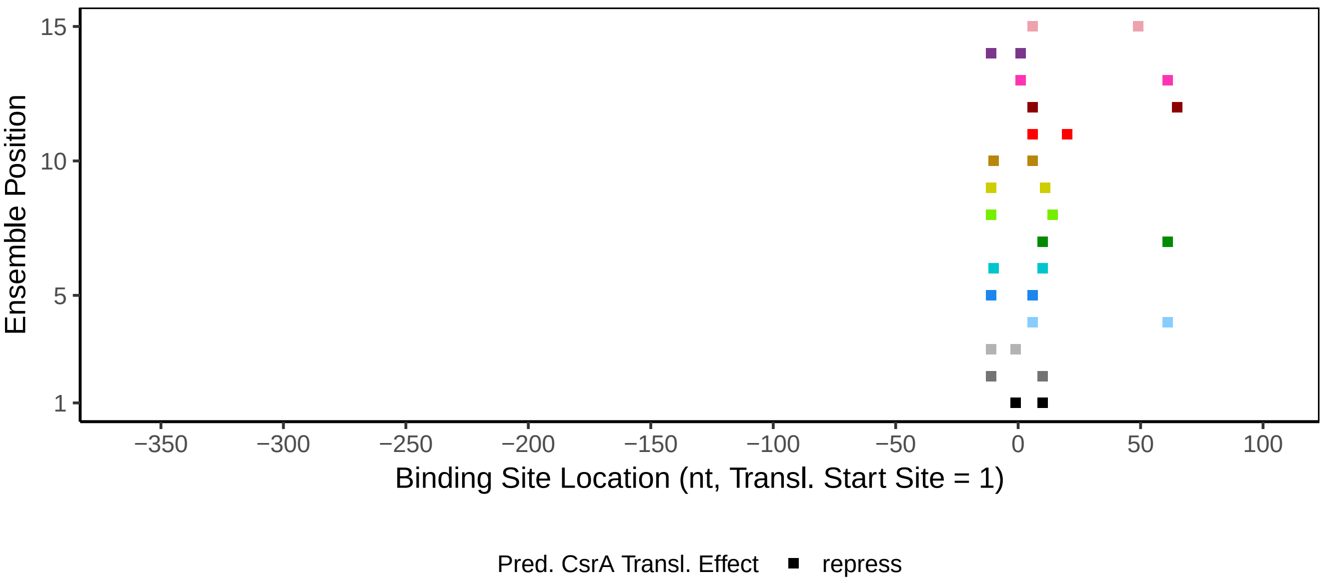

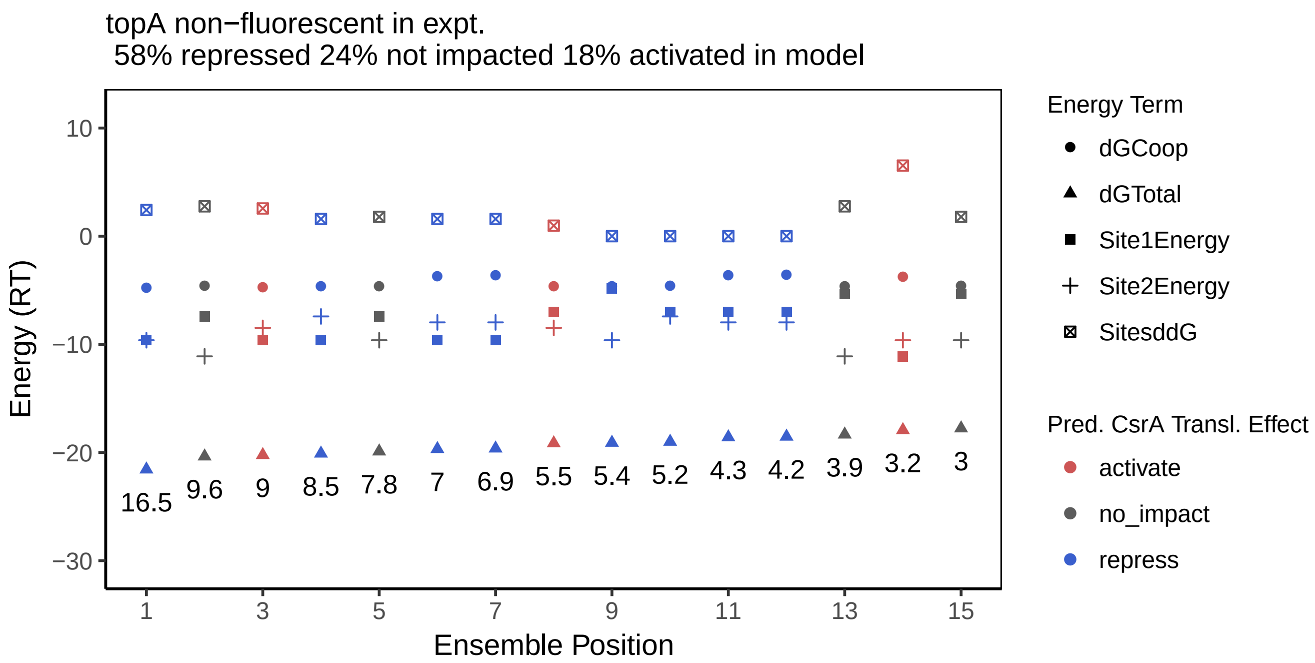
topA non−fluorescent in expt.
58% repressed 24% not impacted 18% activated in model
Energy (RT)
10
0
−10
−20
−30
16.5
9.6
9
8.5
7.8
7
6.9
5.5
5.4
5.2
4.3
4.2
3.9
3.2
3
1
3
5
7
9
11
13
15
Ensemble Position
Energy Term
dGCoop
dGTotal
Site1Energy
Site2Energy
SitesddG
Pred. CsrA Transl. Effect
activate
no_impact
repress

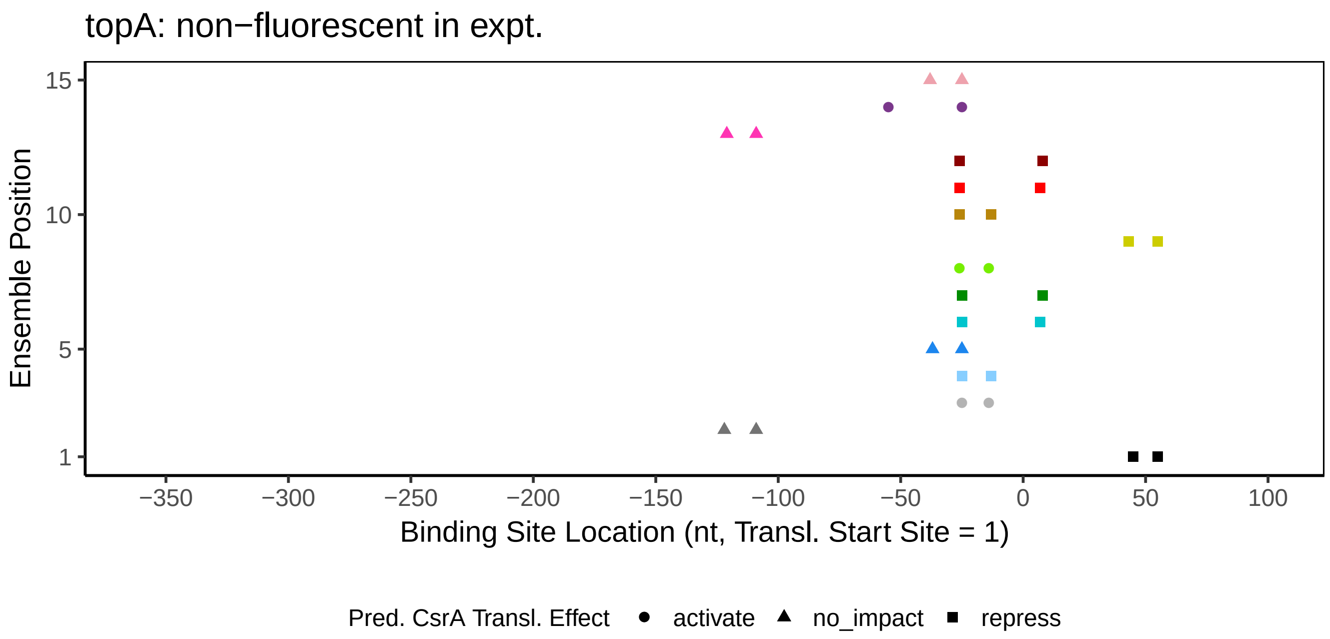
topA: non−fluorescent in expt.
Ensemble Position
15
10
5
1
−350
−300
−250
−200
−150
−100
−50
0
50
100
Binding Site Location (nt, Transl. Start Site = 1)
Pred. CsrA Transl. Effect
activate
no_impact
repress

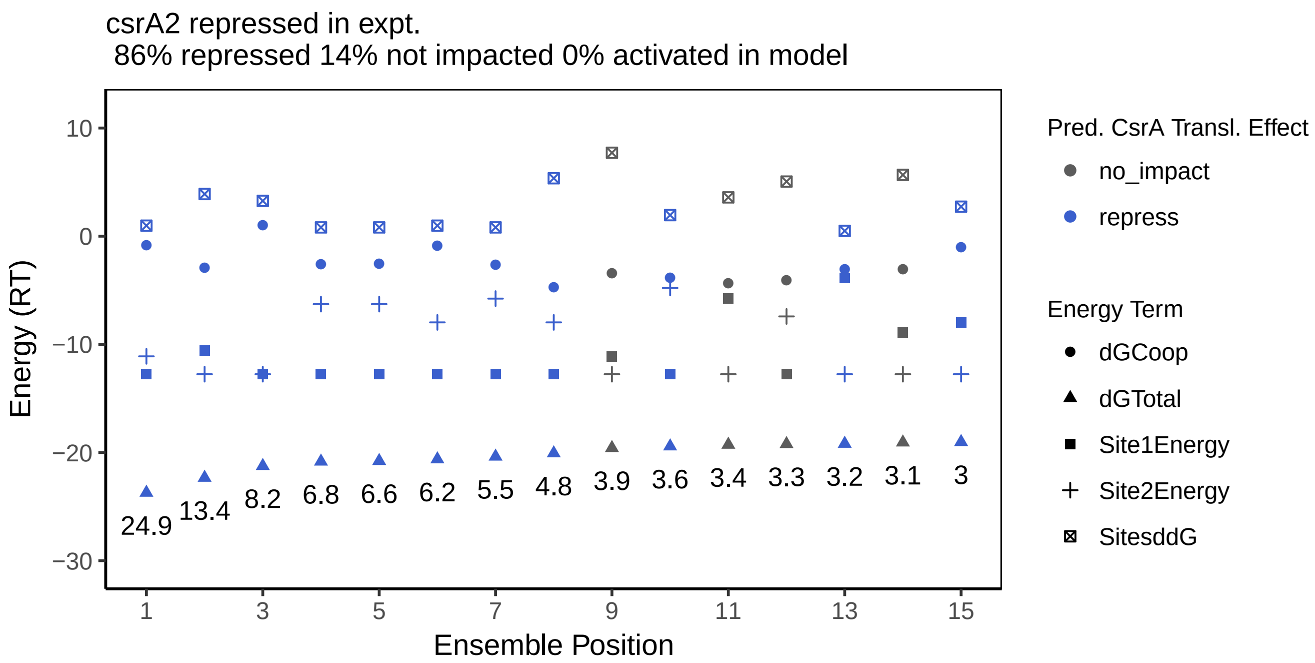

csrA2 repressed in expt.
86% repressed 14% not impacted 0% activated in model
Energy (RT)
10
0
−10
−20
−30
24.9
13.4
8.2
6.8
6.6
6.2
5.5
4.8
3.9
3.6
3.4
3.3
3.2
3.1
3
1
3
5
7
9
11
13
15
Ensemble Position
Pred. CsrA Transl. Effect
no_impact
repress
Energy Term
dGCoop
dGTotal
Site1Energy
Site2Energy
SitesddG

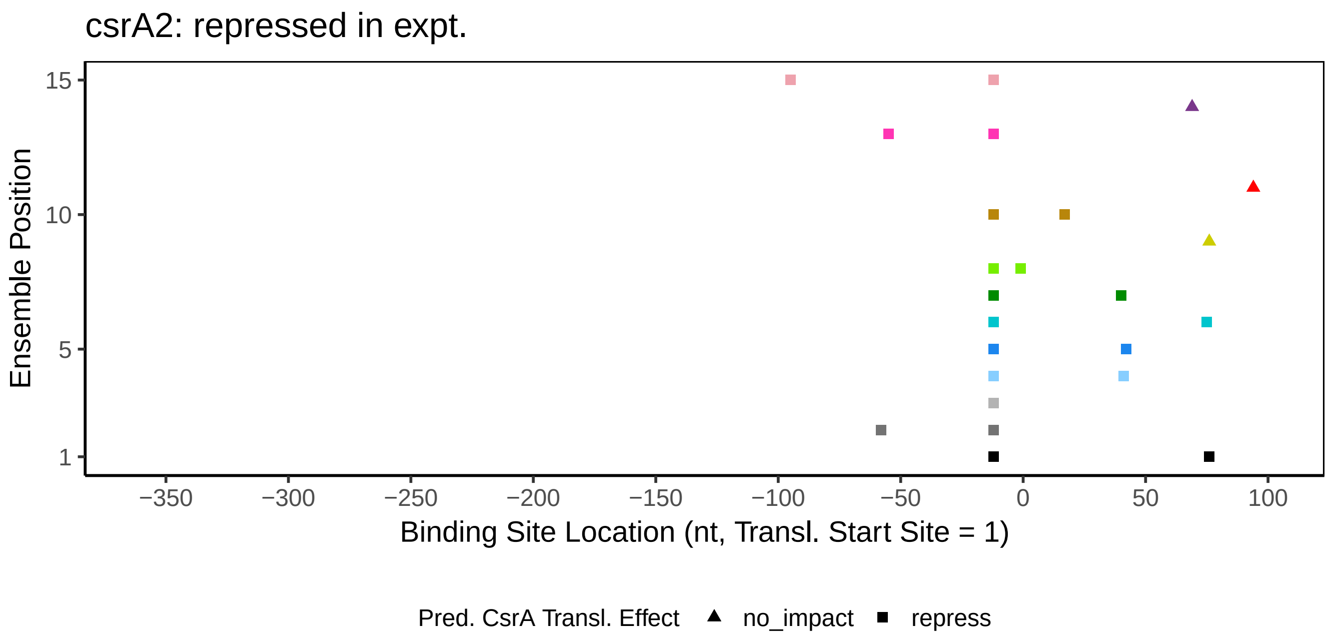

csrA2: repressed in expt.
Ensemble Position
15
10
5
1
−350
−300
−250
−200
−150
−100
−50
0
50
100
Binding Site Location (nt, Transl. Start Site = 1)
Pred. CsrA Transl. Effect
no_impact
repress

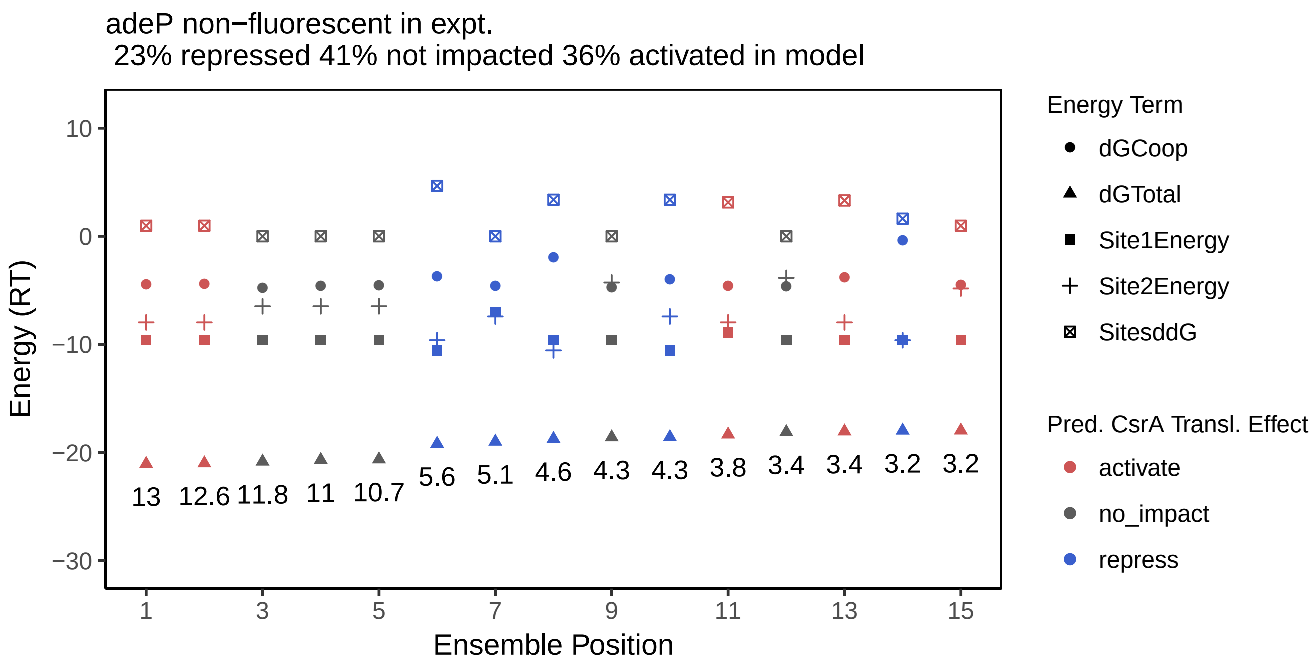

adeP non−fluorescent in expt.
23% repressed 41% not impacted 36% activated in model
Energy (RT)
10
0
−10
−20
−30
13
12.6
11.8
11
10.7
5.6
5.1
4.6
4.3
4.3
3.8
3.4
3.4
3.2
3.2
1
3
5
7
9
11
13
15
Ensemble Position
Energy Term
dGCoop
dGTotal
Site1Energy
Site2Energy
SitesddG
Pred. CsrA Transl. Effect
activate
no_impact
repress

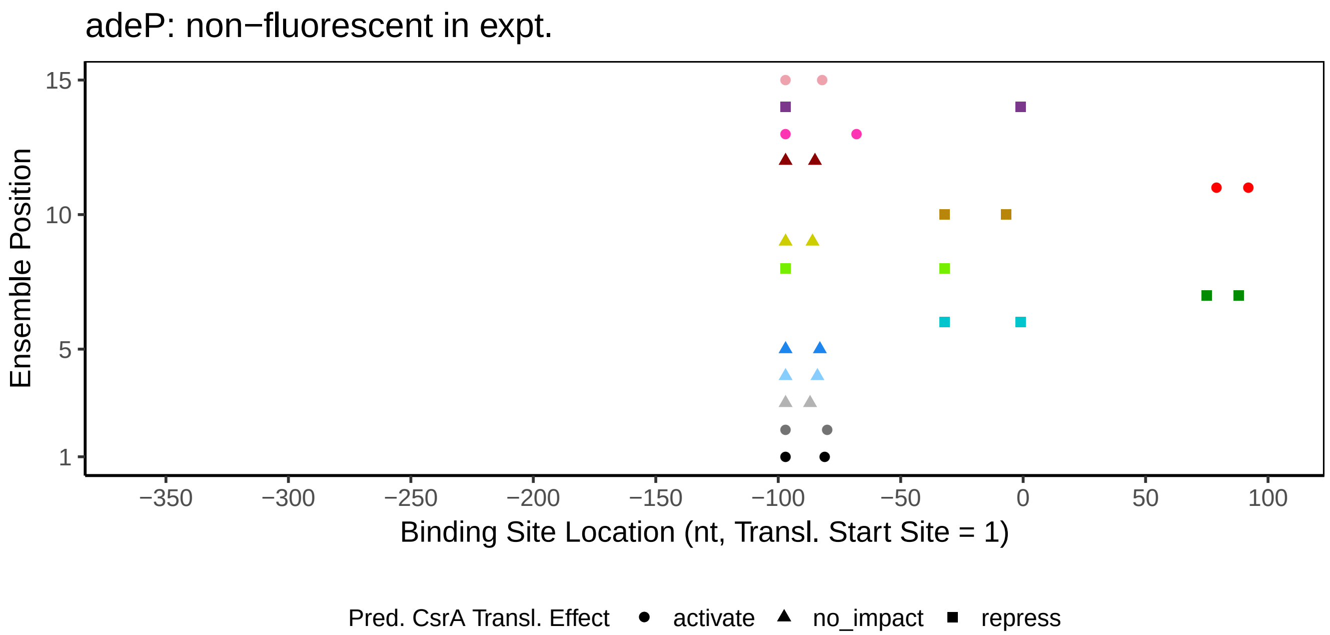

adeP: non−fluorescent in expt.
Ensemble Position
15
10
5
1
−350
−300
−250
−200
−150
−100
−50
0
50
100
Binding Site Location (nt, Transl. Start Site = 1)
Pred. CsrA Transl. Effect
activate
no_impact
repress

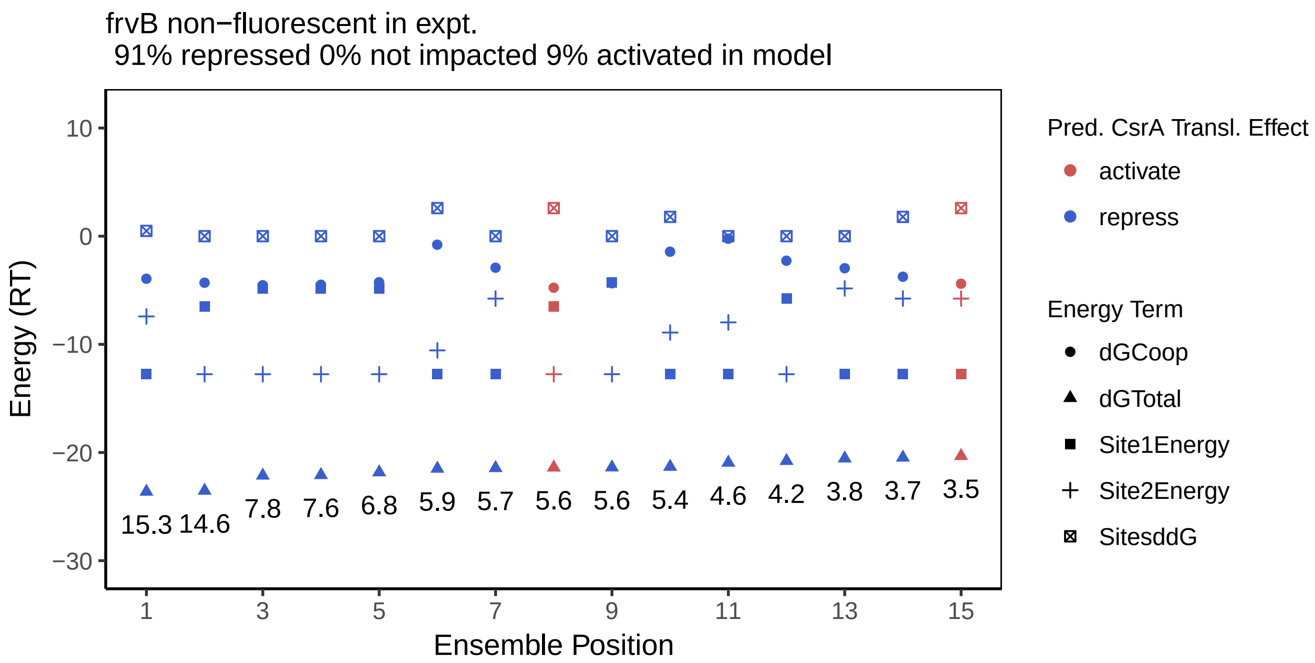

frvB non−fluorescent in expt.
91% repressed 0% not impacted 9% activated in model
Energy (RT)
10
0
−10
−20
−30
15.3 14.6 7.8 7.6 6.8 5.9 5.7 5.6 5.6 5.4 4.6 4.2 3.8 3.7 3.5
1
3
5
7
9
11
13
15
Ensemble Position
Pred. CsrA Transl. Effect
activate
repress
Energy Term
dGCoop
dGTotal
Site1Energy
Site2Energy
SitesddG

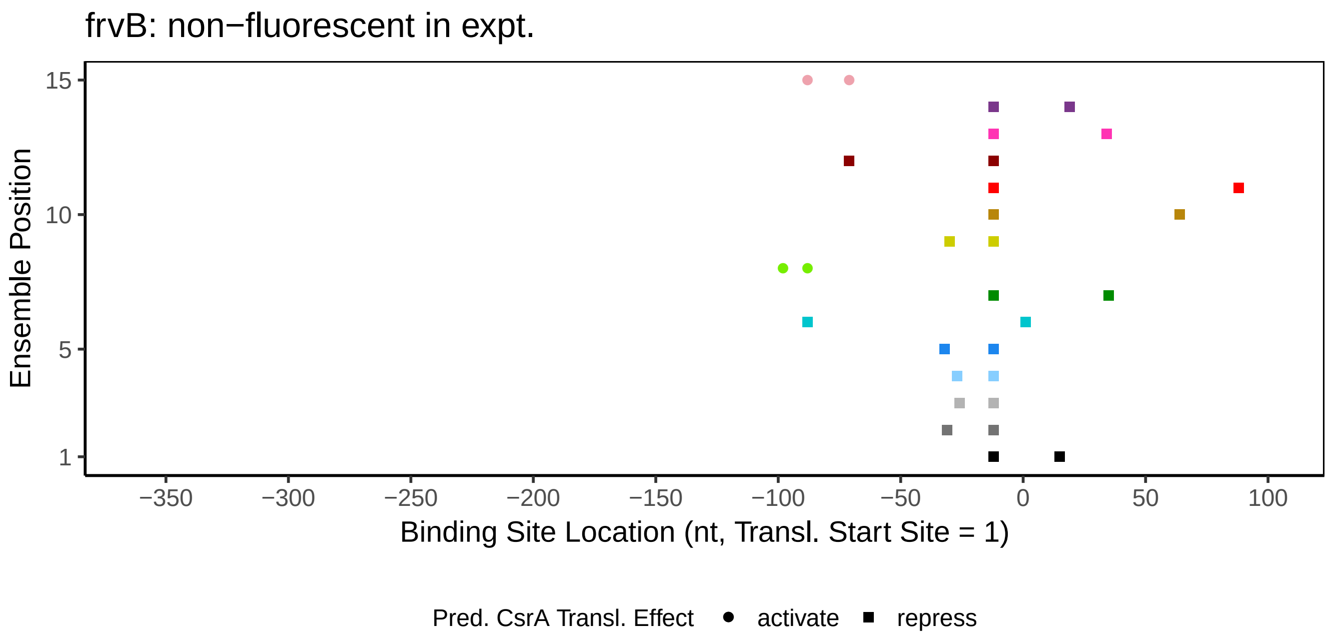

frvB: non−fluorescent in expt.
Ensemble Position
15
10
5
1
−350
−300
−250
−200
−150
−100
−50
0
50
100
Binding Site Location (nt, Transl. Start Site = 1)
Pred. CsrA Transl. Effect
activate
repress

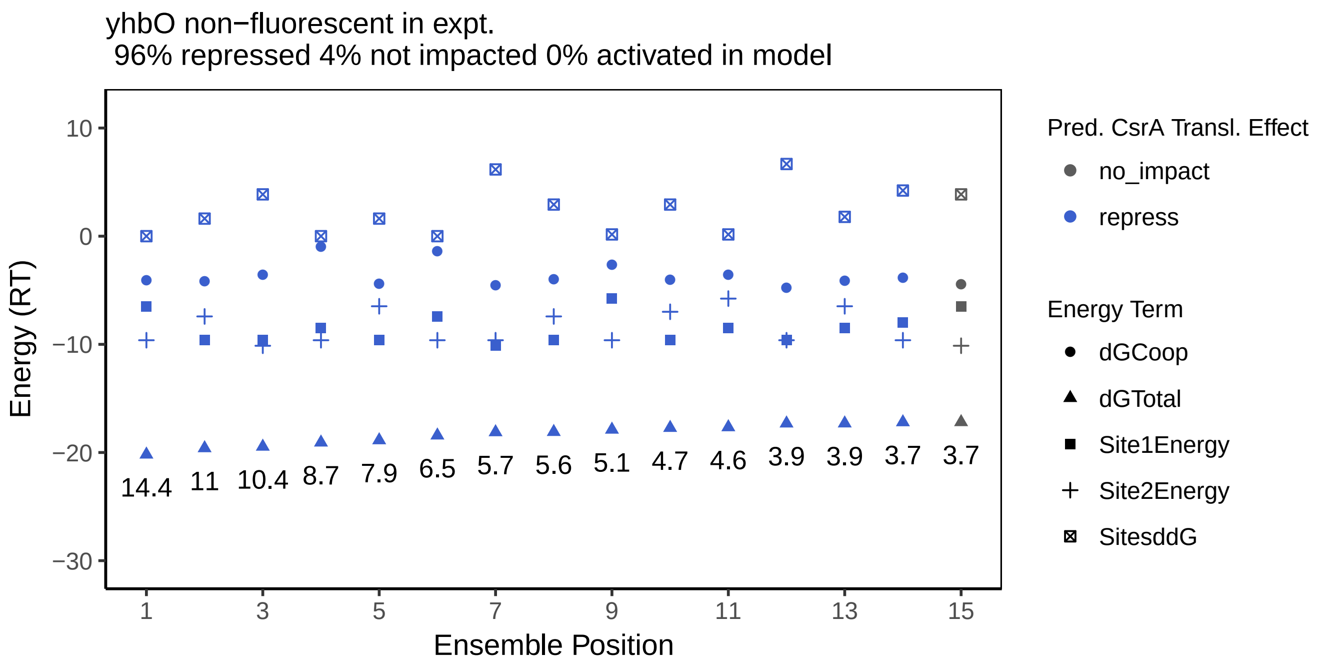
yhbO non−fluorescent in expt.
96% repressed 4% not impacted 0% activated in model
Energy (RT)
Ensemble Position
14.4 11 10.4 8.7 7.9 6.5 5.7 5.6 5.1 4.7 4.6 3.9 3.9 3.7 3.7
Pred. CsrA Transl. Effect
no_impact
repress
Energy Term
dGCoop
dGTotal
Site1Energy
Site2Energy
SitesddG

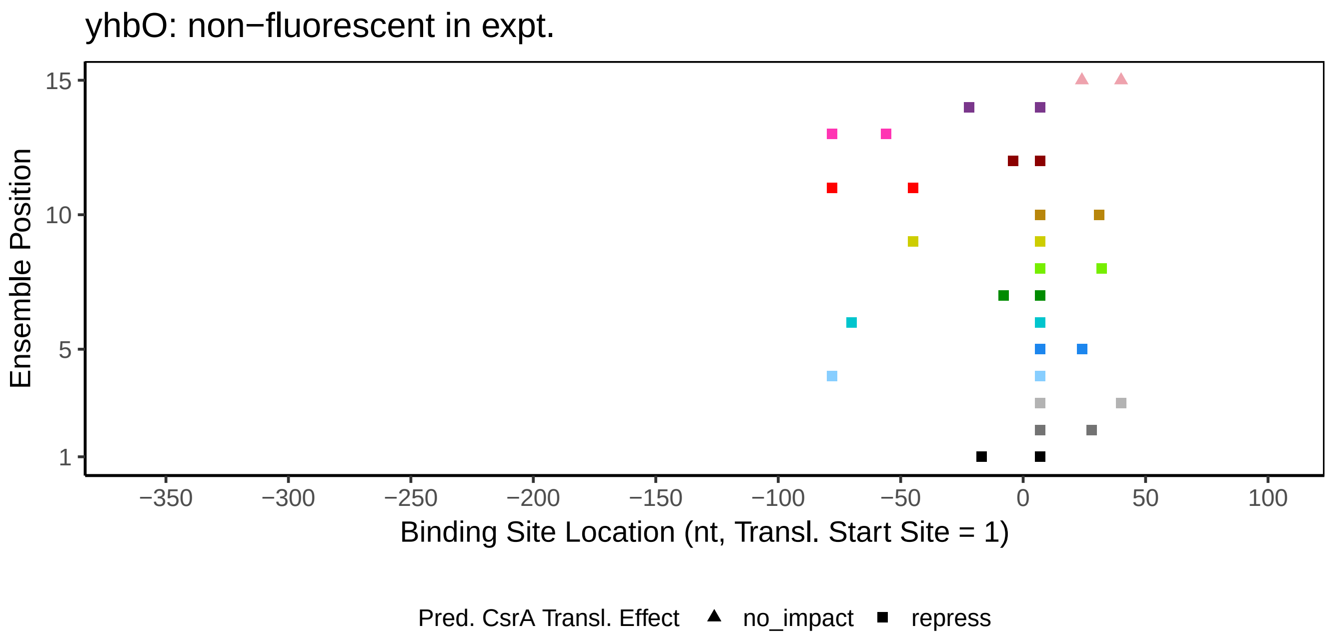
yhbO: non−fluorescent in expt.
Ensemble Position
Binding Site Location (nt, Transl. Start Site = 1)
Pred. CsrA Transl. Effect
no_impact
repress

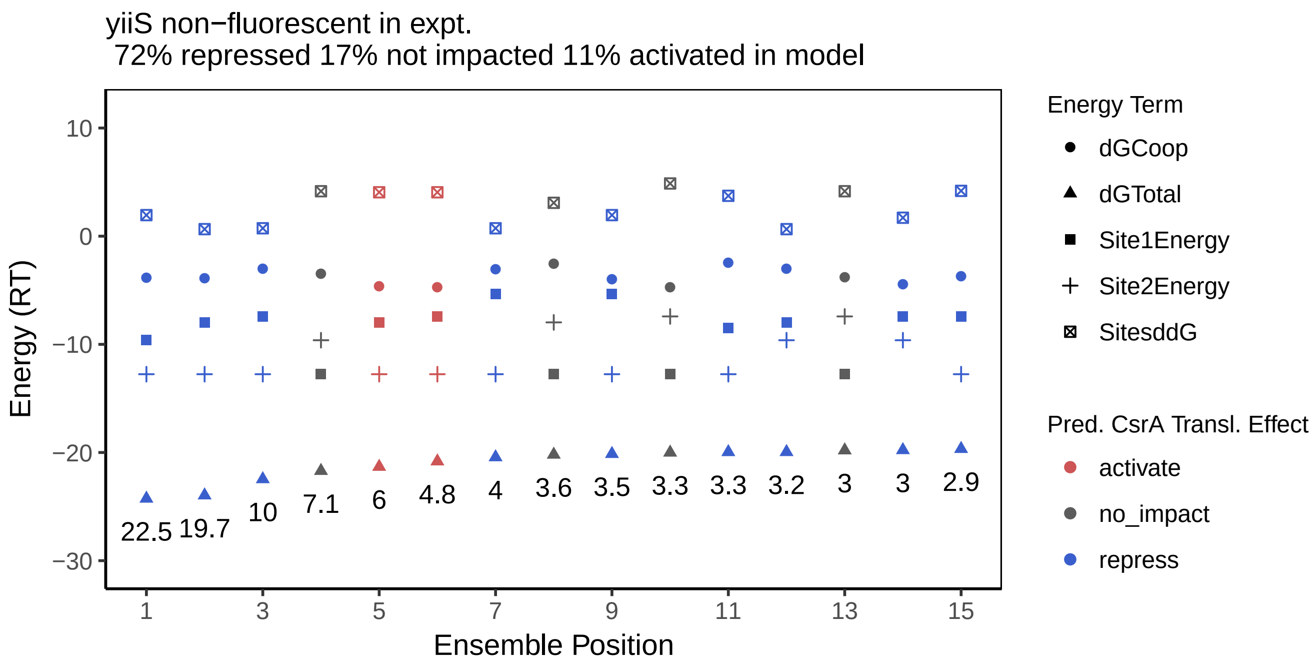

yiiS non−fluorescent in expt.
72% repressed 17% not impacted 11% activated in model
Energy (RT)
10
0
−10
−20
−30
22.5
19.7
10
7.1
6
4.8
4
3.6
3.5
3.3
3.3
3.2
3
3
2.9
1
3
5
7
9
11
13
15
Ensemble Position
Energy Term
dGCoop
dGTotal
Site1Energy
Site2Energy
SitesddG
Pred. CsrA Transl. Effect
activate
no_impact
repress

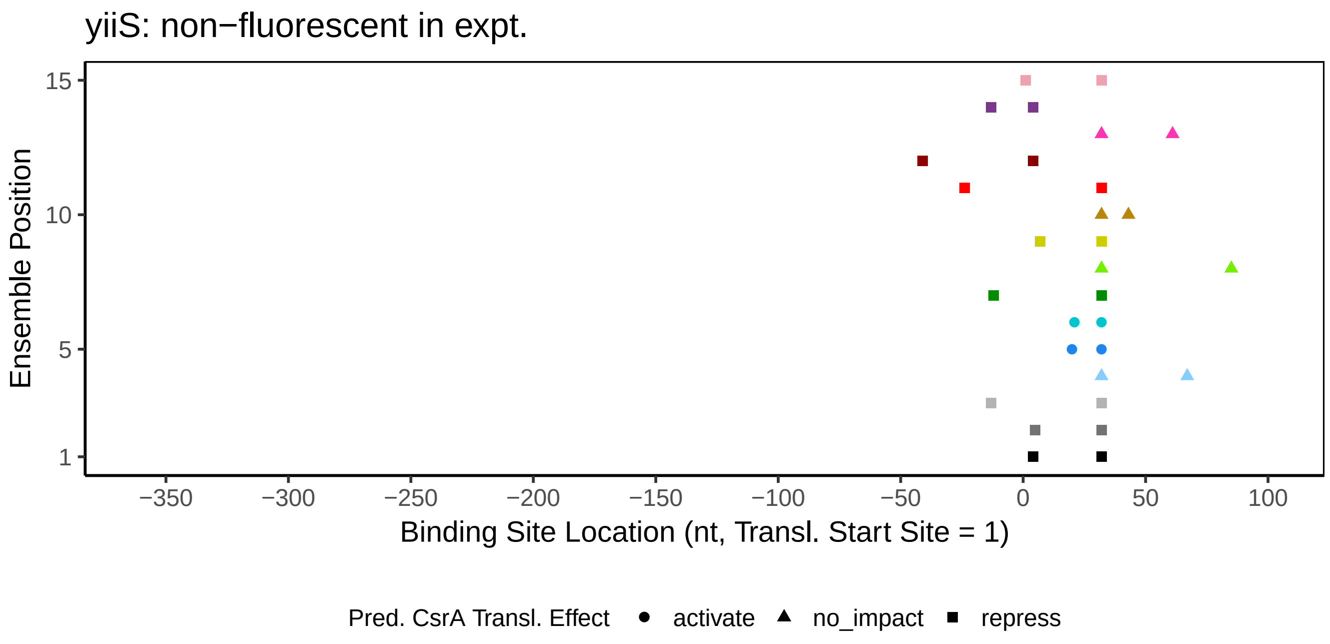

yiiS: non−fluorescent in expt.
Ensemble Position
15
10
5
1
−350
−300
−250
−200
−150
−100
−50
0
50
100
Binding Site Location (nt, Transl. Start Site = 1)
Pred. CsrA Transl. Effect
activate
no_impact
repress

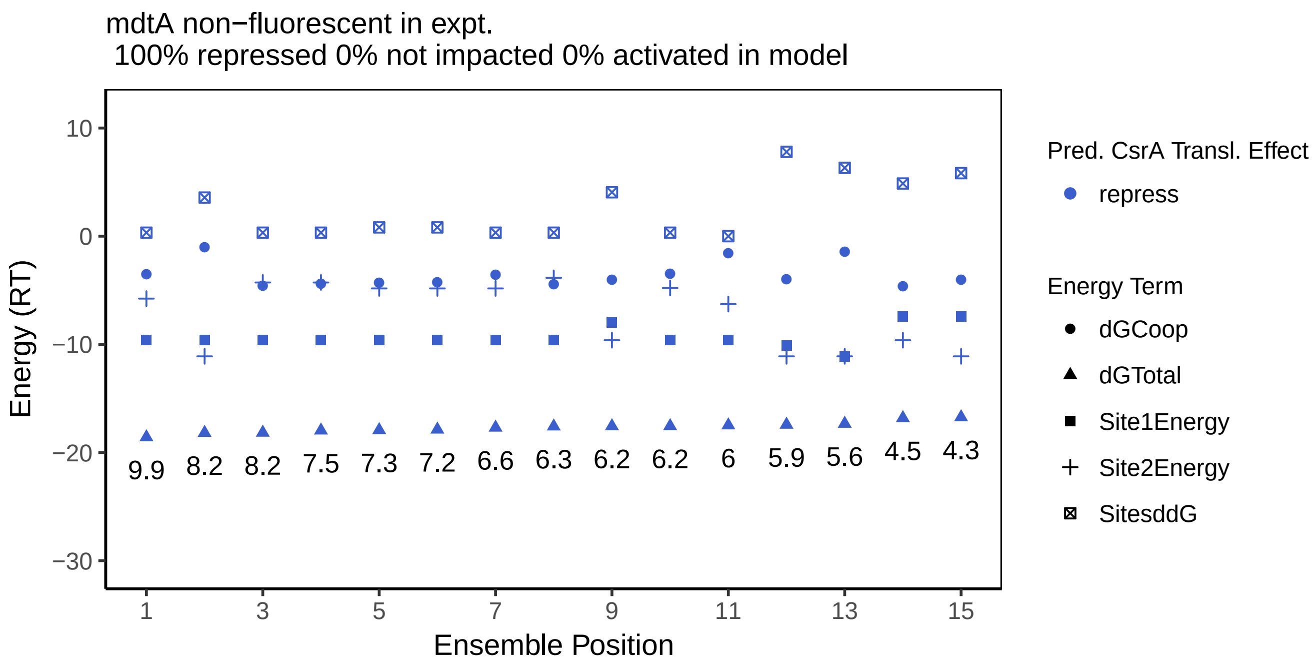

mdtA non−fluorescent in expt.
100% repressed 0% not impacted 0% activated in model
Energy (RT)
10
0
−10
−20
−30
9.9
8.2
8.2
7.5
7.3
7.2
6.6
6.3
6.2
6.2
6
5.9
5.6
4.5
4.3
1
3
5
7
9
11
13
15
Ensemble Position
Pred. CsrA Transl. Effect
repress
Energy Term
dGCoop
dGTotal
Site1Energy
Site2Energy
SitesddG

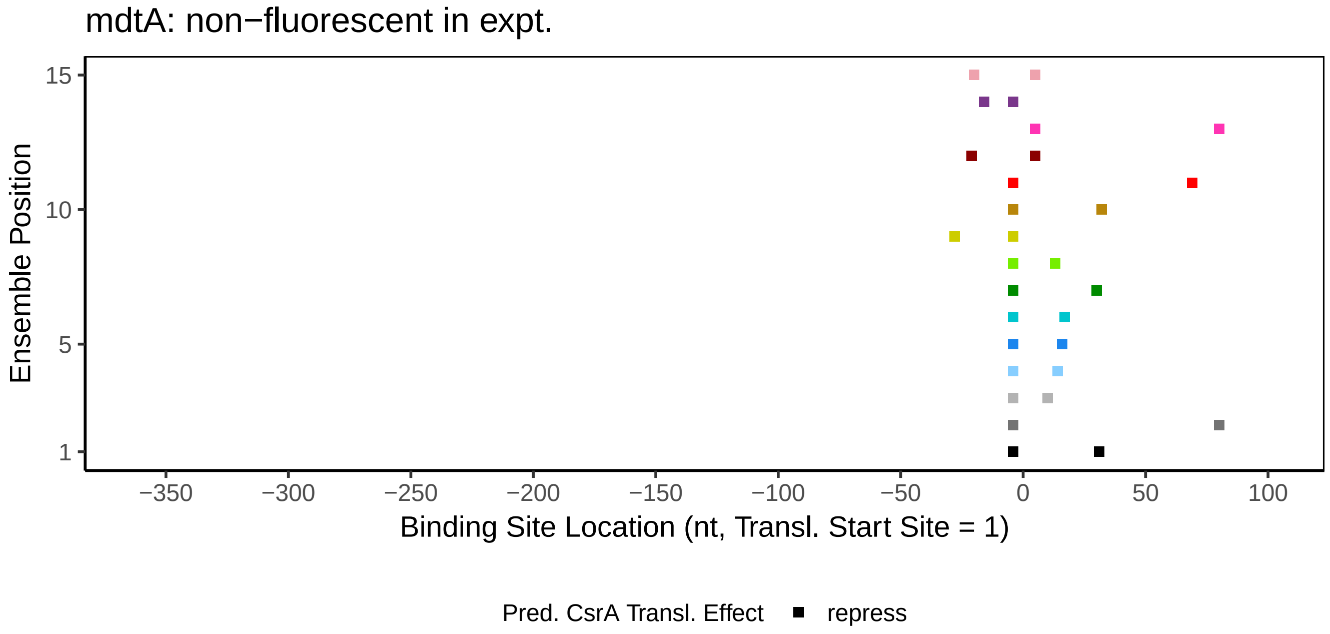

mdtA: non−fluorescent in expt.
Ensemble Position
15
10
5
1
−350
−300
−250
−200
−150
−100
−50
0
50
100
Binding Site Location (nt, Transl. Start Site = 1)
Pred. CsrA Transl. Effect
repress

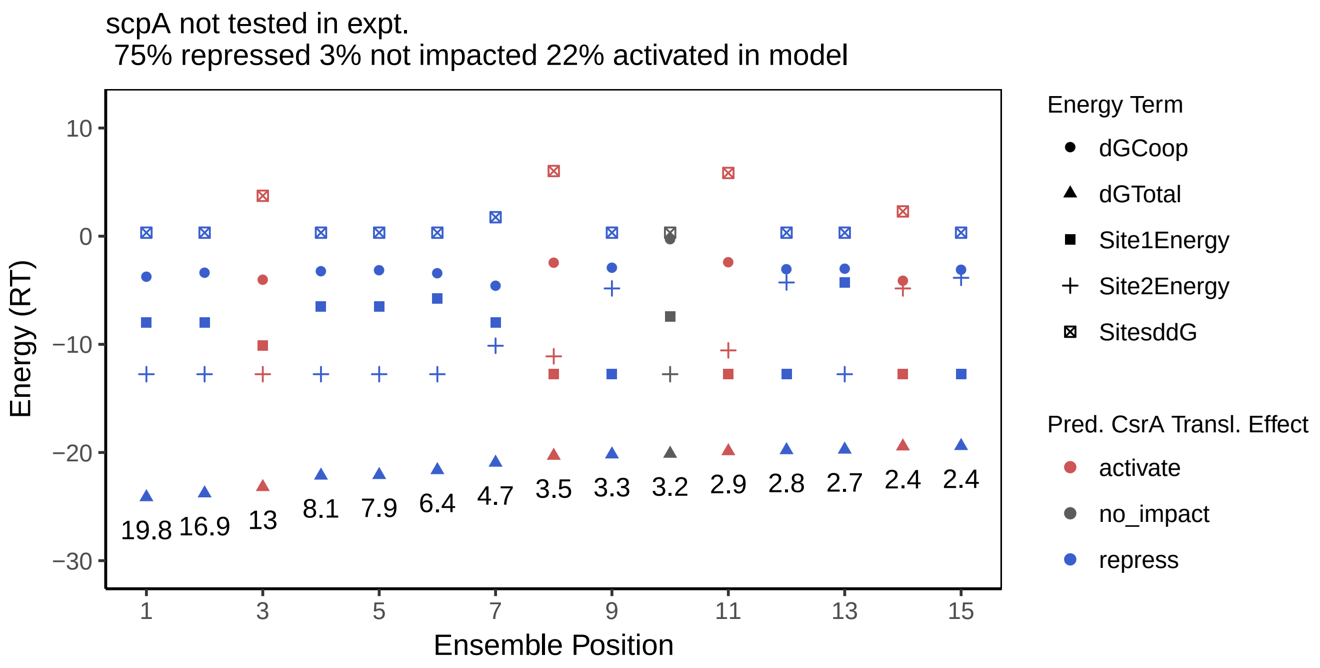
scpA not tested in expt.
75% repressed 3% not impacted 22% activated in model
Energy (RT)
10
0
−10
−20
−30
19.8
16.9
13
8.1
7.9
6.4
4.7
3.5
3.3
3.2
2.9
2.8
2.7
2.4
2.4
1
3
5
7
9
11
13
15
Ensemble Position
Energy Term
dGCoop
dGTotal
Site1Energy
Site2Energy
SitesddG
Pred. CsrA Transl. Effect
activate
no_impact
repress

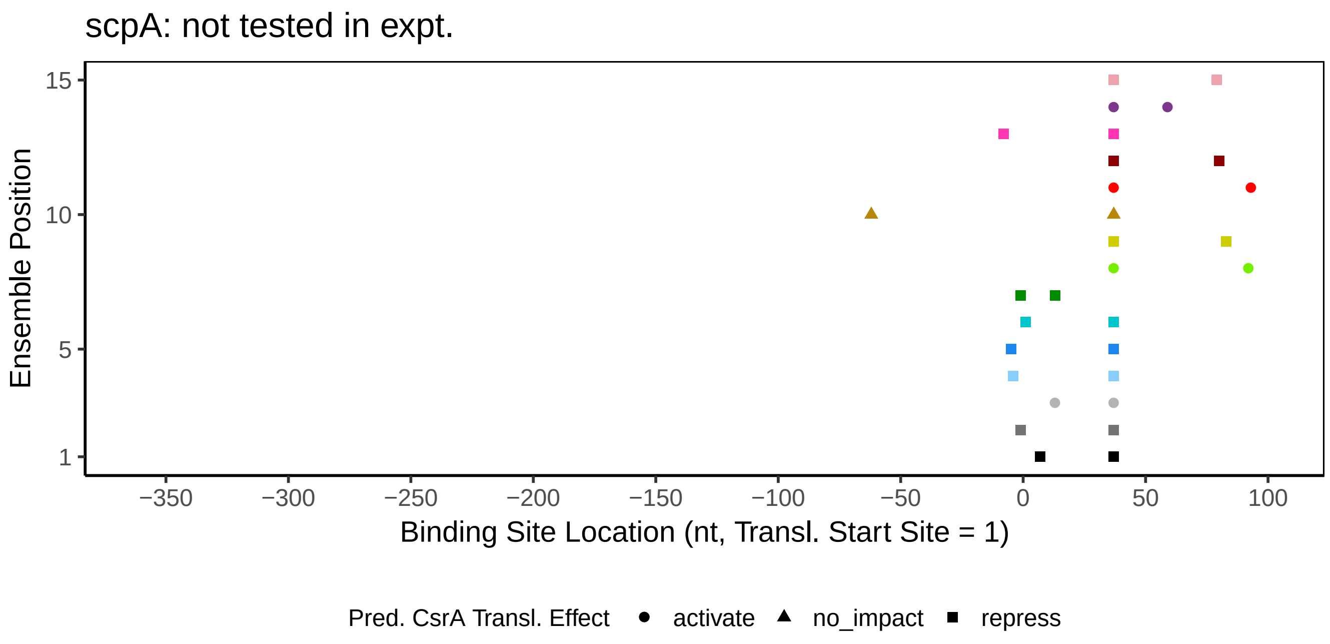
scpA: not tested in expt.
Ensemble Position
15
10
5
1
−350
−300
−250
−200
−150
−100
−50
0
50
100
Binding Site Location (nt, Transl. Start Site = 1)
Pred. CsrA Transl. Effect
activate
no_impact
repress

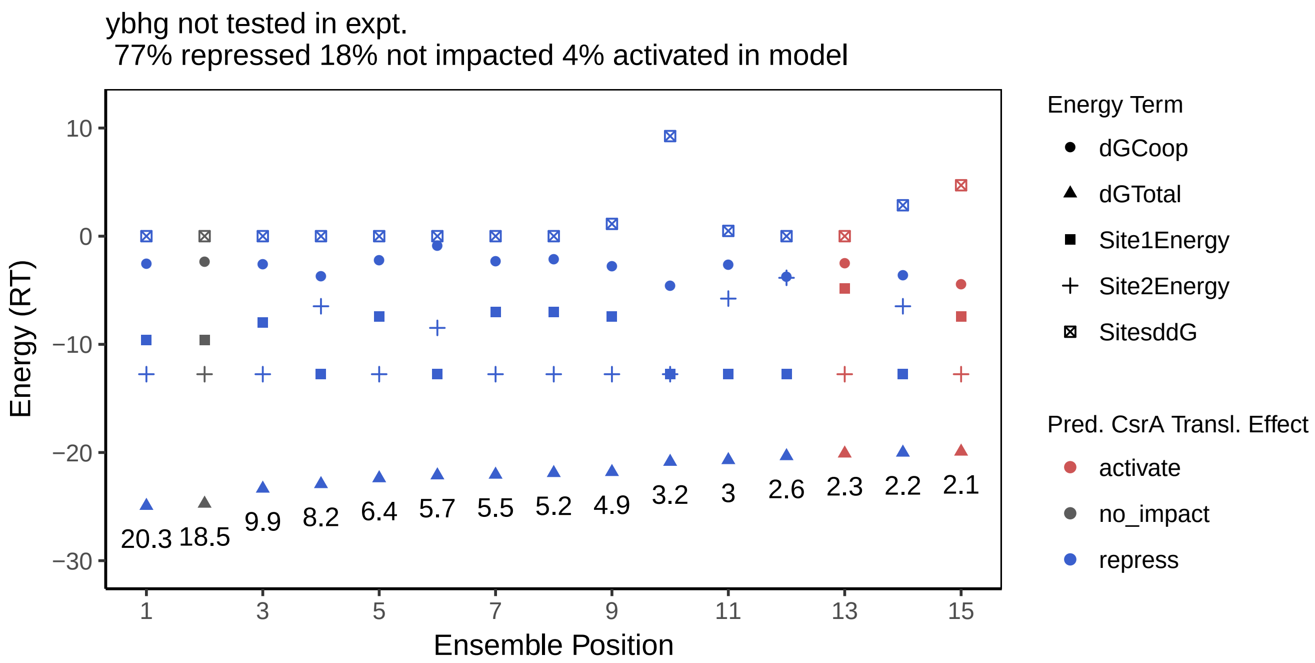

ybhg not tested in expt.
77% repressed 18% not impacted 4% activated in model
Energy (RT)
10
0
−10
−20
−30
20.3
18.5
9.9
8.2
6.4
5.7
5.5
5.2
4.9
3.2
3
2.6
2.3
2.2
2.1
1
3
5
7
9
11
13
15
Ensemble Position
Energy Term
dGCoop
dGTotal
Site1Energy
Site2Energy
SitesddG
Pred. CsrA Transl. Effect
activate
no_impact
repress

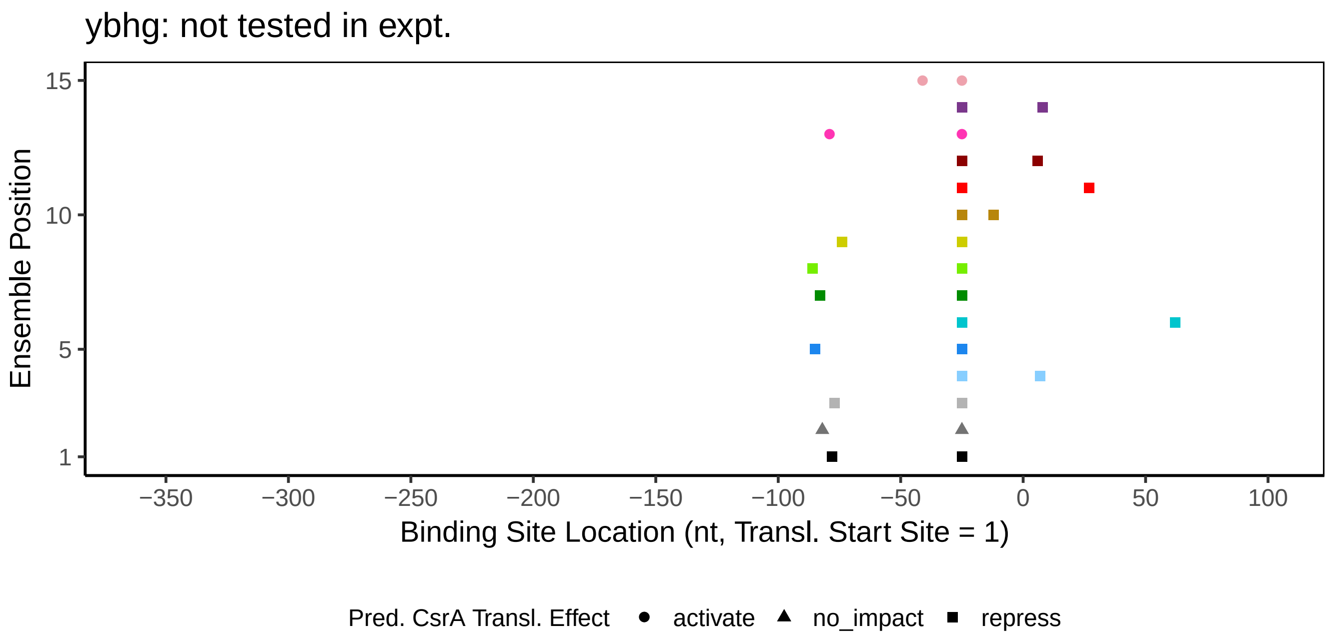

ybhg: not tested in expt.
Ensemble Position
15
10
5
1
−350
−300
−250
−200
−150
−100
−50
0
50
100
Binding Site Location (nt, Transl. Start Site = 1)
Pred. CsrA Transl. Effect
activate
no_impact
repress

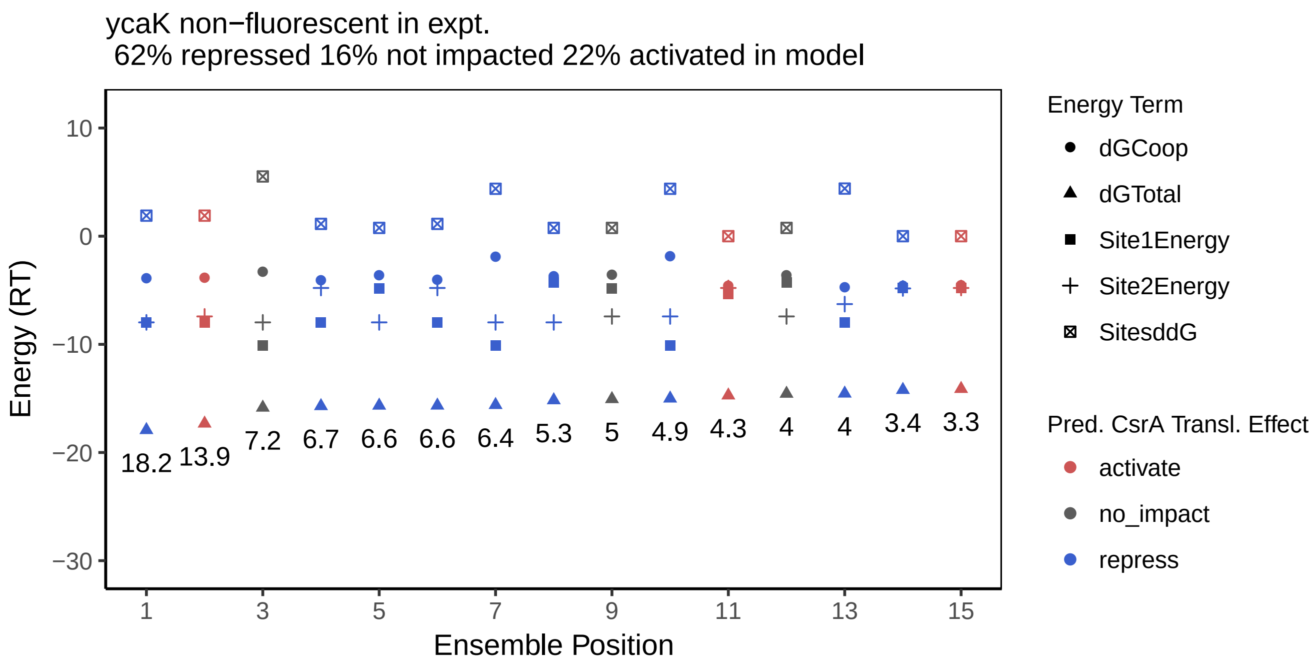

ycaK non−fluorescent in expt.
62% repressed 16% not impacted 22% activated in model
Energy (RT)
10
0
−10
−20
−30
18.2
13.9
7.2
6.7
6.6
6.6
6.4
5.3
5
4.9
4.3
4
4
3.4
3.3
1
3
5
7
9
11
13
15
Ensemble Position
Energy Term
dGCoop
dGTotal
Site1Energy
Site2Energy
SitesddG
Pred. CsrA Transl. Effect
activate
no_impact
repress

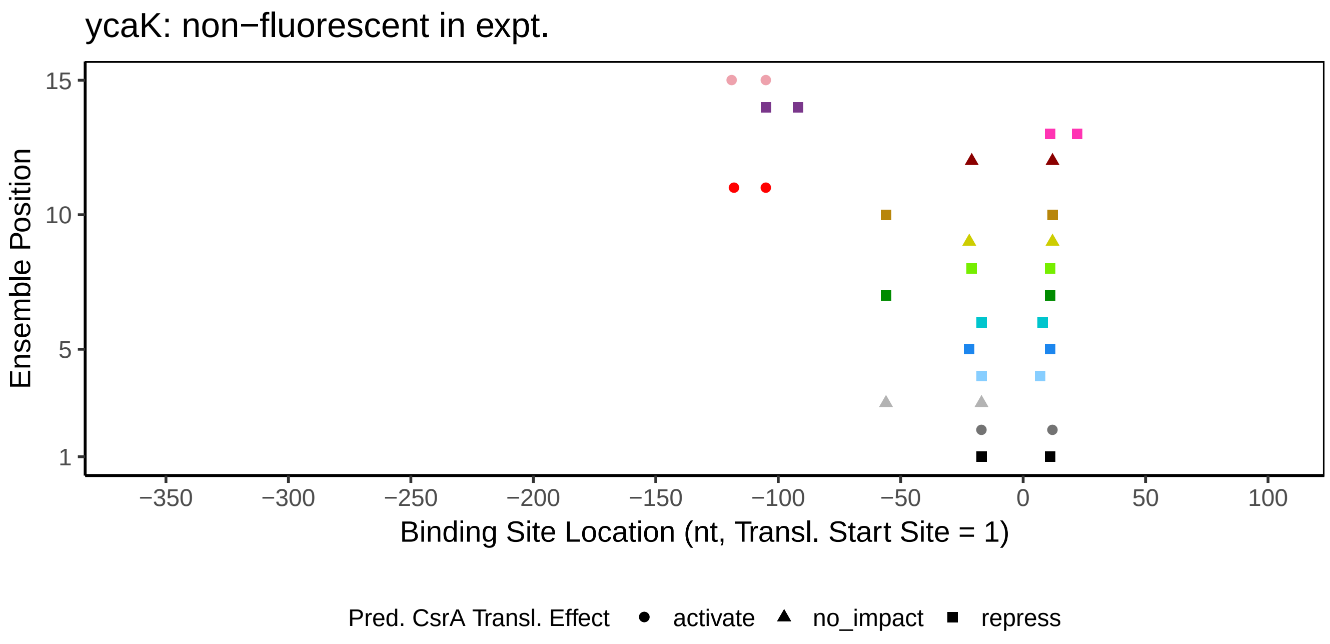

ycaK: non−fluorescent in expt.
Ensemble Position
15
10
5
1
−350
−300
−250
−200
−150
−100
−50
0
50
100
Binding Site Location (nt, Transl. Start Site = 1)
Pred. CsrA Transl. Effect
activate
no_impact
repress

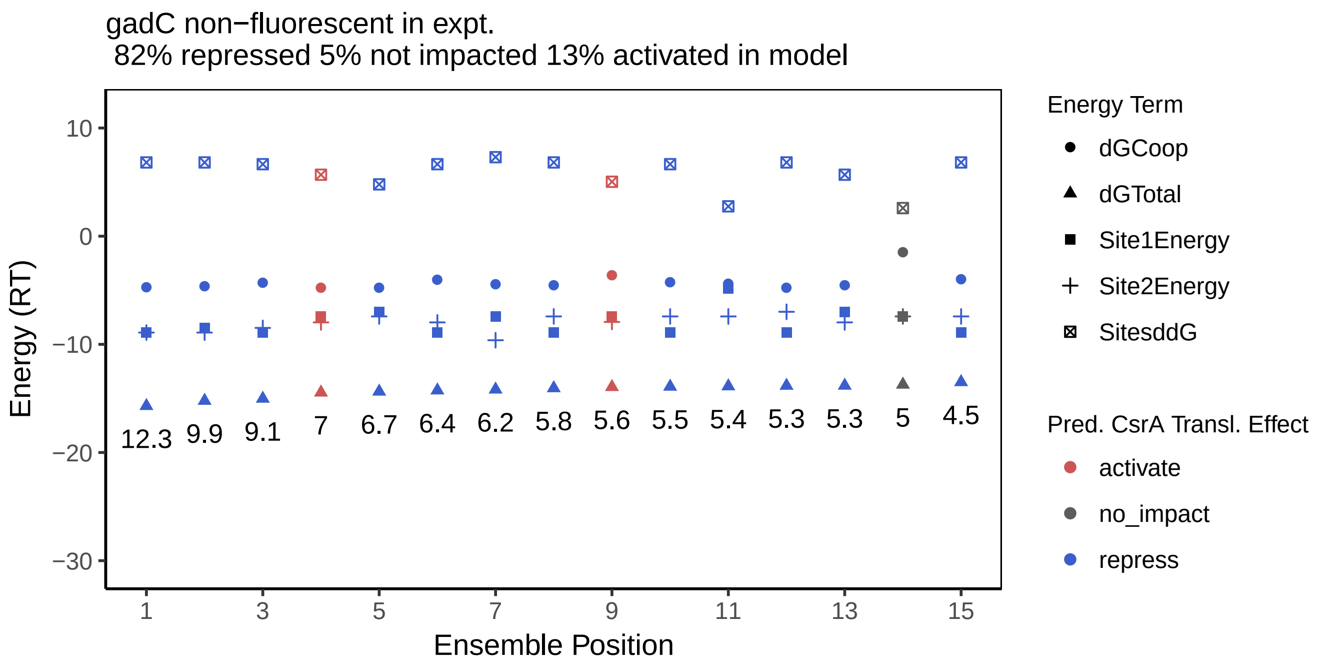

gadC non−fluorescent in expt.
82% repressed 5% not impacted 13% activated in model
Energy (RT)
10
0
−10
−20
−30
12.3 9.9 9.1 7 6.7 6.4 6.2 5.8 5.6 5.5 5.4 5.3 5.3 5 4.5
1 3 5 7 9 11 13 15
Ensemble Position
Energy Term
dGCoop
dGTotal
Site1Energy
Site2Energy
SitesddG
Pred. CsrA Transl. Effect
activate
no_impact
repress

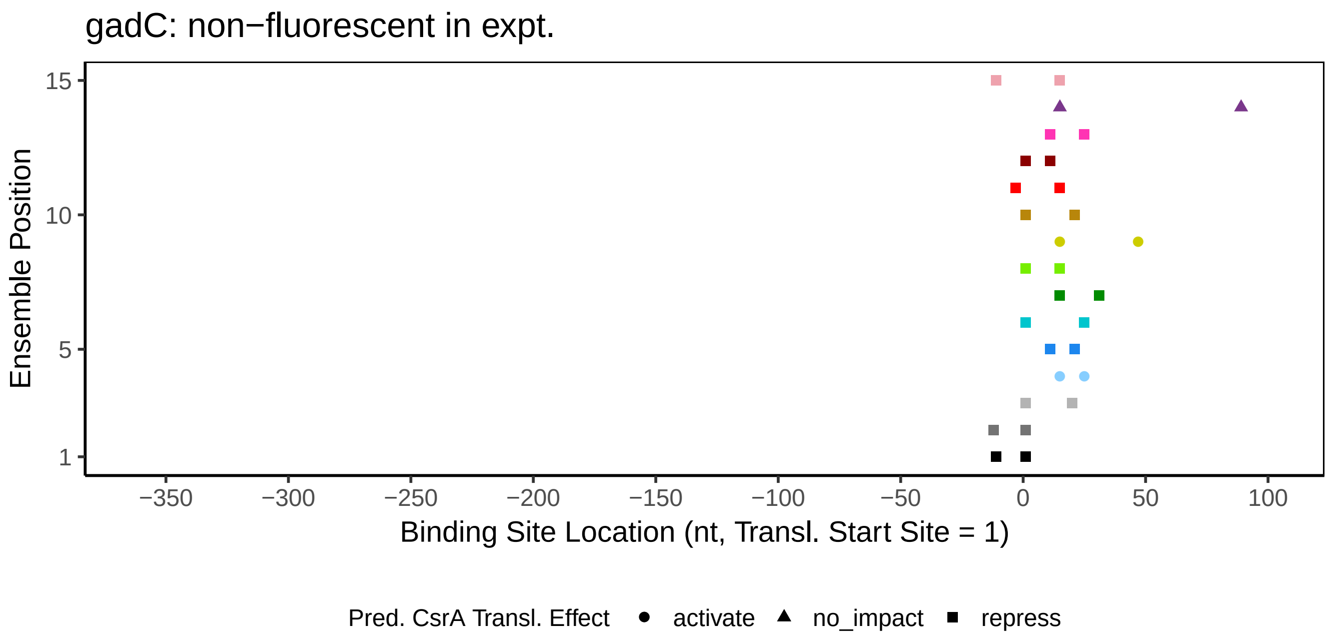

gadC: non−fluorescent in expt.
Ensemble Position
15
10
5
1
−350 −300 −250 −200 −150 −100 −50 0 50 100
Binding Site Location (nt, Transl. Start Site = 1)
Pred. CsrA Transl. Effect
activate
no_impact
repress

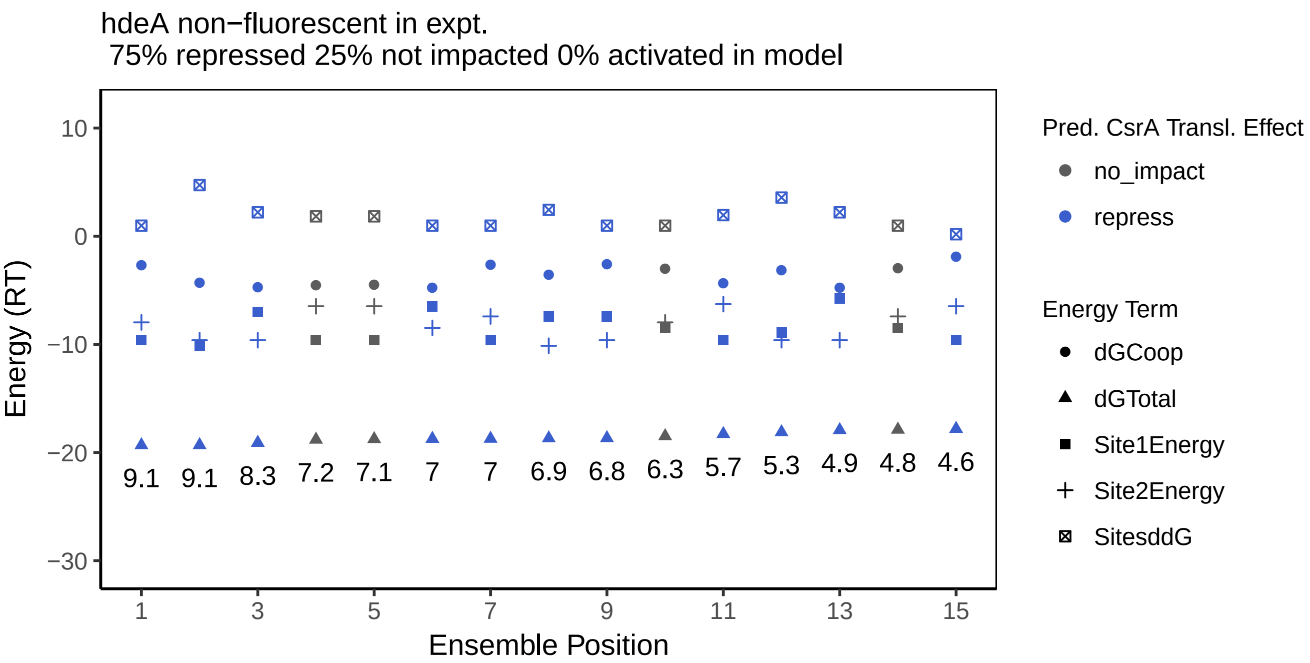
hdeA non−fluorescent in expt.
75% repressed 25% not impacted 0% activated in model
Energy (RT)
Ensemble Position
9.1 9.1 8.3 7.2 7.1 7 7 6.9 6.8 6.3 5.7 5.3 4.9 4.8 4.6
Pred. CsrA Transl. Effect
no_impact
repress
Energy Term
dGCoop
dGTotal
Site1Energy
Site2Energy
SitesddG

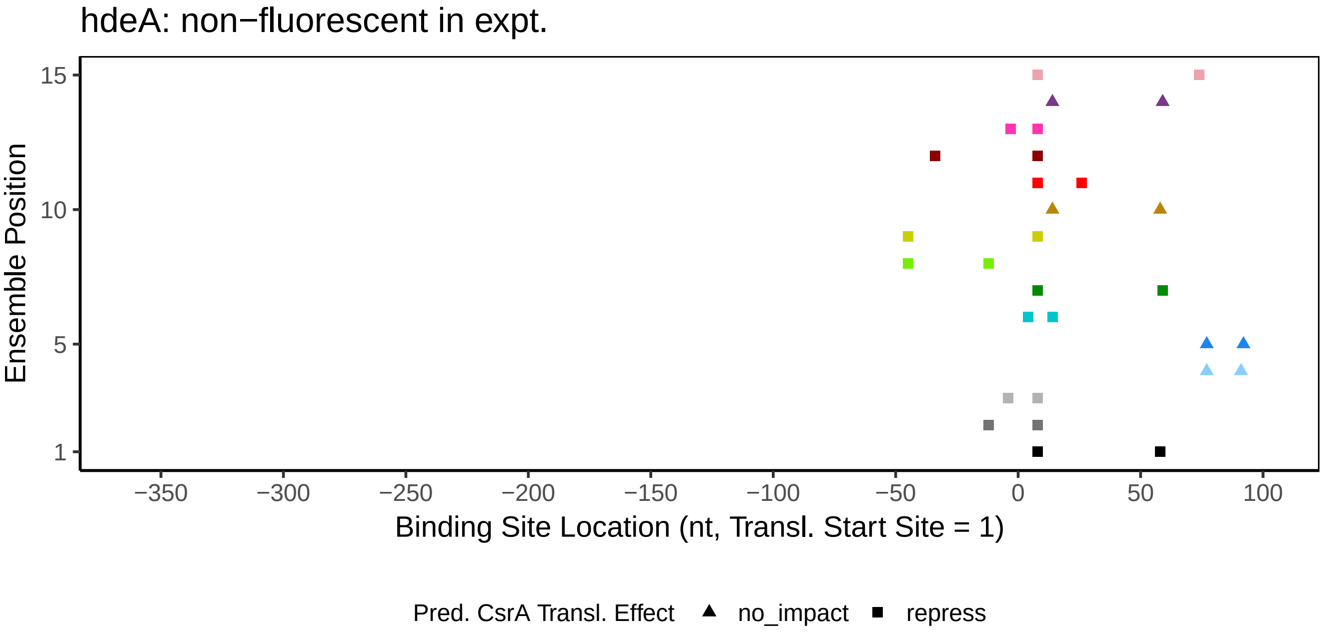
hdeA: non−fluorescent in expt.
Ensemble Position
Binding Site Location (nt, Transl. Start Site = 1)
Pred. CsrA Transl. Effect
no_impact
repress

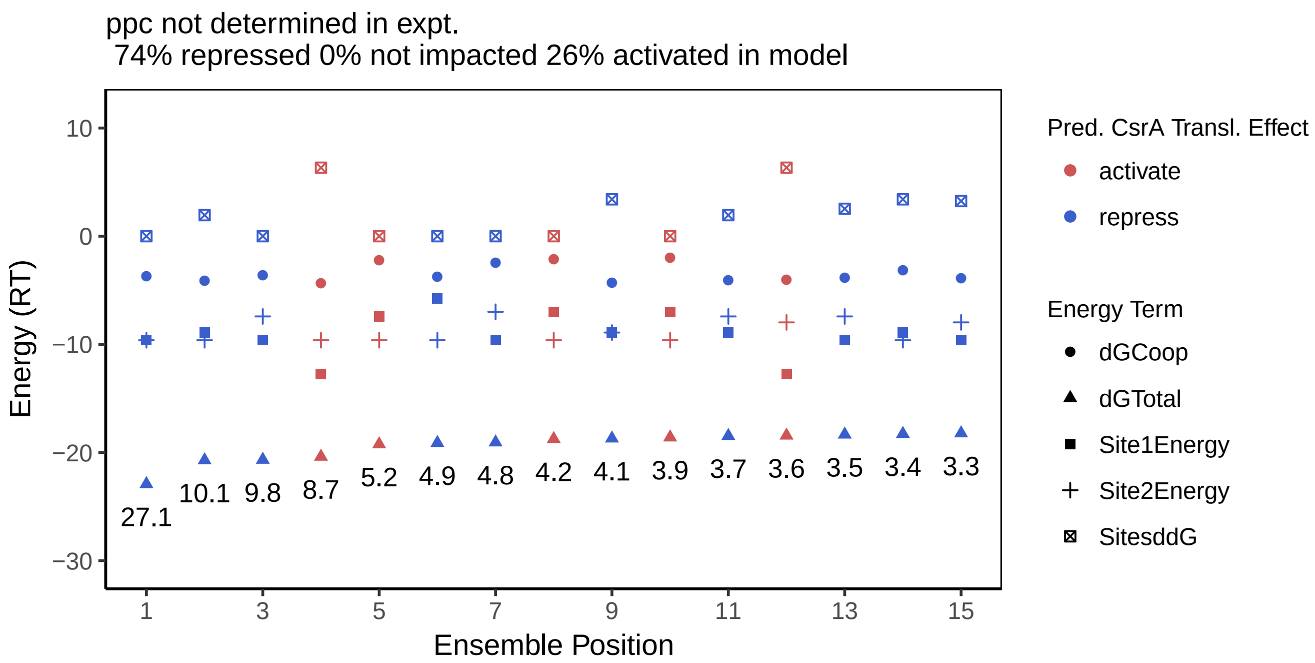

ppc not determined in expt.
74% repressed 0% not impacted 26% activated in model
Energy (RT)
Ensemble Position
27.1
10.1
9.8
8.7
5.2
4.9
4.8
4.2
4.1
3.9
3.7
3.6
3.5
3.4
3.3
Pred. CsrA Transl. Effect
activate
repress
Energy Term
dGCoop
dGTotal
Site1Energy
Site2Energy
SitesddG

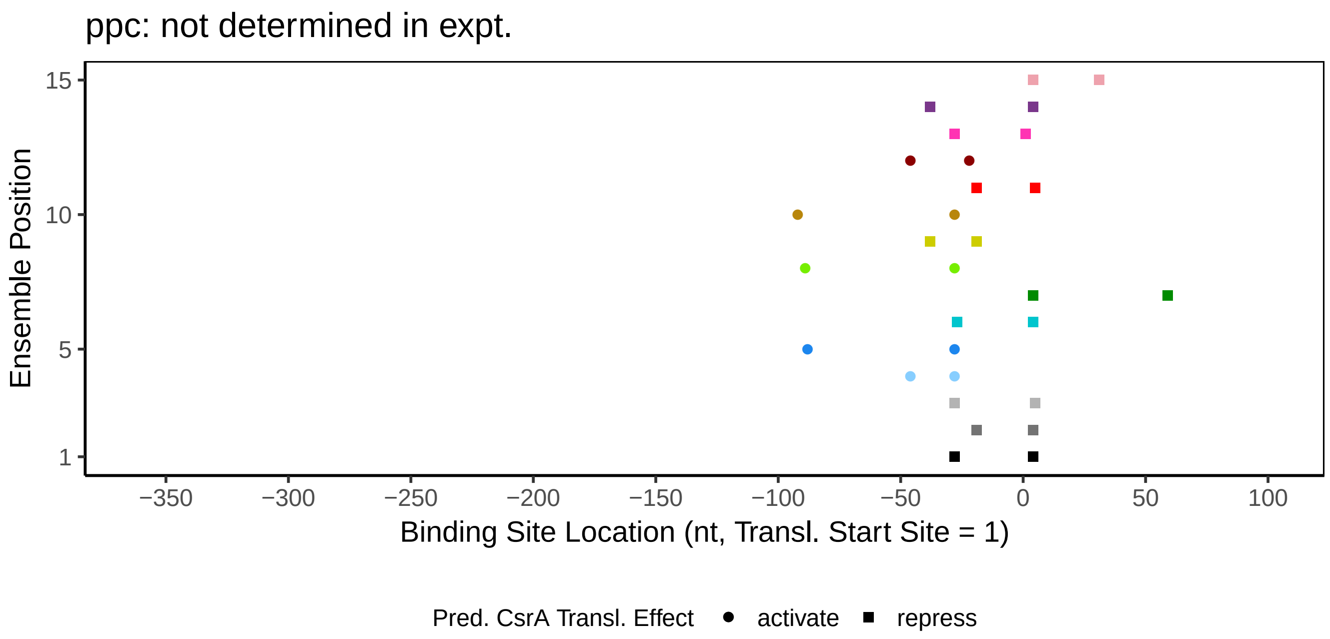

ppc: not determined in expt.
Ensemble Position
Binding Site Location (nt, Transl. Start Site = 1)
Pred. CsrA Transl. Effect
activate
repress

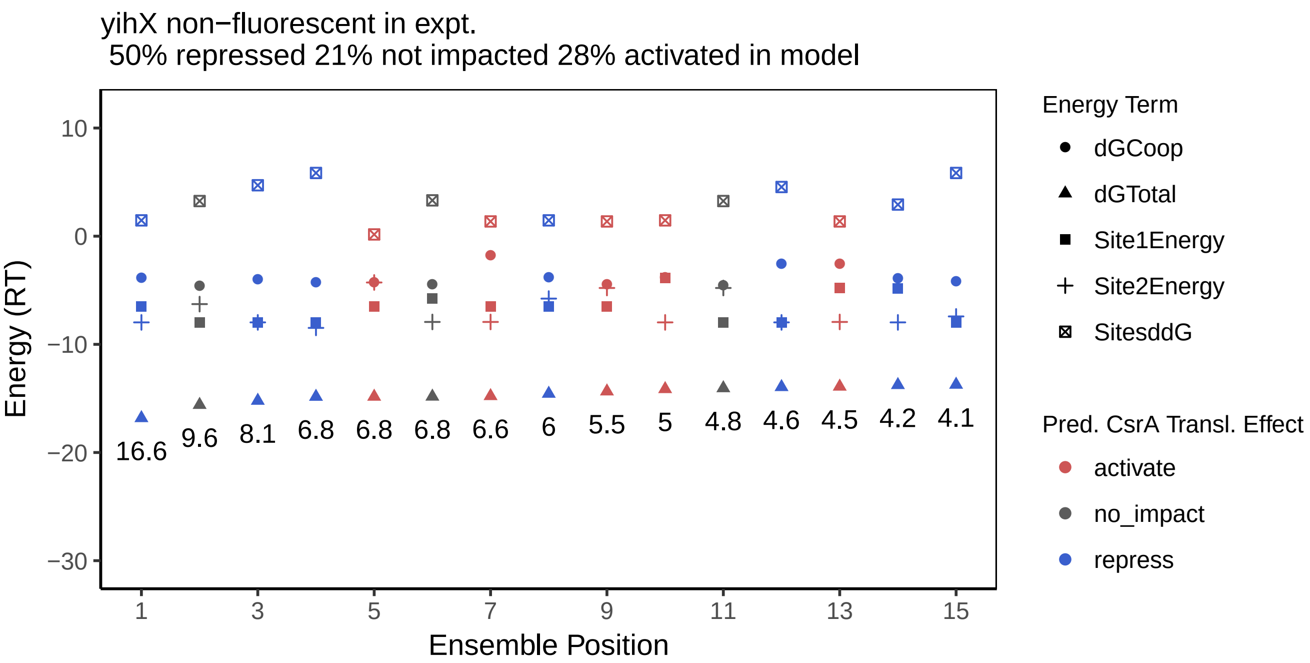

yihX non−fluorescent in expt.
50% repressed 21% not impacted 28% activated in model
Energy (RT)
10
0
−10
−20
−30
16.6
9.6
8.1
6.8
6.8
6.8
6.6
6
5.5
5
4.8
4.6
4.5
4.2
4.1
1
3
5
7
9
11
13
15
Ensemble Position
Energy Term
dGCoop
dGTotal
Site1Energy
Site2Energy
SitesddG
Pred. CsrA Transl. Effect
activate
no_impact
repress

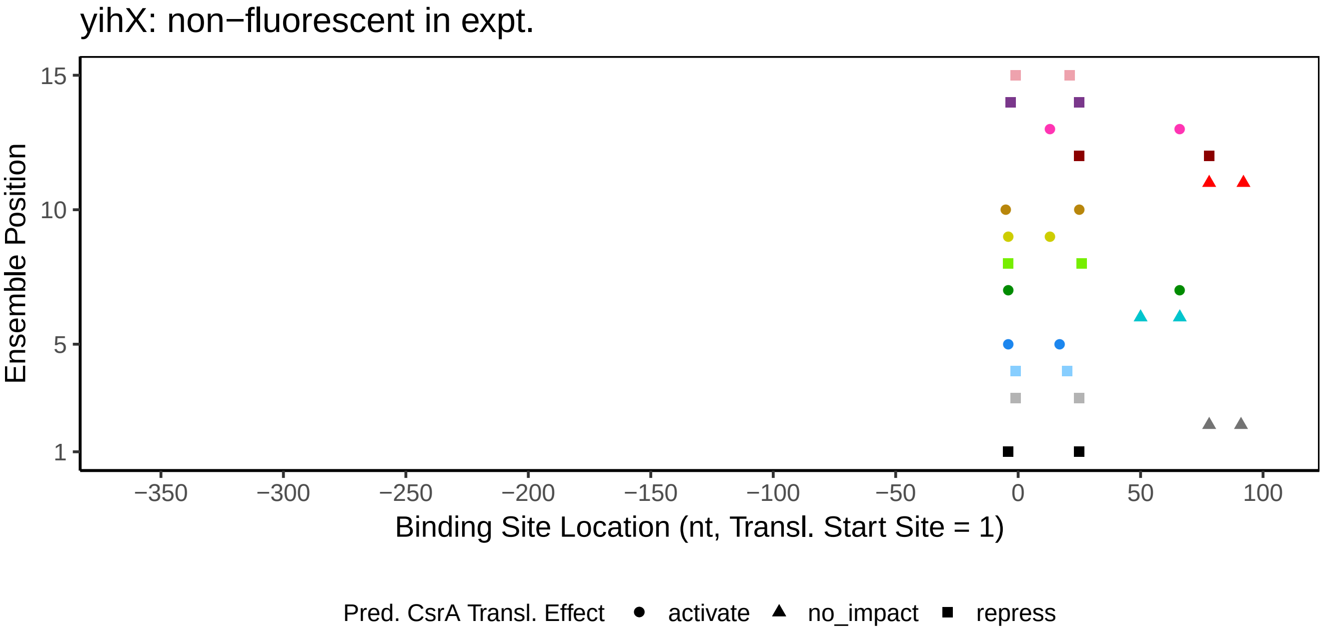

yihX: non−fluorescent in expt.
Ensemble Position
15
10
5
1
−350
−300
−250
−200
−150
−100
−50
0
50
100
Binding Site Location (nt, Transl. Start Site = 1)
Pred. CsrA Transl. Effect
activate
no_impact
repress

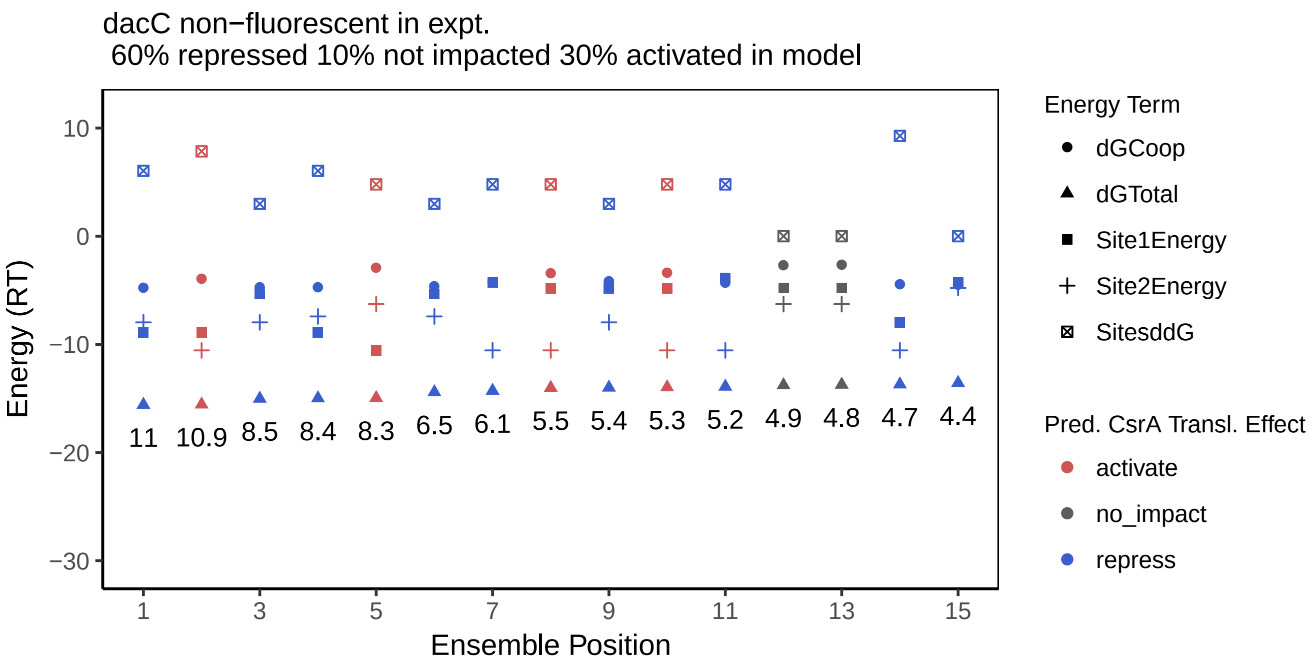

dacC non−fluorescent in expt.
60% repressed 10% not impacted 30% activated in model
Energy (RT)
Ensemble Position
11
10.9
8.5
8.4
8.3
6.5
6.1
5.5
5.4
5.3
5.2
4.9
4.8
4.7
4.4
Energy Term
dGCoop
dGTotal
Site1Energy
Site2Energy
SitesddG
Pred. CsrA Transl. Effect
activate
no_impact
repress

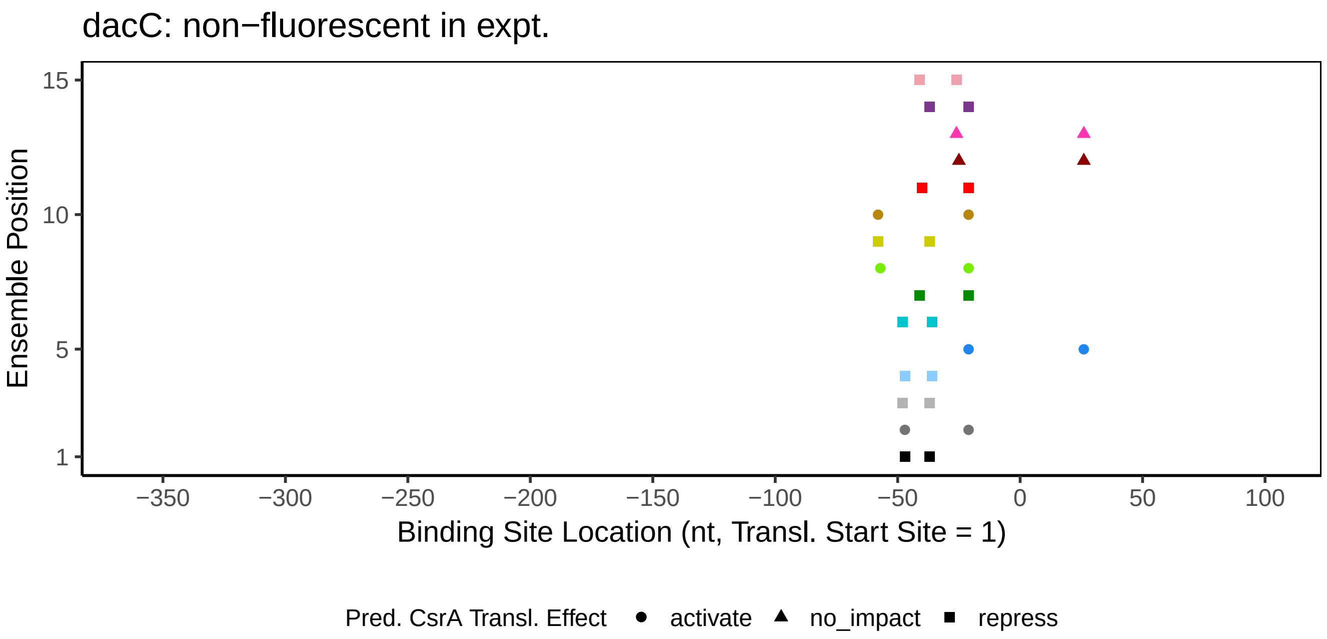

dacC: non−fluorescent in expt.
Ensemble Position
Binding Site Location (nt, Transl. Start Site = 1)
Pred. CsrA Transl. Effect
activate
no_impact
repress

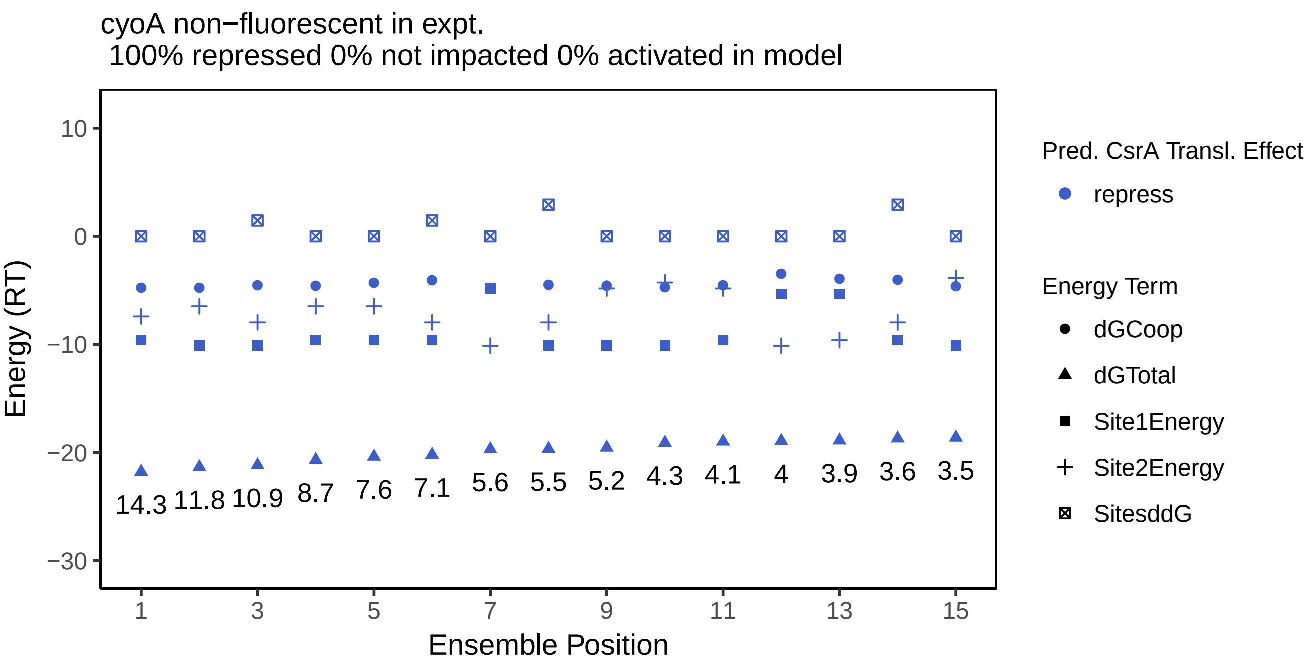
cyoA non−fluorescent in expt.
100% repressed 0% not impacted 0% activated in model
Energy (RT)
10
0
−10
−20
−30
14.3 11.8 10.9 8.7 7.6 7.1 5.6 5.5 5.2 4.3 4.1 4 3.9 3.6 3.5
1 3 5 7 9 11 13 15
Ensemble Position
Pred. CsrA Transl. Effect
repress
Energy Term
dGCoop
dGTotal
Site1Energy
Site2Energy
SitesddG

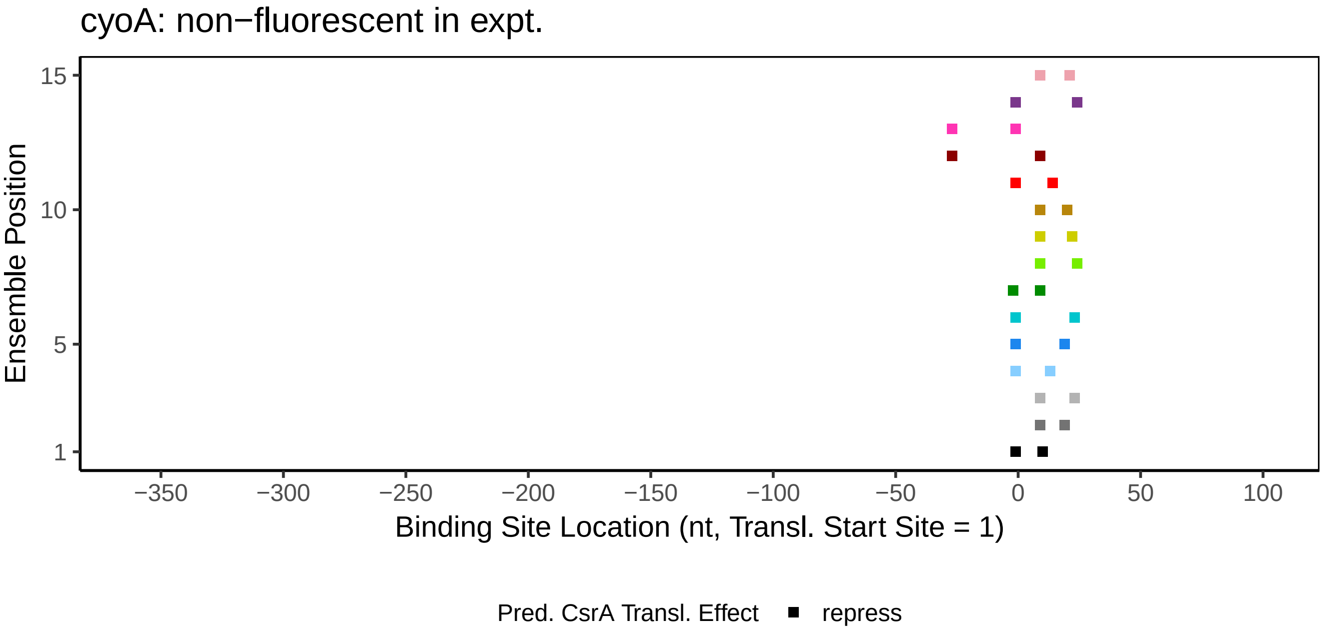
cyoA: non−fluorescent in expt.
Ensemble Position
15
10
5
1
−350 −300 −250 −200 −150 −100 −50 0 50 100
Binding Site Location (nt, Transl. Start Site = 1)
Pred. CsrA Transl. Effect
repress

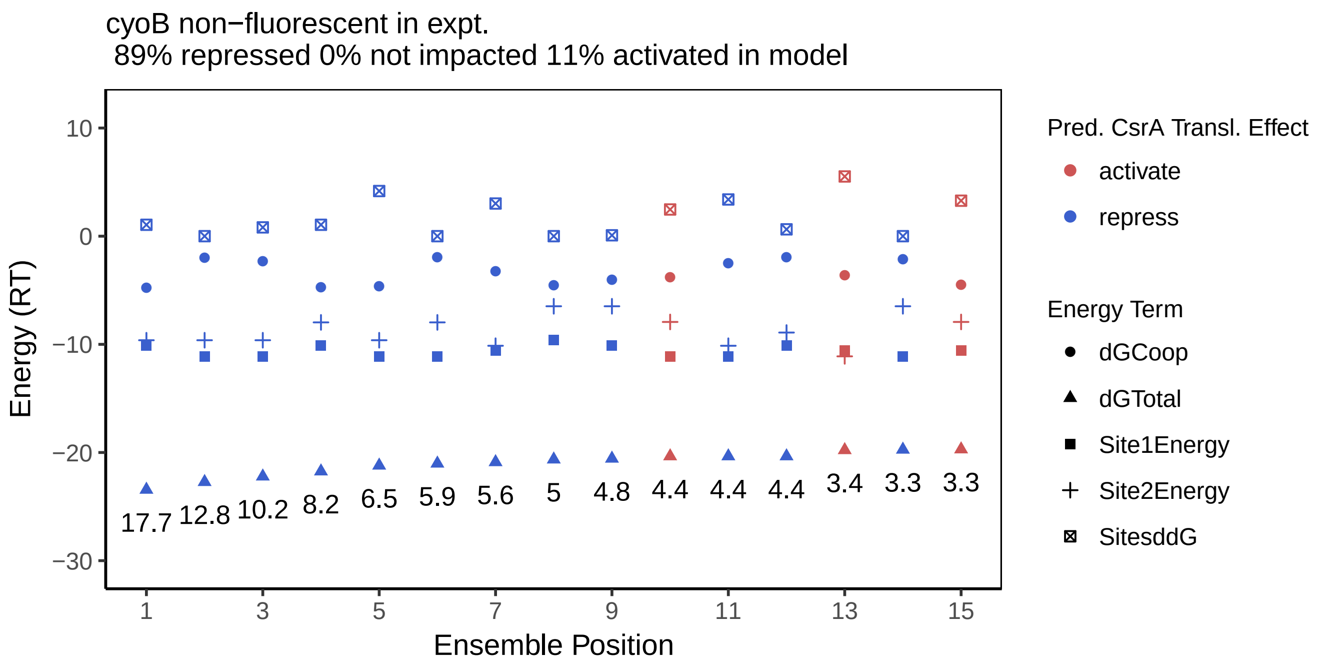
cyoB non−fluorescent in expt.
89% repressed 0% not impacted 11% activated in model
Energy (RT)
Ensemble Position
17.7 12.8 10.2 8.2 6.5 5.9 5.6 5 4.8 4.4 4.4 4.4 3.4 3.3 3.3
Pred. CsrA Transl. Effect
activate
repress
Energy Term
dGCoop
dGTotal
Site1Energy
Site2Energy
SitesddG

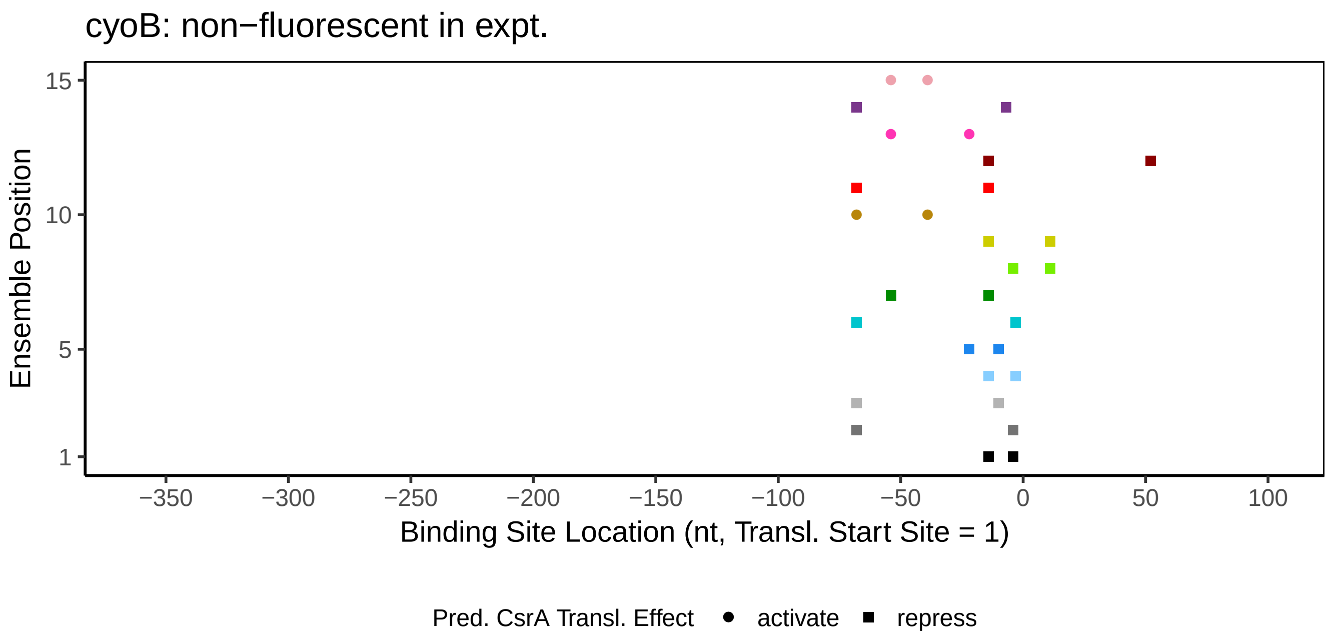
cyoB: non−fluorescent in expt.
Ensemble Position
Binding Site Location (nt, Transl. Start Site = 1)
Pred. CsrA Transl. Effect
activate
repress

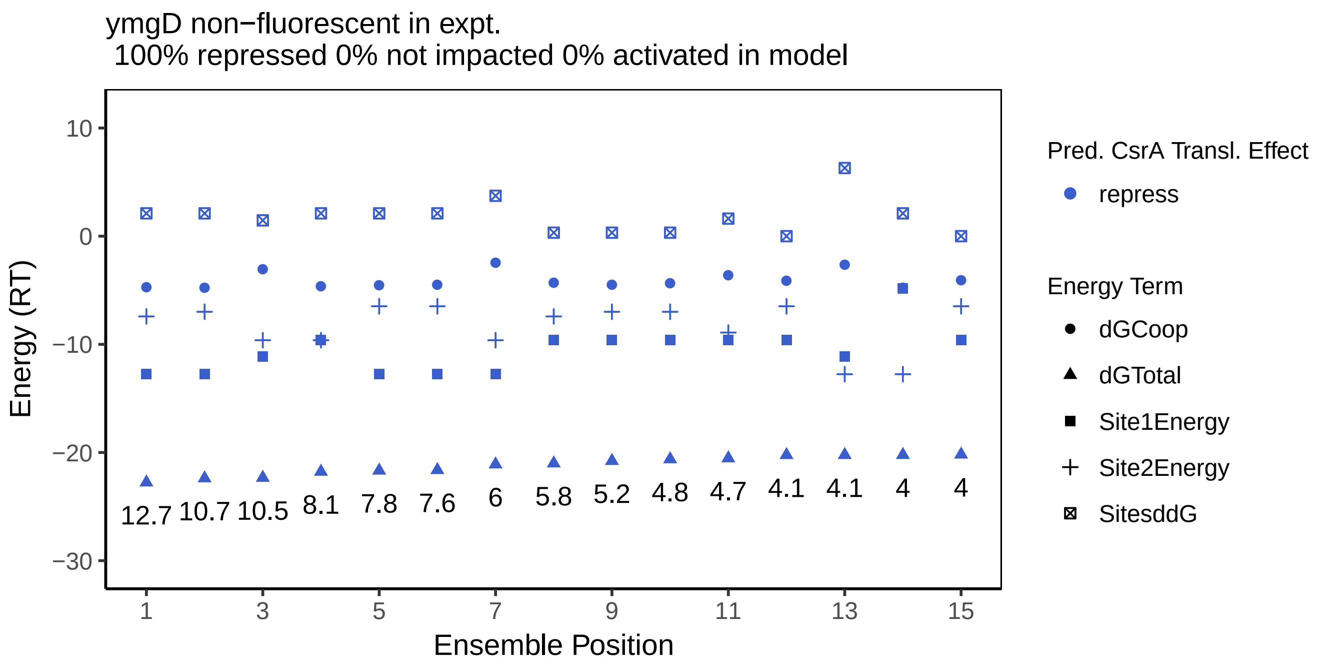
ymgD non−fluorescent in expt.
100% repressed 0% not impacted 0% activated in model
Energy (RT)
10
0
−10
−20
−30
12.7 10.7 10.5 8.1 7.8 7.6 6 5.8 5.2 4.8 4.7 4.1 4.1 4 4
1 3 5 7 9 11 13 15
Ensemble Position
Pred. CsrA Transl. Effect
repress
Energy Term
dGCoop
dGTotal
Site1Energy
Site2Energy
SitesddG

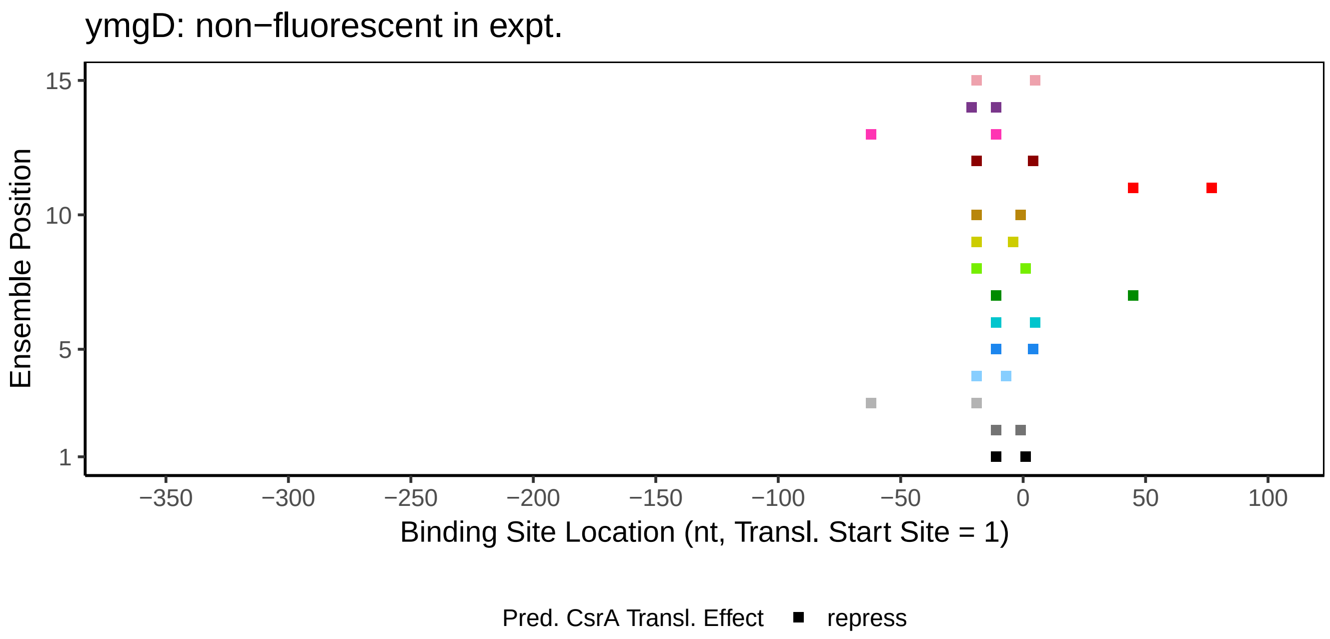
ymgD: non−fluorescent in expt.
Ensemble Position
15
10
5
1
−350 −300 −250 −200 −150 −100 −50 0 50 100
Binding Site Location (nt, Transl. Start Site = 1)
Pred. CsrA Transl. Effect
repress

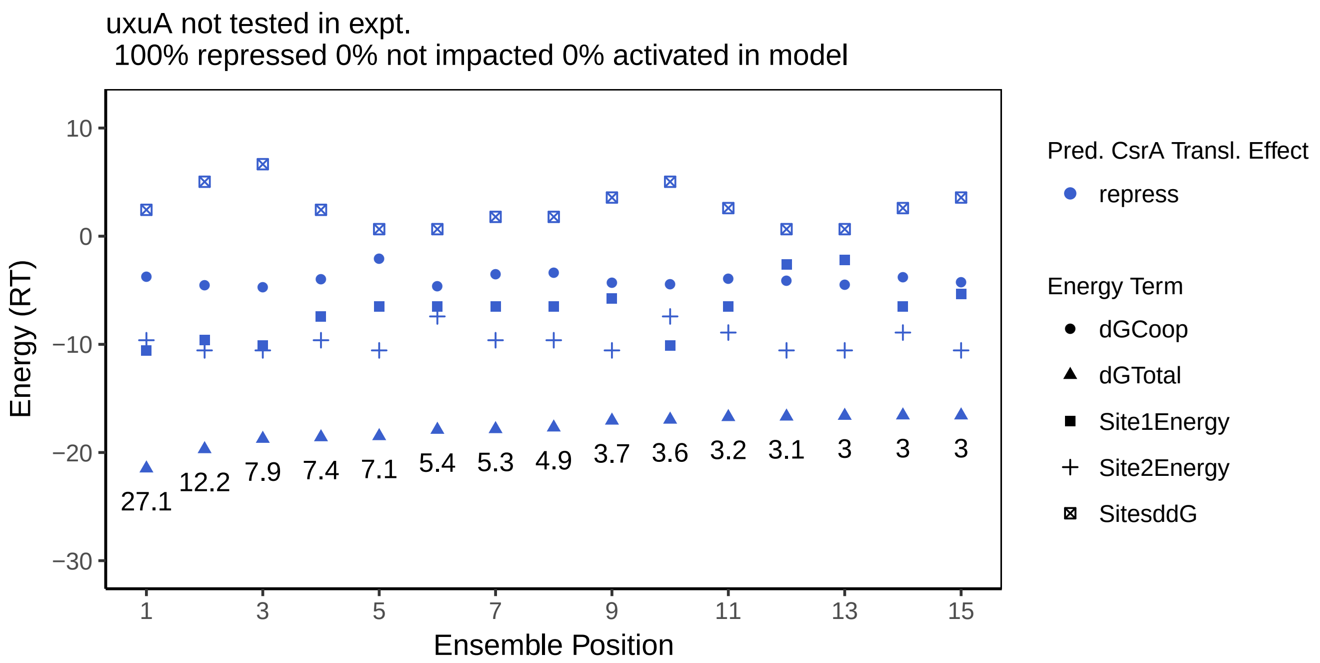

uxuA not tested in expt.
100% repressed 0% not impacted 0% activated in model
Energy (RT)
10
0
−10
−20
−30
27.1
12.2
7.9
7.4
7.1
5.4
5.3
4.9
3.7
3.6
3.2
3.1
3
3
3
1
3
5
7
9
11
13
15
Ensemble Position
Pred. CsrA Transl. Effect
repress
Energy Term
dGCoop
dGTotal
Site1Energy
Site2Energy
SitesddG

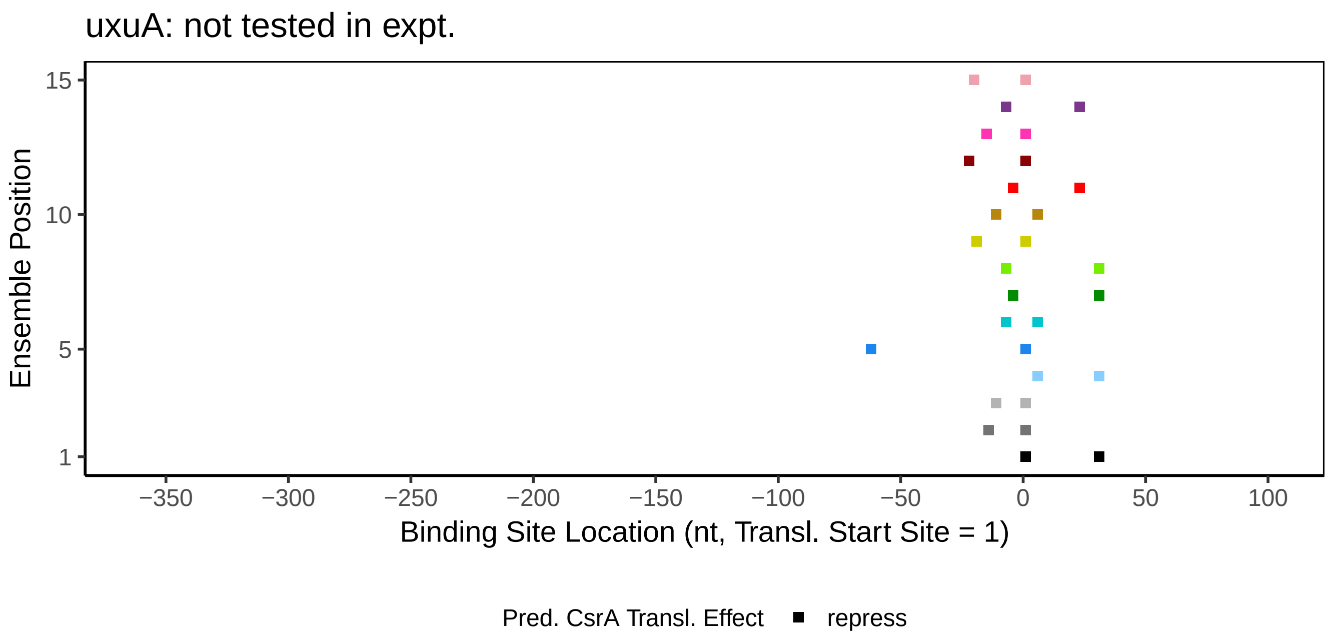

uxuA: not tested in expt.
Ensemble Position
15
10
5
1
−350
−300
−250
−200
−150
−100
−50
0
50
100
Binding Site Location (nt, Transl. Start Site = 1)
Pred. CsrA Transl. Effect
repress

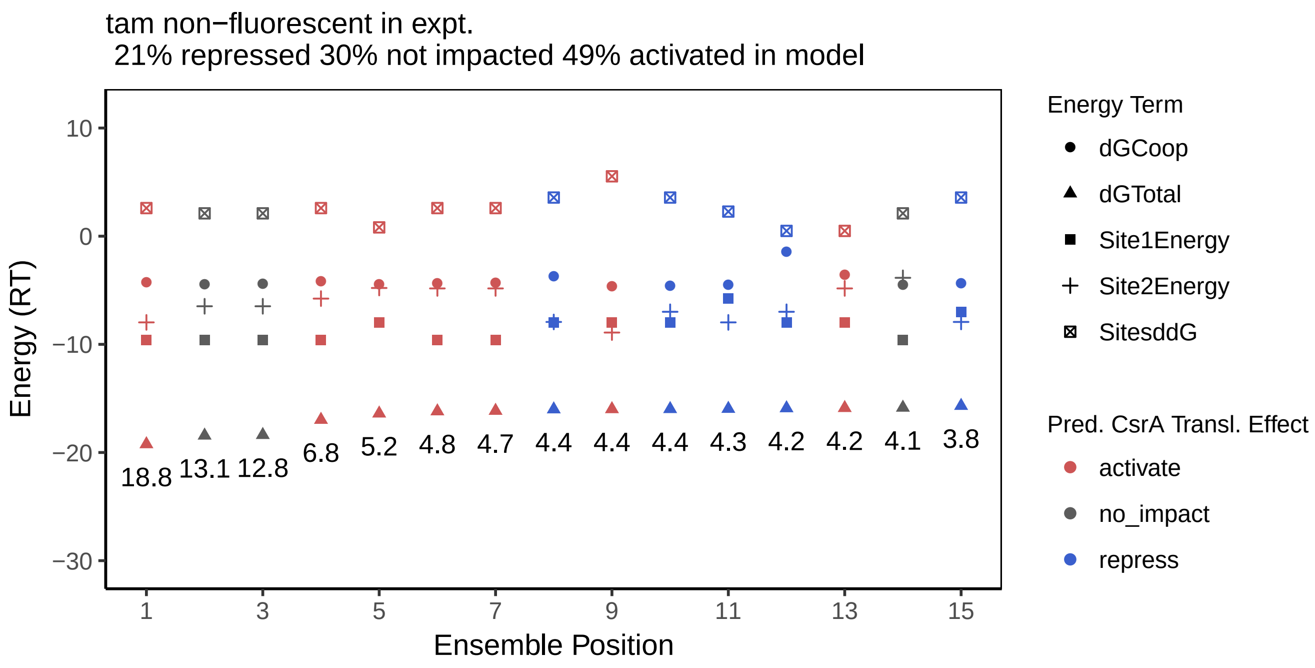
tam non−fluorescent in expt.
21% repressed 30% not impacted 49% activated in model
Energy (RT)
10
0
−10
−20
−30
18.8
13.1
12.8
6.8
5.2
4.8
4.7
4.4
4.4
4.4
4.3
4.2
4.2
4.1
3.8
1
3
5
7
9
11
13
15
Ensemble Position
Energy Term
dGCoop
dGTotal
Site1Energy
Site2Energy
SitesddG
Pred. CsrA Transl. Effect
activate
no_impact
repress

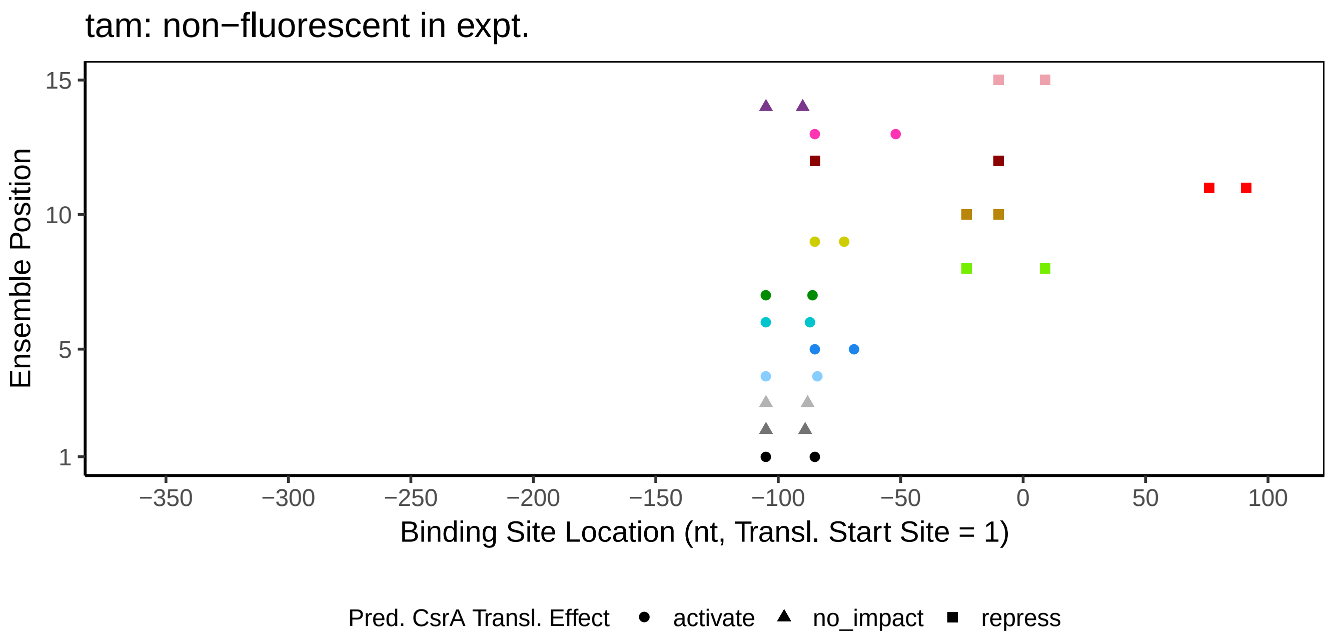
tam: non−fluorescent in expt.
Ensemble Position
15
10
5
1
−350
−300
−250
−200
−150
−100
−50
0
50
100
Binding Site Location (nt, Transl. Start Site = 1)
Pred. CsrA Transl. Effect
activate
no_impact
repress

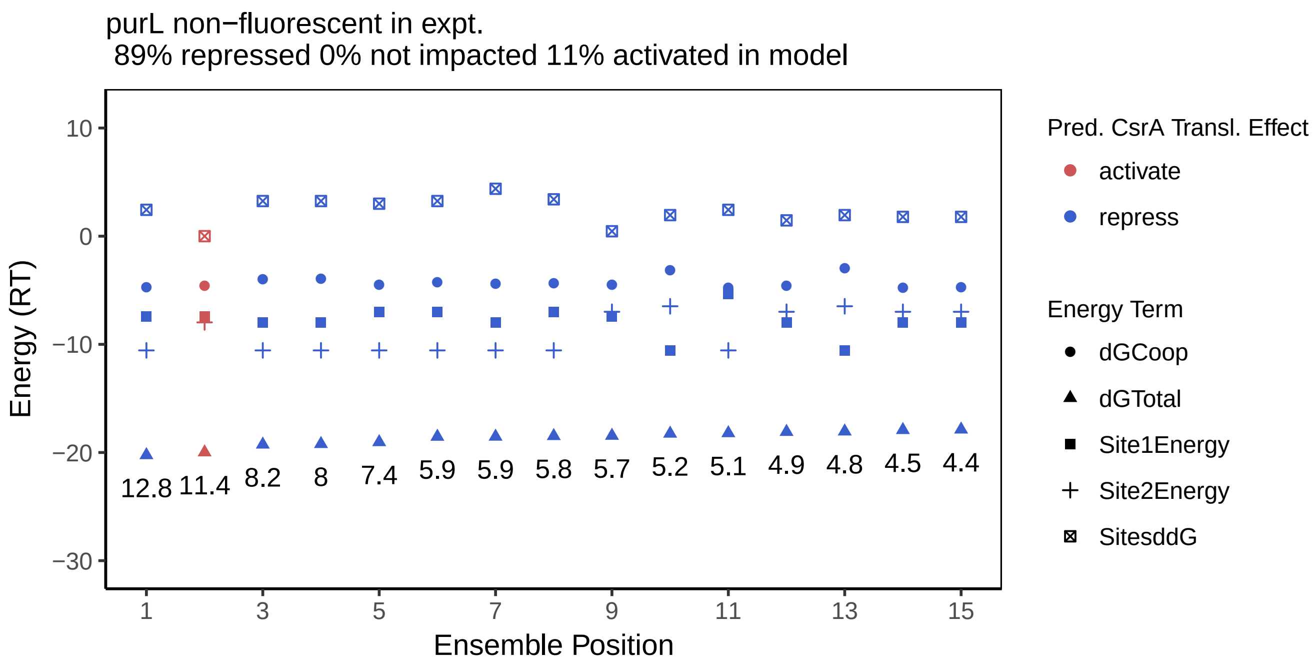

purL non−fluorescent in expt.
89% repressed 0% not impacted 11% activated in model
Energy (RT)
Ensemble Position
12.8 11.4 8.2 8 7.4 5.9 5.9 5.8 5.7 5.2 5.1 4.9 4.8 4.5 4.4
Pred. CsrA Transl. Effect
activate
repress
Energy Term
dGCoop
dGTotal
Site1Energy
Site2Energy
SitesddG

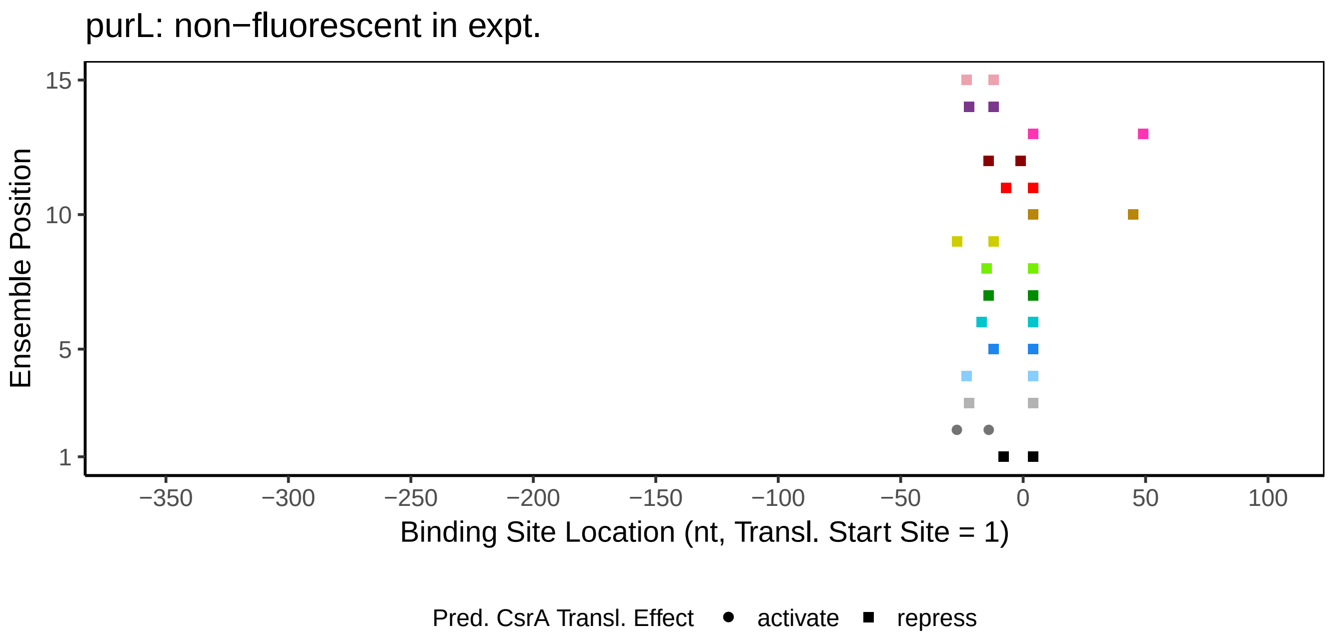

purL: non−fluorescent in expt.
Ensemble Position
Binding Site Location (nt, Transl. Start Site = 1)
Pred. CsrA Transl. Effect
activate
repress

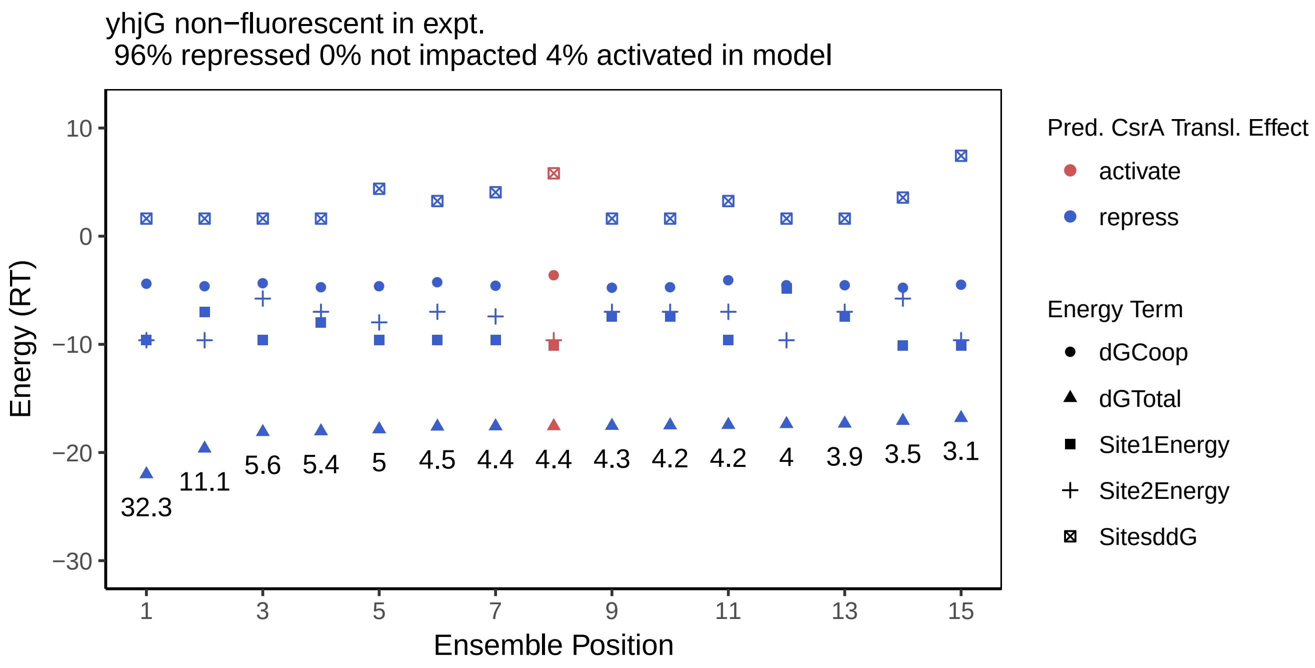

yhjG non−fluorescent in expt.
96% repressed 0% not impacted 4% activated in model
Energy (RT)
Ensemble Position
32.3
11.1
5.6
5.4
5
4.5
4.4
4.4
4.3
4.2
4.2
4
3.9
3.5
3.1
Pred. CsrA Transl. Effect
activate
repress
Energy Term
dGCoop
dGTotal
Site1Energy
Site2Energy
SitesddG

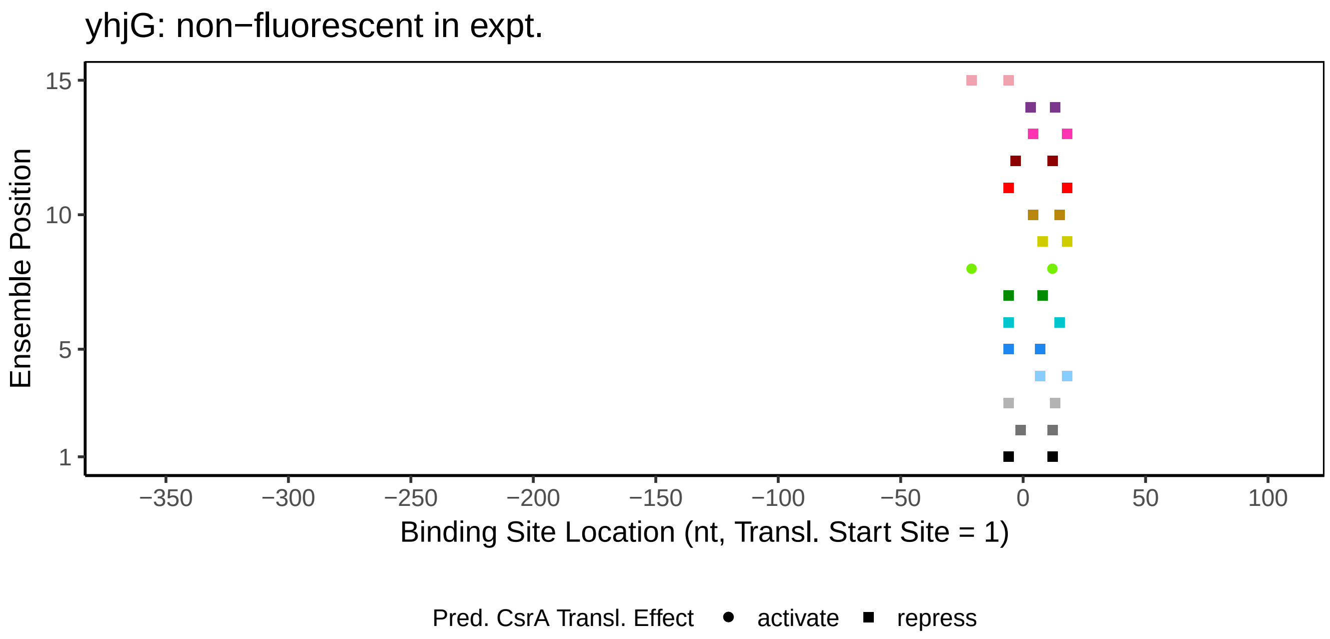

yhjG: non−fluorescent in expt.
Ensemble Position
Binding Site Location (nt, Transl. Start Site = 1)
Pred. CsrA Transl. Effect
activate
repress

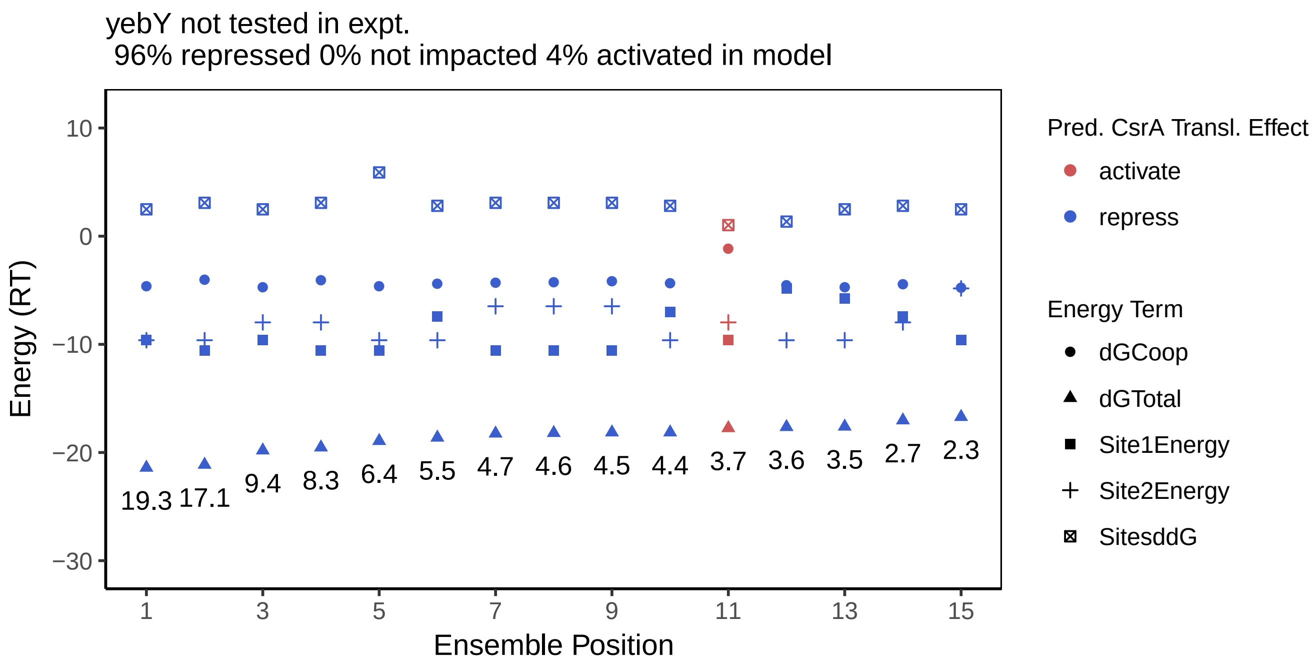
yebY not tested in expt.
96% repressed 0% not impacted 4% activated in model
Energy (RT)
10
0
−10
−20
−30
19.3
17.1
9.4
8.3
6.4
5.5
4.7
4.6
4.5
4.4
3.7
3.6
3.5
2.7
2.3
1
3
5
7
9
11
13
15
Ensemble Position
Pred. CsrA Transl. Effect
activate
repress
Energy Term
dGCoop
dGTotal
Site1Energy
Site2Energy
SitesddG

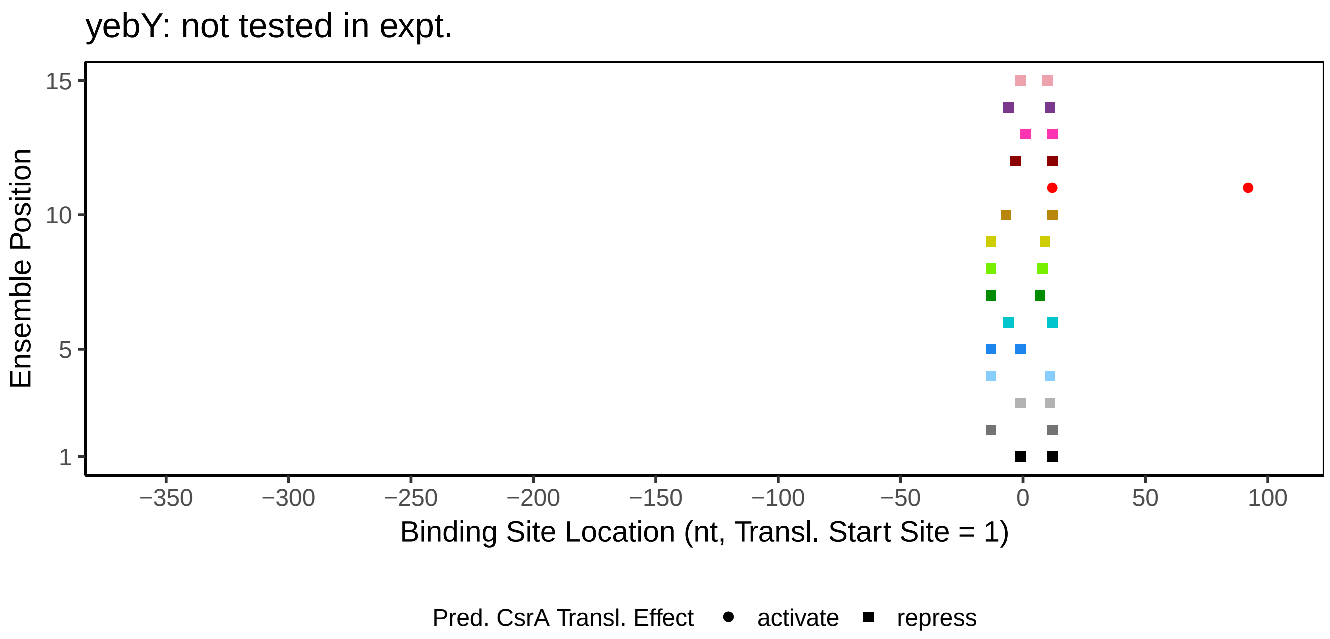
yebY: not tested in expt.
Ensemble Position
15
10
5
1
−350
−300
−250
−200
−150
−100
−50
0
50
100
Binding Site Location (nt, Transl. Start Site = 1)
Pred. CsrA Transl. Effect
activate
repress

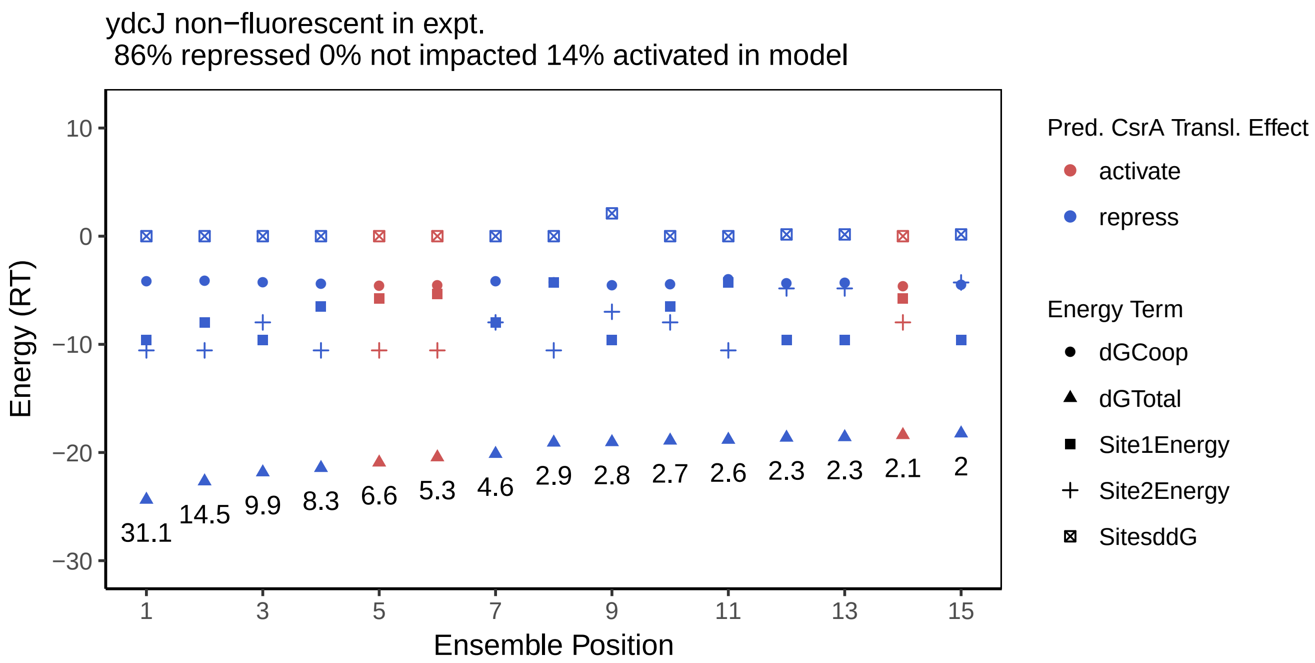
ydcJ non−fluorescent in expt.
86% repressed 0% not impacted 14% activated in model
Energy (RT)
10
0
−10
−20
−30
31.1
14.5
9.9
8.3
6.6
5.3
4.6
2.9
2.8
2.7
2.6
2.3
2.3
2.1
2
1
3
5
7
9
11
13
15
Ensemble Position
Pred. CsrA Transl. Effect
activate
repress
Energy Term
dGCoop
dGTotal
Site1Energy
Site2Energy
SitesddG

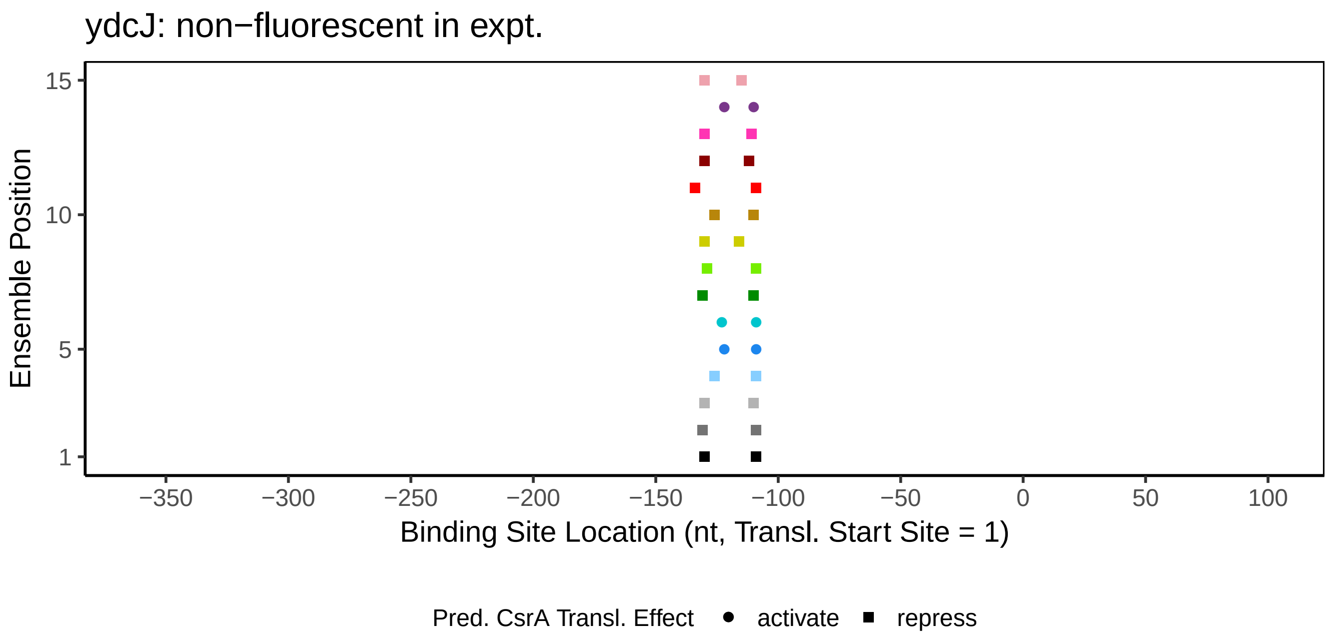
ydcJ: non−fluorescent in expt.
Ensemble Position
15
10
5
1
−350
−300
−250
−200
−150
−100
−50
0
50
100
Binding Site Location (nt, Transl. Start Site = 1)
Pred. CsrA Transl. Effect
activate
repress

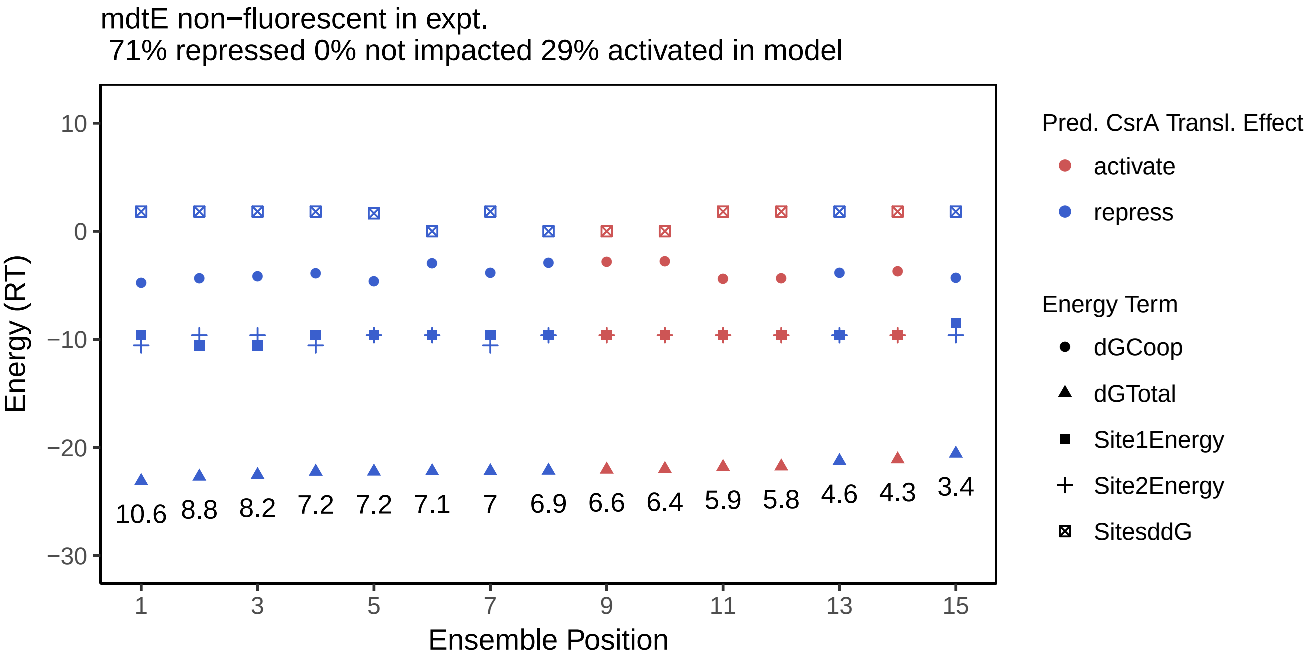
mdtE non−fluorescent in expt.
71% repressed 0% not impacted 29% activated in model
Energy (RT)
Ensemble Position
10.6 8.8 8.2 7.2 7.2 7.1 7 6.9 6.6 6.4 5.9 5.8 4.6 4.3 3.4
Pred. CsrA Transl. Effect
activate
repress
Energy Term
dGCoop
dGTotal
Site1Energy
Site2Energy
SitesddG

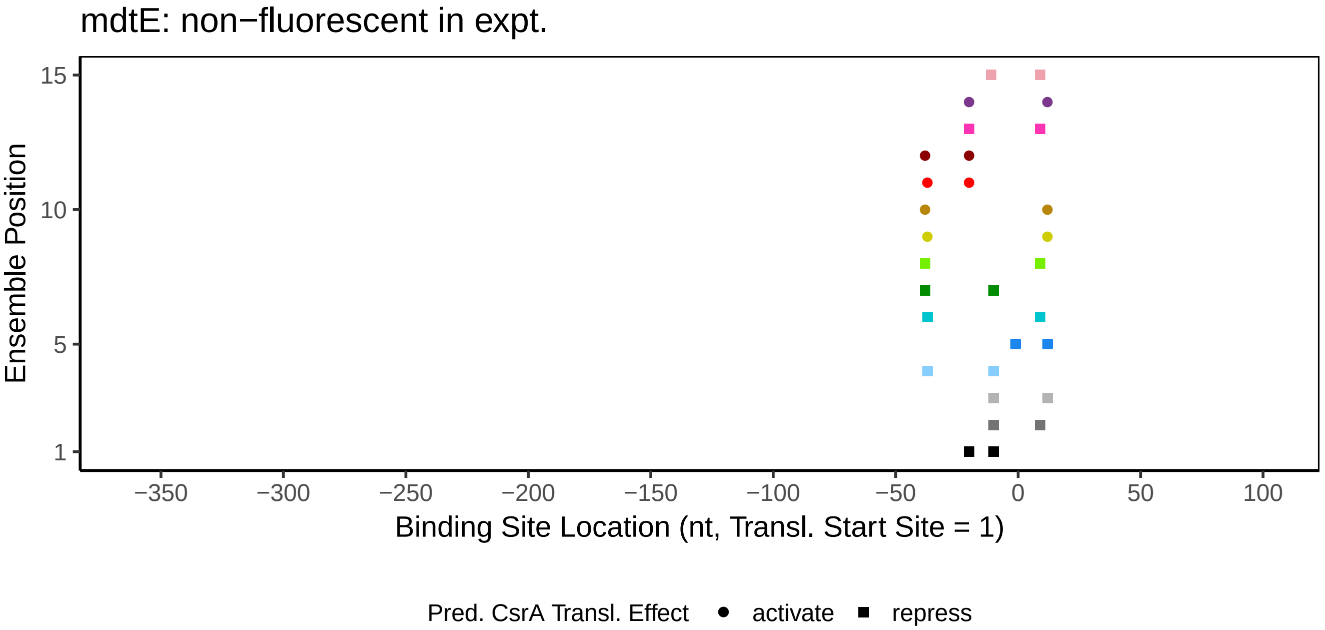
mdtE: non−fluorescent in expt.
Ensemble Position
Binding Site Location (nt, Transl. Start Site = 1)
Pred. CsrA Transl. Effect
activate
repress

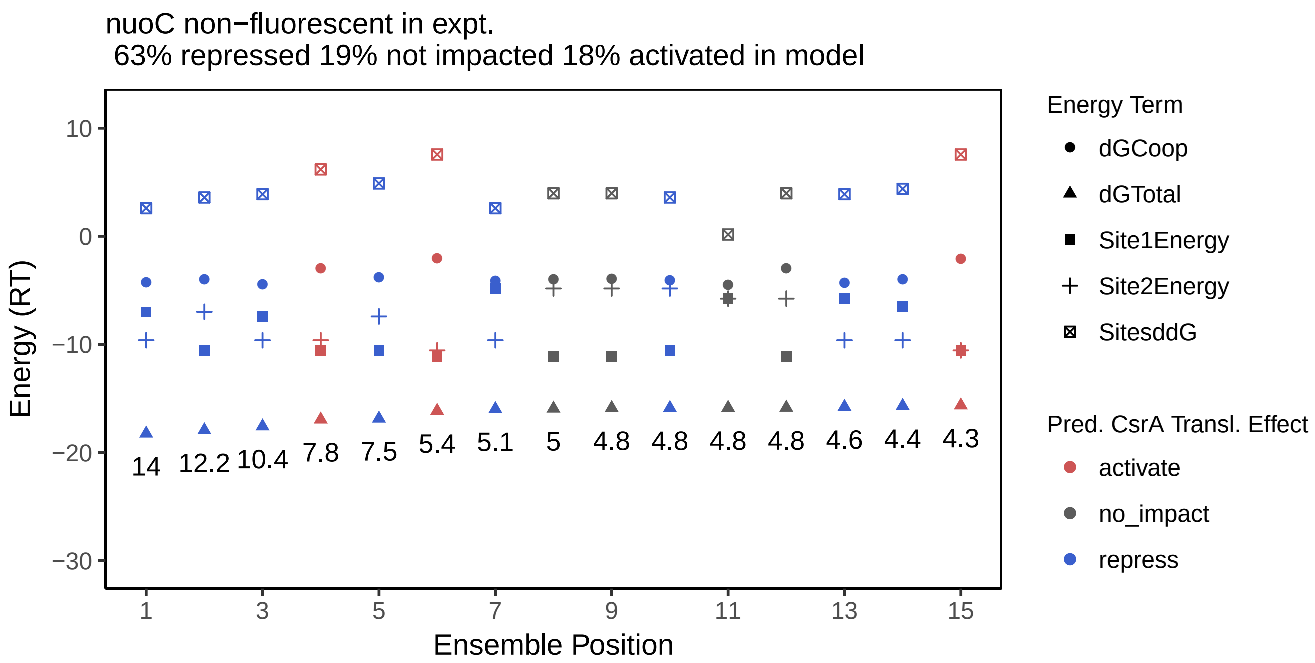

nuoC non−fluorescent in expt.
63% repressed 19% not impacted 18% activated in model
Energy (RT)
Ensemble Position
14
12.2
10.4
7.8
7.5
5.4
5.1
5
4.8
4.8
4.8
4.8
4.6
4.4
4.3
Energy Term
dGCoop
dGTotal
Site1Energy
Site2Energy
SitesddG
Pred. CsrA Transl. Effect
activate
no_impact
repress

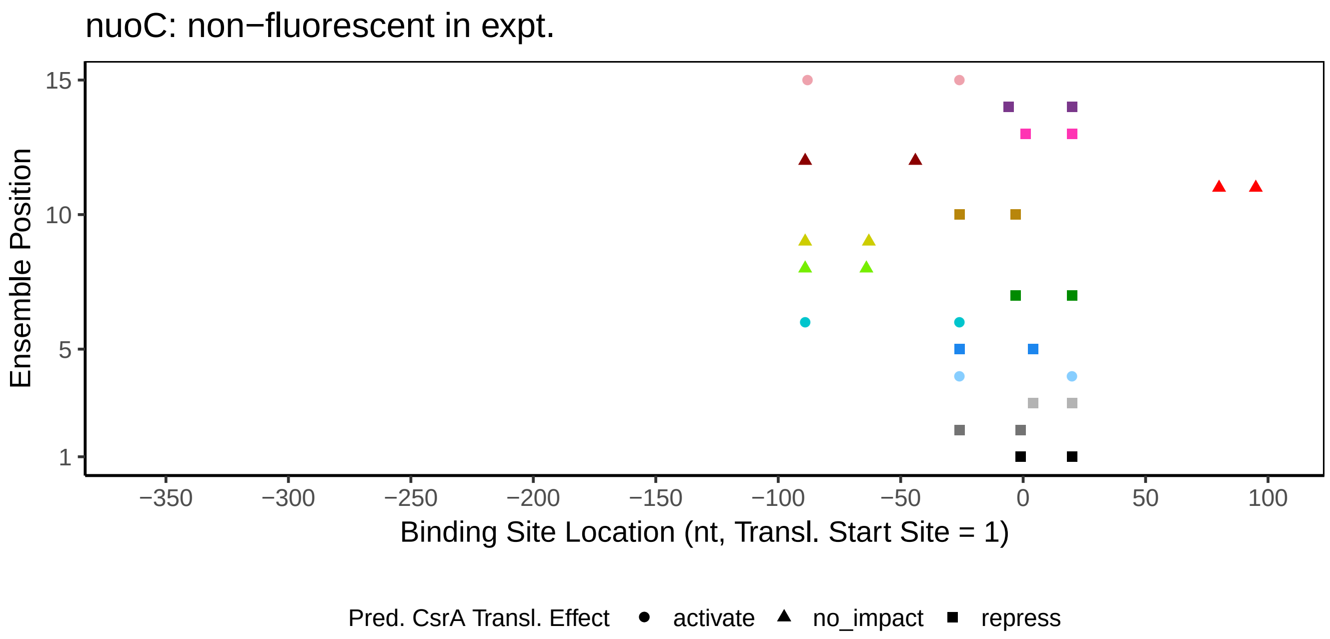

nuoC: non−fluorescent in expt.
Ensemble Position
Binding Site Location (nt, Transl. Start Site = 1)
Pred. CsrA Transl. Effect
activate
no_impact
repress

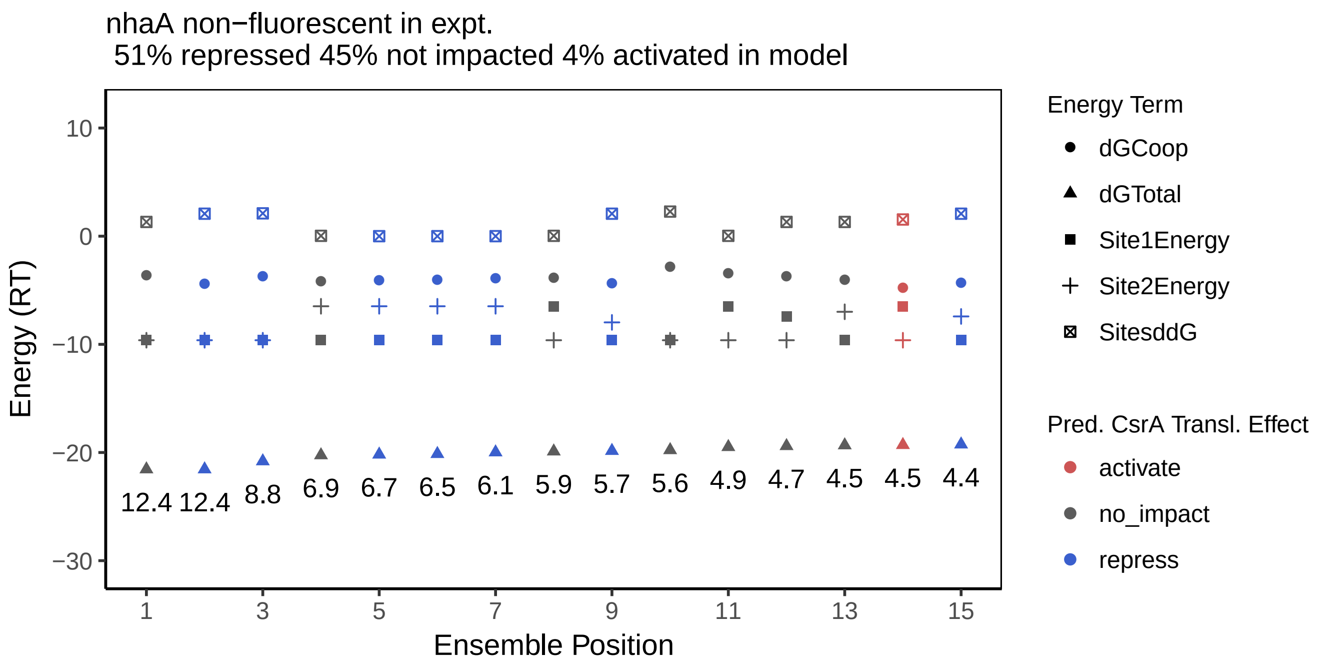

nhaA non−fluorescent in expt.
51% repressed 45% not impacted 4% activated in model
Energy (RT)
10
0
−10
−20
−30
12.4 12.4 8.8 6.9 6.7 6.5 6.1 5.9 5.7 5.6 4.9 4.7 4.5 4.5 4.4
1 3 5 7 9 11 13 15
Ensemble Position
Energy Term
dGCoop
dGTotal
Site1Energy
Site2Energy
SitesddG
Pred. CsrA Transl. Effect
activate
no_impact
repress

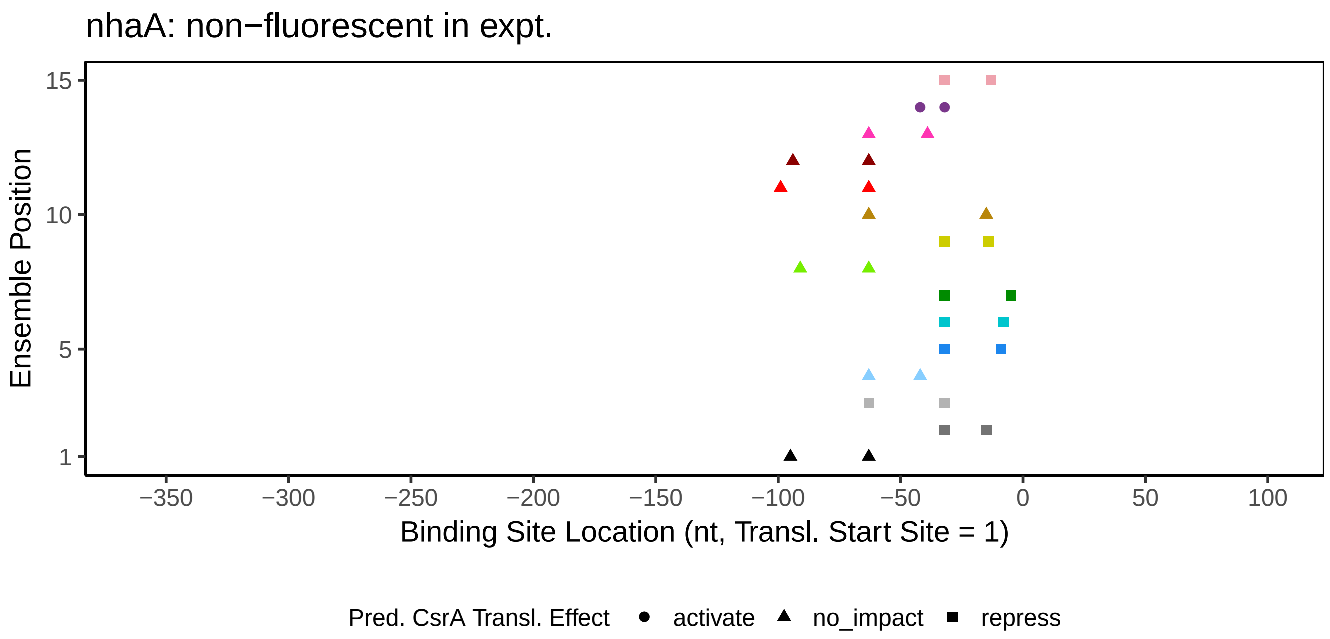

nhaA: non−fluorescent in expt.
Ensemble Position
15
10
5
1
−350 −300 −250 −200 −150 −100 −50 0 50 100
Binding Site Location (nt, Transl. Start Site = 1)
Pred. CsrA Transl. Effect
activate
no_impact
repress

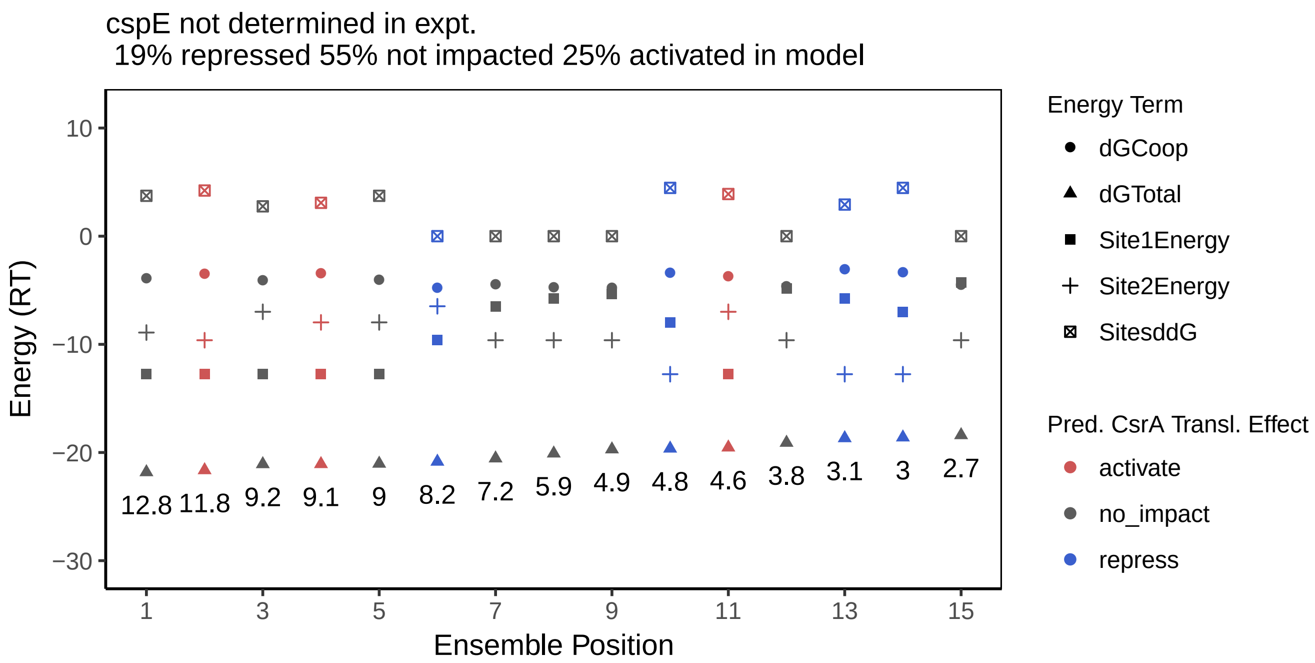
cspE not determined in expt.
19% repressed 55% not impacted 25% activated in model
Energy (RT)
10
0
−10
−20
−30
12.8 11.8 9.2 9.1 9 8.2 7.2 5.9 4.9 4.8 4.6 3.8 3.1 3 2.7
1
3
5
7
9
11
13
15
Ensemble Position
Energy Term
dGCoop
dGTotal
Site1Energy
Site2Energy
SitesddG
Pred. CsrA Transl. Effect
activate
no_impact
repress

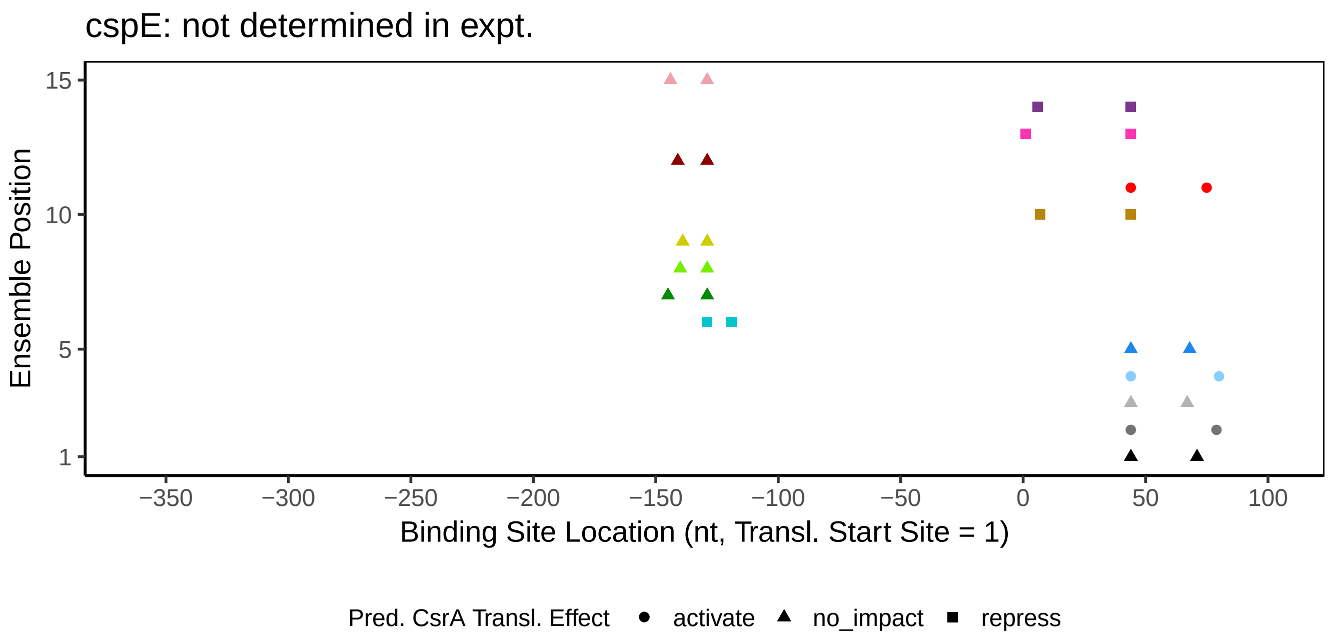
cspE: not determined in expt.
Ensemble Position
15
10
5
1
−350
−300
−250
−200
−150
−100
−50
0
50
100
Binding Site Location (nt, Transl. Start Site = 1)
Pred. CsrA Transl. Effect
activate
no_impact
repress

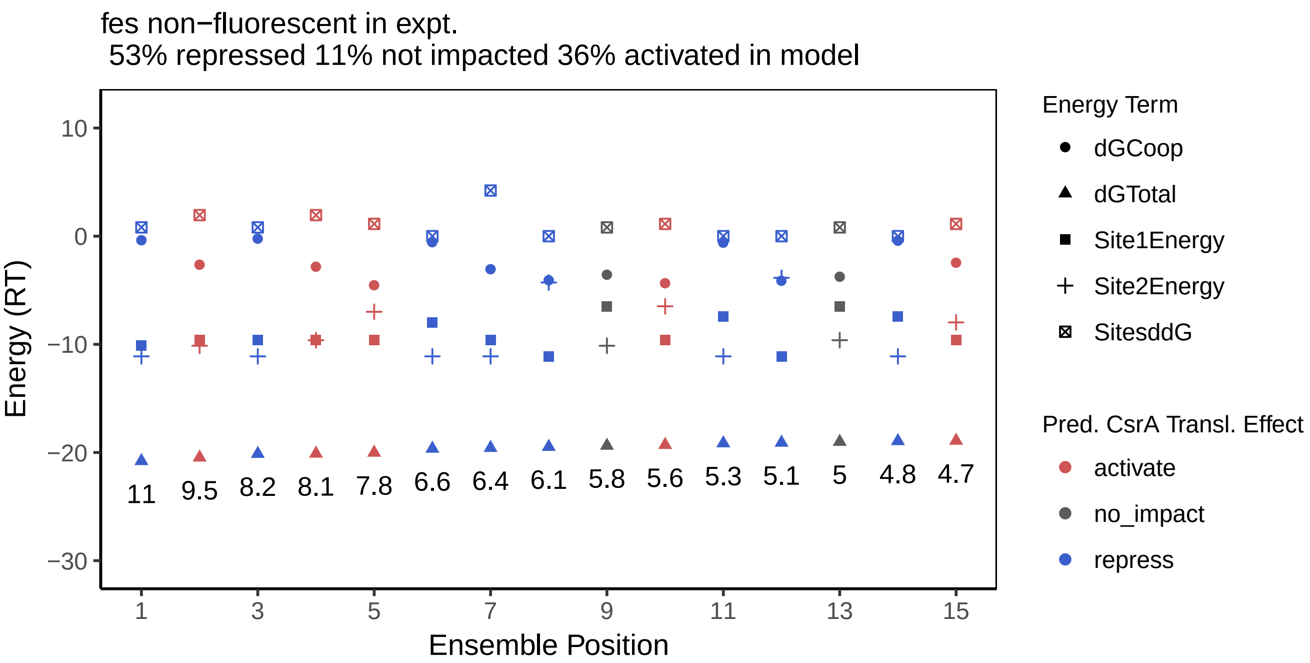
fes non−fluorescent in expt.
53% repressed 11% not impacted 36% activated in model
Energy (RT)
10
0
−10
−20
−30
11 9.5 8.2 8.1 7.8 6.6 6.4 6.1 5.8 5.6 5.3 5.1 5 4.8 4.7
1 3 5 7 9 11 13 15
Ensemble Position
Energy Term
dGCoop
dGTotal
Site1Energy
Site2Energy
SitesddG
Pred. CsrA Transl. Effect
activate
no_impact
repress

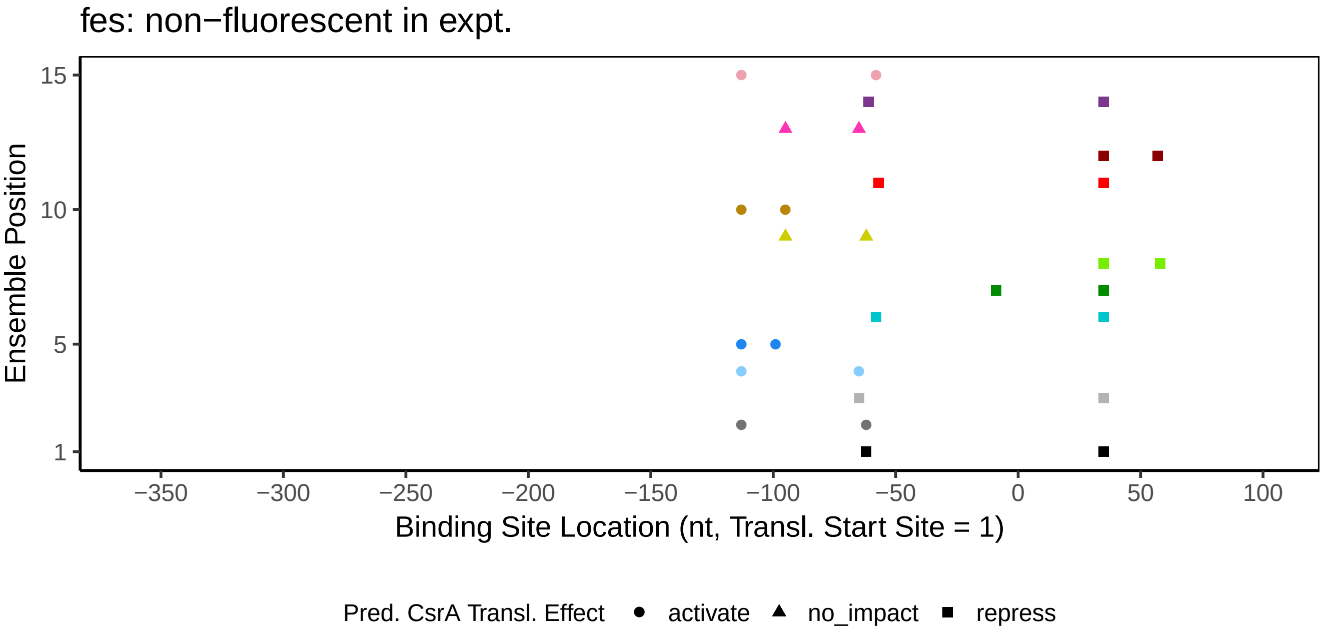
fes: non−fluorescent in expt.
Ensemble Position
15
10
5
1
−350 −300 −250 −200 −150 −100 −50 0 50 100
Binding Site Location (nt, Transl. Start Site = 1)
Pred. CsrA Transl. Effect
activate
no_impact
repress

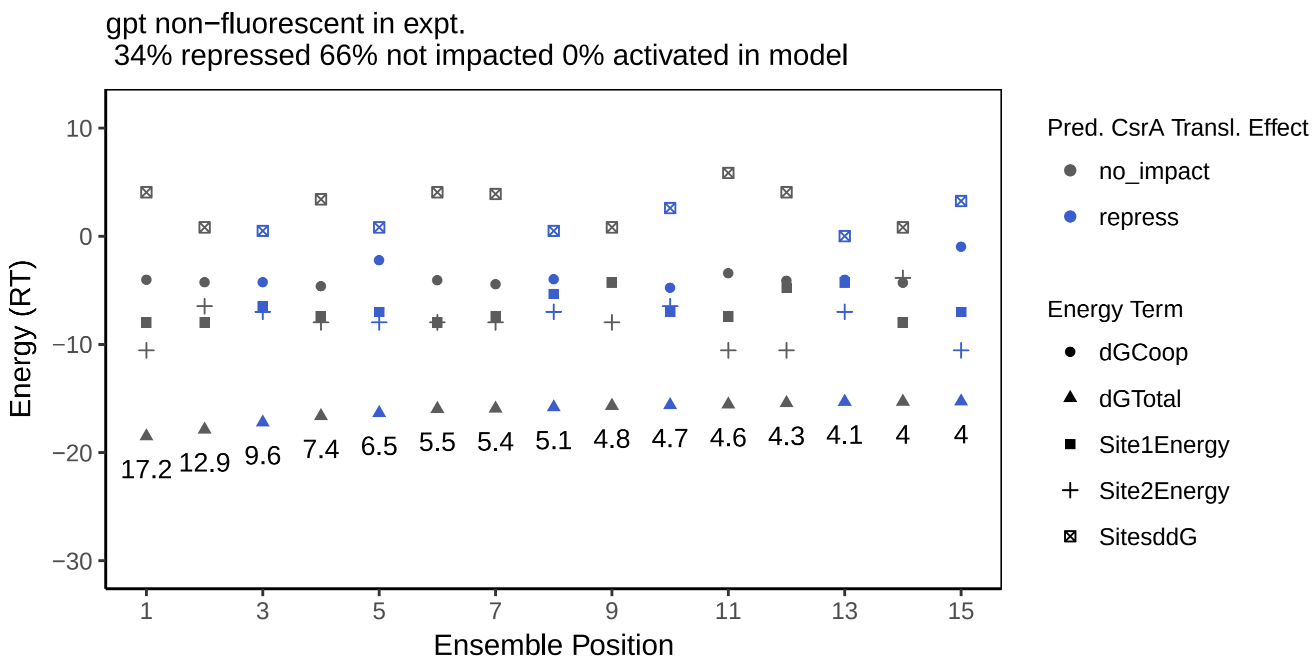
gpt non−fluorescent in expt.
34% repressed 66% not impacted 0% activated in model
Energy (RT)
Ensemble Position
17.2 12.9 9.6 7.4 6.5 5.5 5.4 5.1 4.8 4.7 4.6 4.3 4.1 4 4
Pred. CsrA Transl. Effect
no_impact
repress
Energy Term
dGCoop
dGTotal
Site1Energy
Site2Energy
SitesddG

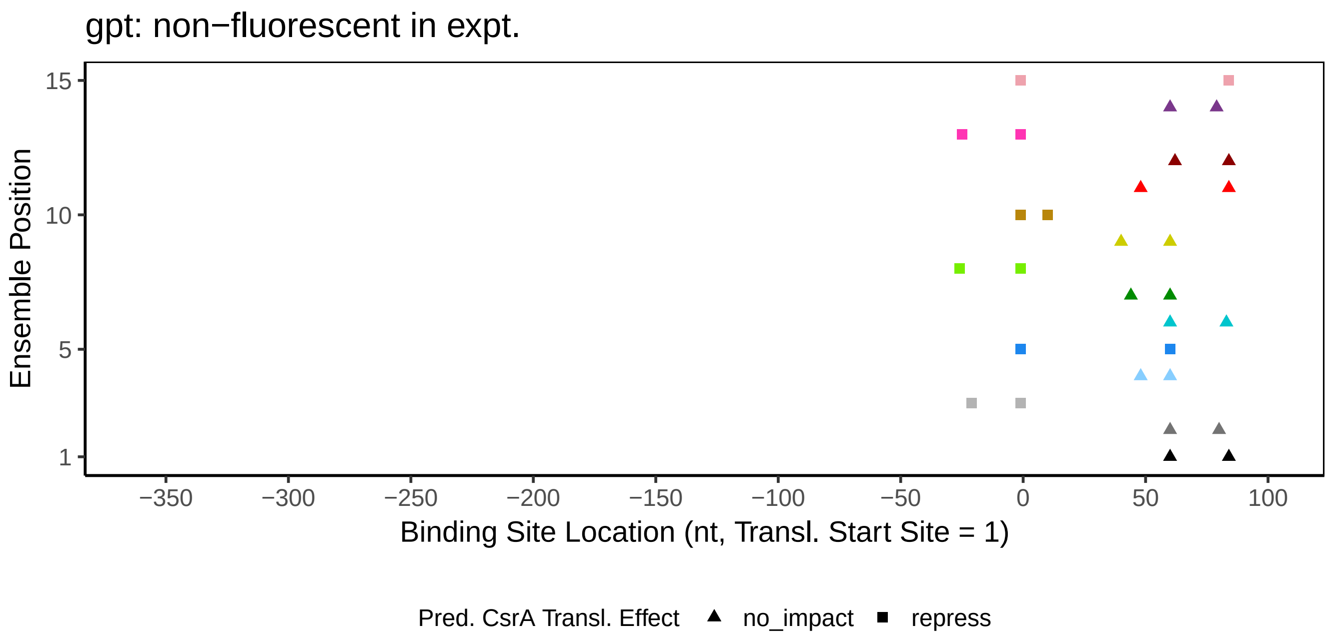
gpt: non−fluorescent in expt.
Ensemble Position
Binding Site Location (nt, Transl. Start Site = 1)
Pred. CsrA Transl. Effect
no_impact
repress

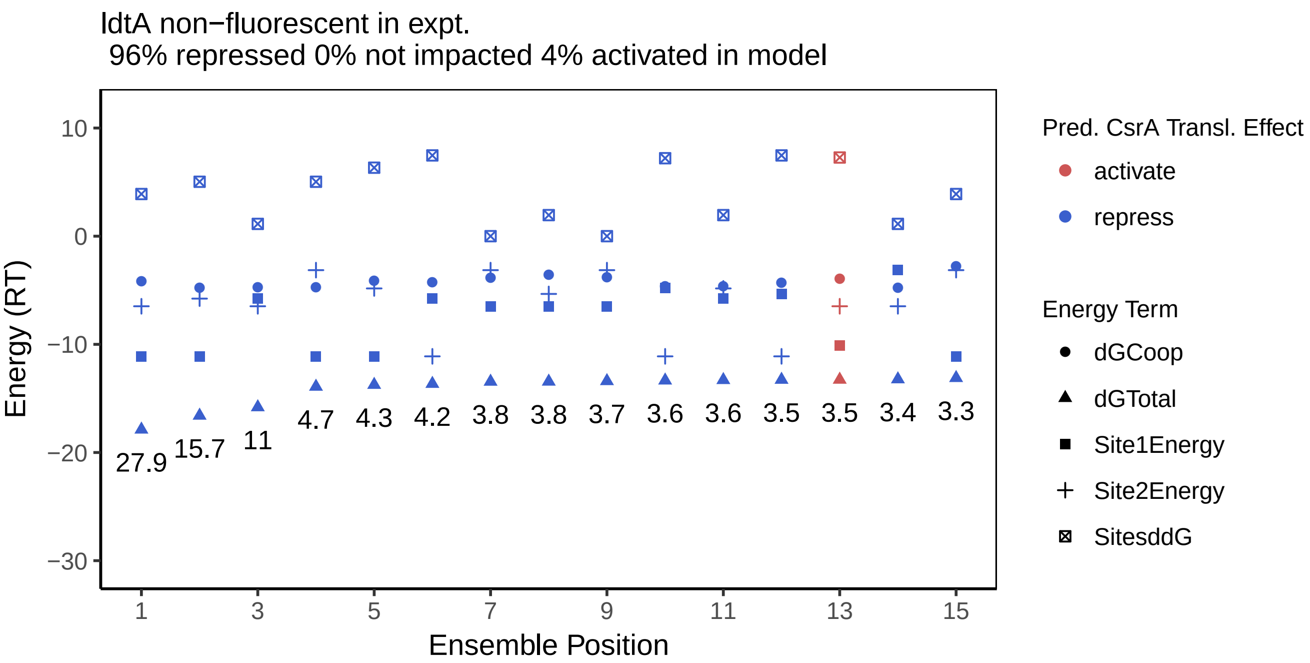

ldtA non−fluorescent in expt.
96% repressed 0% not impacted 4% activated in model
Energy (RT)
Ensemble Position
27.9
15.7
11
4.7
4.3
4.2
3.8
3.8
3.7
3.6
3.6
3.5
3.5
3.4
3.3
Pred. CsrA Transl. Effect
activate
repress
Energy Term
dGCoop
dGTotal
Site1Energy
Site2Energy
SitesddG

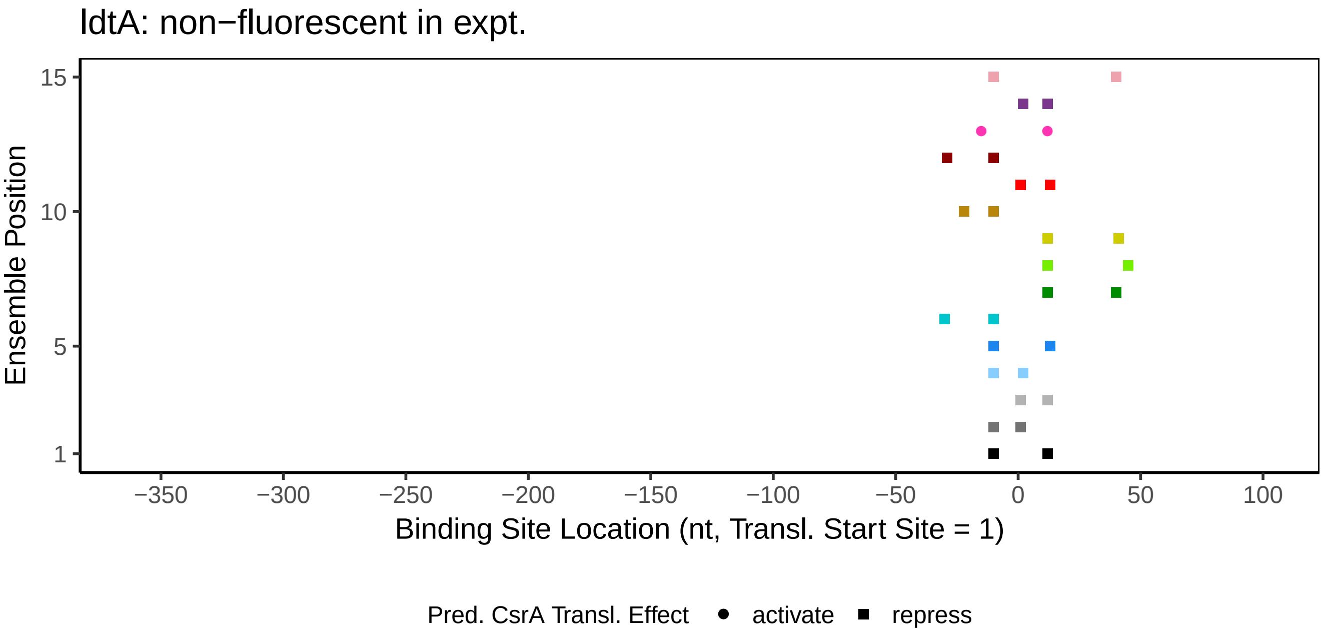

ldtA: non−fluorescent in expt.
Ensemble Position
Binding Site Location (nt, Transl. Start Site = 1)
Pred. CsrA Transl. Effect
activate
repress

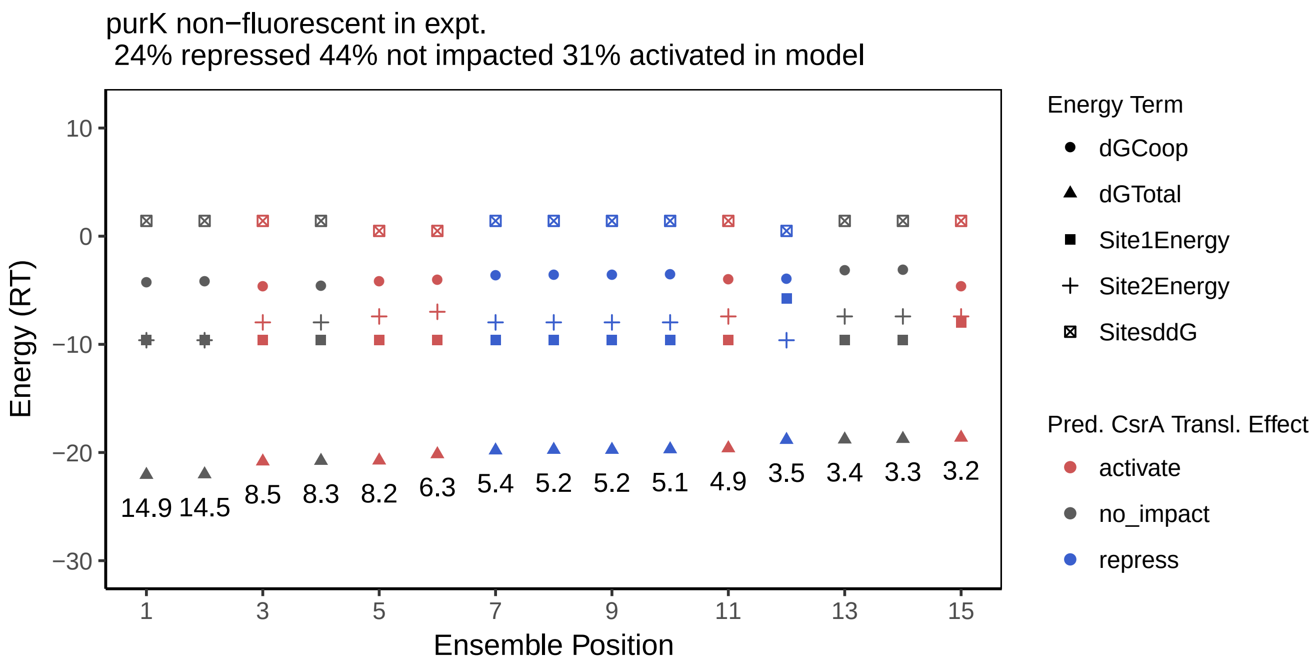
purK non−fluorescent in expt.
24% repressed 44% not impacted 31% activated in model
Energy (RT)
10
0
−10
−20
−30
14.9 14.5 8.5 8.3 8.2 6.3 5.4 5.2 5.2 5.1 4.9 3.5 3.4 3.3 3.2
1
3
5
7
9
11
13
15
Ensemble Position
Energy Term
dGCoop
dGTotal
Site1Energy
Site2Energy
SitesddG
Pred. CsrA Transl. Effect
activate
no_impact
repress

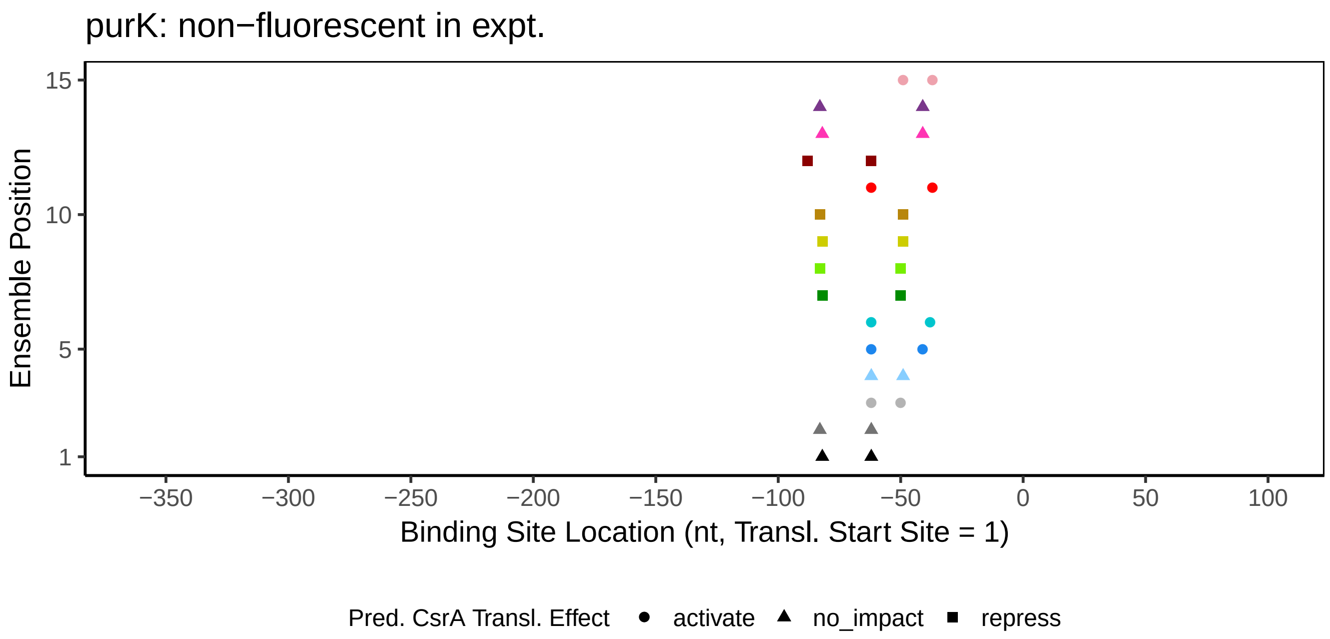
purK: non−fluorescent in expt.
Ensemble Position
15
10
5
1
−350
−300
−250
−200
−150
−100
−50
0
50
100
Binding Site Location (nt, Transl. Start Site = 1)
Pred. CsrA Transl. Effect
activate
no_impact
repress

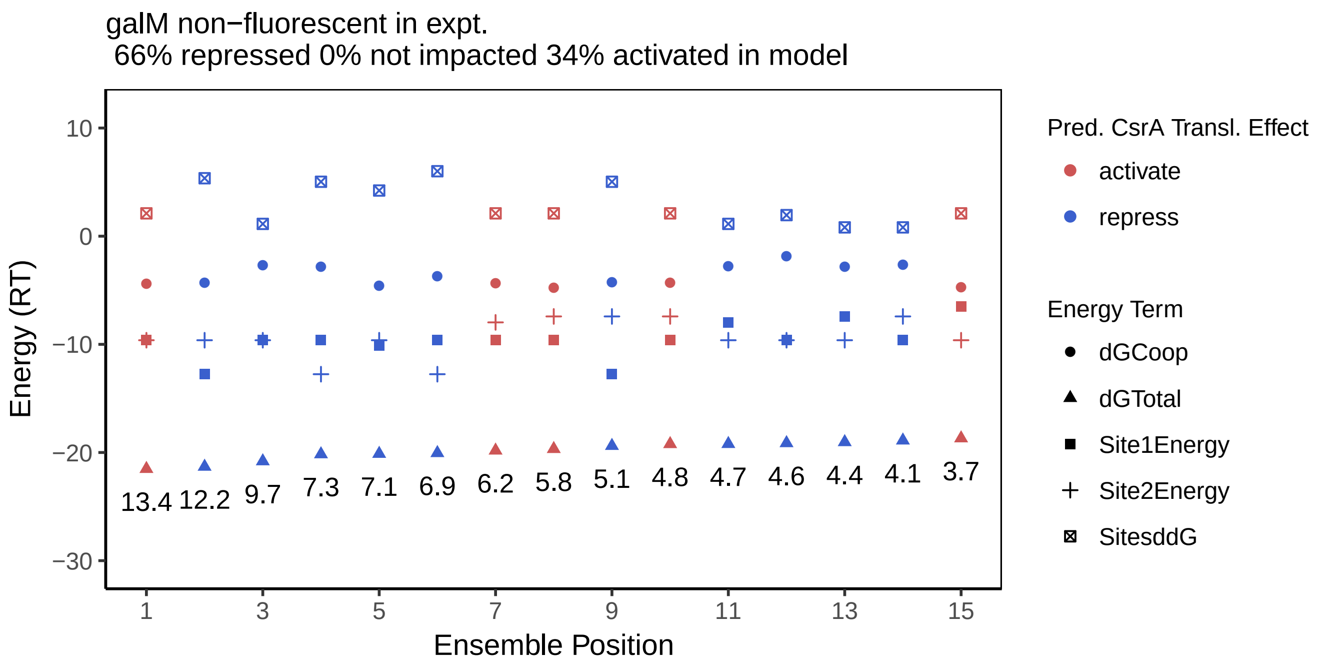
galM non−fluorescent in expt.
66% repressed 0% not impacted 34% activated in model
Energy (RT)
10
0
−10
−20
−30
13.4 12.2 9.7 7.3 7.1 6.9 6.2 5.8 5.1 4.8 4.7 4.6 4.4 4.1 3.7
1 3 5 7 9 11 13 15
Ensemble Position
Pred. CsrA Transl. Effect
activate
repress
Energy Term
dGCoop
dGTotal
Site1Energy
Site2Energy
SitesddG

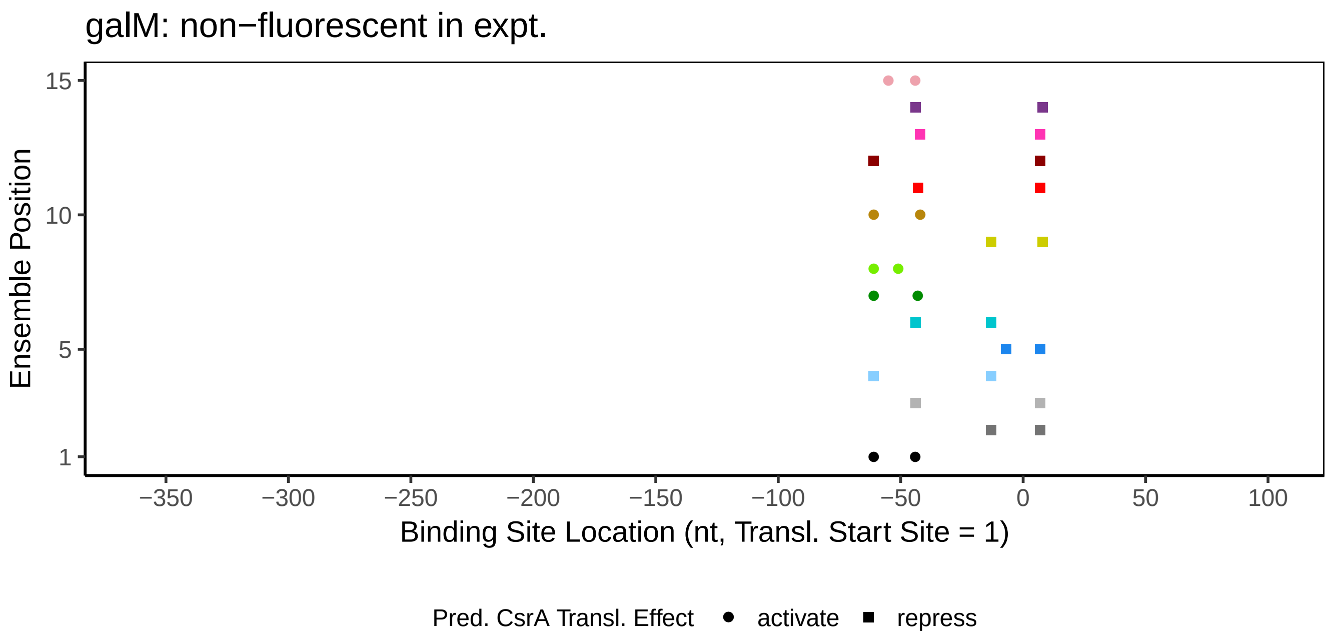
galM: non−fluorescent in expt.
Ensemble Position
15
10
5
1
−350 −300 −250 −200 −150 −100 −50 0 50 100
Binding Site Location (nt, Transl. Start Site = 1)
Pred. CsrA Transl. Effect
activate
repress

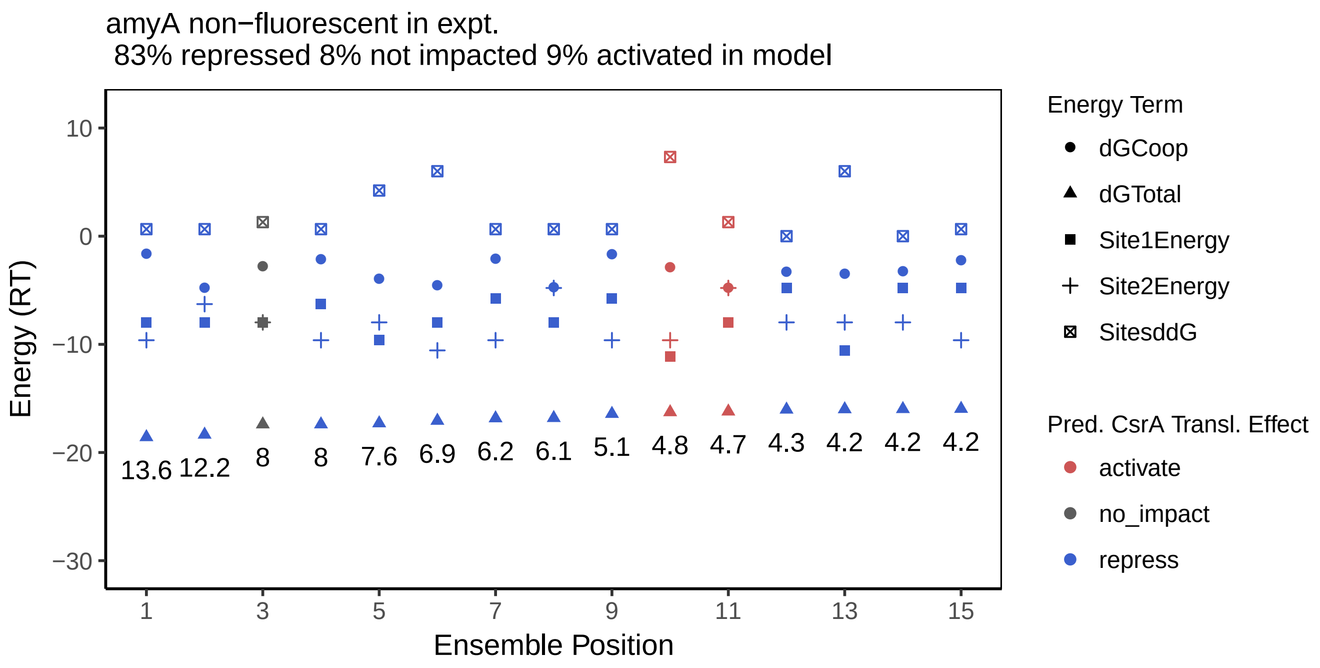
amyA non−fluorescent in expt.
83% repressed 8% not impacted 9% activated in model
Energy (RT)
10
0
−10
−20
−30
13.6 12.2 8 8 7.6 6.9 6.2 6.1 5.1 4.8 4.7 4.3 4.2 4.2 4.2
1 3 5 7 9 11 13 15
Ensemble Position
Energy Term
dGCoop
dGTotal
Site1Energy
Site2Energy
SitesddG
Pred. CsrA Transl. Effect
activate
no_impact
repress

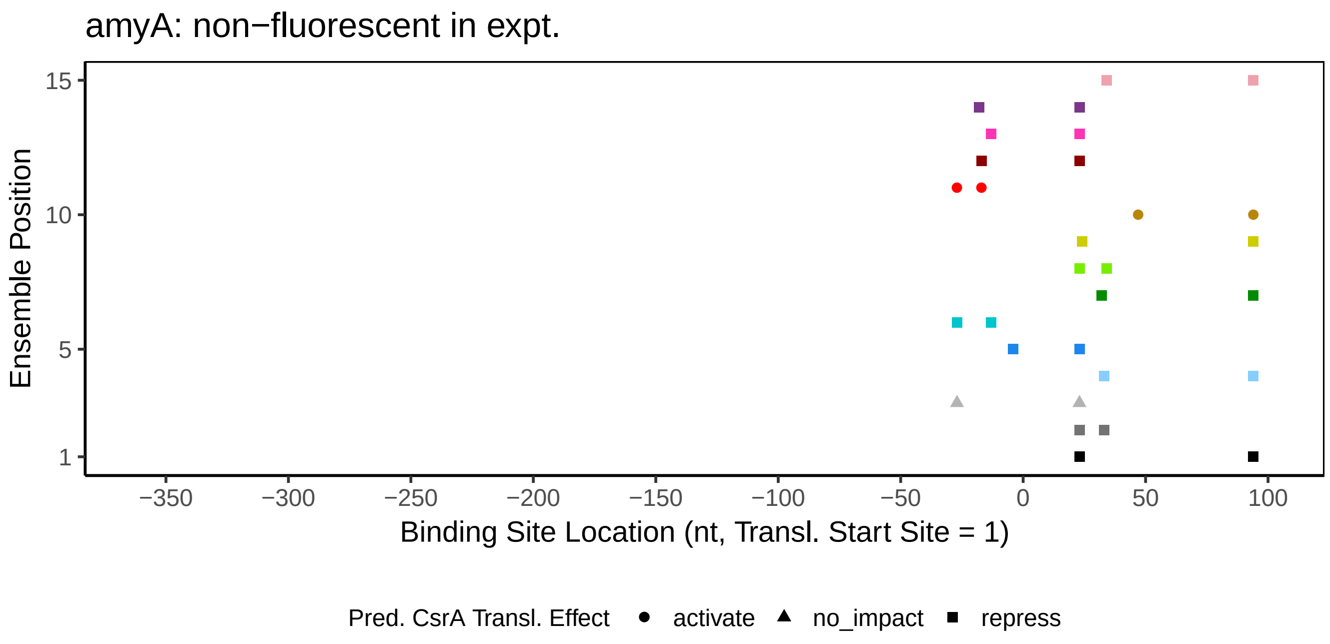
amyA: non−fluorescent in expt.
Ensemble Position
15
10
5
1
−350 −300 −250 −200 −150 −100 −50 0 50 100
Binding Site Location (nt, Transl. Start Site = 1)
Pred. CsrA Transl. Effect
activate
no_impact
repress

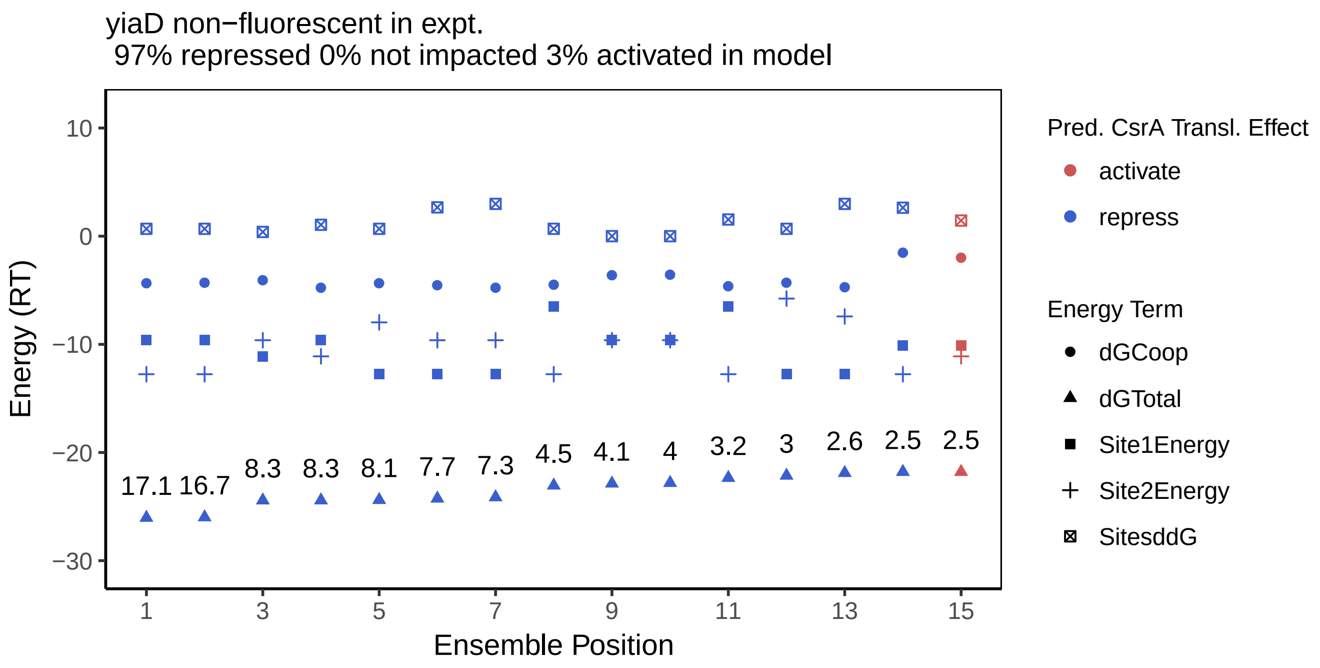
yiaD non−fluorescent in expt.
97% repressed 0% not impacted 3% activated in model
Energy (RT)
10
0
−10
−20
−30
17.1 16.7 8.3 8.3 8.1 7.7 7.3 4.5 4.1 4 3.2 3 2.6 2.5 2.5
1
3
5
7
9
11
13
15
Ensemble Position
Pred. CsrA Transl. Effect
activate
repress
Energy Term
dGCoop
dGTotal
Site1Energy
Site2Energy
SitesddG

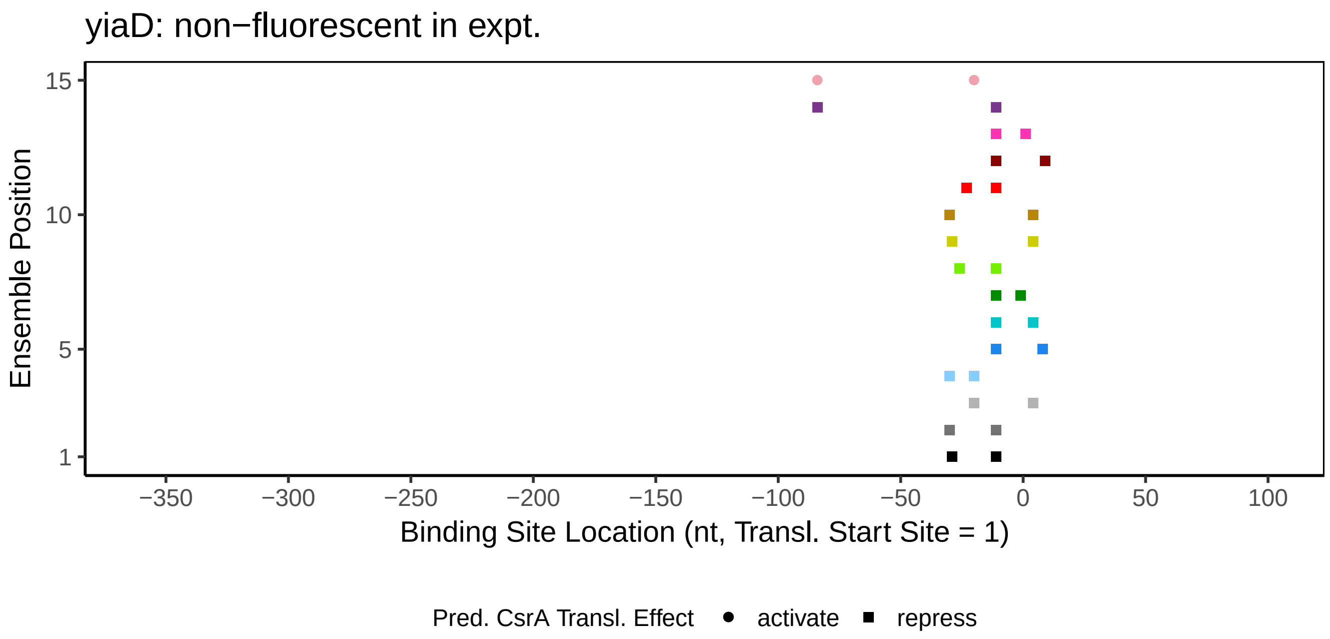
yiaD: non−fluorescent in expt.
Ensemble Position
15
10
5
1
−350
−300
−250
−200
−150
−100
−50
0
50
100
Binding Site Location (nt, Transl. Start Site = 1)
Pred. CsrA Transl. Effect
activate
repress

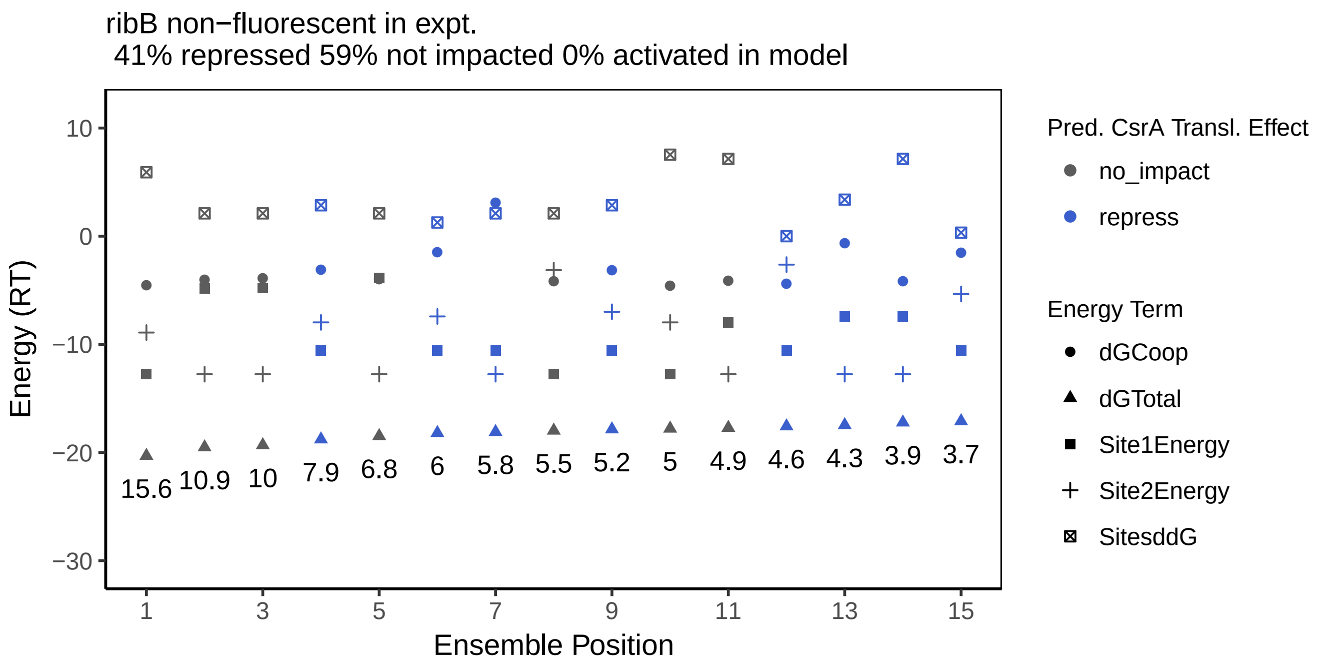
ribB non−fluorescent in expt.
41% repressed 59% not impacted 0% activated in model
Energy (RT)
Ensemble Position
15.6 10.9 10 7.9 6.8 6 5.8 5.5 5.2 5 4.9 4.6 4.3 3.9 3.7
Pred. CsrA Transl. Effect
no_impact
repress
Energy Term
dGCoop
dGTotal
Site1Energy
Site2Energy
SitesddG

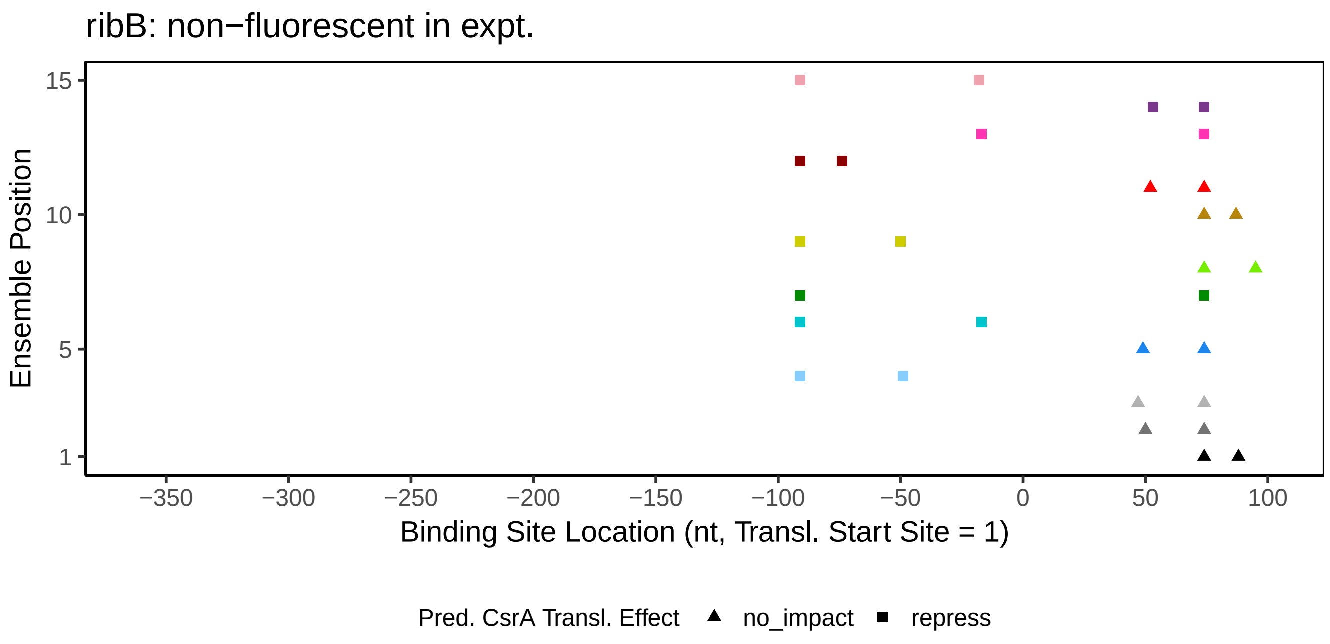
ribB: non−fluorescent in expt.
Ensemble Position
Binding Site Location (nt, Transl. Start Site = 1)
Pred. CsrA Transl. Effect
no_impact
repress

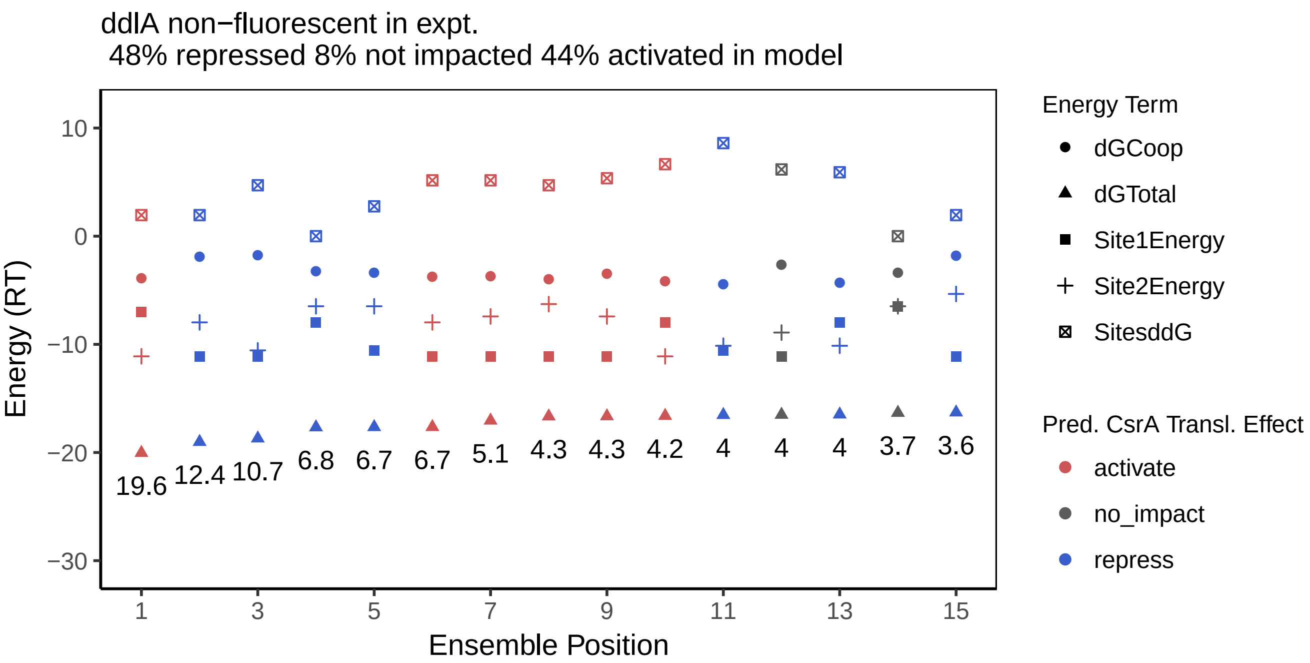
ddlA non−fluorescent in expt.
48% repressed 8% not impacted 44% activated in model
Energy (RT)
10
0
−10
−20
−30
19.6
12.4
10.7
6.8
6.7
6.7
5.1
4.3
4.3
4.2
4
4
4
3.7
3.6
1
3
5
7
9
11
13
15
Ensemble Position
Energy Term
dGCoop
dGTotal
Site1Energy
Site2Energy
SitesddG
Pred. CsrA Transl. Effect
activate
no_impact
repress

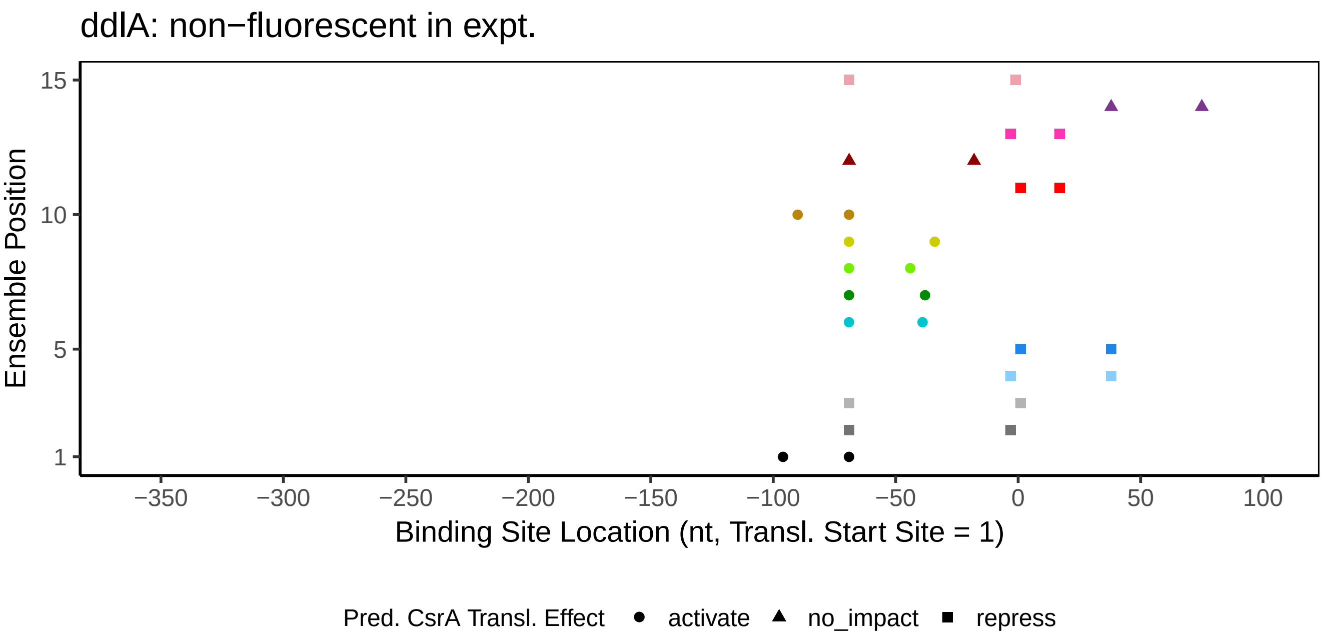
ddlA: non−fluorescent in expt.
Ensemble Position
15
10
5
1
−350
−300
−250
−200
−150
−100
−50
0
50
100
Binding Site Location (nt, Transl. Start Site = 1)
Pred. CsrA Transl. Effect
activate
no_impact
repress

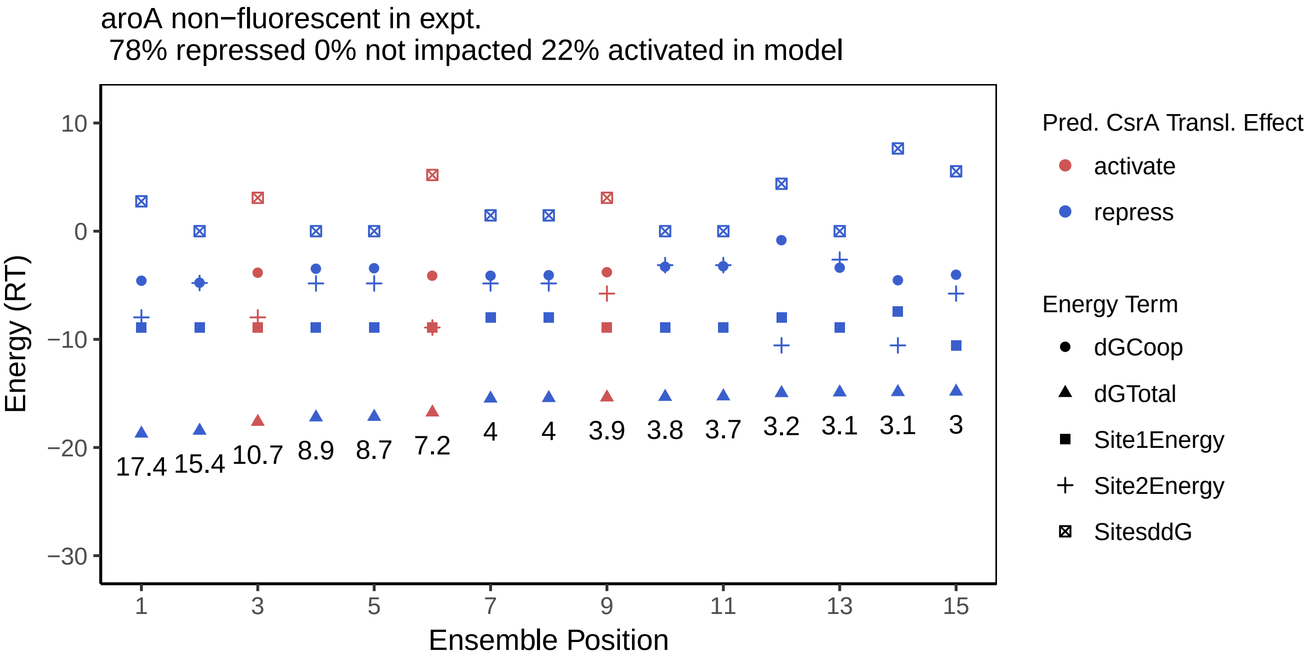

aroA non−fluorescent in expt.
78% repressed 0% not impacted 22% activated in model
Energy (RT)
Ensemble Position
17.4 15.4 10.7 8.9 8.7 7.2 4 4 3.9 3.8 3.7 3.2 3.1 3.1 3
Pred. CsrA Transl. Effect
activate
repress
Energy Term
dGCoop
dGTotal
Site1Energy
Site2Energy
SitesddG

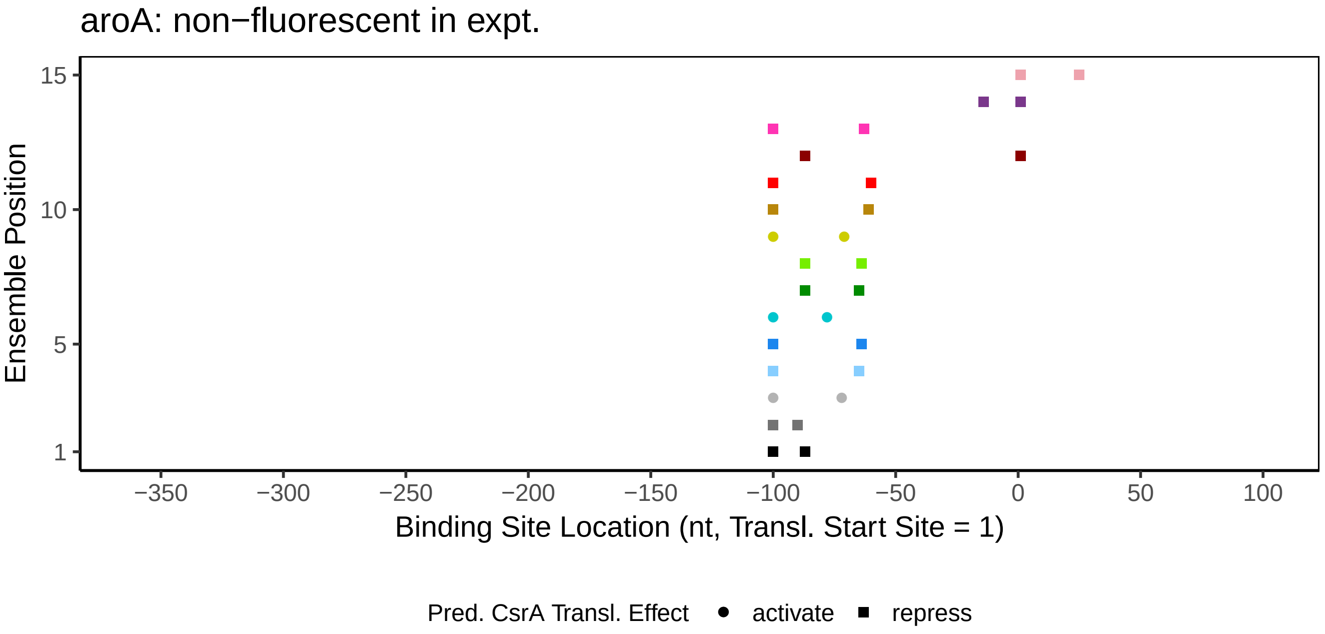

aroA: non−fluorescent in expt.
Ensemble Position
Binding Site Location (nt, Transl. Start Site = 1)
Pred. CsrA Transl. Effect
activate
repress

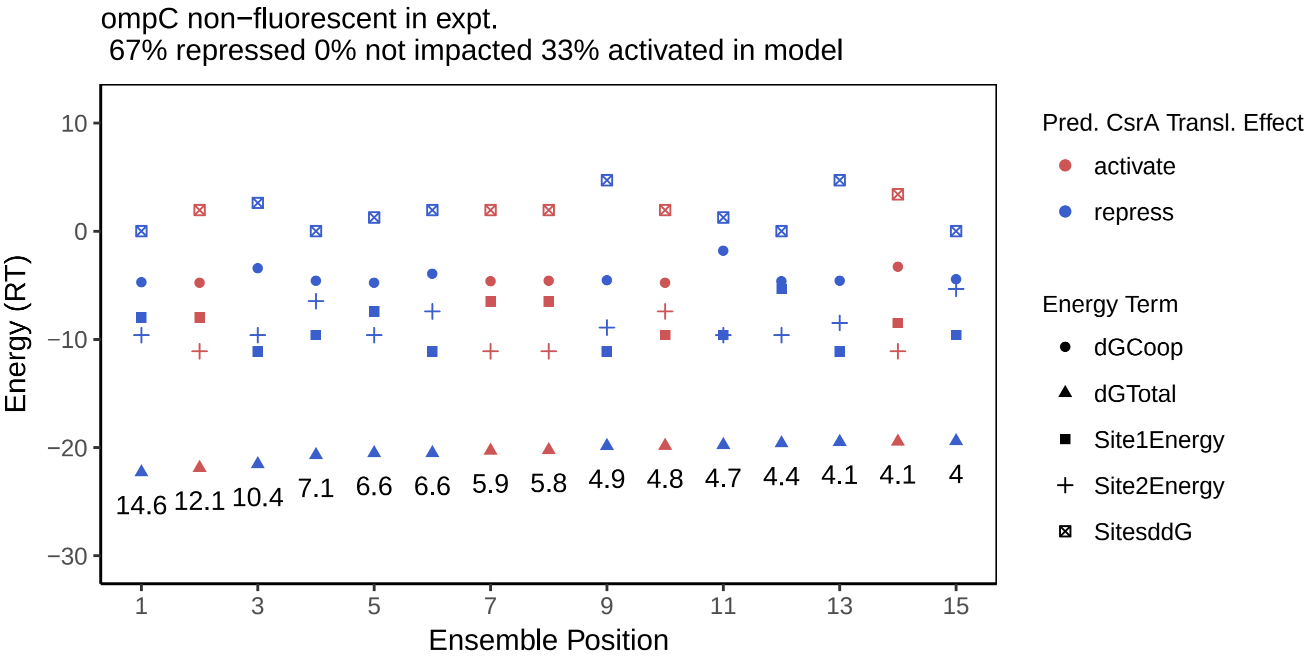
ompC non−fluorescent in expt.
67% repressed 0% not impacted 33% activated in model
Energy (RT)
Ensemble Position
14.6 12.1 10.4 7.1 6.6 6.6 5.9 5.8 4.9 4.8 4.7 4.4 4.1 4.1 4
Pred. CsrA Transl. Effect
activate
repress
Energy Term
dGCoop
dGTotal
Site1Energy
Site2Energy
SitesddG

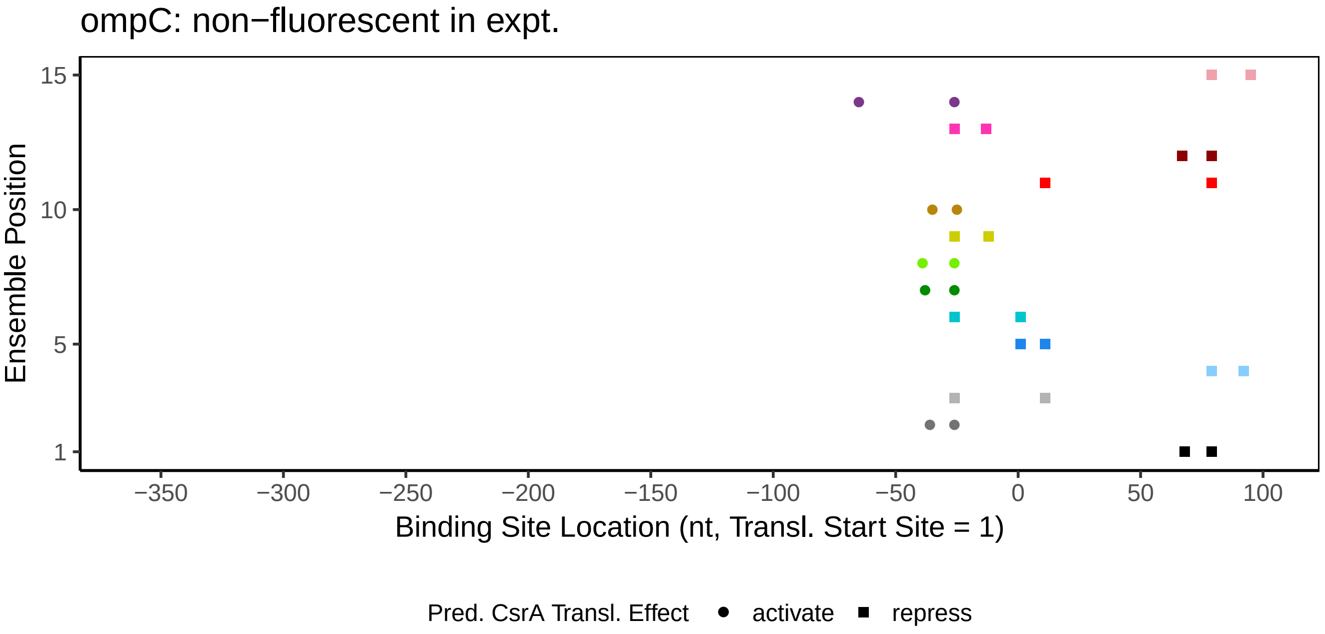
ompC: non−fluorescent in expt.
Ensemble Position
Binding Site Location (nt, Transl. Start Site = 1)
Pred. CsrA Transl. Effect
activate
repress

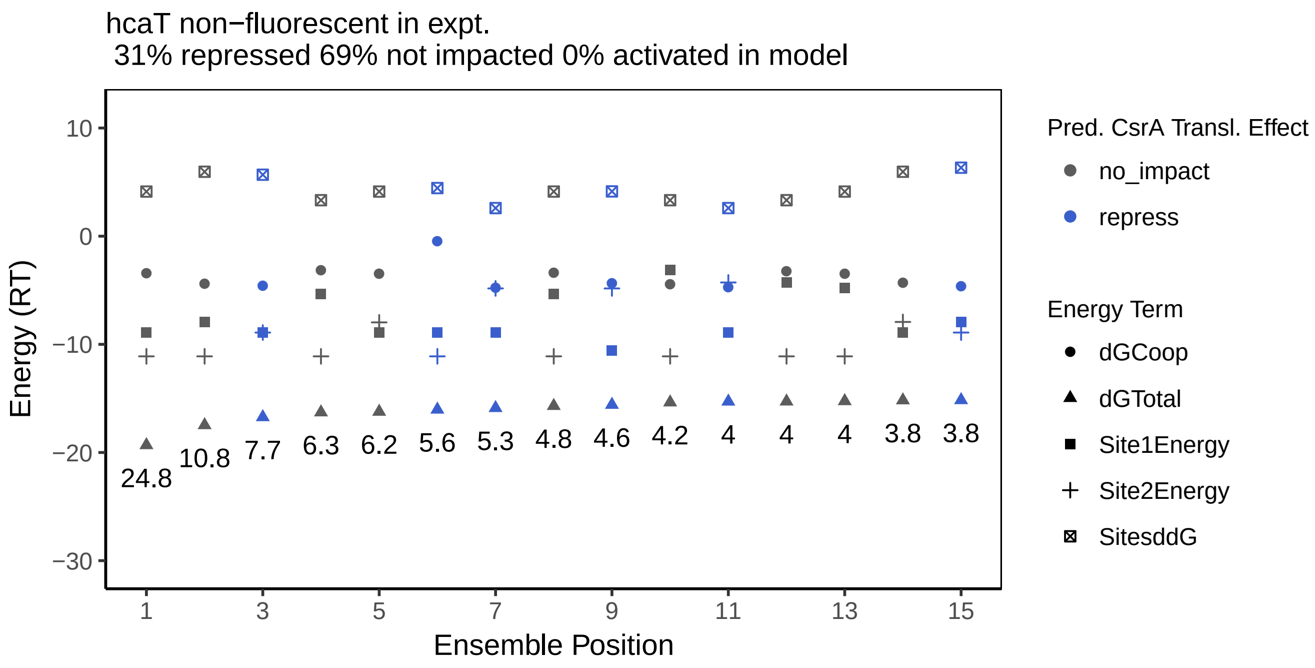

hcaT non−fluorescent in expt.
31% repressed 69% not impacted 0% activated in model
Energy (RT)
10
0
−10
−20
−30
24.8
10.8
7.7
6.3
6.2
5.6
5.3
4.8
4.6
4.2
4
4
4
3.8
3.8
1
3
5
7
9
11
13
15
Ensemble Position
Pred. CsrA Transl. Effect
no_impact
repress
Energy Term
dGCoop
dGTotal
Site1Energy
Site2Energy
SitesddG

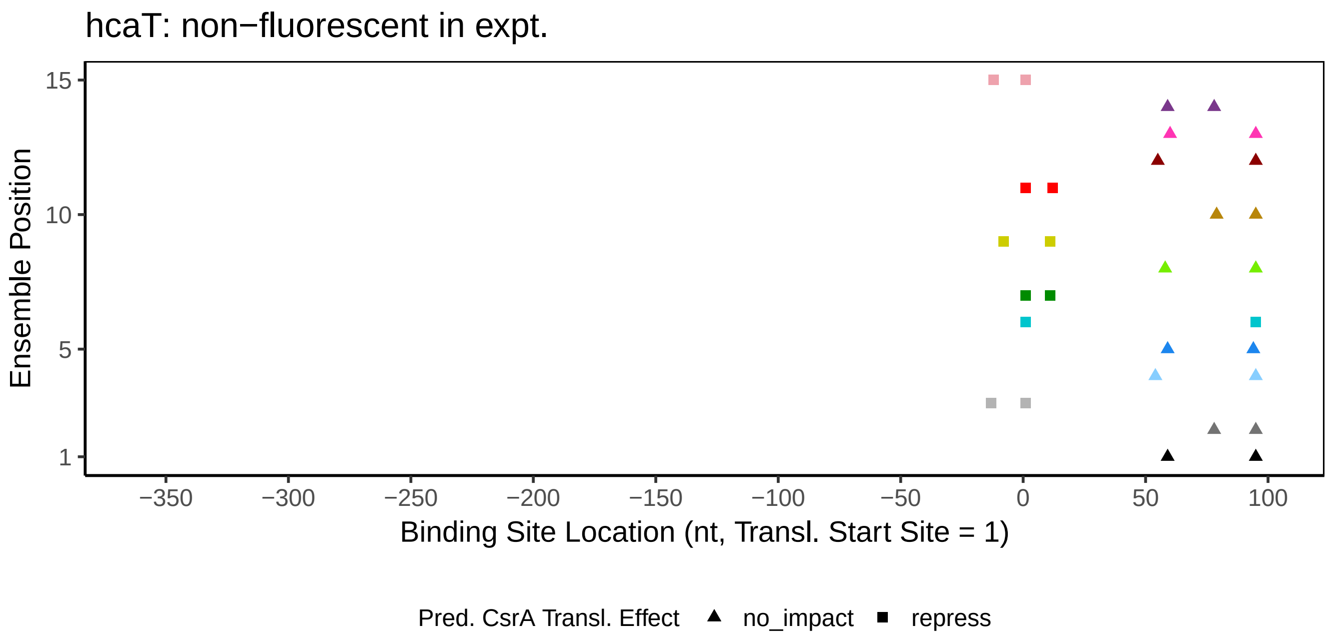

hcaT: non−fluorescent in expt.
Ensemble Position
15
10
5
1
−350
−300
−250
−200
−150
−100
−50
0
50
100
Binding Site Location (nt, Transl. Start Site = 1)
Pred. CsrA Transl. Effect
no_impact
repress

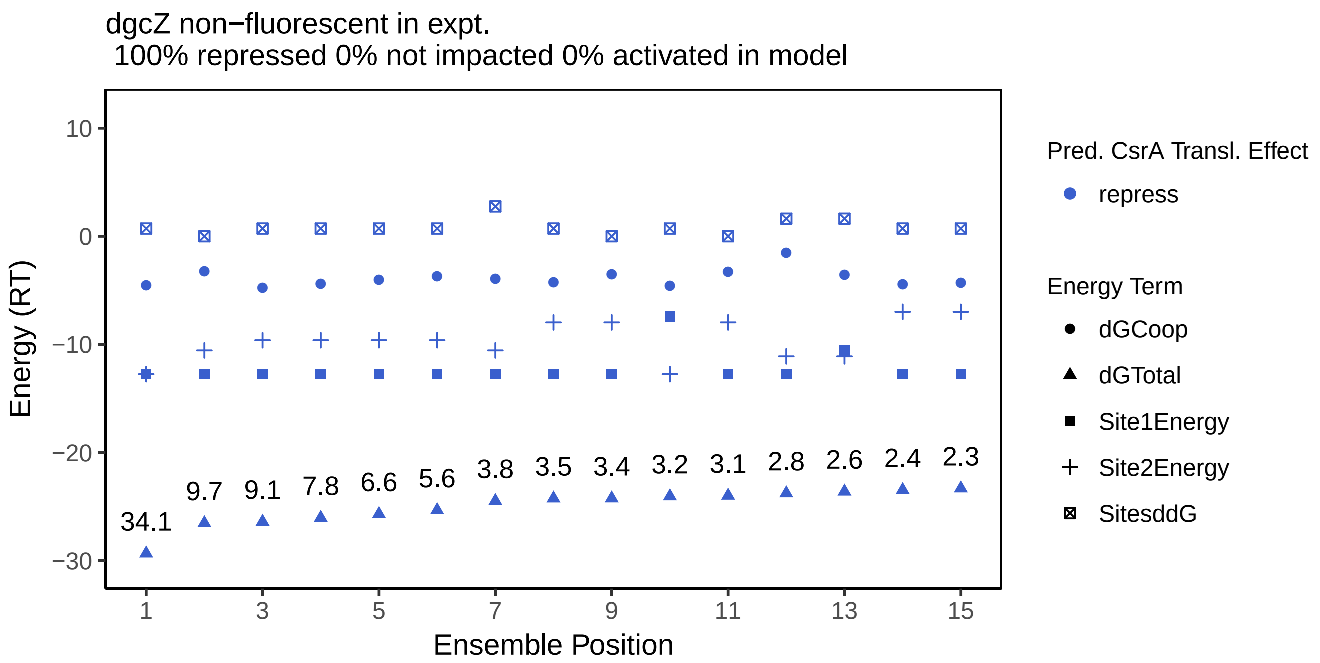

dgcZ non−fluorescent in expt.
100% repressed 0% not impacted 0% activated in model
Energy (RT)
Ensemble Position
34.1
9.7
9.1
7.8
6.6
5.6
3.8
3.5
3.4
3.2
3.1
2.8
2.6
2.4
2.3
Pred. CsrA Transl. Effect
repress
Energy Term
dGCoop
dGTotal
Site1Energy
Site2Energy
SitesddG

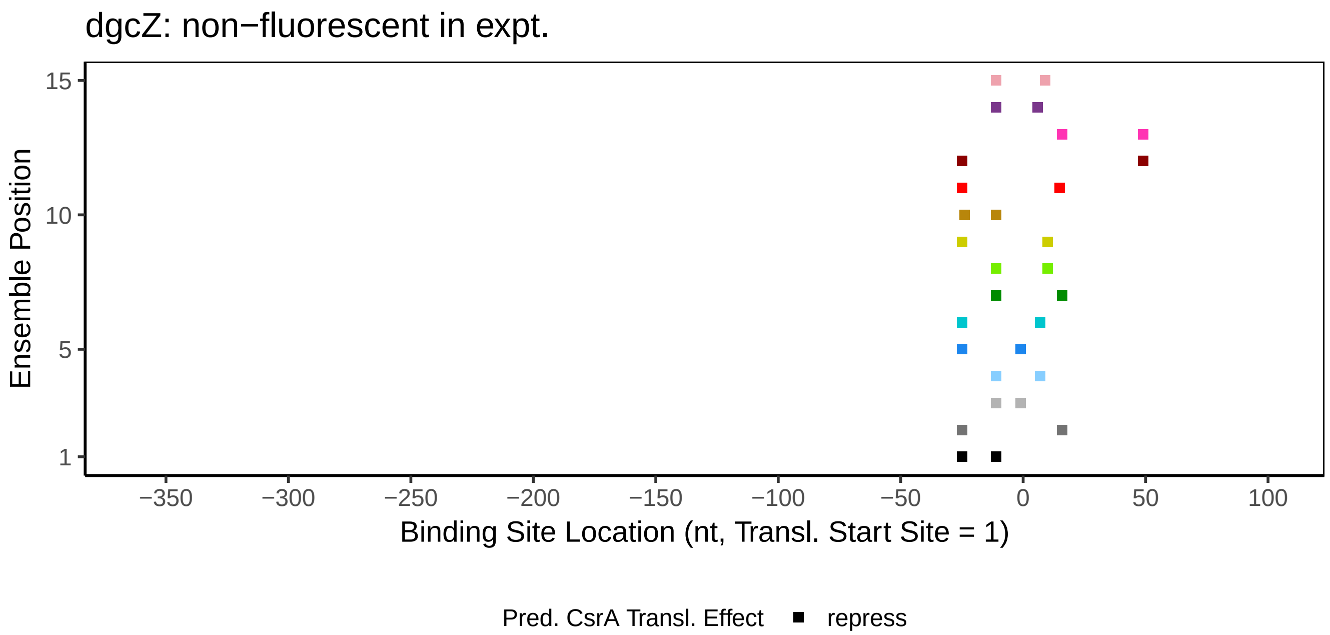

dgcZ: non−fluorescent in expt.
Ensemble Position
Binding Site Location (nt, Transl. Start Site = 1)
Pred. CsrA Transl. Effect
repress

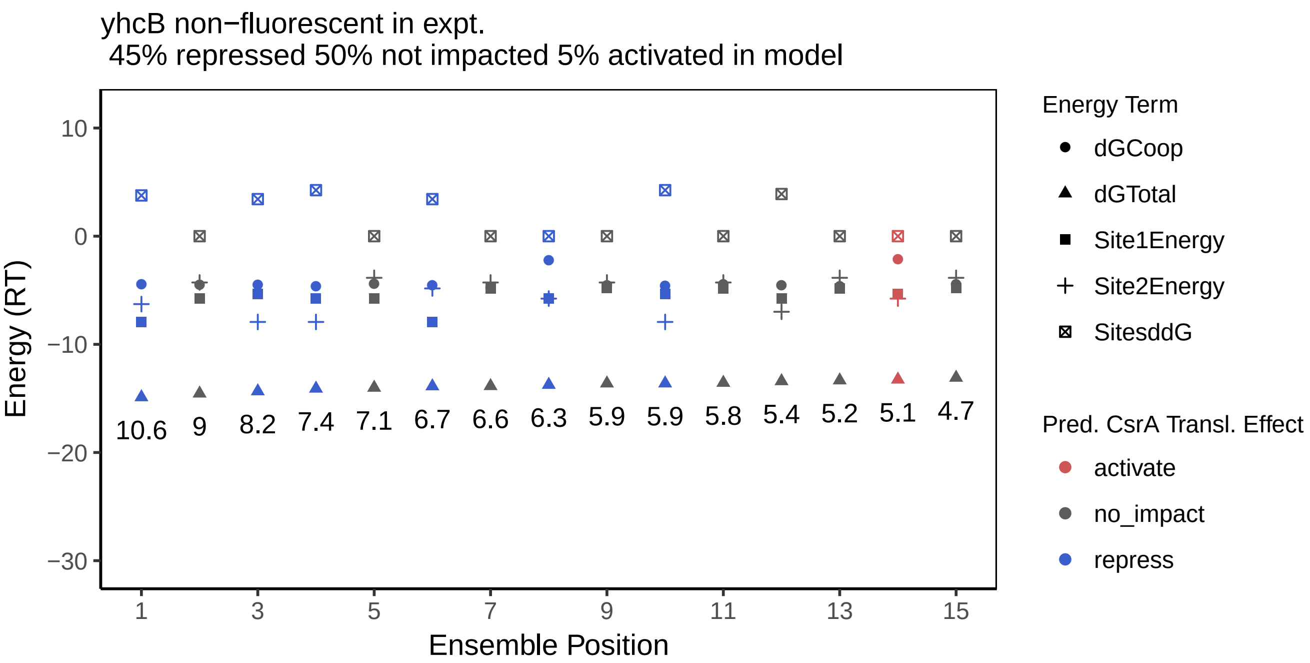
yhcB non−fluorescent in expt.
45% repressed 50% not impacted 5% activated in model
Energy (RT)
Ensemble Position
10.6 9 8.2 7.4 7.1 6.7 6.6 6.3 5.9 5.9 5.8 5.4 5.2 5.1 4.7
Energy Term
dGCoop
dGTotal
Site1Energy
Site2Energy
SitesddG
Pred. CsrA Transl. Effect
activate
no_impact
repress

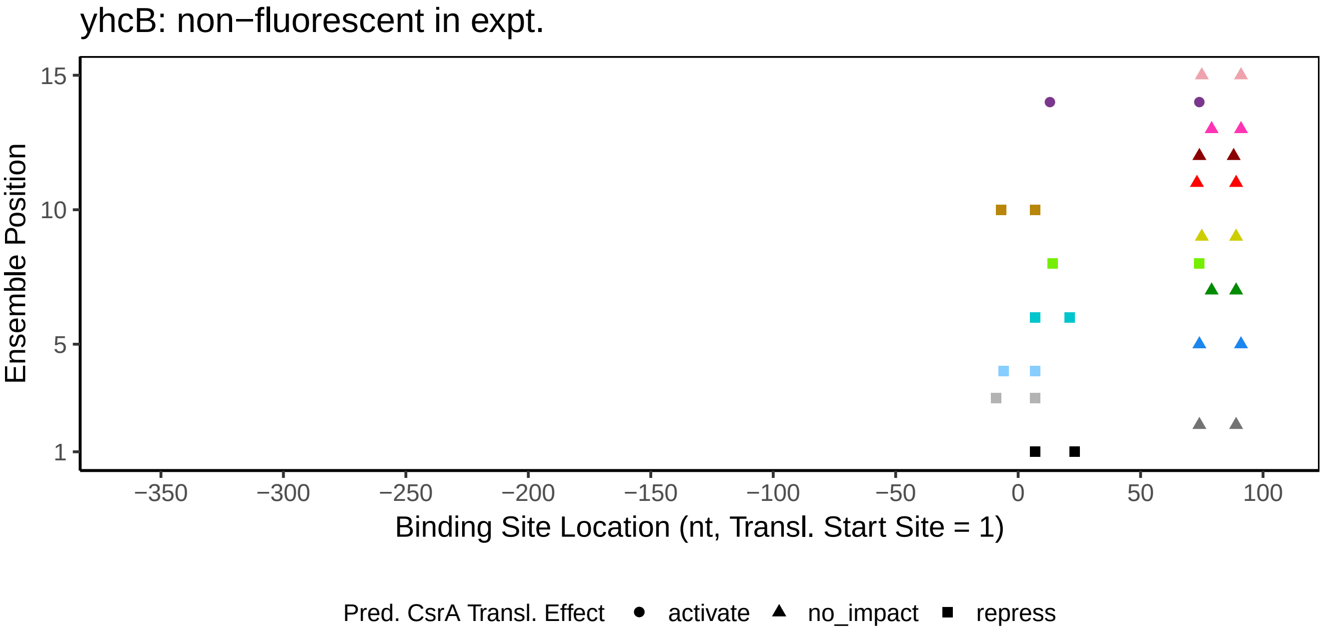
yhcB: non−fluorescent in expt.
Ensemble Position
Binding Site Location (nt, Transl. Start Site = 1)
Pred. CsrA Transl. Effect
activate
no_impact
repress

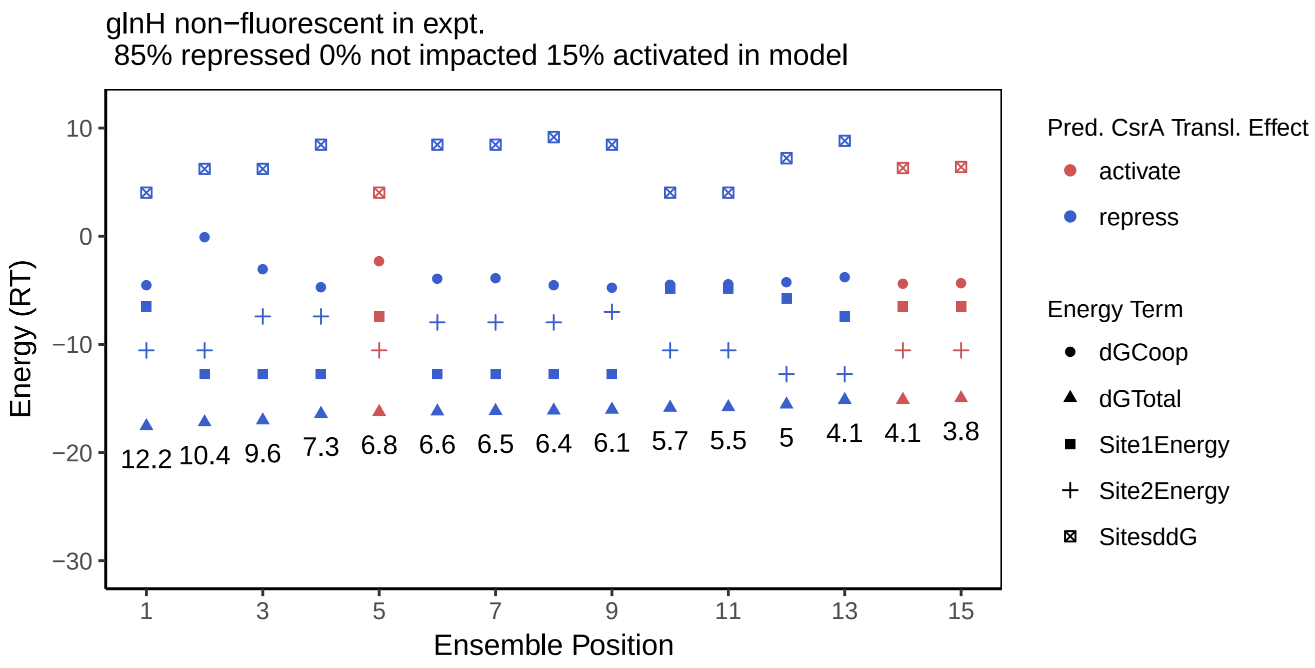

glnH non−fluorescent in expt.
85% repressed 0% not impacted 15% activated in model
Energy (RT)
Ensemble Position
12.2
10.4
9.6
7.3
6.8
6.6
6.5
6.4
6.1
5.7
5.5
5
4.1
4.1
3.8
Pred. CsrA Transl. Effect
activate
repress
Energy Term
dGCoop
dGTotal
Site1Energy
Site2Energy
SitesddG

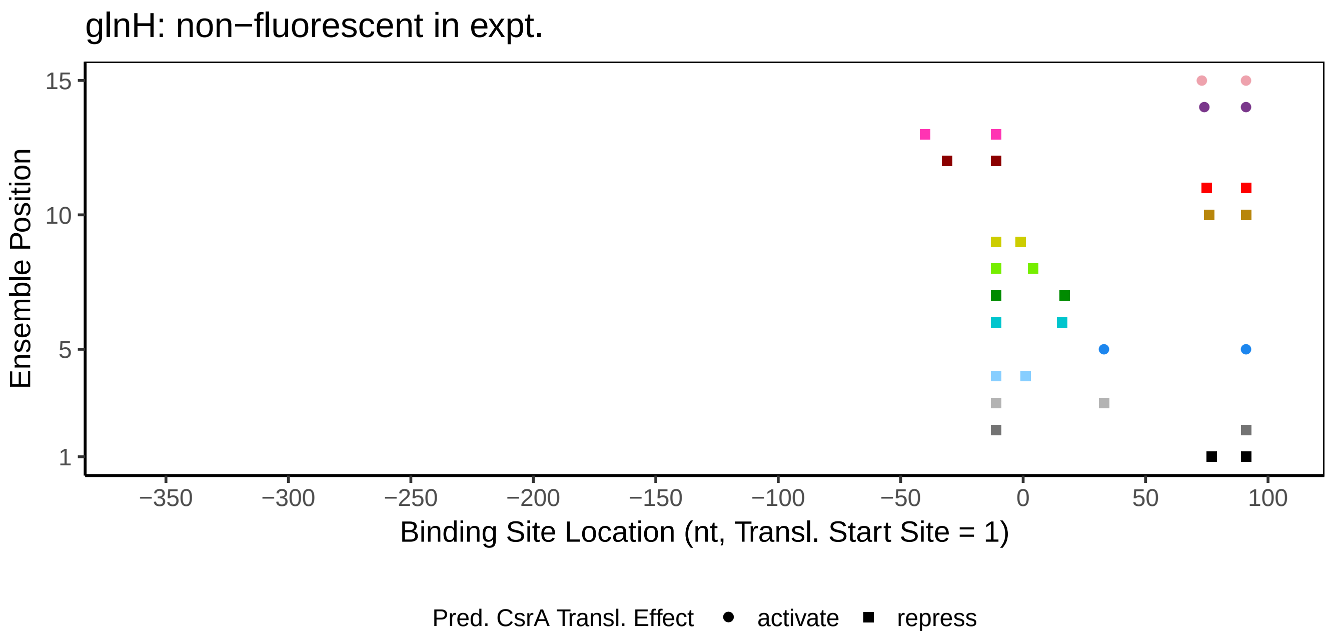

glnH: non−fluorescent in expt.
Ensemble Position
Binding Site Location (nt, Transl. Start Site = 1)
Pred. CsrA Transl. Effect
activate
repress

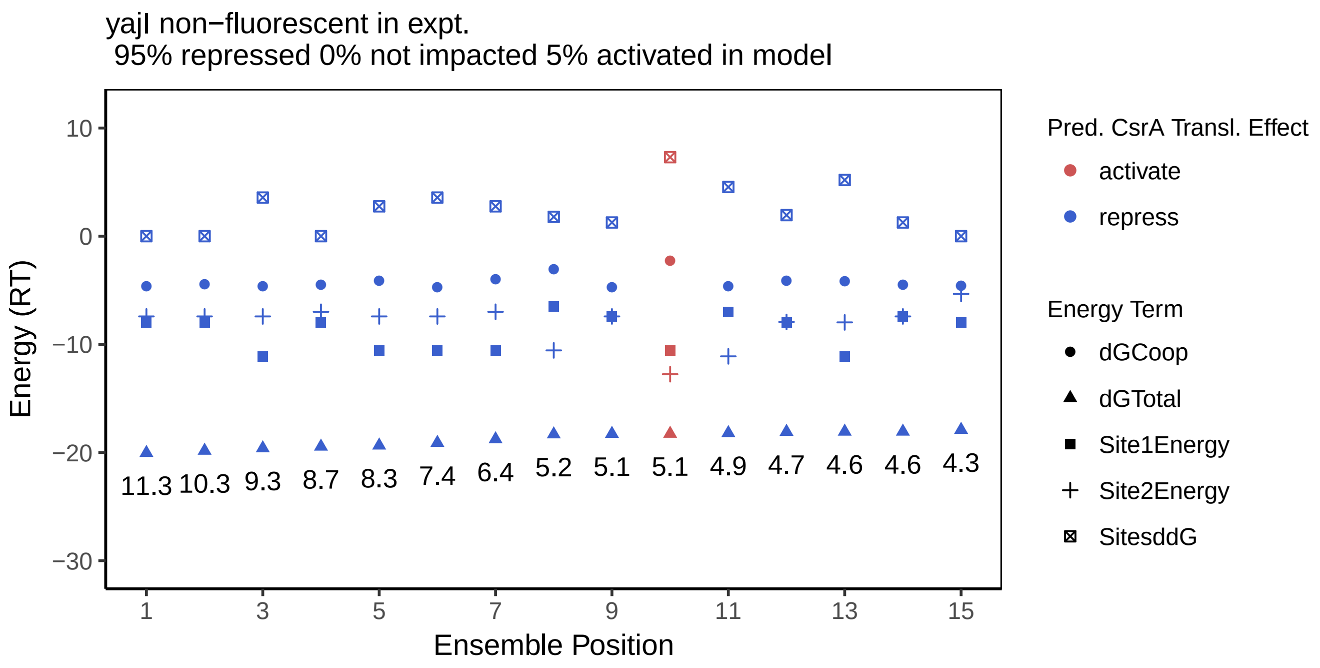
yajI non−fluorescent in expt.
95% repressed 0% not impacted 5% activated in model
Energy (RT)
10
0
−10
−20
−30
11.3 10.3 9.3 8.7 8.3 7.4 6.4 5.2 5.1 5.1 4.9 4.7 4.6 4.6 4.3
1
3
5
7
9
11
13
15
Ensemble Position
Pred. CsrA Transl. Effect
activate
repress
Energy Term
dGCoop
dGTotal
Site1Energy
Site2Energy
SitesddG

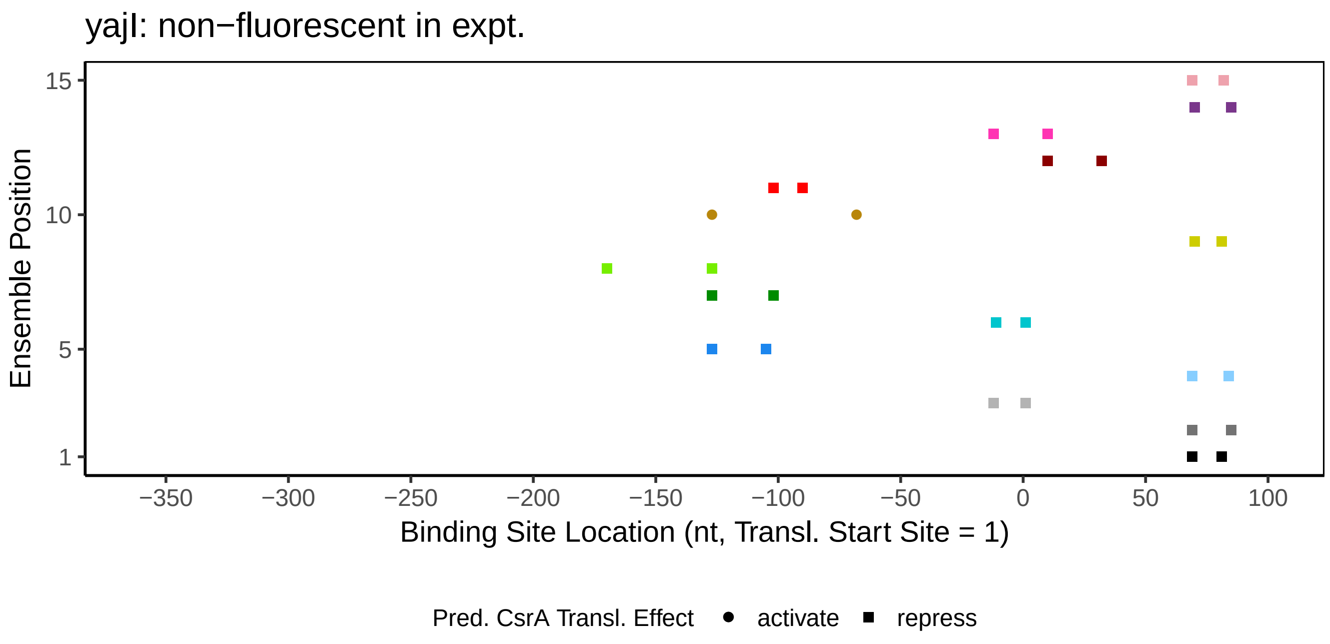
yajI: non−fluorescent in expt.
Ensemble Position
15
10
5
1
−350
−300
−250
−200
−150
−100
−50
0
50
100
Binding Site Location (nt, Transl. Start Site = 1)
Pred. CsrA Transl. Effect
activate
repress

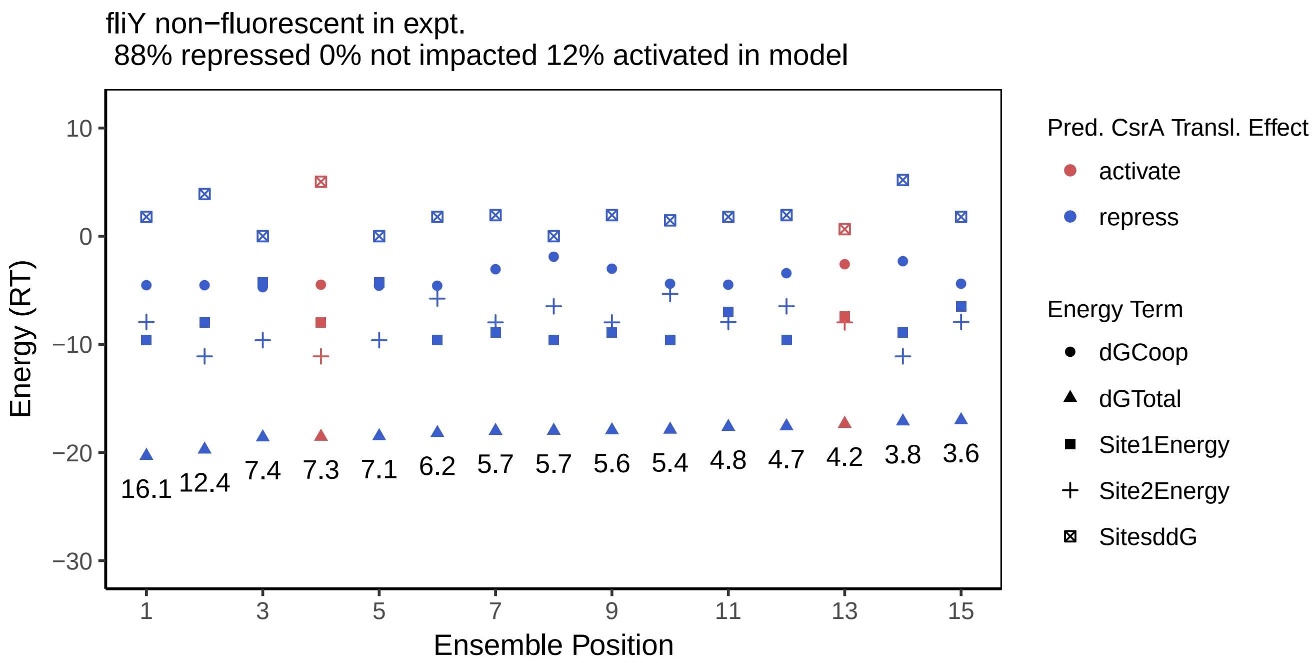

fliY non−fluorescent in expt.
88% repressed 0% not impacted 12% activated in model
Energy (RT)
10
0
−10
−20
−30
16.1
12.4
7.4
7.3
7.1
6.2
5.7
5.7
5.6
5.4
4.8
4.7
4.2
3.8
3.6
1
3
5
7
9
11
13
15
Ensemble Position
Pred. CsrA Transl. Effect
activate
repress
Energy Term
dGCoop
dGTotal
Site1Energy
Site2Energy
SitesddG

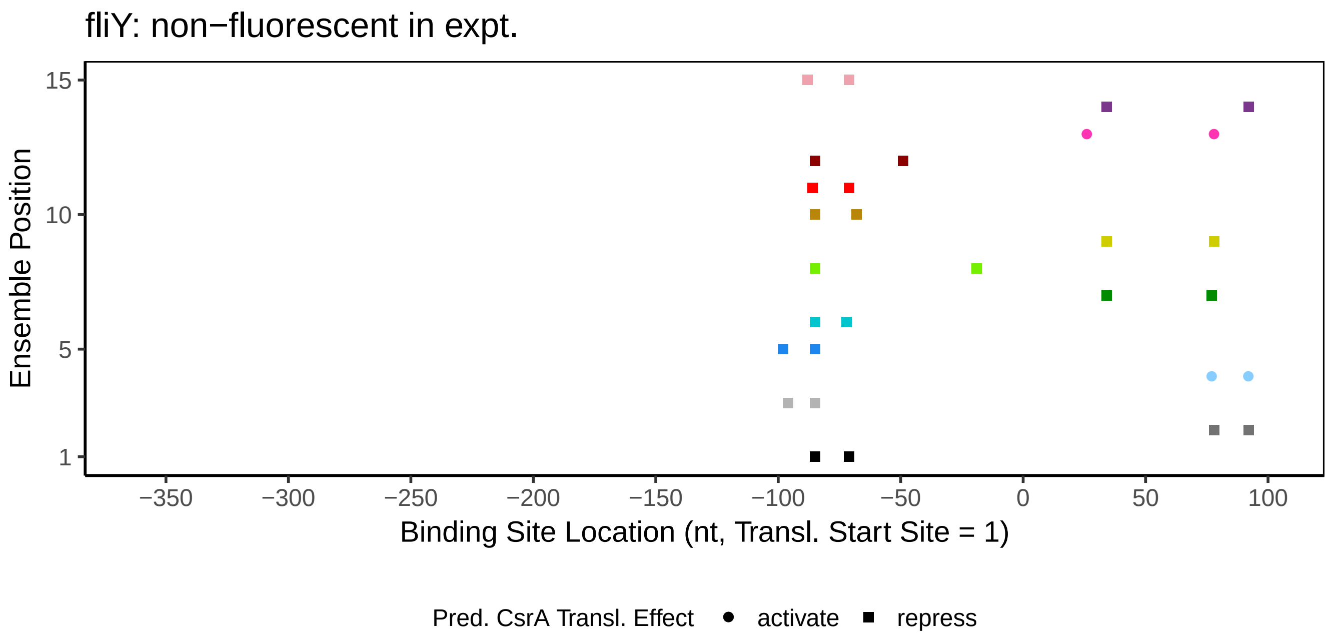

fliY: non−fluorescent in expt.
Ensemble Position
15
10
5
1
−350
−300
−250
−200
−150
−100
−50
0
50
100
Binding Site Location (nt, Transl. Start Site = 1)
Pred. CsrA Transl. Effect
activate
repress

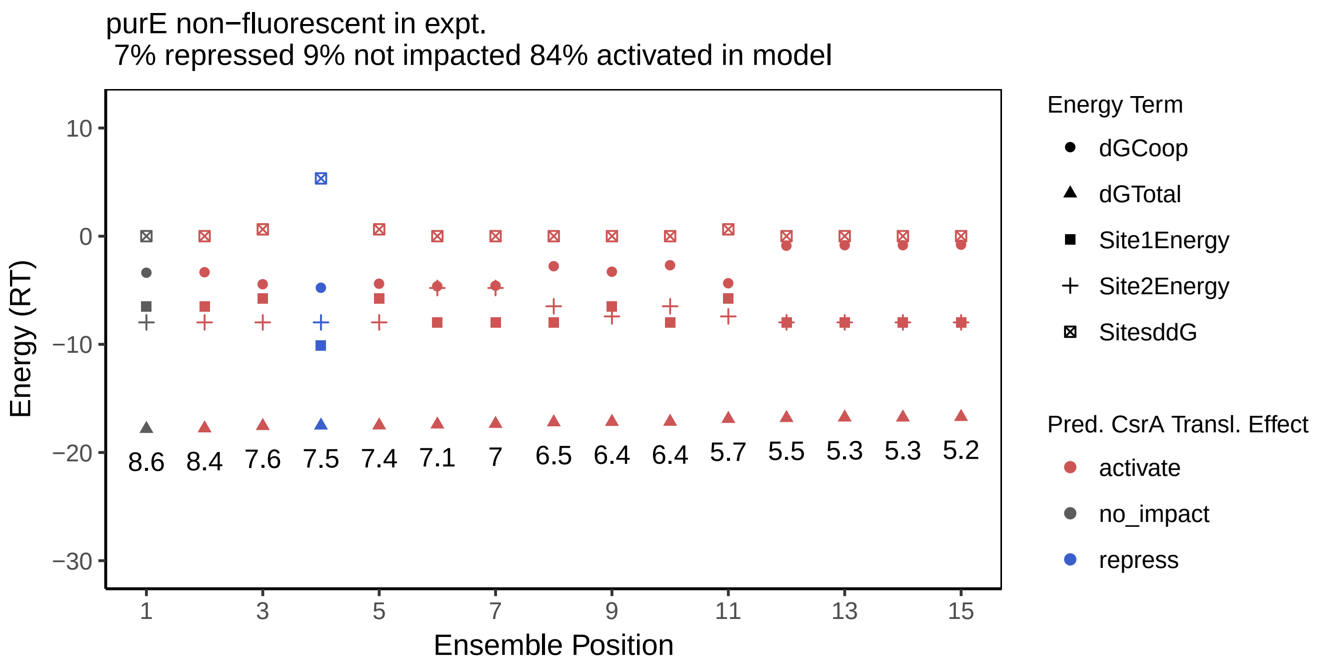

purE non−fluorescent in expt.
7% repressed 9% not impacted 84% activated in model
Energy (RT)
Ensemble Position
8.6 8.4 7.6 7.5 7.4 7.1 7 6.5 6.4 6.4 5.7 5.5 5.3 5.3 5.2
Energy Term
dGCoop
dGTotal
Site1Energy
Site2Energy
SitesddG
Pred. CsrA Transl. Effect
activate
no_impact
repress

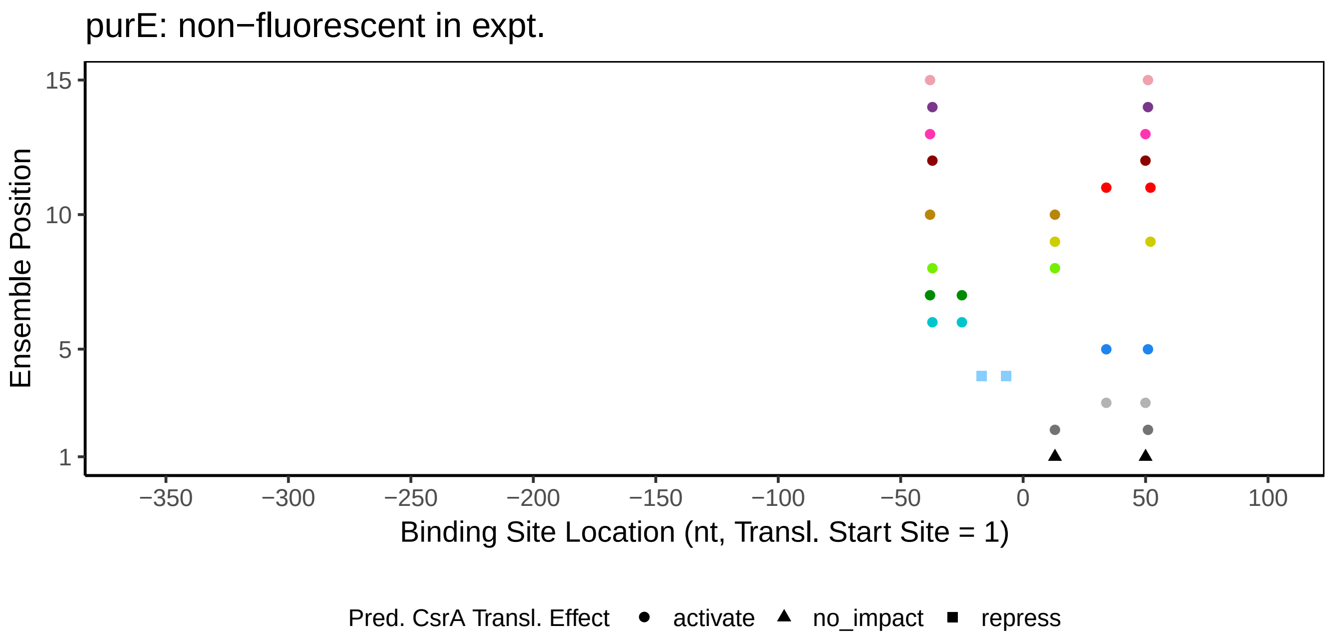

purE: non−fluorescent in expt.
Ensemble Position
Binding Site Location (nt, Transl. Start Site = 1)
Pred. CsrA Transl. Effect
activate
no_impact
repress

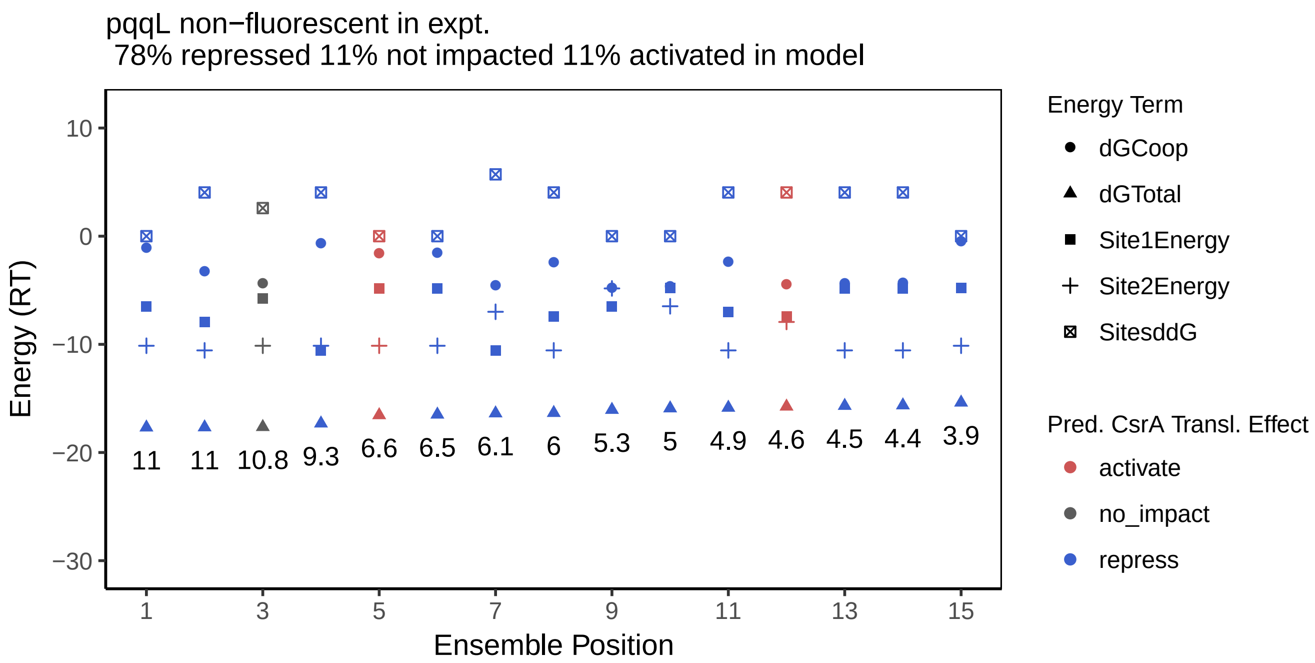
pqqL non−fluorescent in expt.
78% repressed 11% not impacted 11% activated in model
Energy (RT)
Ensemble Position
11
11
10.8
9.3
6.6
6.5
6.1
6
5.3
5
4.9
4.6
4.5
4.4
3.9
Energy Term
dGCoop
dGTotal
Site1Energy
Site2Energy
SitesddG
Pred. CsrA Transl. Effect
activate
no_impact
repress

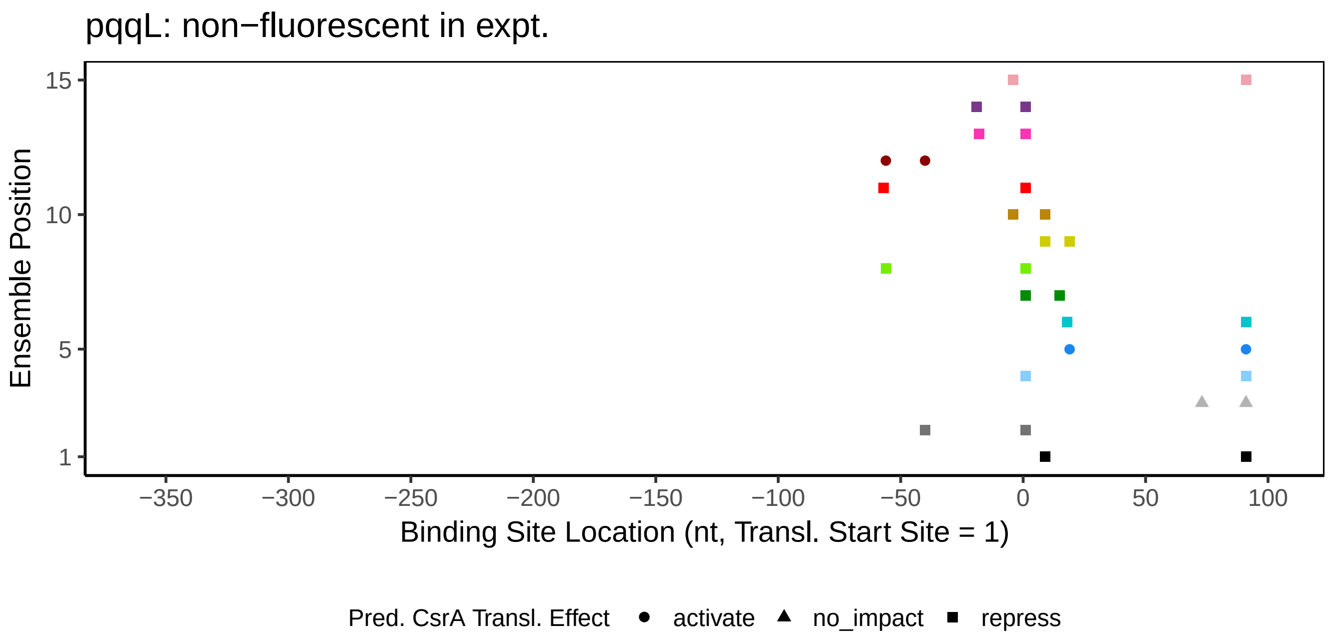
pqqL: non−fluorescent in expt.
Ensemble Position
Binding Site Location (nt, Transl. Start Site = 1)
Pred. CsrA Transl. Effect
activate
no_impact
repress

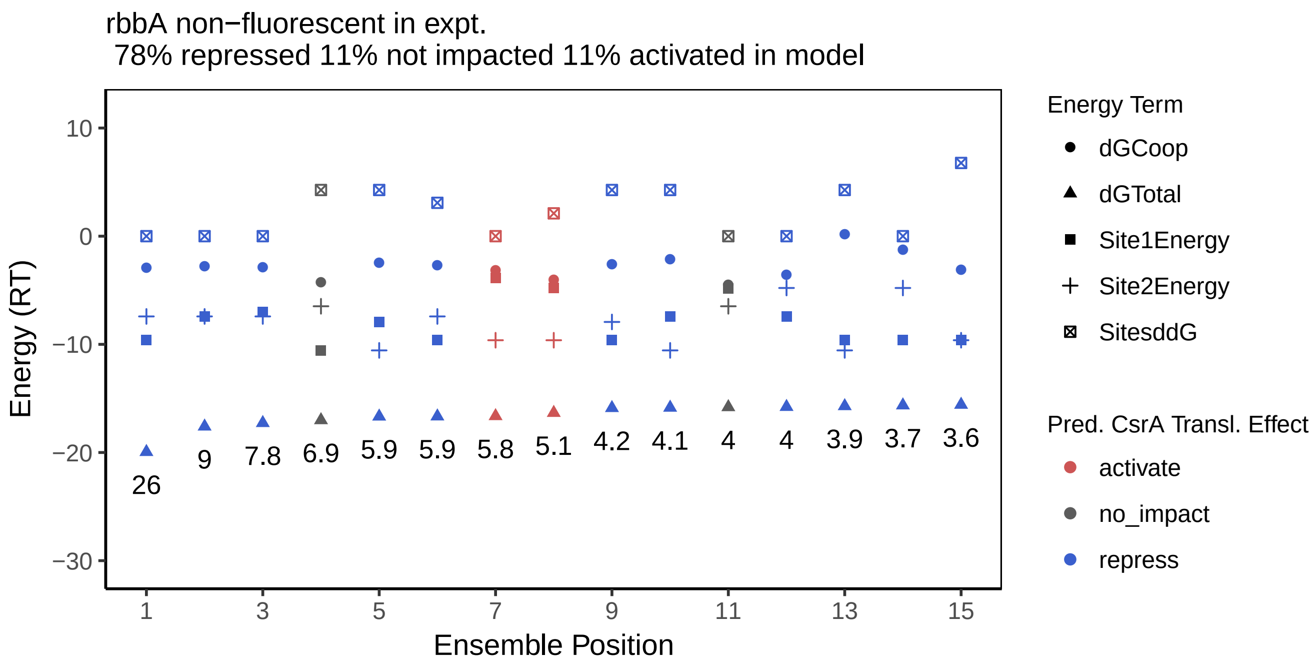
rbbA non−fluorescent in expt.
78% repressed 11% not impacted 11% activated in model
Energy (RT)
Ensemble Position
26
9
7.8
6.9
5.9
5.9
5.8
5.1
4.2
4.1
4
4
3.9
3.7
3.6
Energy Term
dGCoop
dGTotal
Site1Energy
Site2Energy
SitesddG
Pred. CsrA Transl. Effect
activate
no_impact
repress

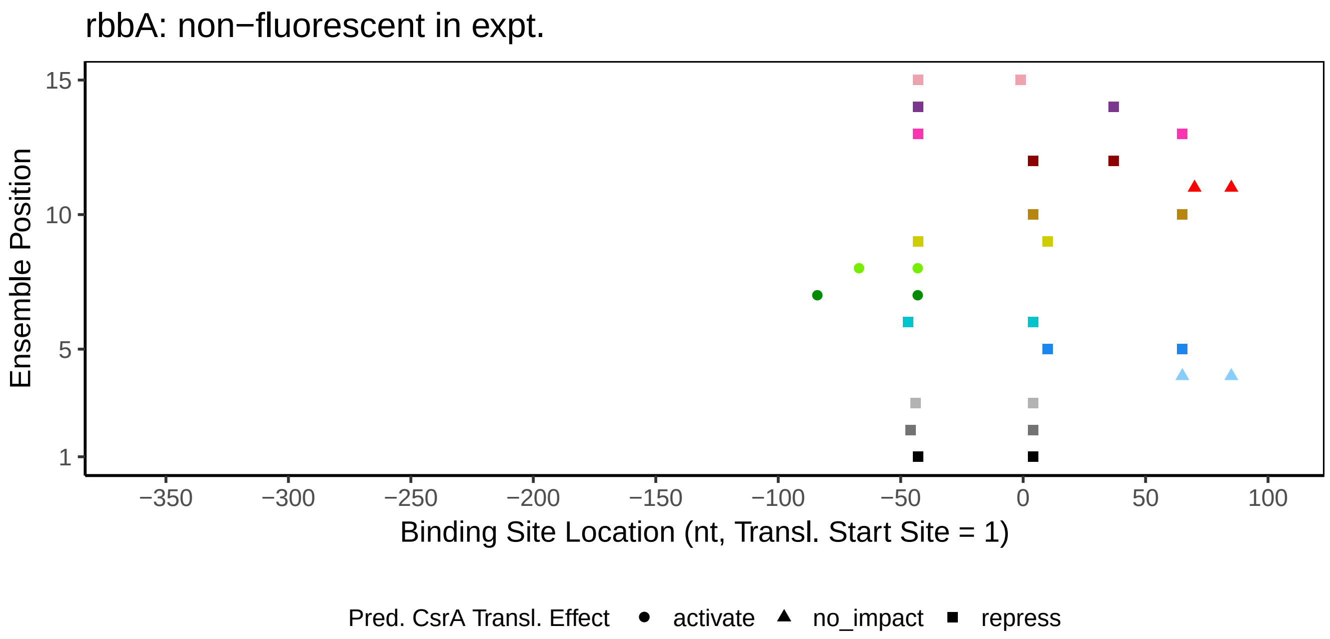
rbbA: non−fluorescent in expt.
Ensemble Position
Binding Site Location (nt, Transl. Start Site = 1)
Pred. CsrA Transl. Effect
activate
no_impact
repress

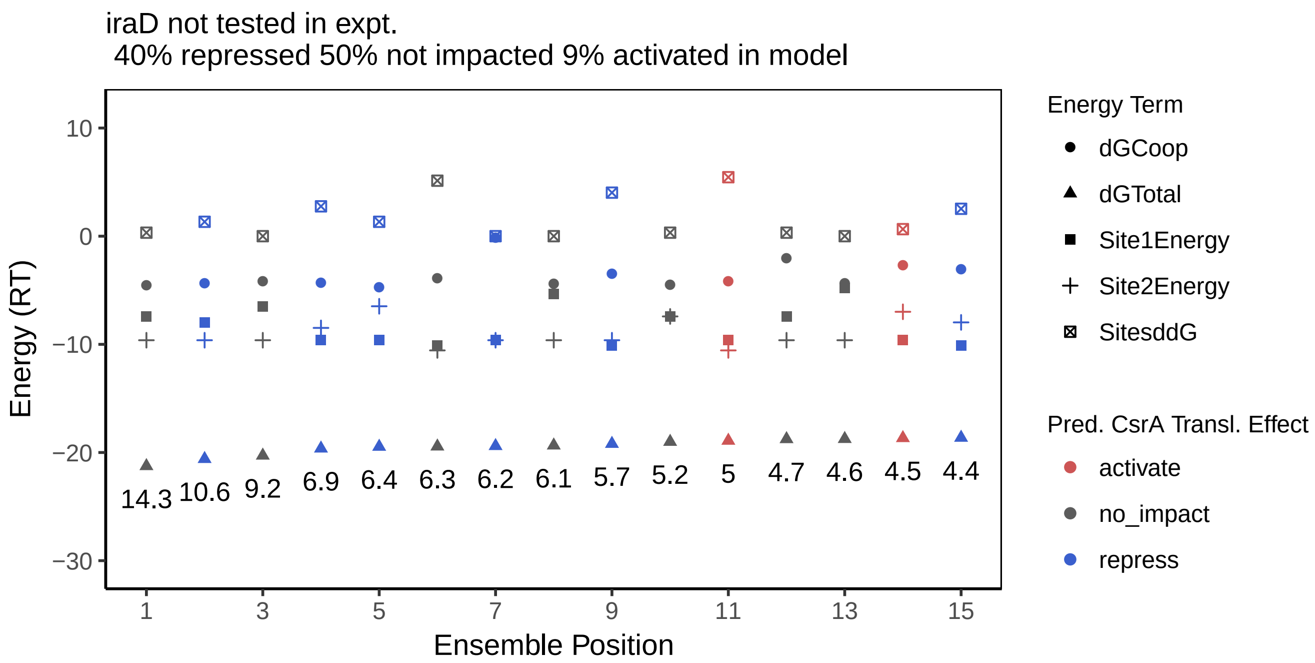
iraD not tested in expt.
40% repressed 50% not impacted 9% activated in model
Energy (RT)
10
0
−10
−20
−30
14.3
10.6
9.2
6.9
6.4
6.3
6.2
6.1
5.7
5.2
5
4.7
4.6
4.5
4.4
1
3
5
7
9
11
13
15
Ensemble Position
Energy Term
dGCoop
dGTotal
Site1Energy
Site2Energy
SitesddG
Pred. CsrA Transl. Effect
activate
no_impact
repress

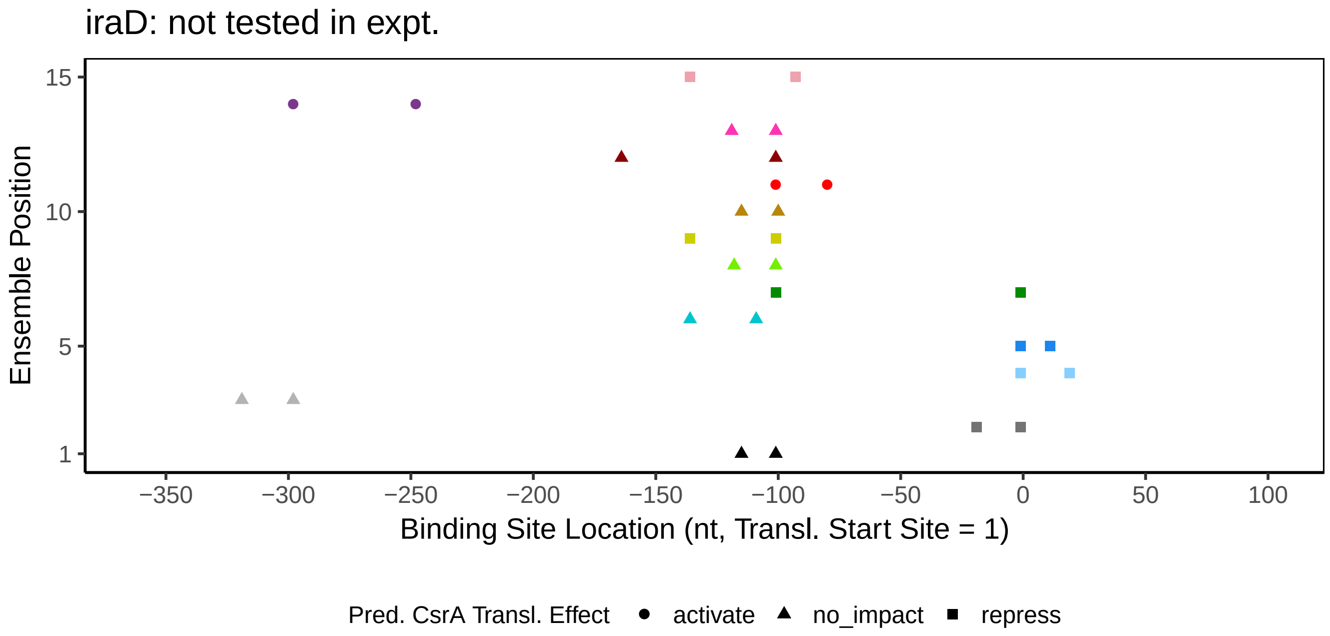
iraD: not tested in expt.
Ensemble Position
15
10
5
1
−350
−300
−250
−200
−150
−100
−50
0
50
100
Binding Site Location (nt, Transl. Start Site = 1)
Pred. CsrA Transl. Effect
activate
no_impact
repress

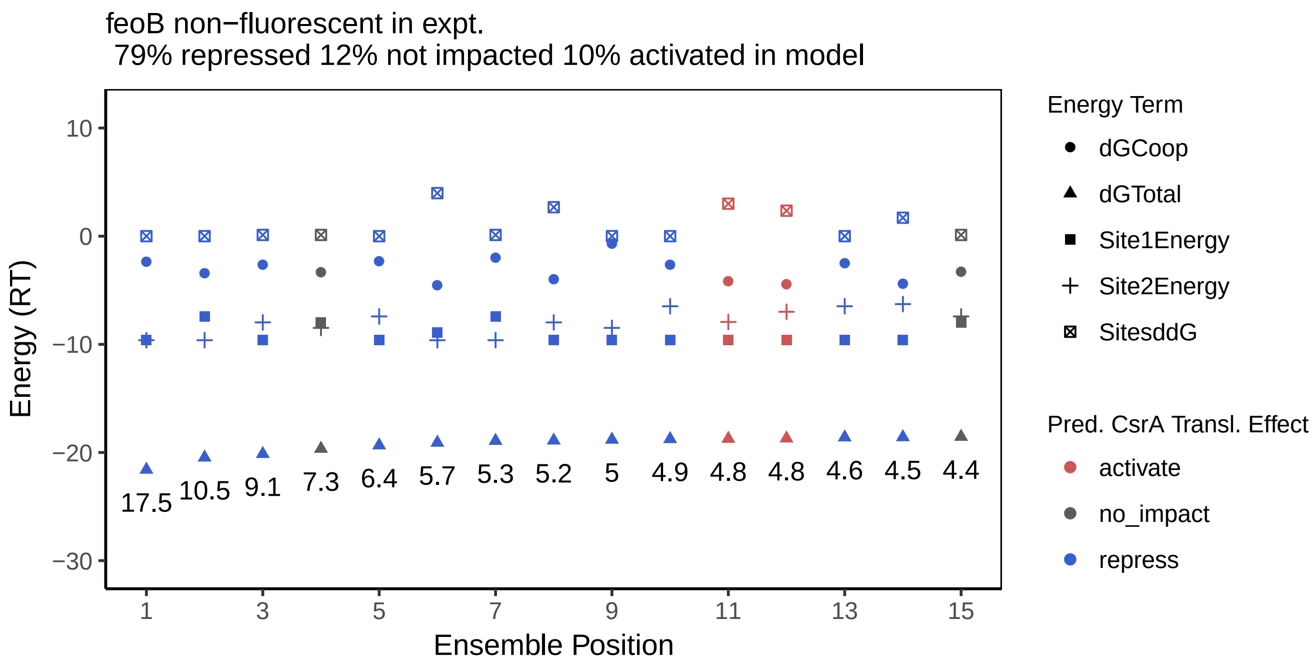
feoB non−fluorescent in expt.
79% repressed 12% not impacted 10% activated in model
Energy (RT)
10
0
−10
−20
−30
17.5
10.5
9.1
7.3
6.4
5.7
5.3
5.2
5
4.9
4.8
4.8
4.6
4.5
4.4
1
3
5
7
9
11
13
15
Ensemble Position
Energy Term
dGCoop
dGTotal
Site1Energy
Site2Energy
SitesddG
Pred. CsrA Transl. Effect
activate
no_impact
repress

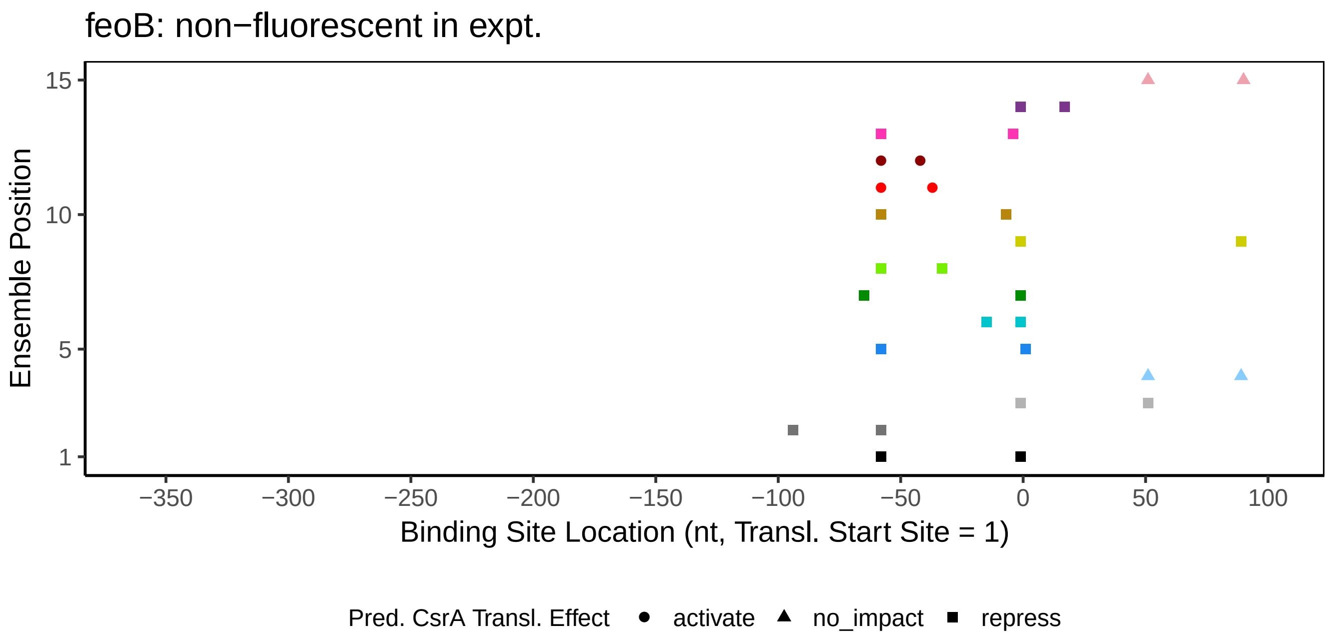
feoB: non−fluorescent in expt.
Ensemble Position
15
10
5
1
−350
−300
−250
−200
−150
−100
−50
0
50
100
Binding Site Location (nt, Transl. Start Site = 1)
Pred. CsrA Transl. Effect
activate
no_impact
repress

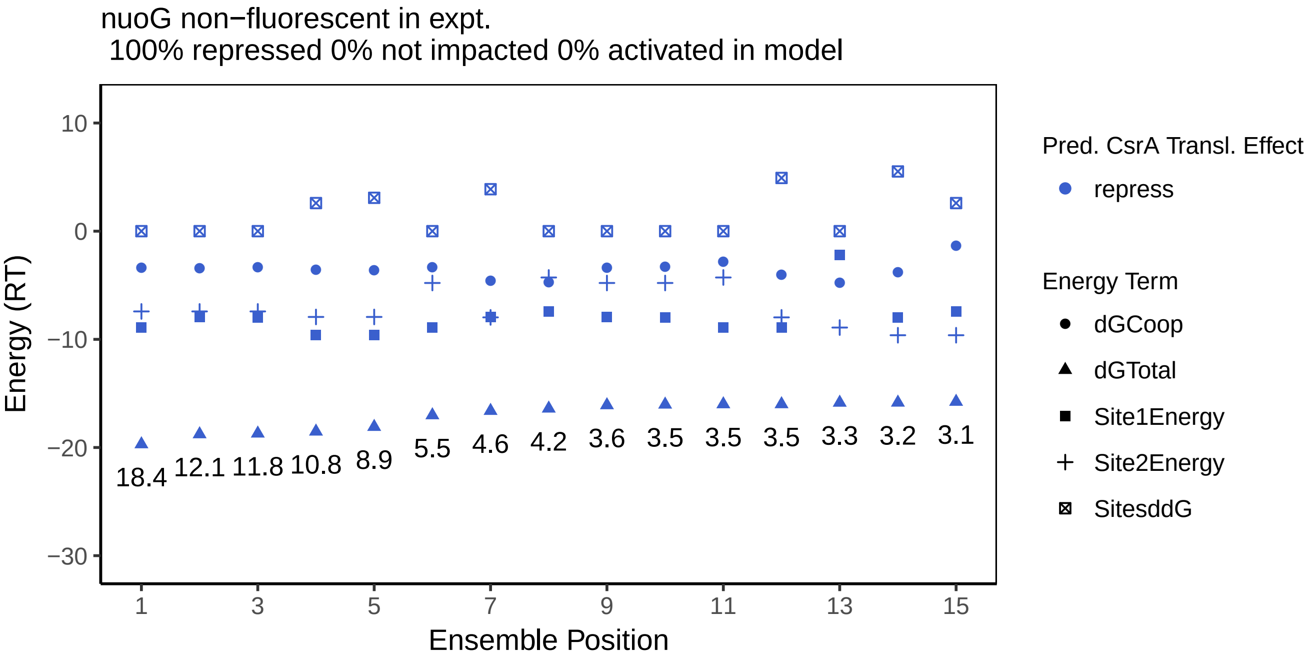
nuoG non−fluorescent in expt.
100% repressed 0% not impacted 0% activated in model
Energy (RT)
10
0
−10
−20
−30
18.4 12.1 11.8 10.8 8.9 5.5 4.6 4.2 3.6 3.5 3.5 3.5 3.3 3.2 3.1
1 3 5 7 9 11 13 15
Ensemble Position
Pred. CsrA Transl. Effect
repress
Energy Term
dGCoop
dGTotal
Site1Energy
Site2Energy
SitesddG

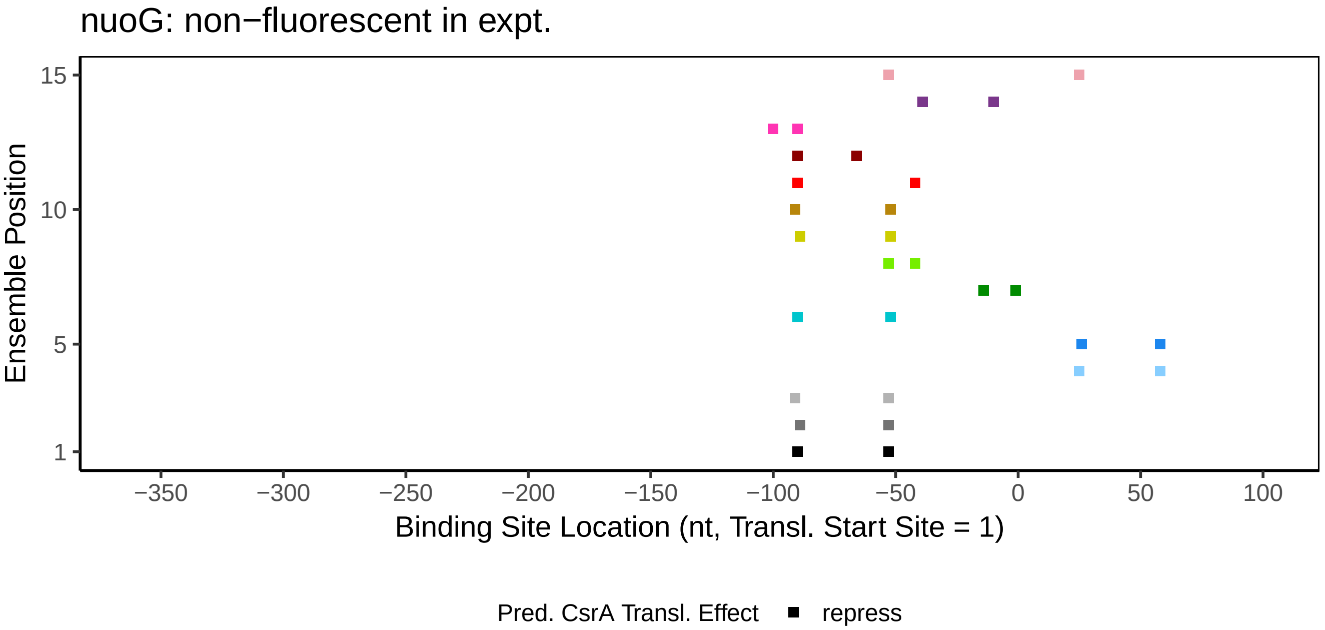
nuoG: non−fluorescent in expt.
Ensemble Position
15
10
5
1
−350 −300 −250 −200 −150 −100 −50 0 50 100
Binding Site Location (nt, Transl. Start Site = 1)
Pred. CsrA Transl. Effect
repress

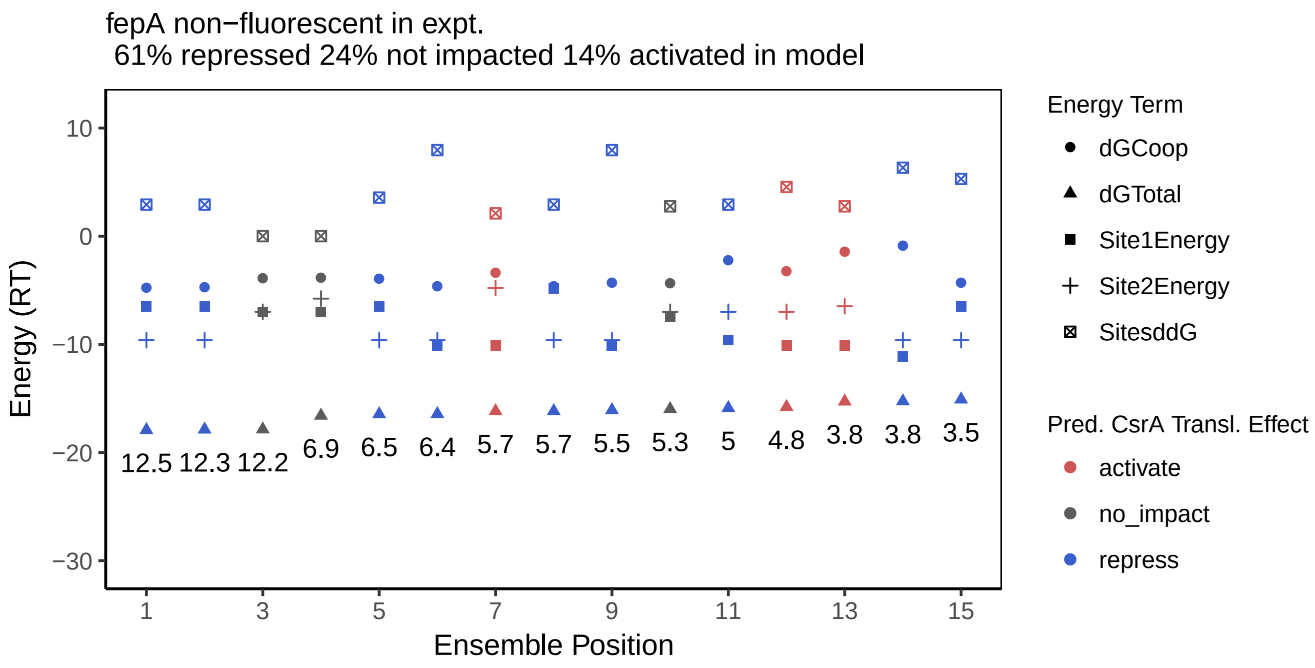

fepA non−fluorescent in expt.
61% repressed 24% not impacted 14% activated in model
Energy (RT)
10
0
−10
−20
−30
12.5 12.3 12.2 6.9 6.5 6.4 5.7 5.7 5.5 5.3 5 4.8 3.8 3.8 3.5
1 3 5 7 9 11 13 15
Ensemble Position
Energy Term
dGCoop
dGTotal
Site1Energy
Site2Energy
SitesddG
Pred. CsrA Transl. Effect
activate
no_impact
repress

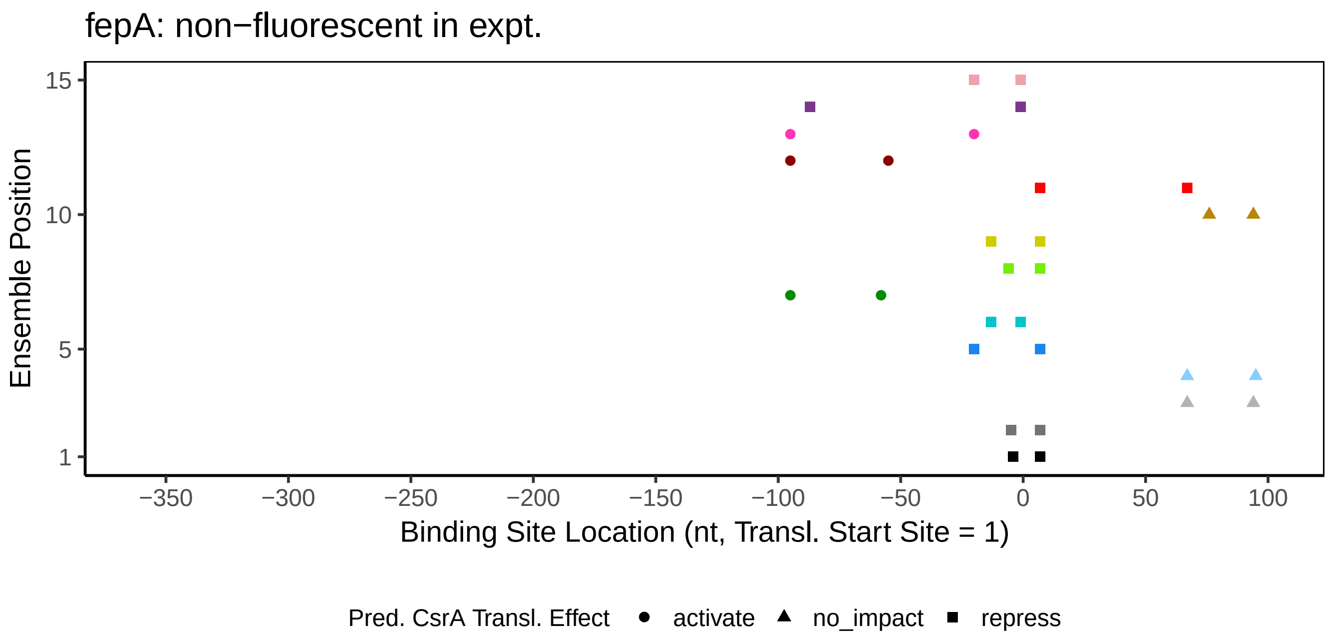

fepA: non−fluorescent in expt.
Ensemble Position
15
10
5
1
−350 −300 −250 −200 −150 −100 −50 0 50 100
Binding Site Location (nt, Transl. Start Site = 1)
Pred. CsrA Transl. Effect activate no_impact repress

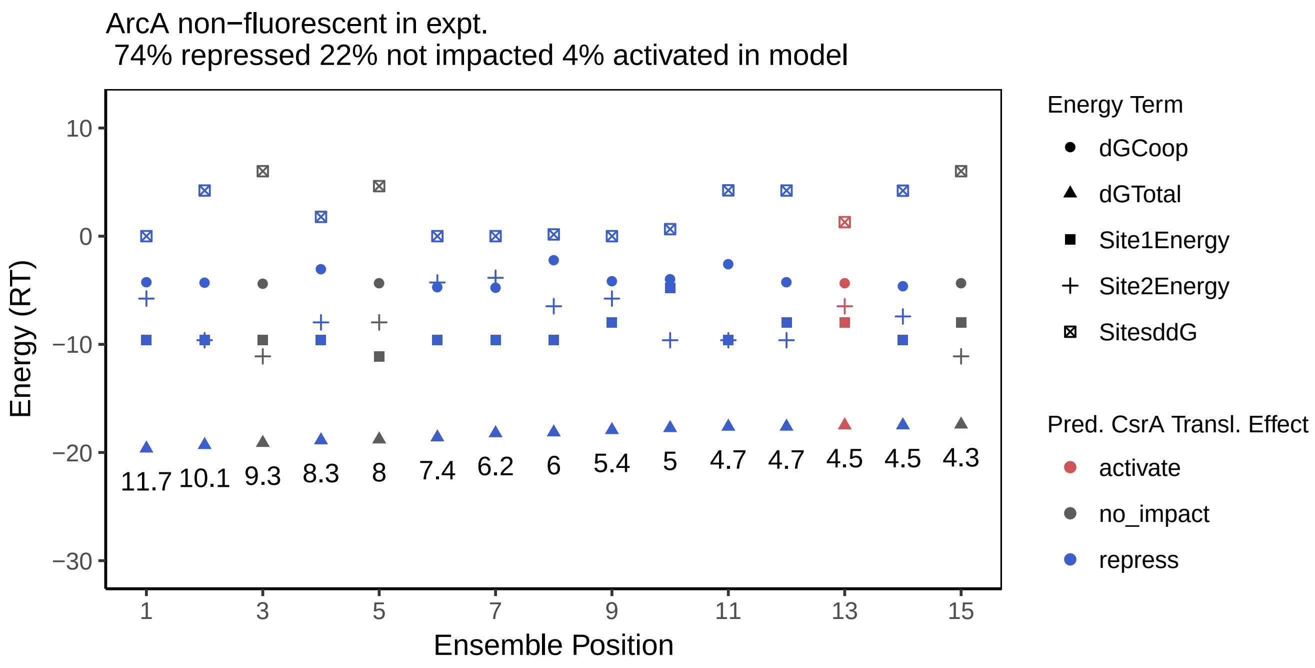
ArcA non−fluorescent in expt.
74% repressed 22% not impacted 4% activated in model
Energy (RT)
Ensemble Position
11.7 10.1 9.3 8.3 8 7.4 6.2 6 5.4 5 4.7 4.7 4.5 4.5 4.3
Energy Term
dGCoop
dGTotal
Site1Energy
Site2Energy
SitesddG
Pred. CsrA Transl. Effect
activate
no_impact
repress

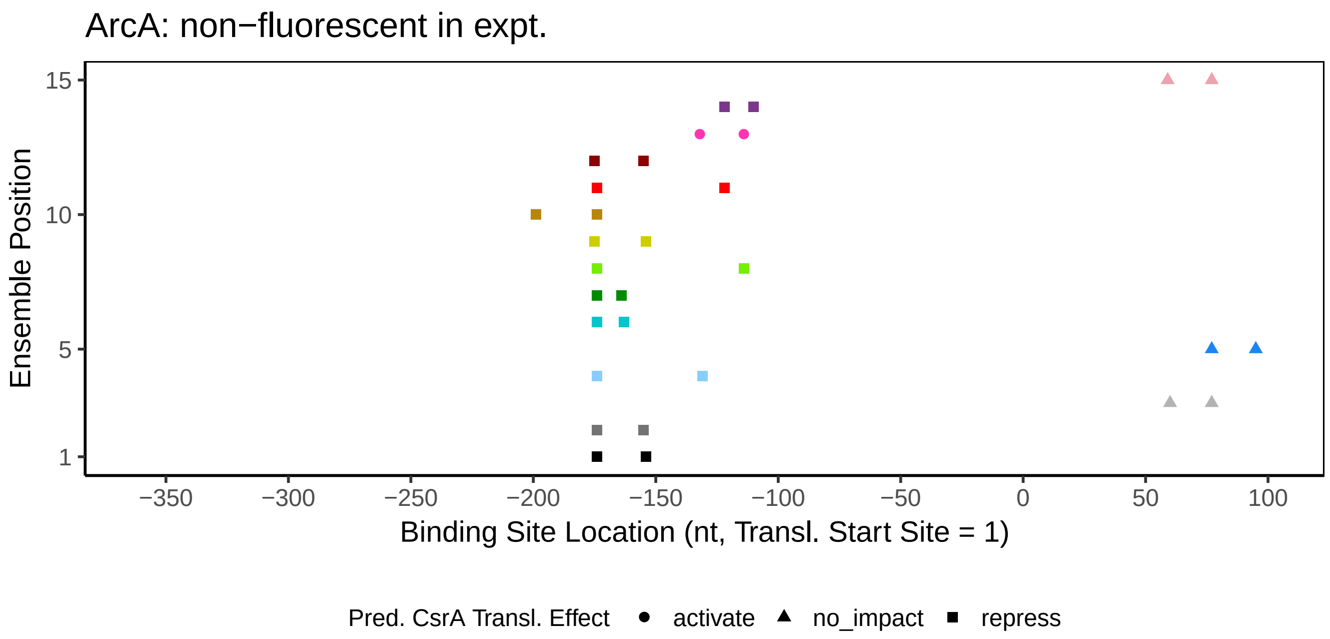
ArcA: non−fluorescent in expt.
Ensemble Position
Binding Site Location (nt, Transl. Start Site = 1)
Pred. CsrA Transl. Effect
activate
no_impact
repress

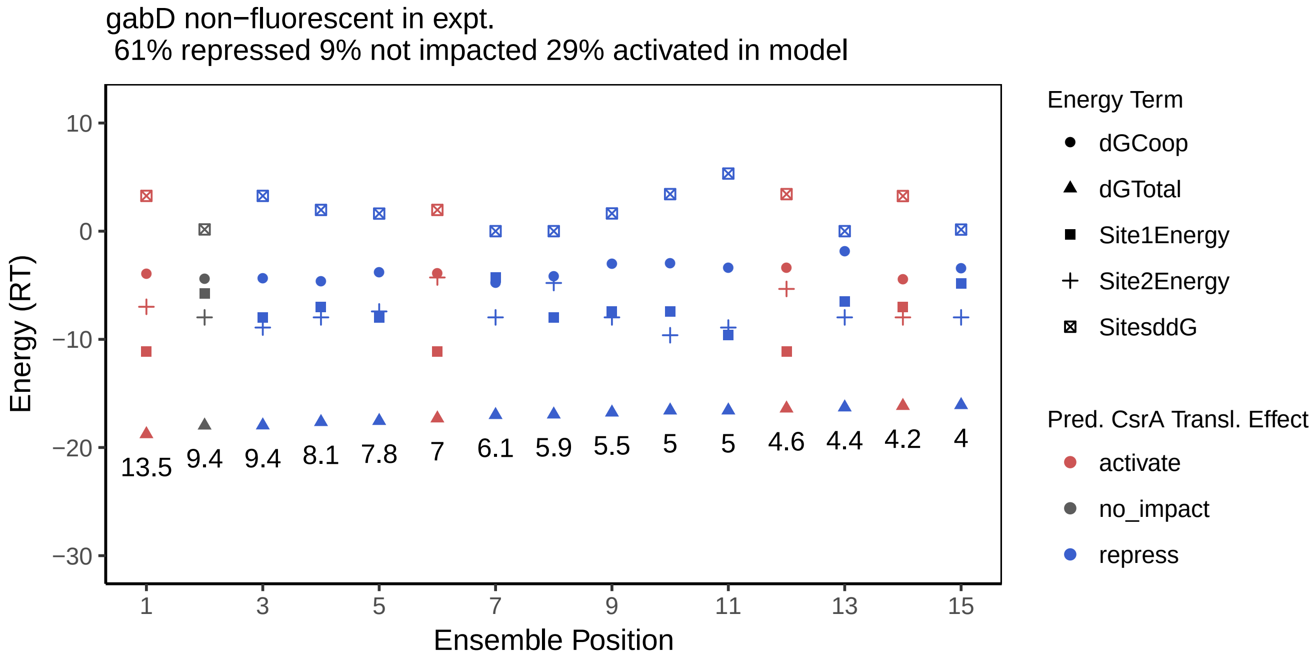
gabD non−fluorescent in expt.
61% repressed 9% not impacted 29% activated in model
Energy (RT)
10
0
−10
−20
−30
13.5
9.4
9.4
8.1
7.8
7
6.1
5.9
5.5
5
5
4.6
4.4
4.2
4
1
3
5
7
9
11
13
15
Ensemble Position
Energy Term
dGCoop
dGTotal
Site1Energy
Site2Energy
SitesddG
Pred. CsrA Transl. Effect
activate
no_impact
repress

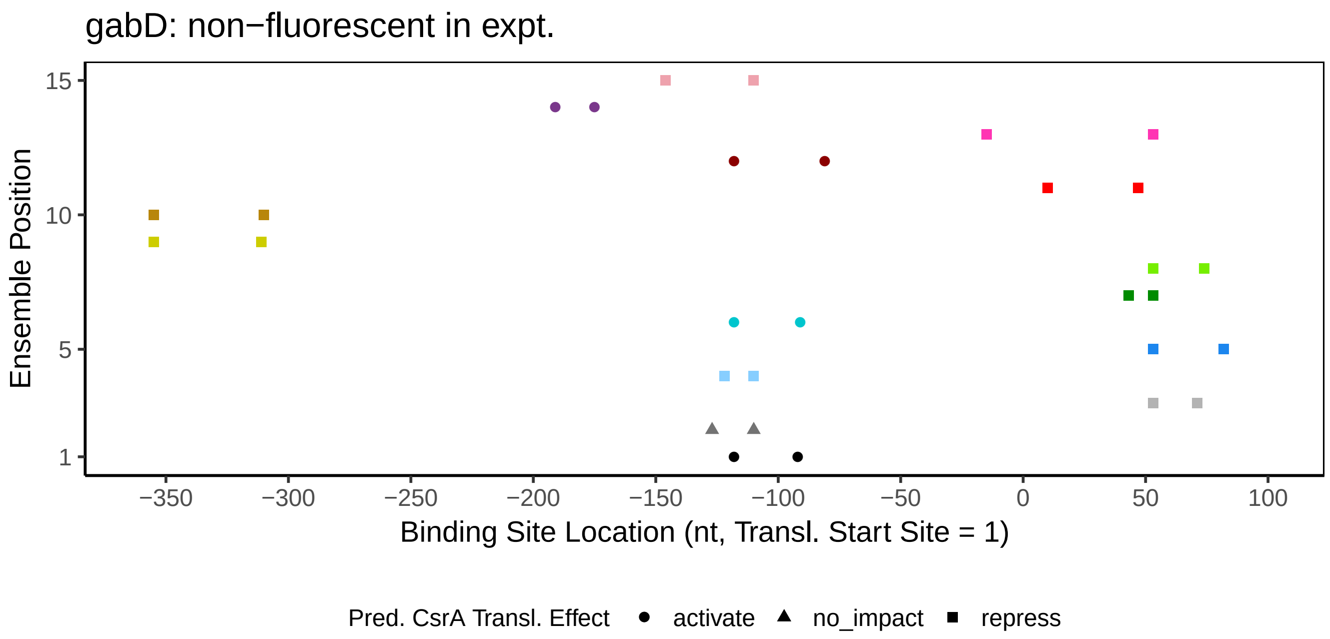
gabD: non−fluorescent in expt.
Ensemble Position
15
10
5
1
−350
−300
−250
−200
−150
−100
−50
0
50
100
Binding Site Location (nt, Transl. Start Site = 1)
Pred. CsrA Transl. Effect
activate
no_impact
repress
